# Supplementary material for: Sex- and region-specific cortical and hippocampal whole genome transcriptome profiles from control and APP/PS1 Alzheimer’s disease mice
Source: PLoS One. 2024 Feb 7;19(2):e0296959. doi: 10.1371/journal.pone.0296959 (PMC10849391; doi:10.1371/journal.pone.0296959)
Supplement: S1 File — S1 Fig: Genotyping of APP/PS1 AD mice and WT control animals. S2 Fig: 3D image of the murine brain including the RS cortex and hippocampus (BROIs) used for transcriptome analysis in our study. S3 Fig: PCA of transcriptomes from the RS cortex and hippocampus of WT controls and APP/PS1 AD mice of both sexes. S4 Fig: Hierarchical clustering of transcriptome data from the RS cortex and hippocampus of WT control and APP/PS1 AD mice of both sexes. S5 Fig: Bar diagrams of the top 30 candidates of DEGs with highest significant FCs (FC > 1.5 and FC < -1.5, p < 0.05). S6 Fig: Pathway analysis of intersectional and signature gene sets in APP/PS1 subgroups. S7 Fig: Comparative qPCR analysis of selected gene transcript levels from the hippocampus of female and male APP/PS1 AD with 5XFAD mice. S1 Table: PCR reaction set-up using PCR Mastermix and genomic DNA. S2 Table: Materials used for one-color microarray-based gene expression data collection. S3 Table: Software used for one-color microarray-based gene expression data collection. S4 Table: Details on genes, forward and reverse primer sequences and annealing temperatures relevant for qPCR experimentation. S5 Table: Characteristics of DEGs in the RS cortex of female APP/PS1 AD mice. S6 Table: Characteristics of DEGs in the hippocampus of female APP/PS1 AD mice. S7 Table: Characteristics of DEGs in the RS cortex of male APP/PS1 AD mice. S8 Table: Characteristics of DEGs in the hippocampus of male APP/PS1 AD mice. S9 Table: Venn analysis of DEGs in the RS cortex and hippocampus of female APP/PS1 AD mice. S10 Table: Venn analysis of DEGs genes in the RS cortex and hippocampus of male APP/PS1 AD mice. S11 Table: Venn analysis of DEGs in the RS cortex of male and female APP/PS1 AD mice. S12 Table: Venn analysis of DEGs in the hippocampus of male and female APP/PS1 AD mice. S13 Table: Differentially regulated l(i)ncRNAs in APP/PS1 AD vs. WT mice. S14 Table: qPCR-based FC analysis of selected genes in the hippocampus of APP/PS1 AD vs. [file pone.0296959.s001.zip › Supplementary Files_R1/Supplementary Figure 6_Pathways_downreg genes/Signature_down_DEGs_female_Hip_APPPS1/Pathway analysis report.pdf]

Pathway Analysis Report

This report contains the pathway analysis results for the submitted sample ''. Analysis was performed against Reactome version 85 on 17/08/2023. The web link to these results is:

https://reactome.org/PathwayBrowser/#/ANALYSIS=MjAyMzA4MTcwNjQzNThfMjExOTg%3D

Please keep in mind that analysis results are temporarily stored on our server. The storage period depends on usage of the service but is at least 7 days. As a result, please note that this URL is only valid for a limited time period and it might have expired.

Table of Contents

1. Introduction
2. Properties
3. Genome-wide overview
4. Most significant pathways
5. Pathways details
6. Identifiers found
7. Identifiers not found

1. Introduction

Reactome is a curated database of pathways and reactions in human biology. Reactions can be considered as pathway 'steps'. Reactome defines a 'reaction' as any event in biology that changes the state of a biological molecule. Binding, activation, translocation, degradation and classical biochemical events involving a catalyst are all reactions. Information in the database is authored by expert biologists, entered and maintained by Reactome’s team of curators and editorial staff. Reactome content frequently cross-references other resources e.g. NCBI, Ensembl, UniProt, KEGG (Gene and Compound), ChEBI, PubMed and GO. Orthologous reactions inferred from annotation for Homo sapiens are available for 14 non-human species including mouse, rat, chicken, puffer fish, worm, fly and yeast. Pathways are represented by simple diagrams following an SBGN-like format.

Reactome's annotated data describe reactions possible if all annotated proteins and small molecules were present and active simultaneously in a cell. By overlaying an experimental dataset on these annotations, a user can perform a pathway over-representation analysis. By overlaying quantitative expression data or time series, a user can visualize the extent of change in affected pathways and its progression. A binomial test is used to calculate the probability shown for each result, and the p-values are corrected for the multiple testing (Benjamini–Hochberg procedure) that arises from evaluating the submitted list of identifiers against every pathway.

To learn more about our Pathway Analysis, please have a look at our relevant publications:

Fabregat A, Sidiropoulos K, Garapati P, Gillespie M, Hausmann K, Haw R, … D’Eustachio P (2016). The reactome pathway knowledgebase. Nucleic Acids Research, 44(D1), D481–D487. https://doi.org/10.1093/nar/gkv1351.

Fabregat A, Sidiropoulos K, Viteri G, Forner O, Marin-Garcia P, Arnau V, … Hermjakob H (2017). Reactome pathway analysis: a high-performance in-memory approach. BMC Bioinformatics, 18.

2. Properties

- This is an **overrepresentation** analysis: A statistical (hypergeometric distribution) test that determines whether certain Reactome pathways are over-represented (enriched) in the submitted data. It answers the question ‘Does my list contain more proteins for pathway X than would be expected by chance?’ This test produces a probability score, which is corrected for false discovery rate using the Benjamani-Hochberg method.
- 5 out of 13 identifiers in the sample were found in Reactome, where 26 pathways were hit by at least one of them.
- All non-human identifiers have been converted to their human equivalent.
- This report is filtered to show only results for species 'Homo sapiens' and resource 'UniProt'.
- The unique ID for this analysis (token) is MjAyMzA4MTcwNjQzNThfMjExOTg%3D. This ID is valid for at least 7 days in Reactome's server. Use it to access Reactome services with your data.

3. Genome-wide overview

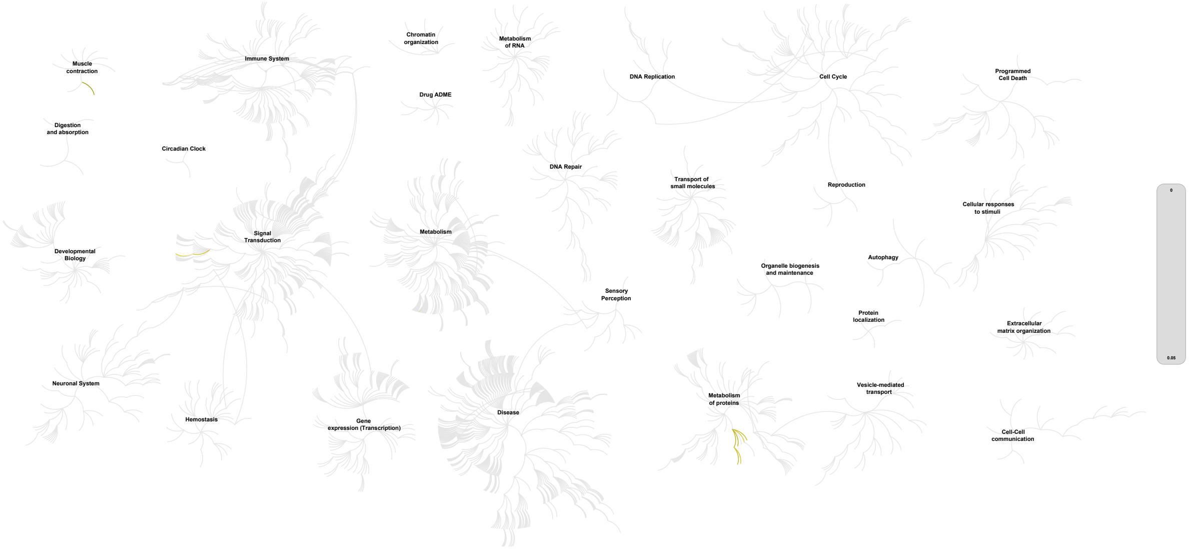

This figure shows a genome-wide overview of the results of your pathway analysis. Reactome pathways are arranged in a hierarchy. The center of each of the circular "bursts" is the root of one top-level pathway, for example "DNA Repair". Each step away from the center represents the next level lower in the pathway hierarchy. The color code denotes over-representation of that pathway in your input dataset. Light grey signifies pathways which are not significantly over-represented.

4. Most significant pathways

The following table shows the 25 most relevant pathways sorted by p-value.

Pathway name	Entities				Reactions	
	found	ratio	p-value	FDR*	found	ratio
Prostanoid ligand receptors	1 / 9	7.74e-04	0.01	0.055	1 / 9	6.29e-04
Peptide hormone biosynthesis	1 / 12	0.001	0.013	0.055	1 / 5	3.49e-04
Synthesis, secretion, and inactivation of Glucose-dependent Insulinotropic Polypeptide (GIP)	1 / 13	0.001	0.014	0.055	1 / 7	4.89e-04
Synthesis of PE	1 / 13	0.001	0.014	0.055	1 / 9	6.29e-04
Eicosanoid ligand-binding receptors	1 / 15	0.001	0.017	0.055	1 / 15	0.001
Synthesis, secretion, and deacylation of Ghrelin	1 / 20	0.002	0.022	0.055	1 / 8	5.59e-04
Synthesis, secretion, and inactivation of Glucagon-like Peptide-1 (GLP-1)	1 / 21	0.002	0.023	0.055	1 / 8	5.59e-04
Incretin synthesis, secretion, and inactivation	1 / 24	0.002	0.027	0.055	2 / 15	0.001
Insulin processing	1 / 25	0.002	0.028	0.055	1 / 17	0.001
Striated Muscle Contraction	1 / 36	0.003	0.04	0.079	4 / 4	2.80e-04
Peptide hormone metabolism	1 / 88	0.008	0.094	0.187	5 / 65	0.005
Stimuli-sensing channels	1 / 107	0.009	0.113	0.187	1 / 33	0.002
Glycerophospholipid biosynthesis	1 / 128	0.011	0.134	0.187	1 / 133	0.009
Ion channel transport	1 / 184	0.016	0.187	0.187	1 / 51	0.004
Muscle contraction	1 / 204	0.018	0.206	0.206	4 / 53	0.004
Phospholipid metabolism	1 / 211	0.018	0.212	0.212	1 / 218	0.015
G alpha (i) signalling events	1 / 317	0.027	0.302	0.302	3 / 74	0.005
Class A/1 (Rhodopsin-like receptors)	1 / 333	0.029	0.315	0.315	1 / 185	0.013
GPCR ligand binding	1 / 470	0.04	0.415	0.415	1 / 217	0.015
GPCR downstream signalling	1 / 638	0.055	0.52	0.52	3 / 175	0.012
Signaling by GPCR	1 / 713	0.061	0.561	0.561	4 / 392	0.027
Transport of small molecules	1 / 729	0.063	0.569	0.569	1 / 454	0.032
Metabolism of lipids	1 / 752	0.065	0.581	0.581	1 / 965	0.067
Metabolism of proteins	1 / 1,949	0.168	0.908	0.908	5 / 812	0.057
Metabolism	1 / 2,145	0.185	0.93	0.93	1 / 2,268	0.159

* False Discovery Rate

5. Pathways details

For every pathway of the most significant pathways, we present its diagram, as well as a short summary, its bibliography and the list of inputs found in it.

1. Prostanoid ligand receptors (R-HSA-391908)

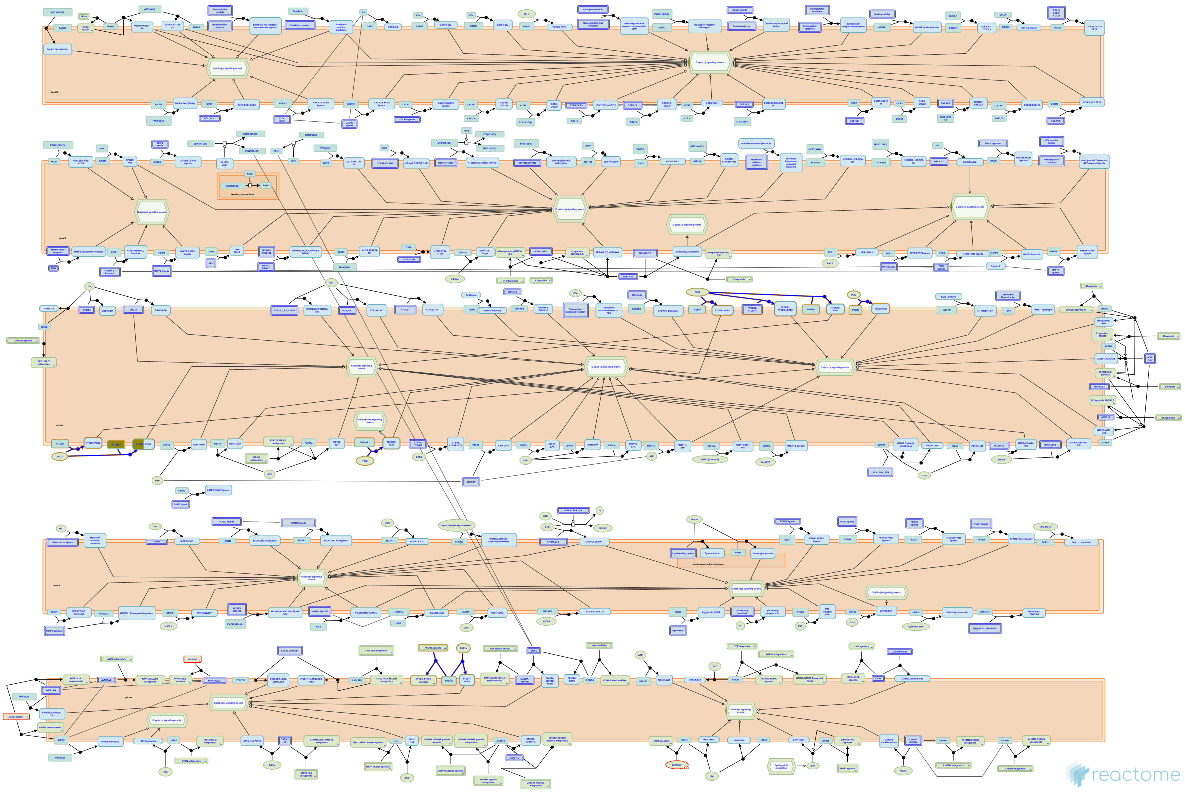

Fatty acid cyclo-oxygenase (COX) converts arachidonic acid to prostaglandin H2 (PGH2) from which the prostanoids PGD2, PGE2, PGF2alpha, PGI2 (prostacyclin) and thromboxane A2 (TXA2) are derived. Based on the agonist potencies, five prostanoid receptors are recognized and correspondingly named DP, EP, FP, IP and TP receptors (Coleman RA et al, 1994). Additionally, EP receptors contains four subtypes, termed EP1, EP2, EP3 and EP4; the DP receptor also has two subtypes, DP1 and DP2 (CRTH2).

References

Smith WL, Narumiya S & Coleman RA (1994). International Union of Pharmacology classification of prostanoid receptors: properties, distribution, and structure of the receptors and their subtypes. Pharmacol Rev, 46, 205-29.

Edit history

Date	Action	Author
2009-02-25	Created	Jassal B
2009-04-02	Edited	Jassal B
2009-04-02	Authored	Jassal B
2009-05-29	Reviewed	D'Eustachio P

Date	Action	Author
2023-05-21	Modified	Wright A

1 submitted entities found in this pathway, mapping to 1 Reactome entities

Input	UniProt Id
Gpr1	Q9Y5Y4

2. Peptide hormone biosynthesis (R-HSA-209952)

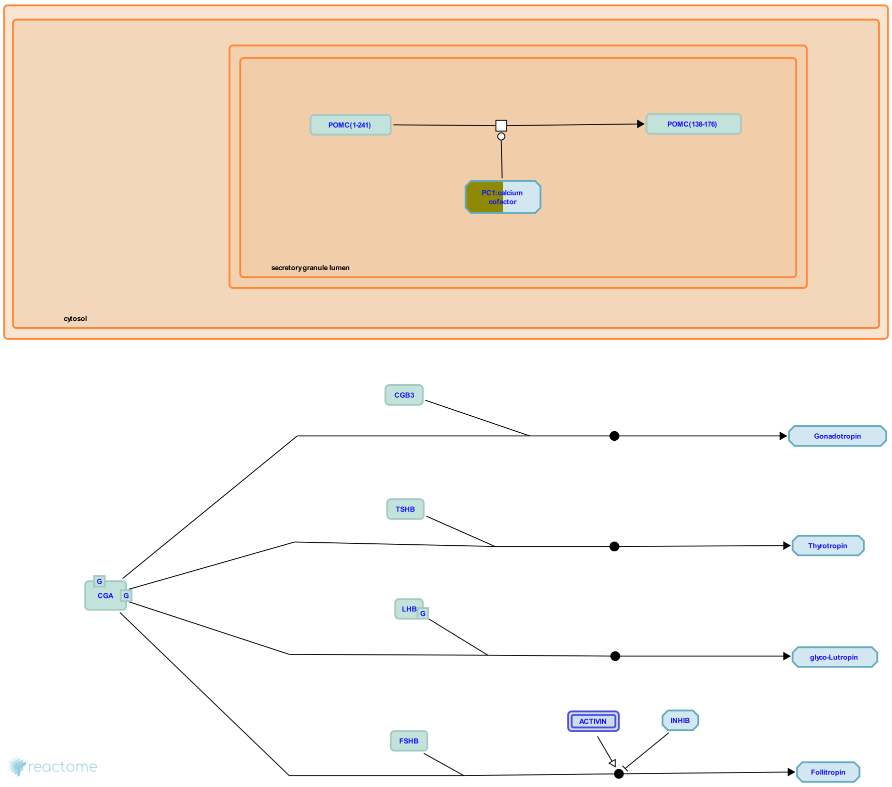

Peptide hormones are peptides that are secreted directly into the blood stream (endocrine hormones). They are synthesized as precursors that require proteolytic processing (not discussed here) to generate the biologically active peptides that mediate neurotransmission and hormonal action. Glycoprotein hormones (those which include carbohydrate side-chains) and the processing of corticotropin are annotated here.

References

Steiner DF & Tager HS (1974). Peptide hormones. Annu Rev Biochem, 43, 509-38.

Edit history

Date	Action	Author
2008-01-08	Created	Jassal B
2008-10-01	Authored	Jassal B
2008-11-17	Edited	Jassal B
2008-11-29	Reviewed	D'Eustachio P
2023-05-21	Modified	Wright A

1 submitted entities found in this pathway, mapping to 1 Reactome entities

Input	UniProt Id
Pcsk1	P29120

3. Synthesis, secretion, and inactivation of Glucose-dependent Insulinotropic Polypeptide (GIP) (R-HSA-400511)

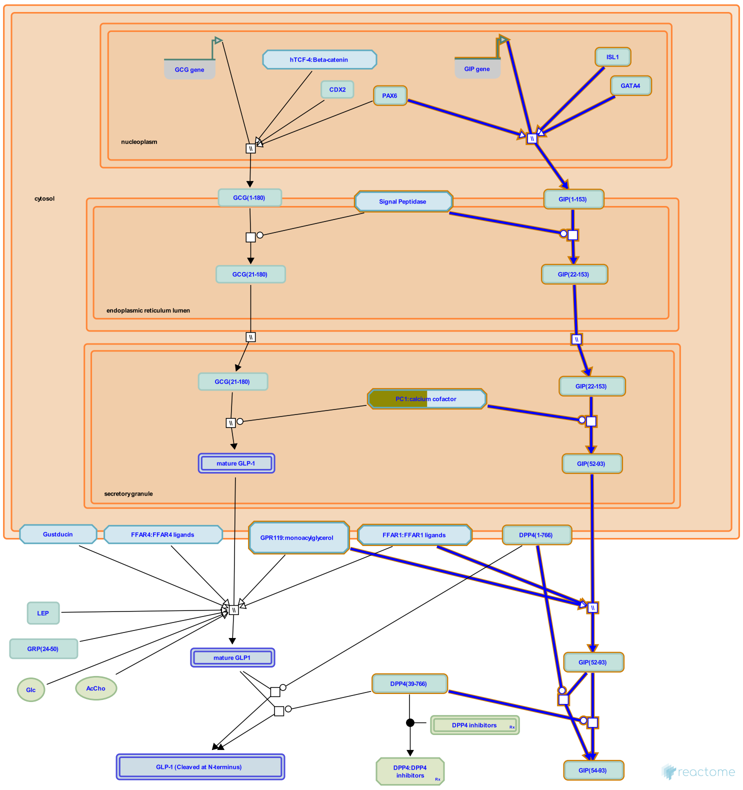

Cellular compartments: endoplasmic reticulum membrane, plasma membrane, nucleoplasm, extracellular region, endoplasmic reticulum lumen, cytosol, secretory granule lumen.

In K cells of the intestine the transcription factors PAX6 and PDX-1 activate transcription of the gene encoding Glucose-dependent Insulinotropic Polypeptide (GIP, first called Gastric Inhibitory Peptide). ProGIP is cleaved in secretory granules by Prohormone Convertase 1 (PC1) at 2 sites to yield mature GIP. In response to fat the GIP is secreted into the bloodstream. The half-life of GIP in the bloodstream is determined by Dipeptidyl Peptidase IV, which cleaves 2 amino acids at the amino terminus of GIP, rendering it biologically inactive.

References

Kim W & Egan JM (2008). The role of incretins in glucose homeostasis and diabetes treatment. Pharmacol Rev, 60, 470-512.

Todd JF & Bloom SR (2007). Incretins and other peptides in the treatment of diabetes. Diabet Med, 24, 223-32.

Drucker DJ & Baggio LL (2007). Biology of incretins: GLP-1 and GIP. Gastroenterology, 132, 2131-57.

Edit history

Date	Action	Author
2009-03-24	Created	May B
2009-05-19	Authored	May B
2009-09-09	Edited	May B

Date	Action	Author
2010-06-25	Reviewed	Bloom SR
2023-05-21	Modified	Wright A

1 submitted entities found in this pathway, mapping to 1 Reactome entities

Input	UniProt Id
Pcsk1	P29120

4. Synthesis of PE (R-HSA-1483213)

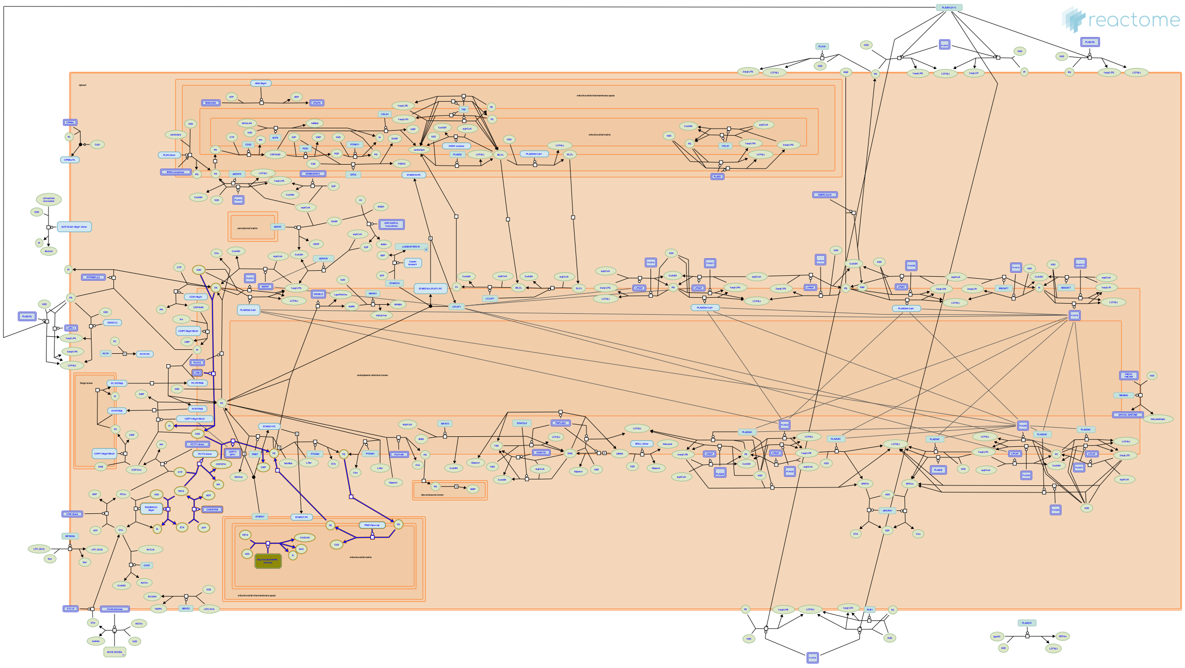

De novo (Kennedy pathway) synthesis of phosphatidylethanolamine (PE) involves phosphorylation of ethanolamine (ETA) to phosphoethanolamine (PETA) followed by condensing with cytidine triphosphate (CTP) to form CDP-ethanolamine (CDP-ETA). Diacylglycerol (DAG) and CDP-ETA together then form PE. Alternatively, PE is formed when phosphatidylserine (PS) is decarboxylated by phosphatidylserine decarboxylase proenzyme (PISD) (Henneberry et al. 2002, Vance 1991, Vance 1990).

References

Henneberry AL, Wright MM & McMaster CR (2002). The major sites of cellular phospholipid synthesis and molecular determinants of Fatty Acid and lipid head group specificity. Mol Biol Cell, 13, 3148-61.

Vance JE (1990). Phospholipid synthesis in a membrane fraction associated with mitochondria. J Biol Chem, 265, 7248-56.

Vance JE (1991). Newly made phosphatidylserine and phosphatidylethanolamine are preferentially translocated between rat liver mitochondria and endoplasmic reticulum. J Biol Chem, 266, 89-97.

Edit history

Date	Action	Author
2011-08-12	Edited	Williams MG
2011-08-12	Created	Williams MG
2011-09-14	Authored	Williams MG
2012-05-14	Reviewed	Wakelam M
2023-05-21	Modified	Wright A

1 submitted entities found in this pathway, mapping to 1 Reactome entities

Input	UniProt Id
Etnppl	Q8TBG4

5. Eicosanoid ligand-binding receptors (R-HSA-391903)

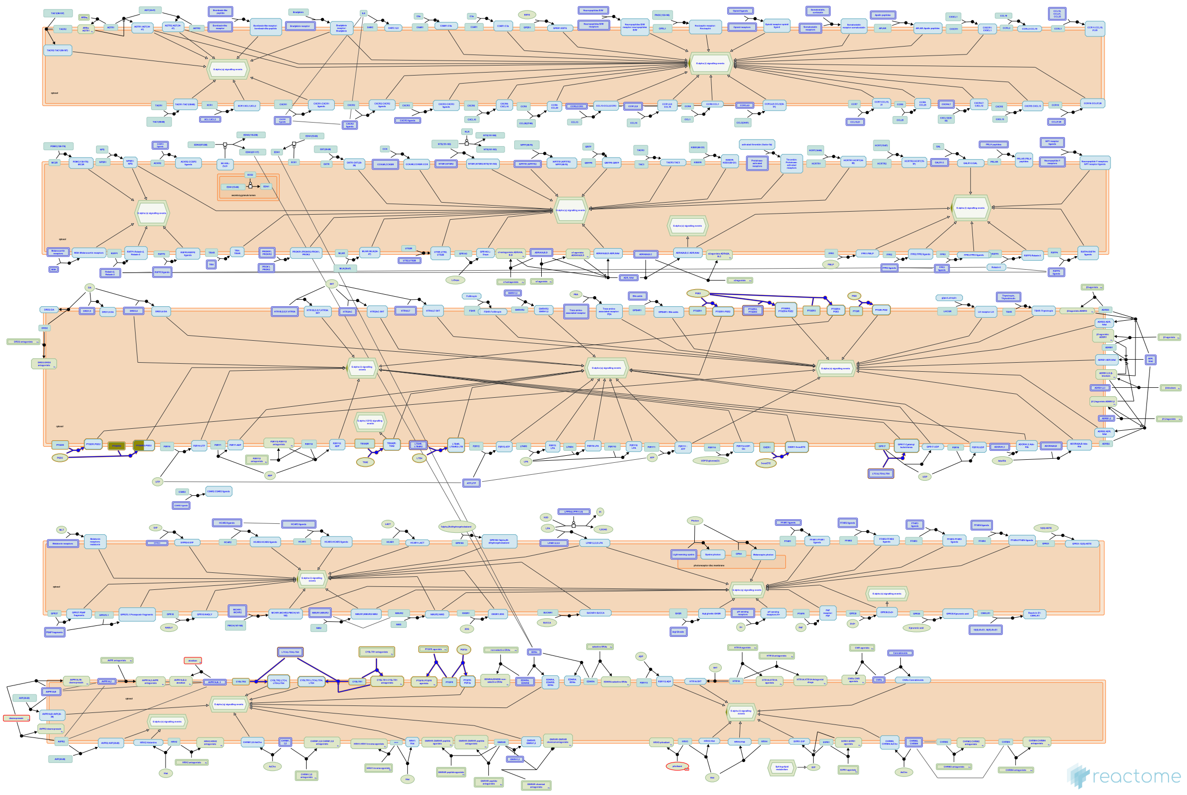

Eicosanoids, derived from polyunsaturated 20-carbon fatty acids, are paracrine and autocrine regulators of inflammation, smooth muscle contraction, and blood coagulation. The actions of eicosanoids are mediated by eicosanoid receptors, most of which are GPCRs. There are four types of eicosanoid GPCRs in humans; leukotriene, lipoxin (Brink C et al, 2003), prostanoid (Coleman RA et al, 1994) and oxoeicosanoid (Brink C et al, 2004) receptors.

References

Evans JF, Dahlén SE, Serhan CN, Yokomizo T, Drazen J, Rovati GE, ... Brink C (2004). International Union of Pharmacology XLIV. Nomenclature for the oxoeicosanoid receptor. Pharmacol Rev, 56 , 149-57.

Evans JF, Dahlén SE, Serhan CN, Yokomizo T, Nicosia S, Drazen J, ... Brink C (2003). International Union of Pharmacology XXXVII. Nomenclature for leukotriene and lipoxin receptors. Pharmacol Rev, 55, 195-227.

Smith WL, Narumiya S & Coleman RA (1994). International Union of Pharmacology classification of prostanoid receptors: properties, distribution, and structure of the receptors and their subtypes. Pharmacol Rev, 46, 205-29.

Edit history

Date	Action	Author
2009-02-25	Edited	Jassal B
2009-02-25	Authored	Jassal B
2009-02-25	Created	Jassal B
2009-05-29	Reviewed	D'Eustachio P

Date	Action	Author
2023-05-21	Modified	Wright A

1 submitted entities found in this pathway, mapping to 1 Reactome entities

Input	UniProt Id
Gpr1	Q9Y5Y4

6. Synthesis, secretion, and deacylation of Ghrelin (R-HSA-422085)

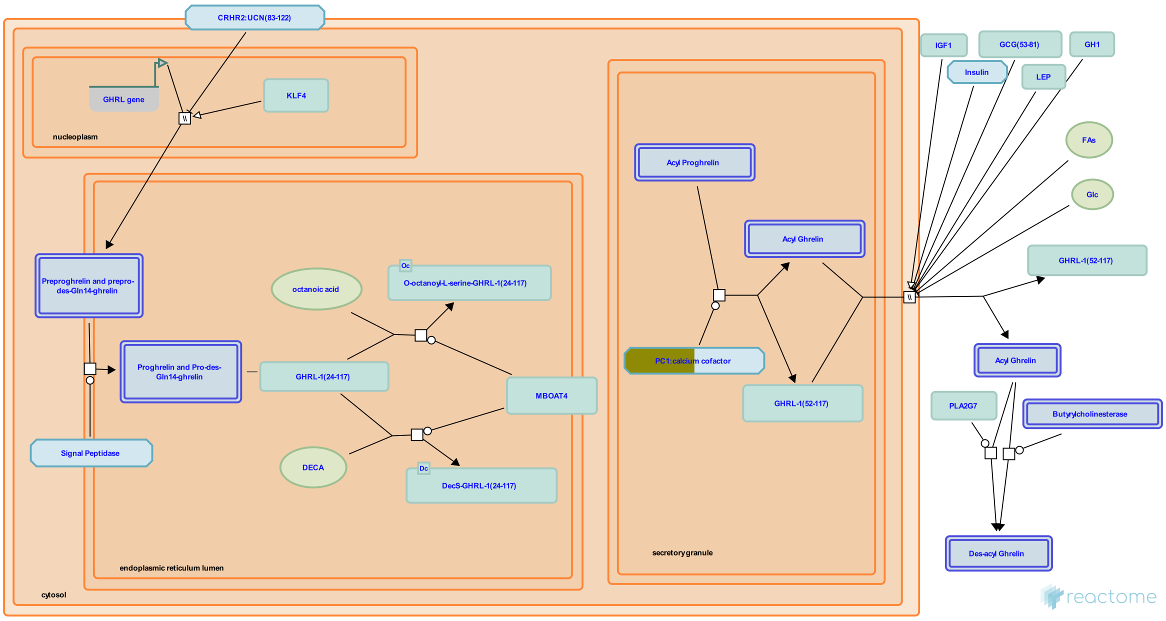

Cellular compartments: endoplasmic reticulum membrane, nucleoplasm, extracellular region, endoplasmic reticulum lumen, cytosol, secretory granule lumen.

Ghrelin is a peptide hormone of 28 amino acid residues which is acylated at the serine-3 of the mature peptide. Ghrelin is synthesized in several tissues: X/A-like cells of the gastric mucosa (the major source of ghrelin), hypothalamus, pituitary, adrenal gland, thyroid, breast, ovary, placenta, fallopian tube, testis, prostate, liver, gall bladder, pancreas, fat tissue, human lymphocytes, spleen, kidney, lung, skeletal muscle, myocardium, vein and skin. Ghrelin binds the GHS-R1a receptor present in hypothalamus pituitary, and other tissues. Binding causes appetite stimulation and release of growth hormone. Levels of circulating ghrelin rise during fasting, peak before a meal, and fall according to the calories ingested.

Preproghrelin is cleaved to yield proghrelin which is then acylated by ghrelin O-acyltransferase to yield octanoyl ghrelin and decanoyl ghrelin. Only octanoyl ghrelin is able to bind and activate the GHS-R1a receptor. Unacylated ghrelin (des-acyl ghrelin) is also present in plasma but its function is controversial.

Acyl proghrelin is cleaved by prohormone convertase 1/3 to yield the mature acyl ghrelin and C-ghrelin. Secretion of ghrelin is inhibited by insulin, growth hormone (somatotropin), leptin, glucose, glucagon, and fatty acids. Secretion is stimulated by insulin-like growth factor-1 and muscarinic agonists.

In the bloodstream acyl ghrelin is deacylated by butyrylcholinesterase and platelet-activating factor acetylhydrolase. Other enzymes may also deacylate acyl ghrelin.

References

Yin X, Li Y, Zhang W, An W & Xu G (2009). Ghrelin fluctuation, what determines its production?. Acta Biochim Biophys Sin (Shanghai), 41, 188-97.

Soares JB & Leite-Moreira AF (2008). Ghrelin, des-acyl ghrelin and obestatin: three pieces of the same puzzle. Peptides, 29, 1255-70.

Edit history

Date	Action	Author
2009-05-26	Created	May B
2009-06-11	Edited	May B
2009-06-11	Authored	May B
2009-08-30	Reviewed	Zhang Weizhen
2023-05-21	Modified	Wright A

1 submitted entities found in this pathway, mapping to 1 Reactome entities

Input	UniProt Id
Pcsk1	P29120

7. Synthesis, secretion, and inactivation of Glucagon-like Peptide-1 (GLP-1) (R-HSA-381771)

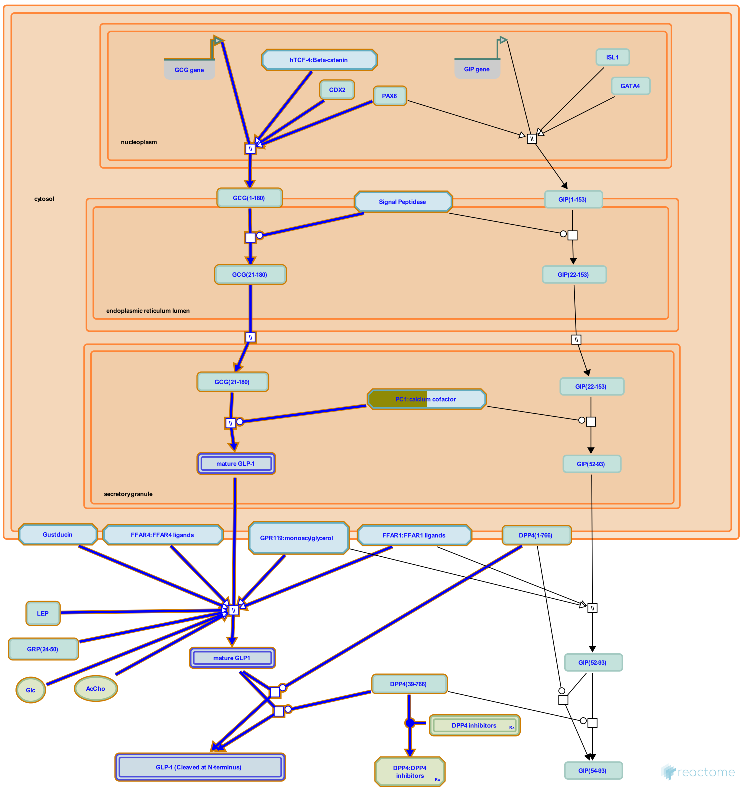

Cellular compartments: endoplasmic reticulum membrane, plasma membrane, nucleoplasm, extracellular region, endoplasmic reticulum lumen, cytosol, secretory granule lumen.

In L cells of the intestine the transcription factors TCF-4 (TCF7L2) and Beta-catenin form a heterodimer and bind the G2 enhancer of the Proglucagon gene GCG,activating its transcription to yield Proglucagon mRNA and, following translation, Proglucagon protein. The prohormone convertase PC1 present in the secretory granules of L cells cleaves Proglucagon at two sites to yield mostly Glucagon-like Peptide-1 (7-36) with a small amount of Glucagon-like Peptide-1 (7-37). Glucagon-like Peptide-1 (7-36 and 7-37) (GLP-1) is secreted into the bloodstream in response to glucose, fatty acids, insulin, leptin, gastrin-releasing peptide, cholinergic transmitters, beta-adrenergic transmitters, and peptidergic transmitters. The half-life of GLP-1 in the bloodstream is determined by Dipeptidyl Peptidase IV, which cleaves 2 amino acids at the amino terminus of GLP-1, rendering it biologically inactive.

References

Holst JJ (2007). The physiology of glucagon-like peptide 1. Physiol Rev, 87, 1409-39.

Kim W & Egan JM (2008). The role of incretins in glucose homeostasis and diabetes treatment. Pharmacol Rev, 60, 470-512.

Todd JF & Bloom SR (2007). Incretins and other peptides in the treatment of diabetes. Diabet Med, 24, 223-32.

Gremlich S, Macé K, Darimont C, Nicolas-Métral V, Rüegg UT & Reimer RA (2001). A human cellular model for studying the regulation of glucagon-like peptide-1 secretion. Endocrinology, 142, 4522-8.

Drucker DJ & Baggio LL (2007). Biology of incretins: GLP-1 and GIP. Gastroenterology, 132, 2131-57.

Edit history

Date	Action	Author
2008-11-20	Created	May B
2009-05-19	Authored	May B
2009-09-09	Edited	May B
2010-06-25	Reviewed	Bloom SR
2023-05-21	Modified	Wright A

1 submitted entities found in this pathway, mapping to 1 Reactome entities

Input	UniProt Id
Pcsk1	P29120

8. Incretin synthesis, secretion, and inactivation (R-HSA-400508)

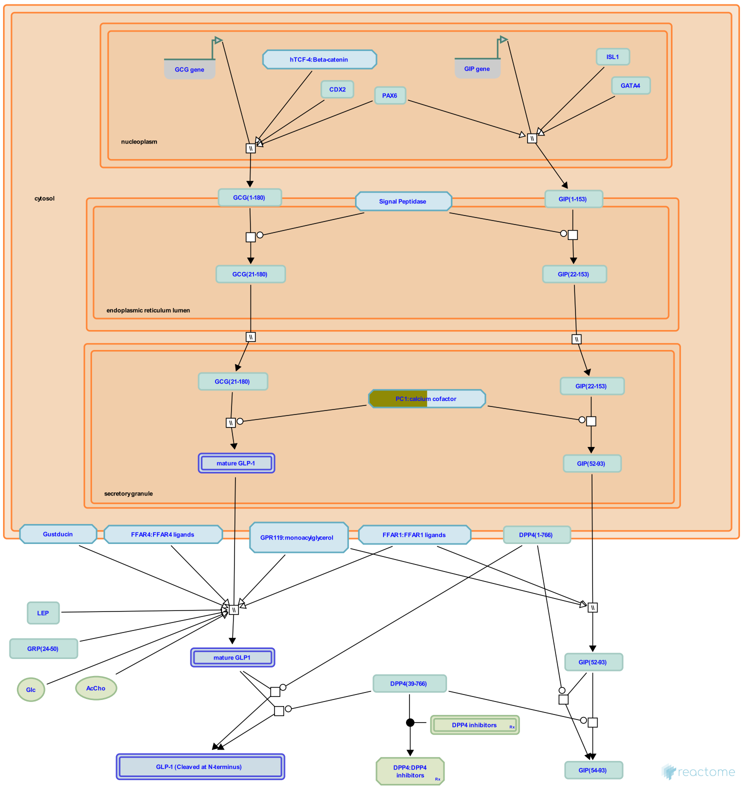

Cellular compartments: endoplasmic reticulum membrane, nucleoplasm, extracellular region, endoplasmic reticulum lumen, cytosol, secretory granule lumen.

Incretins are peptide hormones produced by the gut that enhance the ability of glucose to stimulate insulin secretion from beta cells in the pancreas. Two incretins have been identified: Glucagon-like Peptide-1 (GLP-1) and Glucose-dependent Insulinotropic Polypeptide (GIP, initially named Gastric Inhibitory Peptide). Both are released by cells of the small intestine, GLP-1 from L cells and GIP from K cells.

The control of incretin secretion is complex. Fatty acids, phospholipids, glucose, acetylcholine, leptin, and Gastrin-releasing Peptide all stimulate secretion of GLP-1. Fatty acids and phospholipids are the primary stimulants of secretion of GIP in humans (carbohydrates have more effect in rodents).

Incretins secreted into the bloodstream are subject to rapid inactivation by Dipeptidyl Peptidase IV (DPP IV), which confers half-lives of only a few minutes onto GLP-1 and GIP. Inhibitors of DPP IV, for example sitagliptin, are now being used in the treatment of Type 2 diabetes.

References

Kim W & Egan JM (2008). The role of incretins in glucose homeostasis and diabetes treatment. Pharmacol Rev, 60, 470-512.

Todd JF & Bloom SR (2007). Incretins and other peptides in the treatment of diabetes. Diabet Med, 24, 223-32.

Drucker DJ & Baggio LL (2007). Biology of incretins: GLP-1 and GIP. Gastroenterology, 132, 2131-57.

Edit history

Date	Action	Author
2009-03-24	Created	May B
2009-05-19	Edited	May B
2009-05-19	Authored	May B
2010-06-25	Reviewed	Bloom SR
2023-05-21	Modified	Wright A

1 submitted entities found in this pathway, mapping to 1 Reactome entities

Input	UniProt Id
Pcsk1	P29120

9. Insulin processing (R-HSA-264876)

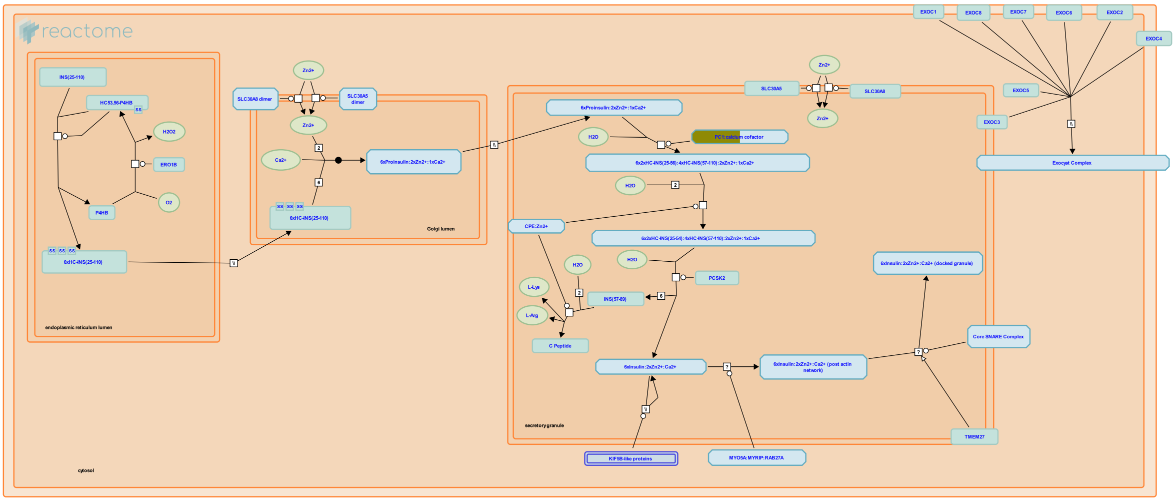

Cellular compartments: secretory granule lumen, cytosol, endoplasmic reticulum lumen, COPII-coated ER to Golgi transport vesicle, extracellular region, Golgi lumen, nucleoplasm, plasma membrane, endoplasmic reticulum membrane, secretory granule membrane.

Generation of insulin containing secretory granules from newly synthesized proinsulin in the lumen of the endoplasmic reticulum (ER) involves formation of proinsulin intramolecular disulfide bonds, formation of proinsulin zinc calcium complexes, proteolytic cleavage of proinsulin to yield insulin and C peptide, and translocation of the granules across the cytosol to the plasma membrane (Dodson & Steiner 1998).

Transcription of the human insulin gene INS is annotated as part of the pathway “Regulation of gene expression in beta cells” (see reaction R-HSA-211289). The preproinsulin mRNA is translated by ribosomes at the rough endoplasmic reticulum (ER) and the preproinsulin enters the secretion pathway by virtue of its signal peptide, which is co-translationally cleaved to yield proinsulin.

In the process annotated here, within the ER, three intramolecular disulfide bonds form in proinsulin, mediated by P4HD (PDI1A) and ERO1B proteins. Correctly folded, disulfide-bonded proinsulin then moves via vesicles from the ER to the Golgi Complex where it forms complexes with zinc and calcium.

Proinsulin zinc calcium complexes bud in vesicles from the trans Golgi to form immature secretory vesicles (secretory granules) in the cytosol. Within the immature granules, endoproteases PCKS1 and PCKS2 (Prohormone Convertases 1 and 2) cleave proinsulin at two sites and CPE (Carboxypeptidase E) removes additional amino acid residues to yield the cystine bonded A and B chains of mature insulin and the C peptide, which will be secreted with the insulin. The insulin zinc calcium complexes form insoluble crystals within the granule.

The insulin containing secretory granules are then translocated across the cytosol to the inner surface of the plasma membrane. Translocation occurs initially by attachment of the granules to Kinesin 1, which motors along microtubules, and then by attachment to Myosin Va, which motors along the microfilaments of the cortical actin network.

A pancreatic beta cell contains about 10,000 insulin granules of which about 1,000 are docked at the plasma membrane and 50 are readily releasable in immediate response to stimulation by glucose or other secretogogues. Docking is due to interaction between the Exocyst proteins EXOC3 on the granule membrane and EXOC4 on the plasma membrane. Exocytosis is accomplished by interaction between SNARE type proteins Syntaxin 1A and Syntaxin 4 on the plasma membrane and Synaptobrevin 2/VAMP2 on the granule membrane. Exocytosis is a calcium dependent process due to interaction of the calcium binding membrane protein Synaptotagmin V/IX with the SNARE type proteins.

References

Rutter GA & Hill EV (2006). Insulin vesicle release: walk, kiss, pause ... then run. Physiology (Bethesda), 21, 189-96.

Straub SG, Yajima H, Gunawardana S, Sharp GW, Daniel S, Bratanova-Tochkova TK, ... Liu YJ (2002). Triggering and augmentation mechanisms, granule pools, and biphasic insulin secretion . Diabetes, 51, S83-90.

Gerber SH & Südhof TC (2002). Molecular determinants of regulated exocytosis. Diabetes, 51, S3-11.

Poitout V, Stein R, Robertson RP, Harmon JS, Artner I & Hagman D (2006). Regulation of the insulin gene by glucose and fatty acids. J Nutr, 136, 873-6.

Steiner D & Dodson G (1998). The role of assembly in insulin's biosynthesis. Curr Opin Struct Biol, 8 , 189-94.

Edit history

Date	Action	Author
2008-04-15	Created	May B
2008-11-20	Edited	May B, Gopinathrao G
2008-11-20	Authored	May B, Gopinathrao G
2008-12-02	Reviewed	Matthews L, Gillespie ME, D'Eustachio P
2023-05-30	Modified	Wright A

1 submitted entities found in this pathway, mapping to 1 Reactome entities

Input	UniProt Id
Pcsk1	P29120

10. Striated Muscle Contraction (R-HSA-390522)

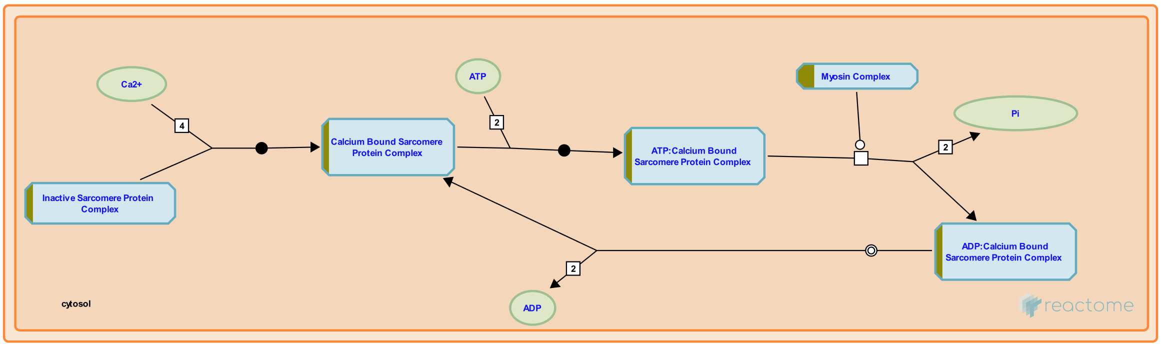

Cellular compartments: cytosol.

Striated muscle contraction is a process whereby force is generated within striated muscle tissue, resulting in a change in muscle geometry, or in short, increased force being exerted on the tendons. Force generation involves a chemo-mechanical energy conversion step that is carried out by the actin/myosin complex activity, which generates force through ATP hydrolysis. Striated muscle is a type of muscle composed of myofibrils, containing repeating units called sarcomeres, in which the contractile myofibrils are arranged in parallel to the axis of the cell, resulting in transverse or oblique striations observable at the level of the light microscope.

Here striated muscle contraction is represented on the basis of calcium binding to the troponin complex, which exposes the active sites of actin. Once the active sites of actin are exposed, the myosin complex bound to ADP can bind actin and the myosin head can pivot, pulling the thin actin and thick myosin filaments past one another. Once the myosin head pivots, ADP is ejected, a fresh ATP can be bound and the energy from the hydrolysis of ATP to ADP is channeled into kinetic energy by resetting the myosin head. With repeated rounds of this cycle the sarcomere containing the thin and thick filaments effectively shortens, forming the basis of muscle contraction.

References

NIEDERGERKE R & HUXLEY AF (1954). Measurement of muscle striations in stretch and contraction. J Physiol, 124, 46-7P.

Cooke R (2004). The sliding filament model: 1972-2004. J Gen Physiol, 123, 643-56.

NIEDERGERKE R & HUXLEY AF (1954). Structural changes in muscle during contraction; interference microscopy of living muscle fibres. Nature, 173, 971-3.

Ohtsuki I & Ebashi S (2007). *Regulatory Mechanisms of Striated Muscle Contraction* .

Szent-Györgyi AG (2004). The early history of the biochemistry of muscle contraction. J Gen Physiol, 123, 631-41.

Edit history

Date	Action	Author
2008-01-11	Reviewed	Rush MG
2009-02-10	Authored	Gillespie ME
2009-02-10	Created	Gillespie ME
2009-03-11	Edited	Gillespie ME

Date	Action	Author
2023-05-21	Modified	Wright A

1 submitted entities found in this pathway, mapping to 1 Reactome entities

Input	UniProt Id
Myh8	P13535

11. Peptide hormone metabolism (R-HSA-2980736)

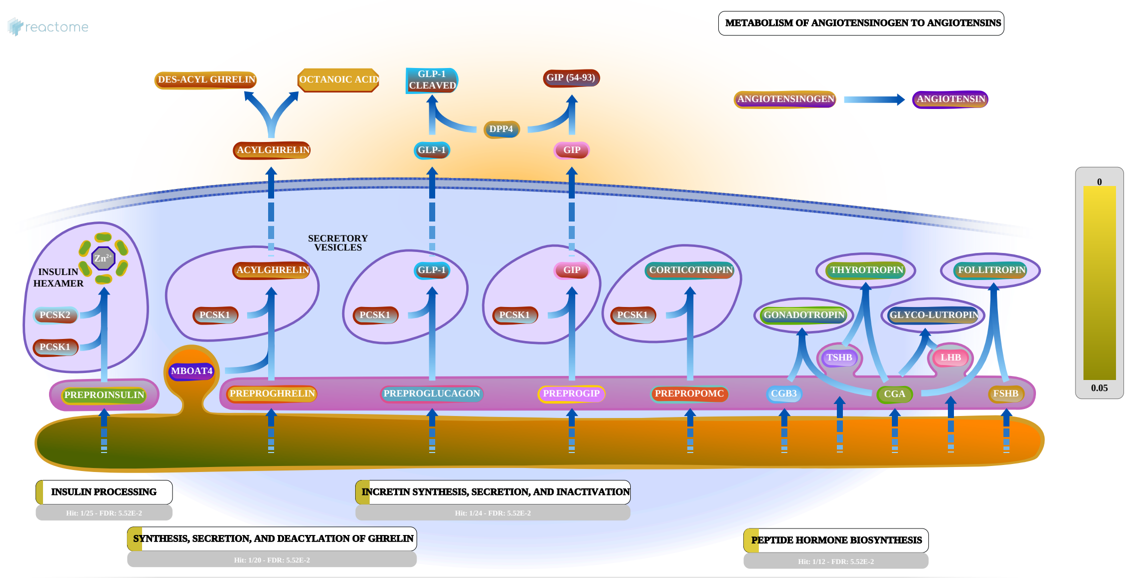

Cellular compartments: secretory granule membrane, endoplasmic reticulum membrane, plasma membrane, nucleoplasm, Golgi lumen, extracellular region, COPII-coated ER to Golgi transport vesicle, endoplasmic reticulum lumen, cytosol, secretory granule lumen.

Peptide hormones are cleaved from larger precursors in the secretory system (endoplasmic reticulum, Golgi apparatus, secretory granules) of the cell. After secretion peptide hormones are modified and degraded by extracellular proteases.

Insulin processing occurs in 4 steps: formation of intramolecular disulfide bonds, formation of proinsulin-zinc-calcium complexes, proteolytic cleavage of proinsulin by PCSK1 (PC1/3) and PCSK2 to yield insulin, translocation of the granules across the cytosol to the plasma membrane.

During Synthesis, secretion, and deacetylation of Ghrelin, proghrelin is acylated by ghrelin O-acyltransferase and cleaved by PCSK1 to yield the mature acyl ghrelin and C-ghrelin. In the bloodstream acyl ghrelin is deacylated by butyrylcholinesterase and platelet-activating factor acetylhydrolase.

During Metabolism of Angiotensinogen to Angiotensin, Renin cleaves angiotensinogen to yield a decapaptide, angiotensin I (angiotensin-1, angiotensin-(1-10)). Two C-terminal amino acid residues of angiotensin I are then removed by angiotensin-converting enzyme (ACE), located on the surface of endothelial cells, to yield angiotensin II (angiotensin-2, angiotensin-(1-8)), the active peptide that causes vasoconstriction, resorption of sodium and chloride, excretion of potassium, water retention, and aldosterone secretion. More recently other, more tissue-localized pathways leading to angiotensin II and alternative derivatives of angiotensinogen have been identified and described.

Incretin synthesis, secretion, and inactivation occurs through processing of incretin precursors (preproGLP-1 and preproGIP) by PCSK1. After secretion both incretins (GLP-1 and GIP) can be inactivated by cleavage by DPP4.

Peptide hormone biosynthesis describes processing of glycoprotein hormones (those which include carbohydrate side-chains) and corticotropin.

References

Mogensen N, Bardram L, Johnsen AH, Hilsted L, Odum L, Cerman J, ... Cantor P (1989). Peptide hormone expression and precursor processing. Acta Oncol, 28, 315-8.

Steiner DF (2011). On the discovery of precursor processing. Methods Mol. Biol., 768, 3-11.

Edit history

Date	Action	Author
2013-01-11	Edited	May B
2013-01-11	Authored	May B
2013-01-12	Reviewed	May B
2013-01-12	Created	May B
2023-05-21	Modified	Wright A

1 submitted entities found in this pathway, mapping to 1 Reactome entities

Input	UniProt Id
Pcsk1	P29120

12. Stimuli-sensing channels (R-HSA-2672351)

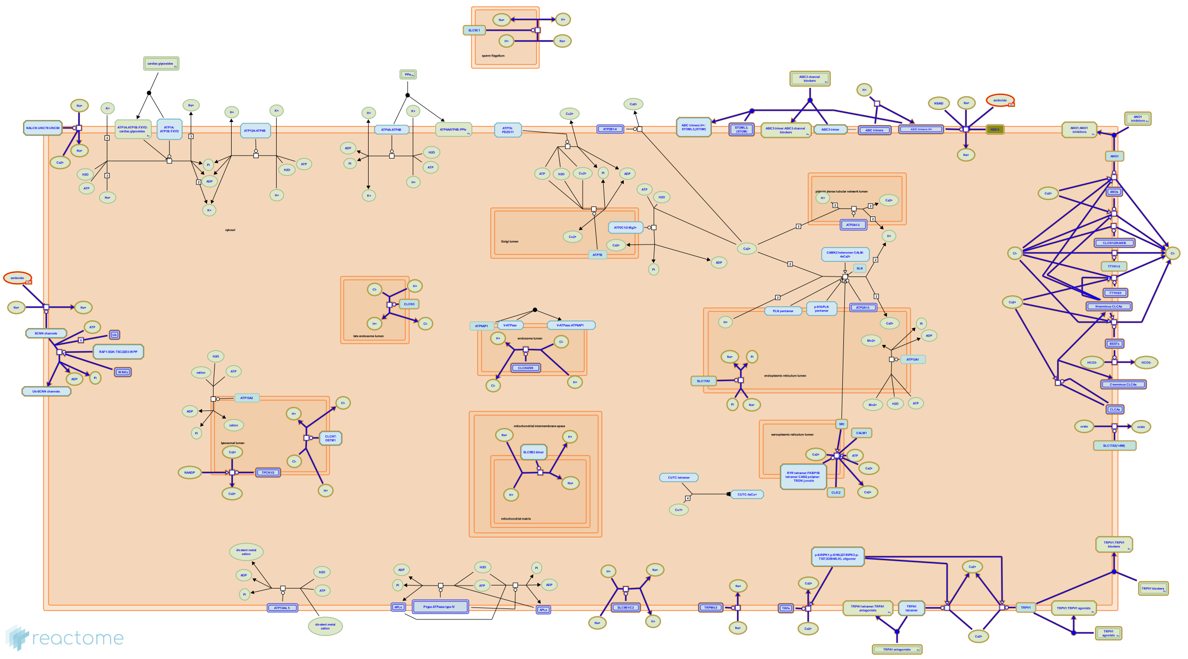

Ion channels that mediate sensations such as pain, warmth, cold, taste pressure and vision. Channels that mediate these sensations include acid-sensing ion channels (ASICs) (Wang & Xu 2011, Qadri et al. 2012, Deval et al. 2010) and the transient receptor potential channels (TRPCs) (Takahashi et al. 2012, Numata et al. 2011 in "TRP Channels" Zhu, MX editor, CRC Press, 2011, Ramsey et al. 2006, Montell 2005). Many channels are sensitive to changes in calcium (Ca2+) levels, both inside and outside the cell. Examples are protein tweety homologs 2 and 3 (TTYH2, 3) (Suzuki 2006), bestrophins 1-4 (BEST1-4) (Sun et al. 2002, Tsunenari et al. 2003, Kunzelmann et al. 2009, Hartzell et al. 2008) and ryanodine receptor tetramers (RYRs) (Beard et al. 2009).

References

Diochot S, Salinas M, Gasull X, Noël J, Deval E, Baron A & Lingueglia E (2010). Acid-sensing ion channels (ASICs): pharmacology and implication in pain. Pharmacol. Ther., 128, 549-58.

Suzuki M (2006). The Drosophila tweety family: molecular candidates for large-conductance Ca2+-activated Cl- channels. Exp. Physiol., 91, 141-7.

Xu TL & Wang YZ (2011). Acidosis, acid-sensing ion channels, and neuronal cell death. Mol. Neurobiol., 44, 350-8.

Yau KW, Williams J, Sun H, Smallwood P, Nathans J, Tsunenari T & Cahill H (2003). Structure-function analysis of the bestrophin family of anion channels. J. Biol. Chem., 278, 41114-25.

Xiao Q, Hartzell HC, Qu Z, Chien LT & Yu K (2008). Molecular physiology of bestrophins: multifunctional membrane proteins linked to best disease and other retinopathies. Physiol. Rev., 88, 639-72.

Edit history

Date	Action	Author
2012-11-27	Edited	Jassal B
2012-11-27	Authored	Jassal B

Date	Action	Author
2012-11-27	Created	Jassal B
2013-01-28	Reviewed	He L
2023-05-21	Modified	Wright A

1 submitted entities found in this pathway, mapping to 1 Reactome entities

Input	UniProt Id
Asic4	Q96FT7

13. Glycerophospholipid biosynthesis (R-HSA-1483206)

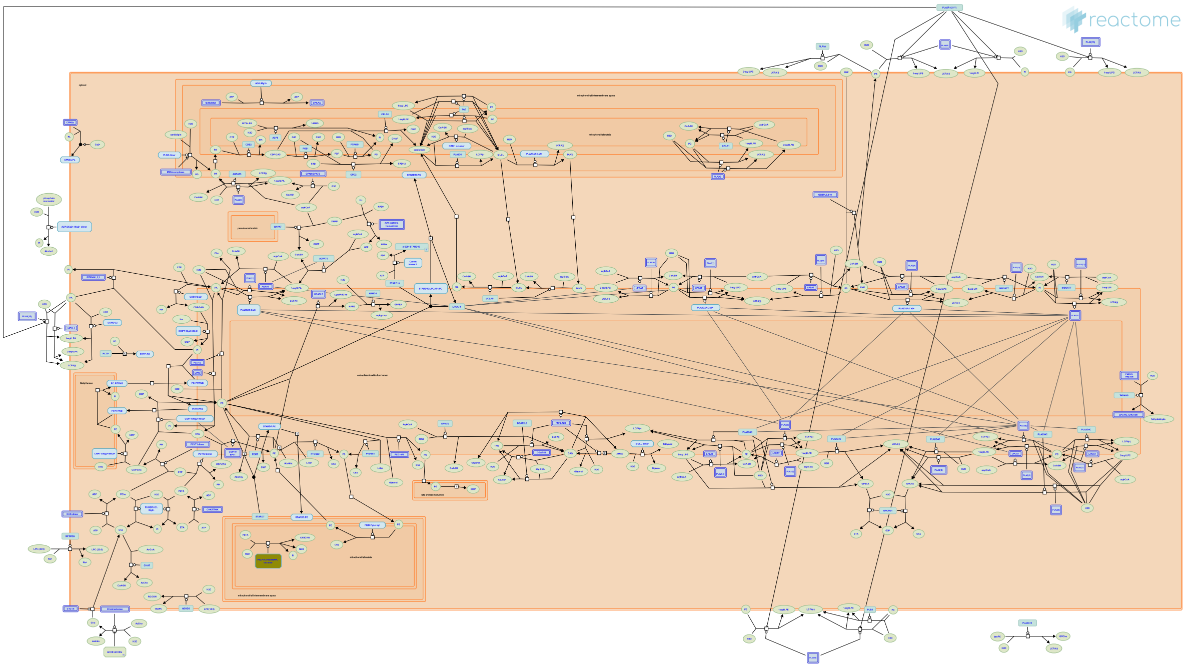

Glycerophospholipids are important structural and functional components of biological membranes and constituents of serum lipoproteins and the pulmonary surfactant. In addition, glycerophospholipids act as precursors of lipid mediators such as platelet-activating factor and eicosanoids. Cellular membranes contains a distinct composition of various glycerophospholipids such as phosphatidic acid (PA), phosphatidylcholine (PC), phosphatidylethanolamine (PE), phosphatidylserine (PS), phosphatidylglycerol (PG), phosphatidylinositol (PI), cardiolipin (CL), lysophosphatidic acid (LPA) and lysobisphosphatidic acid (also known as bis(monoacylglycerol) hydrogen phosphate - BMP).

Glycerophospholipids are first formed by the *de novo* (Kennedy) pathway using fatty acids activated as acyl-CoA donors. However, the acyl groups of glycerophospholipids are highly diverse and distributed in an asymmetric manner. Saturated and monounsaturated fatty acids are usually esterified at the *sn-1* position, whereas polyunsaturated acyl groups are esterified at the *sn-2* position. Subsequent acyl chain remodeling (Lands cycle) generates the diverse glycerophospholipid composition and asymmetry characteristic of cell membranes.

In the *de novo* pathway of glycerophospholipid biosynthesis, lysophosphatidic acid (LPA) is initially formed from glycerol 3-phosphate (G3P). Next, LPA is converted to PA by a LPA acyltransferase (AGPAT, also known as LPAAT), then PA is metabolized into two types of glycerol derivatives. The first is diacylglycerol (DAG) which is converted to triacylglycerol (TAG), PC, and PE. Subsequently, PS is synthesized from PC or PE. The second is cytidine diphosphate-diacylglycerol (CDP-DAG), which is processed into PI, PG, CL, and BMP. Each glycerophospholipid is involved in acyl chain remodeling via cleavage by phospholipases followed by reacylation by an acyltransferase.

Most of the glycerophospholipids are synthesized at the endoplasmic reticulum (ER), however, some, most notably cardiolipin, and BMP are synthesized in the mitochondrial and endosomal membranes respectively. Since the most of the glycerophospholipids are found in all membrane compartments, there must be extensive network of transport of glycerophospholipids from one membrane compartment to another via various mechanisms including diffusion through the cytosol, formation of transportation complexes, and diffusion via membrane contact sites (MCS) (Osman et al. 2011, Lebiedzinska et al. 2009, Lev 2010, Scherer & Schmitz 2011, Orso et al. 2011, Hermansson et al. 2011, Vance & Vance 2008).

References

Jones AW, Lebiedzinska M, Duszynski J, Szabadkai G & Wieckowski MR (2009). Interactions between the endoplasmic reticulum, mitochondria, plasma membrane and other subcellular organelles. Int J Biochem Cell Biol, 41, 1805-16.

Schmitz G, Grandl M & Orsó E (2011). Oxidized LDL-induced endolysosomal phospholipidosis and enzymatically modified LDL-induced foam cell formation determine specific lipid species modulation in human macrophages. Chem Phys Lipids.

Schmitz G & Scherer M (2011). Metabolism, function and mass spectrometric analysis of bis(monoacylglycero)phosphate and cardiolipin. Chem Phys Lipids.

Vance JE & Vance DE (2008). *Phospholipid biosynthesis in eukaryotes, Biochemistry of Lipids, Lipoproteins and Membranes, 5th Edition* , 213-244.

Lev S (2010). Non-vesicular lipid transport by lipid-transfer proteins and beyond. Nat Rev Mol Cell Biol, 11, 739-50.

Edit history

Date	Action	Author
2011-08-12	Edited	Williams MG
2011-08-12	Created	Williams MG
2011-09-14	Authored	Williams MG
2023-05-21	Modified	Wright A

1 submitted entities found in this pathway, mapping to 1 Reactome entities

Input	UniProt Id
Etnppl	Q8TBG4

14. Ion channel transport (R-HSA-983712)

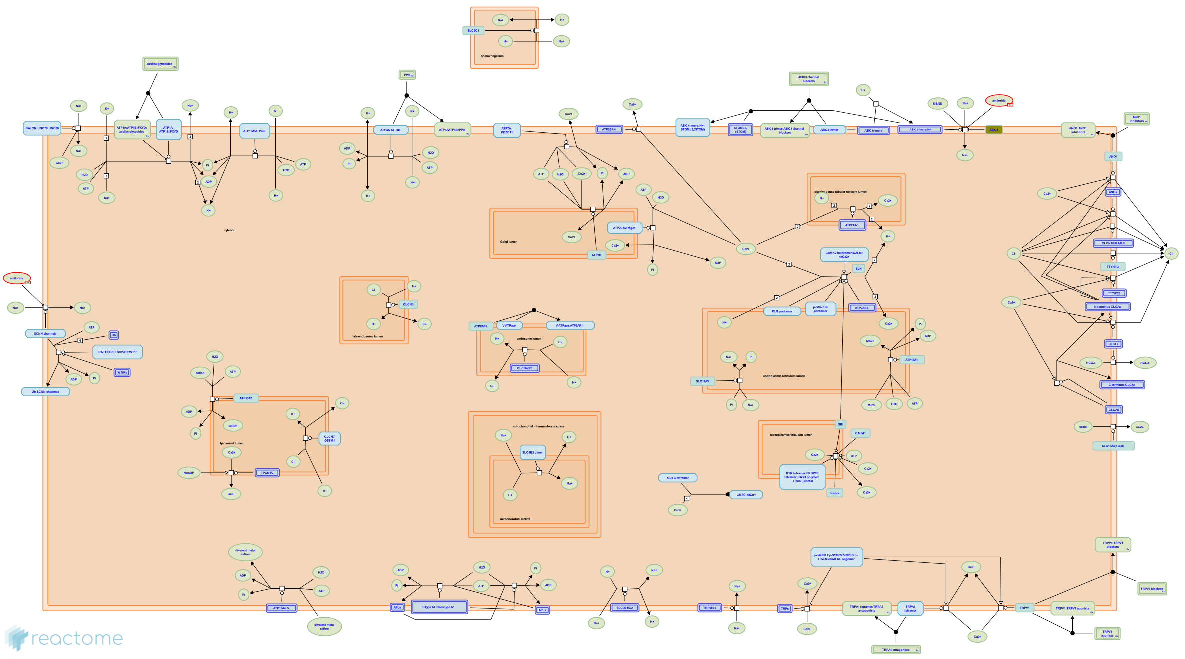

Ion channels mediate the flow of ions across the plasma membrane of cells. They are integral membrane proteins, typically a multimer of proteins, which, when arranged in the membrane, create a pore for the flow of ions. There are different types of ion channels. P-type ATPases undergo conformational changes to translocate ions. Ligand-gated ion channels operate like a gate, opened or closed by a chemical signal. Voltage-gated ion channels are activated by changes in electrical potential difference at the membrane (Purves, 2001; Kuhlbrandt, 2004).

References

Purves D (2001). *Chapter 4: Channels and Transporters, Neuroscience (2nd ed.)* .

Kühlbrandt W (2004). Biology, structure and mechanism of P-type ATPases. Nat Rev Mol Cell Biol, 5 , 282-95.

Edit history

Date	Action	Author
2010-11-02	Edited	Jassal B
2010-11-02	Authored	Jassal B
2010-11-02	Created	Jassal B
2010-11-15	Reviewed	He L
2023-05-21	Modified	Wright A

1 submitted entities found in this pathway, mapping to 1 Reactome entities

Input	UniProt Id
Asic4	Q96FT7

15. Muscle contraction (R-HSA-397014)

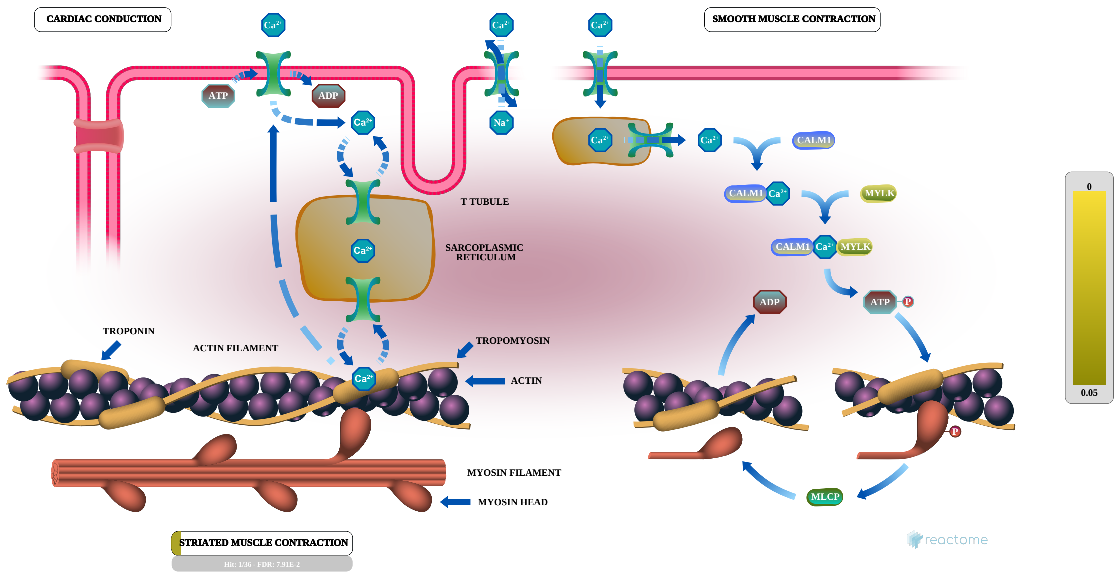

Cellular compartments: plasma membrane, cytosol.

In this module, the processes by which calcium binding triggers actin - myosin interactions and force generation in smooth and striated muscle tissues are annotated.

References

Edit history

Date	Action	Author
2009-02-10	Authored	Gillespie ME
2009-03-11	Edited	Gillespie ME
2009-03-11	Created	May B
2023-05-21	Modified	Wright A

1 submitted entities found in this pathway, mapping to 1 Reactome entities

Input	UniProt Id
Myh8	P13535

16. Phospholipid metabolism (R-HSA-1483257)

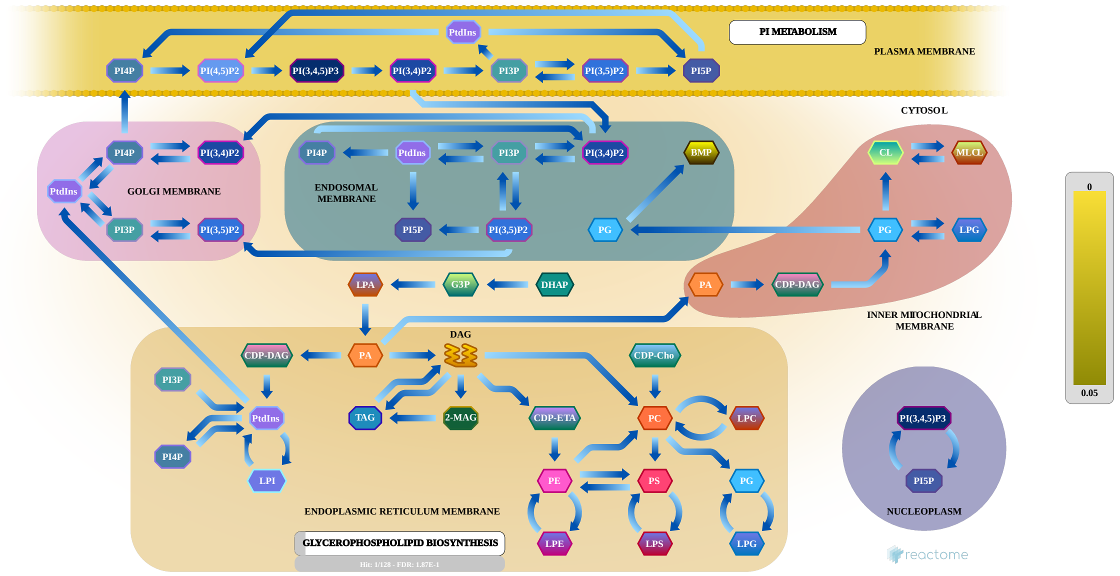

Phospholipids contain a polar head group and two long-chain fatty acyl moieties, one of which is generally unsaturated. The head group is a glycerol or serine phosphate attached to a polar group such as choline. These molecules are a major constituent of cellular membranes, where their diverse structures and asymmetric distributions play major roles in determining membrane properties (Dowhan 1997). The four major classes of phospholipids in human plasma membranes are phosphatidylethanolamine, phosphatidylserine, phosphatidylcholine, and sphingomyelin. The first three are derivatives of glycerol while sphingomyelin is a derivative of serine.

Here, pathways for the metabolism of glycerophospholipids, phosphphatidylinositol (PI), and sphingolipids are annotated.

References

Dowhan W (1997). Molecular basis for membrane phospholipid diversity: why are there so many lipids?. Annu. Rev. Biochem., 66, 199-232.

Edit history

Date	Action	Author
2011-08-12	Authored	Williams MG
2011-08-12	Created	Williams MG
2011-09-09	Edited	Williams MG
2012-05-20	Reviewed	D'Eustachio P
2023-05-21	Modified	Wright A

1 submitted entities found in this pathway, mapping to 1 Reactome entities

Input	UniProt Id
Etnppl	Q8TBG4

17. G alpha (i) signalling events (R-HSA-418594)

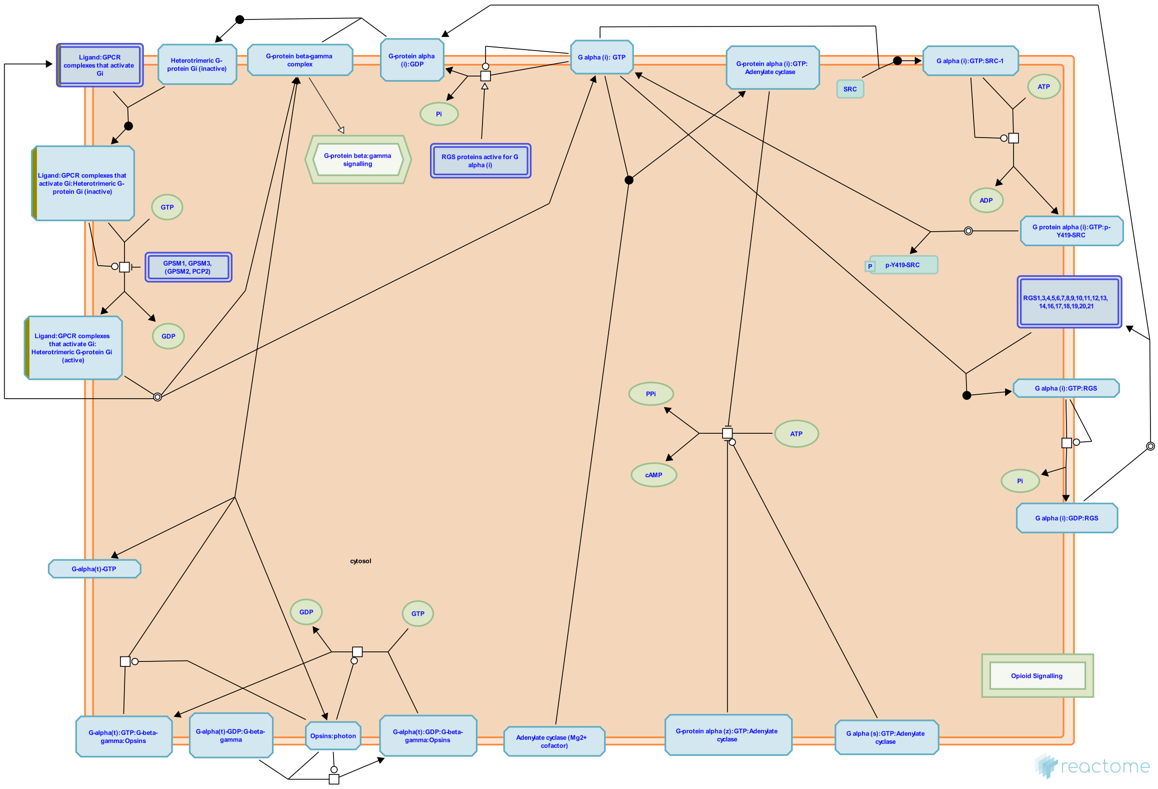

Cellular compartments: plasma membrane.

The classical signalling mechanism for G alpha (i) is inhibition of the cAMP dependent pathway through inhibition of adenylate cyclase (Dessauer C W et al. 2002). Decreased production of cAMP from ATP results in decreased activity of cAMP-dependent protein kinases. Other functions of G alpha (i) includes activation of the protein tyrosine kinase c-Src (Ma Y C et al. 2000). Regulator of G-protein Signalling (RGS) proteins can regulate the activity of G alpha (i) (Soundararajan et al. 2008).

References

Gilman AG (1987). G proteins: transducers of receptor-generated signals. Annu Rev Biochem, 56, 615-49.

Hildebrandt JD (1997). Role of subunit diversity in signaling by heterotrimeric G proteins. Biochem Pharmacol, 54, 325-39.

Edit history

Date	Action	Author
2009-04-24	Created	Jupe S
2009-05-01	Edited	Jupe S
2009-05-01	Authored	Jupe S
2009-06-03	Reviewed	Akkerman JW
2017-07-10	Revised	Varusai TM
2023-05-21	Modified	Wright A

1 submitted entities found in this pathway, mapping to 1 Reactome entities

Input	UniProt Id
Gpr1	Q9Y5Y4

18. Class A/1 (Rhodopsin-like receptors) (R-HSA-373076)

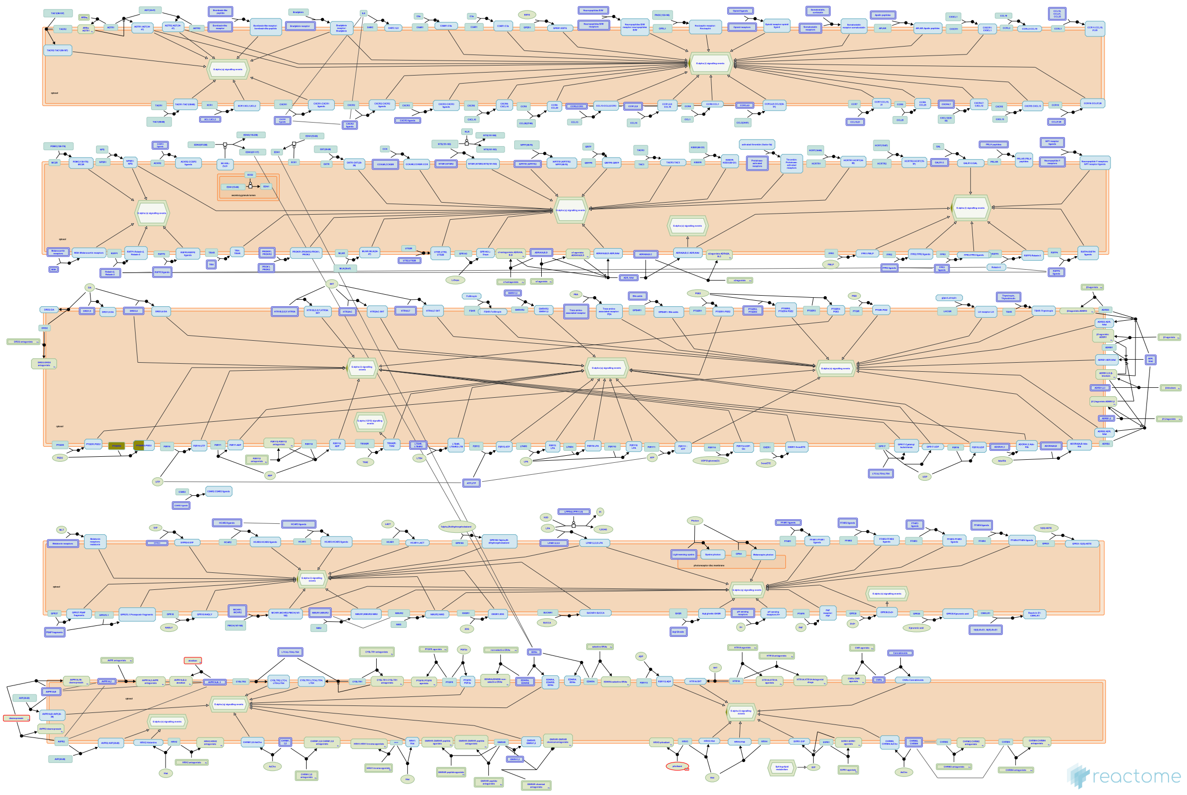

Rhodopsin-like receptors (class A/1) are the largest group of GPCRs and are the best studied group from a functional and structural point of view. They show great diversity at the sequence level and thus, can be subdivided into 19 subfamilies (Subfamily A1-19) based on a phylogenetic analysis (Joost P and Methner A, 2002). They represent members which include hormone, light and neurotransmitter receptors and encompass a wide range of functions including many autocrine, paracrine and endocrine processes.

References

Bouhelal R, Jacoby E, Gerspacher M & Seuwen K (2006). The 7 TM G-protein-coupled receptor target family. ChemMedChem, 1, 761-82.

Edit history

Date	Action	Author
2008-07-03	Authored	Jassal B
2008-07-14	Created	Jassal B
2008-09-01	Edited	D'Eustachio P
2008-09-01	Reviewed	Bockaert J
2016-11-18	Revised	D'Eustachio P
2023-05-21	Modified	Wright A

1 submitted entities found in this pathway, mapping to 1 Reactome entities

Input	UniProt Id
Gpr1	Q9Y5Y4

Input	UniProt Id

19. GPCR ligand binding (R-HSA-500792)

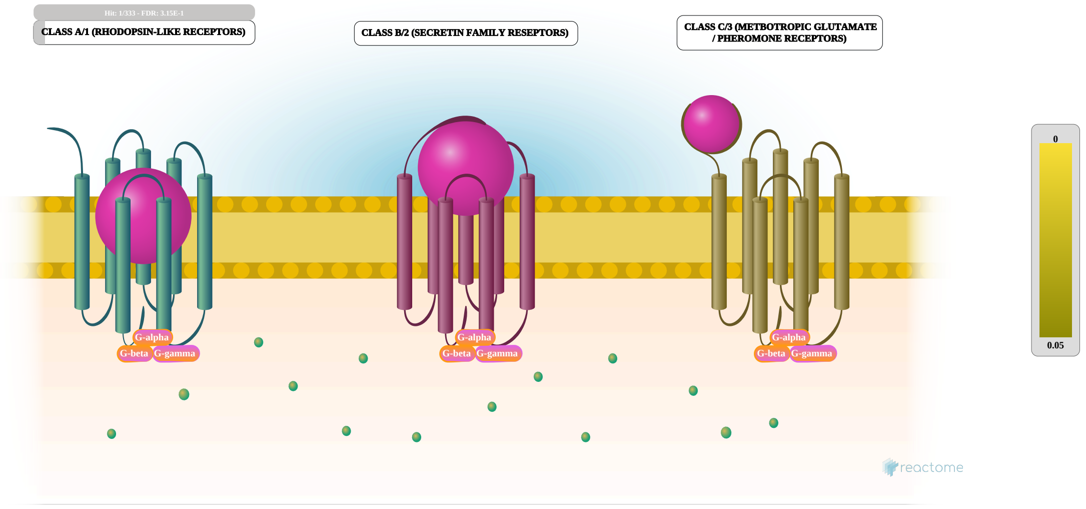

Cellular compartments: plasma membrane.

There are more than 800 G-protein coupled receptor (GPCRs) in the human genome, making it the largest receptor superfamily. GPCRs are also the largest class of drug targets, involved in virtually all physiological processes (Frederiksson 2003). GPCRs are receptors for a diverse range of ligands from large proteins to photons (Kristiansen et al. 2004) and have an equal diversity of ligand-binding mechanisms (Gether et al. 2002). Classical GPCR signaling involves signal transduction via heterotrimeric G-proteins, though G-protein independent mechanisms have been reported.

Rhodopsin-like receptors (class A/1) are by far the largest group of GPCRs and the best studied, though a large proportion of the functional and structural studies have focused on a very few members; many remain functionally uncharacterized. This large family can be subdivided into at least 19 subfamilies (Subfamily A1-19) based on phylogenetic analysis (Joost & Methner 2002). Family A includes receptors for a wide variety of ligands including hormones, light and neurotransmitters, encompassing a wide range of functions including many autocrine, paracrine and endocrine processes.

The secretin-like family B/2 GPCRs includes receptors for many hormone-like peptides, such as secretin, calcitonin, parathyroid hormone/parathyroid hormone-related peptides and vasoactive intestinal peptide, which activate adenylyl cyclase and the phosphatidyl-inositol-calcium pathway (Harmar 2001).

The class C/3 GPCRs include the metabotropic glutamate receptors and taste receptors (Brauner-Osborne et al. 2007). All have a large extracellular N-terminus that structurally resembles a clamshell and has an important role in ligand binding.

References

Kristiansen K (2004). Molecular mechanisms of ligand binding, signaling, and regulation within the superfamily of G-protein-coupled receptors: molecular modeling and mutagenesis approaches to receptor structure and function. Pharmacol Ther, 103, 21-80.

Edit history

Date	Action	Author
2009-12-12	Reviewed	D'Eustachio P
2010-02-05	Authored	Jassal B
2010-02-05	Created	Jassal B
2010-02-10	Edited	Jupe S
2023-05-21	Modified	Wright A

1 submitted entities found in this pathway, mapping to 1 Reactome entities

Input	UniProt Id
Gpr1	Q9Y5Y4

20. GPCR downstream signalling (R-HSA-388396)

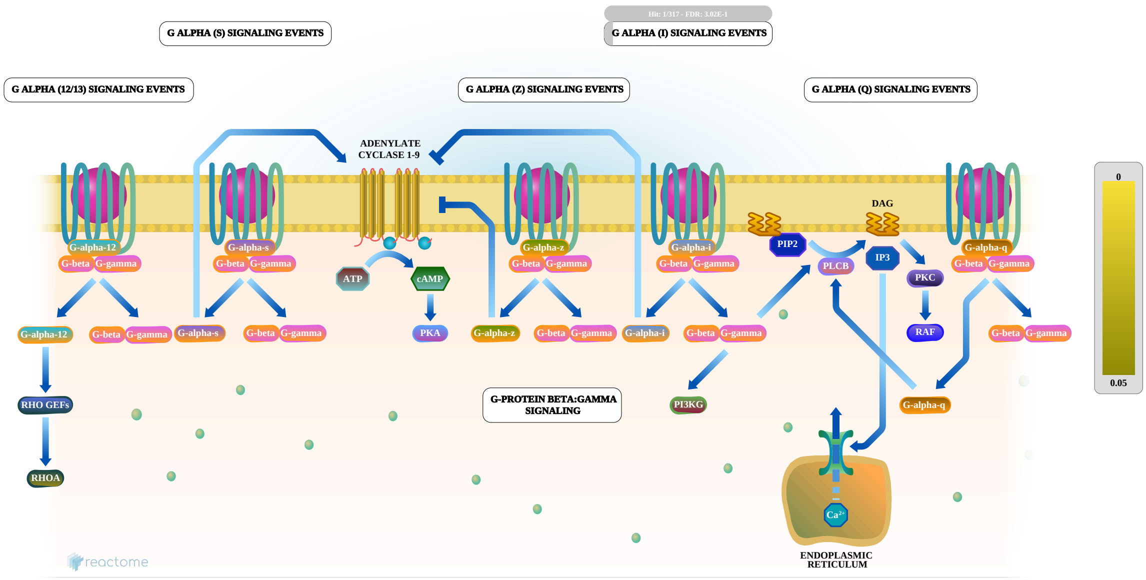

Cellular compartments: plasma membrane.

G protein-coupled receptors (GPCRs) are classically defined as the receptor, G-protein and downstream effectors, the alpha subunit of the G-protein being the primary signaling molecule. However, it has become clear that this greatly oversimplifies the complexities of GPCR signaling (Gurevich & Gurevich 2008). The beta:gamma G-protein dimer is also involved in downstream signaling (Smrcka 2008) and some receptors form metastable complexes with accessory proteins such as the arrestins.

GPCRs are involved in many diverse signaling events (Kristiansen 2004), using a variety of pathways that include modulation of adenylyl cyclase, phospholipase C, the mitogen activated protein kinases (MAPKs), extracellular signal regulated kinase (ERK) c-Jun-NH2-terminal kinase (JNK) and p38 MAPK. Regulator of G-protein Signalling (RGS) proteins can directly inhibit the activity of the G-alpha subunit (Soundararajan et al. 2008).

The general function of the G alpha-s subunit (Gs) is to activate adenylate cyclase (Tesmer et al. 1997), which in turn produces cyclic-AMP (cAMP), leading to the activation of cAMP-dependent protein kinases (often referred to collectively as Protein Kinase A). The signal from the ligand-stimulated GPCR is amplified because the receptor can activate several Gs heterotrimers before it is inactivated.

The classical signalling mechanism for G alpha-i (Gi) is inhibition of the cAMP dependent pathway through inhibition of adenylate cyclase (Dessauer et al. 2002). Decreased production of cAMP results in decreased activity of cAMP-dependent protein kinases.

G alpha-z (Gz) is a member of the Gi family. Unlike other Gi family members it is pertussis toxin-insensitive. Gz interacts with Rap1 GTPase activating protein (RAP1GAP) to attenuate Rap1 signaling.

The classic signalling route for G alpha-q (Gq) is activation of phospholipase C beta, leading to phosphoinositide hydrolysis, calcium mobilization and protein kinase C activation. This provides a path to calcium-regulated kinases and phosphatases, GEFs, MAP kinases and many other proteins.

The G-alpha-12/13 (G12/13) family is probably the least well characterized, at least in part because G12/13 coupling is more difficult to determine than for other subtypes, G12/13 is best known for involvement in the processes of cell proliferation and morphology, such as stress fiber and focal adhesion formation. Interactions with Rho guanine nucleotide exchange factors (RhoGEFs) are thought to mediate many of these processes. (Buhl et al.1995, Sugimoto et al. 2003). Activation of Rho or the regulation of events through Rho is often taken as evidence of G12/13 signaling. Receptors that are coupled with G12/13 invariably couple with one or more other G protein subtypes, usually Gq.

References

Iyengar R, Ram PT & Neves SR (2002). G protein pathways. Science, 296, 1636-9.

Edit history

Date	Action	Author
2008-12-03	Created	Jassal B
2009-03-26	Authored	Jupe S

Date	Action	Author
2009-06-03	Reviewed	Akkerman JW
2009-09-10	Edited	Jupe S
2023-05-21	Modified	Wright A

1 submitted entities found in this pathway, mapping to 1 Reactome entities

Input	UniProt Id
Gpr1	Q9Y5Y4

21. Signaling by GPCR (R-HSA-372790)

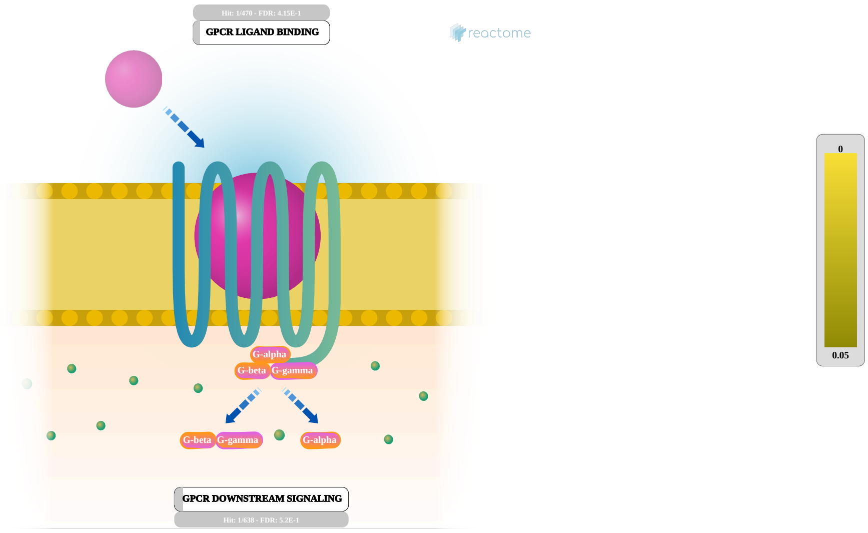

G protein-coupled receptors (GPCRs; 7TM receptors; seven transmembrane domain receptors; heptahelical receptors; G protein-linked receptors [GPLR]) are the largest family of transmembrane receptors in humans, accounting for more than 1% of the protein-coding capacity of the human genome. All known GPCRs share a common architecture of seven membrane-spanning helices connected by intra- and extracellular loops. The extracellular loops contain two highly-conserved cysteine residues that form disulphide bonds to stabilize the structure of the receptor. They recognize diverse messengers such as light, odorants, small molecules, hormones and neurotransmitters. Most GPCRs act as guanine nucleotide exchange factors; activated by ligand binding, they promote GDP-GTP exchange on associated heterotrimeric guanine nucleotide-binding (G) proteins. There are two models for GPCR-G Protein interactions: 1) ligand-GPCR binding first, then binding to G Proteins; 2) "Pre-coupling" of GPCRs and G Proteins before ligand binding (review Oldham WM and Hamm HE, 2008). These in turn activate effector enzymes or ion channels. GPCRs are involved in a range of physiological roles which include the visual sense, smell, behavioural regulation, functions of the autonomic nervous system and regulation of the immune system and inflammation.

GPCRs are divided into classes based on sequence homology and functional similarity. The main mammalian classes, in order of size, are the Rhodopsin-like family A, the Secretin receptor family B, and the Metabotropic glutamate/pheromone receptor family C.

References

Bockaert J & Pin JP (1999). Molecular tinkering of G protein-coupled receptors: an evolutionary success. EMBO J, 18, 1723-9.

Oldham WM & Hamm HE (2008). Heterotrimeric G protein activation by G-protein-coupled receptors. Nat Rev Mol Cell Biol, 9, 60-71.

Bouhelal R, Jacoby E, Gerspacher M & Seuwen K (2006). The 7 TM G-protein-coupled receptor target family. ChemMedChem, 1, 761-82.

Edit history

Date	Action	Author
2008-07-02	Authored	Jassal B
2008-07-02	Created	Jassal B
2008-09-01	Edited	D'Eustachio P
2008-09-01	Reviewed	Bockaert J
2023-05-21	Modified	Wright A

1 submitted entities found in this pathway, mapping to 1 Reactome entities

Input	UniProt Id
Gpr1	Q9Y5Y4

22. Transport of small molecules (R-HSA-382551)

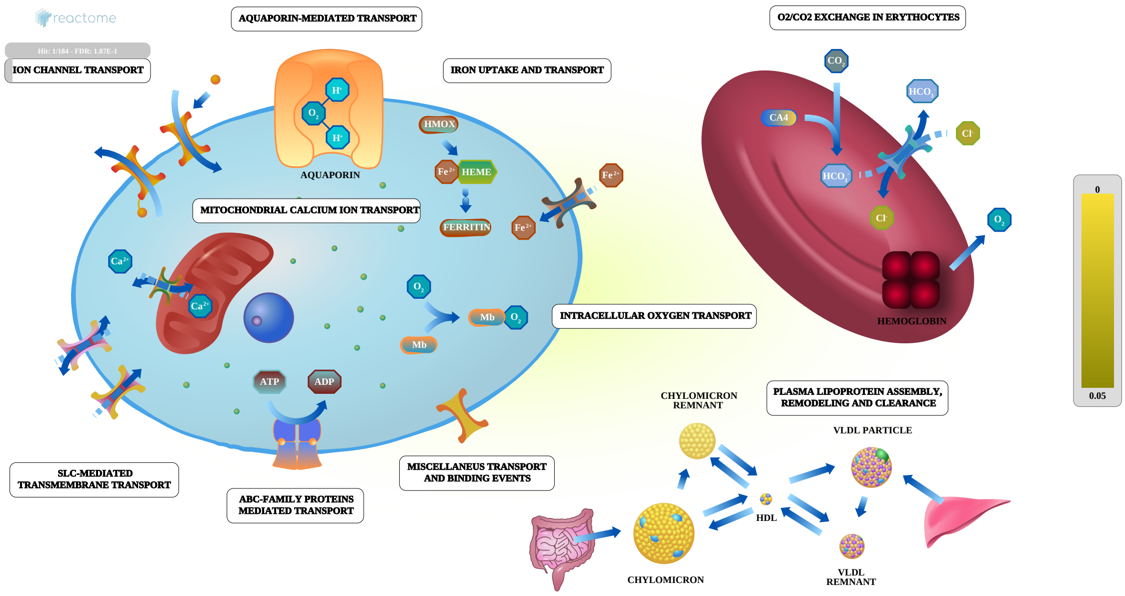

By definition cells have a critical separation between inner (cytoplasmic) and outer (extracellular) compartments. This separation provides for protection, gradient assembly, and environmental control but at the same time isolates the interior compartments of the cell from energy resources, oxygen, and raw materials. Cells have evolved a myriad of mechanisms to regulate, and enable transportation of small molecules ascross plasma membranes and between cellular organelle compartments within cells.

References

Edit history

Date	Action	Author
2008-11-23	Created	Gopinathrao G
2008-12-02	Reviewed	Jassal B, Wright EM, Matthews L
2014-06-09	Revised	Jassal B
2023-05-21	Modified	Wright A

1 submitted entities found in this pathway, mapping to 1 Reactome entities

Input	UniProt Id
Asic4	Q96FT7

23. Metabolism of lipids (R-HSA-556833)

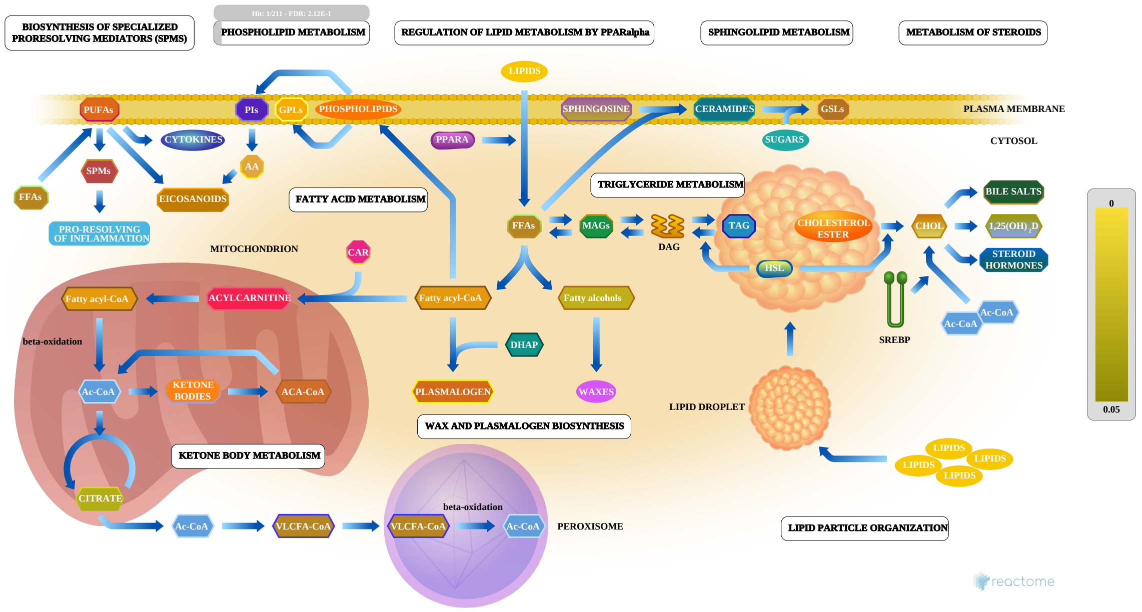

Lipids are hydrophobic but otherwise chemically diverse molecules that play a wide variety of roles in human biology. They include ketone bodies, fatty acids, triacylglycerols, phospholipids and sphingolipids, eicosanoids, cholesterol, bile salts, steroid hormones, and fat-soluble vitamins. They function as a major source of energy (fatty acids, triacylglycerols, and ketone bodies), are major constituents of cell membranes (cholesterol and phospholipids), play a major role in their own digestion and uptake (bile salts), and participate in numerous signaling and regulatory processes (steroid hormones, eicosanoids, phosphatidylinositols, and sphingolipids) (Vance & Vance 2008 - URL).

The central steroid in human biology is cholesterol, obtained from animal fats consumed in the diet or synthesized de novo from acetyl-coenzyme A. (Vegetable fats contain various sterols but no cholesterol.) Cholesterol is an essential constituent of lipid bilayer membranes and is the starting point for the biosyntheses of bile acids and salts, steroid hormones, and vitamin D. Bile acids and salts are mostly synthesized in the liver. They are released into the intestine and function as detergents to solubilize dietary fats. Steroid hormones are mostly synthesized in the adrenal gland and gonads. They regulate energy metabolism and stress responses (glucocorticoids), salt balance (mineralocorticoids), and sexual development and function (androgens and estrogens). At the same time, chronically elevated cholesterol levels in the body are associated with the formation of atherosclerotic lesions and hence increased risk of heart attacks and strokes. The human body lacks a mechanism for degrading excess cholesterol, although an appreciable amount is lost daily in the form of bile salts and acids that escape recycling.

Aspects of lipid metabolism currently annotated in Reactome include lipid digestion, mobilization, and transport; fatty acid, triacylglycerol, and ketone body metabolism; peroxisomal lipid metabolism; phospholipid and sphingolipid metabolism; cholesterol biosynthesis; bile acid and bile salt metabolism; and steroid hormone biosynthesis.

References

Biochemistry of Lipids, Lipoproteins and Membranes (Fifth Edition). Retrieved from http://www.sciencedirect.com/science/book/9780444532190

Edit history

Date	Action	Author
2007-02-03	Authored	Jassal B, Gopinathrao G, D'Eustachio P, Gillespie ME
2010-03-23	Created	D'Eustachio P
2017-02-21	Revised	D'Eustachio P
2023-05-19	Edited	Joshi-Tope G, D'Eustachio P
2023-05-21	Modified	Wright A

1 submitted entities found in this pathway, mapping to 1 Reactome entities

Input	UniProt Id
Etnppl	Q8TBG4

24. Metabolism of proteins (R-HSA-392499)

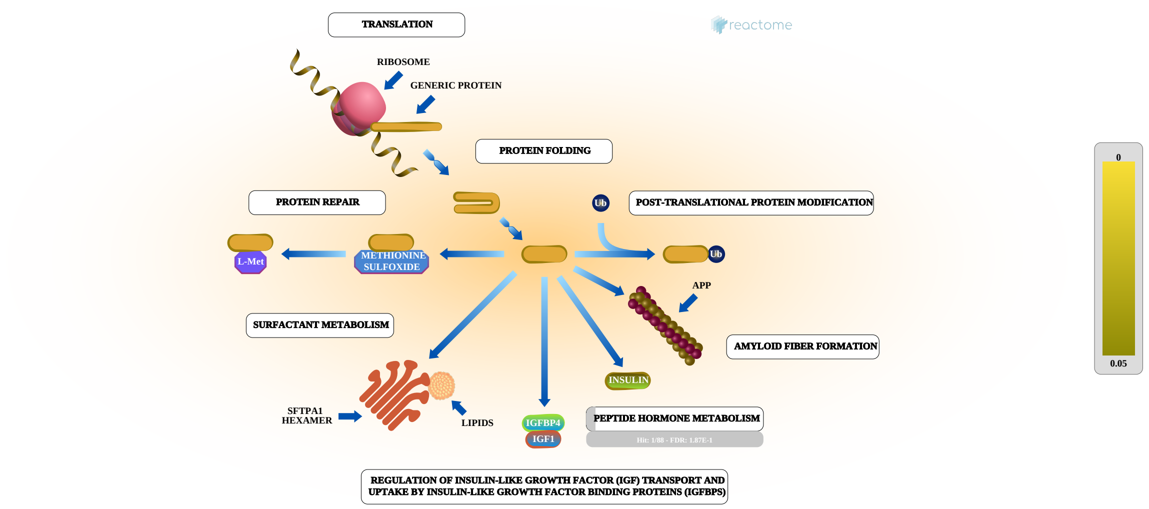

Metabolism of proteins, as annotated here, covers the full life cycle of a protein from its synthesis to its posttranslational modification and degradation, at various levels of specificity. Protein synthesis is accomplished through the process of Translation of an mRNA sequence into a polypeptide chain. Protein folding is achieved through the function of molecular chaperones which recognize and associate with proteins in their non-native state and facilitate their folding by stabilizing the conformation of productive folding intermediates (Young et al. 2004). Following translation, many newly formed proteins undergo Post-translational protein modification, essentially irreversible covalent modifications critical for their mature locations and functions (Knorre et al. 2009), including gamma carboxylation, synthesis of GPI-anchored proteins, asparagine N-linked glycosylation, O-glycosylation, SUMOylation, ubiquitination, deubiquitination, RAB geranylgeranylation, methylation, carboxyterminal post-translational modifications, neddylation, and phosphorylation. Peptide hormones are synthesized as parts of larger precursor proteins whose cleavage in the secretory system (endoplasmic reticulum, Golgi apparatus, secretory granules) is annotated in Peptide hormone metabolism. After secretion, peptide hormones are modified and degraded by extracellular proteases (Chertow, 1981 PMID:6117463). Protein repair enables the reversal of damage to some amino acid side chains caused by reactive oxygen species. Pulmonary surfactants are lipids and proteins that are secreted by the alveolar cells of the lung that decrease surface tension at the air/liquid interface within the alveoli to maintain the stability of pulmonary tissue (Agassandian and Mallampalli 2013). Nuclear regulation, transport, metabolism, reutilization, and degradation of surfactant are described in the Surfactant metabolism pathway. Amyloid fiber formation, the accumulation of mostly extracellular deposits of fibrillar proteins, is associated with tissue damage observed in numerous diseases including late phase heart failure (cardiomyopathy) and neurodegenerative diseases such as Alzheimer's, Parkinson's, and Huntington's.

References

Edit history

Date	Action	Author
2009-03-04	Edited	Matthews L

Date	Action	Author
2009-03-05	Authored	Matthews L
2009-03-05	Created	Matthews L
2023-05-21	Modified	Wright A

1 submitted entities found in this pathway, mapping to 1 Reactome entities

Input	UniProt Id
Pcsk1	P29120

25. Metabolism (R-HSA-1430728)

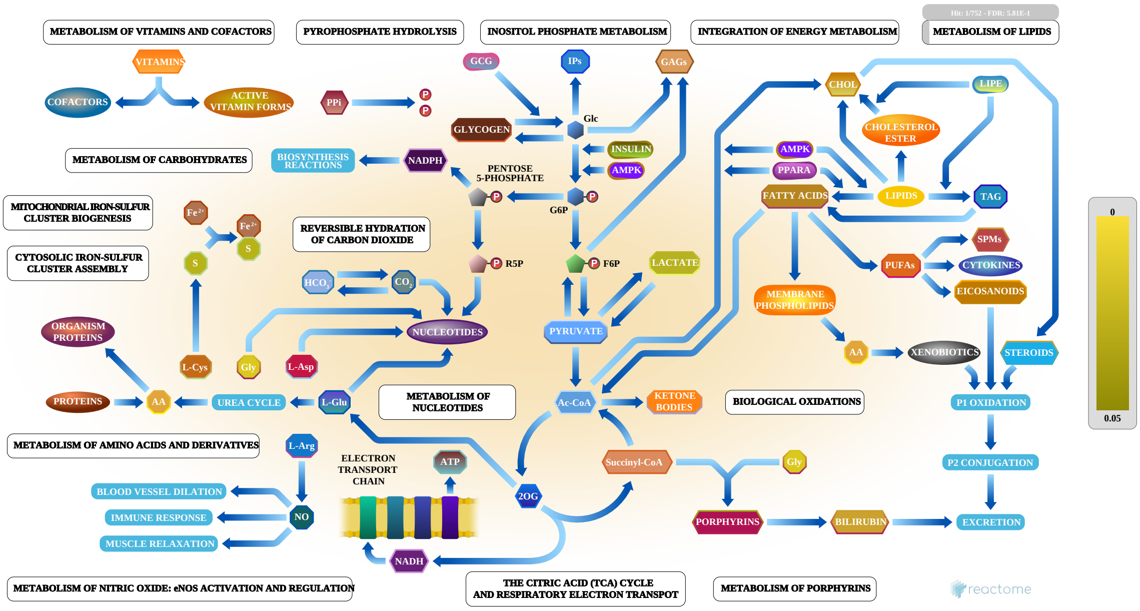

Metabolic processes in human cells generate energy through the oxidation of molecules consumed in the diet and mediate the synthesis of diverse essential molecules not taken in the diet as well as the inactivation and elimination of toxic ones generated endogenously or present in the extracellular environment. The processes of energy metabolism can be classified into two groups according to whether they involve carbohydrate-derived or lipid-derived molecules, and within each group it is useful to distinguish processes that mediate the breakdown and oxidation of these molecules to yield energy from ones that mediate their synthesis and storage as internal energy reserves. Synthetic reactions are conveniently grouped by the chemical nature of the end products, such as nucleotides, amino acids and related molecules, and porphyrins. Detoxification reactions (biological oxidations) are likewise conveniently classified by the chemical nature of the toxin.

At the same time, all of these processes are tightly integrated. Intermediates in reactions of energy generation are starting materials for biosyntheses of amino acids and other compounds, broad-specificity oxidoreductase enzymes can be involved in both detoxification reactions and biosyntheses, and hormone-mediated signaling processes function to coordinate the operation of energy-generating and energy-storing reactions and to couple these to other biosynthetic processes.

References

Edit history

Date	Action	Author
2011-07-07	Created	Jassal B
2023-05-21	Modified	Wright A

1 submitted entities found in this pathway, mapping to 1 Reactome entities

Input	UniProt Id
Etnppl	Q8TBG4

6. Identifiers found

Below is a list of the input identifiers that have been found or mapped to an equivalent element in Reactome, classified by resource.

5 of the submitted entities were found, mapping to 5 Reactome entities

Input	UniProt Id	Input	UniProt Id	Input	UniProt Id
Asic4	Q96FT7	Etnppl	Q8TBG4	Gpr1	Q9Y5Y4
Myh8	P13535	Pcsk1	P29120		

7. Identifiers not found

These 8 identifiers were not found neither mapped to any entity in Reactome.

BC030499	Ccdc184	Myh7b	Pdzph1	Prr16	Rims3	Spag5	Trim66
