# Supplementary material for: Sex- and region-specific cortical and hippocampal whole genome transcriptome profiles from control and APP/PS1 Alzheimer’s disease mice
Source: PLoS One. 2024 Feb 7;19(2):e0296959. doi: 10.1371/journal.pone.0296959 (PMC10849391; doi:10.1371/journal.pone.0296959)
Supplement: S1 File — S1 Fig: Genotyping of APP/PS1 AD mice and WT control animals. S2 Fig: 3D image of the murine brain including the RS cortex and hippocampus (BROIs) used for transcriptome analysis in our study. S3 Fig: PCA of transcriptomes from the RS cortex and hippocampus of WT controls and APP/PS1 AD mice of both sexes. S4 Fig: Hierarchical clustering of transcriptome data from the RS cortex and hippocampus of WT control and APP/PS1 AD mice of both sexes. S5 Fig: Bar diagrams of the top 30 candidates of DEGs with highest significant FCs (FC > 1.5 and FC < -1.5, p < 0.05). S6 Fig: Pathway analysis of intersectional and signature gene sets in APP/PS1 subgroups. S7 Fig: Comparative qPCR analysis of selected gene transcript levels from the hippocampus of female and male APP/PS1 AD with 5XFAD mice. S1 Table: PCR reaction set-up using PCR Mastermix and genomic DNA. S2 Table: Materials used for one-color microarray-based gene expression data collection. S3 Table: Software used for one-color microarray-based gene expression data collection. S4 Table: Details on genes, forward and reverse primer sequences and annealing temperatures relevant for qPCR experimentation. S5 Table: Characteristics of DEGs in the RS cortex of female APP/PS1 AD mice. S6 Table: Characteristics of DEGs in the hippocampus of female APP/PS1 AD mice. S7 Table: Characteristics of DEGs in the RS cortex of male APP/PS1 AD mice. S8 Table: Characteristics of DEGs in the hippocampus of male APP/PS1 AD mice. S9 Table: Venn analysis of DEGs in the RS cortex and hippocampus of female APP/PS1 AD mice. S10 Table: Venn analysis of DEGs genes in the RS cortex and hippocampus of male APP/PS1 AD mice. S11 Table: Venn analysis of DEGs in the RS cortex of male and female APP/PS1 AD mice. S12 Table: Venn analysis of DEGs in the hippocampus of male and female APP/PS1 AD mice. S13 Table: Differentially regulated l(i)ncRNAs in APP/PS1 AD vs. WT mice. S14 Table: qPCR-based FC analysis of selected genes in the hippocampus of APP/PS1 AD vs. [file pone.0296959.s001.zip › Supplementary Files_R1/Supplementary Figure 6_Pathways_downreg genes/Signature_down_DEGs_female_RS Cx_APPPS1/Pathway analysis report.pdf]

Pathway Analysis Report

This report contains the pathway analysis results for the submitted sample ''. Analysis was performed against Reactome version 85 on 17/08/2023. The web link to these results is:

https://reactome.org/PathwayBrowser/#/ANALYSIS=MjAyMzA4MTcwNjUxMDJfMjExOTk%3D

Please keep in mind that analysis results are temporarily stored on our server. The storage period depends on usage of the service but is at least 7 days. As a result, please note that this URL is only valid for a limited time period and it might have expired.

Table of Contents

1. Introduction
2. Properties
3. Genome-wide overview
4. Most significant pathways
5. Pathways details
6. Identifiers found
7. Identifiers not found

1. Introduction

Reactome is a curated database of pathways and reactions in human biology. Reactions can be considered as pathway 'steps'. Reactome defines a 'reaction' as any event in biology that changes the state of a biological molecule. Binding, activation, translocation, degradation and classical biochemical events involving a catalyst are all reactions. Information in the database is authored by expert biologists, entered and maintained by Reactome's team of curators and editorial staff. Reactome content frequently cross-references other resources e.g. NCBI, Ensembl, UniProt, KEGG (Gene and Compound), ChEBI, PubMed and GO. Orthologous reactions inferred from annotation for Homo sapiens are available for 14 non-human species including mouse, rat, chicken, puffer fish, worm, fly and yeast. Pathways are represented by simple diagrams following an SBGN-like format.

Reactome's annotated data describe reactions possible if all annotated proteins and small molecules were present and active simultaneously in a cell. By overlaying an experimental dataset on these annotations, a user can perform a pathway over-representation analysis. By overlaying quantitative expression data or time series, a user can visualize the extent of change in affected pathways and its progression. A binomial test is used to calculate the probability shown for each result, and the p-values are corrected for the multiple testing (Benjamini–Hochberg procedure) that arises from evaluating the submitted list of identifiers against every pathway.

To learn more about our Pathway Analysis, please have a look at our relevant publications:

Fabregat A, Sidiropoulos K, Garapati P, Gillespie M, Hausmann K, Haw R, … D'Eustachio P (2016). The reactome pathway knowledgebase. Nucleic Acids Research, 44(D1), D481–D487. https://doi.org/10.1093/nar/gkv1351.

Fabregat A, Sidiropoulos K, Viteri G, Forner O, Marin-Garcia P, Arnau V, … Hermjakob H (2017). Reactome pathway analysis: a high-performance in-memory approach. BMC Bioinformatics, 18.

2. Properties

- This is an **overrepresentation** analysis: A statistical (hypergeometric distribution) test that determines whether certain Reactome pathways are over-represented (enriched) in the submitted data. It answers the question 'Does my list contain more proteins for pathway X than would be expected by chance?' This test produces a probability score, which is corrected for false discovery rate using the Benjamani-Hochberg method.
- 1 out of 2 identifiers in the sample were found in Reactome, where 43 pathways were hit by at least one of them.
- All non-human identifiers have been converted to their human equivalent.
- This report is filtered to show only results for species 'Homo sapiens' and resource 'all resources'.
- The unique ID for this analysis (token) is MjAyMzA4MTcwNjUxMDJfMjExOTk%3D. This ID is valid for at least 7 days in Reactome's server. Use it to access Reactome services with your data.

3. Genome-wide overview

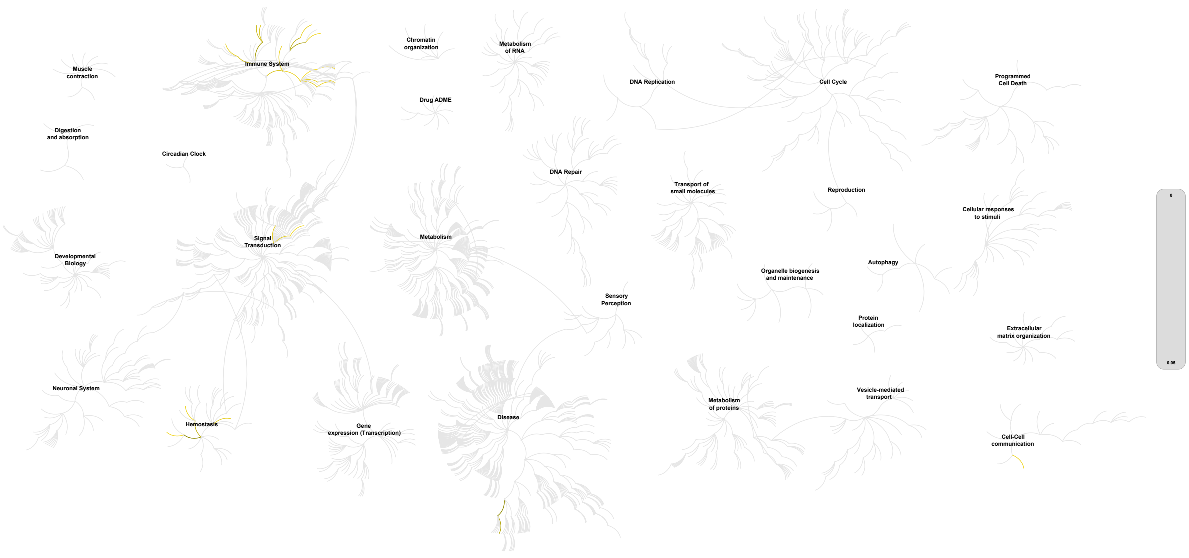

This figure shows a genome-wide overview of the results of your pathway analysis. Reactome pathways are arranged in a hierarchy. The center of each of the circular "bursts" is the root of one top-level pathway, for example "DNA Repair". Each step away from the center represents the next level lower in the pathway hierarchy. The color code denotes over-representation of that pathway in your input dataset. Light grey signifies pathways which are not significantly over-represented.

4. Most significant pathways

The following table shows the 25 most relevant pathways sorted by p-value.

Pathway name	Entities				Reactions	
	found	ratio	p-value	FDR*	found	ratio
Interleukin-37 signaling	2 / 36	0.002	1.67e-05	7.36e-04	1 / 14	9.79e-04
Interleukin-1 family signaling	2 / 183	0.012	4.30e-04	0.009	1 / 92	0.006
PECAM1 interactions	1 / 14	9.19e-04	0.003	0.017	1 / 7	4.89e-04
Regulation of IFNG signaling	1 / 16	0.001	0.003	0.017	1 / 4	2.80e-04
Regulation of KIT signaling	1 / 18	0.001	0.004	0.017	1 / 8	5.59e-04
Signal regulatory protein family interactions	1 / 18	0.001	0.004	0.017	1 / 10	6.99e-04
Platelet sensitization by LDL	1 / 23	0.002	0.005	0.017	1 / 9	6.29e-04
Signaling by Interleukins	2 / 658	0.043	0.005	0.017	4 / 505	0.035
Regulation of IFNA/IFNB signaling	1 / 28	0.002	0.006	0.017	1 / 5	3.49e-04
Interleukin receptor SHC signaling	1 / 29	0.002	0.006	0.017	1 / 6	4.19e-04
Growth hormone receptor signaling	1 / 29	0.002	0.006	0.017	2 / 28	0.002
PD-1 signaling	1 / 33	0.002	0.006	0.017	2 / 5	3.49e-04
GPVI-mediated activation cascade	1 / 43	0.003	0.008	0.017	3 / 25	0.002
Signaling by ALK	1 / 43	0.003	0.008	0.017	1 / 40	0.003
Interleukin-2 family signaling	1 / 47	0.003	0.009	0.018	1 / 59	0.004
Interleukin-3, Interleukin-5 and GM-CSF signaling	1 / 50	0.003	0.01	0.02	3 / 38	0.003
Signaling by SCF-KIT	1 / 51	0.003	0.01	0.02	1 / 39	0.003
Cytokine Signaling in Immune system	2 / 1,039	0.068	0.013	0.023	10 / 745	0.052
CD22 mediated BCR regulation	1 / 72	0.005	0.014	0.023	1 / 4	2.80e-04
Costimulation by the CD28 family	1 / 87	0.006	0.017	0.023	3 / 35	0.002
Antigen activates B Cell Receptor (BCR) leading to generation of second messengers	1 / 103	0.007	0.02	0.023	2 / 26	0.002
Platelet homeostasis	1 / 118	0.008	0.023	0.023	1 / 31	0.002
Interferon alpha/beta signaling	1 / 129	0.008	0.025	0.025	2 / 25	0.002
SARS-CoV-2 activates/modulates innate and adaptive immune responses	1 / 160	0.011	0.031	0.031	1 / 47	0.003
Cell-Cell communication	1 / 165	0.011	0.032	0.032	1 / 93	0.007

* False Discovery Rate

5. Pathways details

For every pathway of the most significant pathways, we present its diagram, as well as a short summary, its bibliography and the list of inputs found in it.

1. Interleukin-37 signaling (R-HSA-9008059)

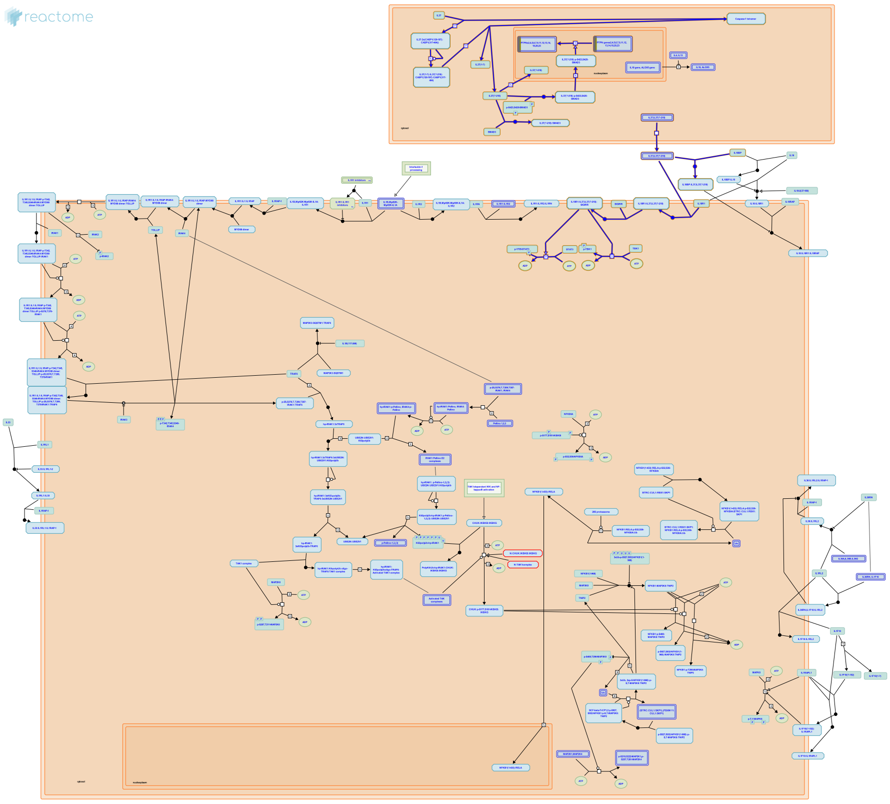

Cellular compartments: plasma membrane, extracellular region, cytosol.

Interleukins (IL) are immunomodulatory proteins that elicit a wide array of responses in cells and tissues. Interleukin 37 (IL37), also known as IL 1F7, is a member of the IL 1 family (Sharma et al. 2008). Isoform b of IL37 (referred just as IL37) is synthesized as a precursor that requires processing (primarily by caspase 1) to attain full receptor agonist or antagonist function (Kumar et al. 2002). Both full length and processed IL37 can bind to the IL 18 binding protein (IL 18BP) and the Interleukin 18 receptor 1 (IL 18R1) (Shi et al. 2003). Upon binding to the IL18R1, IL37 recruits Single Ig IL 1 related receptor (SIGIRR) (Nold-Petry et al. 2015). The IL37:IL18R1 complex can activate phosphorylation of Signal transducer and activator of transcription 3 (STAT3), Tyrosine protein kinase Mer and Phosphatidylinositol 3,4,5 trisphosphate 3 phosphatase and dual specificity protein phosphatase PTEN and can also inhibit Nuclear factor NF kappa B p105 subunit (NFKB) (Nold-Petry et al. 2015). Processed IL37 can be secreted from the cytosol to the extracellular space or translocated into the nucleus (Bulau et al. 2014). Full length IL37 can also be secreted from the cytosol to the extracellular space (Bulau et al. 2014). Processed IL37 can bind with Mothers against decapentaplegic homolog 3 (SMAD3) in the cytosol and then translocate to the nucleus, where it facilitates transcription of Tyrosine protein phosphatase non receptors (PTPNs) (Nold et al. 2010, Luo et al. 2017). These events ultimately lead to suppression of cytokine production in several types of immune cells resulting in reduced inflammation.

References

Dinarello CA, Italiani P, Pfaller T, Pixner C, Nold MF, Lucchesi D, ... Boraschi D (2011). IL-37: a new anti-inflammatory cytokine of the IL-1 family. Eur. Cytokine Netw., 22, 127-47.

Shi H, Wu B, Luo X, Li J, Zhuang X & Jin B (2017). The emerging role of interleukin-37 in cardiovascular diseases. Immun Inflamm Dis.

Edit history

Date	Action	Author
2017-06-07	Created	Varusai TM
2017-08-08	Edited	Varusai TM
2017-08-08	Authored	Varusai TM
2017-11-02	Reviewed	Carriero R, Garlanda C, Mantovani A
2023-05-21	Modified	Wright A

1 submitted entities found in this pathway, mapping to 2 Reactome entities

Input	UniProt Id
Ptpn6	P29350

Input	Ensembl Id
Ptpn6	ENSG00000111679

2. Interleukin-1 family signaling (R-HSA-446652)

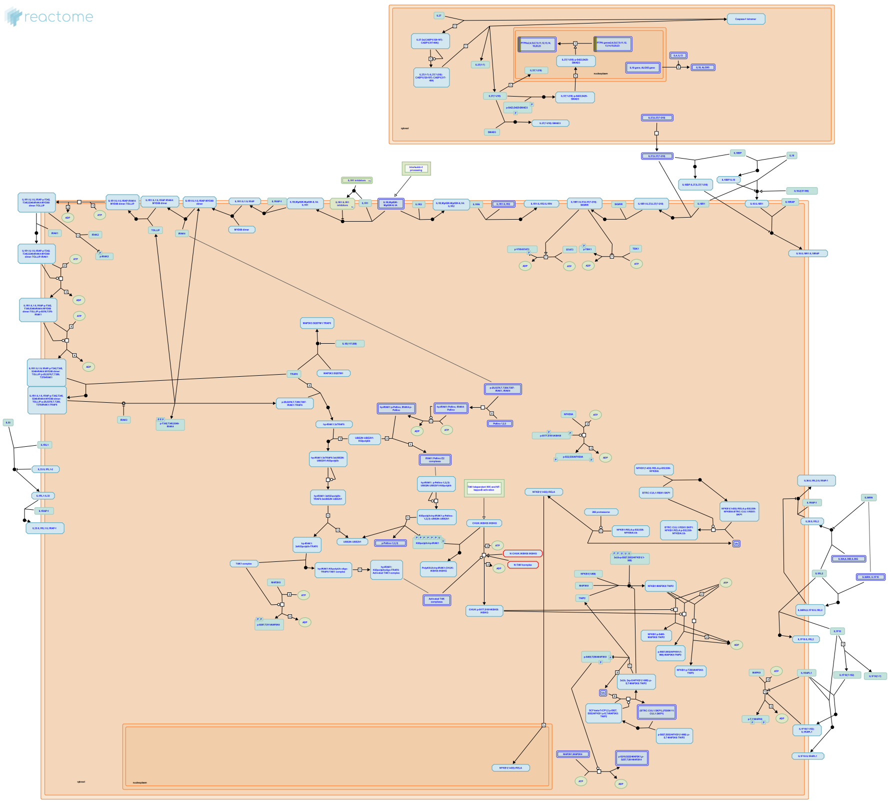

Cellular compartments: plasma membrane.

The Interleukin-1 (IL1) family of cytokines comprises 11 members, namely Interleukin-1 alpha (IL1A), Interleukin-1 beta (IL1B), Interleukin-1 receptor antagonist protein (IL1RN, IL1RA), Interleukin-18 (IL18), Interleukin-33 (IL33), Interleukin-36 receptor antagonist protein (IL36RN, IL36RA), Interleukin-36 alpha (IL36A), Interleukin-36 beta (IL36B), Interleukin-36 gamma (IL36G), Interleukin-37 (IL37) and Interleukin-38 (IL38). The genes encoding all except IL18 and IL33 are on chromosome 2. They share a common C-terminal three-dimensional structure and with apart from IL1RN they are synthesized without a hydrophobic leader sequence and are not secreted via the classical reticulum endoplasmic-Golgi pathway.
IL1B and IL18, are produced as biologically inactive propeptides that are cleaved to produce the mature, active interleukin peptide.

The IL1 receptor (IL1R) family comprises 10 members: Interleukin-1 receptor type 1 (IL1R1, IL1RA), Interleukin-1 receptor type 2 (IL1R2, IL1RB), Interleukin-1 receptor accessory protein (IL1RAP, IL1RAcP, IL1R3), Interleukin-18 receptor 1 (IL18R1, IL18RA) , Interleukin-18 receptor accessory protein (IL18RAP, IL18RB), Interleukin-1 receptor-like 1 (IL1RL1, ST2, IL33R), Interleukin-1 receptor-like 2 (IL1RL2, IL36R), Single Ig IL-1-related receptor (SIGIRR, TIR8), Interleukin-1 receptor accessory protein-like 1 (IL1RAPL1, TIGGIR2) and X-linked interleukin-1 receptor accessory protein-like 2 (IL1RAPL2, TIGGIR1). Most of the genes encoding these receptors are on chromosome 2.

IL1 family receptors heterodimerize upon cytokine binding. IL1, IL33 and IL36 bind specific receptors, IL1R1, IL1RL1, and IL1RL2 respectively. All use IL1RAP as a co-receptor. IL18 binds IL18R1 and uses IL18RAP as co-receptor.

The complexes formed by IL1 family cytokines and their heterodimeric receptors recruit intracellular signaling molecules, including Myeloid differentiation primary response protein MyD88 (MYD88), members of he IL1R-associated kinase (IRAK) family, and TNF receptor-associated factor 6 (TRAF6), activating Nuclear factor NF-kappa-B (NF⊠B), as well as Mitogen-activated protein kinase 14 (MAPK14, p38), c-Jun N-terminal kinases (JNKs), extracellular signal-regulated kinases (ERKs) and other Mitogen-activated protein kinases (MAPKs).

References

Dinarello CA (2009). Immunological and inflammatory functions of the interleukin-1 family. Annu Rev Immunol, 27, 519-50.

Martin P, Dietrich D, Gabay C, Palomo J & Palmer G (2015). The interleukin (IL)-1 cytokine family--Balance between agonists and antagonists in inflammatory diseases. Cytokine, 76, 25-37.

Arend WP, Gabay C & Palmer G (2008). IL-1, IL-18, and IL-33 families of cytokines. Immunol. Rev., 223, 20-38.

Moynagh PN (2009). The Pellino family: IRAK E3 ligases with emerging roles in innate immune signalling. Trends Immunol, 30, 33-42.

Edit history

Date	Action	Author
2009-11-16	Created	Jupe S
2010-05-17	Edited	Jupe S
2010-05-17	Reviewed	Pinteaux E

Date	Action	Author
2010-05-17	Authored	Ray KP
2023-05-21	Modified	Wright A

1 submitted entities found in this pathway, mapping to 2 Reactome entities

Input	UniProt Id
Ptpn6	P29350

Input	Ensembl Id
Ptpn6	ENSG00000111679

3. PECAM1 interactions (R-HSA-210990)

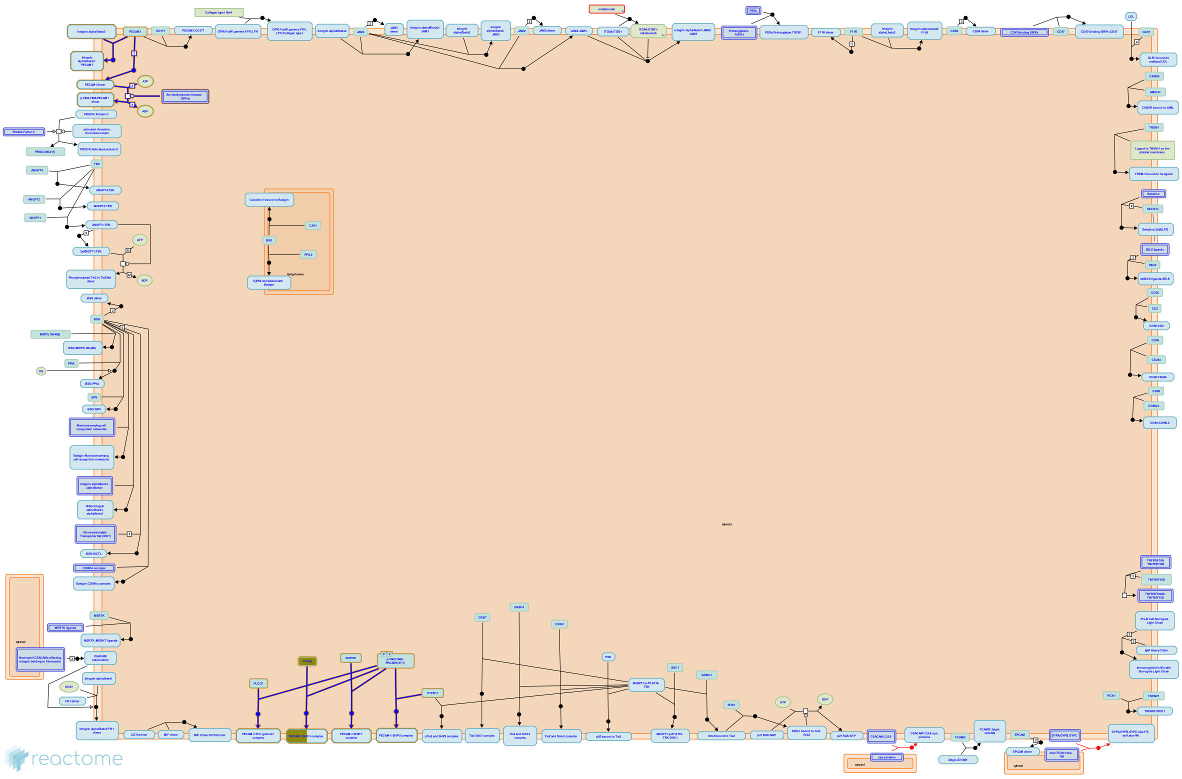

PECAM-1/CD31 is a member of the immunoglobulin superfamily (IgSF) and has been implicated to mediate the adhesion and trans-endothelial migration of T-lymphocytes into the vascular wall, T cell activation and angiogenesis. It has six Ig homology domains within its extracellularly and an ITIM motif within its cytoplasmic region. PECAM-1 mediates cellular interactions by both homophilic and heterophilic interactions. The cytoplasmic domain of PECAM-1 contains tyrosine residues which serves as docking sites for recruitment of cytosolic signaling molecules. Under conditions of platelet activation, PECAM-1 is phosphorylated by Src kinase members. The tyrosine residues 663 and 686 are required for recruitment of the SH2 domain containing PTPs.

References

Jackson DE (2003). The unfolding tale of PECAM-1. FEBS Lett, 540, 7-14.

Gong N & Chatterjee S (2003). Platelet endothelial cell adhesion molecule in cell signaling and thrombosis. Mol Cell Biochem, 253, 151-8.

Edit history

Date	Action	Author
2008-01-22	Created	Garapati P V
2008-02-26	Reviewed	Trowsdale J
2008-02-26	Authored	Garapati P V, de Bono B
2023-05-21	Modified	Wright A

1 submitted entities found in this pathway, mapping to 1 Reactome entities

Input	UniProt Id
Ptpn6	P29350

Input	UniProt Id

4. Regulation of IFNG signaling (R-HSA-877312)

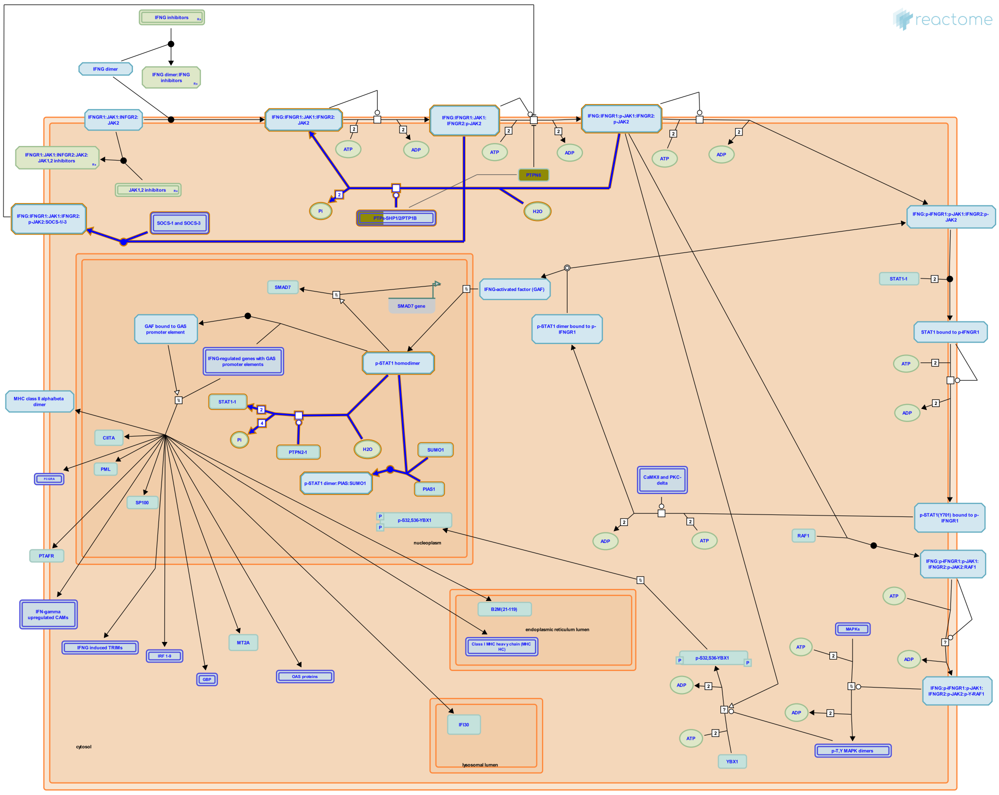

At least three different classes of negative regulators exist to control the extent of INFG stimulation and signaling. These include the feedback inhibitors belonging to protein family suppressors of cytokine signaling (SOCS), the Scr-homology 2 (SH2)-containing protein tyrosine phosphatases (SHPs), and the protein inhibitors of activated STATs (PIAS). The induction of these regulators seems to be able to stop further signal transduction by inhibiting various steps in IFNG cascade.

References

Hilton DJ (1999). Negative regulators of cytokine signal transduction. Cell Mol Life Sci, 55, 1568-77.

Palvimo JJ (2007). PIAS proteins as regulators of small ubiquitin-related modifier (SUMO) modifications and transcription. Biochem Soc Trans, 35, 1405-8.

Röpke C & Larsen L (2002). Suppressors of cytokine signalling: SOCS. APMIS, 110, 833-44.

Edit history

Date	Action	Author
2010-06-08	Edited	Garapati P V
2010-06-08	Authored	Garapati P V
2010-06-11	Created	Garapati P V
2010-08-17	Reviewed	Abdul-Sater AA, Schindler C
2023-05-30	Modified	Wright A

1 submitted entities found in this pathway, mapping to 1 Reactome entities

Input	UniProt Id
Ptpn6	P29350

5. Regulation of KIT signaling (R-HSA-1433559)

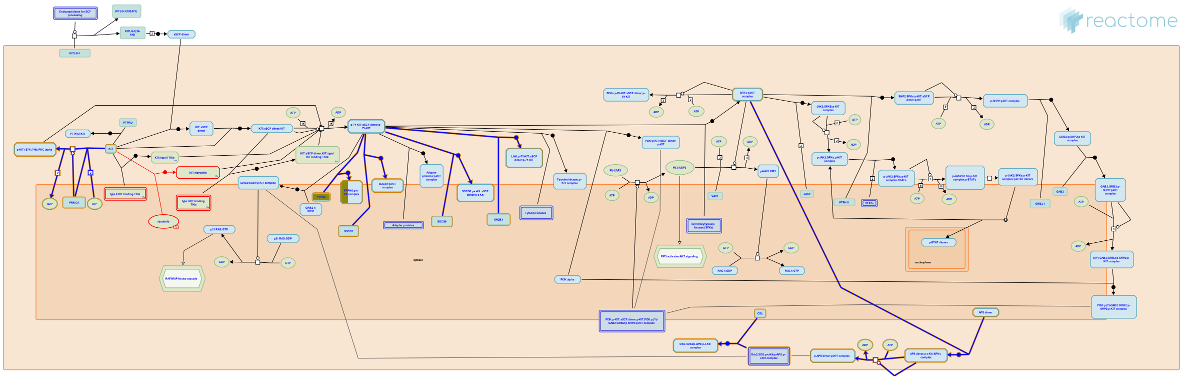

SCF induced proliferation is negatively regulated by various proteins including SHP1, PKC, CBL, SOCS1, SOCS6 and LNK.

References

Frossard N, Da Silva CA & Reber L (2006). Stem cell factor and its receptor c-Kit as targets for inflammatory diseases. Eur J Pharmacol, 533, 327-40.

Linnekin D (1999). Early signaling pathways activated by c-Kit in hematopoietic cells. Int J Biochem Cell Biol, 31, 1053-74.

Edit history

Date	Action	Author
2011-07-11	Edited	Garapati P V
2011-07-11	Authored	Garapati P V
2011-07-11	Created	Garapati P V
2011-08-22	Reviewed	Rönnstrand L
2023-05-21	Modified	Wright A

1 submitted entities found in this pathway, mapping to 1 Reactome entities

Input	UniProt Id
Ptpn6	P29350

6. Signal regulatory protein family interactions (R-HSA-391160)

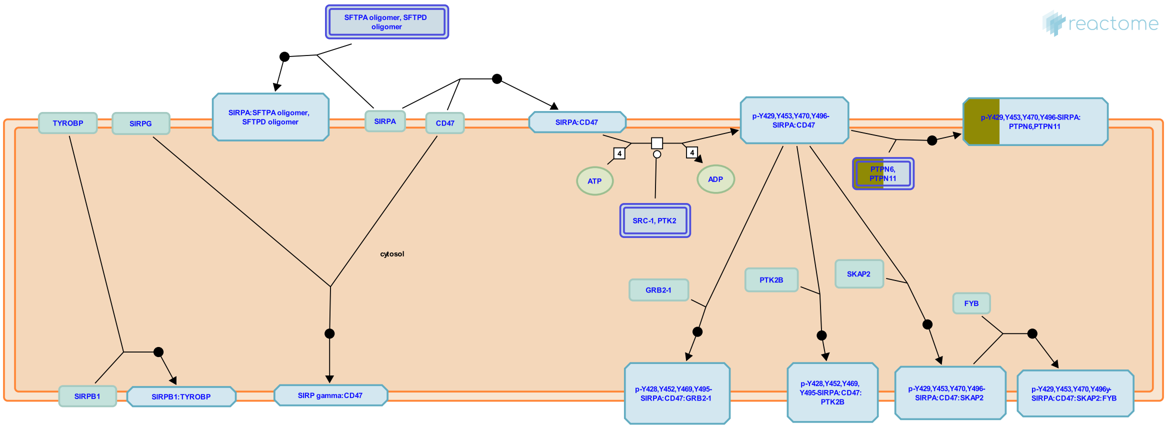

Cellular compartments: plasma membrane.

Signal regulatory protein alpha (SIRPA, SHPS1, CD172a) is a transmembrane protein expressed mostly on myeloid cells. CD47, a widely expressed transmembrane protein, is a ligand for SIRP alpha, with the two proteins constituting a cell-cell communication system. The interaction of SIRPA with CD47 is important for the regulation of migration and phagocytosis. SIRPA functions as a docking protein to recruit and activate PTPN6 (SHP-1) or PTPN11 (SHP-2) at the cell membrane in response to extracellular stimuli. SIRPA also binds other intracellular proteins including the adaptor molecules Src kinase-associated protein (SKAP2 SKAP55hom/R), Fyn-binding protein/SLP-76-associated phosphoprotein (FYB/SLAP-130) and the tyrosine kinase PYK2. SIRPA also binds the extracellular proteins, surfactant-A (SP-A) and surfactant-D (SP-D).

The SIRP family members SIRPB and SIRPG show high sequence similarity and similar extracellular structural topology, including three Ig domains, but their ligand binding topology might differ. SIRPB is expressed on myeloid cells, including monocytes, granulocytes and DCs. It has no known natural ligand. SIRPG can bind CD47 but with lower affinity than SIRPA.

References

Liu Y, Howard CJ, Parkos CA, van den Berg TK, van Beek EM, Vignery A, ... Sano S (2005). A nomenclature for signal regulatory protein family members. J Immunol, 175, 7788-9.

Barclay AN & Brown MH (2006). The SIRP family of receptors and immune regulation. Nat Rev Immunol, 6, 457-64.

Ruhul Amin AR, Matsuda S, Oshima K, Hamaguchi M & Suzuki A (2002). SHPS-1, a multifunctional transmembrane glycoprotein. FEBS Lett, 519, 1-7.

Ohnishi H, Okazawa H, Matozaki T & Murata Y (2009). Functions and molecular mechanisms of the CD47-SIRPalpha signalling pathway. Trends Cell Biol, 19, 72-80.

Parkos CA, Liu Y, Buhring HJ, Zen K, Schnell FJ, Burst SL & Williams IR (2002). Signal regulatory protein (SIRPalpha), a cellular ligand for CD47, regulates neutrophil transmigration. J Biol Chem, 277, 10028-36.

Edit history

Date	Action	Author
2009-02-12	Edited	Garapati P V

Date	Action	Author
2009-02-12	Authored	Garapati P V
2009-02-12	Created	Garapati P V
2010-05-20	Reviewed	Barclay AN
2023-05-21	Modified	Wright A

1 submitted entities found in this pathway, mapping to 1 Reactome entities

Input	UniProt Id
Ptpn6	P29350

7. Platelet sensitization by LDL (R-HSA-432142)

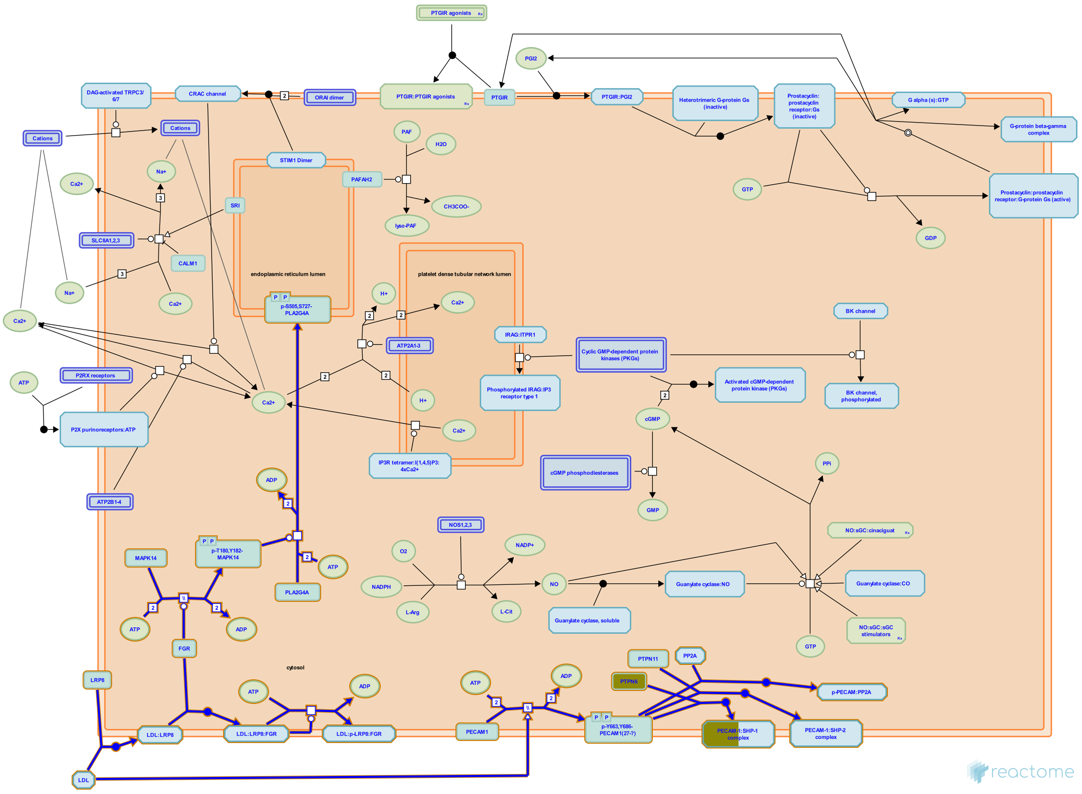

Cellular compartments: plasma membrane.

Physiological concentrations (1g/L) of Low density lipoprotein (LDL) enhance platelet aggregation responses initiated by thrombin, collagen, and ADP. This enhancement involves the rapid phosphorylation of p38 mitogen-activated protein kinase (p38MAPK) at Thr180 and Tyr182. The receptor for LDL is ApoER2, a splice variant of the classical ApoE receptor. ApoER2 stimulation leads to association of the Src family kinase Fgr which is probably responsible for subsequent phosphorylation of p38MAPK. This stimulation is transient because LDL also increases the activity of PECAM-1, which stimulates phosphatases that dephosphorylate p38MAPK.

References

Akkerman JW, Gorter G, Ferreira IA, Relou IA & van Rijn HJ (2003). Platelet endothelial cell adhesion molecule-1 (PECAM-1) inhibits low density lipoprotein-induced signaling in platelets. J Biol Chem, 278, 32638-44.

Akkerman JW, Gorter G, Korporaal SJ, Strasser V, Lenting PJ, Bezemer M, ... van Berkel TJ (2004). Binding of low density lipoprotein to platelet apolipoprotein E receptor 2' results in phosphorylation of p38MAPK. J Biol Chem, 279, 52526-34.

Akkerman JW, Gorter G, van Rijn HJ & Relou AM (2002). Platelet activation by the apoB/E receptor-binding domain of LDL. Thromb Haemost, 87, 880-7.

Edit history

Date	Action	Author
2009-08-11	Created	Jupe S
2009-09-04	Authored	Akkerman JW

Date	Action	Author
2010-06-07	Edited	Jupe S
2010-06-07	Reviewed	Kunapuli SP
2023-05-21	Modified	Wright A

1 submitted entities found in this pathway, mapping to 1 Reactome entities

Input	UniProt Id
Ptpn6	P29350

8. Signaling by Interleukins (R-HSA-449147)

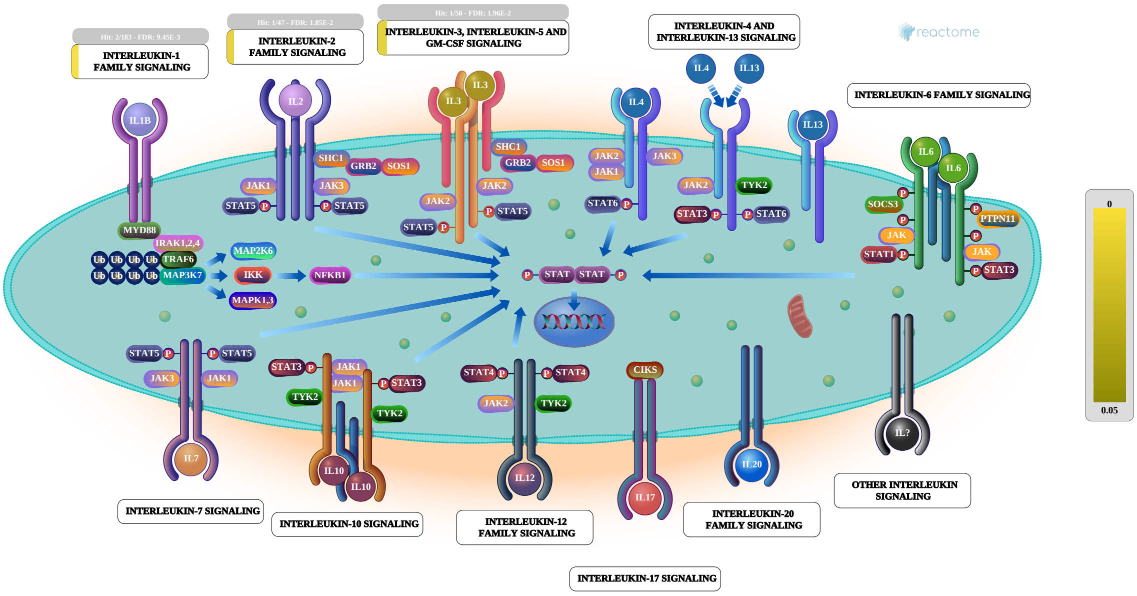

Cellular compartments: plasma membrane.

Interleukins are low molecular weight proteins that bind to cell surface receptors and act in an autocrine and/or paracrine fashion. They were first identified as factors produced by leukocytes but are now known to be produced by many other cells throughout the body. They have pleiotropic effects on cells which bind them, impacting processes such as tissue growth and repair, hematopoietic homeostasis, and multiple levels of the host defense against pathogens where they are an essential part of the immune system.

References

Dinarello CA (2009). Immunological and inflammatory functions of the interleukin-1 family. Annu Rev Immunol, 27, 519-50.

Komlosi Z, Kucuksezer UC, Frei R, Huitema C, Garbani M, Pezer M, ... Eiwegger T (2016). Interleukins (from IL-1 to IL-38), interferons, transforming growth factor ⊠, and TNF-⊠: Receptors, functions, and roles in diseases. J. Allergy Clin. Immunol., 138, 984-1010.

Vosshenrich CA & Di Santo JP (2002). Interleukin signaling. Curr Biol, 12, R760-3.

Edit history

Date	Action	Author
2009-11-27	Created	Jupe S
2010-05-17	Reviewed	Pinteaux E
2010-05-17	Authored	Ray KP
2010-05-26	Edited	Jupe S
2023-05-21	Modified	Wright A

1 submitted entities found in this pathway, mapping to 2 Reactome entities

Input	UniProt Id
Ptpn6	P29350

Input	Ensembl Id
Ptpn6	ENSG00000111679

9. Regulation of IFNA/IFNB signaling (R-HSA-912694)

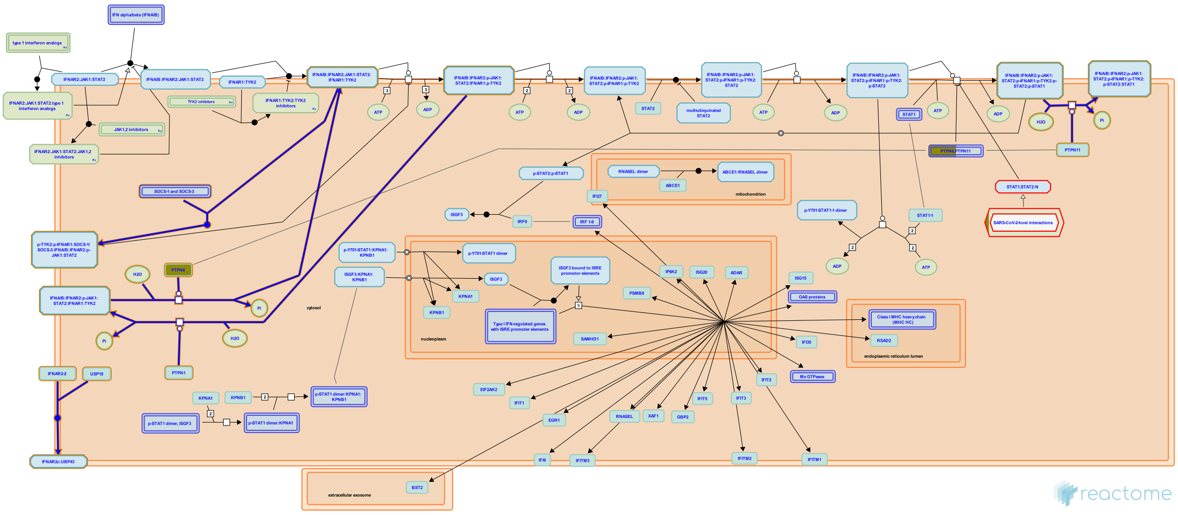

There are several proteins and mechanisms involved in controlling the extent of ligand stimulation of IFNA/B signaling. These mechanisms can effect every step of the IFNA/B cascade. Dephosphorylation of JAK and STAT by SHP protein phosphatases, inhibition of STAT function in the nucleus by protein inhibitors of activated STATs (PIAS) proteins, inhibition of tyrosine kinase activity of JAKs by SOCS as well as inhibition of JAK and IFNAR2 interaction by UBP43 are few of the negative regulation mechanisms in controling type I IFN signaling.

References

Hilton DJ (1999). Negative regulators of cytokine signal transduction. Cell Mol Life Sci, 55, 1568-77.

Fuchs SY, Malakhova OA, Zhang DE, Luo JK, Kim KI, Zou W, ... Kumar KG (2006). UBP43 is a novel regulator of interferon signaling independent of its ISG15 isopeptidase activity. EMBO J, 25, 2358-67.

Alexander WS, Schreiber RD, Sheehan K, Fenner JE, Starr R, Metcalf D, ... Zhang JG (2006). Suppressor of cytokine signaling 1 regulates the immune response to infection by a unique inhibition of type I interferon activity. Nat Immunol, 7, 33-9.

Edit history

Date	Action	Author
2010-07-07	Edited	Garapati P V
2010-07-07	Authored	Garapati P V
2010-07-12	Created	Garapati P V
2010-08-17	Reviewed	Abdul-Sater AA, Schindler C
2023-05-30	Modified	Wright A

1 submitted entities found in this pathway, mapping to 1 Reactome entities

Input	UniProt Id
Ptpn6	P29350

10. Interleukin receptor SHC signaling (R-HSA-912526)

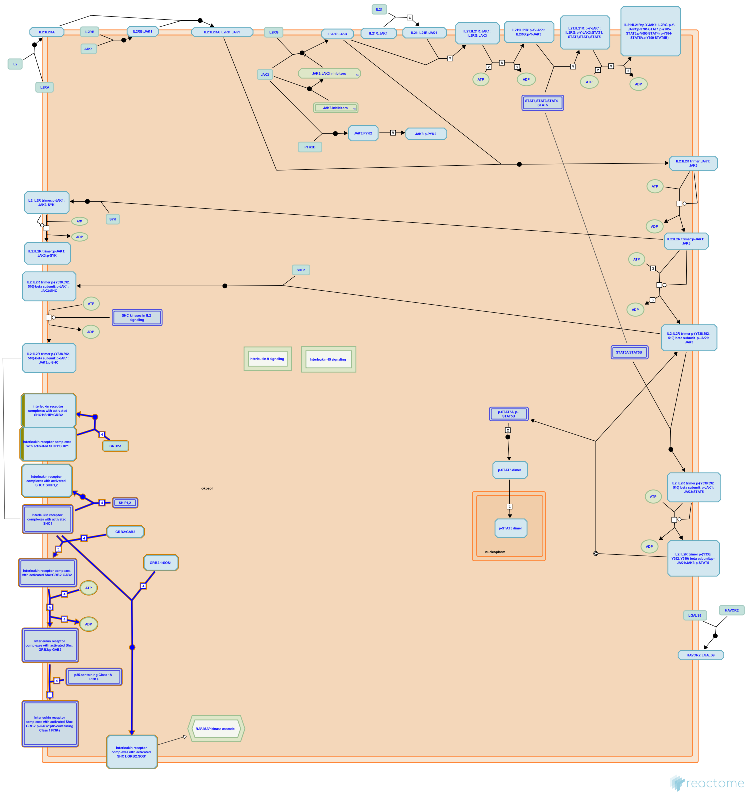

Cellular compartments: plasma membrane.

Phosphorylation of Shc at three tyrosine residues, 239, 240 (Gotoh et al. 1996) and 317 (Salcini et al. 1994) involves unidentified tyrosine kinases presumed to be part of the activated receptor complex. These phosphorylated tyrosines subsequently bind SH2 signaling proteins such as Grb2, Gab2 and SHIP that are involved in the regulation of different signaling pathways. Grb2 can associate with the guanosine diphosphate-guanosine triphosphate exchange factor Sos1, leading to Ras activation and regulation of cell proliferation. Downstream signals are mediated via the Raf-MEK-Erk pathway.Grb2 can also associate through Gab2 with PI3K and with SHIP.

Figure reproduced from Gu, H. et al. 2000. Mol. Cell. Biol. 20(19):7109-7120

Copyright American Society for Microbiology. All Rights Reserved.

References

Ravichandran KS & Burakoff SJ (1994). The adapter protein Shc interacts with the interleukin-2 (IL-2) receptor upon IL-2 stimulation. J Biol Chem, 269, 1599-602.

Edit history

Date	Action	Author
2010-05-17	Authored	Ray KP
2010-07-09	Created	Jupe S
2010-08-06	Edited	Jupe S
2011-02-11	Reviewed	Villarino A
2011-03-17	Reviewed	Dooms H

Date	Action	Author
2023-05-30	Modified	Wright A

1 submitted entities found in this pathway, mapping to 1 Reactome entities

Input	UniProt Id
Ptpn6	P29350

11. Growth hormone receptor signaling (R-HSA-982772)

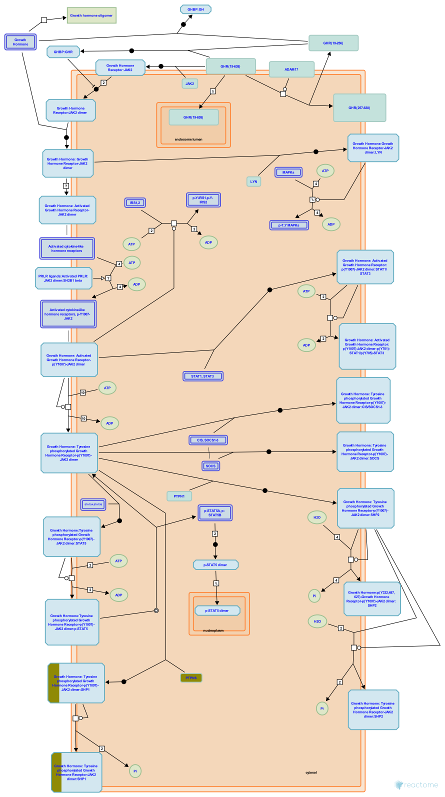

Cellular compartments: cytosol, extracellular region, plasma membrane.

Growth hormone (Somatotropin or GH) is a key factor in determining lean body mass, stimulating the growth and metabolism of muscle, bone and cartilage cells, while reducing body fat. It has many other roles; it acts to regulate cell growth, differentiation, apoptosis, and reorganisation of the cytoskeleton, affecting diverse processes such as cardiac function, immune function, brain function, and aging. GH also has insulin-like effects such as stimulating amino acid transport, protein synthesis, glucose transport, and lipogenesis. The growth hormone receptor (GHR) is a a member of the cytokine receptor family. When the dimeric receptor binds GH it undergoes a conformational change which leads to phosphorylation of key tyrosine residues in its cytoplasmic domains and activation of associated tyrosine kinase JAK2. This leads to recruitment of signaling molecules such as STAT5 and Src family kinases such as Lyn leading to ERK activation. The signal is attenuated by association of Suppressor of Cytokine Signaling (SOCS) proteins and SHP phosphatases which bind to or dephosphorylate specific phosphorylated tyrosines on GHR/JAK. The availability of GHR on the cell surface is regulated by at least two processes; internalization and cleavage from the suface by metalloproteases.

References

Maher SL, d'Aniello EM, Noakes PG, Brown R, Teasdale RD, Waters MJ, ... Lichanska AM (2005). In vivo analysis of growth hormone receptor signaling domains and their associated transcripts. Mol Cell Biol, 25, 66-77.

Fuchs SY, Deng L, Thangavel C, Jiang J, He K, Frank SJ, ... Wang X (2007). Determinants of growth hormone receptor down-regulation. Mol Endocrinol, 21, 1537-51.

Postel-Vinay MC & Finidori J (1995). Growth hormone receptor: structure and signal transduction. Eur J Endocrinol, 133, 654-9.

Waters MJ & Brooks AJ (2010). The growth hormone receptor: mechanism of activation and clinical implications. Nat Rev Endocrinol, 6, 515-25.

Edit history

Date	Action	Author
2010-10-14	Authored	Jupe S
2010-10-28	Created	Jupe S
2011-06-10	Edited	Jupe S
2011-06-13	Reviewed	Herington AC
2011-06-23	Reviewed	Waters MJ
2023-05-30	Modified	Wright A

1 submitted entities found in this pathway, mapping to 1 Reactome entities

Input	UniProt Id
Ptpn6	P29350

12. PD-1 signaling (R-HSA-389948)

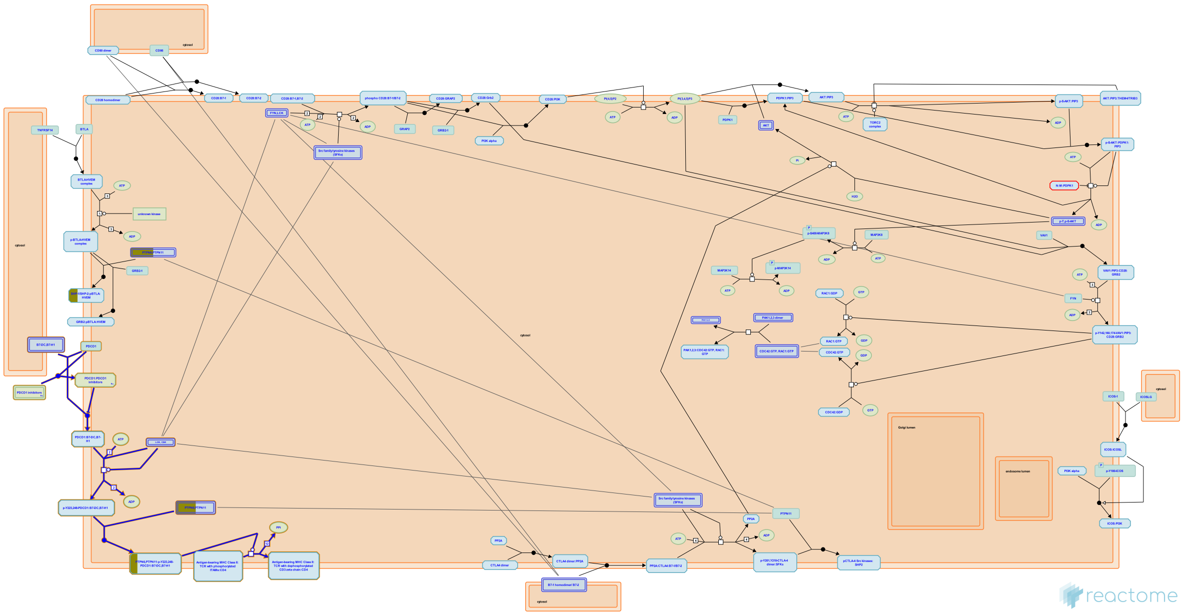

Cellular compartments: plasma membrane.

The Programmed cell death protein 1 (PD-1) is one of the negative regulators of TCR signaling. PD-1 may exert its effects on cell differentiation and survival directly by inhibiting early activation events that are positively regulated by CD28 or indirectly through IL-2. PD-1 ligation inhibits the induction of the cell survival factor Bcl-xL and the expression of transcription factors associated with effector cell function, including GATA-3, Tbet, and Eomes. PD-1 exerts its inhibitory effects by bringing phosphatases SHP-1 and SHP-2 into the immune synapse, leading to dephosphorylation of CD3-zeta chain, PI3K and AKT.

References

Fife BT & Bluestone JA (2008). Control of peripheral T-cell tolerance and autoimmunity via the CTLA-4 and PD-1 pathways. Immunol Rev, 224, 166-82.

Keir ME, Sharpe AH, Freeman GJ & Butte MJ (2008). PD-1 and its ligands in tolerance and immunity . Annu Rev Immunol, 26, 677-704.

Edit history

Date	Action	Author
2008-12-16	Edited	Garapati P V
2008-12-16	Authored	Garapati P V
2009-01-21	Created	Garapati P V
2009-06-01	Reviewed	Bluestone JA, Esensten J
2023-05-21	Modified	Wright A

1 submitted entities found in this pathway, mapping to 1 Reactome entities

Input	UniProt Id
Ptpn6	P29350

13. GPVI-mediated activation cascade (R-HSA-114604)

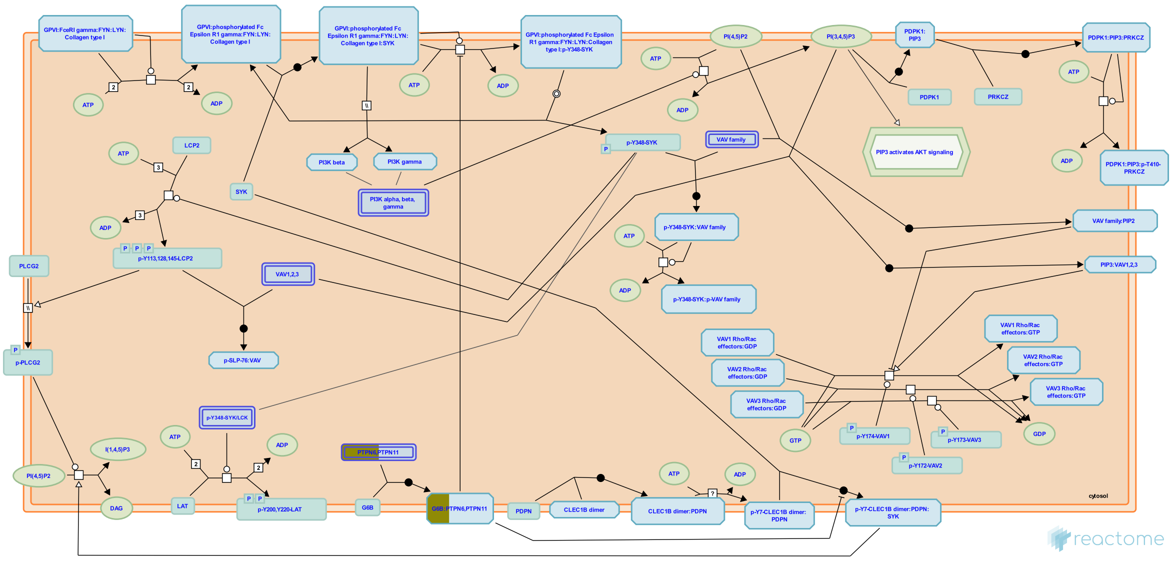

The GPVI receptor is a complex of the GPVI protein with Fc epsilon R1 gamma (FcR). The Src family kinases Fyn and Lyn constitutively associate with the GPVI-FcR complex in platelets and initiate platelet activation through phosphorylation of the immunoreceptor tyrosine-based activation motif (ITAM) in the FcR gamma chain, leading to binding and activation of the tyrosine kinase Syk. Downstream of Syk, a series of adapter molecules and effectors lead to platelet activation.

The GPVI receptor signaling cascade is similar to that of T- and B-cell immune receptors, involving the formation of a signalosome composed of adapter and effector proteins. At the core of the T-cell receptor signalosome is the transmembrane adapter LAT and two cytosolic adapters SLP-76 and Gads. While LAT is essential for signalling to PLCgamma1 downstream of the T-cell receptor, the absence of LAT in platelets only impairs the activation of PLCgamma2, the response to collagen and GPVI receptor ligands remains sufficient to elicit a full aggregation response. In contrast, GPVI signalling is almost entirely abolished in the absence of SLP-76.

References

Suzuki-Inoue K, Moroi M, Bori-Sanz T, Inoue O, Berndt MC, Watson SP, ... Shen Y (2002). Association of Fyn and Lyn with the proline-rich domain of glycoprotein VI regulates intracellular signaling. J Biol Chem, 277, 21561-6.

Auger JM, Watson SP, Pearce AC & McCarty OJ (2005). GPVI and integrin alphaIIb beta3 signaling in platelets. J Thromb Haemost, 3, 1752-62.

Edit history

Date	Action	Author
2004-09-25	Created	Farndale R, Pace NP, de Bono B
2009-11-03	Edited	Jupe S
2017-12-06	Edited	Orlic-Milacic M
2023-05-21	Modified	Wright A

1 submitted entities found in this pathway, mapping to 1 Reactome entities

Input	UniProt Id
Ptpn6	P29350

14. Signaling by ALK (R-HSA-201556)

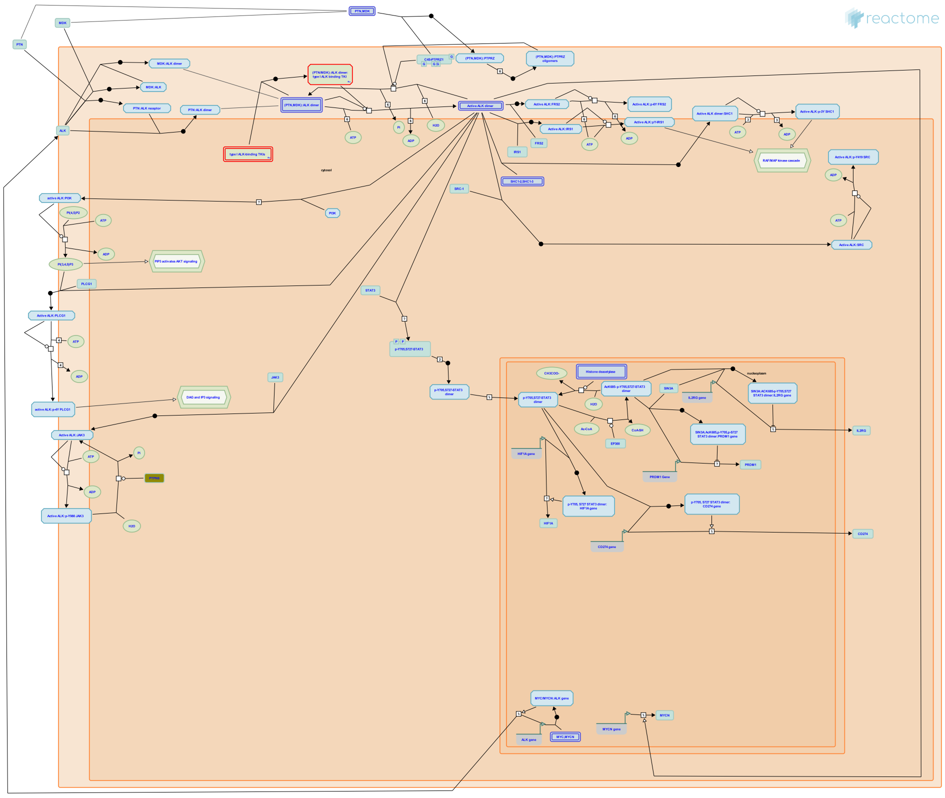

The anaplastic lymphoma kinase (ALK) is a receptor tyrosine kinase that was discovered as an oncogene in anaplastic large cell lymphomas, but also plays an oncogenic role in other cancer types, such as non-small-cell lung cancer (NSCLC), neuroblastoma and glioblastoma. The activation of anaplastic lymphoma kinase (ALK) requires the binding of the ligand pleiotrophin (PTN) or midkine (MDK), which induces the dimerization of the receptor. ALK dimers undergo trans-autophosphorylation, resulting in a fully activated receptor that triggers downstream signaling cascades such as RAS signaling, PI3K signaling and IRS1 signaling. In cancer, ALK gene frequently undergoes translocation, resulting in formation of fusion ALK proteins, such as NPM-ALK and EML4-ALK. These fusion proteins consist of the C-terminal region of ALK, with the kinase domain and the effector protein binding domain, while the N-terminus contains the dimerization domain of the ALK fusion partner. Fusion proteins of ALK are therefore capable of ligand-independent dimerization, resulting in constitutive ALK signaling. ALK can also undergo ligand-independent activation through RPTPB/RPTPZ.

ALK gene is mainly expressed in the developing central and peripheral nervous system. PTN and MDK have neuroprotective effect against neurotoxic agents and in neurodegenerative diseases.

For review, please refer to Chiarle et al. 2008, Wellstein 2012, Deuel 2013, Hallberg and Palmer 2013, Kadomatsu et al. 2013, Winkler and Yao 2014, Herradon and Perez-Garcia 2014, Janoueix-Lerosey et al. 2018, Della Corte et al, 2018.

References

Wellstein A (2012). ALK receptor activation, ligands and therapeutic targeting in glioblastoma and in other cancers. Front Oncol, 2, 192.

Lopez-Delisle L, Delattre O, Rohrer H & Janoueix-Lerosey I (2018). The ALK receptor in sympathetic neuron development and neuroblastoma. Cell Tissue Res., 372, 325-337.

Palmer RH & Hallberg B (2013). Mechanistic insight into ALK receptor tyrosine kinase in human cancer biology. Nat. Rev. Cancer, 13, 685-700.

Herradón G & Pérez-García C (2014). Targeting midkine and pleiotrophin signalling pathways in addiction and neurodegenerative disorders: recent progress and perspectives. Br. J. Pharmacol., 171, 837-48.

Deuel TF (2013). Anaplastic lymphoma kinase: "Ligand Independent Activation" mediated by the PTN/RPTP[illegible]/[illegible] signaling pathway. Biochim. Biophys. Acta, 1834, 2219-23.

Edit history

Date	Action	Author
2007-08-21	Edited	Jassal B
2007-08-21	Authored	Jassal B
2007-08-21	Created	Jassal B
2021-05-04	Reviewed	Inghirami G
2023-05-21	Modified	Wright A

1 submitted entities found in this pathway, mapping to 1 Reactome entities

Input	UniProt Id
Ptpn6	P29350

15. Interleukin-2 family signaling (R-HSA-451927)

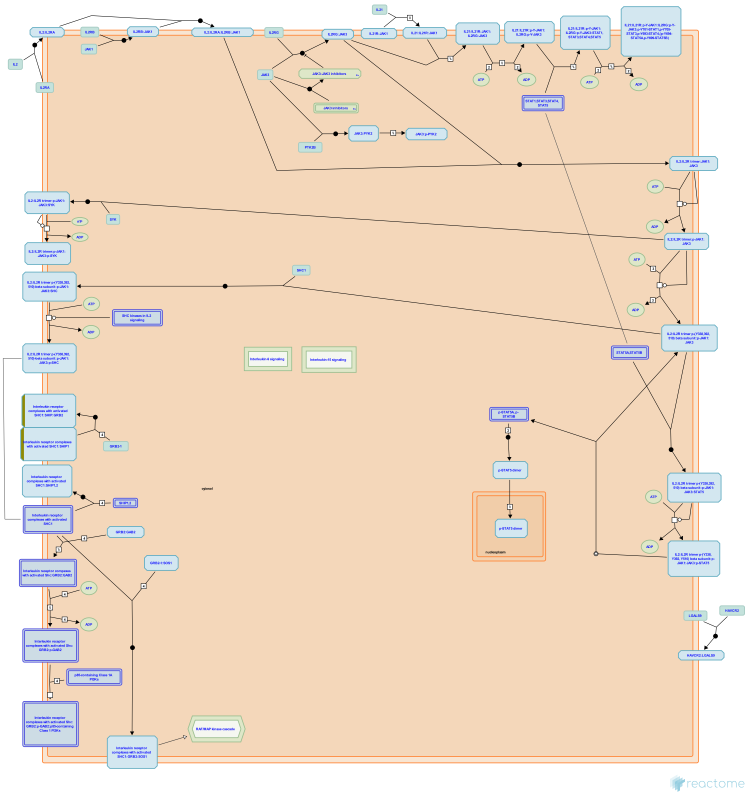

Cellular compartments: plasma membrane.

The interleukin-2 family (also called the common gamma chain cytokine family) consists of interleukin (IL)2, IL9, IL15 and IL21. Although sometimes considered to be within this family, the IL4 and IL7 receptors can form complexes with other receptor chains and are represented separately in Reactome. Receptors of this family associate with JAK1 and JAK3, primarily activating STAT5, although certain family members can also activate STAT1, STAT3 or STAT6.

References

Sim GC & Radvanyi L (2014). The IL-2 cytokine family in cancer immunotherapy. Cytokine Growth Factor Rev., 25, 377-90.

Wang X, Laporte SL, Lupardus P & Garcia KC (2009). Structural biology of shared cytokine receptors. Annu Rev Immunol, 27, 29-60.

Spolski R, Leonard WJ & Rochman Y (2009). New insights into the regulation of T cells by gamma(c) family cytokines. Nat Rev Immunol, 9, 480-90.

Edit history

Date	Action	Author
2010-01-14	Created	Jupe S
2010-05-17	Authored	Ray KP
2010-08-06	Edited	Jupe S
2011-02-11	Reviewed	Villarino A
2011-03-17	Reviewed	Dooms H

Date	Action	Author
2023-05-30	Modified	Wright A

1 submitted entities found in this pathway, mapping to 1 Reactome entities

Input	UniProt Id
Ptpn6	P29350

16. Interleukin-3, Interleukin-5 and GM-CSF signaling (R-HSA-512988)

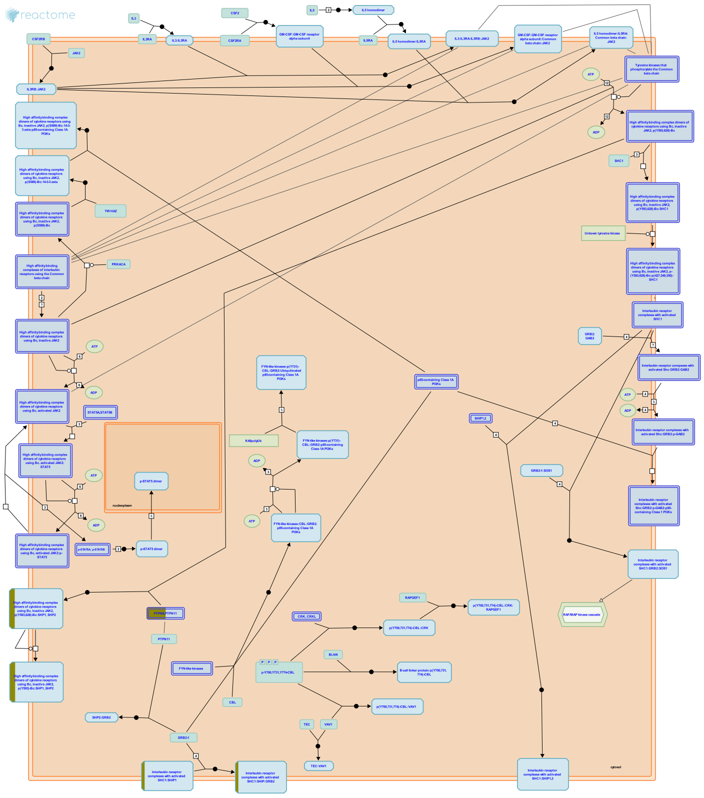

Cellular compartments: plasma membrane.

The Interleukin-3 (IL-3), IL-5 and Granulocyte-macrophage colony stimulating factor (GM-CSF) receptors form a family of heterodimeric receptors that have specific alpha chains but share a common beta subunit, often referred to as the common beta (Bc). Both subunits contain extracellular conserved motifs typical of the cytokine receptor superfamily. The cytoplasmic domains have limited similarity with other cytokine receptors and lack detectable catalytic domains such as tyrosine kinase domains.

IL-3 is a 20-26 kDa product of CD4+ T cells that acts on the most immature marrow progenitors. IL-3 is capable of inducing the growth and differentiation of multi-potential hematopoietic stem cells, neutrophils, eosinophils, megakaryocytes, macrophages, lymphoid and erythroid cells. IL-3 has been used to support the proliferation of murine cell lines with properties of multi-potential progenitors, immature myeloid as well as T and pre-B lymphoid cells (Miyajima et al. 1992). IL-5 is a hematopoietic growth factor responsible for the maturation and differentiation of eosinophils. It was originally defined as a T-cell-derived cytokine that triggers activated B cells for terminal differentiation into antibody-secreting plasma cells. It also promotes the generation of cytotoxic T-cells from thymocytes. IL-5 induces the expression of IL-2 receptors (Kouro & Takatsu 2009). GM-CSF is produced by cells (T-lymphocytes, tissue macrophages, endothelial cells, mast cells) found at sites of inflammatory responses. It stimulates the growth and development of progenitors of granulocytes and macrophages, and the production and maturation of dendritic cells. It stimulates myeloblast and monoblast differentiation, synergises with Epo in the proliferation of erythroid and megakaryocytic progenitor cells, acts as an autocrine mediator of growth for some types of acute myeloid leukemia, is a strong chemoattractant for neutrophils and eosinophils. It enhances the activity of neutrophils and macrophages. Under steady-state conditions GM-CSF is not essential for the production of myeloid cells, but it is required for the proper development of alveolar macrophages, otherwise, pulmonary alvelolar proteinosis (PAP) develops. A growing body of evidence suggests that GM-CSF plays a key role in emergency hematopoiesis (predominantly myelopoiesis) in response to infection, including the production of granulocytes and macrophages in the bone marrow and their maintenance, survival, and functional activation at sites of injury or insult (Hercus et al. 2009).

All three receptors have alpha chains that bind their specific ligands with low affinity (de Groot et al. 1998). Bc then associates with the alpha chain forming a high affinity receptor (Geijsen et al. 2001), though the in vivo receptor is likely be a higher order multimer as recently demonstrated for the GM-CSF receptor (Hansen et al. 2008).

The receptor chains lack intrinsic kinase activity, instead they interact with and activate signaling kinases, notably Janus Kinase 2 (JAK2). These phosphorylate the common beta subunit, allowing recruitment of signaling molecules such as Shc, the phosphatidylinositol 3-kinases (PI3Ks), and the Signal Transducers and Activators of Transcription (STATs). The cytoplasmic domain of Bc has two distinct functional domains: the membrane proximal region mediates the induction of proliferation-associated genes such as c-myc, pim-1 and oncostatin M. This region binds multiple signal-transducing proteins including JAK2 (Quelle et al. 1994), STATs, c-Src and PI3 kinase (Rao and Mufson, 1995). The membrane distal domain is required for cytokine-induced growth inhibition and is necessary for the viability of hematopoietic cells (Inhorn et al. 1995). This region interacts with signal-transducing proteins such as Shc (Inhorn et al. 1995) and SHP and mediates the transcriptional activation of c-fos, c-jun, c-Raf and p70S6K (Reddy et al. 2000).

Figure reproduced by permission from Macmillan Publishers Ltd: Leukemia, WL Blalock et al. 13:1109-1166, copyright 1999. Note that residue numbering in this diagram refers to the mature Common beta chain with signal peptide removed.

References

Bagley CJ, Berndt MC, Stomski FC, Lopez AF, Woodcock JM, Thomas D & Guthridge MA (1998). Mechanism of activation of the GM-CSF, IL-3, and IL-5 family of receptors. Stem Cells, 16, 301-13.

McCubrey JA, Hoyle PE, Steelman LS, Franklin RA, Weinstein-Oppenheimer C, Wang XY, ... Oberhaus SM (1999). Signal transduction, cell cycle regulatory, and anti-apoptotic pathways regulated by IL-3 in hematopoietic cells: possible sites for intervention with anti-neoplastic drugs. Leukemia, 13, 1109-66.

Edit history

Date	Action	Author
2010-02-16	Created	Jupe S
2010-05-17	Authored	Ray KP
2010-08-06	Edited	Jupe S
2010-09-06	Reviewed	Hercus TR, Lopez AF
2023-05-30	Modified	Wright A

1 submitted entities found in this pathway, mapping to 1 Reactome entities

Input	UniProt Id
Ptpn6	P29350

17. Signaling by SCF-KIT (R-HSA-1433557)

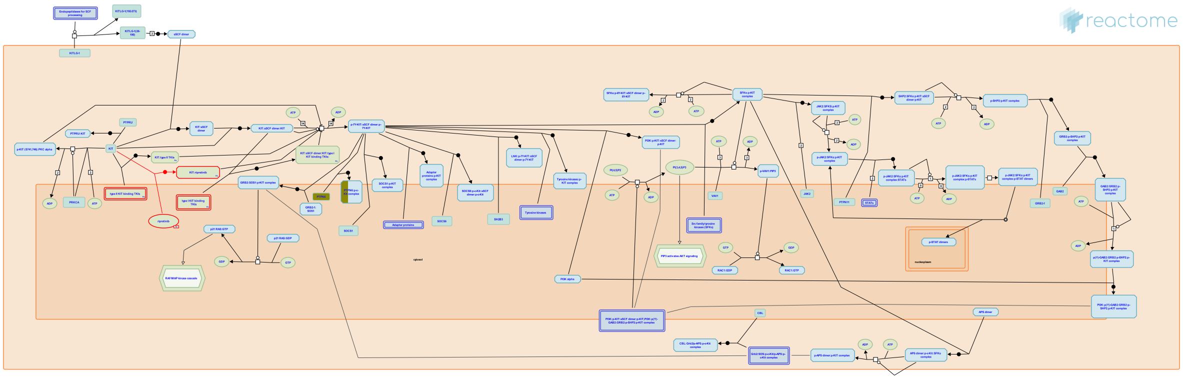

Stem cell factor (SCF) is a growth factor with membrane bound and soluble forms. It is expressed by fibroblasts and endothelial cells throughout the body, promoting proliferation, migration, survival and differentiation of hematopoetic progenitors, melanocytes and germ cells.(Linnekin 1999, Ronnstrand 2004, Lennartsson and Ronnstrand 2006). The receptor for SCF is KIT, a tyrosine kinase receptor (RTK) closely related to the receptors for platelet derived growth factor receptor, colony stimulating factor 1 (Linnekin 1999) and Flt3 (Rosnet et al. 1991). Four isoforms of c-Kit have been identified in humans. Alternative splicing results in isoforms of KIT differing in the presence or absence of four residues (GNNK) in the extracellular region. This occurs due to the use of an alternate 5' splice donor site. These GNNK+ and GNNK- variants are co-expressed in most tissues; the GNNK- form predominates and was more strongly tyrosine-phosphorylated and more rapidly internalized (Ronnstrand 2004). There are also splice variants that arise from alternative usage of splice acceptor site resulting in the presence or absence of a serine residue (Crosier et al., 1993). Finally, there is an alternative shorter transcript of KIT expressed in postmeiotic germ cells in the testis which encodes a truncated KIT consisting only of the second part of the kinase domain and thus lackig the extracellular and transmembrane domains as well as the first part of the kinase domain (Rossi et al. 1991). Binding of SCF homodimers to KIT results in KIT homodimerization followed by activation of its intrinsic tyrosine kinase activity. KIT stimulation activates a wide array of signalling pathways including MAPK, PI3K and JAK/STAT (Reber et al. 2006, Ronnstrand 2004). Defects of KIT in humans are associated with different genetic diseases and also in several types of cancers like mast cell leukaemia, germ cell tumours, certain subtypes of malignant melanoma and gastrointestinal tumours.

References

Lennartsson J & Rönnstrand L (2006). The stem cell factor receptor/c-Kit as a drug target in cancer. Curr Cancer Drug Targets, 6, 65-75.

Frossard N, Da Silva CA & Reber L (2006). Stem cell factor and its receptor c-Kit as targets for inflammatory diseases. Eur J Pharmacol, 533, 327-40.

Rönnstrand L (2004). Signal transduction via the stem cell factor receptor/c-Kit. Cell Mol Life Sci, 61 , 2535-48.

Clark SC, Ricciardi ST, Crosier PS, Vitas MR, Hall LR & Crosier KE (1993). Expression of isoforms of the human receptor tyrosine kinase c-kit in leukemic cell lines and acute myeloid leukemia. Blood, 82, 1151-8.

Hallberg B & Edling CE (2007). c-Kit--a hematopoietic cell essential receptor tyrosine kinase. Int J Biochem Cell Biol, 39, 1995-8.

Edit history

Date	Action	Author
2011-07-11	Edited	Garapati P V
2011-07-11	Authored	Garapati P V
2011-07-11	Created	Garapati P V
2011-08-22	Reviewed	Rönnstrand L
2023-05-21	Modified	Wright A

1 submitted entities found in this pathway, mapping to 1 Reactome entities

Input	UniProt Id
Ptpn6	P29350

18. Cytokine Signaling in Immune system (R-HSA-1280215)

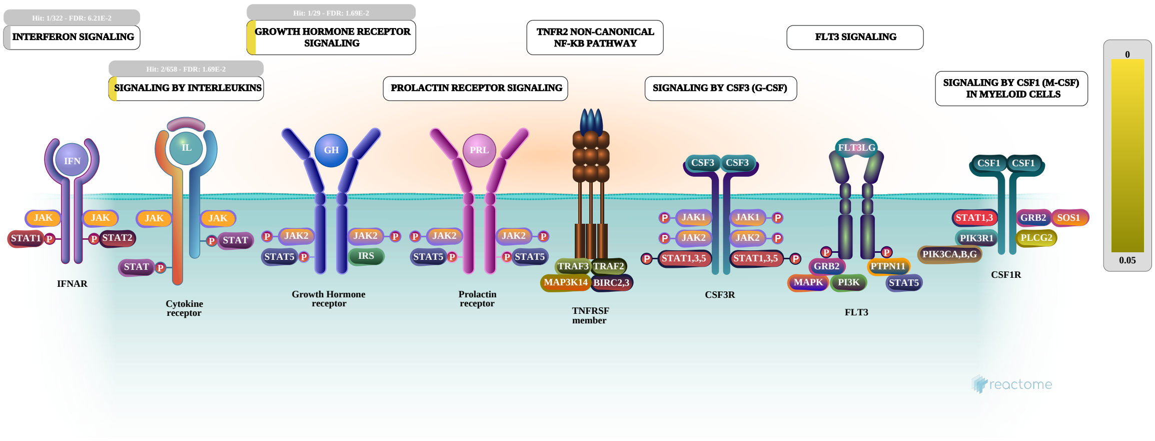

Cytokines are small proteins that regulate and mediate immunity, inflammation, and hematopoiesis. They are secreted in response to immune stimuli, and usually act briefly, locally, at very low concentrations. Cytokines bind to specific membrane receptors, which then signal the cell via second messengers, to regulate cellular activity.

References

Feldmann M & Oppenheim J (2002). *Cytokines and the immune system, Cytokine Reference* .

IMMPORT:Bioinformatics for the future of immunology. Retrieved from https://www.immport.org/immportWeb/queryref/geneListSummary.do

Santamaria P (2003). Cytokines and chemokines in autoimmune disease: an overview. Adv Exp Med Biol, 520, 1-7.

COPE. Retrieved from http://www.copewithcytokines.org/cope.cgi

Edit history

Date	Action	Author
2011-05-12	Created	Garapati P V
2011-05-22	Edited	Ray KP, Jupe S, Garapati P V
2011-05-22	Authored	Ray KP, Jupe S, Garapati P V
2011-05-29	Reviewed	Abdul-Sater AA, Schindler C, Pinteaux E
2023-05-21	Modified	Wright A

1 submitted entities found in this pathway, mapping to 2 Reactome entities

Input	UniProt Id
Ptpn6	P29350

Input	Ensembl Id
Ptpn6	ENSG00000111679

19. CD22 mediated BCR regulation (R-HSA-5690714)

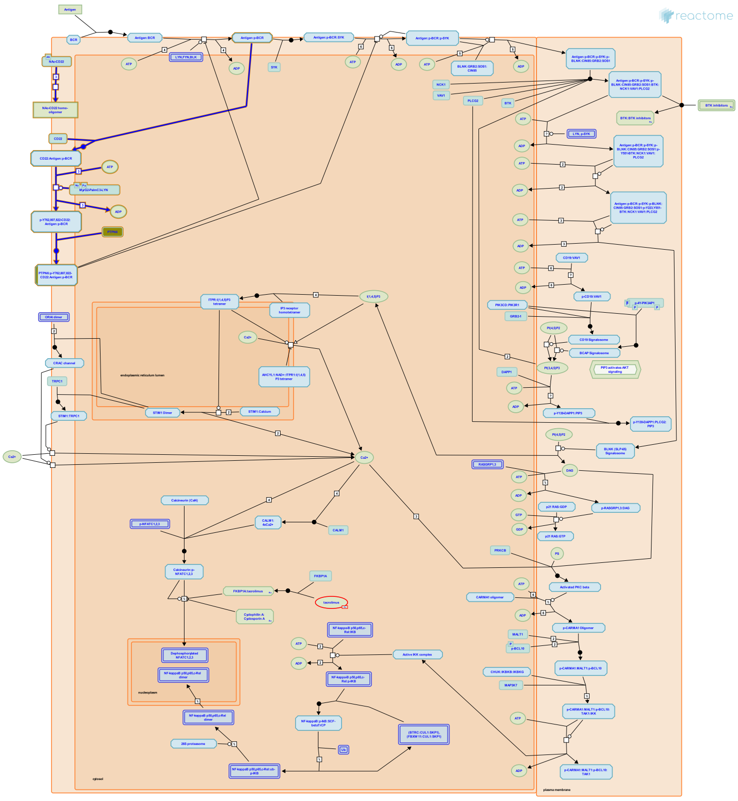

BCR activation is highly regulated and coreceptors like CD22 (SIGLEC2) set a signalling threshold to prevent aberrant immune response and autoimmune disease (Cyster et al. 1997, Han et al. 2005). CD22 is a glycoprotein found on the surface of B cells during restricted stages of development. CD22 is a member of the receptors of the sialic acid-binding Ig-like lectin (Siglec) family which binds specifically to the terminal sequence N-acetylneuraminic acid alpha(2-6) galactose (NeuAc-alpha(2-6)-Gal) present on many B-cell glycoproteins (Powell et al. 1993, Sgroi et al. 1993). CD22 has seven immunoglobulin (Ig)-like extracellular domains and a cytoplasmic tail containing six tyrosines, three of which belong to the inhibitory immunoreceptor tyrosine-based inhibition motifs (ITIMs) sequences. Upon BCR cross-linking CD22 is rapidly tyrosine phosphorylated by the tyrosine kinase Lyn, thereby recruiting and activating tyrosine phosphatase, SHP-1 and inhibiting calcium signalling.

References

Tedder TF, Haas KM, Fujimoto M, Miller AS, Poe JC, Sanford IG, ... Bock CB (2004). CD22 regulates B lymphocyte function in vivo through both ligand-dependent and ligand-independent mechanisms. Nat. Immunol., 5, 1078-87.

Jellusova J & Nitschke L (2011). Regulation of B cell functions by the sialic acid-binding receptors siglec-G and CD22. Front Immunol, 2, 96.

Edit history

Date	Action	Author
2015-04-30	Edited	Garapati P V
2015-04-30	Authored	Garapati P V
2015-04-30	Created	Garapati P V

Date	Action	Author
2015-11-09	Reviewed	Paulson JC
2023-05-21	Modified	Wright A

1 submitted entities found in this pathway, mapping to 1 Reactome entities

Input	UniProt Id
Ptpn6	P29350

20. Costimulation by the CD28 family (R-HSA-388841)

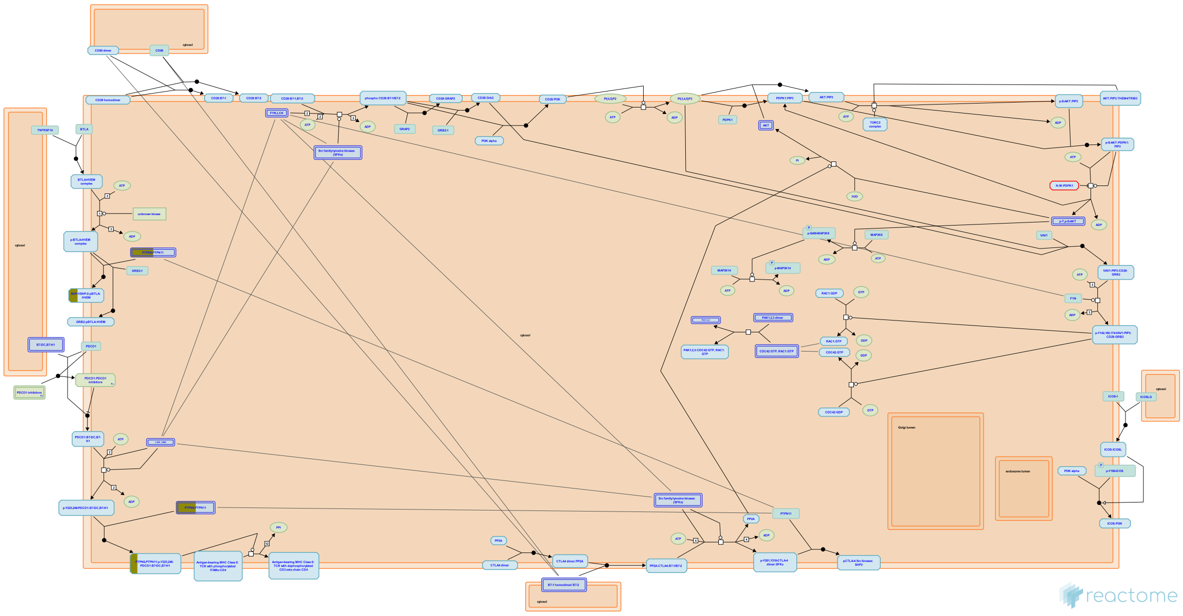

Cellular compartments: plasma membrane.

Optimal activation of T-lymphocytes requires at least two signals. A primary one is delivered by the T-cell receptor (TCR) complex after antigen recognition and additional costimulatory signals are delivered by the engagement of costimulatory receptors such as CD28. The best-characterized costimulatory pathways are mediated by a set of cosignaling molecules belonging to the CD28 superfamily, including CD28, CTLA4, ICOS, PD1 and BTLA receptors. These proteins deliver both positive and negative second signals to T-cells by interacting with B7 family ligands expressed on antigen presenting cells. Different subsets of T-cells have very different requirements for costimulation. CD28 family mediated costimulation is not required for all T-cell responses in vivo, and alternative costimulatory pathways also exist. Different receptors of the CD28 family and their ligands have different regulation of expression. CD28 is constitutively expressed on naive T cells whereas CTLA4 expression is dependent on CD28/B7 engagement and the other receptor members ICOS, PD1 and BTLA are induced after initial T-cell stimulation.

The positive signals induced by CD28 and ICOS molecules are counterbalanced by other members of the CD28 family, including cytotoxic T-lymphocyte associated antigen (CTLA)4, programmed cell death (PD)1, and B and T lymphocyte attenuator (BTLA), which dampen immune responses. The balance of stimulatory and inhibitory signals is crucial to maximize protective immune responses while maintaining immunological tolerance and preventing autoimmunity.

The costimulatory receptors CD28, CTLA4, ICOS and PD1 are composed of single extracellular IgV-like domains, whereas BTLA has one IgC-like domain. Receptors CTLA4, CD28 and ICOS are covalent homodimers, due to an interchain disulphide linkage. The costimulatory ligands B71, B72, B7H2, B7H1 and B7DC, have a membrane proximal IgC-like domain and a membrane distal IgV-like domain that is responsible for receptor binding and dimerization. CD28 and CTLA4 have no known intrinsic enzymatic activity. Instead, engagement by their physiologic ligands B71 and B72 leads to the physical recruitment and activation of downstream T-cell effector molecules.

References

Frauwirth KA, Alegre ML & Thompson CB (2001). T-cell regulation by CD28 and CTLA-4. Nat Rev Immunol, 1, 220-8.

Acuto O & Michel F (2003). CD28-mediated co-stimulation: a quantitative support for TCR signalling. Nat Rev Immunol, 3, 939-51.

Hutchcroft JE, Slavik JM & Bierer BE (1999). CD28/CTLA-4 and CD80/CD86 families: signaling and function. Immunol Res, 19, 1-24.

Edit history

Date	Action	Author
2008-12-16	Edited	Garapati P V
2008-12-16	Authored	Garapati P V
2008-12-16	Created	Garapati P V
2009-06-01	Reviewed	Bluestone JA, Esensten J
2023-05-21	Modified	Wright A

1 submitted entities found in this pathway, mapping to 1 Reactome entities

Input	UniProt Id
Ptpn6	P29350

21. Antigen activates B Cell Receptor (BCR) leading to generation of second messengers (R-HSA-983695)

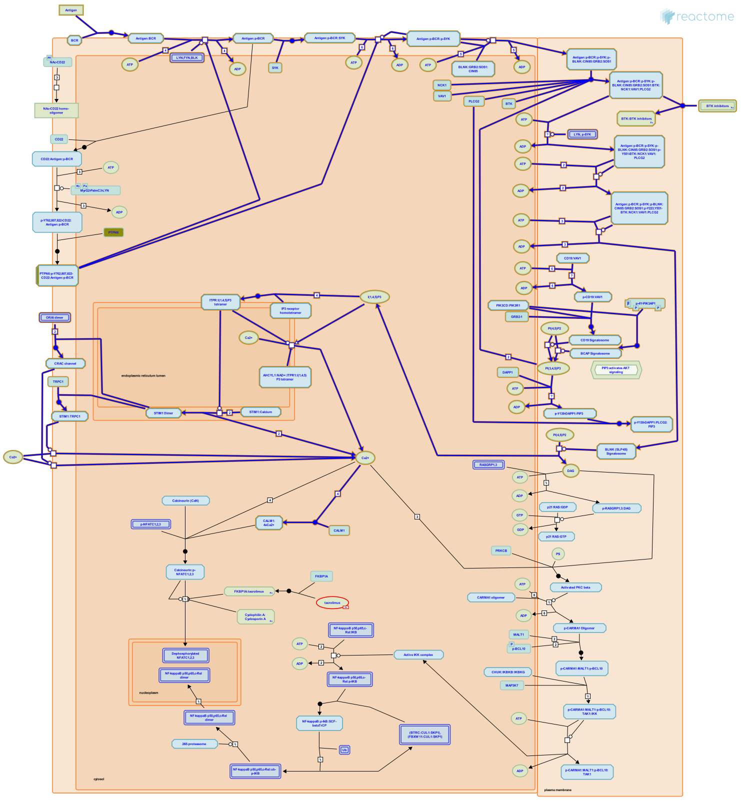

Cellular compartments: cytosol, extracellular region, plasma membrane.

Mature B cells express IgM and IgD immunoglobulins which are complexed with Ig-alpha (CD79A, MB-1) and Ig-beta (CD79B, B29) to form the B cell receptor (BCR) (Fu et al. 1974, Fu et al. 1975, Kunkel et al. 1975, Van Noesal et al. 1992, Sanchez et al. 1993, reviewed in Brezski and Monroe 2008). Binding of antigen to the immunoglobulin activates phosphorylation of immunoreceptor tyrosine-based activation motifs (ITAMs) in the cytoplasmic tails of Ig-alpha and Ig-beta by Src family tyrosine kinases, including LYN, FYN, and BLK (Nel et al. 1984, Yamanashi et al. 1991, Flaswinkel and Reth 1994, Saouaf et al. 1994, Hata et al. 1994, Saouaf et al. 1995, reviewed in Gauld and Cambier 2004, reviewed in Harwood and Batista 2010). The protein kinase SYK may also be involved in phosphorylating the ITAMs.

The protein kinase SYK binds the phosphorylated immunoreceptor tyrosine-activated motifs (ITAMs) on the cytoplasmic tails of Ig-alpha (CD79A, MB-1) and Ig-beta (CD79B, B29) (Wienands et al. 1995, Rowley et al. 1995, Tsang et al. 2008). The binding causes the activation and autophosphorylation of SYK (Law et al. 1994, Irish et al. 2006, Baldock et al. 2008, Tsang et al. 2008, reviewed in Bradshaw 2010).

Activated SYK and other kinases phosphorylate BLNK (SLP-65, BASH) and BCAP. LYN and FYN phosphorylate CD19. Phosphorylated BLNK, BCAP, and CD19 serve as scaffolds which recruit effectors to the plasma membrane and assemble large complexes, the signalosomes. BCAP and CD19 recruit phosphoinositol 3-kinase (PI3K). BLNK recruits phospholipase C gamma (predominantly PLC-gamma2 in B cells, Coggeshall et al. 1992), NCK, BAM32, BTK, VAV1, and SHC. The effectors are phosphorylated by SYK and other kinases.

Phosphorylated BCAP recruits PI3K, which is phosphorylated by a SYK-dependent mechanism (Kuwahara et al. 1996) and produces phosphatidylinositol-3,4,5-trisphosphate (PIP3). Phosphorylated CD19 likewise recruits PIP3K. PIP3 recruits BAM32 (Marshall et al. 2000) and BTK (de Weers et al. 1994, Baba et al. 2001) to the plasma membrane via their PH domains. PIP3 also recruits and activates PLC-gamma1 and PLC-gamma2 (Bae et al. 1998). BTK binds phosphorylated BLNK via its SH2 domain (Baba et al. 2001). BTK phosphorylates PLC-gamma2 (Rodriguez et al. 2001), which activates phospholipase activity (Carter et al. 1991, Roifman and Wang 1992, Kim et al. 2004, Sekiya et al. 2004). Phosphorylated BLNK recruits PLC-gamma, VAV, GRB2, and NCK (Fu and Chan 1997, Fu et al. 1998, Chiu et al. 2002).

PLC-gamma hydrolyzes phosphatidylinositol-4,5-bisphosphate to yield inositol-1,4,5-trisphosphate (IP3) and diacylglycerol (Carter et al. 1991, Kim et al. 2004). IP3 binds receptors on the endoplasmic reticulum and causes release of Ca2+ ions from the ER into the cytosol. The depletion of calcium from the ER in turn activates STIM1 to interact with ORAI and TRPC1 channels (and possibly other TRP channels) in the plasma membrane, resulting in an influx of extracellular calcium ions (Mori et al. 2002, Muik et al. 2008, Luik et al. 2008, Park et al. 2009).

References

Imajoh-Ohmi S, Kawai T, Matsuo Y, Kuwahara K, Hashimoto E, Cooper MD, ... Mitsuyoshi S (1996). Cross-linking of B cell antigen receptor-related structure of pre-B cell lines induces tyrosine phosphorylation of p85 and p110 subunits and activation of phosphatidylinositol 3-kinase. Int Immunol, 8, 1273-85.

Akhlaq M, Graham B, Jones CE, Graff P, Menear K & Baldock D (2000). Purification and characterization of human Syk produced using a baculovirus expression system. Protein Expr Purif, 18, 86-94.

Gruber H, Frischauf I, Romanin C, Derler I, Fahrner M, Madl J, ... Groschner K (2008). Dynamic coupling of the putative coiled-coil domain of ORAI1 with STIM1 mediates ORAI1 channel activation. J Biol Chem, 283, 8014-22.

Reth M & Flaswinkel H (1994). Dual role of the tyrosine activation motif of the Ig-alpha protein during signal transduction via the B cell antigen receptor. EMBO J, 13, 83-9.

Kunkel HG (1975). Surface markers of human lymphocytes. Johns Hopkins Med J, 137, 216-23.

Edit history

Date	Action	Author
2010-09-28	Edited	May B
2010-09-28	Authored	May B
2010-11-02	Created	May B
2012-02-12	Reviewed	Wienands J
2023-05-21	Modified	Wright A

1 submitted entities found in this pathway, mapping to 1 Reactome entities

Input	UniProt Id
Ptpn6	P29350

22. Platelet homeostasis (R-HSA-418346)

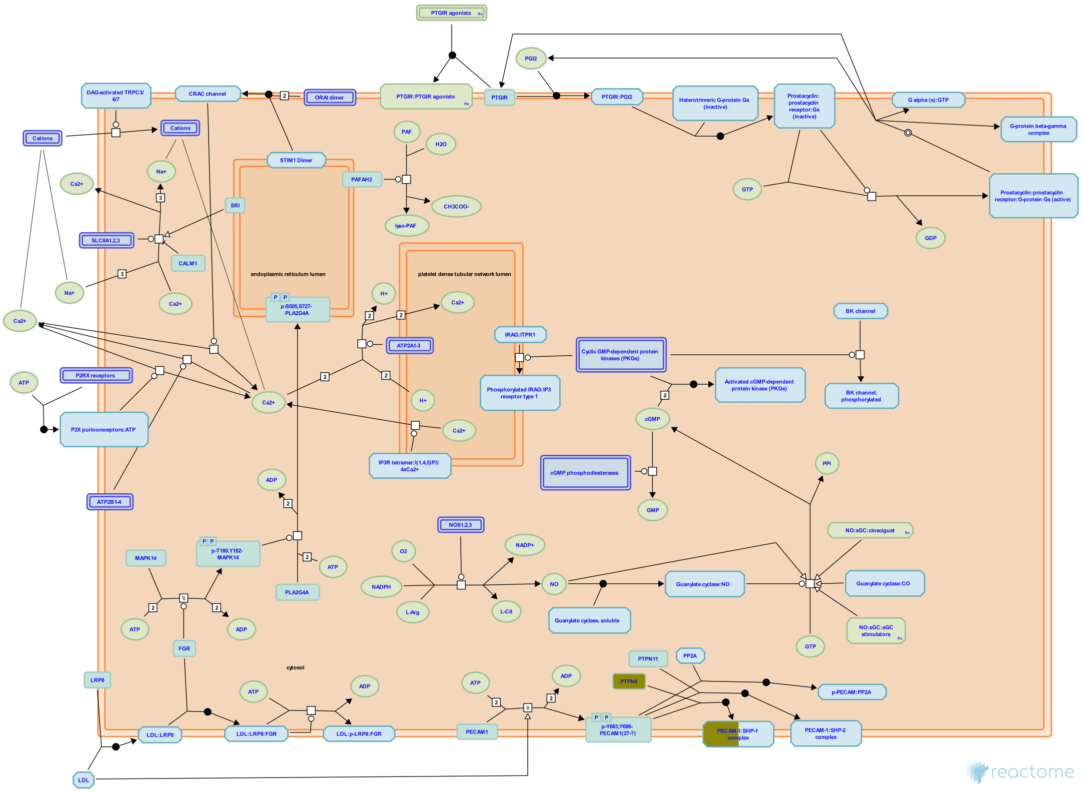

Cellular compartments: plasma membrane.

Under normal conditions the vascular endothelium supports vasodilation, inhibits platelet adhesion and activation, suppresses coagulation, enhances fibrin cleavage and is anti-inflammatory in character. Under acute vascular trauma, vasoconstrictor mechanisms predominate and the endothelium becomes prothrombotic, procoagulatory and proinflammatory in nature. This is achieved by a reduction of endothelial dilating agents: adenosine, NO and prostacyclin; and by the direct action of ADP, serotonin and thromboxane on vascular smooth muscle cells to elicit their contraction (Becker et al. 2000).

Cyclooxygenase-2 (COX-2) and endothelial nitric oxide synthase (eNOS) are primarily expressed in endothelial cells. Both are important regulators of vascular function. Under normal conditions, laminar flow induces vascular endothelial COX-2 expression and synthesis of Prostacyclin (PGI2) which in turn stimulates endothelial Nitric Oxide Synthase (eNOS) activity. PGI2 and NO both oppose platelet activation and aggregation, as does the CD39 ecto-ADPase, which decreases platelet activation and recruitment by metabolizing platelet-released ADP.

References

Heindl B, Becker BF, Kupatt C & Zahler S (2000). Endothelial function and hemostasis. Z Kardiol, 89, 160-7.

Page CP, Vermylen J, Gresele P & Fuster V (2002). *Platelets in thrombotic and non-thrombotic disorders.* , 435-437.

Edit history

Date	Action	Author
2009-04-21	Created	Jupe S
2009-06-03	Authored	Akkerman JW
2010-06-07	Edited	Jupe S
2010-06-07	Reviewed	Kunapuli SP
2023-05-21	Modified	Wright A

1 submitted entities found in this pathway, mapping to 1 Reactome entities

Input	UniProt Id
Ptpn6	P29350

23. Interferon alpha/beta signaling (R-HSA-909733)

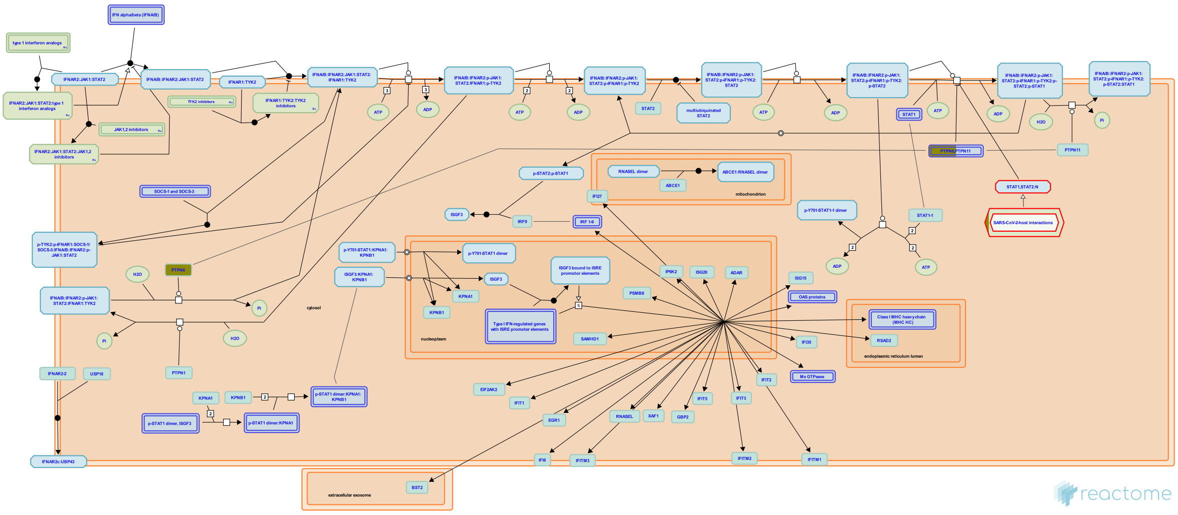

Type I interferons (IFNs) are composed of various genes including IFN alpha (IFNA), beta (IFNB), omega, epsilon, and kappa. In humans the IFNA genes are composed of more than 13 subfamily genes, whereas there is only one IFNB gene. The large family of IFNA/B proteins all bind to a single receptor which is composed of two distinct chains: IFNAR1 and IFNAR2. The IFNA/B stimulation of the IFNA receptor complex leads to the formation of two transcriptional activator complexes: IFNA-activated-factor (AAF), which is a homodimer of STAT1 and IFN-stimulated gene factor 3 (ISGF3), which comprises STAT1, STAT2 and a member of the IRF family, IRF9/P48. AAF mediates activation of the IRF-1 gene by binding to GAS (IFNG-activated site), whereas ISGF3 activates several IFN-inducible genes including IRF3 and IRF7.

References

Stark GR, Darnell JE Jr, Qureshi S, Li X & Leung S (1996). Formation of STAT1-STAT2 heterodimers and their role in the activation of IRF-1 gene transcription by interferon-alpha. J Biol Chem, 271, 5790-4.

Gauzzi MC, Pellegrini S, Velazquez L, McKendry R, Fellous M & Mogensen KE (1996). Interferon-alpha-dependent activation of Tyk2 requires phosphorylation of positive regulatory tyrosines by another kinase. J Biol Chem, 271, 20494-500.

Pellegrini S, Piehler J, Schreiber G & Uzé G (2007). The receptor of the type I interferon family. Curr Top Microbiol Immunol, 316, 71-95.

Gupta S, Greenlund AC, Krolewski JJ, Yan H, Schreiber RD, Schindler CW, ... Krishnan K (1996). Phosphorylated interferon-alpha receptor 1 subunit (IFNaR1) acts as a docking site for the latent form of the 113 kDa STAT2 protein. EMBO J, 15, 1064-74.

Edit history

Date	Action	Author
2010-07-07	Edited	Garapati P V
2010-07-07	Authored	Garapati P V
2010-07-07	Created	Garapati P V
2010-08-17	Reviewed	Abdul-Sater AA, Schindler C
2023-05-30	Modified	Wright A

1 submitted entities found in this pathway, mapping to 1 Reactome entities

Input	UniProt Id
Ptpn6	P29350

24. SARS-CoV-2 activates/modulates innate and adaptive immune responses (R-HSA-9705671)

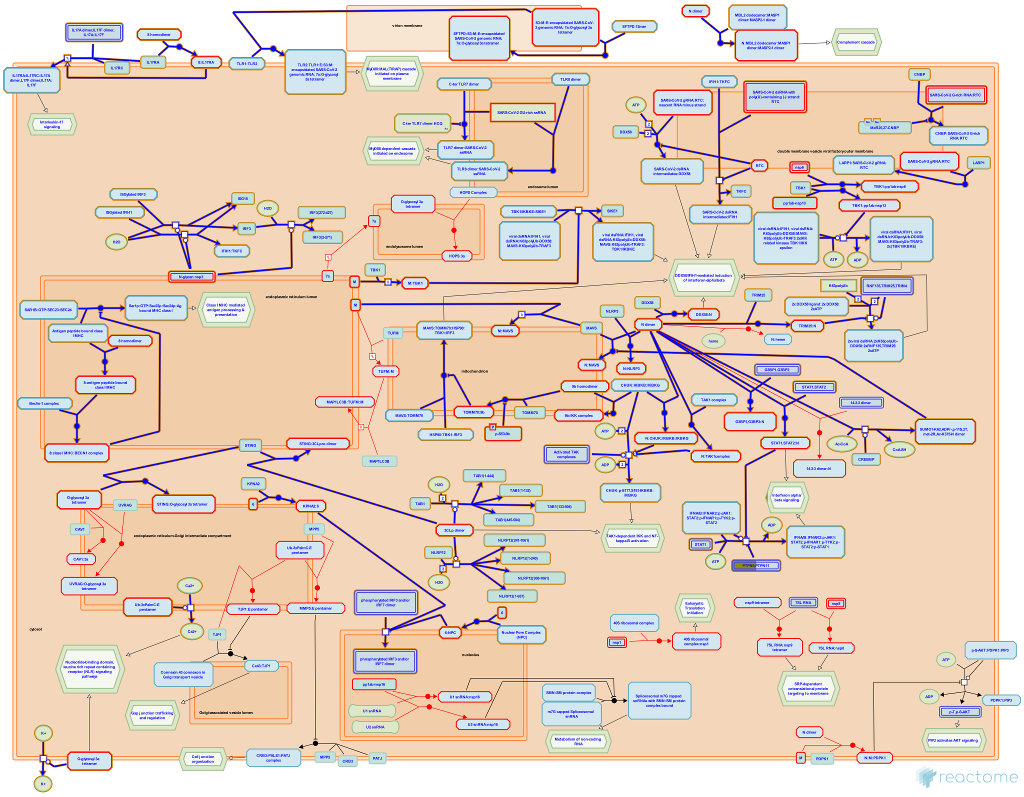

Diseases: COVID-19.

Coronaviruses (CoVs) are positive-sense RNA viruses that replicate in the interior of double membrane vesicles (DMV) in the cytoplasm of infected cells (Stertz S et al. 2007; Knoops K et al. 2008; V'kovski P et al. 2021). The viral replication and transcription are facilitated by virus-encoded non-structural proteins (SARS-CoV-2 nsp1–nsp16) that assemble to form a DMV-bound replication-transcription complex (RTC) (V'kovski P et al. 2021). The replication strategy of CoVs can generate both single-stranded RNA (ssRNA) and double-stranded RNA (dsRNA) species, that may act as pathogen-associated molecular patterns (PAMPs) recognized by pattern recognition receptor (PRR) such as toll-like receptor 7 (TLR7) and TLR8, antiviral innate immune response receptor RIG-I (also known as DEAD box protein 58, DDX58) and interferon-induced helicase C domain-containing protein 1 (IFIH1, also known as MDA5) (Salvi V et al. 2021; Campbell GR et al. 2021; Rebendenne A et al. 2021). The activated PRRs trigger signaling pathways to produce type I and type III interferons IFNs and proinflammatory mediators that perform antiviral functions. This Reactome module describes the mechanisms underlying PRR-mediated sensing of the severe acute respiratory syndrome coronavirus type 2 (SARS-CoV-2) infection. First, endosomal recognition of viral ssRNA occurs by means of TLR7 and TLR8, which detect GU-rich ssRNA sequences (Salvi V et al. 2021; Campbell GR et al. 2021). Second, SARS-CoV-2 dsRNA replication intermediates can be recognized by cytoplasmic receptors DDX58 and IFIH1 which bind to mitochondrial antiviral-signaling protein (MAVS, IPS-1) to induce the IFN-mediated antiviral response (Rebendenne A et al. 2021; Yin X et al. 2021). In addition, SARS-CoV-2 E can be sensed by TLR2 (Zheng M et al. 2021). Further, cellular nucleic acid-binding protein (CNBP) and La-related protein 1 (LARP1) can directly bind SARS-CoV-2 gRNA to repress SARS-CoV-2 replication (Schmidt N et al. 2021). This module also describes several strategies developed by SARS-CoV-2 to evade or alter host immunity, including escaping innate immune

sensors, inhibiting IFN production and signaling, and evading antiviral function of IFN stimulated gene (ISG) products. For example, SARS-CoV-2 encodes nsp14 and nsp16 which possess guanine-N7-methyltransferase activity and 2’-O-methyl-transferase activity respectively (Ogando NS et al. 2020; Krafcikova P et al. 2020; Viswanatha T et al. 2020; Lin S et al. 2021; Yan L et al. 2021). In human coronaviruses nsp14 generates 5' cap-0 viral RNA (m7GpppN, guanine N7-methylated) and nsp16 further methylates cap-0 viral RNA. These viral RNA modifications mimic the 5'-cap structure of host mRNAs allowing the virus to efficiently evade recognition by cytosolic DDX58 and IFIH1 (Chen Y et al. 2009, 2011; Daffis S et al. 2010, shown for CoVs such as SARS-CoV-1 and MERS-CoV). Structural studies and computational analysis suggest that properties and biological functions of SARS-CoV-2 nsp14 and nsp16 could be very similar to these of SARS-CoV-1 (Rosas-Lemus M et al. 2020; Lin S et al. 2020; Viswanathan T et al. 2020; Krafcikova P et al. 2020; Jiang Y et al. 2020; Wilamowski M et al. 2021). Further, the uridylate-specific endoribonuclease (EndoU) activity of SARS-CoV-2 nsp15 degrades viral RNA to hide it from innate immune sensors (Frazier MN et al. 2021). Moreover, SARS-CoV-2 encodes several proteins that directly bind to host targets associated with SARS-CoV-2 infection and cytokine production (Shin D et al. 2020; Viswanathan T et al. 2020; Xia H et al. 2020; Matsuyama T et al. 2020; Yuen CK et al. 2020; reviewed by Park A & Iwasaki A 2020). This Reactome module describes several such binding events and their consequences. For example, as a deubiquitinating and deISGylating enzyme, viral nsp3 binds to and removes ISG15 from signaling proteins such as IRF3 and IFIH1 thereby modulating the formation of signaling complexes and the activation of IRF3/7 and NF-kappaB (Liu CQ et al. 2021). Binding of SARS-CoV-2 nsp6, nsp13 or membrane (M) protein to cytosolic TBK1 prevents IRF3/7 activation and inhibits IFN production downstream of DDX58, IFIH1, MAVS and STING signaling pathways (Xia H et al. 2020; Sui L et al. 2021). Next, M protein targets MAVS to prevent the formation of the MAVS signalosome complex and thereby inhibits downstream signaling pathways of DDX58 and IFIH1 (Fu YZ et al. 2021). Binding of SARS-CoV-2 nucleocapsid (N) protein to E3 ubiquitin ligase TRIM25 inhibits TRIM25-mediated DDX58 ubiquitination and the DDX58 signaling pathway (Gori SG et al. 2021). N interacts with NLRP3 to promote the assembly and activation of the NLRP3 inflammasome (Pan P et al. 2021). The interaction between viral N and MASP2 promotes MASP2-mediated cleavage of C4 (Ali YM et al. 2021) and C2 (Kang S et al. 2021) leading to the hyperactivation of the complement system. Besides, viral N promotes NF-kappaB activation by targeting signaling complexes of TAK1 and IKK (Wu Y et al. 2021). The ion channel activities of accessory protein ORF3a or 3a (open reading frame 3a) and SARS-CoV-2 envelope (E) protein contribute to activation of the NLRP3 inflammasome leading to highly inflammatory pyroptotic cell death (based on findings for SARS-CoV-1, Siu KL et al. 2019). SARS-CoV-2 nsp5 protease (3CLpro) cleaves TAB1, a component of the TAK1 complex, thus inhibiting NF-kappaB activation (Moustaqil M et al .2021). 3CLpro targets NLRP12 which modulates the expression of inflammatory cytokines through the regulation of the NFkappaB and MAPK pathways (Moustaqil M et al. 2021). SARS-CoV-2 6 (ORF6) interacts with importin KPNA2 and components of the nuclear pore complex, NUP98 and RAE1, to block nuclear translocation of IRF3, STAT1 and STAT2 (Xia H et al. 2020; Miorin L et al. 2020). SARS-CoV-2 9b (ORF9b) inhibits the MAVS-mediated production of type I IFNs by targeting TOMM70 on the mitochondria (Jiang HW et al. 2020). Binding of mitochondrial viral 9 to IKBKG prevents MAVS-dependent NF-kappaB activation (Wu J et al. 2021). Although the evasion mechanisms are mainly conserved between SARS-CoV-1 and SARS-CoV-2 (Gordon DE et al. 2020), studies identified SARS-CoV-2-specific modulations of host immune response that may contribute to pathophysiological determinants of COVID-19 (Gordon DE et al. 2020; Schiller HB et al. 2021). This Reactome module describes several virus-host interactions identified in cells during SARS-CoV-2, but not SARS-CoV-1, infection. For example, SARS-CoV-2 8 (ORF8) regulates the expression of class I MHC on the surface of the infected cells through

an autophagy-dependent lysosomal degradation of class I MHC (Zhang Y et al. 2021). At the plasma membrane, binding of secreted viral 8 to IL17RA activates IL17 signaling pathway leading to an increased secretion of cytokines/chemokines thus contributing to cytokine storm during SARS-CoV-2 infection (Lin X et al. 2021). Furthermore, SARS-CoV-2-host interactome and proteomics studies identified various human proteins that are targeted by SARS-CoV-2 proteins (Gordon DE et al. 2020a, b; Bojkova D et al. 2020; Stukalov A et al. 2021; Li J et al. 2021; Messina F et al. 2021). This Reactome module does not cover all identified SARS-CoV-2–human interactions; the module describes those associations that were functionally validated.

References

Bouayad A (2020). Innate immune evasion by SARS-CoV-2: Comparison with SARS-CoV. Rev Med Virol, 30, 1-9.

Sgarbanti M, Hiscott J, Di Carlo D & Palermo E (2021). Type I Interferons in COVID-19 Pathogenesis. Biology (Basel), 10.

Park A & Iwasaki A (2020). Type I and Type III Interferons - Induction, Signaling, Evasion, and Application to Combat COVID-19. Cell Host Microbe, 27, 870-878.

Lowery SA, Perlman S & Sariol A (2021). Innate immune and inflammatory responses to SARS-CoV-2: Implications for COVID-19. Cell Host Microbe, 29, 1052-1062.

Sandini S, Ricci D, Coccia EM, Etna MP, Rizzo F & Severa M (2021). Innate Immune Response to SARS-CoV-2 Infection: From Cells to Soluble Mediators. Int J Mol Sci, 22.

Edit history

Date	Action	Author
2020-10-28	Created	Shamovsky V
2021-05-03	Authored	Stephan R
2021-10-27	Authored	Shamovsky V
2022-02-18	Edited	Shamovsky V
2022-02-18	Reviewed	Messina F
2023-03-08	Modified	Matthews L

1 submitted entities found in this pathway, mapping to 1 Reactome entities

Input	UniProt Id
Ptpn6	P29350

25. Cell-Cell communication (R-HSA-1500931)

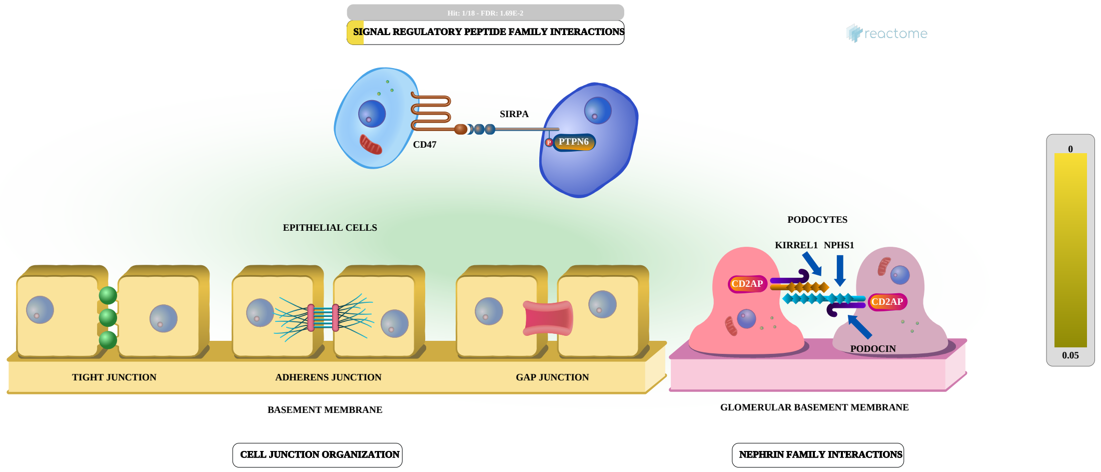

Cell-to-Cell communication is crucial for multicellular organisms because it allows organisms to coordinate the activity of their cells. Some cell-to-cell communication requires direct cell-cell contacts mediated by receptors on their cell surfaces. Members of the immunoglobulin superfamily (IgSF) proteins are some of the cell surface receptors involved in cell-cell recognition, communication and many aspects of the axon guidance and synapse formation-the crucial processes during embryonal development (Rougon & Hobert 2003).

Processes annotated here as aspects of **cell junction organization** mediate the formation and maintenance of adherens junctions, tight junctions, and gap junctions, as well as aspects of cellular interactions with extracellular matrix and hemidesmosome assembly. **Nephrin protein family interactions** are central to the formation of the slit diaphragm, a modified adherens junction. Interactions among members of the **signal regulatory protein family** are important for the regulation of migration and phagocytosis by myeloid cells.

References

Barclay AN & Brown MH (2006). The SIRP family of receptors and immune regulation. Nat Rev Immunol, 6, 457-64.

Lynch RD & Schneeberger EE (1992). Structure, function, and regulation of cellular tight junctions. Am J Physiol, 262, L647-61.

Tryggvason K & Patrakka J (2007). Nephrin--a unique structural and signaling protein of the kidney filter. Trends Mol Med, 13, 396-403.

Kurihara H, Sakai T, Harita Y, Ichimura K & Hattori S (2010). SIRP-alpha-CD47 system functions as an intercellular signal in the renal glomerulus. Am J Physiol Renal Physiol, 299, F517-27.

Rougon G & Hobert O (2003). New insights into the diversity and function of neuronal immunoglobulin superfamily molecules. Annu Rev Neurosci, 26, 207-38.

Edit history

Date	Action	Author
2011-08-23	Edited	Garapati P V
2011-08-23	Authored	Garapati P V
2011-08-23	Created	Matthews L
2023-05-21	Modified	Wright A

1 submitted entities found in this pathway, mapping to 1 Reactome entities

Input	UniProt Id
Ptpn6	P29350

6. Identifiers found

Below is a list of the input identifiers that have been found or mapped to an equivalent element in Reactome, classified by resource.

1 of the submitted entities were found, mapping to 2 Reactome entities

Input	UniProt Id
Ptpn6	P29350

Input	Ensembl Id
Ptpn6	ENSG00000111679

7. Identifiers not found

These 1 identifiers were not found neither mapped to any entity in Reactome.

LOC10524729
