# Supplementary material for: Sex- and region-specific cortical and hippocampal whole genome transcriptome profiles from control and APP/PS1 Alzheimer’s disease mice
Source: PLoS One. 2024 Feb 7;19(2):e0296959. doi: 10.1371/journal.pone.0296959 (PMC10849391; doi:10.1371/journal.pone.0296959)
Supplement: S1 File — S1 Fig: Genotyping of APP/PS1 AD mice and WT control animals. S2 Fig: 3D image of the murine brain including the RS cortex and hippocampus (BROIs) used for transcriptome analysis in our study. S3 Fig: PCA of transcriptomes from the RS cortex and hippocampus of WT controls and APP/PS1 AD mice of both sexes. S4 Fig: Hierarchical clustering of transcriptome data from the RS cortex and hippocampus of WT control and APP/PS1 AD mice of both sexes. S5 Fig: Bar diagrams of the top 30 candidates of DEGs with highest significant FCs (FC > 1.5 and FC < -1.5, p < 0.05). S6 Fig: Pathway analysis of intersectional and signature gene sets in APP/PS1 subgroups. S7 Fig: Comparative qPCR analysis of selected gene transcript levels from the hippocampus of female and male APP/PS1 AD with 5XFAD mice. S1 Table: PCR reaction set-up using PCR Mastermix and genomic DNA. S2 Table: Materials used for one-color microarray-based gene expression data collection. S3 Table: Software used for one-color microarray-based gene expression data collection. S4 Table: Details on genes, forward and reverse primer sequences and annealing temperatures relevant for qPCR experimentation. S5 Table: Characteristics of DEGs in the RS cortex of female APP/PS1 AD mice. S6 Table: Characteristics of DEGs in the hippocampus of female APP/PS1 AD mice. S7 Table: Characteristics of DEGs in the RS cortex of male APP/PS1 AD mice. S8 Table: Characteristics of DEGs in the hippocampus of male APP/PS1 AD mice. S9 Table: Venn analysis of DEGs in the RS cortex and hippocampus of female APP/PS1 AD mice. S10 Table: Venn analysis of DEGs genes in the RS cortex and hippocampus of male APP/PS1 AD mice. S11 Table: Venn analysis of DEGs in the RS cortex of male and female APP/PS1 AD mice. S12 Table: Venn analysis of DEGs in the hippocampus of male and female APP/PS1 AD mice. S13 Table: Differentially regulated l(i)ncRNAs in APP/PS1 AD vs. WT mice. S14 Table: qPCR-based FC analysis of selected genes in the hippocampus of APP/PS1 AD vs. [file pone.0296959.s001.zip › Supplementary Files_R1/Supplementary Figure 6_Pathways_downreg genes/Signature_down_DEGs_male_Hip_APPPS1/Pathway analysis report.pdf]

reactome

# Pathway Analysis Report

This report contains the pathway analysis results for the submitted sample ''. Analysis was performed against Reactome version 85 on 17/08/2023. The web link to these results is:

https://reactome.org/PathwayBrowser/#/ANALYSIS=MjAyMzA4MTcwNzAwMTlfMjEyMDI%3D

Please keep in mind that analysis results are temporarily stored on our server. The storage period depends on usage of the service but is at least 7 days. As a result, please note that this URL is only valid for a limited time period and it might have expired.

# Table of Contents

1. Introduction
2. Properties
3. Genome-wide overview
4. Most significant pathways
5. Pathways details
6. Identifiers found
7. Identifiers not found

# 1. Introduction

Reactome is a curated database of pathways and reactions in human biology. Reactions can be considered as pathway 'steps'. Reactome defines a 'reaction' as any event in biology that changes the state of a biological molecule. Binding, activation, translocation, degradation and classical biochemical events involving a catalyst are all reactions. Information in the database is authored by expert biologists, entered and maintained by Reactome’s team of curators and editorial staff. Reactome content frequently cross-references other resources e.g. NCBI, Ensembl, UniProt, KEGG (Gene and Compound), ChEBI, PubMed and GO. Orthologous reactions inferred from annotation for Homo sapiens are available for 14 non-human species including mouse, rat, chicken, puffer fish, worm, fly and yeast. Pathways are represented by simple diagrams following an SBGN-like format.

Reactome's annotated data describe reactions possible if all annotated proteins and small molecules were present and active simultaneously in a cell. By overlaying an experimental dataset on these annotations, a user can perform a pathway over-representation analysis. By overlaying quantitative expression data or time series, a user can visualize the extent of change in affected pathways and its progression. A binomial test is used to calculate the probability shown for each result, and the p-values are corrected for the multiple testing (Benjamini–Hochberg procedure) that arises from evaluating the submitted list of identifiers against every pathway.

To learn more about our Pathway Analysis, please have a look at our relevant publications:

Fabregat A, Sidiropoulos K, Garapati P, Gillespie M, Hausmann K, Haw R, … D’Eustachio P (2016). The reactome pathway knowledgebase. Nucleic Acids Research, 44(D1), D481–D487. https://doi.org/10.1093/nar/gkv1351.

Fabregat A, Sidiropoulos K, Viteri G, Forner O, Marin-Garcia P, Arnau V, … Hermjakob H (2017). Reactome pathway analysis: a high-performance in-memory approach. BMC Bioinformatics, 18.

## 2. Properties

- This is an **overrepresentation** analysis: A statistical (hypergeometric distribution) test that determines whether certain Reactome pathways are over-represented (enriched) in the submitted data. It answers the question ‘Does my list contain more proteins for pathway X than would be expected by chance?’ This test produces a probability score, which is corrected for false discovery rate using the Benjamani-Hochberg method.
- 4 out of 6 identifiers in the sample were found in Reactome, where 38 pathways were hit by at least one of them.
- All non-human identifiers have been converted to their human equivalent.
- This report is filtered to show only results for species 'Homo sapiens' and resource 'UniProt'.
- The unique ID for this analysis (token) is MjAyMzA4MTcwNzAwMTlfMjEyMDI%3D. This ID is valid for at least 7 days in Reactome's server. Use it to access Reactome services with your data.

# 3. Genome-wide overview

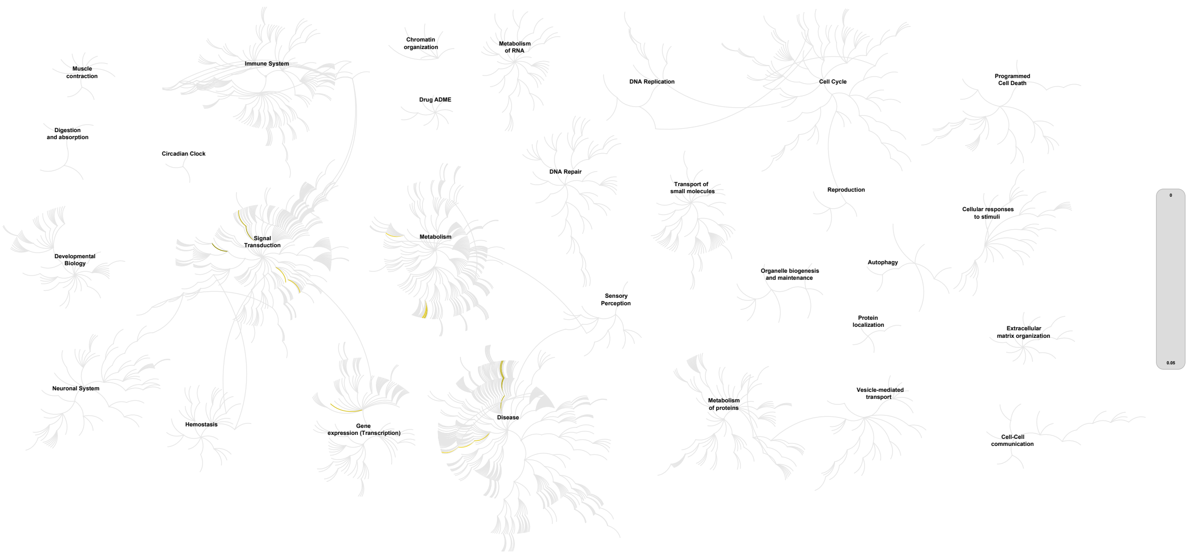


This figure shows a genome-wide overview of the results of your pathway analysis. Reactome pathways are arranged in a hierarchy. The center of each of the circular "bursts" is the root of one top-level pathway, for example "DNA Repair". Each step away from the center represents the next level lower in the pathway hierarchy. The color code denotes over-representation of that pathway in your input dataset. Light grey signifies pathways which are not significantly over-represented.

# 4. Most significant pathways

The following table shows the 25 most relevant pathways sorted by p-value.

| Pathway name | Entities | | | | Reactions | |
|---|---|---|---|---|---|---|
| | found | ratio | p-value | FDR* | found | ratio |
| Defective HLCS causes multiple carboxylase deficiency | 1 / 7 | 6.02e-04 | 0.004 | 0.042 | 4 / 4 | 2.80e-04 |
| Defects in biotin (Btn) metabolism | 1 / 8 | 6.88e-04 | 0.004 | 0.042 | 4 / 6 | 4.19e-04 |
| Hydrolysis of LPC | 1 / 9 | 7.74e-04 | 0.005 | 0.042 | 2 / 6 | 4.19e-04 |
| Biotin transport and metabolism | 1 / 11 | 9.46e-04 | 0.006 | 0.042 | 5 / 13 | 9.09e-04 |
| Negative regulation of TCF-dependent signaling by WNT ligand antagonists | 1 / 15 | 0.001 | 0.008 | 0.042 | 1 / 5 | 3.49e-04 |
| Acyl chain remodelling of PI | 1 / 17 | 0.001 | 0.009 | 0.042 | 1 / 6 | 4.19e-04 |
| Defects in vitamin and cofactor metabolism | 1 / 22 | 0.002 | 0.011 | 0.042 | 4 / 20 | 0.001 |
| Acyl chain remodelling of PS | 1 / 22 | 0.002 | 0.011 | 0.042 | 1 / 8 | 5.59e-04 |
| WNT ligand biogenesis and trafficking | 1 / 26 | 0.002 | 0.013 | 0.042 | 8 / 12 | 8.39e-04 |
| Acyl chain remodelling of PC | 1 / 27 | 0.002 | 0.014 | 0.042 | 1 / 9 | 6.29e-04 |
| Notch-HLH transcription pathway | 1 / 28 | 0.002 | 0.014 | 0.043 | 1 / 2 | 1.40e-04 |
| Acyl chain remodelling of PE | 1 / 29 | 0.002 | 0.015 | 0.045 | 2 / 9 | 6.29e-04 |
| NOTCH1 Intracellular Domain Regulates Transcription | 1 / 49 | 0.004 | 0.025 | 0.049 | 1 / 18 | 0.001 |
| Constitutive Signaling by NOTCH1 PEST Domain Mutants | 1 / 59 | 0.005 | 0.03 | 0.049 | 1 / 21 | 0.001 |
| Constitutive Signaling by NOTCH1 HD+PEST Domain Mutants | 1 / 59 | 0.005 | 0.03 | 0.049 | 1 / 21 | 0.001 |
| Signaling by NOTCH1 HD+PEST Domain Mutants in Cancer | 1 / 59 | 0.005 | 0.03 | 0.049 | 1 / 21 | 0.001 |
| Signaling by NOTCH1 PEST Domain Mutants in Cancer | 1 / 59 | 0.005 | 0.03 | 0.049 | 1 / 21 | 0.001 |
| Signaling by NOTCH1 in Cancer | 1 / 59 | 0.005 | 0.03 | 0.049 | 1 / 39 | 0.003 |
| Signaling by NOTCH1 | 1 / 75 | 0.006 | 0.038 | 0.049 | 1 / 39 | 0.003 |
| Class B/2 (Secretin family receptors) | 1 / 97 | 0.008 | 0.049 | 0.049 | 1 / 24 | 0.002 |
| Metabolism of water-soluble vitamins and cofactors | 1 / 127 | 0.011 | 0.064 | 0.064 | 5 / 143 | 0.01 |
| Glycerophospholipid biosynthesis | 1 / 128 | 0.011 | 0.064 | 0.064 | 7 / 133 | 0.009 |
| Metabolism of vitamins and cofactors | 1 / 192 | 0.017 | 0.095 | 0.095 | 5 / 205 | 0.014 |
| TCF dependent signaling in response to WNT | 1 / 201 | 0.017 | 0.099 | 0.099 | 2 / 71 | 0.005 |

| Pathway name | Entities | | | | Reactions | |
|---|---|---|---|---|---|---|
| | found | ratio | p-value | FDR* | found | ratio |
| Signaling by NOTCH | 1 / 205 | 0.018 | 0.101 | 0.101 | 1 / 154 | 0.011 |

* False Discovery Rate

# 5. Pathways details

For every pathway of the most significant pathways, we present its diagram, as well as a short summary, its bibliography and the list of inputs found in it.

## 1. Defective HLCS causes multiple carboxylase deficiency (R-HSA-3371599)

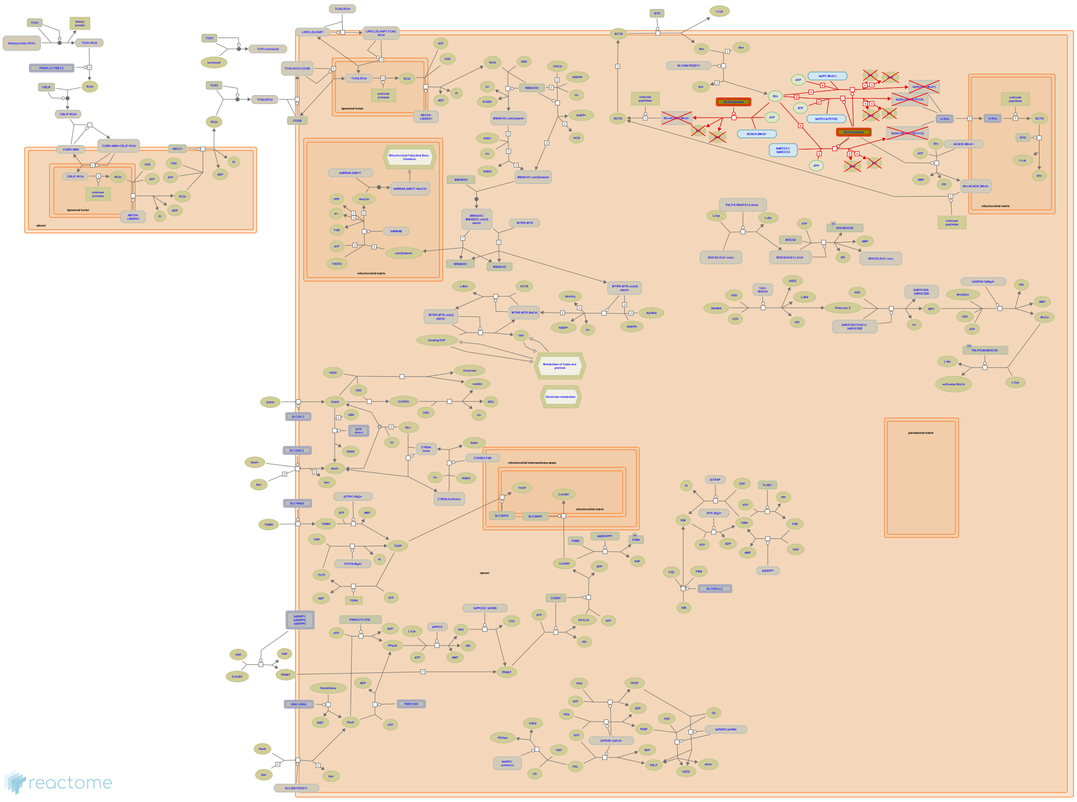

**Diseases:** vitamin metabolic disorder.

Defects in HLCS causes holocarboxylase synthetase deficiency (HLCS deficiency aka early onset multiple carboxylase deficiency; MIM:253270). HLCS deficiency is an autosomal recessive disorder whereby deficient HLCS activity results in reduced activity of all five biotin-dependent carboxylases. Symptoms include metabolic acidosis, organic aciduria, lethargy, hypotonia, convulsions and dermatitis (Suzuki et al. 2005). Patients can present symptoms shortly after birth to up to early childhood and will be prescribed oral biotin supplements, typically 10-20 mg daily. Two classes of HLCS deficiency have been reported depending on whether patients respond to biotin therapy. Most patients respond favourably to treatment and show complete reversal of biochemical and clinical symptoms (Morrone et al. 2002, Dupuis et al. 1999). Here mutations in the HLCS active site cause a reduced affinity for biotin that can be overcome by pharmacological doses of the vitamin (Pendini et al. 2008). Patients who display incomplete responsiveness to biotin therapy have a poor long-term prognosis (Bailey et al. 2008). Here mutations that reside outside of the enzyme's active site have no effect on biotin binding but do compromise the protein-protein interaction between the HLCS and its substrates, resulting in reduced biotinylation of all five carboxylases thus reducing their enzymatic activity (Mayende et al. 2012).

### References

Jitrapakdee S, Wilson CJ, Bailey LM, Wallace JC, Polyak SW & Ivanov RA (2008). Reduced half-life of holocarboxylase synthetase from patients with severe multiple carboxylase deficiency. Hum. Mutat., 29, E47-57.

Mayende L, Booker GW, Bailey LM, Wallace JC, Swift RD, Polyak SW & Soares da Costa TP (2012). A novel molecular mechanism to explain biotin-unresponsive holocarboxylase synthetase deficiency. J. Mol. Med., 90, 81-8.

Suzuki Y, Aoki Y, Kure S, Yang X & Matsubara Y (2005). Mutations in the holocarboxylase synthetase gene HLCS. Hum. Mutat., 26, 285-90.

Pendini NR, Booker GW, Bailey LM, Wallace JC, Polyak SW & Wilce MC (2008). Microbial biotin protein ligases aid in understanding holocarboxylase synthetase deficiency. Biochim. Biophys. Acta, 1784, 973-82.

Pela I, Boneh A, Zammarchi E, Morrone A, Funghini S, Pasquini E, ... Malvagia S (2002). Clinical findings and biochemical and molecular analysis of four patients with holocarboxylase synthetase deficiency. Am. J. Med. Genet., 111, 10-8.

## Edit history

| Date | Action | Author |
|---|---|---|
| 2013-05-13 | Edited | Jassal B |
| 2013-05-13 | Authored | Jassal B |
| 2013-05-13 | Created | Jassal B |
| 2013-08-15 | Reviewed | Polyak SW |
| 2023-03-08 | Modified | Matthews L |

## 1 submitted entities found in this pathway, mapping to 1 Reactome entities

| Input | UniProt Id |
|---|---|
| Hlcs | P50747 |

## 2. Defects in biotin (Btn) metabolism (R-HSA-3323169)

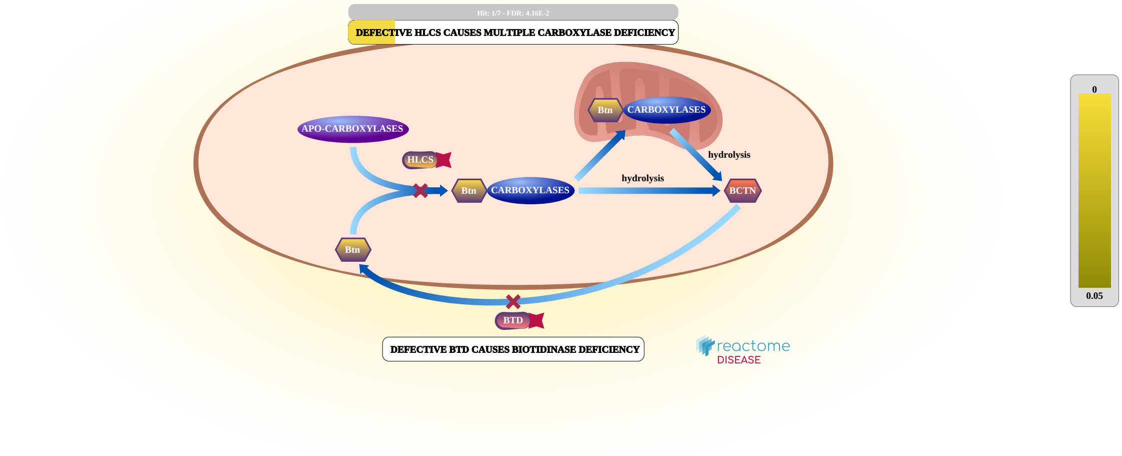


**Diseases:** vitamin metabolic disorder.

Biotin (Btn, vitamin B7, vitamin H, coenzyme R) is an essential cofactor for five biotin-dependent carboxylase enzymes, involved in the synthesis of fatty acids, isoleucine, valine and in gluconeogenesis. Thus, Btn is necessary for cell growth, fatty acid synthesis and the metabolism of fats and amino acids. Inherited metabolic disorders characterized by deficient activities of all five biotin dependent carboxylases are termed multiple carboxylase deficiencies. Two congenital defects in biotin metabolism leading to multiple carboxylase deficiency are known, holocarboxylase synthetase deficiency (MIM 609018) and biotinidase deficiency (MIM 253260). In both scenarios symptoms include ketolactic acidosis, organic aciduria, hyperammonemia, skin rashes, hypotonia, seizures, developmental delay, alopecia, and coma. As humans are auxotrophic for Btn, the micronutrient must be obtained from external soures such as intestinal microflora and dietary forms. Accordingly, severe malnutrition can also give rise to biotin deficiency and multiple carboxylase deficiency. Biotin deficiency can also be induced by the excessive consumption of raw egg white that contains the biotin-binding protein avidin. Holocarboxylase synthetase deficiency arises when all five biotin-dependent enzymes are not biotinylated leading to their reduced activities. The defective genes causing these conditions are described here (Pendini et al. 2008, Suzuki et al. 2005). Biotinidase deficiency is caused by defects in the recycling of Btn. General symptoms include decreased appetite and growth, dermatitis and perosis. The defective genes causing these conditions are described here (Procter et al. 2013).

### References


Wolf B, Crockett DK, Procter M & Mao R (2013). The Biotinidase Gene Variants Registry: A Paradigm Public Database. G3 (Bethesda).

Suzuki Y, Aoki Y, Kure S, Yang X & Matsubara Y (2005). Mutations in the holocarboxylase synthetase gene HLCS. Hum. Mutat., 26, 285-90.

Pendini NR, Booker GW, Bailey LM, Wallace JC, Polyak SW & Wilce MC (2008). Microbial biotin protein ligases aid in understanding holocarboxylase synthetase deficiency. Biochim. Biophys. Acta, 1784, 973-82.

## Edit history

| Date | Action | Author |
| --- | --- | --- |
| 2013-05-09 | Edited | Jassal B |
| 2013-05-09 | Authored | Jassal B |
| 2013-05-09 | Created | Jassal B |
| 2013-08-15 | Reviewed | Polyak SW |
| 2023-03-08 | Modified | Matthews L |

## 1 submitted entities found in this pathway, mapping to 1 Reactome entities

| Input | UniProt Id |
| --- | --- |
| Hlcs | P50747 |

## 3. Hydrolysis of LPC (R-HSA-1483115)

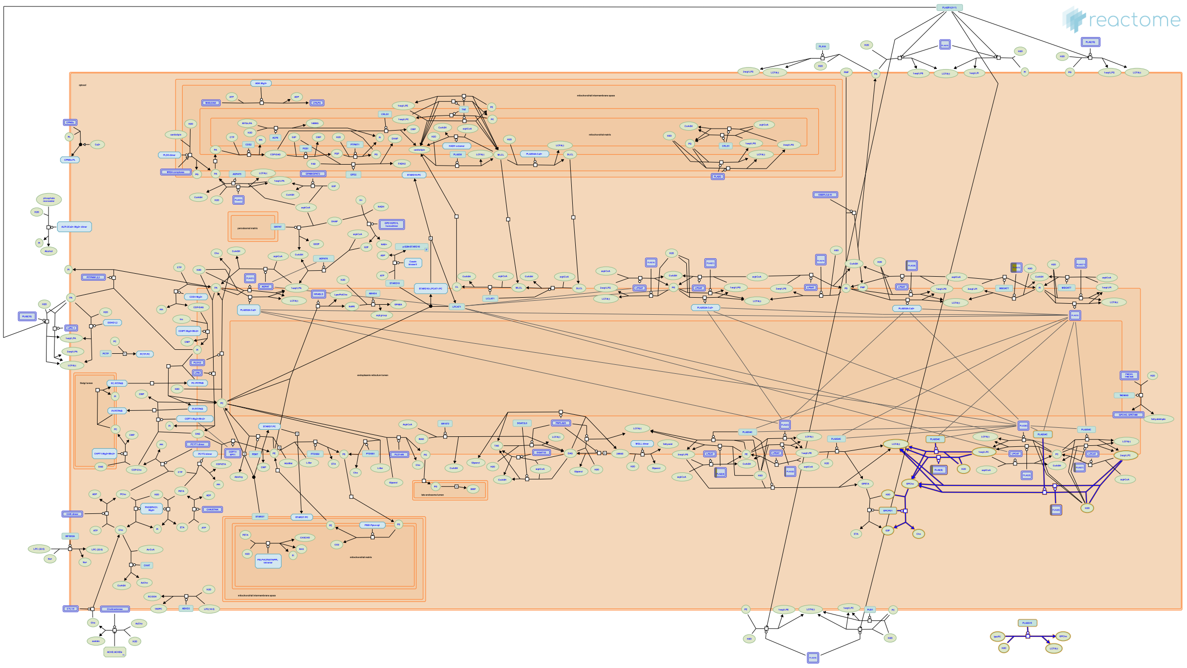

Lysophosphatidylcholine (LPC) is hydrolyzed by phospholipases to produce glycerophosphocholine (GPCho) which is in turn hydrolyzed by glycerophosphocholine phosphodiesterase to produce choline (Cho) and glycerol-3-phosphate (G3P) (Yamashita et al. 2009, Yamashita et al. 2005, Ghomashchi et al. 2010).

### References


Lehr M, Gelb MH, Aloulou A, Naika GS, Bollinger JG, Ghomashchi F & Leslie CC (2010). Interfacial kinetic and binding properties of mammalian group IVB phospholipase A2 (cPLA2beta) and comparison with the other cPLA2 isoforms. J Biol Chem, 285, 36100-11.

Waku K, Nakanishi H, Yamashita A, Kawagishi N, Kamata R, Sugiura T & Suzuki H (2005). Roles of C-terminal processing, and involvement in transacylation reaction of human group IVC phospholipase A2 (cPLA2gamma). J Biochem, 137, 557-67.

Waku K, Tanaka K, Kumazawa T, Yamashita A, Kamata R, Sugiura T, ... Suzuki N (2009). Subcellular localization and lysophospholipase/transacylation activities of human group IVC phospholipase A2 (cPLA2gamma). Biochim Biophys Acta, 1791, 1011-22.


### Edit history

| Date | Action | Author |
|---|---|---|
| 2011-08-12 | Edited | Williams MG |
| 2011-08-12 | Created | Williams MG |
| 2011-09-14 | Authored | Williams MG |
| 2012-05-14 | Reviewed | Wakelam M |
| 2023-05-21 | Modified | Wright A |

### 1 submitted entities found in this pathway, mapping to 1 Reactome entities

| Input | UniProt Id |
| --- | --- |
| Pla2g4e | Q3MJ16 |

## 4. Biotin transport and metabolism (R-HSA-196780)

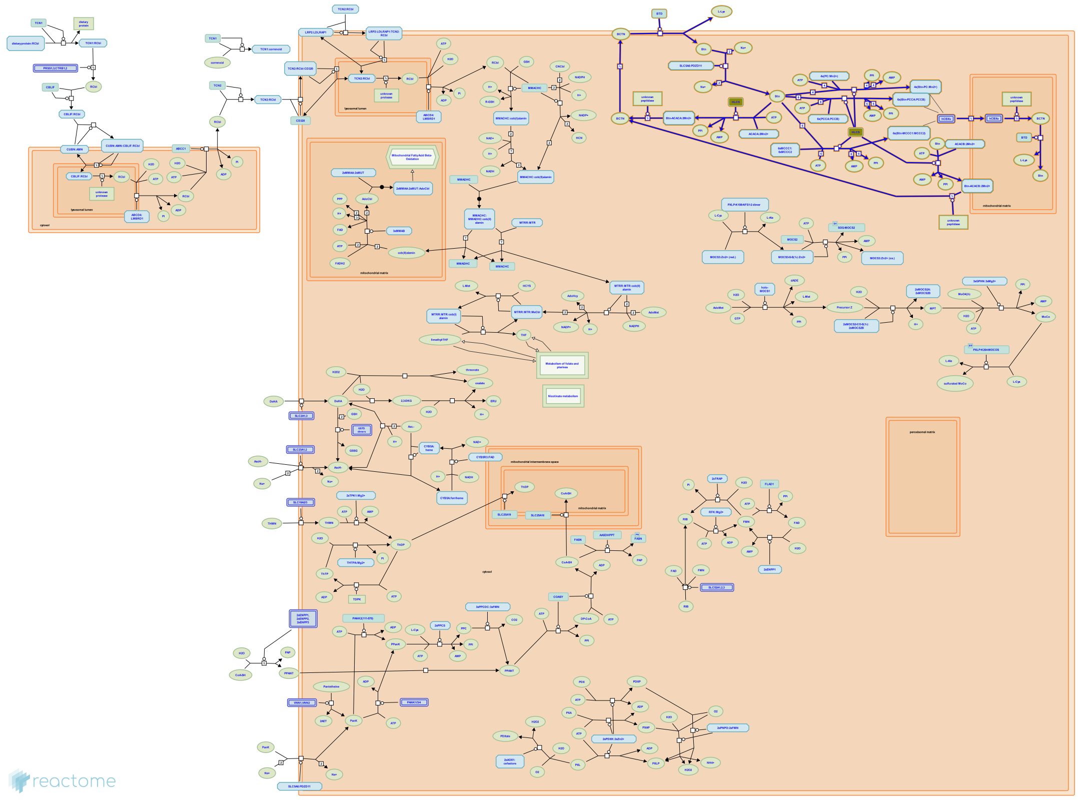

Biotin (Btn) is an essential cofactor in a variety of carboxylation reactions (Zempleni et al. 2009). Humans cannot synthesize Btn but it is abundant in the human diet and can be taken up from the intestinal lumen by the SLC5A6 transporter. Its uptake, intracellular translocation, covalent conjugation to apoenzymes, and salvage are described here.

### References


Wijeratne SS, Hassan YI & Zempleni J (2009). Biotin. Biofactors, 35, 36-46.


### Edit history

| Date | Action | Author |
|---|---|---|
| 2007-04-24 | Edited | Jassal B |
| 2007-04-24 | Authored | Jassal B |
| 2007-04-24 | Created | Jassal B |
| 2013-02-07 | Revised | Jassal B |
| 2014-04-07 | Revised | Jassal B |
| 2023-05-21 | Modified | Wright A |

### 1 submitted entities found in this pathway, mapping to 1 Reactome entities

| Input | UniProt Id |
|---|---|
| Hlcs | P50747 |

## 5. Negative regulation of TCF-dependent signaling by WNT ligand antagonists (R-HSA-3772470)

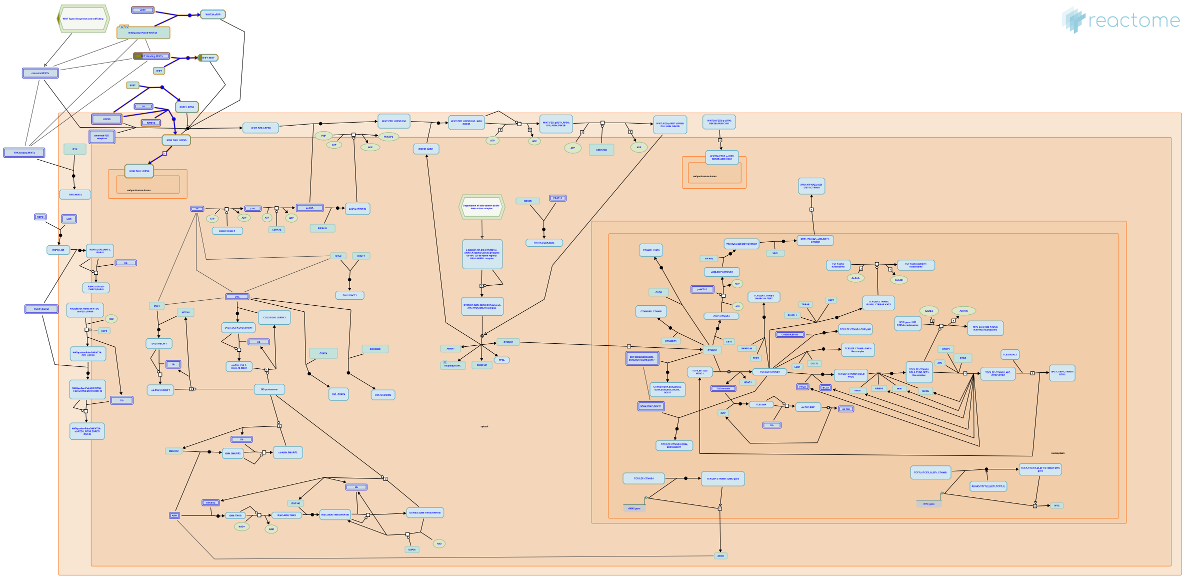

**Cellular compartments:** extracellular region.

Several unrelated families of secreted proteins antagonize WNT signaling. Secreted frizzled-related proteins (sFRPs) have a cysteine rich domain (CRD) that is also found in FZD and ROR receptors, while WNT inhibitory factor (WIF) proteins contain a WIF domain also present in the WNT-receptor RYK. Both these classes of secreted WNT antagonists inhibit signaling by binding to WNTs and preventing their interaction with the FZD receptors. sFRPs may also able to bind the receptors, blocking ligand binding (Bafico et al, 1999; reviewed in Kawano and Kypta, 2003). The interaction of WIF and sFRPs with WNT ligand may also play a role in regulating WNT diffusion and gradient formation (reviewed in Boloventa et al, 2008).

Dickkopf (DKK) and Sclerostin (SOST) family members, in contrast, antagonize WNT signaling by binding to LRP5/6. There are four DKK family members in vertebrates; the closely related DKK1, 2 and 4 proteins have been shown to have roles in WNT signaling, while the more divergent DKK3 appears not to (Glinka et al, 1998; Fedi et al, 1999; Mao et al, 2001; Semenov et al, 2001; reviewed in Niehrs, 2006). Secreted DKK proteins bind to LRP6 in conjunction with the single-pass transmembrane proteins Kremen 1 and 2, and this interaction is thought to disrupt the WNT-induced FZD-LRP5/6 complex. In some cases, DKK2 has also been shown to function as a WNT agonist (reviewed in N (reviewed in Niehrs, 2006).

Like DKK proteins, SOST binds LRP5/6 and disrupts WNT-dependent receptor activation (Semenov et al, 2005).

### References


Miki T, Fedi P, Kraus MH, Burgess WH, Aaronson SA, Bottaro DP, ... Bafico A (1999). Isolation and biochemical characterization of the human Dkk-1 homologue, a novel inhibitor of mammalian Wnt signaling. J. Biol. Chem., 274, 19465-72.

Lopez-Rios J, Bovolenta P, Cisneros E, Esteve P & Ruiz JM (2008). Beyond Wnt inhibition: new functions of secreted Frizzled-related proteins in development and disease. J. Cell. Sci., 121, 737-46.

Kawano Y & Kypta R (2003). Secreted antagonists of the Wnt signalling pathway. J. Cell. Sci., 116, 2627-34.

Wu W, Niehrs C, Stannek P, Mao B, Li Y, Hoppe D & Glinka A (2001). LDL-receptor-related protein 6 is a receptor for Dickkopf proteins. Nature, 411, 321-5.

He X, Tamai K & Semënov M (2005). SOST is a ligand for LRP5/LRP6 and a Wnt signaling inhibitor. J. Biol. Chem., 280, 26770-5.

## Edit history

| Date | Action | Author |
|---|---|---|
| 2007-09-04 | Edited | Matthews L |
| 2013-05-30 | Authored | Rothfels K |
| 2013-06-25 | Created | Rothfels K |
| 2013-10-03 | Edited | Gillespie ME |
| 2014-01-22 | Reviewed | Rajakulendran N |
| 2014-02-15 | Reviewed | van Amerongen R |
| 2014-04-22 | Reviewed | Kikuchi A |
| 2023-05-21 | Modified | Wright A |

## 1 submitted entities found in this pathway, mapping to 1 Reactome entities

| Input | UniProt Id |
|---|---|
| Wnt9a | O14904 |

## 6. Acyl chain remodelling of PI (R-HSA-1482922)

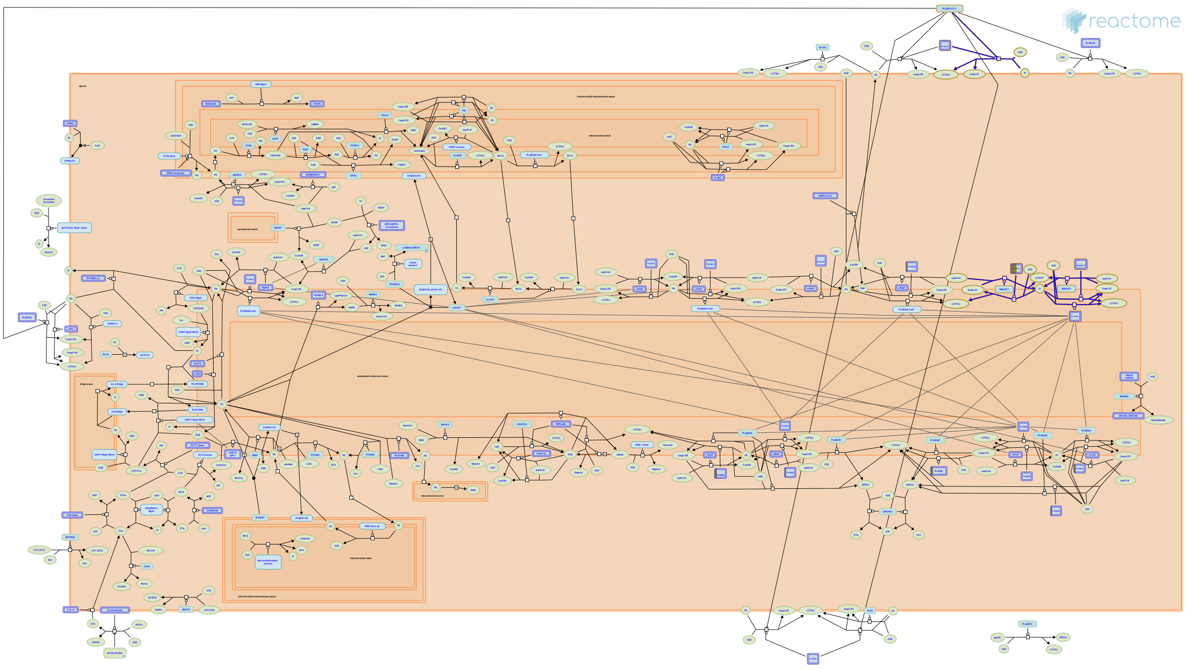

In the acyl chain remodelling pathway (Lands cycle), phosphatidylinositol (PI) is hydrolyzed by phospholipases and subsequently reacylated by acyltransferases. These cycles modify the fatty acid composition of glycerophospholipids to generate diverse molecules asymmetrically distributed in the cell membrane (Ghomashchi et al. 2010, Singer et al. 2002, Gijon et al. 2008, Lee et al. 2008).

### References


Zarini S, Voelker DR, Riekhof WR, Gijon MA & Murphy RC (2008). Lysophospholipid acyltransferases and arachidonate recycling in human neutrophils. J Biol Chem, 283, 30235-45.

Lehr M, Gelb MH, Aloulou A, Naika GS, Bollinger JG, Ghomashchi F & Leslie CC (2010). Interfacial kinetic and binding properties of mammalian group IVB phospholipase A2 (cPLA2beta) and comparison with the other cPLA2 isoforms. J Biol Chem, 285, 36100-11.

Kono N, Matsuda S, Mitani S, Arai H, Gengyo-Ando K, Inoue T, ... Shirae S (2008). Caenorhabditis elegans mboa-7, a member of the MBOAT family, is required for selective incorporation of polyunsaturated fatty acids into phosphatidylinositol. Mol Biol Cell, 19, 1174-84.

Gelb MH, Le Calvez C, Ghomashchi F, Rouault M, Singer AG, Sadilek M, ... Nguyen E (2002). Interfacial kinetic and binding properties of the complete set of human and mouse groups I, II, V, X, and XII secreted phospholipases A2. J Biol Chem, 277, 48535-49.


### Edit history

| Date | Action | Author |
|---|---|---|
| 2011-08-12 | Edited | Williams MG |
| 2011-08-12 | Created | Williams MG |
| 2011-09-14 | Authored | Williams MG |
| 2012-05-14 | Reviewed | Wakelam M |
| 2023-05-21 | Modified | Wright A |

## 1 submitted entities found in this pathway, mapping to 1 Reactome entities

| Input | UniProt Id |
| --- | --- |
| Pla2g4e | Q3MJ16 |

# 7. Defects in vitamin and cofactor metabolism (R-HSA-3296482)

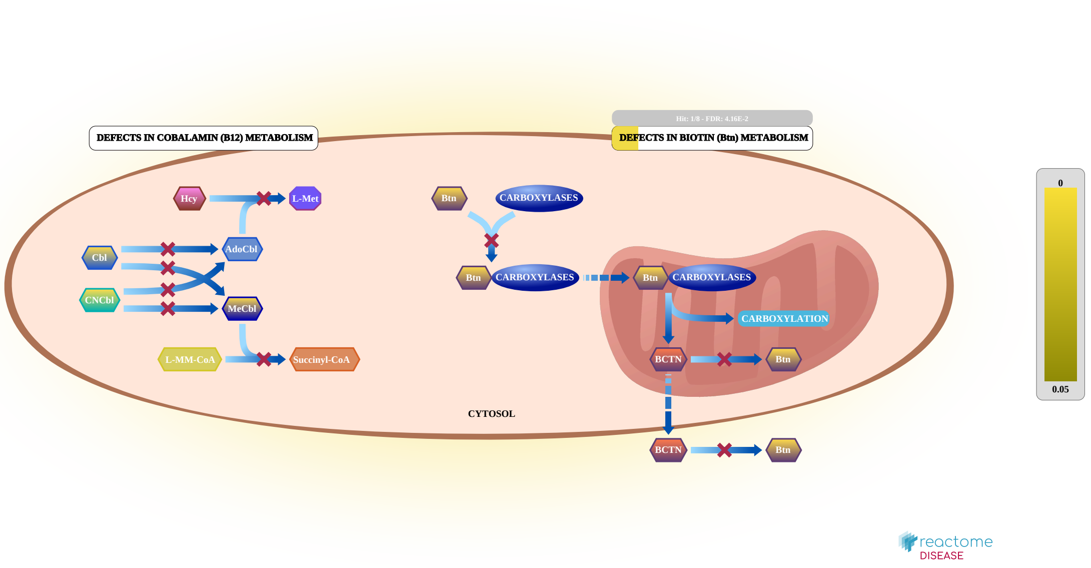


**Diseases:** vitamin metabolic disorder.

Vitamins are essential nutrients, required in small amounts from the diet for the normal growth and development of a multicellular organism. Where there is vitamin deficiency, either by poor diet or a defect in metabolic conversion, diseases called Avitaminoses occur. Currently, cobalamin (Cbl, vitamin B12) metabolic defects are described below (Chapter 155 in The Metabolic and Molecular Bases of Inherited Disease, 8th ed, Scriver et al. 2001)

## References

Beaudet AL, Antonarakis SE, Ballabio A, Kinzler KW, Valle D & Vogelstein B (2001). *Chapter 155: Inherited Disorders of Folate and Cobalamin Transport and Metabolism, The Online Metabolic and Molecular Bases of Inherited Disease* .

Wijeratne SS, Hassan YI & Zempleni J (2008). Biotin and biotinidase deficiency. Expert Rev Endocrinol Metab, 3, 715-724.

## Edit history

| Date | Action | Author |
|---|---|---|
| 2013-04-18 | Edited | Jassal B |
| 2013-04-18 | Authored | Jassal B |
| 2013-04-18 | Created | Jassal B |
| 2013-08-14 | Reviewed | Watkins D |
| 2023-03-08 | Modified | Matthews L |

## 1 submitted entities found in this pathway, mapping to 1 Reactome entities

| Input | UniProt Id |
|---|---|
| Hlcs | P50747 |

## 8. Acyl chain remodelling of PS (R-HSA-1482801)

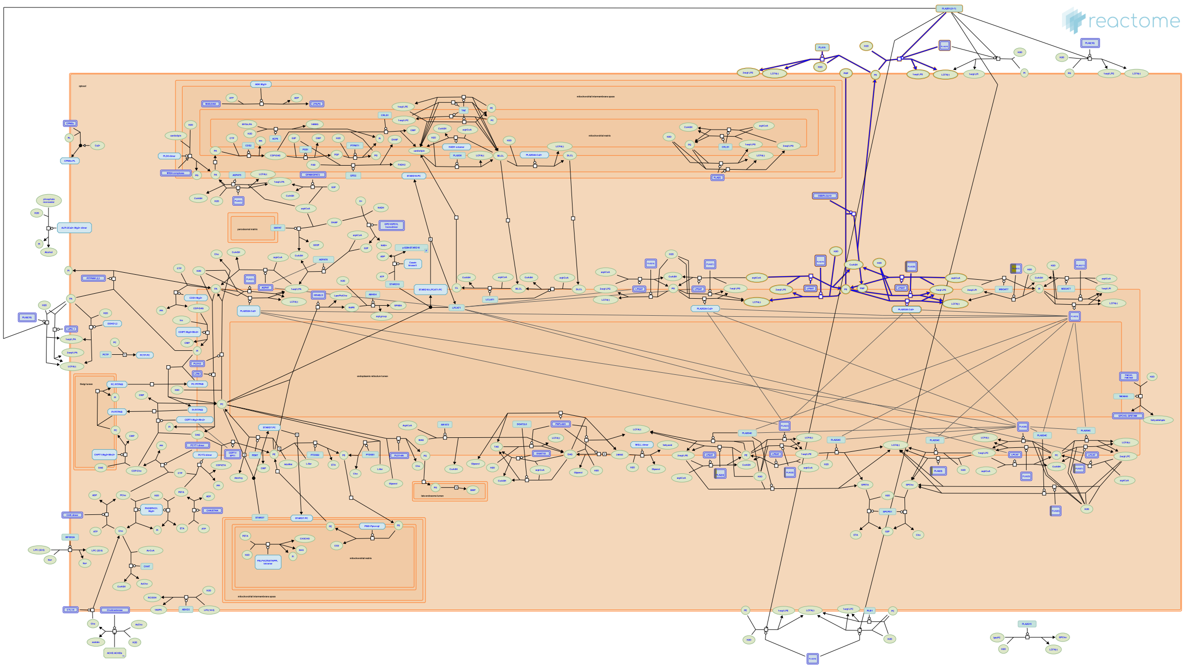

In the acyl chain remodelling pathway (Lands cycle), phosphatidylserine (PS) is hydrolysed by phospholipases and subsequently reacylated by acyltransferases. These cycles modify the fatty acid composition of glycerophospholipids to generate diverse molecules asymmetrically distributed in the cell membrane (Ghomashchi et al. 2010, Singer et al. 2002, Cao et al. 2008; Hishikawa et al. 2008).

### References


Lehr M, Gelb MH, Aloulou A, Naika GS, Bollinger JG, Ghomashchi F & Leslie CC (2010). Interfacial kinetic and binding properties of mammalian group IVB phospholipase A2 (cPLA2beta) and comparison with the other cPLA2 isoforms. J Biol Chem, 285, 36100-11.

Revett T, Tobin JF, Cao J, Li D, Shan D, Gimeno RE, ... Liu W (2008). Molecular identification of a novel mammalian brain isoform of acyl-CoA:lysophospholipid acyltransferase with prominent ethanolamine lysophospholipid acylating activity, LPEAT2. J Biol Chem, 283, 19049-57.

Gelb MH, Le Calvez C, Ghomashchi F, Rouault M, Singer AG, Sadilek M, ... Nguyen E (2002). Interfacial kinetic and binding properties of the complete set of human and mouse groups I, II, V, X, and XII secreted phospholipases A2. J Biol Chem, 277, 48535-49.

Shindou H, Nakanishi H, Taguchi R, Kobayashi S, Shimizu T & Hishikawa D (2008). Discovery of a lysophospholipid acyltransferase family essential for membrane asymmetry and diversity. Proc Natl Acad Sci U S A, 105, 2830-5.


### Edit history

| Date | Action | Author |
|---|---|---|
| 2011-08-12 | Edited | Williams MG |
| 2011-08-12 | Created | Williams MG |
| 2011-09-14 | Authored | Williams MG |
| 2023-05-21 | Modified | Wright A |

## 1 submitted entities found in this pathway, mapping to 1 Reactome entities

| Input | UniProt Id |
| --- | --- |
| Pla2g4e | Q3MJ16 |

## 9. WNT ligand biogenesis and trafficking (R-HSA-3238698)

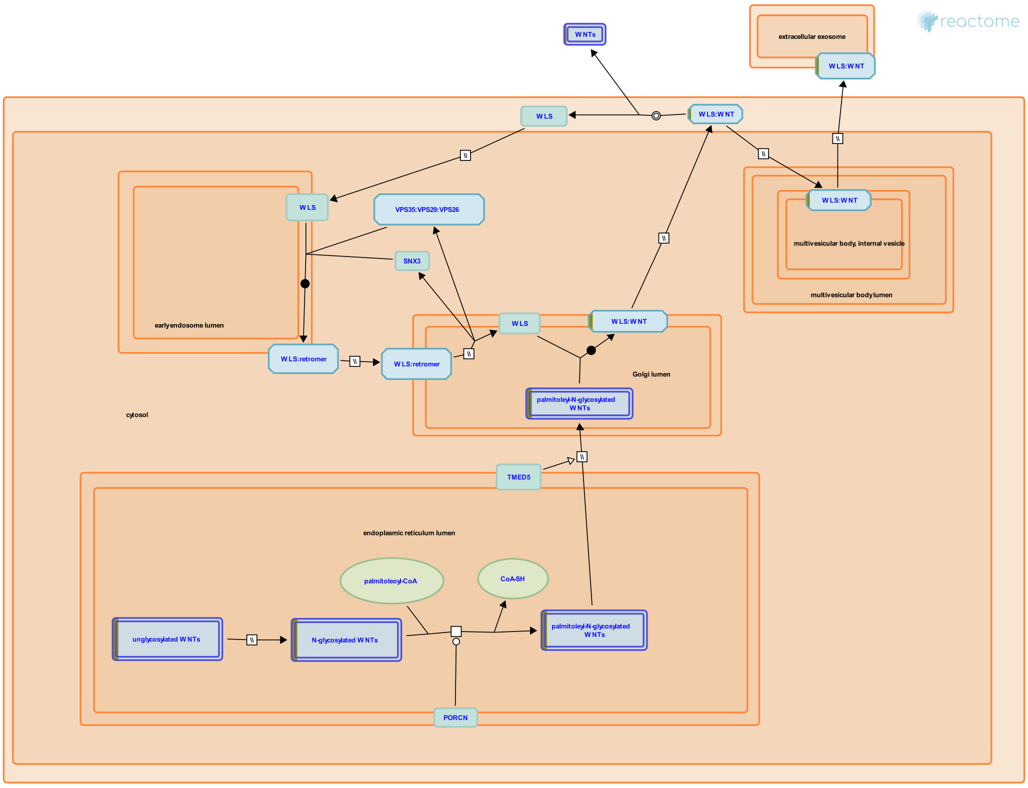


19 WNT proteins have been identified in human cells. The WNTs are members of a conserved metazoan family of secreted morphogens that activate several signaling pathways in the responding cell: the canonical (beta-catenin) WNT signaling cascade and several non-canonical pathways, including the planar cell polarity (PCP), the regulation of intracellular calcium signaling and activation of JNK kinases. WNT proteins exist in a gradient outside the secreting cell and are able to act over both short and long ranges to promote proliferation, changes in cell migration and polarity and tissue homeostasis, among others (reviewed in Saito-Diaz et al, 2012; Willert and Nusse, 2012).

The WNTs are ~40kDa proteins with 23 conserved cysteine residues in the N-terminal that may form intramolecular disulphide bonds. They also contain an N-terminal signal sequence and a number of N-linked glycosylation sites (Janda et al, 2012). In addition to being glycosylated, WNTs are also lipid-modified in the endoplasmic reticulum by a WNT-specific O-acyl-transferase, Porcupine (PORCN), contributing to their characteristic hydrophobicity. PORCN-dependent palmitoylation is required for the secretion of WNT as well as its signaling activity, as either depletion of PORCN or mutation of the conserved serine acylation site results in the intracellular accumulation of WNT ligand (Takada et al, 2006; Barrott et al, 2011; Biechele et al, 2011; reviewed in Willert and Nusse, 2012).

Secretion of WNT requires a number of other dedicated factors including the sorting receptor Wntless (WLS) (also knownas Evi, Sprinter, and GPR177), which binds WNT and escorts it to the cell surface (Banziger et al, 2006; Bartscherer et al, 2006; Goodman et al, 2006). A WNT-specific retromer containing SNX3 is subsequently required for the recycling of WLS back to the Golgi (reviewed in Herr et al, 2012; Johannes and Wunder, 2011). Once at the cell surface, WNT makes extensive contacts with components of the extracellular matrix such as heparan sulphate proteoglycans (HSPGs) and may be bound by any of a number of regulatory proteins, including WIFs and SFRPs. The diffusion of the WNT ligand may be aided by its packing either into WNT multimers, exosomes or onto lipoprotein particles to shield the hydrophobic lipid adducts from the aqueous extracellular environment (Gross et al, 2012; Luga et al, 2012, Korkut et al, 2009; reviewed in Willert and Nusse, 2012).

<br >

## References


Rossant J, Cox BJ & Biechele S (2011). Porcupine homolog is required for canonical Wnt signaling and gastrulation in mouse embryos. Dev. Biol., 355, 275-85.

Kurata T, Satomi Y, Kondoh H, Takada R, Takada S, Takao T, ... Norioka S (2006). Monounsaturated fatty acid modification of Wnt protein: its role in Wnt secretion. Dev. Cell, 11, 791-801.

Wang X, Wallace HA, Page-McCaw A, Lee E, Thorne CA, Chen TW & Saito-Diaz K (2013). The way Wnt works: Components and mechanism. Growth Factors, 31, 1-31.

Thomas C, Garcia KC, Waghray D, Levin AM & Janda CY (2012). Structural basis of Wnt recognition by Frizzled. Science, 337, 59-64.

Luga V, Wrana JL, Inanlou MR, Chiu E, Viloria-Petit AM, Buchanan M, ... Ogunjimi AA (2012). Exosomes mediate stromal mobilization of autocrine Wnt-PCP signaling in breast cancer cell migration. Cell, 151, 1542-56.


## Edit history

| Date | Action | Author |
|---|---|---|
| 2013-03-27 | Created | Rothfels K |
| 2013-04-06 | Authored | Rothfels K |
| 2013-04-12 | Edited | Matthews L |
| 2013-05-24 | Reviewed | Boutros M |
| 2023-05-21 | Modified | Wright A |

## 1 submitted entities found in this pathway, mapping to 1 Reactome entities

| Input | UniProt Id |
|---|---|
| Wnt9a | O14904 |

## 10. Acyl chain remodelling of PC (R-HSA-1482788)

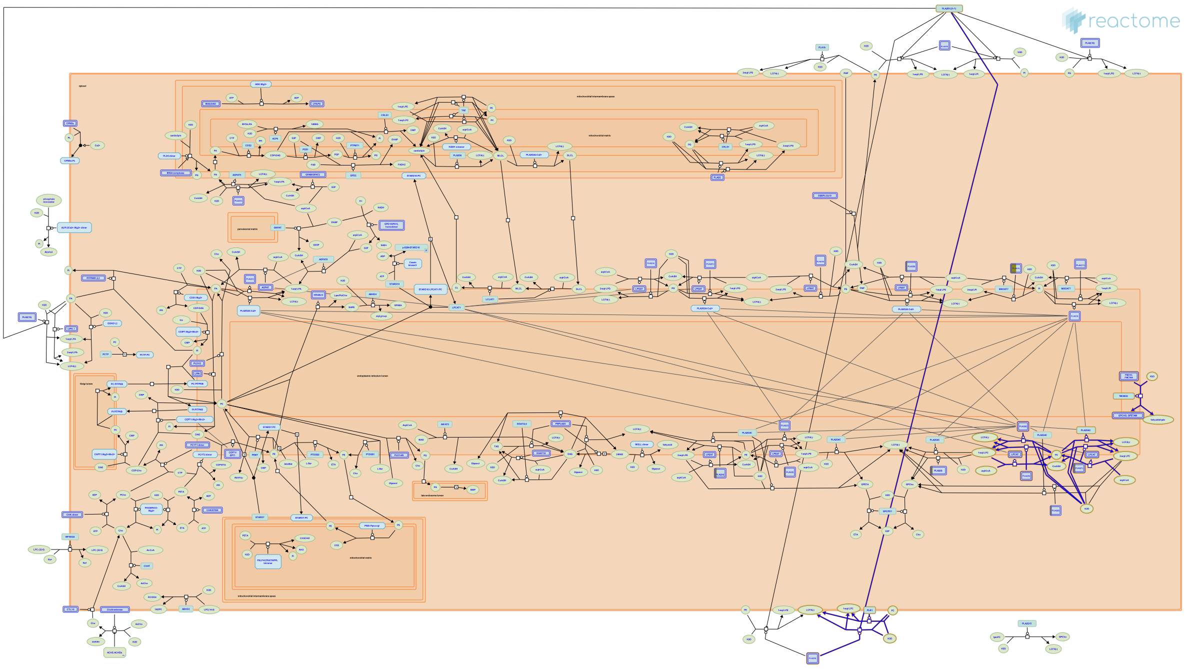

In the acyl chain remodelling pathway (Lands cycle), phosphatidylcholine (PC) is hydrolysed by phopholipases and subsequently reacylated by acyltransferases. These cycles modify the fatty acid composition of glycerophospholipids to generate diverse molecules asymmetrically distributed in the cell membrane (Ghomashchi et al. 2010, Singer et al. 2002, Cao et al. 2008, Zhao et al. 2008).

### References


Lehr M, Gelb MH, Aloulou A, Naika GS, Bollinger JG, Ghomashchi F & Leslie CC (2010). Interfacial kinetic and binding properties of mammalian group IVB phospholipase A2 (cPLA2beta) and comparison with the other cPLA2 isoforms. J Biol Chem, 285, 36100-11.

Bensch WR, Li S, Cao G, Bonacci TM, Moller DE, Bredt DS, ... Konrad RJ (2008). Identification and characterization of a major liver lysophosphatidylcholine acyltransferase. J Biol Chem, 283, 8258-65.

Revett T, Tobin JF, Cao J, Li D, Shan D, Gimeno RE, ... Liu W (2008). Molecular identification of a novel mammalian brain isoform of acyl-CoA:lysophospholipid acyltransferase with prominent ethanolamine lysophospholipid acylating activity, LPEAT2. J Biol Chem, 283, 19049-57.

Gelb MH, Le Calvez C, Ghomashchi F, Rouault M, Singer AG, Sadilek M, ... Nguyen E (2002). Interfacial kinetic and binding properties of the complete set of human and mouse groups I, II, V, X, and XII secreted phospholipases A2. J Biol Chem, 277, 48535-49.


### Edit history

| Date | Action | Author |
| --- | --- | --- |
| 2011-08-12 | Edited | Williams MG |
| 2011-08-12 | Created | Williams MG |
| 2011-09-14 | Authored | Williams MG |
| 2012-05-14 | Reviewed | Wakelam M |
| 2023-05-21 | Modified | Wright A |

## 1 submitted entities found in this pathway, mapping to 1 Reactome entities

| Input | UniProt Id |
|---|---|
| Pla2g4e | Q3MJ16 |

## 11. Notch-HLH transcription pathway (R-HSA-350054)

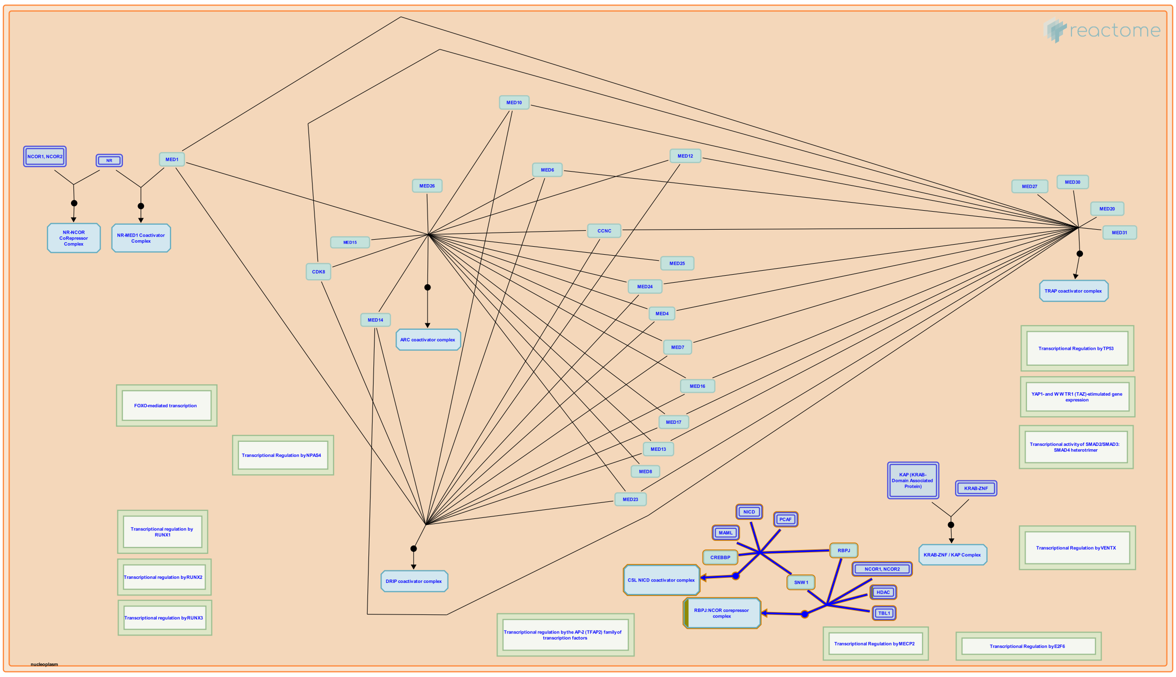


**THE NOTCH-HLH TRANSCRIPTION PATHWAY:**

Notch signaling was first identified in Drosophila, where it has been studied in detail at the genetic, molecular, biochemical and cellular levels (reviewed in Justice, 2002; Bray, 2006; Schweisguth, 2004; Louvri, 2006). In Drosophila, Notch signaling to the nucleus is thought always to be mediated by one specific DNA binding transcription factor, Suppressor of Hairless. In mammals, the homologous genes are called CBF1 (or RBPJkappa), while in worms they are called Lag-1, so that the acronym "CSL" has been given to this conserved transcription factor family. There are at least two human CSL homologues, which are now named RBPJ and RBPJL.

CSL is an example of a bifunctional DNA-binding transcription factor that mediates repression of specific target genes in one context, but activation of the same targets in another context. This bifunctionality is mediated by the association of specific Co-Repressor complexes vs. specific Co-Activator complexes in different contexts, namely in the absence or presence of Notch signaling.

In Drosophila, Su(H) represses target gene transcription in the absence of Notch signaling, but activates target genes during Notch signaling. At least some of the mammalian CSL homologues are believed also to be bifunctional, and to mediate target gene repression in the absence of Notch signaling, and activation in the presence of Notch signaling.

Notch Co-Activator and Co-Repressor complexes: This repression is mediated by at least one specific co-repressor complexes (Co-R) bound to CSL in the absence of Notch signaling. In Drosophila, this co-repressor complex consists of at least three distinct co-repressor proteins: Hairless, Groucho, and dCtBP (Drosophila C-terminal Binding Protein). Hairless has been show to bind directly to Su(H), and Groucho and dCtBP have been shown to bind directly to Hairless (Barolo, 2002). All three of the co-repressor proteins have been shown to be necessary for proper gene regulation during Notch signaling in vivo (Nagel, 2005).

In mammals, the same general pathway and mechanisms are observed, where CSL proteins are bifunctional DNA binding transcription factors (TFs), that bind to Co-Repressor complexes to mediate repression in the absence of Notch signaling, and bind to Co-Activator complexes to mediate activation in the presence of Notch signaling. However, in mammals, there may be multiple co-repressor complexes, rather than the single Hairless co-repressor complex that has been observed in Drosophila.

During Notch signaling in all systems, the Notch transmembrane receptor is cleaved and the Notch intracellular domain (NICD) translocates to the nucleus, where it there functions as a specific transcription co-activator for CSL proteins. In the nucleus, NICD replaces the Co-R complex bound to CSL, thus resulting in de-repression of Notch target genes in the nucleus. Once bound to CSL, NICD and CSL proteins recruit an additional co-activator protein, Mastermind, to form a CSL-NICD-Mam ternary co-activator (Co-A) complex. This Co-A complex was initially thought to be sufficient to mediate activation of at least some Notch target genes. However, there now is evidence that still other co-activators and additional DNA-binding transcription factors are required in at least some contexts (reviewed in Barolo, 2002).

Mammalian CSL Corepressor Complexes: In the absence of activated Notch signaling, DNA-bound CSL proteins recruit a corepressor complex to maintain target genes in the repressed state until Notch is specifically activated. The mammalian corepressor complexes include NCOR complexes, but may also include additional corepressor proteins, such as SHARP (reviewed in Mumm, 2000 and Kovall, 2007). The exact composition of the CSL NCOR complex is not known, but in other pathways the "core" NCOR corepressor complex includes at least one NCOR protein (NCOR1, NCOR2, CIR), one Histone Deacetylase protein (HDAC1, HDAC2, HDAC3, etc), and one TBL1 protein (TBL1X, TBL1XR1) (reviewed in Rosenfeld, 2006). In some contexts, the core NCOR corepressor complex may also recruit additional corepressor proteins or complexes, such as the SIN3 complex, which consists of SIN3 (SIN3A, SIN3B), and SAP30, or other SIN3-associated proteins. An additional CSL - NCOR binding corepressor, SHARP, may also contribute to the CSL corepressor complex in some contexts (Oswald, 2002). The CSL corepressor complex also includes a bifunctional cofactor, SKIP, that is present in both CSL corepressor complexes and CSL coactivator complexes, and may function in the binding of NICD and displacement of the corepressor complex during activated Notch signaling (Zhou, 2000).

Mammalian CSL Coactivator Complexes: Upon activation of Notch signaling, cleavage of the transmembrane Notch receptor releases the Notch Intracellular Domain (NICD), which translocates to the nucleus, where it binds to CSL and displaces the corepressor complex from CSL (reviewed in Mumm, 2000 and Kovall, 2007). The resulting CSL-NICD "binary complex" then recruits an additional coactivator, Mastermind (Mam), to form a ternary complex. The ternary complex then recruits additional, more general coactivators, such as CREB Binding Protein (CBP), or the related p300 coactivator, and a number of Histone Acetytransferase (HAT) proteins, including GCN5 and PCAF (Fryer, 2002). There is evidence that Mam also can subsequently recruit specific kinases that phosphorylate NICD, to downregulate its function and turn off Notch signaling (Fryer, 2004).

Combinatorial Complexity in Transcription Cofactor Complexes: HDAC9 has at least 7 splice isoforms, with some having distinct interaction and functional properties. Isoforms 6 and 7 interact with NCOR1. Isoforms 1 and 4 interact with MEF2 (Sparrow, 1999), which is a specific DNA-binding cofactor for a subset of HLH proteins. Isoform 3 interacts with both NCOR1 and MEF2. Although many HDACs only have one or two isoforms, this complexity for HDAC9 illustrates the level of transcript complexity and functional specificity that such "general" transcriptional cofactors can have.

## References


Dillinger K, Oswald F, Zechner U, Liptay S, Ludwig L, Kostezka U, ... Astrahantseff K (2002). SHARP is a novel component of the Notch/RBP-Jkappa signalling pathway. EMBO J, 21, 5417-26.

Schwabe JW & Ariyoshi M (2003). A conserved structural motif reveals the essential transcriptional repression function of Spen proteins and their role in developmental signaling. Genes Dev, 17, 1909-20.

Schweisguth F (2004). Notch signaling activity. Curr Biol, 14, R129-38.

Fryer CJ, Jones KA & White JB (2004). Mastermind recruits CycC:CDK8 to phosphorylate the Notch ICD and coordinate activation with turnover. Mol Cell, 16, 509-20.

Kovall RA & Wilson JJ (2006). Crystal structure of the CSL-Notch-Mastermind ternary complex bound to DNA. Cell, 124, 985-96.


## Edit history

| Date | Action | Author |
|---|---|---|
| 2008-02-09 | Edited | Caudy M |
| 2008-02-09 | Authored | Caudy M |
| 2008-05-14 | Created | Caudy M |
| 2023-05-21 | Modified | Wright A |

## 1 submitted entities found in this pathway, mapping to 1 Reactome entities

| Input | UniProt Id |
|---|---|
| Hdac9 | Q9UKV0 |

## 12. Acyl chain remodelling of PE (R-HSA-1482839)

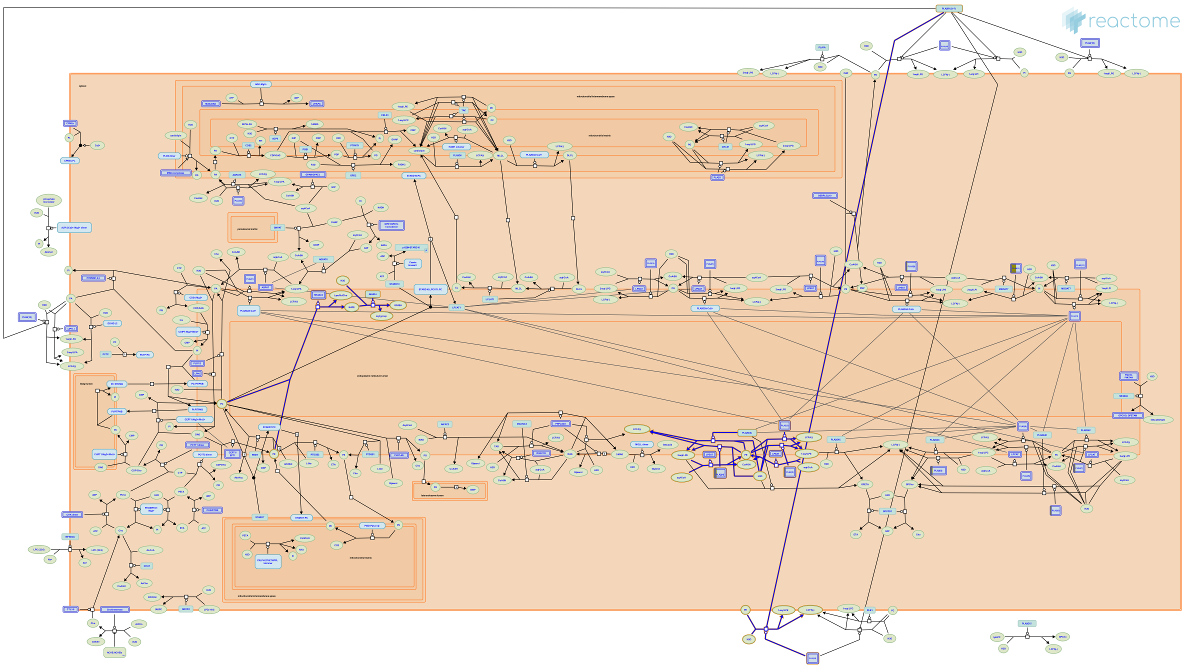

In the acyl chain remodelling pathway (Lands cycle), phosphatidylethanolamine (PE) is hydrolyzed by phopholipases and subsequently reacylated by acyltransferases. These cycles modify the fatty acid composition of glycerophospholipids to generate diverse molecules asymmetrically distributed in the cell membrane (Ghomashchi et al. 2010, Singer et al. 2002, Cao et al. 2008, Zhao et al. 2008, Hishikawa et al. 2008).

### References


Lehr M, Gelb MH, Aloulou A, Naika GS, Bollinger JG, Ghomashchi F & Leslie CC (2010). Interfacial kinetic and binding properties of mammalian group IVB phospholipase A2 (cPLA2beta) and comparison with the other cPLA2 isoforms. J Biol Chem, 285, 36100-11.

Bensch WR, Li S, Cao G, Bonacci TM, Moller DE, Bredt DS, ... Konrad RJ (2008). Identification and characterization of a major liver lysophosphatidylcholine acyltransferase. J Biol Chem, 283, 8258-65.

Revett T, Tobin JF, Cao J, Li D, Shan D, Gimeno RE, ... Liu W (2008). Molecular identification of a novel mammalian brain isoform of acyl-CoA:lysophospholipid acyltransferase with prominent ethanolamine lysophospholipid acylating activity, LPEAT2. J Biol Chem, 283, 19049-57.

Gelb MH, Le Calvez C, Ghomashchi F, Rouault M, Singer AG, Sadilek M, ... Nguyen E (2002). Interfacial kinetic and binding properties of the complete set of human and mouse groups I, II, V, X, and XII secreted phospholipases A2. J Biol Chem, 277, 48535-49.

Shindou H, Nakanishi H, Taguchi R, Kobayashi S, Shimizu T & Hishikawa D (2008). Discovery of a lysophospholipid acyltransferase family essential for membrane asymmetry and diversity. Proc Natl Acad Sci U S A, 105, 2830-5.


### Edit history

| Date | Action | Author |
|---|---|---|
| 2011-08-12 | Edited | Williams MG |
| 2011-08-12 | Created | Williams MG |

| Date | Action | Author |
| --- | --- | --- |
| 2011-09-14 | Authored | Williams MG |
| 2012-05-14 | Reviewed | Wakelam M |
| 2023-05-21 | Modified | Wright A |

## 1 submitted entities found in this pathway, mapping to 1 Reactome entities

| Input | UniProt Id |
| --- | --- |
| Pla2g4e | Q3MJ16 |

## 13. NOTCH1 Intracellular Domain Regulates Transcription (R-HSA-2122947)

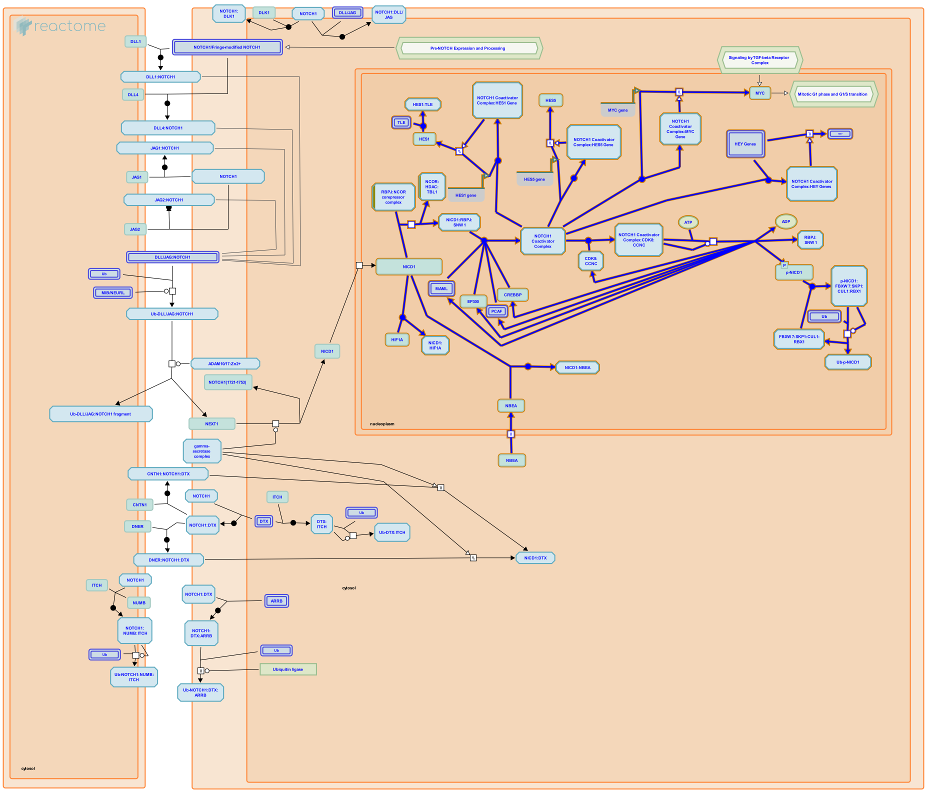

**Cellular compartments:** nucleoplasm.

NICD1 produced by activation of NOTCH1 in response to Delta and Jagged ligands (DLL/JAG) presented in trans, traffics to the nucleus where it acts as a transcription regulator. In the nucleus, NICD1 displaces the NCOR corepressor complex from RBPJ (CSL). When bound to the co-repressor complex that includes NCOR proteins (NCOR1 and NCOR2) and HDAC histone deacetylases, RBPJ (CSL) represses transcription of NOTCH target genes (Kao et al. 1998, Zhou et al. 2000, Perissi et al. 2004, Perissi et al. 2008). Once the co-repressor complex is displaced, NICD1 recruits MAML (mastermind-like) to RBPJ, while MAML recruits histone acetyltransferases EP300 (p300) and PCAF, resulting in formation of the NOTCH coactivator complex that activates transcription from NOTCH regulatory elements. The minimal functional NOTCH coactivator complex that activates transcription from NOTCH regulatory elements is a heterotrimer composed of NICD, MAML and RBPJ (Fryer et al. 2002, Wallberg et al. 2002, Nam et al. 2006).

NOTCH1 coactivator complex is known to activate transcription of HES1 (Jarriault et al. 1995), HES5 (Arnett et al. 2010), HEY genes (Fischer et al. 2004, Leimeister et al. 2000, Maier et al. 2000, Arnett et al. 2010) and MYC (Palomero et al. 2006) and likely regulates transcription of many other genes (Wang et al. 2011). NOTCH1 coactivator complex on any specific regulatory element may involve additional transcriptional regulatory proteins. HES1 binds TLE proteins, forming an evolutionarily conserved transcriptional corepressor involved in regulation of neurogenesis, segmentation and sex determination (Grbavec et al. 1996, Fisher et al. 1996, Paroush et al. 1994).

After NOTCH1 coactivator complex is assembled on a NOTCH-responsive promoter, MAML (mastermind-like) recruits CDK8 in complex with cyclin C, triggering phosphorylation of conserved serine residues in TAD and PEST domains of NICD1 by CDK8. Phosphorylated NICD1 is recognized by the E3 ubiquitin ligase FBXW7 which ubiquitinates NICD1, leading to degradation of NICD1 and downregulation of NOTCH1 signaling. FBXW7-mediated ubiquitination and degradation of NOTCH1 depend on C-terminally located PEST domain sequences in NOTCH1 (Fryer et al. 2004, Oberg et al. 2001, Wu et al. 2001). The PEST domain of NOTCH1 and the substrate binding WD40 domain of FBXW7 are frequent targets of mutations in T-cell acute lymphoblastic leukemia - T-ALL (Welcker and Clurman 2008).

NICD1, which normally has a short half-life, can be stabilized by binding to the hypoxia-inducable factor 1-alpha (HIF1A) which accumulates in the nucleus when oxygen levels are low. This results in HIF1A-induced inhibition of cellular differentiation that is NOTCH-dependent (Gustafsson et al. 2005).

## References


Blacklow SC, Nam Y, Aster JC, Song L & Sliz P (2006). Structural basis for cooperativity in recruitment of MAML coactivators to Notch transcription complexes. Cell, 124, 973-83.

Gessler M, Schumacher N, Maier M, Sendtner M & Fischer A (2004). The Notch target genes Hey1 and Hey2 are required for embryonic vascular development. Genes Dev, 18, 901-11.

Grbavec D & Stifani S (1996). Molecular interaction between TLE1 and the carboxyl-terminal domain of HES-1 containing the WRPW motif. Biochem Biophys Res Commun, 223, 701-5.

Kieff E, Ashworth T, Blacklow SC, Wong H, Schug J, Bernstein BE, ... Zou J (2011). Genome-wide analysis reveals conserved and divergent features of Notch1/RBPJ binding in human and murine T-lymphoblastic leukemia cells. Proc. Natl. Acad. Sci. U.S.A., 108, 14908-13.

Lendahl U, Wallberg AE, Pedersen K & Roeder RG (2002). p300 and PCAF act cooperatively to mediate transcriptional activation from chromatin templates by notch intracellular domains in vitro. Mol Cell Biol, 22, 7812-9.


## Edit history

| Date | Action | Author |
|---|---|---|
| 2011-11-14 | Authored | Egan SE, Orlic-Milacic M |
| 2012-02-06 | Reviewed | Haw R |
| 2012-02-07 | Edited | D'Eustachio P |
| 2012-02-11 | Edited | Orlic-Milacic M |
| 2012-02-14 | Created | Orlic-Milacic M |
| 2023-05-21 | Modified | Wright A |

## 1 submitted entities found in this pathway, mapping to 1 Reactome entities

| Input | UniProt Id |
|---|---|
| Hdac9 | Q9UKV0 |

## 14. Constitutive Signaling by NOTCH1 PEST Domain Mutants (R-HSA-2644606)

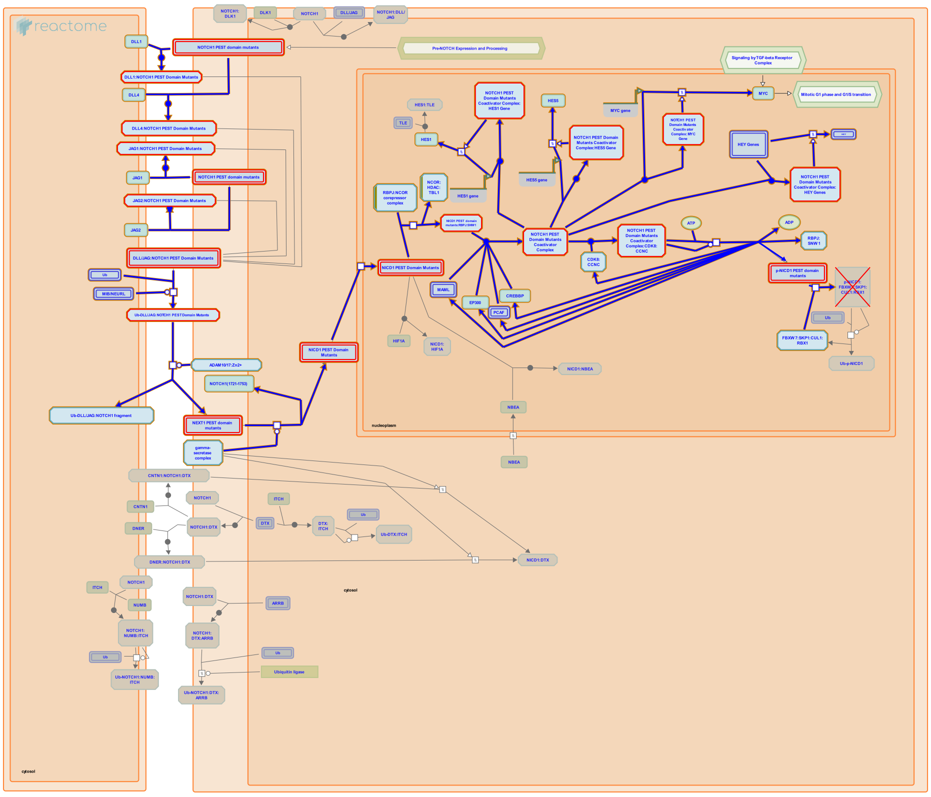

**Diseases:** cancer, T-cell acute lymphoblastic leukemia.

As NOTCH1 PEST domain is intracellular, NOTCH1 PEST domain mutants are expected to behave as the wild-type NOTCH1 with respect to ligand binding and proteolytic cleavage mediated activation of signaling. However, once the NICD1 fragment of NOTCH1 is released, PEST domain mutations prolong its half-life and transcriptional activity through interference with FBXW7 (FBW7)-mediated ubiquitination and degradation of NICD1 (Weng et al. 2004, Thompson et al. 2007, O'Neil et al. 2007). All NOTCH1 PEST domain mutants annotated here (NOTCH1 Q2395*, NOTCH1 Q2440*, NOTCH1 P2474Afs*4 and NOTCH1 P2514Rfs*4) either have a truncated PEST domain or lack the PEST domain completely.

### References


Lee W, Blacklow SC, Silverman LB, Look AT, Sanchez-Irizarry C, Aster JC, ... Morris JP 4th (2004). Activating mutations of NOTCH1 in human T cell acute lymphoblastic leukemia. Science, 306, 269-71.

Basso G, Sulis ML, Buonamici S, Thompson BJ, Aifantis I, Palomero T, ... Ferrando AA (2007). The SCFFBW7 ubiquitin ligase complex as a tumor suppressor in T cell leukemia. J Exp Med, 204, 1825-35.

Draetta G, Rao S, Sears R, Strack P, Tibbitts D, Hardwick J, ... Clurman BE (2007). FBW7 mutations in leukemic cells mediate NOTCH pathway activation and resistance to gamma-secretase inhibitors. J Exp Med, 204, 1813-24.


### Edit history

| Date | Action | Author |
|---|---|---|
| 2012-11-22 | Created | Orlic-Milacic M |

| Date | Action | Author |
|---|---|---|
| 2013-01-04 | Authored | Orlic-Milacic M |
| 2013-01-09 | Edited | Jassal B |
| 2013-02-10 | Reviewed | Haw R |
| 2023-03-08 | Modified | Matthews L |

## 1 submitted entities found in this pathway, mapping to 1 Reactome entities

| Input | UniProt Id |
|---|---|
| Hdac9 | Q9UKV0 |

## 15. Constitutive Signaling by NOTCH1 HD+PEST Domain Mutants (R-HSA-2894862)

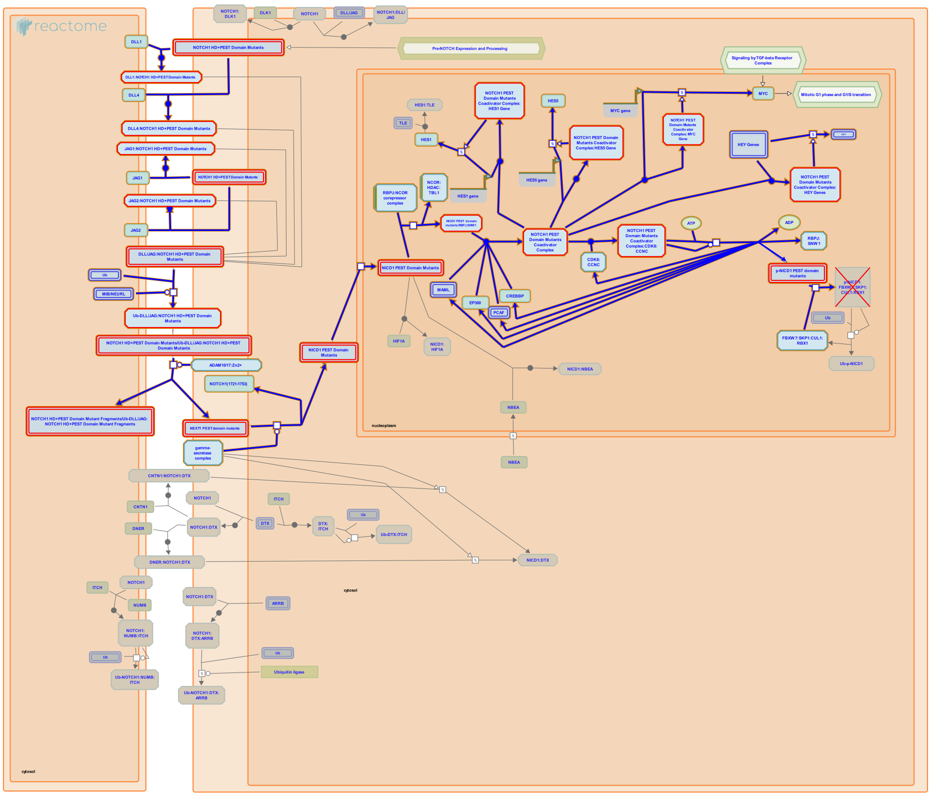

**Diseases:** cancer, T-cell acute lymphoblastic leukemia.

When found in cis, HD and PEST domain mutations act synergistically, increasing NOTCH1 transcriptional activity up to ~40-fold, compared with up to ~10-fold and up to ~2-fold increase with HD mutations alone and PEST domain mutations alone, respectively (Weng et al. 2004). HD domain mutations enable spontaneous, ligand-independent, proteolytic release of the NICD1 fragment, although mutants remain responsive to ligand binding (Malecki et al. 2006), while PEST domain mutations prolong NICD1 half-life and transcriptional activity through interference with FBXW7 (FBW7)-mediated ubiquitination and degradation (Thompson et al. 2007, O'Neil et al. 2007). NOTCH1 HD+PEST domain mutants annotated here are NOTCH1 L1600P;P2514Rfs*4, NOTCH1 L1600P;Q2440*, NOTCH1 L1600P;Q2395* and NOTCH1 L1574P;P2474Afs*4.

### References


Lee W, Blacklow SC, Silverman LB, Look AT, Sanchez-Irizarry C, Aster JC, ... Morris JP 4th (2004). Activating mutations of NOTCH1 in human T cell acute lymphoblastic leukemia. Science, 306, 269-71.

Basso G, Sulis ML, Buonamici S, Thompson BJ, Aifantis I, Palomero T, ... Ferrando AA (2007). The SCFFBW7 ubiquitin ligase complex as a tumor suppressor in T cell leukemia. J Exp Med, 204, 1825-35.

Draetta G, Rao S, Sears R, Strack P, Tibbitts D, Hardwick J, ... Clurman BE (2007). FBW7 mutations in leukemic cells mediate NOTCH pathway activation and resistance to gamma-secretase inhibitors. J Exp Med, 204, 1813-24.

Mitchell JL, Malecki MJ, Blacklow SC, Xu ML, Sanchez-Irizarry C, Aster JC & Histen G (2006). Leukemia-associated mutations within the NOTCH1 heterodimerization domain fall into at least two distinct mechanistic classes. Mol. Cell. Biol., 26, 4642-51.

## Edit history

| Date | Action | Author |
|---|---|---|
| 2013-01-02 | Created | Orlic-Milacic M |
| 2013-01-04 | Authored | Orlic-Milacic M |
| 2013-01-09 | Edited | Jassal B |
| 2013-02-10 | Reviewed | Haw R |
| 2023-03-08 | Modified | Matthews L |

## 1 submitted entities found in this pathway, mapping to 1 Reactome entities

| Input | UniProt Id |
|---|---|
| Hdac9 | Q9UKV0 |

## 16. Signaling by NOTCH1 HD+PEST Domain Mutants in Cancer (R-HSA-2894858)

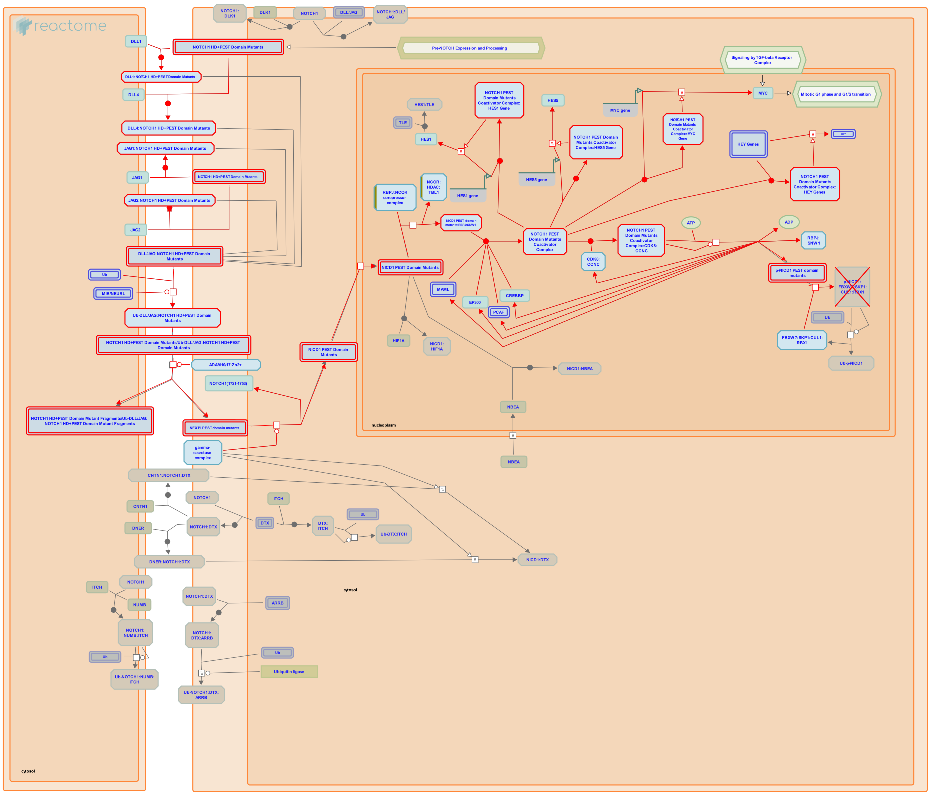

**Diseases:** cancer, T-cell acute lymphoblastic leukemia.

Mutations in the heterodimerization domain (HD) and PEST domain of NOTCH1 are frequently found in cis in T-cell acute lymphoblastic leukemia. While HD mutations alone result in up to ~10-fold increase in NOTCH1 transcriptional activity and PEST domain mutations alone result in up to ~2-fold increase in NOTCH1 transcriptional activity, in cis mutations of HD and PEST domains act synergistically, increasing NOTCH1 transcriptional activity up to ~40-fold (Weng et al. 2004).

### References


Lee W, Blacklow SC, Silverman LB, Look AT, Sanchez-Irizarry C, Aster JC, ... Morris JP 4th (2004). Activating mutations of NOTCH1 in human T cell acute lymphoblastic leukemia. Science, 306, 269-71.


### Edit history

| Date | Action | Author |
|---|---|---|
| 2013-01-02 | Created | Orlic-Milacic M |
| 2013-01-04 | Authored | Orlic-Milacic M |
| 2013-01-09 | Edited | Jassal B |
| 2013-02-10 | Reviewed | Haw R |
| 2023-03-08 | Modified | Matthews L |

### 1 submitted entities found in this pathway, mapping to 1 Reactome entities

| Input | UniProt Id |
|---|---|
| Hdac9 | Q9UKV0 |

| Input | UniProt Id |
|---|---|

## 17. Signaling by NOTCH1 PEST Domain Mutants in Cancer (R-HSA-2644602)

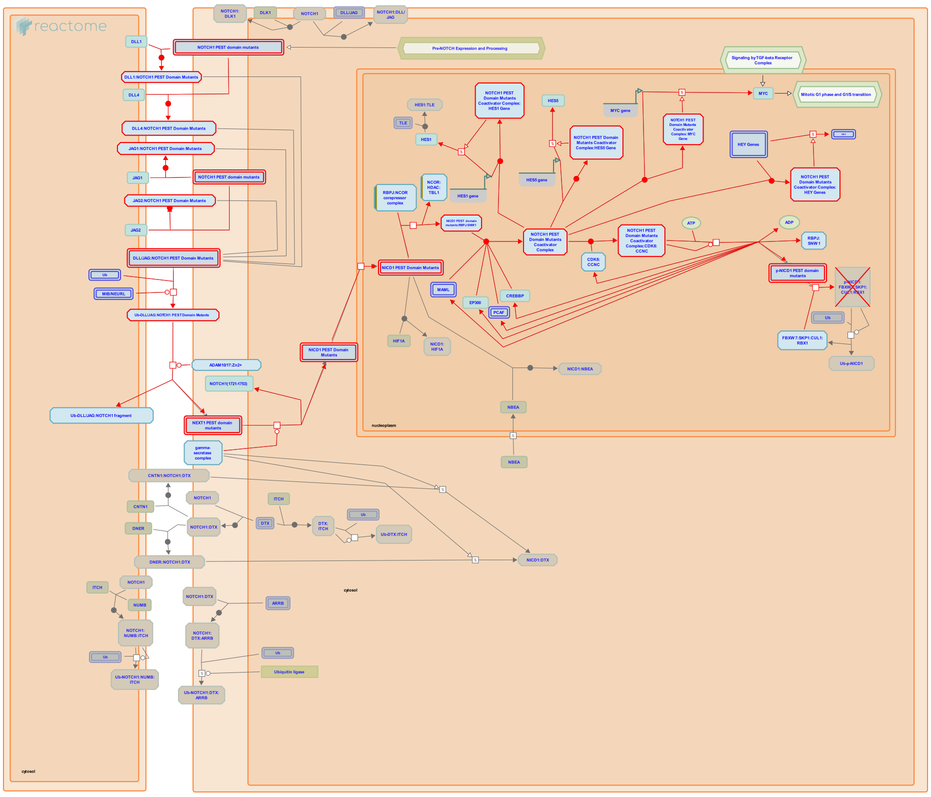

**Diseases:** cancer.

NOTCH1 PEST domain mutations are frequently found in T-cell acute lymphoblastic leukemia (T-ALL). PEST domain mutations interfere with ubiquitination-mediated NOTCH1 downregulation and result in prolonged half-life of the intracellular NOTCH1 fragment, NICD1, and increased NICD1 transcriptional activity (Weng et al. 2004, Thompson et al. 2007, O'Neil et al. 2007).

### References


Lee W, Blacklow SC, Silverman LB, Look AT, Sanchez-Irizarry C, Aster JC, ... Morris JP 4th (2004). Activating mutations of NOTCH1 in human T cell acute lymphoblastic leukemia. Science, 306, 269-71.

Basso G, Sulis ML, Buonamici S, Thompson BJ, Aifantis I, Palomero T, ... Ferrando AA (2007). The SCFFBW7 ubiquitin ligase complex as a tumor suppressor in T cell leukemia. J Exp Med, 204, 1825-35.

Draetta G, Rao S, Sears R, Strack P, Tibbitts D, Hardwick J, ... Clurman BE (2007). FBW7 mutations in leukemic cells mediate NOTCH pathway activation and resistance to gamma-secretase inhibitors. J Exp Med, 204, 1813-24.


### Edit history

| Date | Action | Author |
|---|---|---|
| 2012-11-22 | Created | Orlic-Milacic M |
| 2013-01-04 | Authored | Orlic-Milacic M |
| 2013-01-09 | Edited | Jassal B |
| 2013-02-10 | Reviewed | Haw R |

| Date | Action | Author |
| --- | --- | --- |
| 2023-03-08 | Modified | Matthews L |

## 1 submitted entities found in this pathway, mapping to 1 Reactome entities

| Input | UniProt Id |
| --- | --- |
| Hdac9 | Q9UKV0 |

## 18. Signaling by NOTCH1 in Cancer (R-HSA-2644603)

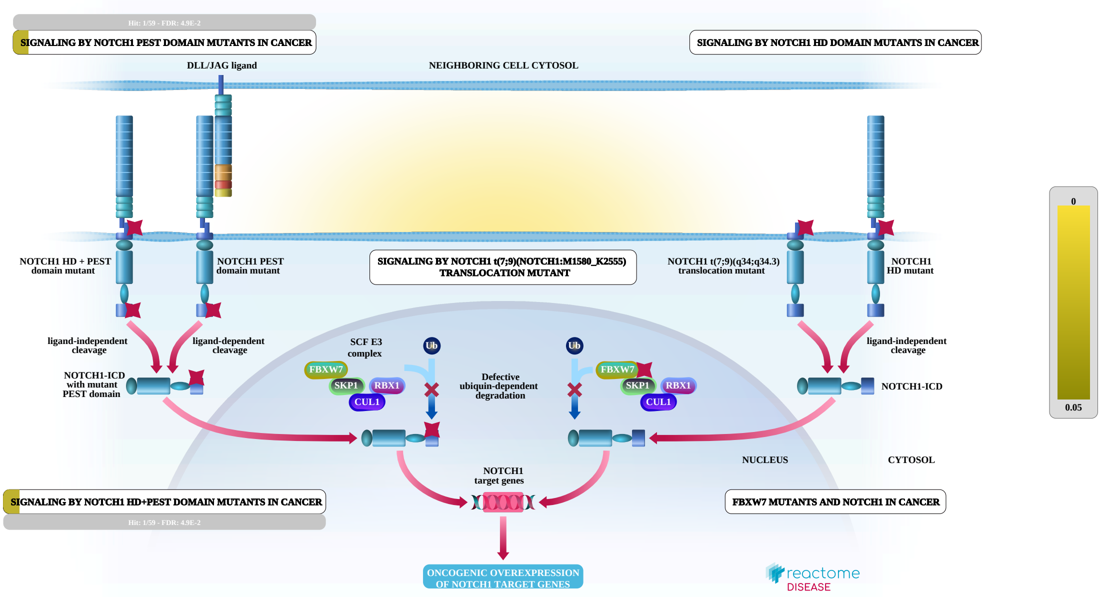


**Diseases:** cancer.

Human NOTCH1 was cloned as a chromosome 9 gene, translocated to the T-cell beta receptor (TCBR) promoter on chromosome 7 in T-cell acute lymphoblastic leukemia (T-ALL) (Ellisen et al. 1991). This translocation, present in only a small percentage of T-ALL patients, results in the over-expression of a truncated NOTCH1 receptor, which lacks almost the entire extracellular domain, in T lymphocytes. Oncogenic NOTCH1 mutations were subsequently found to be present in >50% of T-ALL patients, with hotspots in the heterodimerization domain (HD domain) and PEST domain of NOTCH1 (Weng et al. 2004).

Normal NOTCH1 becomes activated by binding DLL (DLL1 or DLL4) or JAG (JAG1 or JAG2) ligands expressed on the surface of a neighboring cell, which leads to proteolytic cleavage of NOTCH1 by ADAM10/17 and gamma-secretase, and release of the NOTCH1 intracellular domain (NICD1) which regulates expression of genes that play important roles in the development of T lymphocytes (Washburn et al. 1997. Radtke et al. 1999, Maillard et al. 2004, Sambandam et al. 2005, Tan et al. 2005). Mutations in the HD domain, responsible for association of NOTCH1 extracellular and trans-membrane regions after furin-mediated cleavage of NOTCH1 precursor, as well as the truncation of the NOTCH1 extracellular domain by the rare T-ALL translocation, enable constitutive production of NICD1, in the absence of ligand binding (Malecki et al. 2006, Ellisen et al. 1991).

Mutations in the NOTCH1 PEST domain interfere with FBXW7 (FBW7)-mediated ubiquitination and degradation of NICD1, resulting in prolonged half-life and increased transcriptional activity of NICD1, which promotes growth and division of T-lymphocytes (Weng et al. 2004, Thompson et al. 2007, O'Neil et al. 2007).

Mutations in the HD domain and PEST domain of NOTCH1 are frequently found in cis in T-ALL. While HD mutations alone result in up to ~10-fold increase in NOTCH1 transcriptional activity and PEST domain mutations alone result in up to ~2-fold increase in NOTCH1 transcriptional activity, in cis mutations of HD and PEST domains act synergistically, increasing NOTCH1 transcriptional activity up to ~40-fold (Weng et al. 2004).

FBXW7 (FBW7), a component of the SCF (SKP1, CUL1, and F-box protein) ubiquitin ligase complex SCF-FBW7 involved in the degradation of NOTCH1 (Oberg et al. 2001, Wu et al. 2001, Fryer et al. 2004), is subject to loss of function mutations in T-ALL (Akhoondi et al. 2007, Thompson et al. 2007, O'Neil et al. 2007) which are mutually exclusive with NOTCH1 PEST domain mutations (Thompson et al. 2007, O'Neil et al. 2007).

Although gamma-secretase inhibitors (GSIs) are successfully used in vitro to inhibit NOTCH1 signaling in T-ALL cell lines, the gamma-secretase complex has many other substrates besides NOTCH. The specificity of GSIs is therefore limited and, as they are not considered to be particularly promising drugs for the clinical treatment of T-ALL (reviewed by Purow, 2012), they have not been annotated.

For a recent review of NOTCH1 signaling in cancer, please refer to Grabher et al. 2006.

## References


Lee W, Blacklow SC, Silverman LB, Look AT, Sanchez-Irizarry C, Aster JC, ... Morris JP 4th (2004). Activating mutations of NOTCH1 in human T cell acute lymphoblastic leukemia. Science, 306, 269-71.

von Boehmer H, Look AT & Grabher C (2006). Notch 1 activation in the molecular pathogenesis of T-cell acute lymphoblastic leukaemia. Nat Rev Cancer, 6, 347-59.

Basso G, Sulis ML, Buonamici S, Thompson BJ, Aifantis I, Palomero T, ... Ferrando AA (2007). The SCFFBW7 ubiquitin ligase complex as a tumor suppressor in T cell leukemia. J Exp Med, 204, 1825-35.

Das I, Wu G, Deshaies RJ, Kitajewski J, Li J, Pauley A, ... Lyapina S (2001). SEL-10 is an inhibitor of notch signaling that targets notch for ubiquitin-mediated protein degradation. Mol Cell Biol, 21, 7403-15.

Carpenter AC, Xu L, Maillard I, Pear WS, Sai H, Aster JC, ... Rodriguez CG (2004). Mastermind critically regulates Notch-mediated lymphoid cell fate decisions. Blood, 104, 1696-702.


## Edit history

| Date | Action | Author |
|---|---|---|
| 2012-11-22 | Created | Orlic-Milacic M |
| 2013-01-04 | Authored | Orlic-Milacic M |
| 2013-01-09 | Edited | Jassal B |
| 2013-02-10 | Reviewed | Haw R |
| 2023-03-08 | Modified | Matthews L |

## 1 submitted entities found in this pathway, mapping to 1 Reactome entities

| Input | UniProt Id |
|---|---|
| Hdac9 | Q9UKV0 |

## 19. Signaling by NOTCH1 (R-HSA-1980143)

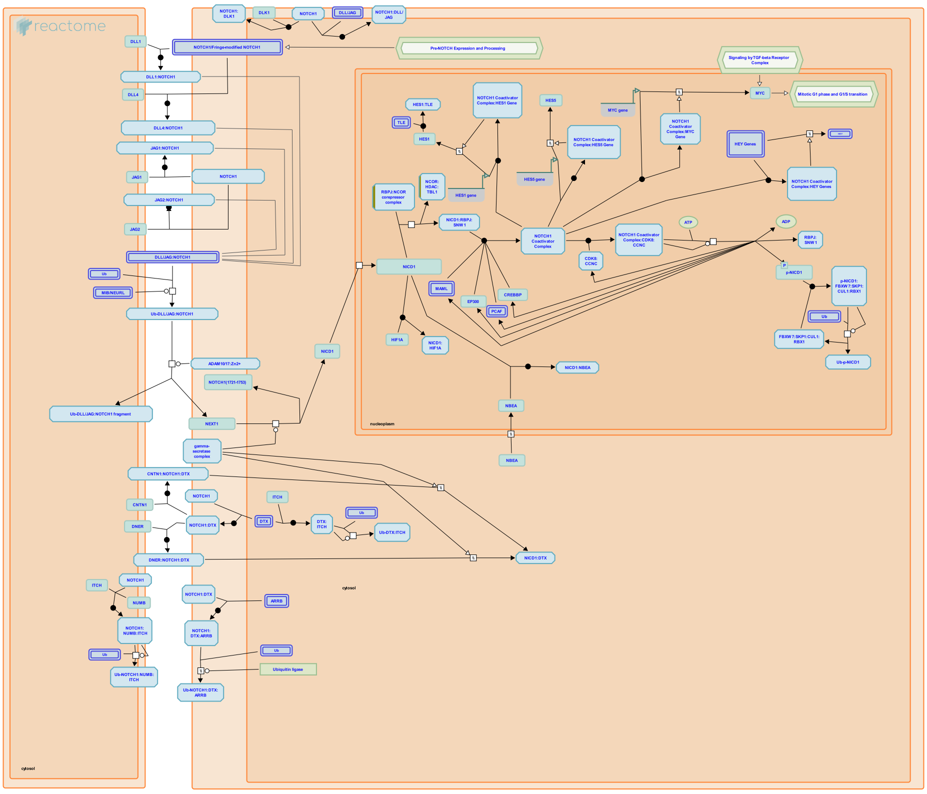

**Cellular compartments:** cytosol, plasma membrane, nucleoplasm.

NOTCH1 functions as both a transmembrane receptor presented on the cell surface and as a transcriptional regulator in the nucleus.

NOTCH1 receptor presented on the plasma membrane is activated by a membrane bound ligand expressed in trans on the surface of a neighboring cell. In trans, ligand binding triggers proteolytic cleavage of NOTCH1 and results in release of the NOTCH1 intracellular domain, NICD1, into the cytosol.

NICD1 translocates to the nucleus where it associates with RBPJ (also known as CSL or CBF) and mastermind-like (MAML) proteins (MAML1, MAML2 or MAML3; possibly also MAMLD1) to form NOTCH1 coactivator complex. NOTCH1 coactivator complex activates transcription of genes that possess RBPJ binding sites in their promoters.

### References


Das I, Wu G, Deshaies RJ, Kitajewski J, Li J, Pauley A, ... Lyapina S (2001). SEL-10 is an inhibitor of notch signaling that targets notch for ubiquitin-mediated protein degradation. Mol Cell Biol, 21, 7403-15.

Clurman BE & Welcker M (2008). FBW7 ubiquitin ligase: a tumour suppressor at the crossroads of cell division, growth and differentiation. Nat Rev Cancer, 8, 83-93.

Ingham PW, Paroush Z, Finley RL Jr, Brent R, Kidd T, Ish-Horowicz D & Wainwright SM (1994). Groucho is required for Drosophila neurogenesis, segmentation, and sex determination and interacts directly with hairy-related bHLH proteins. Cell, 79, 805-15.

Gessler M, Schumacher N, Steidl C & Leimeister C (2000). Analysis of HeyL expression in wild-type and Notch pathway mutant mouse embryos. Mech Dev, 98, 175-8.

Kengaku M, Eiraku M, Ono K, Kaneko M, Fujishima K, Tohgo A & Hirano T (2005). DNER acts as a neuron-specific Notch ligand during Bergmann glial development. Nat Neurosci, 8, 873-80.

## Edit history

| Date | Action | Author |
|---|---|---|
| 2011-11-09 | Created | Orlic-Milacic M |
| 2011-11-14 | Authored | Egan SE, Orlic-Milacic M |
| 2012-02-06 | Reviewed | Haw R |
| 2012-02-07 | Edited | D'Eustachio P |
| 2012-02-11 | Edited | Orlic-Milacic M |
| 2023-05-21 | Modified | Wright A |

## 1 submitted entities found in this pathway, mapping to 1 Reactome entities

| Input | UniProt Id |
|---|---|
| Hdac9 | Q9UKV0 |

## 20. Class B/2 (Secretin family receptors) (R-HSA-373080)

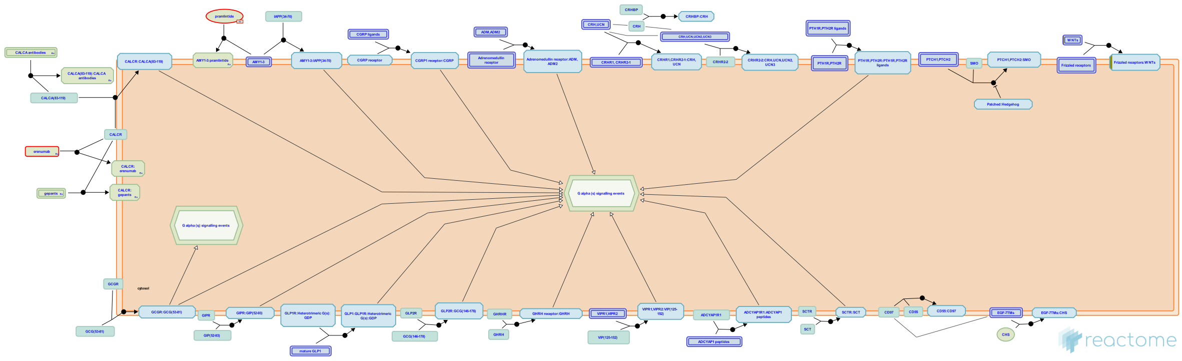

This family is known as Family B (secretin-receptor family, family 2) G-protein-coupled receptors. Family B GPCRs include secretin, calcitonin, parathyroid hormone/parathyroid hormone-related peptides and vasoactive intestinal peptide receptors; all of which activate adenylyl cyclase and the phosphatidyl-inositol-calcium pathway (Harmar AJ, 2001).

### References


Harmar AJ (2001). Family-B G-protein-coupled receptors. Genome Biol, 2, REVIEWS3013.


### Edit history

| Date | Action | Author |
|---|---|---|
| 2008-07-14 | Edited | Jassal B |
| 2008-07-14 | Authored | Jassal B |
| 2008-07-14 | Created | Jassal B |
| 2009-05-29 | Reviewed | D'Eustachio P |
| 2023-05-21 | Modified | Wright A |

### 1 submitted entities found in this pathway, mapping to 1 Reactome entities

| Input | UniProt Id |
|---|---|
| Wnt9a | O14904 |

## 21. Metabolism of water-soluble vitamins and cofactors (R-HSA-196849)

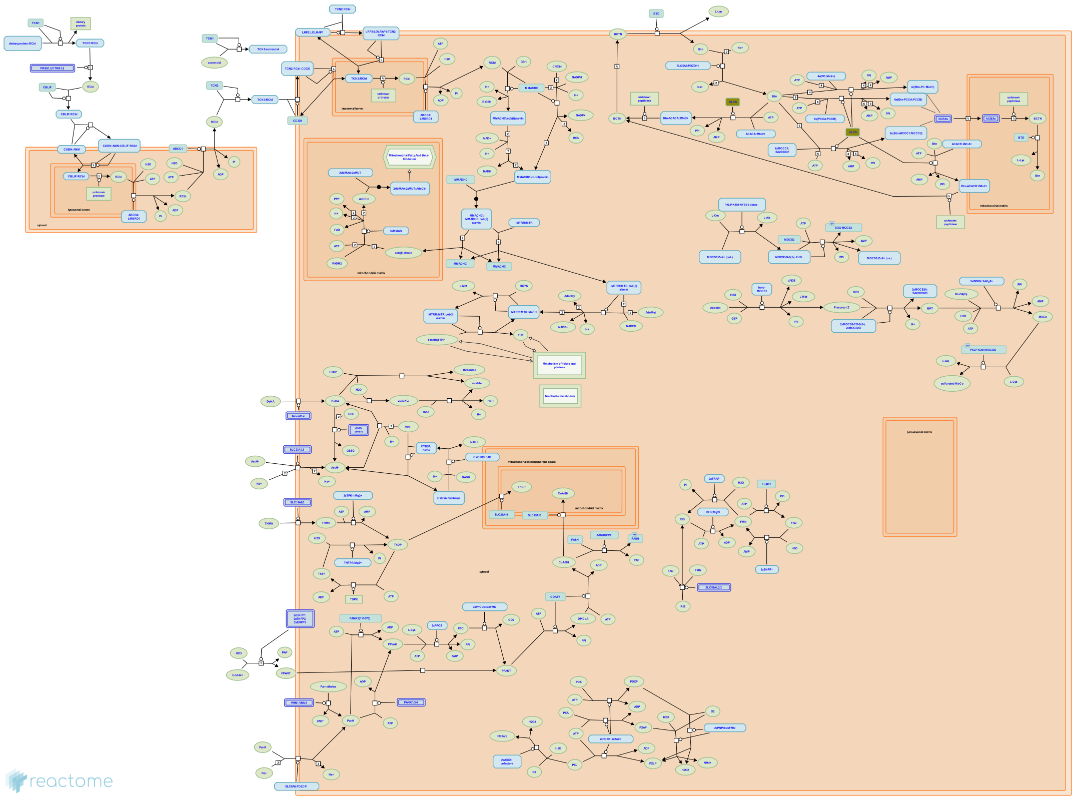

Vitamins are a diverse group of organic compounds, required in small amounts in the diet. They have distinct biochemical roles, often as coenzymes, and are either not synthesized or synthesized only in limited amounts by human cells. Vitamins are classified according to their solubility, either fat-soluble or water-soluble. The physiological processes dependent on vitamin-requiring reactions include many aspects of intermediary metabolism, vision, bone formation, and blood coagulation, and vitamin deficiencies are associated with a correspondingly diverse and severe group of diseases.

Water-soluble vitamins include ascorbate (vitamin C) and the members of the B group: thiamin (vitamin B1), riboflavin (B2), niacin (B3), pantothenate (B5), pyridoxine (B6), biotin (B7), folate (B9), and cobalamin (B12). Metabolic processes annotated here include the synthesis of thiamin pyrophosphate (TPP) from thiamin (B1), the synthesis of FMN and FAD from riboflavin (B2), the synthesis of nicotinic acid (niacin - B3) from tryptophan, the synthesis of Coenzyme A from pantothenate (B5), features of the metabolism of folate (B9), the uptake, transport, and metabolism of cobalamin (B12), and molybdenum cofactor biosynthesis.

### References

### Edit history

| Date | Action | Author |
|---|---|---|
| 2007-04-24 | Authored | Jassal B |
| 2007-04-24 | Created | Jassal B |
| 2023-05-21 | Modified | Wright A |

### 1 submitted entities found in this pathway, mapping to 1 Reactome entities

| Input | UniProt Id |
|---|---|
| Hlcs | P50747 |

## 22. Glycerophospholipid biosynthesis (R-HSA-1483206)

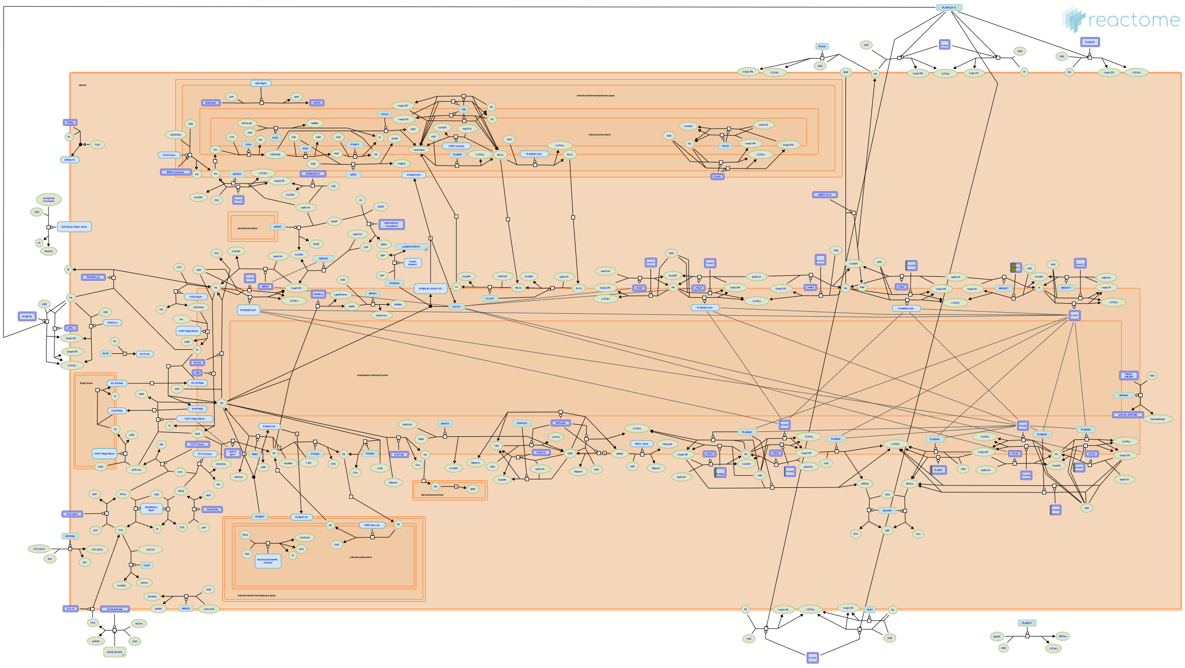

Glycerophospholipids are important structural and functional components of biological membranes and constituents of serum lipoproteins and the pulmonary surfactant. In addition, glycerophospholipids act as precursors of lipid mediators such as platelet-activating factor and eicosanoids. Cellular membranes contains a distinct composition of various glycerophospholipids such as phosphatidic acid (PA), phosphatidylcholine (PC), phosphatidylethanolamine (PE), phosphatidylserine (PS), phosphatidylglycerol (PG), phosphatidylinositol (PI), cardiolipin (CL), lysophosphatidic acid (LPA) and lysobisphosphatidic acid (also known as bis(monoacylglycerol) hydrogen phosphate - BMP).

Glycerophospholipids are first formed by the *de novo* (Kennedy) pathway using fatty acids activated as acyl-CoA donors. However, the acyl groups of glycerophospholipids are highly diverse and distributed in an asymmetric manner. Saturated and monounsaturated fatty acids are usually esterified at the *sn-1* position, whereas polyunsaturated acyl groups are esterified at the *sn-2* position. Subsequent acyl chain remodeling (Lands cycle) generates the diverse glycerophospholipid composition and asymmetry characteristic of cell membranes.

In the *de novo* pathway of glycerophospholipid biosynthesis, lysophosphatidic acid (LPA) is initially formed from glycerol 3-phosphate (G3P). Next, LPA is converted to PA by a LPA acyltransferase (AGPAT, also known as LPAAT), then PA is metabolized into two types of glycerol derivatives. The first is diacylglycerol (DAG) which is converted to triacylglycerol (TAG), PC, and PE. Subsequently, PS is synthesized from PC or PE. The second is cytidine diphosphate-diacylglycerol (CDP-DAG), which is processed into PI, PG, CL, and BMP. Each glycerophospholipid is involved in acyl chain remodeling via cleavage by phospholipases followed by reacylation by an acyltransferase.

Most of the glycerophospholipids are synthesized at the endoplasmic reticulum (ER), however, some, most notably cardiolipin, and BMP are synthesized in the mitochondrial and endosomal membranes respectively. Since the most of the glycerophospholipids are found in all membrane compartments, there must be extensive network of transport of glycerophospholipids from one membrane compartment to another via various mechanisms including diffusion through the cytosol, formation of transportation complexes, and diffusion via membrane contact sites (MCS) (Osman et al. 2011, Lebiedzinska et al. 2009, Lev 2010, Scherer & Schmitz 2011, Orso et al. 2011, Hermansson et al. 2011, Vance & Vance 2008).

## References


Jones AW, Lebiedzinska M, Duszynski J, Szabadkai G & Wieckowski MR (2009). Interactions between the endoplasmic reticulum, mitochondria, plasma membrane and other subcellular organelles. Int J Biochem Cell Biol, 41, 1805-16.

Schmitz G, Grandl M & Orsó E (2011). Oxidized LDL-induced endolysosomal phospholipidosis and enzymatically modified LDL-induced foam cell formation determine specific lipid species modulation in human macrophages. Chem Phys Lipids.

Schmitz G & Scherer M (2011). Metabolism, function and mass spectrometric analysis of bis(monoacylglycero)phosphate and cardiolipin. Chem Phys Lipids.

Vance JE & Vance DE (2008). *Phospholipid biosynthesis in eukaryotes, Biochemistry of Lipids, Lipoproteins and Membranes, 5th Edition* , 213-244.

Lev S (2010). Non-vesicular lipid transport by lipid-transfer proteins and beyond. Nat Rev Mol Cell Biol, 11, 739-50.


## Edit history

| Date | Action | Author |
| --- | --- | --- |
| 2011-08-12 | Edited | Williams MG |
| 2011-08-12 | Created | Williams MG |
| 2011-09-14 | Authored | Williams MG |
| 2023-05-21 | Modified | Wright A |

## 1 submitted entities found in this pathway, mapping to 1 Reactome entities

| Input | UniProt Id |
| --- | --- |
| Pla2g4e | Q3MJ16 |

## 23. Metabolism of vitamins and cofactors (R-HSA-196854)

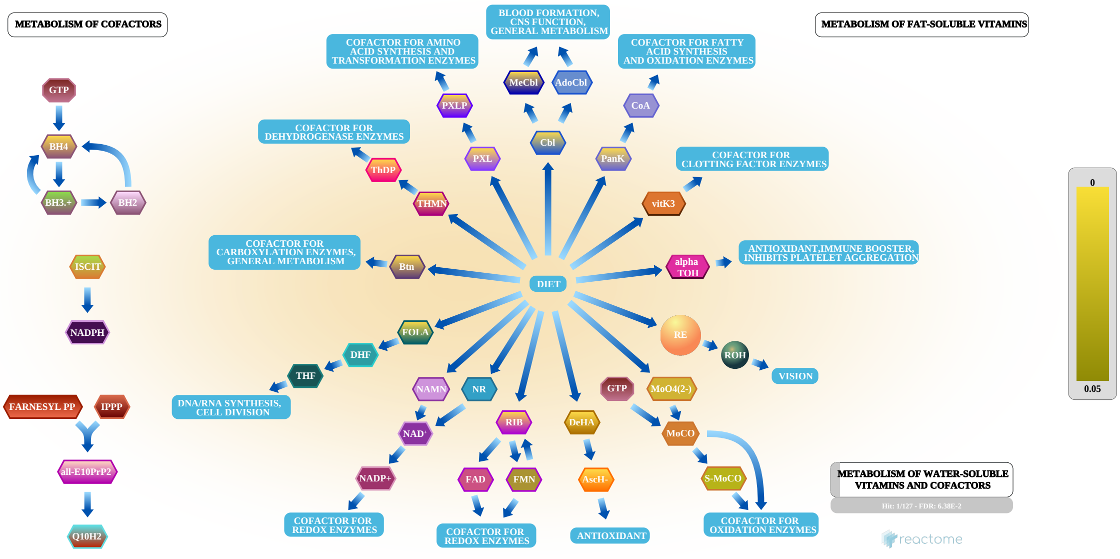


Vitamins are a diverse group of organic compounds, classified according to their solubility, either fat-soluble or water-soluble, that are either not synthesized or synthesized only in limited amounts by human cells. They are required in small amounts in the diet and have distinct biochemical roles, often as coenzymes (cofactors). The physiological processes dependent on vitamin-requiring reactions include many aspects of intermediary metabolism, vision, bone formation, and blood coagulation, and vitamin deficiencies are associated with a correspondingly diverse and severe group of diseases. Metabolic pathways for water-soluble B group and C vitamins, and for fat-soluble vitamins A, D and K are annotated in Reactome, covering processes that convert dietary forms of these molecules into active forms, and that regenerate active forms of vitamin cofactors consumed in other metabolic processes.

### References

### Edit history

| Date | Action | Author |
|---|---|---|
| 2007-04-24 | Authored | Jassal B |
| 2007-04-24 | Created | Jassal B |
| 2023-05-21 | Modified | Wright A |

### 1 submitted entities found in this pathway, mapping to 1 Reactome entities

| Input | UniProt Id |
|---|---|
| Hlcs | P50747 |

## 24. TCF dependent signaling in response to WNT (R-HSA-201681)

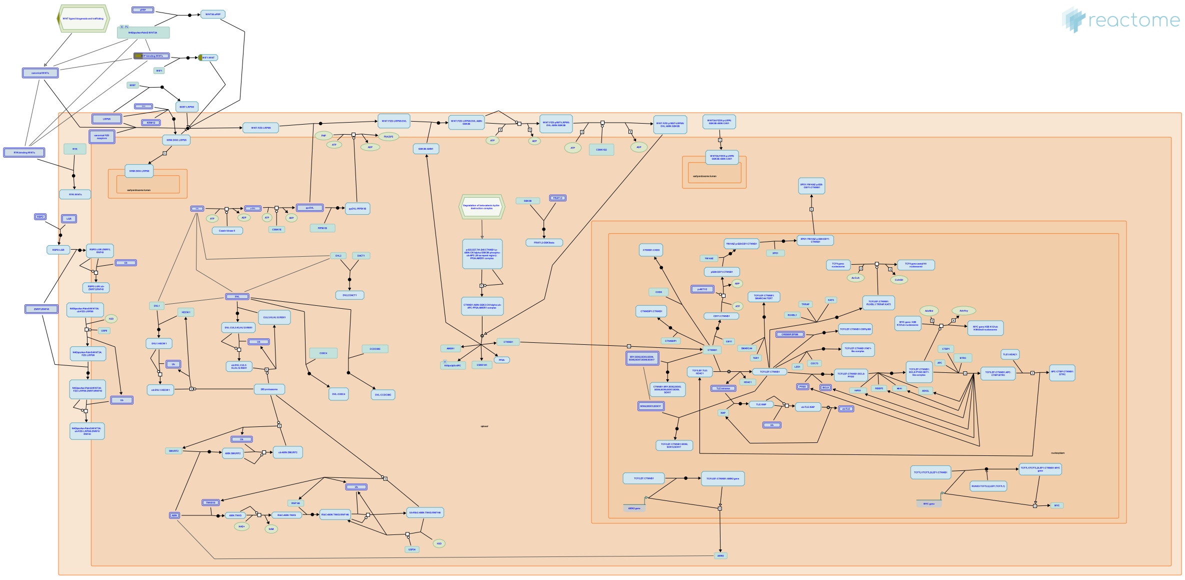

**Cellular compartments:** cytosol, extracellular region, nucleoplasm.

19 WNT ligands and 10 FZD receptors have been identified in human cells; interactions amongst these ligands and receptors vary in a developmental and tissue-specific manner and lead to activation of so-called 'canonical' and 'non-canonical' WNT signaling. In the canonical WNT signaling pathway, binding of a WNT ligand to the Frizzled (FZD) and lipoprotein receptor-related protein (LRP) receptors results in the inactivation of the destruction complex, the stabilization and nuclear translocation of beta-catenin and subsequent activation of T-cell factor/lymphoid enhancing factor (TCF/LEF)-dependent transcription. Transcriptional activation in response to canonical WNT signaling controls processes such as cell fate, proliferation and self renewal of stem cells, as well as contributing to oncogenesis (reviewed in MacDonald et al, 2009; Saito-Diaz et al, 2013; Kim et al, 2013).

### References


MacDonald BT, Tamai K & He X (2009). Wnt/beta-catenin signaling: components, mechanisms, and diseases. Dev Cell, 17, 9-26.

Wang X, Wallace HA, Page-McCaw A, Lee E, Thorne CA, Chen TW & Saito-Diaz K (2013). The way Wnt works: Components and mechanism. Growth Factors, 31, 1-31.

Jho EH, Kim W & Kim M (2013). Wnt/?-catenin signalling: from plasma membrane to nucleus. Biochem. J., 450, 9-21.


### Edit history

| Date | Action | Author |
|---|---|---|
| 2007-09-04 | Edited | Matthews L |
| 2007-09-11 | Created | Matthews L |
| 2013-08-24 | Authored | Rothfels K |
| 2013-10-03 | Edited | Gillespie ME |
| 2014-01-22 | Reviewed | Rajakulendran N |

| Date | Action | Author |
|---|---|---|
| 2014-02-15 | Reviewed | van Amerongen R |
| 2014-04-22 | Reviewed | Kikuchi A |
| 2023-05-30 | Modified | Wright A |

## 1 submitted entities found in this pathway, mapping to 1 Reactome entities

| Input | UniProt Id |
|---|---|
| Wnt9a | O14904 |

## 25. Signaling by NOTCH (R-HSA-157118)

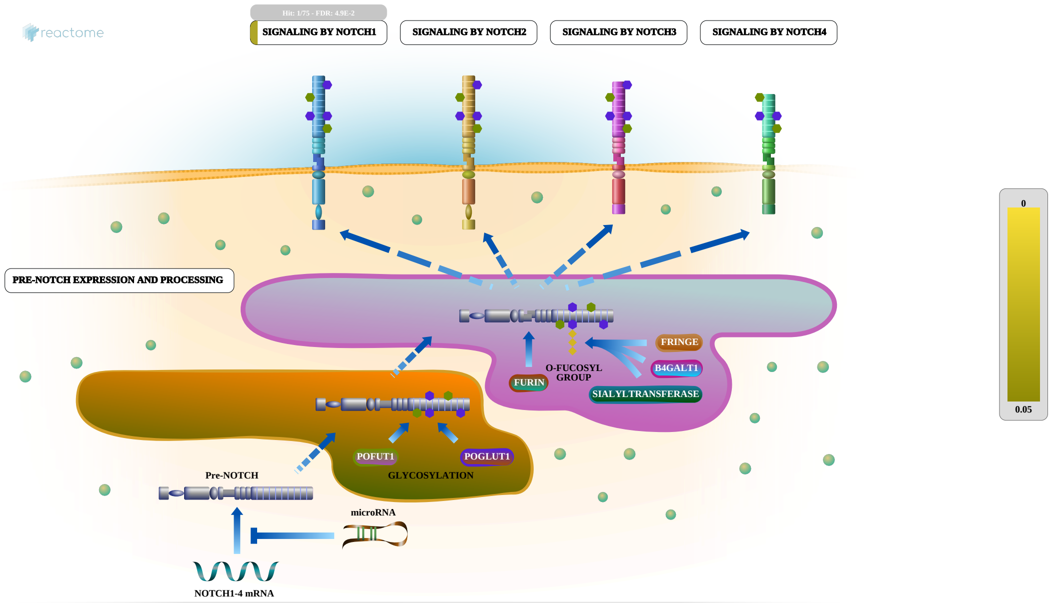


The Notch Signaling Pathway (NSP) is a highly conserved pathway for cell-cell communication. NSP is involved in the regulation of cellular differentiation, proliferation, and specification. For example, it is utilised by continually renewing adult tissues such as blood, skin, and gut epithelium not only to maintain stem cells in a proliferative, pluripotent, and undifferentiated state but also to direct the cellular progeny to adopt different developmental cell fates. Analogously, it is used during embryonic development to create fine-grained patterns of differentiated cells, notably during neurogenesis where the NSP controls patches such as that of the vertebrate inner ear where individual hair cells are surrounded by supporting cells.

This process is known as lateral inhibition: a molecular mechanism whereby individual cells within a field are stochastically selected to adopt particular cell fates and the NSP inhibits their direct neighbours from doing the same. The NSP has been adopted by several other biological systems for binary cell fate choice. In addition, the NSP is also used during vertebrate segmentation to divide the growing embryo into regular blocks called somites which eventually form the vertebrae. The core of this process relies on regular pulses of Notch signaling generated from a molecular oscillator in the presomatic mesoderm.

The Notch receptor is synthesized in the rough endoplasmic reticulum as a single polypeptide precursor. Newly synthesized Notch receptor is proteolytically cleaved in the trans-golgi network, creating a heterodimeric mature receptor comprising of non-covalently associated extracellular and transmembrane subunits. This assembly travels to the cell surface ready to interact with specific ligands. Following ligand activation and further proteolytic cleavage, an intracellular domain is released and translocates to the nucleus where it regulates gene expression.

### References

### Edit history

| Date | Action | Author |
|---|---|---|
| 2004-12-15 | Reviewed | Joutel A |
| 2004-12-15 | Authored | Jassal B |

| Date | Action | Author |
| --- | --- | --- |
| 2004-12-15 | Created | Jassal B |
| 2023-05-21 | Modified | Wright A |

## 1 submitted entities found in this pathway, mapping to 1 Reactome entities

| Input | UniProt Id |
| --- | --- |
| Hdac9 | Q9UKV0 |

# 6. Identifiers found

Below is a list of the input identifiers that have been found or mapped to an equivalent element in Reactome, classified by resource.

### 4 of the submitted entities were found, mapping to 4 Reactome entities

| Input | UniProt Id | Input | UniProt Id |
|---|---|---|---|
| Hdac9 | Q9UKV0 | Hlcs | P50747 |
| Pla2g4e | Q3MJ16 | Wnt9a | O14904 |

# 7. Identifiers not found

These 2 identifiers were not found neither mapped to any entity in Reactome.

| | |
|---|---|
| 4933407I18Rik | Shisa9 |
