# Supplementary material for: Sex- and region-specific cortical and hippocampal whole genome transcriptome profiles from control and APP/PS1 Alzheimer’s disease mice
Source: PLoS One. 2024 Feb 7;19(2):e0296959. doi: 10.1371/journal.pone.0296959 (PMC10849391; doi:10.1371/journal.pone.0296959)
Supplement: S1 File — S1 Fig: Genotyping of APP/PS1 AD mice and WT control animals. S2 Fig: 3D image of the murine brain including the RS cortex and hippocampus (BROIs) used for transcriptome analysis in our study. S3 Fig: PCA of transcriptomes from the RS cortex and hippocampus of WT controls and APP/PS1 AD mice of both sexes. S4 Fig: Hierarchical clustering of transcriptome data from the RS cortex and hippocampus of WT control and APP/PS1 AD mice of both sexes. S5 Fig: Bar diagrams of the top 30 candidates of DEGs with highest significant FCs (FC > 1.5 and FC < -1.5, p < 0.05). S6 Fig: Pathway analysis of intersectional and signature gene sets in APP/PS1 subgroups. S7 Fig: Comparative qPCR analysis of selected gene transcript levels from the hippocampus of female and male APP/PS1 AD with 5XFAD mice. S1 Table: PCR reaction set-up using PCR Mastermix and genomic DNA. S2 Table: Materials used for one-color microarray-based gene expression data collection. S3 Table: Software used for one-color microarray-based gene expression data collection. S4 Table: Details on genes, forward and reverse primer sequences and annealing temperatures relevant for qPCR experimentation. S5 Table: Characteristics of DEGs in the RS cortex of female APP/PS1 AD mice. S6 Table: Characteristics of DEGs in the hippocampus of female APP/PS1 AD mice. S7 Table: Characteristics of DEGs in the RS cortex of male APP/PS1 AD mice. S8 Table: Characteristics of DEGs in the hippocampus of male APP/PS1 AD mice. S9 Table: Venn analysis of DEGs in the RS cortex and hippocampus of female APP/PS1 AD mice. S10 Table: Venn analysis of DEGs genes in the RS cortex and hippocampus of male APP/PS1 AD mice. S11 Table: Venn analysis of DEGs in the RS cortex of male and female APP/PS1 AD mice. S12 Table: Venn analysis of DEGs in the hippocampus of male and female APP/PS1 AD mice. S13 Table: Differentially regulated l(i)ncRNAs in APP/PS1 AD vs. WT mice. S14 Table: qPCR-based FC analysis of selected genes in the hippocampus of APP/PS1 AD vs. [file pone.0296959.s001.zip › Supplementary Files_R1/Supplementary Figure 6_Pathways_downreg genes/Signature_down_DEGs_male_Rs Cx_APPPS1/Pathway analysis report.pdf]

reactome

Pathway Analysis Report

This report contains the pathway analysis results for the submitted sample ''. Analysis was performed against Reactome version 85 on 17/08/2023. The web link to these results is:

https://reactome.org/PathwayBrowser/#/ANALYSIS=MjAyMzA4MTcwNzEyNDJfMjEyMDQ%3D

Please keep in mind that analysis results are temporarily stored on our server. The storage period depends on usage of the service but is at least 7 days. As a result, please note that this URL is only valid for a limited time period and it might have expired.

Table of Contents

1. Introduction
2. Properties
3. Genome-wide overview
4. Most significant pathways
5. Pathways details
6. Identifiers found
7. Identifiers not found

1. Introduction

Reactome is a curated database of pathways and reactions in human biology. Reactions can be considered as pathway 'steps'. Reactome defines a 'reaction' as any event in biology that changes the state of a biological molecule. Binding, activation, translocation, degradation and classical biochemical events involving a catalyst are all reactions. Information in the database is authored by expert biologists, entered and maintained by Reactome's team of curators and editorial staff. Reactome content frequently cross-references other resources e.g. NCBI, Ensembl, UniProt, KEGG (Gene and Compound), ChEBI, PubMed and GO. Orthologous reactions inferred from annotation for Homo sapiens are available for 14 non-human species including mouse, rat, chicken, puffer fish, worm, fly and yeast. Pathways are represented by simple diagrams following an SBGN-like format.

Reactome's annotated data describe reactions possible if all annotated proteins and small molecules were present and active simultaneously in a cell. By overlaying an experimental dataset on these annotations, a user can perform a pathway over-representation analysis. By overlaying quantitative expression data or time series, a user can visualize the extent of change in affected pathways and its progression. A binomial test is used to calculate the probability shown for each result, and the p-values are corrected for the multiple testing (Benjamini–Hochberg procedure) that arises from evaluating the submitted list of identifiers against every pathway.

To learn more about our Pathway Analysis, please have a look at our relevant publications:

Fabregat A, Sidiropoulos K, Garapati P, Gillespie M, Hausmann K, Haw R, … D'Eustachio P (2016). The reactome pathway knowledgebase. Nucleic Acids Research, 44(D1), D481–D487. https://doi.org/10.1093/nar/gkv1351.

Fabregat A, Sidiropoulos K, Viteri G, Forner O, Marin-Garcia P, Arnau V, … Hermjakob H (2017). Reactome pathway analysis: a high-performance in-memory approach. BMC Bioinformatics, 18.

2. Properties

- This is an **overrepresentation** analysis: A statistical (hypergeometric distribution) test that determines whether certain Reactome pathways are over-represented (enriched) in the submitted data. It answers the question 'Does my list contain more proteins for pathway X than would be expected by chance?' This test produces a probability score, which is corrected for false discovery rate using the Benjamani-Hochberg method.
- 1 out of 4 identifiers in the sample were found in Reactome, where 9 pathways were hit by at least one of them.
- All non-human identifiers have been converted to their human equivalent.
- This report is filtered to show only results for species 'Homo sapiens' and resource 'UniProt'.
- The unique ID for this analysis (token) is MjAyMzA4MTcwNzEyNDJfMjEyMDQ%3D. This ID is valid for at least 7 days in Reactome's server. Use it to access Reactome services with your data.

3. Genome-wide overview

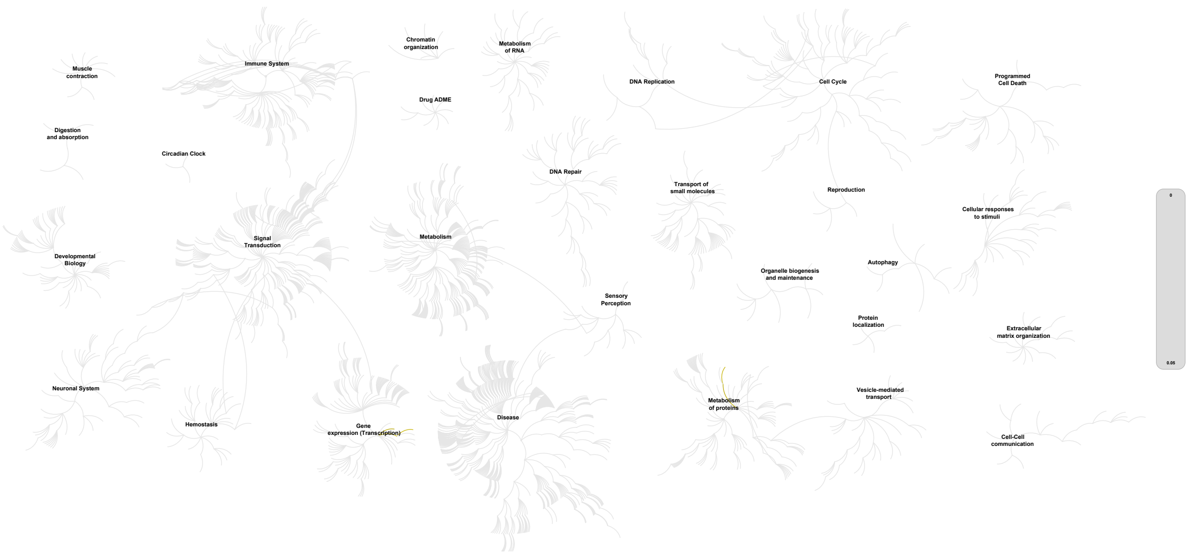

This figure shows a genome-wide overview of the results of your pathway analysis. Reactome pathways are arranged in a hierarchy. The center of each of the circular "bursts" is the root of one top-level pathway, for example "DNA Repair". Each step away from the center represents the next level lower in the pathway hierarchy. The color code denotes over-representation of that pathway in your input dataset. Light grey signifies pathways which are not significantly over-represented.

4. Most significant pathways

The following table shows the 9 most relevant pathways sorted by p-value.

Pathway name	Entities				Reactions	
	found	ratio	p-value	FDR*	found	ratio
RNA Polymerase II Transcription Elongation	1 / 58	0.005	0.02	0.028	4 / 8	5.59e-04
Formation of RNA Pol II elongation complex	1 / 58	0.005	0.02	0.028	1 / 2	1.40e-04
E3 ubiquitin ligases ubiquitinate target proteins	1 / 59	0.005	0.02	0.028	2 / 16	0.001
Protein ubiquitination	1 / 79	0.007	0.027	0.028	2 / 32	0.002
RNA Polymerase II Pre-transcription Events	1 / 81	0.007	0.028	0.028	6 / 17	0.001
RNA Polymerase II Transcription	1 / 1,396	0.12	0.401	0.401	10 / 945	0.066
Post-translational protein modification	1 / 1,429	0.123	0.408	0.408	2 / 538	0.038
Gene expression (Transcription)	1 / 1,568	0.135	0.44	0.44	10 / 1,090	0.076
Metabolism of proteins	1 / 1,949	0.168	0.52	0.52	2 / 812	0.057

* False Discovery Rate

5. Pathways details

For every pathway of the most significant pathways, we present its diagram, as well as a short summary, its bibliography and the list of inputs found in it.

1. RNA Polymerase II Transcription Elongation (R-HSA-75955)

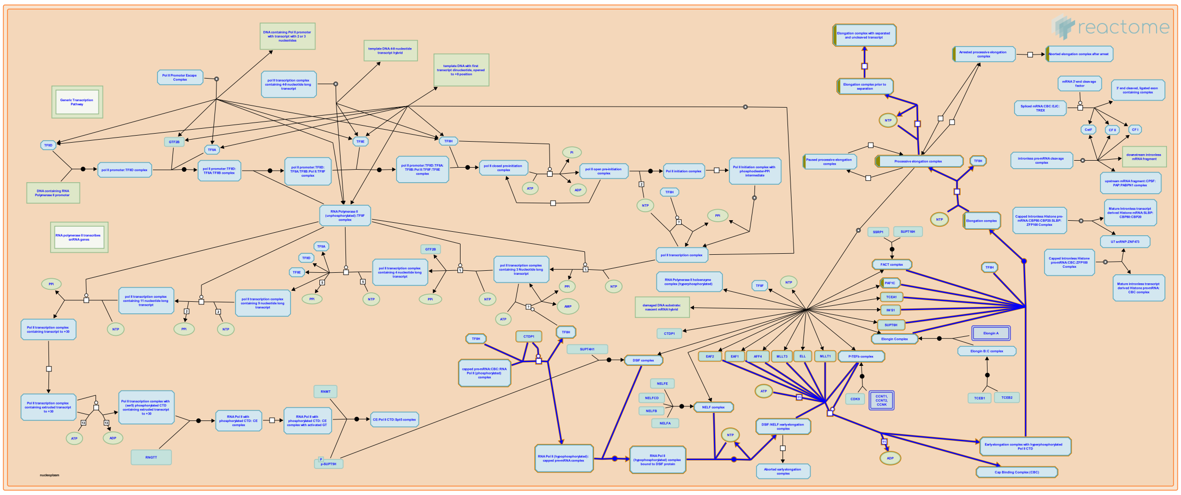

Cellular compartments: nucleoplasm.

The mechanisms governing the process of elongation during eukaryotic mRNA synthesis are being unraveled by recent studies. These studies have led to the expected discovery of a diverse collection of transcription factors that directly regulate the activities of RNA Polymerase II and unexpected discovery of roles for many elongation factors in other basic processes like DNA repair, recombination, etc. The transcription machinery and structural features of the major RNA polymerases are conserved across species. The genes active during elongation fall under different classes like, housekeeping, cell-cycle regulated, development and differentiation specific genes etc. The list of genes involved in elongation has been growing in recent times, and include: -TFIIS,DSIF, NELF, P-Tefb etc. that are involved in drug induced or sequence-dependent arrest - TFIIF, ELL, elongin, elongator etc. that are involved in increasing the catalytic rate of elongation by altering the Km and/or the Vmax of Pol II -FACT, Paf1 and other factors that are involved chromatin modification -DNA repair proteins, RNA processing and export factors, the 19S proteasome and a host of other factors like Spt5-Spt5, Paf1, and NELF complexes, FCP1P etc. (Arndt and Kane, 2003). Elongation also represents processive phase of transcription in which the activities of several mRNA processing factors are coupled to transcription through their binding to RNA polymerase (Pol II). One of the key events that enables this interaction is the differential phosphorylation of Pol II CTD. Phosphorylation pattern of CTD changes during transcription, most significantly at the beginning and during elongation process. TFIIH-dependent Ser5 phosphorylation is observed primarily at promoter regions while P-Tefb mediated Ser2 phosphorylation is seen mainly in the coding regions, during elongation. Experimental evidence suggests a dynamic association of RNA processing factors with differently modified forms of the polymerase during the transcription cycle. (Komarnitsky et al., 2000). [Komarnitsky et al 2000, Arndt & Kane 2003, Shilatifard et al 2003]

References

Buratowski S, Cho EJ & Komarnitsky P (2000). Different phosphorylated forms of RNA polymerase II and associated mRNA processing factors during transcription. Genes Dev, 14, 2452-60.

Conaway JW, Shilatifard A & Conaway RC (2003). The RNA polymerase II elongation complex. Annu Rev Biochem, 72, 693-715.

Kane CM & Arndt KM (2003). Running with RNA polymerase: eukaryotic transcript elongation. Trends Genet, 19, 543-50.

Edit history

Date	Action	Author
2003-09-11	Authored	Conaway JW, Conaway RC
2003-09-11	Created	Conaway JW, Conaway RC
2023-05-19	Edited	Joshi-Tope G
2023-05-21	Modified	Wright A

1 submitted entities found in this pathway, mapping to 1 Reactome entities

Input	UniProt Id
Ctr9	Q6PD62

2. Formation of RNA Pol II elongation complex (R-HSA-112382)

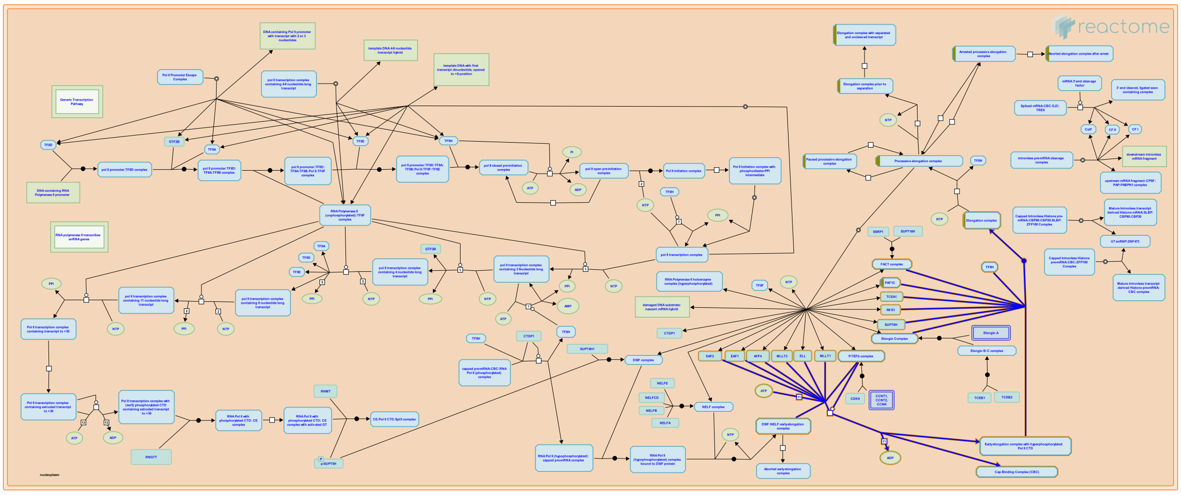

Cellular compartments: nucleoplasm.

TFIIS is a transcription factor involved in different phases of transcription, occurring in a major ubiquitous form and other tissue specific forms. TFIIS stimulates RNA Pol II complex out of elongation arrest.

Other transcription factors like ELL, Elongin family members and TFIIF interact directly with elongating Pol II and increase its elongation rate. These factors have been observed to act on naked DNA templates by suppressing transient pausing by the enzyme at all or most steps of nucleotide addition. In Drosophila, ELL is found at a large number of transcriptionally active sites on polytene chromosomes. In general, ELL is suspected to have more unidentified functions.

Elongin is a heterotrimeric protein complex that stimulates the overall rate of elongation. In addition, Elongin may act as an E3 Ubiquitin ligase. Ubiquitylation of RNA Pol II occurs rapidly after genotoxic assault by UV light or chemicals, and results in degradation by proteasome. The FACT complex appears to promote elongation by facilitating passage of polymerase through chromatin.

All these factors contribute to the formation of a processive elongation complex centered around the RNA Pol II complex positioned on the DNA:RNA hybrid. This enables the RNA Pol II elongation complex to function as a platform that coordinates mRNA processing and export (Reviewed by Shilatifard et al., 2003).

References

Conaway JW, Shilatifard A & Conaway RC (2003). The RNA polymerase II elongation complex. Annu Rev Biochem, 72, 693-715.

Edit history

Date	Action	Author
2003-09-11	Created	Conaway JW, Conaway RC
2004-06-22	Authored	Gopinathrao G
2023-05-21	Modified	Wright A

1 submitted entities found in this pathway, mapping to 1 Reactome entities

Input	UniProt Id
Ctr9	Q6PD62

3. E3 ubiquitin ligases ubiquitinate target proteins (R-HSA-8866654)

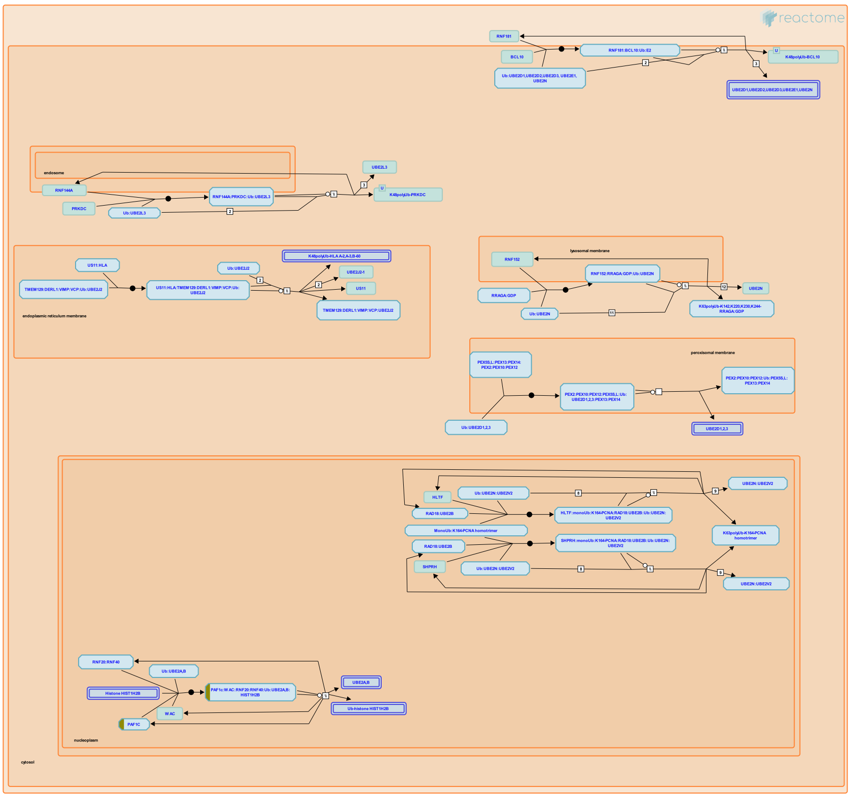

E3 ubiquitin ligases catalyze the transfer of an ubiquitin from an E2-ubiquitin conjugate to a target protein. Generally, ubiquitin is transferred via formation of an amide bond to a particular lysine residue of the target protein, but ubiquitylation of cysteine, serine and threonine residues in a few targeted proteins has also been demonstrated (reviewed in McDowell and Philpott 2013, Berndsen and Wolberger 2014). Based on protein homologies, families of E3 ubiquitin ligases have been identified that include RING-type ligases (reviewed in Deshaies et al. 2009, Metzger et al. 2012, Metzger et al. 2014), HECT-type ligases (reviewed in Rotin et al. 2009, Metzger et al. 2012), and RBR-type ligases (reviewed in Dove et al. 2016). A subset of the RING-type ligases participate in CULLIN-RING ligase complexes (CRLs which include SCF complexes, reviewed in Lee and Zhou 2007, Genschik et al. 2013, Skaar et al. 2013, Lee et al. 2014).

Some E3-E2 combinations catalyze mono-ubiquitination of the target protein (reviewed in Nakagawa and Nakayama 2015). Other E3-E2 combinations catalyze conjugation of further ubiquitin monomers to the initial ubiquitin, forming polyubiquitin chains. (It may also be possible for some E3-E2 combinations to preassemble polyubiquitin and transfer it as a unit to the target protein.) Ubiquitin contains several lysine (K) residues and a free alpha amino group to which further ubiquitin can be conjugated. Thus different types of polyubiquitin are possible: K11 linked polyubiquitin is observed in endoplasmic reticulum-associated degradation (ERAD), K29 linked polyubiquitin is observed in lysosomal degradation, K48 linked polyubiquitin directs target proteins to the proteasome for degradation, whereas K63 linked polyubiquitin generally acts as a scaffold to recruit other proteins in several cellular processes, notably DNA repair (reviewed in Komander et al. 2009).

References

Lee J & Zhou P (2007). DCAFs, the missing link of the CUL4-DDB1 ubiquitin ligase. Mol Cell, 26, 775-80.

Lechner E, Sumara I & Genschik P (2013). The emerging family of CULLIN3-RING ubiquitin ligases (CRL3s): cellular functions and disease implications. EMBO J., 32, 2307-20.

Klevit RE, Rittinger K, Dove KK, Stieglitz B & Duncan ED (2016). Molecular insights into RBR E3 ligase ubiquitin transfer mechanisms. EMBO Rep., 17, 1221-35.

Wolberger C & Berndsen CE (2014). New insights into ubiquitin E3 ligase mechanism. Nat. Struct. Mol. Biol., 21, 301-7.

Komander D (2009). The emerging complexity of protein ubiquitination. Biochem. Soc. Trans., 37, 937-53.

Edit history

Date	Action	Author
2016-04-02	Edited	May B
2016-04-02	Authored	May B
2016-04-02	Created	May B
2016-11-03	Reviewed	Azevedo JE
2023-05-30	Modified	Wright A

1 submitted entities found in this pathway, mapping to 1 Reactome entities

Input	UniProt Id
Ctr9	Q6PD62

4. Protein ubiquitination (R-HSA-8852135)

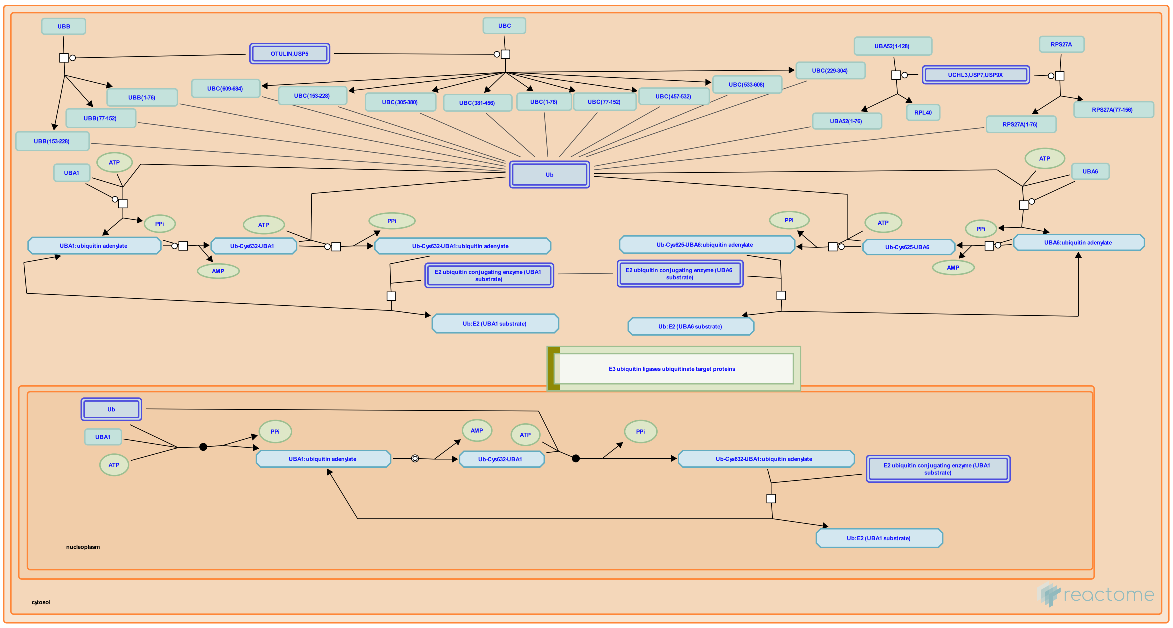

Ubiquitin is a small, 76 amino acid residue protein that is conjugated by E3 ubiquitin ligases to other proteins in order to regulate their function or degradation (enzymatic cascade reviewed in Neutzner and Neutzner 2012, Kleiger and Mayor 2014, structures and mechanisms of conjugating enzymes reviewed in Lorenz et al. 2013). Ubiquitination of target proteins usually occurs between the C-terminal glycine residue of ubiquitin and a lysine residue of the target, although linkages with cysteine, serine, and threonine residues are also observed (reviewed in Wang et al. 2012, McDowell and Philpott 2013).

Ubiquitin must first be processed from larger precursors and then activated by formation of a thiol ester bond between ubiquitin and an E1 activating enzyme (UBA1 or UBA6) and transfer to an E2 conjugating enzyme before being transferred by an E3 ligase to a target protein. Precursor proteins containing multiple ubiquitin monomers (polyubiquitins) are produced from the UBB and UBC genes; precursors containing a single ubiquitin monomer and a ribosomal protein are produced from the UBA52 and RPS27A genes. Many proteases (deubiquitinases) may potentially process these precursors yielding monomeric ubiquitin. The proteases OTULIN and USP5 are particularly active in cleaving the polyubiquitin precursors, whereas the proteases UCHL3, USP7, and USP9X cleave the ubiquitin-ribosomal protein precursors yielding ubiquitin monomers (Grou et al. 2015). A resultant ubiquitin monomer is activated by adenylation of the C-terminal glycine followed by conjugation of the C-terminus to a cysteine residue of the E1 enzymes UBA1 or UBA6 via a thiol ester bond. The ubiquitin is then transferred from the E1 enzyme to a cysteine residue of one of several E2 enzymes (reviewed in van Wijk and Timmers 2010, Stewart et al. 2016). Through a less well characterized mechanism, E3 ubiquitin ligases then bring a target protein and the E2-ubiquitin conjugate into proximity so that the ubiquitin is transferred via formation of an amide bond to a particular lysine residue (or, in rarer cases, a thiol ester bond to a cysteine residue or an ester bond to a serine or threonine residue) of the target protein (reviewed in Berndsen and Wolberger 2014). Based on protein homologies, families of E3 ubiquitin ligases have been identified that include RING-type ligases (reviewed in Deshaies et al. 2009, Metzger et al. 2012, Metzger et al. 2014), HECT-type ligases (reviewed in Rotin et al. 2009, Metzger et al. 2012), and RBR-type ligases (reviewed in Dove et al. 2016). A subset of the RING-type ligases participate in CULLIN-RING ligase complexes (CRLs which include SCF complexes, reviewed in Lee and Zhou 2007, Genschik et al. 2013, Skaar et al. 2013, Lee et al. 2014).

Some E3-E2 combinations catalyze mono-ubiquitination of the target protein (reviewed in Nakagawa and Nakayama 2015). Other E3-E2 combinations catalyze conjugation of further ubiquitin monomers to the initial ubiquitin, forming polyubiquitin chains. (It may also be possible for some E3-E2 combinations to preassemble polyubiquitin and transfer it as a unit to the target protein.) Ubiquitin contains several lysine (K) residues and a free alpha amino group to which further ubiquitin can be conjugated. Thus different types of polyubiquitin are possible: K11 linked polyubiquitin is observed in endoplasmic reticulum-associated degradation (ERAD), K29 linked polyubiquitin is observed in lysosomal degradation, K48 linked polyubiquitin directs target proteins to the proteasome for degradation, whereas K63 linked polyubiquitin generally acts as a scaffold to recruit other proteins in several cellular processes, notably DNA repair (reviewed in Komander et al. 2009). Ubiquitination is highly regulated (reviewed in Vittal et al. 2015) and affects all cellular processes including DNA damage response (reviewed in Brown and Jackson 2015), immune signaling (reviewed in Park et al. 2014, Lutz-Nicoladoni et al. 2015), and regulation of normal and cancerous cell growth (reviewed in Skaar and Pagano 2009, Yerlikaya and Yontem 2013, Strikoudis et al. 2014).

References

Brown JS & Jackson SP (2015). Ubiquitylation, neddylation and the DNA damage response. Open Biol, 5, 150018.

Lutz-Nicoladoni C, Wolf D & Sopper S (2015). Modulation of Immune Cell Functions by the E3 Ligase Cbl-b. Front Oncol, 5, 58.

Lee J & Zhou P (2007). DCAFs, the missing link of the CUL4-DDB1 ubiquitin ligase. Mol Cell, 26, 775-80.

Lechner E, Sumara I & Genschik P (2013). The emerging family of CULLIN3-RING ubiquitin ligases (CRL3s): cellular functions and disease implications. EMBO J., 32, 2307-20.

Klevit RE, Brzovic PS, Vittal V & Stewart MD (2015). Regulating the Regulators: Recent Revelations in the Control of E3 Ubiquitin Ligases. J. Biol. Chem., 290, 21244-51.

Edit history

Date	Action	Author
2016-01-12	Edited	May B
2016-01-12	Authored	May B
2016-01-13	Created	May B
2016-08-11	Reviewed	Azevedo JE
2023-05-21	Modified	Wright A

1 submitted entities found in this pathway, mapping to 1 Reactome entities

Input	UniProt Id
Ctr9	Q6PD62

5. RNA Polymerase II Pre-transcription Events (R-HSA-674695)

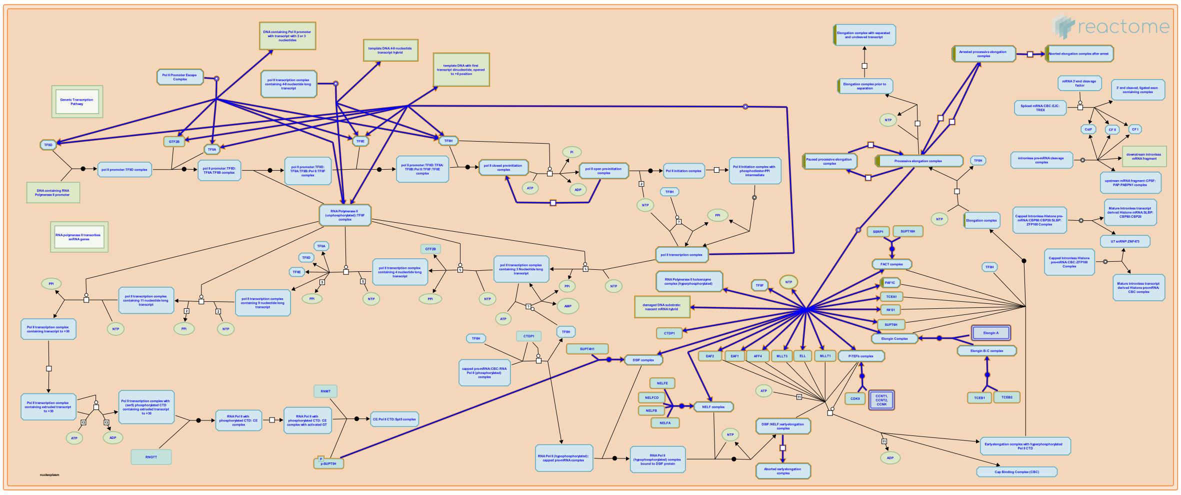

Cellular compartments: nucleoplasm.

For initiation, Pol II assembles with the general transcription factors TFIIB, TFIID, TFIIE, TFIIF and TFIIH, which are collectively known as the general transcription factors, at promoter DNA to form the pre-initiation complex (PIC). Until the nascent transcript is about 15 nucleotides long, the early transcribing complex is functionally unstable. In the beginning, short RNAs are frequently released and Pol II has to restart transcription (abortive cycling). There is a decline in the level of abortive transcription when the RNA reaches a length of about four nucleotides, and this transition is termed escape commitment

References

Cramer P (2004). Structure and function of RNA polymerase II. Adv. Protein Chem., 67, 1-42.

Edit history

Date	Action	Author
2010-05-05	Created	Gillespie ME
2023-05-21	Modified	Wright A

1 submitted entities found in this pathway, mapping to 1 Reactome entities

Input	UniProt Id
Ctr9	Q6PD62

6. RNA Polymerase II Transcription (R-HSA-73857)

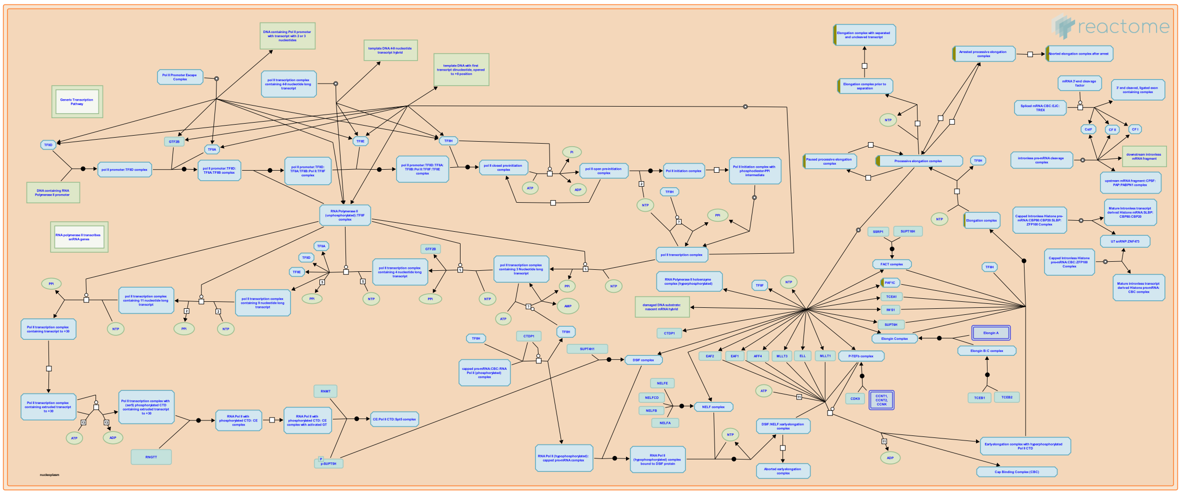

Cellular compartments: nucleoplasm.

RNA polymerase II (Pol II) is the central enzyme that catalyses DNA- directed mRNA synthesis during the transcription of protein-coding genes. Pol II consists of a 10-subunit catalytic core, which alone is capable of elongating the RNA transcript, and a complex of two subunits, Rpb4/7, that is required for transcription initiation.

The transcription cycle is divided in three major phases: initiation, elongation, and termination. Transcription initiation include promoter DNA binding, DNA melting, and initial synthesis of short RNA transcripts. The transition from initiation to elongation, is referred to as promoter escape and leads to a stable elongation complex that is characterized by an open DNA region or transcription bubble. The bubble contains the DNA-RNA hybrid, a heteroduplex of eight to nine base pairs. The growing 3-end of the RNA is engaged with the polymerase complex active site. Ultimately transcription terminates and Pol II dissociates from the template.

References

Cramer P (2004). Structure and function of RNA polymerase II. Adv. Protein Chem., 67, 1-42.

Edit history

Date	Action	Author
2005-01-12	Created	Reinberg D, Timmers HTM, Conaway JW, Proudfoot NJ, Conaway RC
2023-05-21	Modified	Wright A

1 submitted entities found in this pathway, mapping to 1 Reactome entities

Input	UniProt Id
Ctr9	Q6PD62

7. Post-translational protein modification (R-HSA-597592)

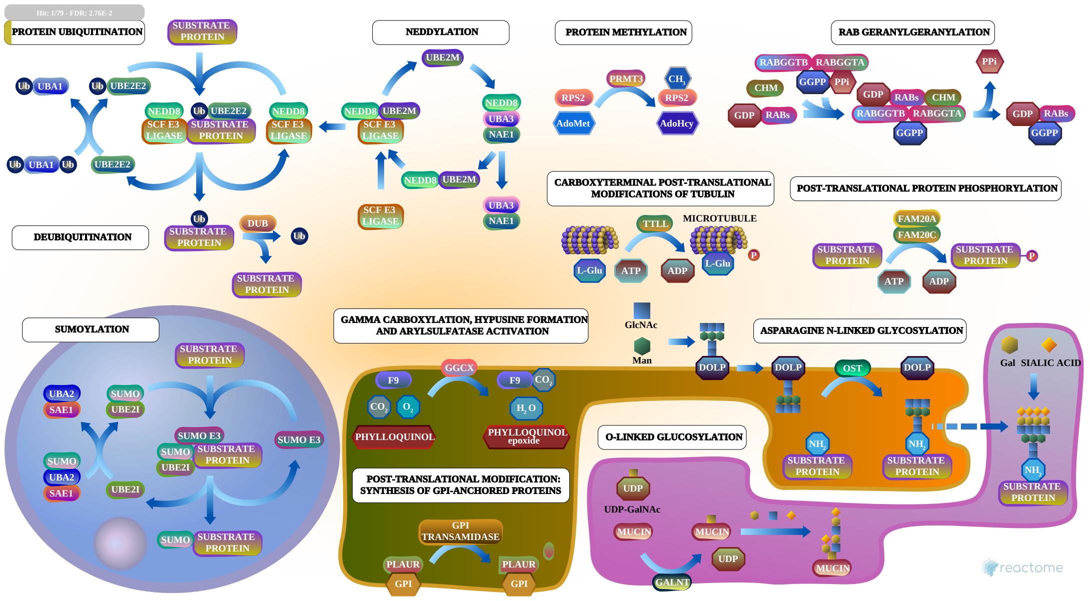

After translation, many newly formed proteins undergo further covalent modifications that alter their functional properties. Modifications associated with protein localization include the attachment of oligosaccharide moieties to membrane-bound and secreted proteins (**N-linked** and **O-linked glycosylation**), the attachment of lipid (**RAB geranylgeranylation**) or glycolipid moieties (**GPI-anchored proteins**) that anchor proteins to cellular membranes, and the vitamin K-dependent attachment of carboxyl groups to glutamate residues. Modifications associated with functions of specific proteins include **gamma carboxylation** of clotting factors, **hypusine formation** on eukaryotic translation initiation factor 5A, conversion of a cysteine residue to formylglycine (**arylsulfatase activation**), methylation of lysine and arginine residues on non-histone proteins (**protein methylation**), **protein phosphorylation** by secretory pathway kinases, and **carboxyterminal modifications of tubulin** involving the addition of polyglutamate chains.

Protein ubiquitination and **deubiquitination** play a major role in regulating protein stability and, together with **SUMOylation** and **neddylation**, can modulate protein function as well.

References

Edit history

Date	Action	Author
2005-04-18	Authored	D'Eustachio P
2010-04-14	Created	D'Eustachio P
2023-05-19	Edited	D'Eustachio P
2023-05-19	Reviewed	Stafford DW, Orlean P
2023-05-21	Modified	Wright A

1 submitted entities found in this pathway, mapping to 1 Reactome entities

Input	UniProt Id
Ctr9	Q6PD62

Input	UniProt Id

8. Gene expression (Transcription) (R-HSA-74160)

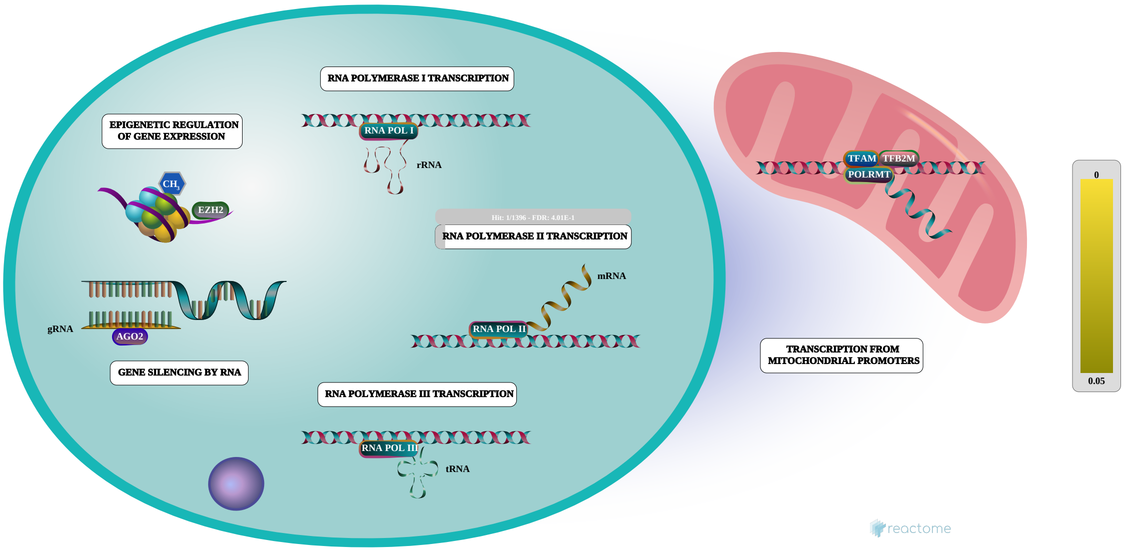

Gene expression encompasses transcription and translation and the regulation of these processes. RNA Polymerase I Transcription produces the large preribosomal RNA transcript (45S pre-rRNA) that is processed to yield 18S rRNA, 28S rRNA, and 5.8S rRNA, accounting for about half the RNA in a cell. RNA Polymerase II transcription produces messenger RNAs (mRNA) as well as a subset of non-coding RNAs including many small nucleolar RNAs (snRNA) and microRNAs (miRNA). RNA Polymerase III Transcription produces transfer RNAs (tRNA), 5S RNA, 7SL RNA, and U6 snRNA. Transcription from mitochondrial promoters is performed by the mitochondrial RNA polymerase, POLRMT, to yield long transcripts from each DNA strand that are processed to yield 12S rRNA, 16S rRNA, tRNAs, and a few RNAs encoding components of the electron transport chain. Regulation of gene expression can be divided into epigenetic regulation, transcriptional regulation, and post-transcription regulation (comprising translational efficiency and RNA stability). Epigenetic regulation of gene expression is the result of heritable chemical modifications to DNA and DNA-binding proteins such as histones. Epigenetic changes result in altered chromatin complexes that influence transcription. Gene Silencing by RNA mostly occurs post-transcriptionally but can also affect transcription. Small RNAs originating from the genome (miRNAs) or from exogenous RNA (siRNAs) are processed and transferred to the RNA-induced silencing complex (RISC), which interacts with complementary RNA to cause cleavage, translational inhibition, or transcriptional inhibition.

References

Edit history

Date	Action	Author
2003-09-11	Authored	Larsson NG, Gustafsson CM, Comai L, Reinberg D, Timmers HTM et al.
2003-09-11	Created	Proudfoot NJ, Kornblihtt AR
2008-12-03	Authored	Caudy M, Proudfoot NJ, Kornblihtt AR, D'Eustachio P
2016-12-29	Revised	D'Eustachio P
2023-05-19	Edited	Joshi-Tope G

Date	Action	Author
2023-05-19	Reviewed	Paule M, Willis I, Zhao X
2023-05-21	Modified	Wright A

1 submitted entities found in this pathway, mapping to 1 Reactome entities

Input	UniProt Id
Ctr9	Q6PD62

9. Metabolism of proteins (R-HSA-392499)

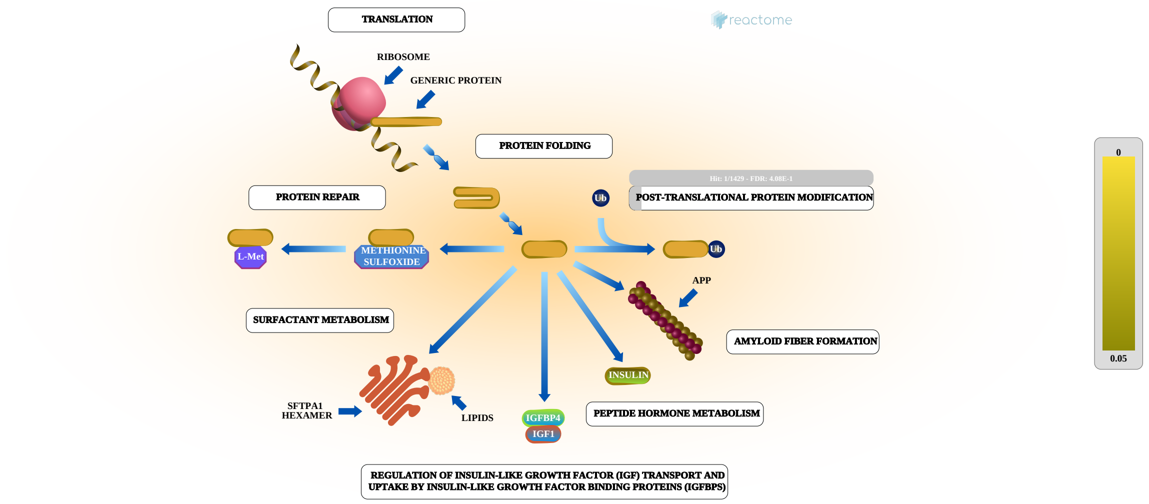

Metabolism of proteins, as annotated here, covers the full life cycle of a protein from its synthesis to its posttranslational modification and degradation, at various levels of specificity. Protein synthesis is accomplished through the process of Translation of an mRNA sequence into a polypeptide chain. Protein folding is achieved through the function of molecular chaperones which recognize and associate with proteins in their non-native state and facilitate their folding by stabilizing the conformation of productive folding intermediates (Young et al. 2004). Following translation, many newly formed proteins undergo Post-translational protein modification, essentially irreversible covalent modifications critical for their mature locations and functions (Knorre et al. 2009), including gamma carboxylation, synthesis of GPI-anchored proteins, asparagine N-linked glycosylation, O-glycosylation, SUMOylation, ubiquitination, deubiquitination, RAB geranylgeranylation, methylation, carboxyterminal post-translational modifications, neddylation, and phosphorylation. Peptide hormones are synthesized as parts of larger precursor proteins whose cleavage in the secretory system (endoplasmic reticulum, Golgi apparatus, secretory granules) is annotated in Peptide hormone metabolism. After secretion, peptide hormones are modified and degraded by extracellular proteases (Chertow, 1981 PMID:6117463). Protein repair enables the reversal of damage to some amino acid side chains caused by reactive oxygen species. Pulmonary surfactants are lipids and proteins that are secreted by the alveolar cells of the lung that decrease surface tension at the air/liquid interface within the alveoli to maintain the stability of pulmonary tissue (Agassandian and Mallampalli 2013). Nuclear regulation, transport, metabolism, reutilization, and degradation of surfactant are described in the Surfactant metabolism pathway. Amyloid fiber formation, the accumulation of mostly extracellular deposits of fibrillar proteins, is associated with tissue damage observed in numerous diseases including late phase heart failure (cardiomyopathy) and neurodegenerative diseases such as Alzheimer's, Parkinson's, and Huntington's.

References

Edit history

Date	Action	Author
2009-03-04	Edited	Matthews L

Date	Action	Author
2009-03-05	Authored	Matthews L
2009-03-05	Created	Matthews L
2023-05-21	Modified	Wright A

1 submitted entities found in this pathway, mapping to 1 Reactome entities

Input	UniProt Id
Ctr9	Q6PD62

6. Identifiers found

Below is a list of the input identifiers that have been found or mapped to an equivalent element in Reactome, classified by resource.

1 of the submitted entities were found, mapping to 1 Reactome entities

Input	UniProt Id
Ctr9	Q6PD62

7. Identifiers not found

These 3 identifiers were not found neither mapped to any entity in Reactome.

1700001L05Rik	6530402F18Rik	Scyl2
