# Supplementary material for: Sex- and region-specific cortical and hippocampal whole genome transcriptome profiles from control and APP/PS1 Alzheimer’s disease mice
Source: PLoS One. 2024 Feb 7;19(2):e0296959. doi: 10.1371/journal.pone.0296959 (PMC10849391; doi:10.1371/journal.pone.0296959)
Supplement: S1 File — S1 Fig: Genotyping of APP/PS1 AD mice and WT control animals. S2 Fig: 3D image of the murine brain including the RS cortex and hippocampus (BROIs) used for transcriptome analysis in our study. S3 Fig: PCA of transcriptomes from the RS cortex and hippocampus of WT controls and APP/PS1 AD mice of both sexes. S4 Fig: Hierarchical clustering of transcriptome data from the RS cortex and hippocampus of WT control and APP/PS1 AD mice of both sexes. S5 Fig: Bar diagrams of the top 30 candidates of DEGs with highest significant FCs (FC > 1.5 and FC < -1.5, p < 0.05). S6 Fig: Pathway analysis of intersectional and signature gene sets in APP/PS1 subgroups. S7 Fig: Comparative qPCR analysis of selected gene transcript levels from the hippocampus of female and male APP/PS1 AD with 5XFAD mice. S1 Table: PCR reaction set-up using PCR Mastermix and genomic DNA. S2 Table: Materials used for one-color microarray-based gene expression data collection. S3 Table: Software used for one-color microarray-based gene expression data collection. S4 Table: Details on genes, forward and reverse primer sequences and annealing temperatures relevant for qPCR experimentation. S5 Table: Characteristics of DEGs in the RS cortex of female APP/PS1 AD mice. S6 Table: Characteristics of DEGs in the hippocampus of female APP/PS1 AD mice. S7 Table: Characteristics of DEGs in the RS cortex of male APP/PS1 AD mice. S8 Table: Characteristics of DEGs in the hippocampus of male APP/PS1 AD mice. S9 Table: Venn analysis of DEGs in the RS cortex and hippocampus of female APP/PS1 AD mice. S10 Table: Venn analysis of DEGs genes in the RS cortex and hippocampus of male APP/PS1 AD mice. S11 Table: Venn analysis of DEGs in the RS cortex of male and female APP/PS1 AD mice. S12 Table: Venn analysis of DEGs in the hippocampus of male and female APP/PS1 AD mice. S13 Table: Differentially regulated l(i)ncRNAs in APP/PS1 AD vs. WT mice. S14 Table: qPCR-based FC analysis of selected genes in the hippocampus of APP/PS1 AD vs. [file pone.0296959.s001.zip › Supplementary Files_R1/Supplementary Figure 6_Pathways_upreg genes/Signature genes up_DEGs_female_Hip_APPPS1/Pathway analysis report.pdf]

# Pathway Analysis Report

This report contains the pathway analysis results for the submitted sample ''. Analysis was performed against Reactome version 85 on 11/08/2023. The web link to these results is:

https://reactome.org/PathwayBrowser/#/ANALYSIS=MjAyMzA4MTExMjU1NTZfNzU3NA%3D%3D

Please keep in mind that analysis results are temporarily stored on our server. The storage period depends on usage of the service but is at least 7 days. As a result, please note that this URL is only valid for a limited time period and it might have expired.

# Table of Contents


1. Introduction
2. Properties
3. Genome-wide overview
4. Most significant pathways
5. Pathways details
6. Identifiers found
7. Identifiers not found

# 1. Introduction

Reactome is a curated database of pathways and reactions in human biology. Reactions can be considered as pathway 'steps'. Reactome defines a 'reaction' as any event in biology that changes the state of a biological molecule. Binding, activation, translocation, degradation and classical biochemical events involving a catalyst are all reactions. Information in the database is authored by expert biologists, entered and maintained by Reactome’s team of curators and editorial staff. Reactome content frequently cross-references other resources e.g. NCBI, Ensembl, UniProt, KEGG (Gene and Compound), ChEBI, PubMed and GO. Orthologous reactions inferred from annotation for Homo sapiens are available for 14 non-human species including mouse, rat, chicken, puffer fish, worm, fly and yeast. Pathways are represented by simple diagrams following an SBGN-like format.

Reactome's annotated data describe reactions possible if all annotated proteins and small molecules were present and active simultaneously in a cell. By overlaying an experimental dataset on these annotations, a user can perform a pathway over-representation analysis. By overlaying quantitative expression data or time series, a user can visualize the extent of change in affected pathways and its progression. A binomial test is used to calculate the probability shown for each result, and the p-values are corrected for the multiple testing (Benjamini–Hochberg procedure) that arises from evaluating the submitted list of identifiers against every pathway.

To learn more about our Pathway Analysis, please have a look at our relevant publications:


Fabregat A, Sidiropoulos K, Garapati P, Gillespie M, Hausmann K, Haw R, … D’Eustachio P (2016). The reactome pathway knowledgebase. Nucleic Acids Research, 44(D1), D481–D487. https://doi.org/10.1093/nar/gkv1351.

Fabregat A, Sidiropoulos K, Viteri G, Forner O, Marin-Garcia P, Arnau V, … Hermjakob H (2017). Reactome pathway analysis: a high-performance in-memory approach. BMC Bioinformatics, 18.

## 2. Properties

- This is an **overrepresentation** analysis: A statistical (hypergeometric distribution) test that determines whether certain Reactome pathways are over-represented (enriched) in the submitted data. It answers the question 'Does my list contain more proteins for pathway X than would be expected by chance?' This test produces a probability score, which is corrected for false discovery rate using the Benjamani-Hochberg method.
- 11 out of 17 identifiers in the sample were found in Reactome, where 149 pathways were hit by at least one of them.
- All non-human identifiers have been converted to their human equivalent.
- This report is filtered to show only results for species 'Homo sapiens' and resource 'all resources'.
- The unique ID for this analysis (token) is MjAyMzA4MTExMjU1NTZfNzU3NA%3D%3D. This ID is valid for at least 7 days in Reactome's server. Use it to access Reactome services with your data.

# 3. Genome-wide overview

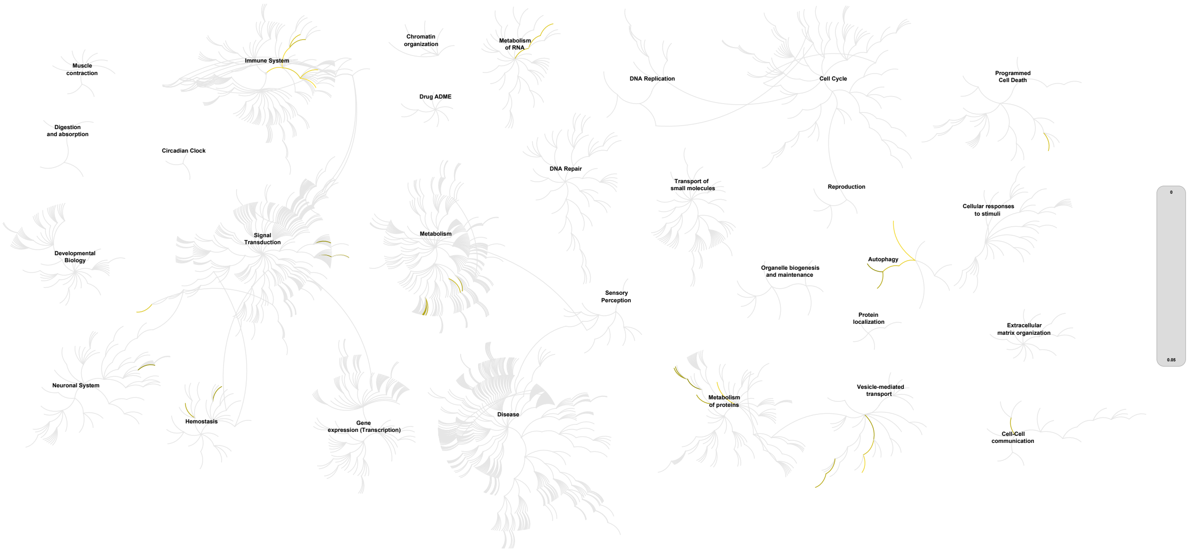


This figure shows a genome-wide overview of the results of your pathway analysis. Reactome pathways are arranged in a hierarchy. The center of each of the circular "bursts" is the root of one top-level pathway, for example "DNA Repair". Each step away from the center represents the next level lower in the pathway hierarchy. The color code denotes over-representation of that pathway in your input dataset. Light grey signifies pathways which are not significantly over-represented.

# 4. Most significant pathways

The following table shows the 25 most relevant pathways sorted by p-value.

| Pathway name | Entities | | | | Reactions | |
|---|---|---|---|---|---|---|
| | found | ratio | p-value | FDR* | found | ratio |
| Interleukin-4 and Interleukin-13 signaling | 4 / 211 | 0.014 | 1.82e-04 | 0.028 | 2 / 47 | 0.003 |
| Interferon Signaling | 4 / 322 | 0.021 | 8.96e-04 | 0.067 | 4 / 79 | 0.006 |
| Aggrephagy | 2 / 47 | 0.003 | 0.002 | 0.083 | 4 / 15 | 0.001 |
| Cytokine Signaling in Immune system | 6 / 1,039 | 0.068 | 0.002 | 0.083 | 7 / 745 | 0.052 |
| COPI-independent Golgi-to-ER retrograde traffic | 2 / 63 | 0.004 | 0.003 | 0.099 | 3 / 7 | 4.89e-04 |
| RAB geranylgeranylation | 2 / 68 | 0.004 | 0.004 | 0.099 | 2 / 5 | 3.49e-04 |
| Interleukin-10 signaling | 2 / 86 | 0.006 | 0.006 | 0.12 | 1 / 15 | 0.001 |
| Selective autophagy | 2 / 89 | 0.006 | 0.007 | 0.12 | 4 / 48 | 0.003 |
| Formation of the Editosome | 1 / 8 | 5.25e-04 | 0.011 | 0.124 | 1 / 2 | 1.40e-04 |
| Signaling by Interleukins | 4 / 658 | 0.043 | 0.012 | 0.124 | 3 / 505 | 0.035 |
| phospho-PLA2 pathway | 1 / 9 | 5.91e-04 | 0.012 | 0.124 | 3 / 3 | 2.10e-04 |
| Interferon alpha/beta signaling | 2 / 129 | 0.008 | 0.014 | 0.124 | 2 / 25 | 0.002 |
| mRNA Editing: C to U Conversion | 1 / 10 | 6.57e-04 | 0.014 | 0.124 | 2 / 3 | 2.10e-04 |
| Caspase-mediated cleavage of cytoskeletal proteins | 1 / 12 | 7.88e-04 | 0.016 | 0.124 | 3 / 10 | 6.99e-04 |
| mRNA Editing | 1 / 12 | 7.88e-04 | 0.016 | 0.124 | 2 / 9 | 6.29e-04 |
| Golgi-to-ER retrograde transport | 2 / 148 | 0.01 | 0.018 | 0.124 | 5 / 18 | 0.001 |
| Macroautophagy | 2 / 150 | 0.01 | 0.018 | 0.124 | 4 / 87 | 0.006 |
| Autophagy | 2 / 166 | 0.011 | 0.022 | 0.124 | 15 / 108 | 0.008 |
| Arachidonic acid metabolism | 2 / 168 | 0.011 | 0.022 | 0.124 | 2 / 79 | 0.006 |
| Interferon gamma signaling | 2 / 177 | 0.012 | 0.024 | 0.124 | 2 / 23 | 0.002 |
| Hydrolysis of LPC | 1 / 18 | 0.001 | 0.025 | 0.124 | 2 / 6 | 4.19e-04 |
| Acyl chain remodeling of CL | 1 / 18 | 0.001 | 0.025 | 0.124 | 1 / 12 | 8.39e-04 |
| Cell-extracellular matrix interactions | 1 / 19 | 0.001 | 0.026 | 0.124 | 1 / 10 | 6.99e-04 |
| Microtubule-dependent trafficking of connexons from Golgi to the plasma membrane | 1 / 22 | 0.001 | 0.03 | 0.124 | 1 / 2 | 1.40e-04 |
| Chaperone Mediated Autophagy | 1 / 23 | 0.002 | 0.031 | 0.124 | 9 / 19 | 0.001 |

* False Discovery Rate

# 5. Pathways details

For every pathway of the most significant pathways, we present its diagram, as well as a short summary, its bibliography and the list of inputs found in it.

## 1. Interleukin-4 and Interleukin-13 signaling (R-HSA-6785807)

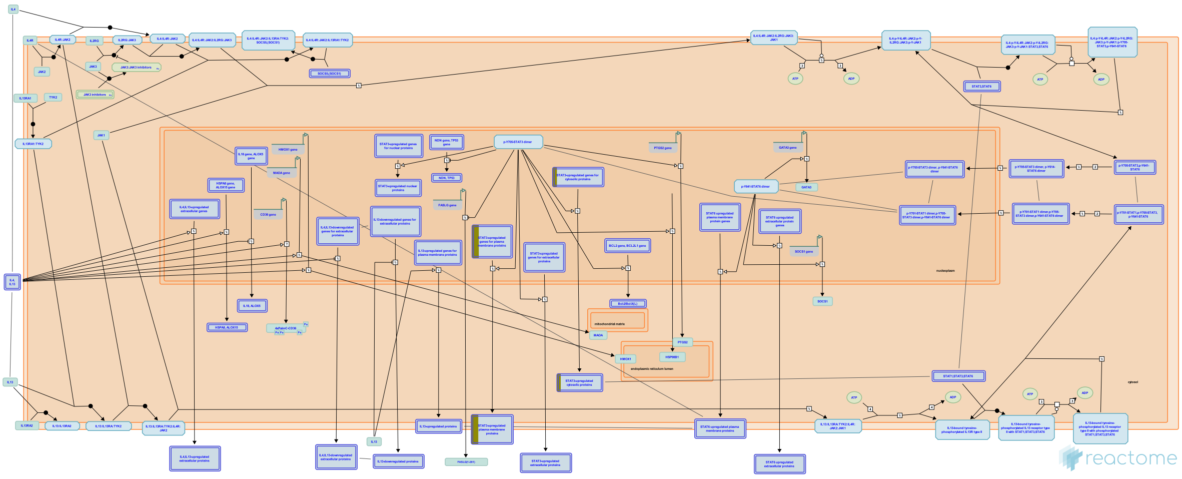

Interleukin-4 (IL4) is a principal regulatory cytokine during the immune response, crucially important in allergy and asthma (Nelms et al. 1999). When resting T cells are antigen-activated and expand in response to Interleukin-2 (IL2), they can differentiate as Type 1 (Th1) or Type 2 (Th2) T helper cells. The outcome is influenced by IL4. Th2 cells secrete IL4, which both stimulates Th2 in an autocrine fashion and acts as a potent B cell growth factor to promote humoral immunity (Nelms et al. 1999).

Interleukin-13 (IL13) is an immunoregulatory cytokine secreted predominantly by activated Th2 cells. It is a key mediator in the pathogenesis of allergic inflammation. IL13 shares many functional properties with IL4, stemming from the fact that they share a common receptor subunit. IL13 receptors are expressed on human B cells, basophils, eosinophils, mast cells, endothelial cells, fibroblasts, monocytes, macrophages, respiratory epithelial cells, and smooth muscle cells, but unlike IL4, not T cells. Thus IL13 does not appear to be important in the initial differentiation of CD4 T cells into Th2 cells, rather it is important in the effector phase of allergic inflammation (Hershey et al. 2003).

IL4 and IL13 induce “alternative activation” of macrophages, inducing an anti-inflammatory phenotype by signaling through IL4R alpha in a STAT6 dependent manner. This signaling plays an important role in the Th2 response, mediating anti-parasitic effects and aiding wound healing (Gordon & Martinez 2010, Loke et al. 2002)

There are two types of IL4 receptor complex (Andrews et al. 2006). Type I IL4R (IL4R1) is predominantly expressed on the surface of hematopoietic cells and consists of IL4R and IL2RG, the common gamma chain. Type II IL4R (IL4R2) is predominantly expressed on the surface of nonhematopoietic cells, it consists of IL4R and IL13RA1 and is also the type II receptor for IL13. (Obiri et al. 1995, Aman et al. 1996, Hilton et al. 1996, Miloux et al. 1997, Zhang et al. 1997). The second receptor for IL13 consists of IL4R and Interleukin-13 receptor alpha 2 (IL13RA2), sometimes called Interleukin-13 binding protein (IL13BP). It has a high affinity receptor for IL13 (Kd = 250 pmol/L) but is not sufficient to render cells responsive to IL13, even in the presence of IL4R (Donaldson et al. 1998). It is reported to exist in soluble form (Zhang et al. 1997) and when overexpressed reduces JAK-STAT signaling (Kawakami et al. 2001). It's function may be to prevent IL13 signalling via the functional IL4R:IL13RA1 receptor. IL13RA2 is overexpressed and enhances cell invasion in some human cancers (Joshi & Puri 2012).

The first step in the formation of IL4R1 (IL4:IL4R:IL2RB) is the binding of IL4 with IL4R (Hoffman et al. 1995, Shen et al. 1996, Hage et al. 1999). This is also the first step in formation of IL4R2 (IL4:IL4R:IL13RA1). After the initial binding of IL4 and IL4R, IL2RB binds (LaPorte et al. 2008), to form IL4R1. Alternatively, IL13RA1 binds, forming IL4R2. In contrast, the type II IL13 complex (IL13R2) forms with IL13 first binding to IL13RA1 followed by recruitment of IL4R (Wang et al. 2009).

Crystal structures of the IL4:IL4R:IL2RG, IL4:IL4R:IL13RA1 and IL13:IL4R:IL13RA1 complexes have been determined (LaPorte et al. 2008). Consistent with these structures, in monocytes IL4R is tyrosine phosphorylated in response to both IL4 and IL13 (Roy et al. 2002, Gordon & Martinez 2010) while IL13RA1 phosphorylation is induced only by IL13 (Roy et al. 2002, LaPorte et al. 2008) and IL2RG phosphorylation is induced only by IL4 (Roy et al. 2002).

Both IL4 receptor complexes signal through Jak/STAT cascades. IL4R is constitutively-associated with JAK2 (Roy et al. 2002) and associates with JAK1 following binding of IL4 (Yin et al. 1994) or IL13 (Roy et al. 2002). IL2RG constitutively associates with JAK3 (Boussiotis et al. 1994, Russell et al. 1994). IL13RA1 constitutively associates with TYK2 (Umeshita-Suyama et al. 2000, Roy et al. 2002, LaPorte et al. 2008, Bhattacharjee et al. 2013).

IL4 binding to IL4R1 leads to phosphorylation of JAK1 (but not JAK2) and STAT6 activation (Takeda et al. 1994, Ratthe et al. 2007, Bhattacharjee et al. 2013).

IL13 binding increases activating tyrosine-99 phosphorylation of IL13RA1 but not that of IL2RG. IL4 binding to IL2RG leads to its tyrosine phosphorylation (Roy et al. 2002). IL13 binding to IL4R2 leads to TYK2 and JAK2 (but not JAK1) phosphorylation (Roy & Cathcart 1998, Roy et al. 2002).

Phosphorylated TYK2 binds and phosphorylates STAT6 and possibly STAT1 (Bhattacharjee et al. 2013).

A second mechanism of signal transduction activated by IL4 and IL13 leads to the insulin receptor substrate (IRS) family (Kelly-Welch et al. 2003). IL4R1 associates with insulin receptor substrate 2 and activates the PI3K/Akt and Ras/MEK/Erk pathways involved in cell proliferation, survival and translational control. IL4R2 does not associate with insulin receptor substrate 2 and consequently the PI3K/Akt and Ras/MEK/Erk pathways are not activated (Busch-Dienstfertig & González-Rodríguez 2013).

## References


Ryan JJ, Nelms K, Paul WE, Zamorano J & Keegan AD (1999). The IL-4 receptor: signaling mechanisms and biologic functions. Annu. Rev. Immunol., 17, 701-38.

Hershey GK (2003). IL-13 receptors and signaling pathways: an evolving web. J. Allergy Clin. Immunol., 111, 677-90; quiz 691.


## Edit history

| Date | Action | Author |
|---|---|---|
| 2015-07-01 | Authored | Jupe S |
| 2015-07-01 | Created | Jupe S |
| 2016-09-02 | Edited | Jupe S |
| 2016-09-02 | Reviewed | Leibovich SJ |
| 2023-05-30 | Modified | Wright A |

## 2 submitted entities found in this pathway, mapping to 4 Reactome entities

| Input | UniProt Id |
|---|---|
| Icam1 | P05362 |

| Input | UniProt Id |
|---|---|
| Vim | P08670 |

| Input | Ensembl Id |
|---|---|
| Icam1 | ENSG00000090339 |

| Input | Ensembl Id |
|---|---|
| Vim | ENSG00000026025 |

## 2. Interferon Signaling (R-HSA-913531)

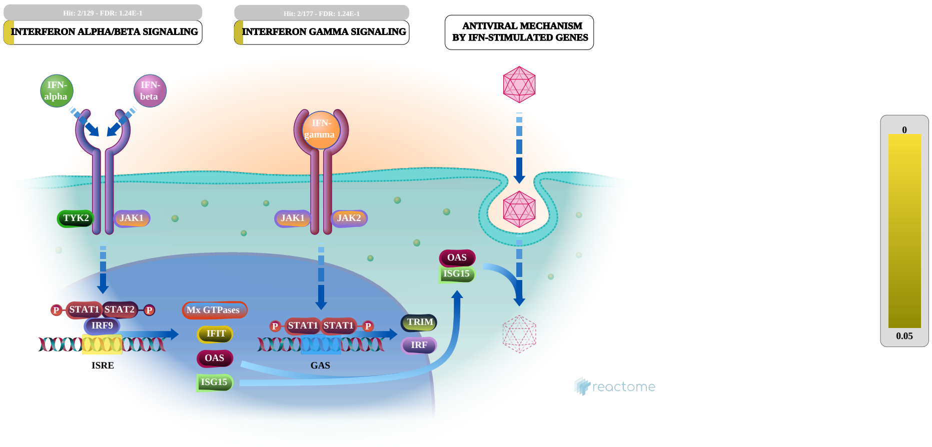


Interferons (IFNs) are cytokines that play a central role in initiating immune responses, especially antiviral and antitumor effects. There are three types of IFNs:Type I (IFN-alpha, -beta and others, such as omega, epsilon, and kappa), Type II (IFN-gamma) and Type III (IFN-lamda). In this module we are mainly focusing on type I IFNs alpha and beta and type II IFN-gamma. Both type I and type II IFNs exert their actions through cognate receptor complexes, IFNAR and IFNGR respectively, present on cell surface membranes. Type I IFNs are broadly expressed heterodimeric receptors composed of the IFNAR1 and IFNAR2 subunits, while the type II IFN receptor consists of IFNGR1 and IFNGR2. Type III interferon lambda has three members: lamda1 (IL-29), lambda2 (IL-28A), and lambda3 (IL-28B) respectively. IFN-lambda signaling is initiated through unique heterodimeric receptor composed of IFN-LR1/IF-28Ralpha and IL10R2 chains.

Type I IFNs typically recruit JAK1 and TYK2 proteins to transduce their signals to STAT1 and 2; in combination with IRF9 (IFN-regulatory factor 9), these proteins form the heterotrimeric complex ISGF3. In nucleus ISGF3 binds to IFN-stimulated response elements (ISRE) to promote gene induction.

Type II IFNs in turn rely upon the activation of JAKs 1 and 2 and STAT1. Once activated, STAT1 dimerizes to form the transcriptional regulator GAF (IFNG activated factor) and this binds to the IFNG activated sequence (GAS) elements and initiate the transcription of IFNG-responsive genes.

Like type I IFNs, IFN-lambda recruits TYK2 and JAK1 kinases and then promote the phosphorylation of STAT1/2, and induce the ISRE3 complex formation.

### References

Schroder K, Ravasi T, Hume DA & Hertzog PJ (2004). Interferon-gamma: an overview of signals, mechanisms and functions. J Leukoc Biol, 75, 163-89.

Platanias LC (2005). Mechanisms of type-I- and type-II-interferon-mediated signalling. Nat Rev Immunol, 5, 375-86.

Gough DJ, Levy DE, Clarke CJ & Johnstone RW (2008). IFNgamma signaling-does it mean JAK-STAT?. Cytokine Growth Factor Rev, 19, 383-94.

Ferreira PC, Bonjardim CA & Kroon EG (2009). Interferons: signaling, antiviral and viral evasion. Immunol Lett, 122, 1-11.

Platanias LC & Uddin S (2004). Mechanisms of type-I interferon signal transduction. J Biochem Mol Biol, 37, 635-41.

## Edit history

| Date | Action | Author |
|---|---|---|
| 2010-07-07 | Edited | Garapati P V |
| 2010-07-07 | Authored | Garapati P V |
| 2010-07-16 | Created | Garapati P V |
| 2010-08-17 | Reviewed | Abdul-Sater AA, Schindler C |
| 2023-05-21 | Modified | Wright A |

## 2 submitted entities found in this pathway, mapping to 4 Reactome entities

| Input | UniProt Id |
|---|---|
| Icam1 | P05362 |

| Input | UniProt Id |
|---|---|
| Ifitm3 | Q01628 |

| Input | Ensembl Id |
|---|---|
| Icam1 | ENSG00000090339 |

| Input | Ensembl Id |
|---|---|
| Ifitm3 | ENSG00000142089 |

## 3. Aggrephagy (R-HSA-9646399)

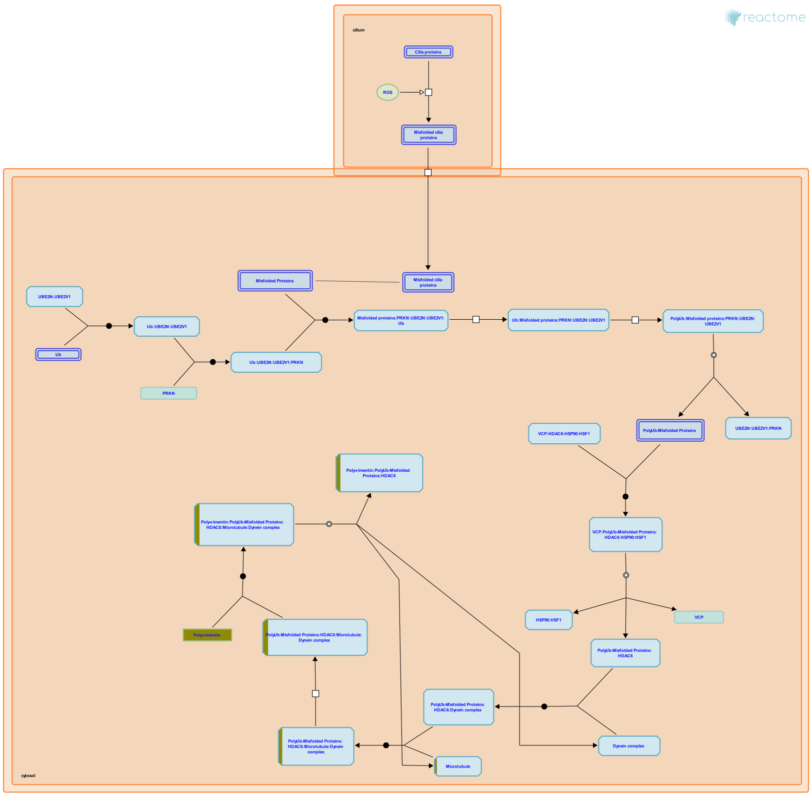

**Cellular compartments:** cytosol.

When the capacity of the proteosome to degrade misfolded proteins is limited, the alternate route to eliminate denatured proteins is via forming aggresomes - a process known as aggrephagy. Aggresome formation starts with ubiquitination of misfolded proteins following transport to the microtubule-organizing center (MTOC) with the help of dynein motor proteins. At the MTOC the cargo is encapsulated with intermediate filament proteins to result in the aggresome. Subsequently, this aggresome recruits chaperones that result in its autophagic elimination (Garcia Mata R et al. 2002).

### References


Gao YS, Sztul E & Garcia-Mata R (2002). Hassles with taking out the garbage: aggravating aggresomes. Traffic, 3, 388-96.


### Edit history

| Date | Action | Author |
|---|---|---|
| 2019-05-23 | Authored | Varusai TM |
| 2019-05-23 | Created | Varusai TM |
| 2019-05-24 | Reviewed | Metzakopian E |
| 2019-11-08 | Edited | Varusai TM |
| 2023-05-30 | Modified | Wright A |

### 2 submitted entities found in this pathway, mapping to 2 Reactome entities

| Input | UniProt Id | Input | UniProt Id |
|---|---|---|---|
| Tubb6 | Q9BUF5 | Vim | P08670 |

## 4. Cytokine Signaling in Immune system (R-HSA-1280215)

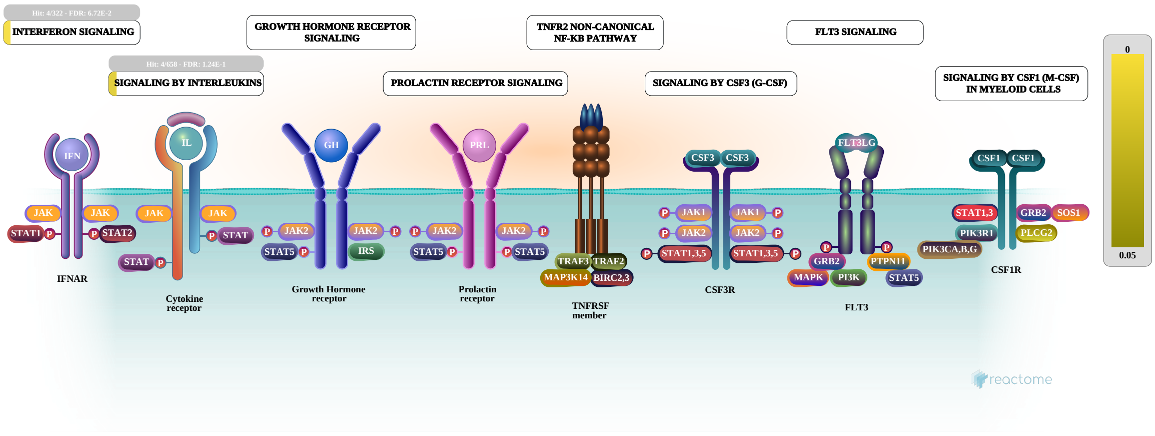


Cytokines are small proteins that regulate and mediate immunity, inflammation, and hematopoiesis. They are secreted in response to immune stimuli, and usually act briefly, locally, at very low concentrations. Cytokines bind to specific membrane receptors, which then signal the cell via second messengers, to regulate cellular activity.

### References


Feldmann M & Oppenheim J (2002). *Cytokines and the immune system, Cytokine Reference* .

IMMPORT:Bioinformatics for the future of immunology. Retrieved from https://www.immport.org/immportWeb/queryref/geneListSummary.do

Santamaria P (2003). Cytokines and chemokines in autoimmune disease: an overview. Adv Exp Med Biol, 520, 1-7.

COPE. Retrieved from http://www.copewithcytokines.org/cope.cgi


### Edit history

| Date | Action | Author |
|---|---|---|
| 2011-05-12 | Created | Garapati P V |
| 2011-05-22 | Edited | Ray KP, Jupe S, Garapati P V |
| 2011-05-22 | Authored | Ray KP, Jupe S, Garapati P V |
| 2011-05-29 | Reviewed | Abdul-Sater AA, Schindler C, Pinteaux E |
| 2023-05-21 | Modified | Wright A |

### 3 submitted entities found in this pathway, mapping to 6 Reactome entities

| Input | UniProt Id | Input | UniProt Id | Input | UniProt Id |
|---|---|---|---|---|---|
| Icam1 | P05362 | Ifitm3 | Q01628 | Vim | P08670 |

| Input | Ensembl Id | Input | Ensembl Id | Input | Ensembl Id |
|---|---|---|---|---|---|
| Icam1 | ENSG00000090339 | Ifitm3 | ENSG00000142089 | Vim | ENSG00000026025 |

## 5. COPI-independent Golgi-to-ER retrograde traffic (R-HSA-6811436)

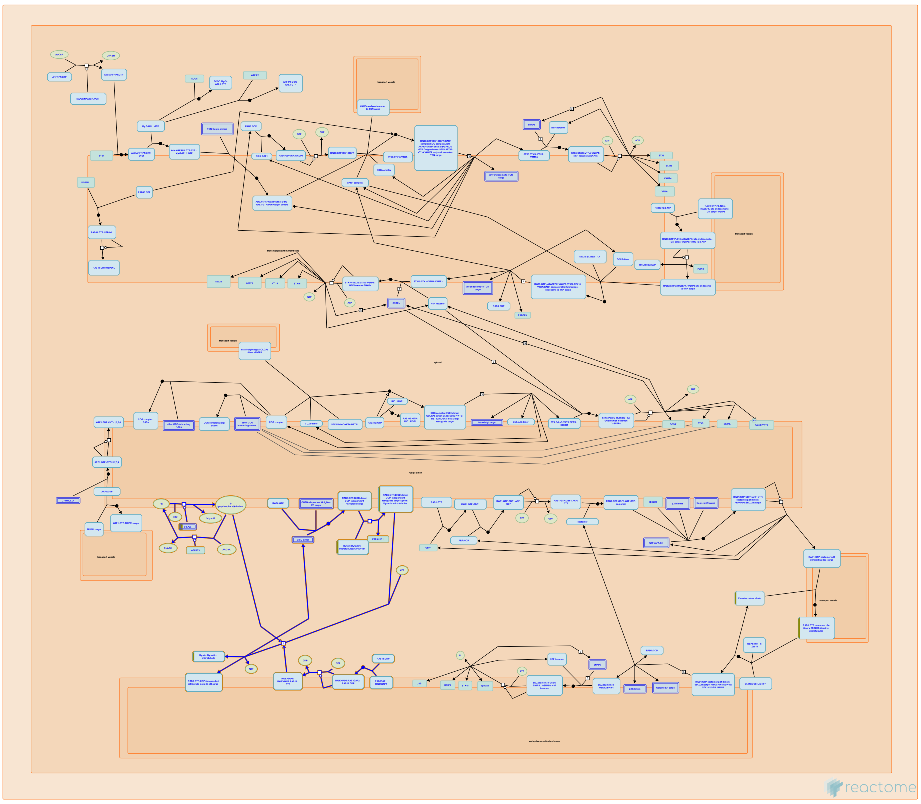

In addition to the better characterized COPI-dependent retrograde Golgi-to-ER pathway, a second COPI-independent pathway has also been identified. This pathway is RAB6 dependent and transports cargo such as glycosylation enzymes and Shiga and Shiga-like toxin through tubular carriers rather than vesicles (White et al, 1999; Girod et al, 1999; reviewed in Heffernan and Simpson, 2014). In the absence of a COPI coat, the membrane curvature necessary to initiate tubulation may be provided through the action of phospholipase A, which hydrolyzes phospholipids at the sn2 position to yield lysophospholipids. This activity is countered by lysophospholipid acyltransferases, and the balance of these may influence whether transport tubules or transport vesicles form (de Figuiredo et al, 1998; reviewed in Bechler et al, 2012). RAB6-dependent tubules also depend on the dynein-dynactin motor complex and the hoomodimeric Bicaudal proteins (Matanis et al, 2002; Yamada et al, 2013; reviewed in Heffernan and Simpson, 2014).

### References


Bechler ME, Brown WJ & de Figueiredo P (2012). A PLA1-2 punch regulates the Golgi complex. Trends Cell Biol., 22, 116-24.

Girod A, White J, Grill S, Reinsch S, Johannes L, Stelzer EH, ... Mallard F (1999). Rab6 coordinates a novel Golgi to ER retrograde transport pathway in live cells. J. Cell Biol., 147, 743-60.

Weide T, Grosveld F, Matanis T, Wulf P, Barnekow A, Galjart N, ... del Nery E (2002). Bicaudal-D regulates COPI-independent Golgi-ER transport by recruiting the dynein-dynactin motor complex. Nat. Cell Biol., 4, 986-92.

Drecktrah D, Katzenellenbogen JA, Strang M, Brown WJ & de Figueiredo P (1998). Evidence that phospholipase A2 activity is required for Golgi complex and trans Golgi network membrane tubulation. Proc. Natl. Acad. Sci. U.S.A., 95, 8642-7.

Fukui M, Mikuni S, Jin M, Kumamoto K, Arai Y, Tsukasaki Y, ... Toba S (2013). Rab6a releases LIS1 from a dynein idling complex and activates dynein for retrograde movement. Nat Commun, 4, 2033.

## Edit history

| Date | Action | Author |
|---|---|---|
| 2015-11-09 | Edited | Rothfels K |
| 2015-11-09 | Authored | Rothfels K |
| 2015-11-19 | Created | Rothfels K |
| 2016-02-02 | Reviewed | Gillespie ME |
| 2023-05-21 | Modified | Wright A |

## 2 submitted entities found in this pathway, mapping to 2 Reactome entities

| Input | UniProt Id | Input | UniProt Id |
|---|---|---|---|
| Pla2g4a | P47712 | Tubb6 | Q9BUF5 |

## 6. RAB geranylgeranylation (R-HSA-8873719)

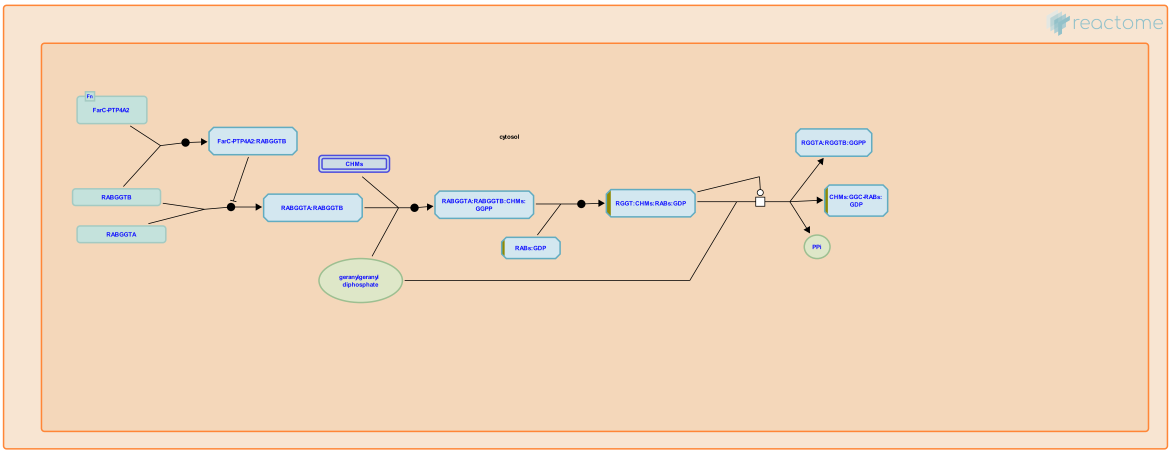


Human cells have more than 60 RAB proteins that are involved in trafficking of proteins in the endolysosomal system. These small GTPases contribute to trafficking specificity by localizing to the membranes of different endocytic compartments and interacting with effectors such as sorting adaptors, tethering factors, kinases, phosphatases and tubular-vesicular cargo (reviewed in Stenmark et al, 2009; Wandinger-Ness and Zerial, 2014). RAB localization depends on a number of factors including C-terminal prenylation, the sequence of an upstream hypervariable regions and what nucleotide is bound (Chavrier et al, 1991; Ullrich et al, 1993; Soldati et al, 1994; Farnsworth et al, 1994; Seabra, 1996; Wu et al, 2010; reviewed in Stenmark, 2009; Wandinger-Ness and Zerial, 2014). In the active, GTP-bound form, prenylated RAB proteins are membrane associated, while in the inactive GDP-bound form, RABs are extracted from the target membrane and exist in a soluble form in complex with GDP dissociation inhibitors (GDIs) (Ullrich et al, 1993; Soldati et al, 1994; Gavriljuk et al, 2103). Conversion between the inactive and active form relies on the activities of RAB guanine nucleotide exchange factors (GEFs) and GTPase activating proteins (GAPs) (Yoshimura et al, 2010; Wu et al, 2011; Pan et al, 2006; Frasa et al, 2012; reviewed in Stenmark, 2009; Wandinger-Ness and Zerial, 2014).

Newly synthesized RABs are bound by a RAB escort protein, CHM (also known as REP1) or CHML (REP2) (Alexandrov et al, 1994; Shen and Seabra, 1996). CHM/REP proteins are the substrate-binding component of the trimeric RAB geranylgeranyltransferase enzyme (GGTaseII) along with the two catalytic subunits RABGGTA and RABGGTB (reviewed in Gutkowska and Swiezewska, 2012; Palsuledesai and Distefano, 2015). REP proteins recruit the unmodified RAB in its GDP-bound state to the GGTase for sequential geranylgeranylation at one or two C-terminal cysteine residues (Alexandrov et al, 1994; Seabra et al 1996; Shen and Seabra, 1996; Baron and Seabra, 2008). After geranylgeranylation, CHM/REP proteins remain in complex with the geranylgeranylated RAB and escort it to its target membrane, where its activity is regulated by GAPs, GEFs, GDIs and membrane-bound GDI displacement factors (GDFs) (Sivars et al, 2003; reviewed in Stenmark, 2009; Wandinger-Ness and Zerial, 2014).

### References

Goody RS, Kötting C, Itzen A, Gerwert K & Gavriljuk K (2013). Membrane extraction of Rab proteins by GDP dissociation inhibitor characterized using attenuated total reflection infrared spectroscopy. Proc. Natl. Acad. Sci. U.S.A., 110, 13380-5.

Seabra MC (1996). Nucleotide dependence of Rab geranylgeranylation. Rab escort protein interacts preferentially with GDP-bound Rab. J. Biol. Chem., 271, 14398-404.

Stenmark H (2009). Rab GTPases as coordinators of vesicle traffic. Nat. Rev. Mol. Cell Biol., 10, 513-25.

Lambright DG, Pan X, Munson M & Eathiraj S (2006). TBC-domain GAPs for Rab GTPases accelerate GTP hydrolysis by a dual-finger mechanism. Nature, 442, 303-6.

Swiezewska E & Gutkowska M (2012). Structure, regulation and cellular functions of Rab geranyl-geranyl transferase and its cellular partner Rab Escort Protein. Mol. Membr. Biol., 29, 243-56.

## Edit history

| Date | Action | Author |
|---|---|---|
| 2016-05-20 | Created | Rothfels K |
| 2016-06-03 | Edited | Rothfels K |
| 2016-06-03 | Authored | Rothfels K |
| 2016-08-04 | Reviewed | Palsuledesai CC |
| 2023-05-21 | Modified | Wright A |

## 1 submitted entities found in this pathway, mapping to 2 Reactome entities

| Input | UniProt Id |
|---|---|
| Rab32 | P57729, Q13637 |

## 7. Interleukin-10 signaling (R-HSA-6783783)

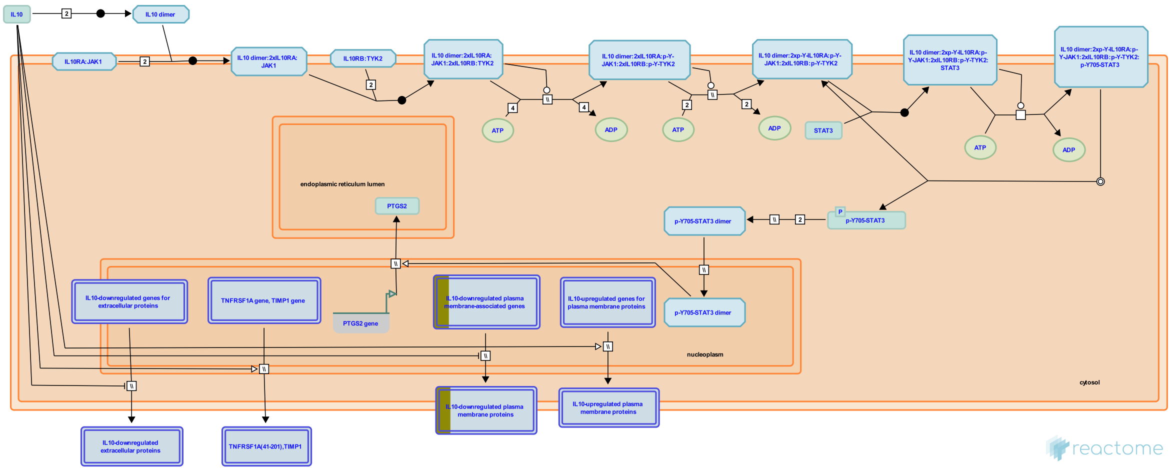

Interleukin-10 (IL10) was originally described as a factor named cytokine synthesis inhibitory factor that inhibited T-helper (Th) 1 activation and Th1 cytokine production (Fiorentino et al. 1989). It was found to be expressed by a variety of cell types including macrophages, dendritic cell subsets, B cells, several T-cell subpopulations including Th2 and T-regulatory cells (Tregs) and Natural Killer (NK) cells (Moore et al. 2001). It is now recognized that the biological effects of IL10 are directed at antigen-presenting cells (APCs) such as macrophages and dendritic cells (DCs), its effects on T-cell development and differentiation are largely indirect via inhibition of macrophage/dendritic cell activation and maturation (Pestka et al. 2004, Mocellin et al. 2004). T cells are thought to be the main source of IL10 (Hedrich & Bream 2010). IL10 inhibits a broad spectrum of activated macrophage/monocyte functions including monokine synthesis, NO production, and expression of class II MHC and costimulatory molecules such as IL12 and CD80/CD86 (de Waal Malefyt et al. 1991, Gazzinelli et al. 1992). Studies with recombinant cytokine and neutralizing antibodies revealed pleiotropic activities of IL10 on B, T, and mast cells (de Waal Malefyt et al. 1993, Rousset et al. 1992, Thompson-Snipes et al. 1991) and provided evidence for the in vivo significance of IL10 activities (Ishida et al. 1992, 1993). IL10 antagonizes the expression of MHC class II and the co-stimulatory molecules CD80/CD86 as well as the pro-inflammatory cytokines IL1Beta, IL6, IL8, TNFalpha and especially IL12 (Fiorentino et al. 1991, D'Andrea et al. 1993). The biological role of IL10 is not limited to inactivation of APCs, it also enhances B cell, granulocyte, mast cell, and keratinocyte growth/differentiation, as well as NK-cell and CD8+ cytotoxic T-cell activation (Moore et al. 2001, Hedrich & Bream 2010). IL10 also enhances NK-cell proliferation and/or production of IFN-gamma (Cai et al. 1999).

IL10-deficient mice exhibited inflammatory bowel disease (IBD) and other exaggerated inflammatory responses (Kuhn et al. 1993, Berg et al. 1995) indicating a critical role for IL10 in limiting inflammatory responses. Dysregulation of IL10 is linked with susceptibility to numerous infectious and autoimmune diseases in humans and mouse models (Hedrich & Bream 2010).

IL10 signaling is initiated by binding of homodimeric IL10 to the extracellular domains of two adjoining IL10RA molecules. This tetramer then binds two IL10RB chains. IL10RB cannot bind to IL10 unless bound to IL10RA (Ding et al. 2001, Yoon et al. 2006); binding of IL10 to IL10RA without the co-presence of IL10RB fails to initiate signal transduction (Kotenko et al. 1997).

IL10 binding activates the receptor-associated Janus tyrosine kinases, JAK1 and TYK2, which are constitutively bound to IL10R1 and IL10R2 respectively. In the classic model of receptor activation assembly of the receptor complex is believed to enable JAK1/TYK2 to phosphorylate and activate each other. Alternatively the binding of IL10 may cause conformational changes that allow the pseudokinase inhibitory domain of one JAK kinase to move away from the kinase domain of the other JAK within the receptor dimer-JAK complex, allowing the two kinase domains to interact and trans-activate (Waters & Brooks 2015).

The activated JAK kinases phosphorylate the intracellular domains of the IL10R1 chains on specific tyrosine residues. These phosphorylated tyrosine residues and their flanking peptide sequences serve as temporary docking sites for the latent, cytosolic, transcription factor, STAT3. STAT3 transiently docks on the IL10R1 chain via its SH2 domain, and is in turn tyrosine phosphorylated by the receptor-associated JAKs. Once activated, it dissociates from the receptor, dimerizes with other STAT3 molecules, and translocates to the nucleus where it binds with high affinity to STAT-binding elements (SBEs) in the promoters of IL-10-inducible genes (Donnelly et al. 1999).

## References


Moore KW, O'Garra A, Coffman RL & de Waal Malefyt R (2001). Interleukin-10 and the interleukin-10 receptor. Annu. Rev. Immunol., 19, 683-765.


## Edit history

| Date | Action | Author |
|---|---|---|
| 2015-06-17 | Authored | Jupe S |
| 2015-06-17 | Created | Jupe S |
| 2016-09-05 | Reviewed | Meldal BH |
| 2016-11-14 | Edited | Jupe S |
| 2023-05-30 | Modified | Wright A |

## 1 submitted entities found in this pathway, mapping to 2 Reactome entities

| Input | UniProt Id |
|---|---|
| Icam1 | P05362 |

| Input | Ensembl Id |
|---|---|
| Icam1 | ENSG00000090339 |

## 8. Selective autophagy (R-HSA-9663891)

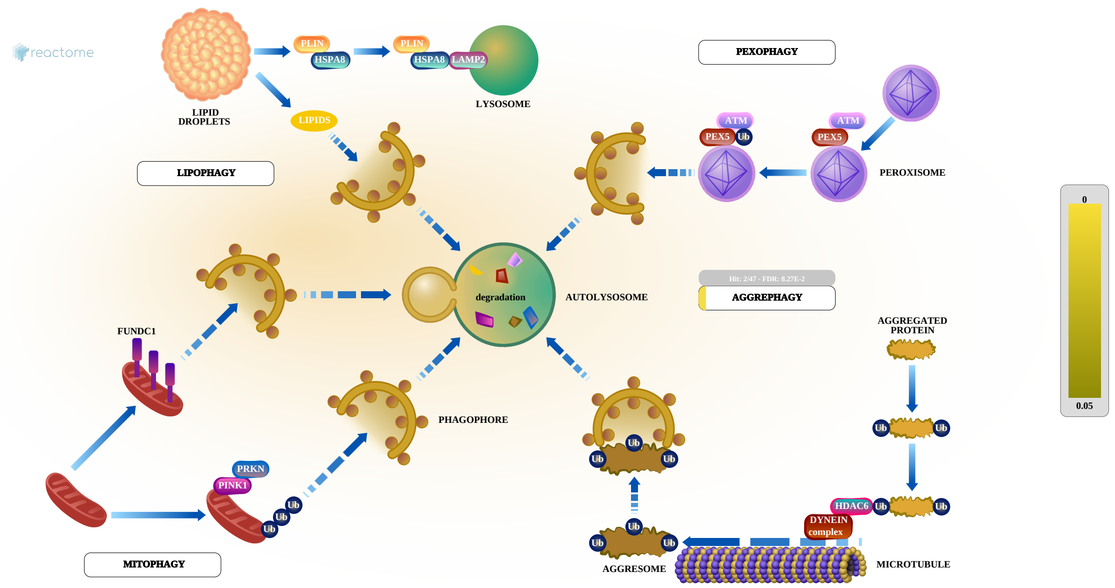


**Cellular compartments:** phagophore assembly site membrane, phagocytic vesicle, lysosomal lumen, autophagosome membrane, phagocytic vesicle membrane, cytosol, lysosomal membrane, autophagosome.

Autophagy can be a selective process where specific cargo (organelles/proteins) are targetted to degradation in the lysosome. In general, selective autophagy is initiated when a cellular signal tags the cargo organelle for degradation. Subsequently, cargo recognition proteins detect and recruit the organelle to interact directly or indirectly with Atg proteins forming the phagophore. The next steps involve formation of the autophagosome and fusion with the lysosome for degradation. Depending upon the organelle, different molecules are used to for the autophagy mechanism (Andling AL et al. 2017). Consequently, the different mechanisms are known by the organelle degraded such as mitophagy for mitochondria, lipophagy for lipid droplets, pexophagy for peroxisomes and aggrephagy for aggregated proteins.

### References

Anding AL & Baehrecke EH (2017). Cleaning House: Selective Autophagy of Organelles. Dev. Cell, 41, 10-22.

### Edit history

| Date | Action | Author |
|---|---|---|
| 2019-10-17 | Created | Varusai TM |
| 2019-11-08 | Edited | Varusai TM |
| 2019-11-08 | Authored | Varusai TM |
| 2023-05-21 | Modified | Wright A |

### 2 submitted entities found in this pathway, mapping to 2 Reactome entities

| Input | UniProt Id | Input | UniProt Id |
|---|---|---|---|
| Tubb6 | Q9BUF5 | Vim | P08670 |

| Input | UniProt Id | Input | UniProt Id |
| --- | --- | --- | --- |

## 9. Formation of the Editosome (R-HSA-75094)

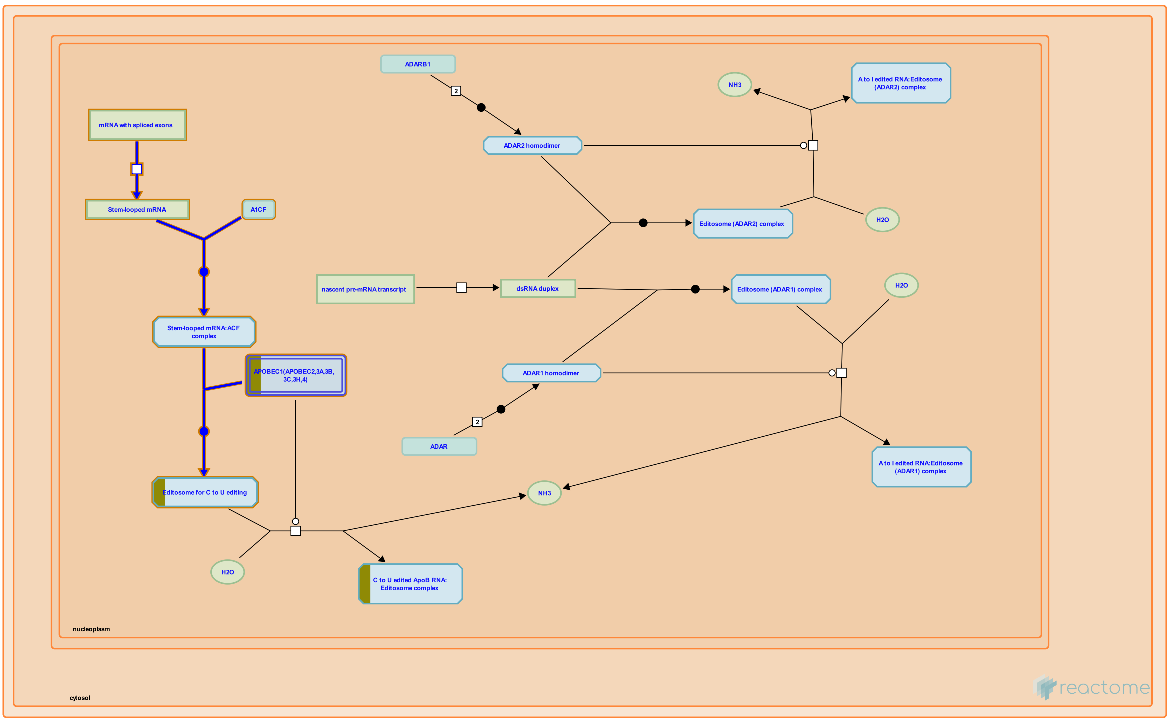

**Cellular compartments:** nucleoplasm.

The editosome for C to U editing in mammals consist of a member of cytidine deaminase family of enzymes, apoB mRNA editig catalytic polypeptide 1 (APOBEC-1) and a complementing specificity factor (ACF) in addition to the target mRNA.

### References

### Edit history

| Date | Action | Author |
|---|---|---|
| 2003-08-22 | Created | Carmichael GG |
| 2003-12-05 | Authored | Gopinathrao G |
| 2023-05-21 | Modified | Wright A |

### 1 submitted entities found in this pathway, mapping to 1 Reactome entities

| Input | UniProt Id |
|---|---|
| Apobec1 | P41238 |

## 10. Signaling by Interleukins (R-HSA-449147)

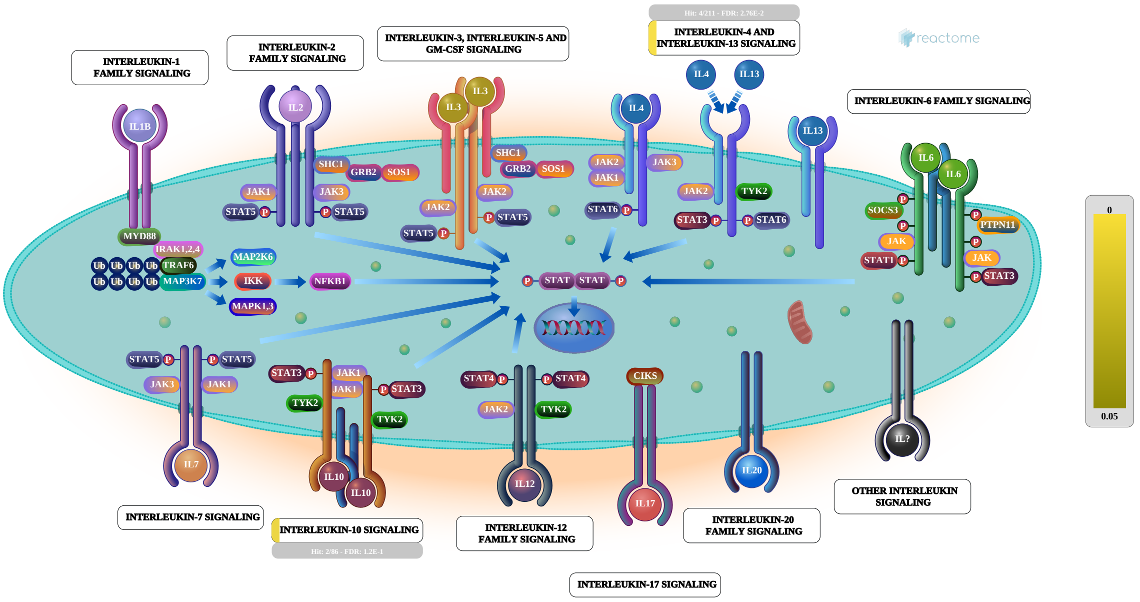


**Cellular compartments:** plasma membrane.

Interleukins are low molecular weight proteins that bind to cell surface receptors and act in an autocrine and/or paracrine fashion. They were first identified as factors produced by leukocytes but are now known to be produced by many other cells throughout the body. They have pleiotropic effects on cells which bind them, impacting processes such as tissue growth and repair, hematopoietic homeostasis, and multiple levels of the host defense against pathogens where they are an essential part of the immune system.

### References


Dinarello CA (2009). Immunological and inflammatory functions of the interleukin-1 family. Annu Rev Immunol, 27, 519-50.

Komlosi Z, Kucuksezer UC, Frei R, Huitema C, Garbani M, Pezer M, ... Eiwegger T (2016). Interleukins (from IL-1 to IL-38), interferons, transforming growth factor [illegible], and TNF-[illegible]: Receptors, functions, and roles in diseases. J. Allergy Clin. Immunol., 138, 984-1010.

Vosshenrich CA & Di Santo JP (2002). Interleukin signaling. Curr Biol, 12, R760-3.


### Edit history

| Date | Action | Author |
|---|---|---|
| 2009-11-27 | Created | Jupe S |
| 2010-05-17 | Reviewed | Pinteaux E |
| 2010-05-17 | Authored | Ray KP |
| 2010-05-26 | Edited | Jupe S |
| 2023-05-21 | Modified | Wright A |

### 2 submitted entities found in this pathway, mapping to 4 Reactome entities

| Input | UniProt Id |
|---|---|
| Icam1 | P05362 |

| Input | UniProt Id |
|---|---|
| Vim | P08670 |

| Input | Ensembl Id |
|---|---|
| Icam1 | ENSG00000090339 |

| Input | Ensembl Id |
|---|---|
| Vim | ENSG00000026025 |

## 11. phospho-PLA2 pathway (R-HSA-111995)

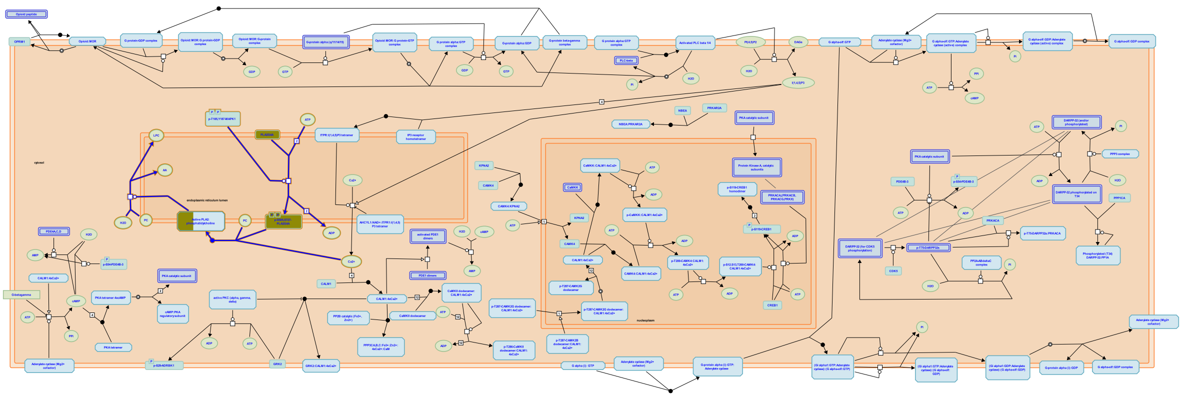

**Cellular compartments:** endoplasmic reticulum membrane, endoplasmic reticulum lumen, cytosol.

Phospholipase A2 (PLA2) enzymes hydrolyze arachidonic acid (AA) from the sn-2 position of phospholipids. AA is a precursor of eicosanoids, lipid mediators involved in inflammtory responses. PLA2 enzymes function as regulators of phospholipid acyl turnover, either as housekeepers for membrane repair or for the production of imflammatory lipid mediators. There are diverse forms of PLA2 enyzmes including secretory (sPLA2), calcium-independent and cytosolic (cPLA2). The cPLA2 form which mediates arachidonic acid release is annotated here.

### References


Leslie CC (1997). Properties and regulation of cytosolic phospholipase A2. J Biol Chem, 272, 16709-12.


### Edit history

| Date | Action | Author |
| --- | --- | --- |
| 2004-03-31 | Authored | Jassal B, Le Novere N |
| 2004-03-31 | Created | Schmidt EE |
| 2008-11-06 | Edited | Jassal B |
| 2008-11-06 | Reviewed | Castagnoli L |
| 2023-05-21 | Modified | Wright A |

### 1 submitted entities found in this pathway, mapping to 1 Reactome entities

| Input | UniProt Id |
| --- | --- |
| Pla2g4a | P47712 |

## 12. Interferon alpha/beta signaling (R-HSA-909733)

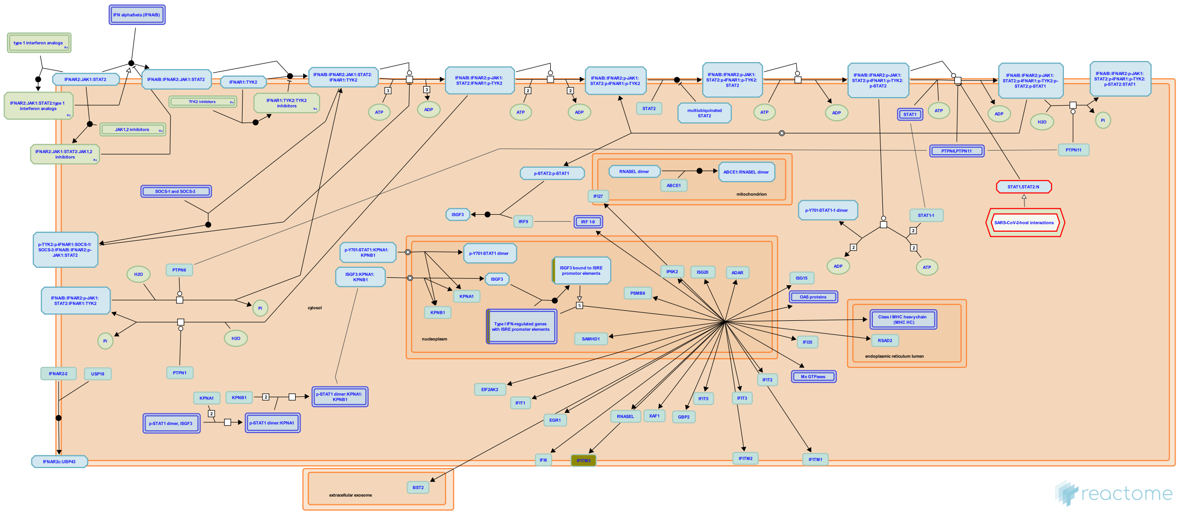

Type I interferons (IFNs) are composed of various genes including IFN alpha (IFNA), beta (IFNB), omega, epsilon, and kappa. In humans the IFNA genes are composed of more than 13 subfamily genes, whereas there is only one IFNB gene. The large family of IFNA/B proteins all bind to a single receptor which is composed of two distinct chains: IFNAR1 and IFNAR2. The IFNA/B stimulation of the IFNA receptor complex leads to the formation of two transcriptional activator complexes: IFNA-activated-factor (AAF), which is a homodimer of STAT1 and IFN-stimulated gene factor 3 (ISGF3), which comprises STAT1, STAT2 and a member of the IRF family, IRF9/P48. AAF mediates activation of the IRF-1 gene by binding to GAS (IFNG-activated site), whereas ISGF3 activates several IFN-inducible genes including IRF3 and IRF7.

### References


Stark GR, Darnell JE Jr, Qureshi S, Li X & Leung S (1996). Formation of STAT1-STAT2 heterodimers and their role in the activation of IRF-1 gene transcription by interferon-alpha. J Biol Chem, 271, 5790-4.

Gauzzi MC, Pellegrini S, Velazquez L, McKendry R, Fellous M & Mogensen KE (1996). Interferon-alpha-dependent activation of Tyk2 requires phosphorylation of positive regulatory tyrosines by another kinase. J Biol Chem, 271, 20494-500.

Pellegrini S, Piehler J, Schreiber G & Uzé G (2007). The receptor of the type I interferon family. Curr Top Microbiol Immunol, 316, 71-95.

Gupta S, Greenlund AC, Krolewski JJ, Yan H, Schreiber RD, Schindler CW, ... Krishnan K (1996). Phosphorylated interferon-alpha receptor 1 subunit (IFNaR1) acts as a docking site for the latent form of the 113 kDa STAT2 protein. EMBO J, 15, 1064-74.


### Edit history

| Date | Action | Author |
|---|---|---|
| 2010-07-07 | Edited | Garapati P V |
| 2010-07-07 | Authored | Garapati P V |
| 2010-07-07 | Created | Garapati P V |
| 2010-08-17 | Reviewed | Abdul-Sater AA, Schindler C |
| 2023-05-30 | Modified | Wright A |

### 1 submitted entities found in this pathway, mapping to 2 Reactome entities

| Input | UniProt Id |
|---|---|
| Ifitm3 | Q01628 |

| Input | Ensembl Id |
|---|---|
| Ifitm3 | ENSG00000142089 |

## 13. mRNA Editing: C to U Conversion (R-HSA-72200)

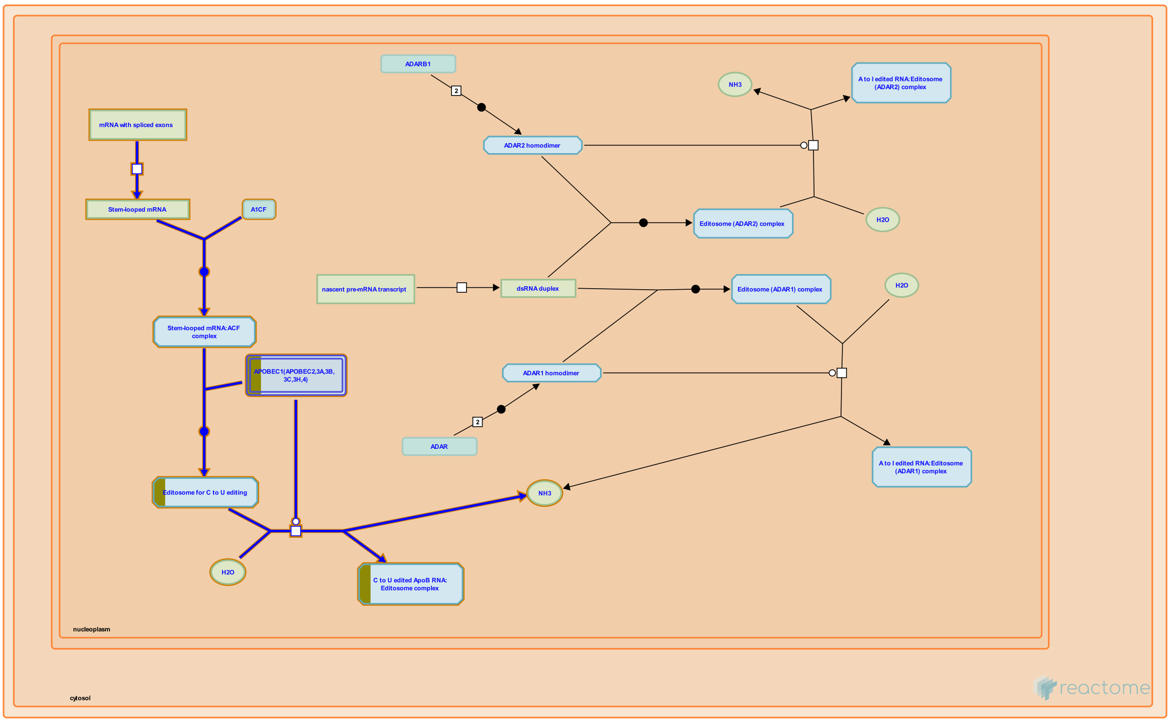


**Cellular compartments:** nucleoplasm.

The best characterized case of C to U editing is in the intestinal apolipoprotein B transcript, where the editing event creates a premature translation stop codon and consequently leads to a shorter form of the protein. In the liver, C to U editing is important in the expression of specific isoforms of the apolipoprotein B enzyme. ApoB mRNA editing is a posttranscriptional, nuclear process that can be initiated after splicing, at the time of polyadenylation and is completed by the time pre-mRNA matures fully (reviewed by Blanc and Davidson, 2003).

This editing event is a simple hydrolytic cytidine deamination to uridine, and is carried out by the Apobec-1 enzyme, along with the Apobec-1 complementing factor, ACF. The editing of apo-B mRNA involves the site-specific deamination of (C6666 to U), which converts codon 2153 from a glutamine codon, CAA, to a premature stop codon, UAA. As ACF is distributed in a variety of tissues, and these genes contain multiple family members, it is possible that editing events in additional targets will be found.

The cis-acting regulatory elements for C to U editing include: 22 nt editing site within ApoB mRNA, 5' tripartite motif with an enhancer element adjacent to the target cytidine, a spacer element and mooring sequence both 3' to the cytidine (reviewed by Smith et al., 1997).

### References


Smith HC, Dance GS, Sowden MP & Wedekind JE (2003). Messenger RNA editing in mammals: new members of the APOBEC family seeking roles in the family business. Trends Genet, 19, 207-16.

Davidson NO & Blanc V (2003). C-to-U RNA editing: mechanisms leading to genetic diversity. J Biol Chem, 278, 1395-8.

Emeson RB & Gott JM (2001). Functions and mechanisms of RNA editing. Annu Rev Genet, 34, 499-531.

Anant S, Navaratnam N, Scott J & Chester A (2000). RNA editing: cytidine to uridine conversion in apolipoprotein B mRNA. Biochim Biophys Acta, 1494, 1-13.

## Edit history

| Date | Action | Author |
| --- | --- | --- |
| 2003-08-22 | Created | Carmichael GG |
| 2023-05-21 | Modified | Wright A |

## 1 submitted entities found in this pathway, mapping to 1 Reactome entities

| Input | UniProt Id |
| --- | --- |
| Apobec1 | P41238 |

## 14. Caspase-mediated cleavage of cytoskeletal proteins (R-HSA-264870)

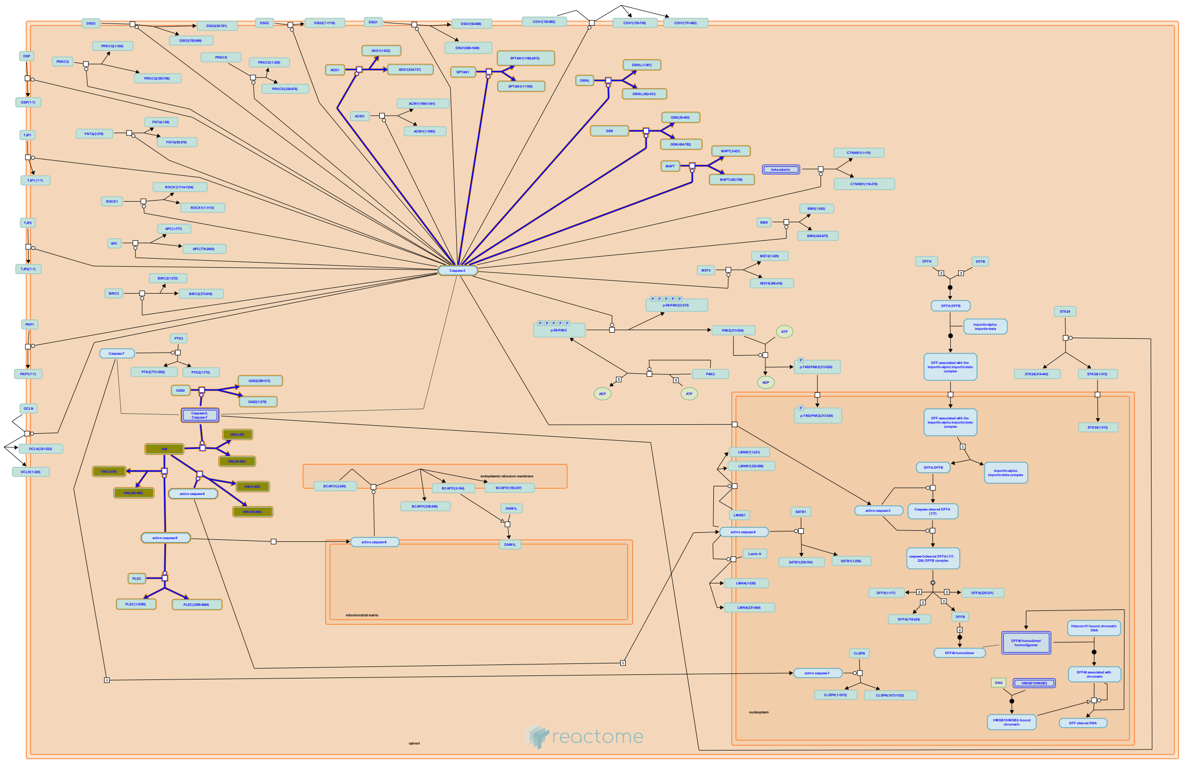

Caspase-mediated cleavage of a number of proteins in the cortical actin network ( ) microfilament system and others involved in maintenance of the cytoskeletal architecture (vimentin, or Gas2 and plectin) may directly contribute to apoptotic changes in cell shape.

### References


Tan TW, Ranganathan S & Wee LJ (2006). SVM-based prediction of caspase substrate cleavage sites. BMC Bioinformatics, 7, S14.

Janicke RU, Schulze-Osthoff K & Fischer U (2003). Many cuts to ruin: a comprehensive update of caspase substrates. Cell Death Differ, 10, 76-100.

Tan TW, Ranganathan S & Wee LJ (2007). CASVM: web server for SVM-based prediction of caspase substrates cleavage sites. Bioinformatics, 23, 3241-3.


### Edit history

| Date | Action | Author |
|---|---|---|
| 2007-09-03 | Authored | Schulze-Osthoff K |
| 2008-04-14 | Edited | Matthews L |
| 2008-04-14 | Created | Matthews L |
| 2008-06-11 | Reviewed | Ranganathan S |
| 2008-06-12 | Edited | Matthews L |
| 2023-05-30 | Modified | Wright A |

### 1 submitted entities found in this pathway, mapping to 1 Reactome entities

| Input | UniProt Id |
|---|---|
| Vim | P08670 |

## 15. mRNA Editing (R-HSA-75072)

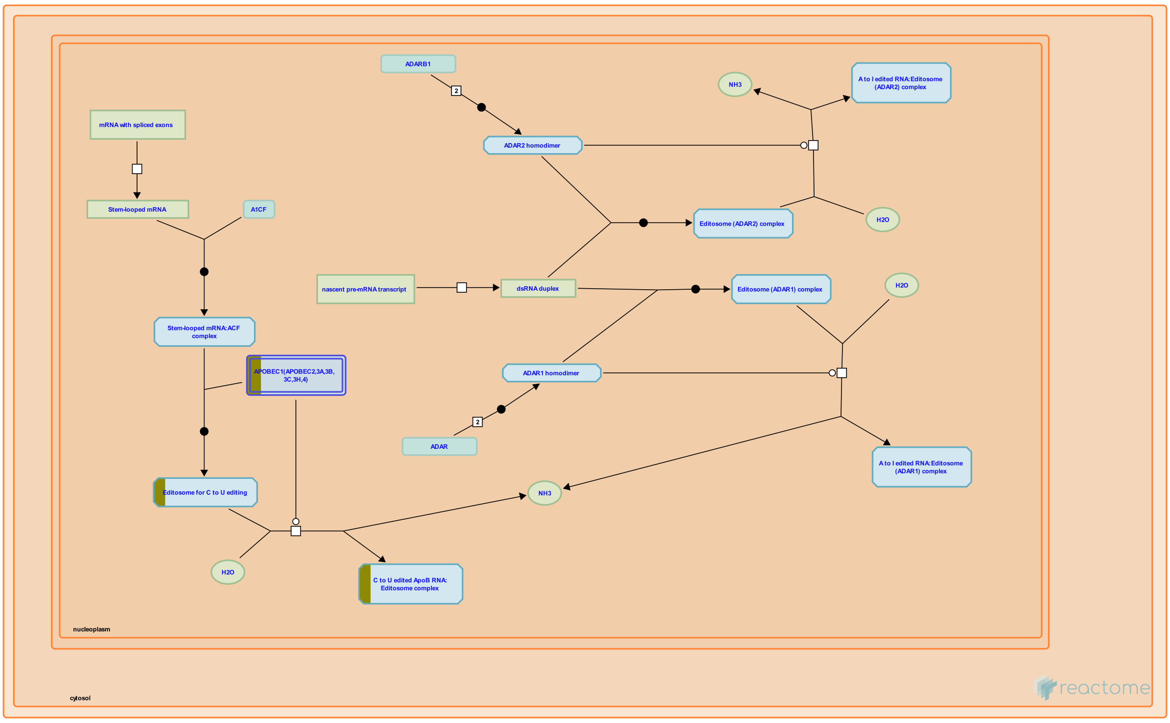


**Cellular compartments:** nucleoplasm.

After transcription, some RNA molecules are altered to contain bases not encoded in the genome. Most often this involves the editing or modification of one base to another, but in some organisms can involve the insertion or deletion of a base. Such editing events alter the coding properties of mRNA.

RNA editing can be generally defined as the co- or post transcriptional modification of the primary sequence of RNA from that encoded in the genome through nucleotide deletion, insertion, or base modification mechanisms.

There are two pathways of RNA editing: the substitution/conversion pathway and the insertion/deletion pathway. The insertion/deletion editing occurs in protozoans like Trypanosoma, Leishmania; in slime molds like Physarum spp., and in some viral categories like paramyxoviruses, Ebola virus etc. To date, the substitution/conversion pathway has been observed in human along with other mammals, Drosophila, and some plants. The RNA editing processes are known to create diversity in proteins involved in various pathways like lipid transport, metabolism etc. and may act as potential targets for therapeutic intervention (Smith et al., 1997).

The reaction mechanisms of cytidine and adenosine deaminases is represented below. In both these reactions, NH3 is presumed to be released:

### References


Emeson RB & Gott JM (2001). Functions and mechanisms of RNA editing. Annu Rev Genet, 34, 499-531.

Panigrahi AK & Stuart K (2002). RNA editing: complexity and complications. Mol Microbiol, 45, 591-6.

## Edit history

| Date | Action | Author |
|---|---|---|
| 2003-08-22 | Authored | Carmichael GG |
| 2003-08-22 | Created | Carmichael GG |
| 2023-05-19 | Edited | Gopinathrao G |
| 2023-05-21 | Modified | Wright A |

## 1 submitted entities found in this pathway, mapping to 1 Reactome entities

| Input | UniProt Id |
|---|---|
| Apobec1 | P41238 |

## 16. Golgi-to-ER retrograde transport (R-HSA-8856688)

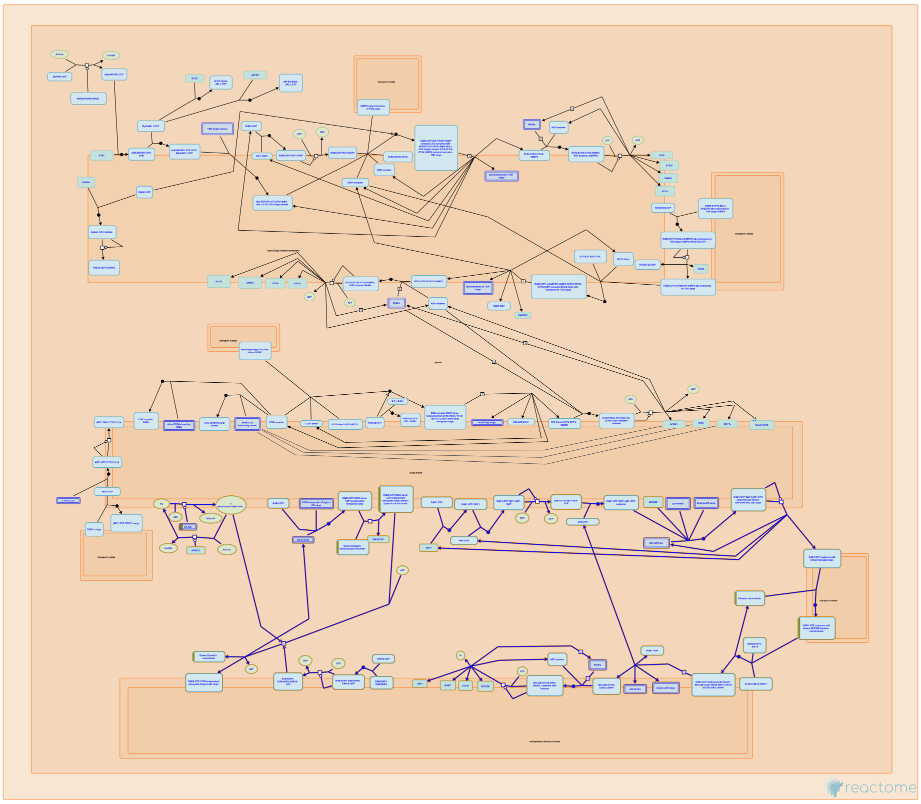

Retrograde traffic from the cis-Golgi to the ERGIC or the ER occurs through either COPI-coated vesicles or through a less well characterized RAB6-dependent route that makes use of tubular carriers (reviewed in Lord et al, 2013; Spang et al, 2013; Heffernan and Simpson, 2014). The balance between these two pathways may be influenced cargo type and concentration and membrane composition, though the details remain to be worked out (reviewed in Heffernan and Simpson, 2014).

### References


Spang A (2013). Retrograde traffic from the Golgi to the endoplasmic reticulum. Cold Spring Harb Perspect Biol, 5.

Ferro-Novick S, Miller EA & Lord C (2013). The highly conserved COPII coat complex sorts cargo from the endoplasmic reticulum and targets it to the golgi. Cold Spring Harb Perspect Biol, 5.

Simpson JC & Heffernan LF (2014). The trials and tubule-ations of Rab6 involvement in Golgi-to-ER retrograde transport. Biochem. Soc. Trans., 42, 1453-9.


### Edit history

| Date | Action | Author |
|---|---|---|
| 2016-01-06 | Edited | Rothfels K |
| 2016-01-06 | Authored | Rothfels K |
| 2016-02-02 | Reviewed | Gillespie ME |
| 2016-02-12 | Created | Rothfels K |
| 2023-05-21 | Modified | Wright A |

### 2 submitted entities found in this pathway, mapping to 2 Reactome entities

| Input | UniProt Id | Input | UniProt Id |
|---|---|---|---|
| Pla2g4a | P47712 | Tubb6 | Q9BUF5 |

## 17. Macroautophagy (R-HSA-1632852)

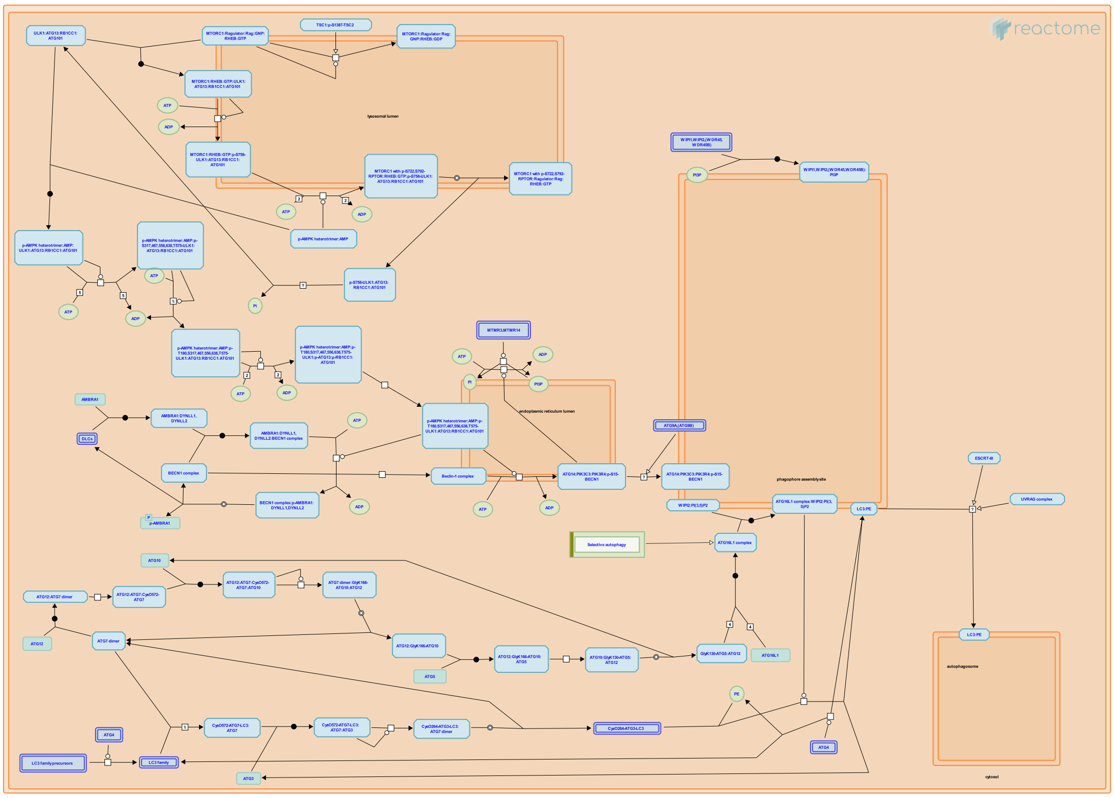

Macroautophagy (hereafter referred to as autophagy) acts as a buffer against starvation by liberating building materials and energy sources from cellular components. It has additional roles in embryonic development, removal of apoptotic cells or organelles, antigen presentation, protection against toxins and as a degradation route for aggregate-prone proteins and infectious agents. The dysregulation of autophagy is involved in several human diseases, for example, Crohn's disease, cancer and neurodegeneration (Ravikumar et al. 2010).

Autophagy is highly conserved from yeast to humans; much of the machinery was first identified in yeast (see Klionsky et al. 2011). Initially, double-membraned cup-shaped structures called the isolation membrane or phagophore engulf portions of cytoplasm. The membranes fuse to form the autophagosome. In yeast cells, autophagosomes are formed at the phagophore assembly site (PAS) next to the vacuole. In mammals, autophagosomes appear throughout the cytoplasm then move along microtubules towards the microtubule-organising centre. This transport requires microtubules and the function of dynein motor proteins; depolymerization of microtubules or inhibition of dynein-dependent transport results in inhibition of autophagy (Kochl et al. 2006, Kimura et al. 2008). Autophagosomes fuse with lysosomes forming autolysosomes whose contents are degraded by lysosomal hydrolases (Mizushima et al. 2011).

The origins of the autophagosomal membrane and the incorporation of existing membrane material have been extensively debated. The endoplasmic reticulum (ER), mitochondria, mitochondria-associated ER membranes (MAMs), the Golgi, the plasma membrane and recycling endosomes have all been implicated in the nucleation of the isolation membrane and subsequent growth of the membrane (Lamb et al. 2013). Recently 3D tomographic imaging of isolation membranes has shown the cup-shaped isolation membrane tightly sandwiched between two sheets of ER and physically connected to the ER through a narrow membrane tube (Hayashi-Nishino et al. 2009, Yla-Anttila et al. 2009). This suggests that isolation membrane formation and elongation are guided by adjacent ER sheets, supporting the now prevalent 'ER cradle' model, which suggests that the isolation membrane arises from the ER (Hayashi-Nishino et al. 2009, Shibutani & Yoshimori 2014).

Autophagy is tightly regulated. The induction of autophagy in response to starvation is partly mediated by inactivation of the mammalian target of rapamycin (mTOR) (Noda & Ohsumi 1998) and activation of Jun N-terminal kinase (JNK), while energy loss induces autophagy by activation of AMP kinase (AMPK). Other pathways regulating autophagy are regulated by calcium, cyclic AMP, calpains and the inositol trisphosphate (IP3) receptor (Rubinsztein et al. 2012).

In mammals, two complexes cooperatively produce the isolation membrane. The ULK complex consists of ULK1/2, ATG13, (FIP200) and ATG101 (Alers et al. 2012). The PIK3C3-containing Beclin-1 complex consists of PIK3C3 (Vps34), BECN1 (Beclin-1, Atg6), PIK3R4 (p150, Vps15) and ATG14 (Barkor) (Matsunaga et al. 2009, Zhong et al. 2009). A similar complex where ATG14 is replaced by UVRAG functions later in autophagosome maturation and endocytic traffic (Itakura et al. 2008, Liang et al. 2008). Binding of KIAA0226 to this complex negatively regulates the maturation process (Matsunaga et al. 2009). The ULK and Beclin-1 complexes are recruited to specific autophagosome nucleation regions where they stimulate phosphatidylinositol-3-phosphate (PI3P) production and facilitate the elongation and initial membrane curvature of the phagophore membrane (Carlsson & Simonsen 2015).

The ULK complex is considered the most upstream component of the mammalian autophagy pathway (Itakura & Mizushima 2010), acting as an integrator of the autophagy signals downstream of mTORC1. It is not fully understood how ULK1 is modulated in response to environmental cues. Phosphorylation plays an essential role (Dunlop & Tee 2013) but it is not clear how phosphorylation regulates ULK1 activities (Ravikumar et al. 2010). ULK1 kinase activity is required for autophagy, but the substrate(s) of ULK1 that mediate its autophagic function are not certain. ULK1 may also have kinase-independent functions in autophagy (Wong et al. 2013).

PIK3C3 (Vps34) is a class III phosphatidylinositol 3-kinase that produces PI3P. It is essential for the early stages of autophagy and colocalizes strongly with early autophagosome markers (Axe et al. 2008). BECN1 binds several further proteins that affect autophagosome formation. Partners that induce autophagy include AMBRA1 (Fimia et al. 2007), UVRAG (Liang et al. 2006) and SH3GLB1 (Takahashi et al. 2007). Binding of BCL2 or BCL2L1 (Bcl-xL) inhibit autophagy (Pattingre et al. 2005, Ciechomska et al. 2009). The inositol 1,4,5-trisphosphate receptor complex that binds BCL2 also interacts with BECN1, inhibiting autophagy (Vincencio et al. 2009). CISD2 (Nutrient-deprivation autophagy factor-1, NAF1), a component in the IP3R complex, interacts with BCL2 at the ER and stabilizes the BCL2-BECN1 interaction (Chang et al. 2010). Starvation leads to activation of c-Jun NH2-terminal kinase-1 (JNK1), which results in the phosphorylation of BCL2 and BCL2L1, which release their binding to BECN1 and thus induces autophagosome formation (Wei et al. 2008).

AMBRA1 can simultaneously bind dynein and the Beclin-1 complex. During nutrient starvation, AMBRA1 is phosphorylated in a ULK1-dependent manner (Di Bartolomeo et al. 2010). This phosphorylation releases AMBRA1-associated Beclin-1 complexes from dynein and the microtubule network, freeing the complex to translocate to autophagy initiation sites (Di Bartolomeo et al. 2010).

A characteristic of this early phase of autophagosome formation is the formation of PI3P-enriched ER-associated structures called omegasomes (Axe et al. 2008) or cradles (Hayashi-Nishino et al. 2009). Omegasomes appear to concentrate at or near the connected mitochondria-associated ER membrane (Hamasaki et al. 2013). However, the phagophore also can incorporate existing material from other membrane sources such as ER exit sites (ERES), the ER-Golgi intermediate compartment (ERGIC), the Golgi, the plasma membrane and recycling endosomes (Carlsson & Simonsen 2015). Omegasomes lead to the formation of the isolation membrane or phagophore, which is thought to form de novo by an unknown mechanism (Simonsen & Stenmark 2008, Roberts & Ktistakis 2013). Phagophore expansion is probably mediated by membrane uptake from endomembranes and semi-autonomous organelles (Lamb et al. 2013, Shibutani & Yoshimori 2014).

ATG9 is a direct target of ULK1. In nutrient-rich conditions mammalian ATG9 is localized to the trans-Golgi network and endosomes (including early, late and recycling endosomes), whereas under starvation conditions it is localized to autophagosomes, in a process that is dependent on ULK1 (Young et al. 2006). ATG9 is believed to play a role in the delivery of vesicles derived from existing membranes to the expanding phagophore (Lamb et al. 2013). Yeast Atg9 forms a complex with Atg2 and Atg18 (Reggiori et al. 2004).

PI3P produced at the initiation site is sensed by WIPI2b, the mammalian homologue of Atg18 (Polson et al. 2010). WIPI2b then recruits Atg16L1 (Dooley et al. 2014). There are four WIPI proteins in mammalian cells (Proikas-Cezanne et al. 2015). They are all likely bind PI3P and be recruited to membranes but the function of WIPI1, 3 and 4 in autophagy is not yet clear. WIPI4 (WDR45) has been shown to bind Atg2 and to be involved in lipid droplet formation (Velikkakath et al. 2012); mutations in WIPI4 have been shown to cause a neurodegenerative disease (Saitsu et al. 2013).

The elongation of the membrane that will become the autophagosome is regulated by two ubiquitination-like reactions. First, the ubiquitin-like molecule ATG12 is conjugated to ATG5 by ATG7, which acts as an E1-like activating enzyme, and ATG10, which has a role similar to an E2 ubiquitin-conjugating enzyme. The ATG5:ATG12 complex then interacts non-covalently with ATG16L1. This complex associates with the forming autophagosome but dissociates from completed autophagosomes (Geng & Klionski 2008). The second ubiquitin-like reaction involves the conjugation of ubiquitin-like molecules of the LC3 family (Weidberg et al. 2010). LC3 proteins are conjugated through their C-terminal glycine residues with PE by the E1-like ATG7 and E2-like ATG3. This allows LC3 proteins to associate with the autophagosome membrane.

The ATG12:ATG5:ATG16L1 complex (Mizushima et al., 2011) acts as an E3 like enzyme for the conjugation of LC3 family proteins (mammalian homologues of yeast Atg8) to phosphatidylethanolamine (PE) (Hanada et al. 2007, Fujita et al. 2008). LC3 PE can be deconjugated by the protease ATG4 (Li et al. 2011, 2012). ATG4 is also responsible for priming LC3 proteins by cleaving the C terminus to expose a glycine residue (Kirisako et al, 2000, Scherz Shouval et al. 2007). LC3 proteins remain associated with autophagosomes until they fuse with lysosomes. The LC3-like proteins inside the resulting autolysosomes are degraded, while those on the cytoplasmic surface are delipidated and recycled. ATG5:ATG12:ATG16L1-positive LC3-negative vesicles represent pre-autophagosomal structures (pre-phagophores and possibly early phagophores), ATG5:ATG12:ATG16L1-positive LC3-positive structures can be considered to be phagophores, and ATG5:ATG12:ATG16L1-negative LC3-positive vesicles can be regarded as mature autophagosomes (Tandia et al. 2011).

Phagophore expansion is probably mediated by membrane uptake from endomembranes as well as from semiautonomous organelles (Lamb et al. 2013, Shibutani & Yoshimori 2014).

The mechanisms involved in the closure of the phagophore membrane are poorly understood. As the phagophore is a double-membraned structure, its closure involves the fusion of a narrow opening, a process that is distinct from other membrane fusion events (Carlsson & Simonsen 2015). The topology of the phagophore is similar to that of cytokinesis, viral budding or multivesicular body (MVB) formation. These processes rely on the Endosomal Sorting Complex Required for Transport (ESCRT) (Rusten et al. 2012). ESCRT and associated proteins facilitate membrane budding away from the cytosol and subsequent cleavage of the bud neck (Hurley & Hanson 2010). Several studies have shown that depletion of ESCRT subunits or the regulatory ATPase Vps4 causes an accumulation of autophagosomes (Filimonenko et al. 2007, Rusten et al. 2007) but it is not clear whether ESCRTs are required for autophagosome closure or for autophagosome to endosome fusion. UVRAG is also involved in the maturation step, recruiting proteins that bring about membrane fusion such as the class C Vps proteins, which activate Rab7 thereby promoting fusion with late endosomes and lysosomes (Liang et al. 2008).

## References

Shibutani ST & Yoshimori T (2014). A current perspective of autophagosome biogenesis. Cell Res., 24, 58-68.

## Edit history

| Date | Action | Author |
|---|---|---|
| 2011-10-05 | Created | Jassal B |
| 2015-02-02 | Authored | Jupe S |
| 2015-05-13 | Edited | Jupe S |
| 2019-03-05 | Revised | Varusai TM |
| 2019-10-31 | Revised | Varusai TM |
| 2023-05-21 | Modified | Wright A |

## 2 submitted entities found in this pathway, mapping to 2 Reactome entities

| Input | UniProt Id | Input | UniProt Id |
|---|---|---|---|
| Tubb6 | Q9BUF5 | Vim | P08670 |

## 18. Autophagy (R-HSA-9612973)

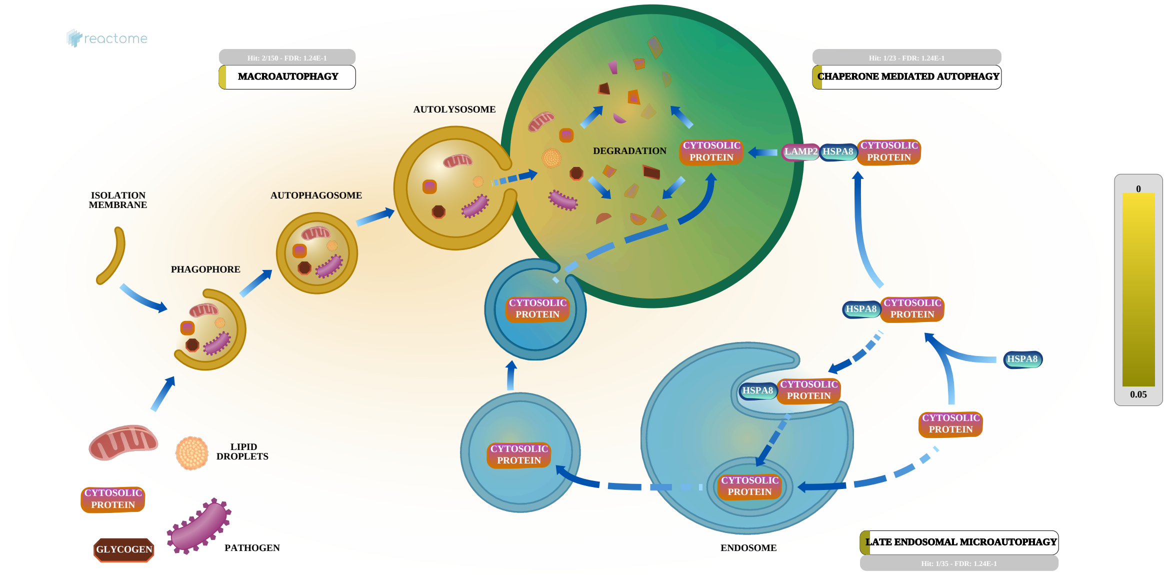


Autophagy is an intracellular degradation process that is triggered by cellular stresses. There are three primary types of autophagy - macroautophagy, chaperone-mediated autophagy (CMA) and late endosomal microautophagy. Despite being morphologically distinct, all three processes culminate in the delivery of cargo to the lysosome for degradation and recycling (Parzych KR et al, 2014). In macroautophagy a double membrane compartment sequesters the cargo and delivers it to the lysosome. Chaperones are used to deliver specific cargo proteins to the lysosome in CMA. In microautophagy invaginations of the endosomal membrane are used to capture cargo from the cytosol. Autophagy can target a wide range of entities ranging from bulk proteins and lipids to cell organelles and pathogens giving rise to several subclasses such as mitophagy, lipophagy, xenophagy, etc. (Shibutani ST 2014 et al).

### References


Shibutani ST & Yoshimori T (2014). A current perspective of autophagosome biogenesis. Cell Res., 24, 58-68.

Parzych KR & Klionsky DJ (2014). An overview of autophagy: morphology, mechanism, and regulation. Antioxid. Redox Signal., 20, 460-73.


### Edit history

| Date | Action | Author |
|---|---|---|
| 2015-02-02 | Authored | Jupe S |
| 2015-05-13 | Edited | Jupe S |
| 2018-07-05 | Created | Varusai TM |
| 2019-02-21 | Authored | Varusai TM |
| 2023-05-21 | Modified | Wright A |

### 2 submitted entities found in this pathway, mapping to 2 Reactome entities

| Input | UniProt Id | Input | UniProt Id |
|---|---|---|---|
| Tubb6 | Q9BUF5 | Vim | P08670 |

## 19. Arachidonic acid metabolism (R-HSA-2142753)

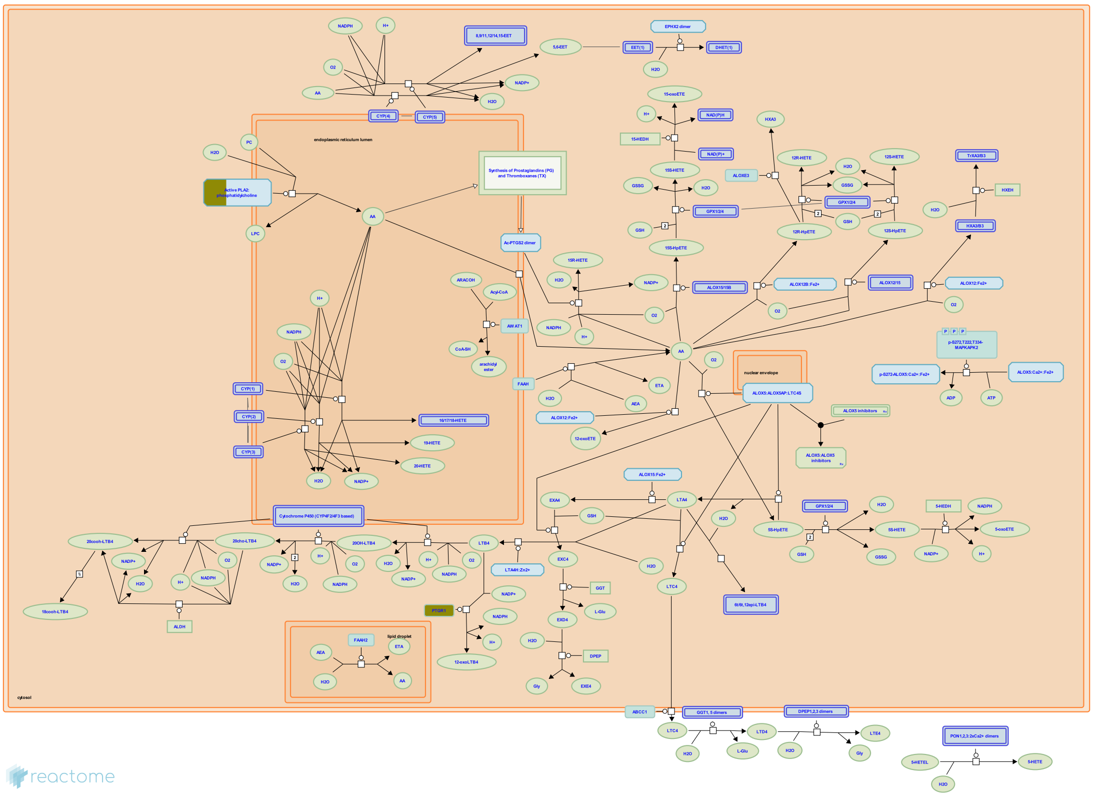

Eicosanoids, oxygenated, 20-carbon fatty acids, are autocrine and paracrine signaling molecules that modulate physiological processes including pain, fever, inflammation, blood clot formation, smooth muscle contraction and relaxation, and the release of gastric acid. Eicosanoids are synthesized in humans primarily from arachidonic acid (all-cis 5,8,11,14-eicosatetraenoic acid) that is released from membrane phospholipids. Once released, arachidonic acid is acted on by prostaglandin G/H synthases (PTGS, also known as cyclooxygenases (COX)) to form prostaglandins and thromboxanes, by arachidonate lipoxygenases (ALOX) to form leukotrienes, epoxygenases (cytochrome P450s and epoxide hydrolase) to form epoxides such as 15-eicosatetraenoic acids, and omega-hydrolases (cytochrome P450s) to form hydroxyeicosatetraenoic acids (Buczynski et al. 2009, Vance & Vance 2008).

Levels of free arachidonic acid in the cell are normally very low so the rate of synthesis of eicosanoids is determined primarily by the activity of phospholipase A2, which mediates phospholipid cleavage to generate free arachidonic acid. The enzymes involved in arachidonic acid metabolism are typically constitutively expressed so the subset of these enzymes expressed by a cell determines the range of eicosanoids it can synthesize.

Eicosanoids are unstable, undergoing conversion to inactive forms with half-times under physiological conditions of seconds or minutes. Many of these reactions appear to be spontaneous.

### References


Vance JE & Vance DE (2008). *The eicosanoids: cyclooxygenase, lipoxygenase, and epoxygenase pathways, Biochemistry of Lipids, Lipoproteins and Membranes, 5th Edition* , 331-362.

Dumlao DS, Buczynski MW & Dennis EA (2009). Thematic Review Series: Proteomics. An integrated omics analysis of eicosanoid biology. J Lipid Res, 50, 1015-38.

## Edit history

| Date | Action | Author |
|---|---|---|
| 2012-02-24 | Edited | Williams MG |
| 2012-02-24 | Authored | Williams MG |
| 2012-02-24 | Created | Williams MG |
| 2012-11-10 | Reviewed | Rush MG |
| 2023-05-21 | Modified | Wright A |

## 2 submitted entities found in this pathway, mapping to 2 Reactome entities

| Input | UniProt Id | Input | UniProt Id |
|---|---|---|---|
| Pla2g4a | P47712 | Ptgr1 | Q14914 |

## 20. Interferon gamma signaling (R-HSA-877300)

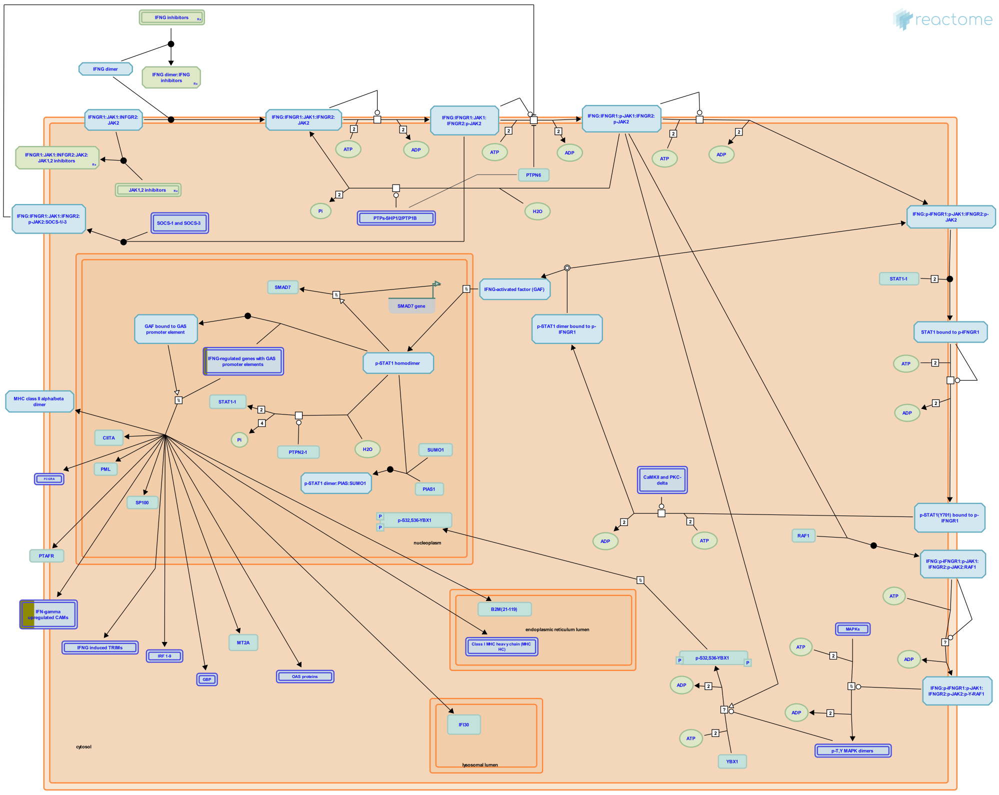

Interferon-gamma (IFN-gamma) belongs to the type II interferon family and is secreted by activated immune cells-primarily T and NK cells, but also B-cells and APC. INFG exerts its effect on cells by interacting with the specific IFN-gamma receptor (IFNGR). IFNGR consists of two chains, namely IFNGR1 (also known as the IFNGR alpha chain) and IFNGR2 (also known as the IFNGR beta chain). IFNGR1 is the ligand binding receptor and is required but not sufficient for signal transduction, whereas IFNGR2 do not bind IFNG independently but mainly plays a role in IFNG signaling and is generally the limiting factor in IFNG responsiveness. Both IFNGR chains lack intrinsic kinase/phosphatase activity and thus rely on other signaling proteins like Janus-activated kinase 1 (JAK1), JAK2 and Signal transducer and activator of transcription 1 (STAT-1) for signal transduction. IFNGR complex in its resting state is a preformed tetramer and upon IFNG association undergoes a conformational change. This conformational change induces the phosphorylation and activation of JAK1, JAK2, and STAT1 which in turn induces genes containing the gamma-interferon activation sequence (GAS) in the promoter.

### References


Schroder K, Ravasi T, Hume DA & Hertzog PJ (2004). Interferon-gamma: an overview of signals, mechanisms and functions. J Leukoc Biol, 75, 163-89.

Aguet M, Bach EA & Schreiber RD (1997). The IFN gamma receptor: a paradigm for cytokine receptor signaling. Annu Rev Immunol, 15, 563-91.

Gough DJ, Levy DE, Clarke CJ & Johnstone RW (2008). IFNgamma signaling-does it mean JAK-STAT?. Cytokine Growth Factor Rev, 19, 383-94.

Izotova LS, Garotta G, Muthukumaran G, Kotenko SV, Cook JR & Pestka S (1997). The interferon gamma (IFN-gamma) receptor: a paradigm for the multichain cytokine receptor. Cytokine Growth Factor Rev, 8, 189-206.

## Edit history

| Date | Action | Author |
|---|---|---|
| 2010-06-08 | Edited | Garapati P V |
| 2010-06-08 | Authored | Garapati P V |
| 2010-06-11 | Created | Garapati P V |
| 2010-08-17 | Reviewed | Abdul-Sater AA, Schindler C |
| 2023-05-30 | Modified | Wright A |

## 1 submitted entities found in this pathway, mapping to 2 Reactome entities

| Input | UniProt Id |
|---|---|
| Icam1 | P05362 |

| Input | Ensembl Id |
|---|---|
| Icam1 | ENSG00000090339 |

## 21. Hydrolysis of LPC (R-HSA-1483115)

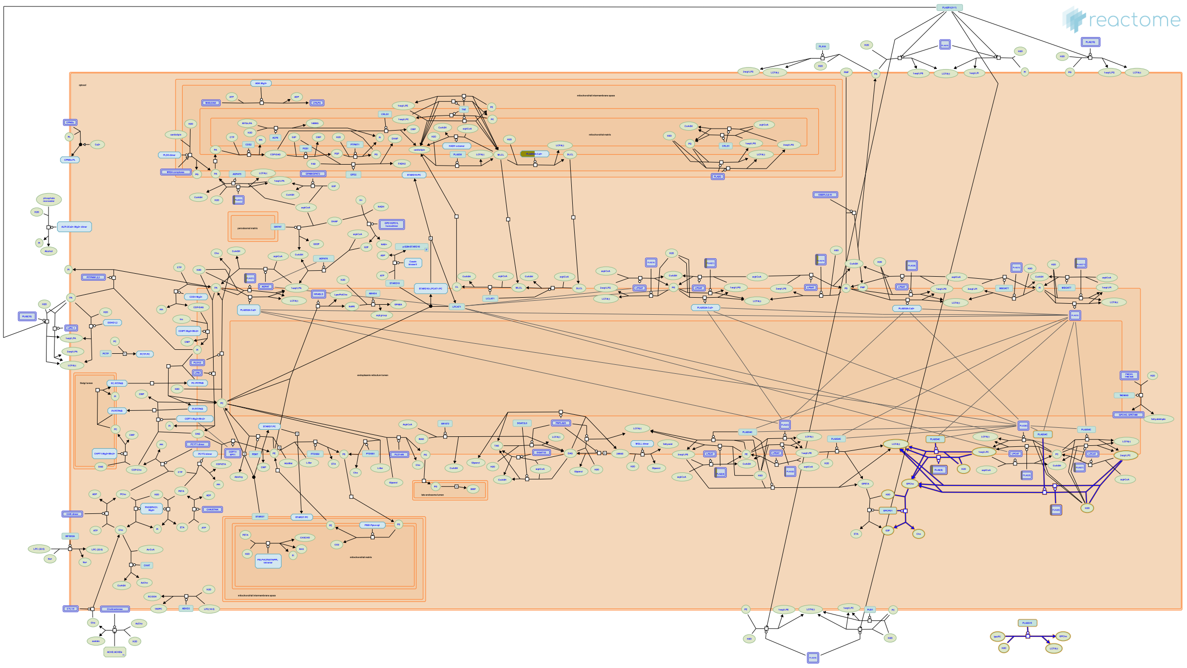

Lysophosphatidylcholine (LPC) is hydrolyzed by phospholipases to produce glycerophosphocholine (GPCho) which is in turn hydrolyzed by glycerophosphocholine phosphodiesterase to produce choline (Cho) and glycerol-3-phosphate (G3P) (Yamashita et al. 2009, Yamashita et al. 2005, Ghomashchi et al. 2010).

### References


Lehr M, Gelb MH, Aloulou A, Naika GS, Bollinger JG, Ghomashchi F & Leslie CC (2010). Interfacial kinetic and binding properties of mammalian group IVB phospholipase A2 (cPLA2beta) and comparison with the other cPLA2 isoforms. J Biol Chem, 285, 36100-11.

Waku K, Nakanishi H, Yamashita A, Kawagishi N, Kamata R, Sugiura T & Suzuki H (2005). Roles of C-terminal processing, and involvement in transacylation reaction of human group IVC phospholipase A2 (cPLA2gamma). J Biochem, 137, 557-67.

Waku K, Tanaka K, Kumazawa T, Yamashita A, Kamata R, Sugiura T, ... Suzuki N (2009). Subcellular localization and lysophospholipase/transacylation activities of human group IVC phospholipase A2 (cPLA2gamma). Biochim Biophys Acta, 1791, 1011-22.


### Edit history

| Date | Action | Author |
|---|---|---|
| 2011-08-12 | Edited | Williams MG |
| 2011-08-12 | Created | Williams MG |
| 2011-09-14 | Authored | Williams MG |
| 2012-05-14 | Reviewed | Wakelam M |
| 2023-05-21 | Modified | Wright A |

### 1 submitted entities found in this pathway, mapping to 1 Reactome entities

| Input | UniProt Id |
|---|---|
| Pla2g4a | P47712 |

## 22. Acyl chain remodeling of CL (R-HSA-1482798)

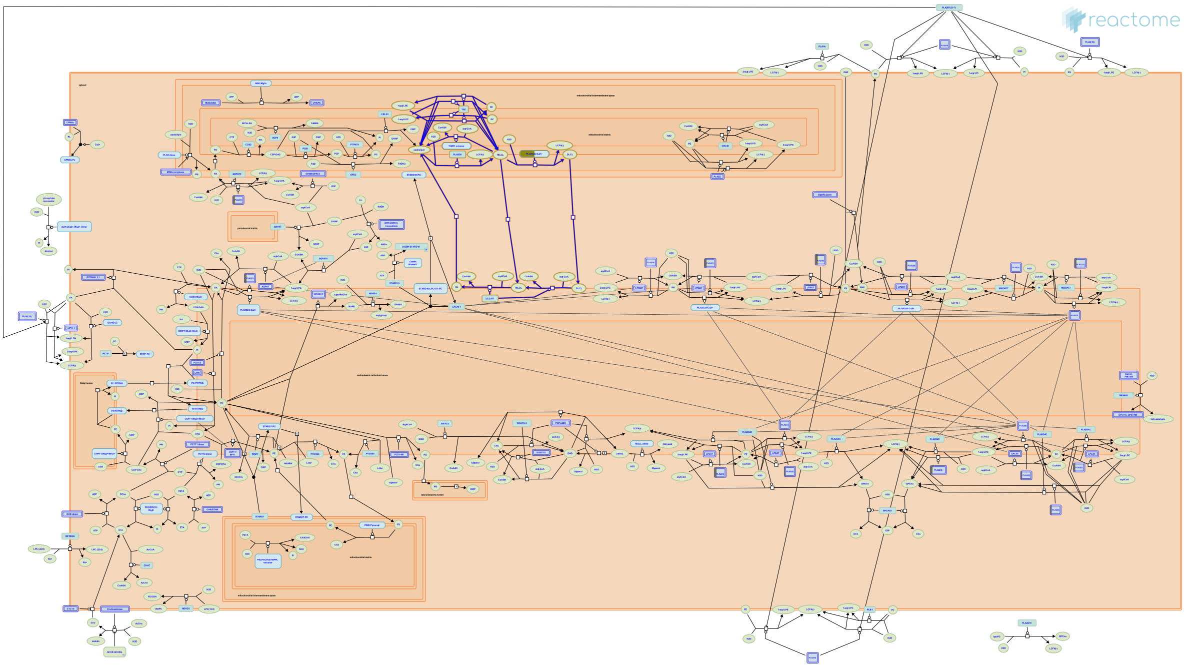

Acyl chain remodeling of cardiolipin (CL) occurs in the inner mitochondria membranes (IM) via hydrolysis by phopholipases and subsequent reacylation by acyltransferases. At the endoplasmic reticulum (ER) membrane the situation is more complicated with monolysocardiolipin (MLCL) involved in hydrolysis and subsequent reacylation back to CL (Zachman et al. 2010, Malhotra et al. 2009, Xu et al. 2003, Taylor & Hatch 2009, Cao et al. 2004, Zhao et al. 2009, Buckland et al. 1998).

### References


Ren M, Schlame M, Xu Y & Malhotra A (2009). Formation of molecular species of mitochondrial cardiolipin. 1. A novel transacylation mechanism to shuttle fatty acids between sn-1 and sn-2 positions of multiple phospholipid species. Biochim Biophys Acta, 1791, 314-20.

Cao G, Li S, Chen YQ, Zhao Y & Konrad RJ (2009). The microsomal cardiolipin remodeling enzyme acyl-CoA lysocardiolipin acyltransferase is an acyltransferase of multiple anionic lysophospholipids. J Lipid Res, 50, 945-56.

Shi Y, Lockwood J, Liu Y, Burn P & Cao J (2004). A novel cardiolipin-remodeling pathway revealed by a gene encoding an endoplasmic reticulum-associated acyl-CoA:lysocardiolipin acyltransferase (ALCAT1) in mouse. J Biol Chem, 279, 31727-34.

McCune SA, Sparagna GC, Murphy RC, Zachman DK, Moore RL & Chicco AJ (2010). The role of calcium-independent phospholipase A2 in cardiolipin remodeling in the spontaneously hypertensive heart failure rat heart. J Lipid Res, 51, 525-34.

Taylor WA & Hatch GM (2009). Identification of the human mitochondrial linoleoyl-coenzyme A monolysocardiolipin acyltransferase (MLCL AT-1). J Biol Chem, 284, 30360-71.


### Edit history

| Date | Action | Author |
|---|---|---|
| 2011-08-12 | Edited | Williams MG |
| 2011-08-12 | Created | Williams MG |

| Date | Action | Author |
| --- | --- | --- |
| 2011-09-14 | Authored | Williams MG |
| 2023-05-21 | Modified | Wright A |

## 1 submitted entities found in this pathway, mapping to 1 Reactome entities

| Input | UniProt Id |
| --- | --- |
| Pla2g4a | P47712 |

## 23. Cell-extracellular matrix interactions (R-HSA-446353)

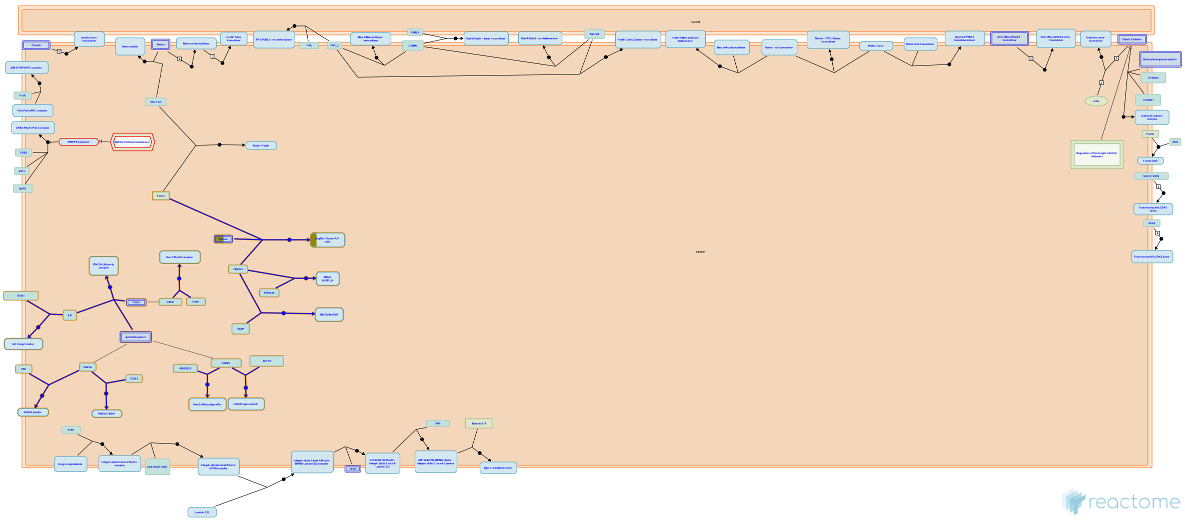

Cell-extracellular matrix (ECM) interactions play a critical role in regulating a variety of cellular processes in multicellular organisms including motility, shape change, survival, proliferation and differentiation. Cell-ECM contact is mediated by transmembrane cell adhesion receptors, such as integrins, that interact with extracellular matrix proteins as well as a number of cytoplasmic adaptor proteins. Many of these adaptor proteins physically interact with the actin cytoskeleton or function in signal transduction.

Several protein complexes interact with the cytoplasmic tail of integrins and function in transducing bi-directional signals between the ECM and intracellular signaling pathways (reviewed in Sepulveda et al., 2005).

Early events that are triggered by interactions with ECM, such as formation/turnover of Focal Adhesions, regulation of actin dynamics and protrusion of lamellipodia to promote cellular spreading and motility are modulated by PINCH- ILK- parvin complexes (see Sepulveda et al., 2005). A number of partners of the PINCH-ILK-parvin complex components have been identified that regulate and/or mediate the functions of these complexes (reviewed in Wu, 2004). Interactions with some of these partners modulate cytoskeletal remodeling and cell spreading.

### References


Wu C (2004). The PINCH-ILK-parvin complexes: assembly, functions and regulation. Biochim Biophys Acta, 1692, 55-62.


### Edit history

| Date | Action | Author |
|---|---|---|
| 2009-10-12 | Authored | Matthews L |
| 2009-11-10 | Edited | Matthews L |
| 2009-11-12 | Reviewed | Wu C |
| 2009-11-12 | Created | Matthews L |
| 2023-05-21 | Modified | Wright A |

### 1 submitted entities found in this pathway, mapping to 1 Reactome entities

| Input | UniProt Id |
|---|---|
| Flnc | Q14315 |

## 24. Microtubule-dependent trafficking of connexons from Golgi to the plasma membrane (R-HSA-190840)

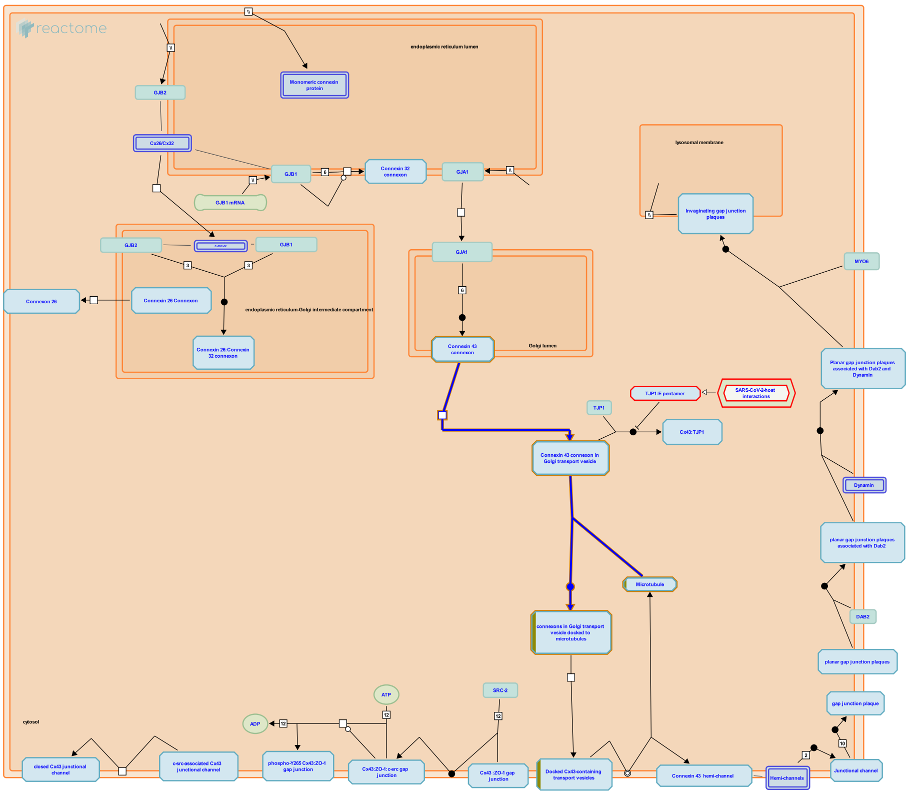

**Cellular compartments:** cytosol.

Through videomicroscopy, a saltatory transport of connexon vesicles along curvilinear microtubules from the Golgi to the plasma membrane has been observed (Lauf et al., 2002). Such a transport system has been described for similar secretory vesicles (Toomre et al., 1999).

### References


Braconnot S, Lauf U, Chen SC, Giepmans BN, Lopez P & Falk MM (2002). Dynamic trafficking and delivery of connexons to the plasma membrane and accretion to gap junctions in living cells. Proc Natl Acad Sci U S A, 99, 10446-51.


### Edit history

| Date | Action | Author |
|---|---|---|
| 2007-01-03 | Authored | Gilleron J, Segretain D, Falk MM |
| 2007-01-09 | Created | Matthews L |
| 2007-04-12 | Edited | Matthews L |
| 2023-05-21 | Modified | Wright A |

### 1 submitted entities found in this pathway, mapping to 1 Reactome entities

| Input | UniProt Id |
|---|---|
| Tubb6 | Q9BUF5 |

## 25. Chaperone Mediated Autophagy (R-HSA-9613829)

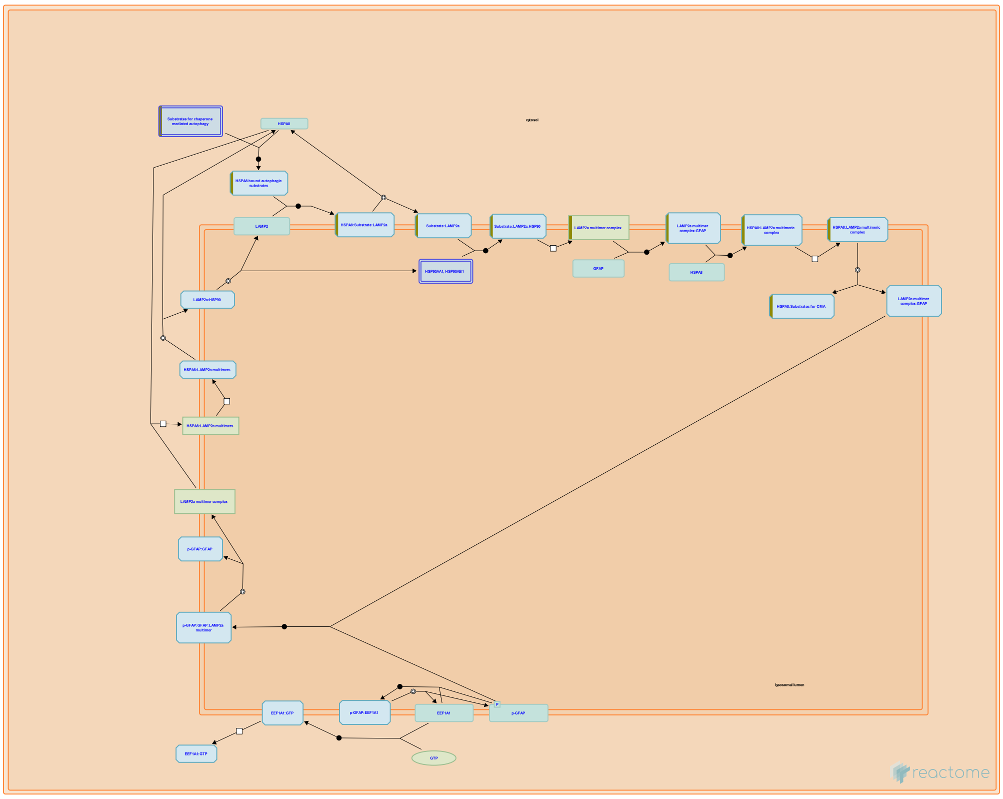

**Cellular compartments:** lysosomal lumen, cytosol, lysosomal membrane.

In contrary to the vesicle-mediated macroautophagy, the chaperone mediated mechanism of autophagy selectively targets individual proteins to the lysosome for degradation. Chaperones bind intracellular proteins based on recognition motifs and transports them from the cytosol to the lysosomal membrane. Subsequently, the protein is translocated into the lumen for digestion (Cuervo A M et al. 2014, Kaushik S et al. 2018).

### References


Cuervo AM & Wong E (2014). Chaperone-mediated autophagy: roles in disease and aging. Cell Res., 24, 92-104.


### Edit history

| Date | Action | Author |
| --- | --- | --- |
| 2018-07-19 | Created | Varusai TM |
| 2019-02-21 | Authored | Varusai TM |
| 2019-02-22 | Reviewed | Metzakopian E |
| 2019-10-31 | Revised | Varusai TM |
| 2019-11-08 | Edited | Varusai TM |
| 2023-05-30 | Modified | Wright A |

### 1 submitted entities found in this pathway, mapping to 1 Reactome entities

| Input | UniProt Id |
| --- | --- |
| Vim | P08670 |

# 6. Identifiers found

Below is a list of the input identifiers that have been found or mapped to an equivalent element in Reactome, classified by resource.

## 11 of the submitted entities were found, mapping to 15 Reactome entities

| Input | UniProt Id | Input | UniProt Id | Input | UniProt Id |
|---|---|---|---|---|---|
| Apobec1 | P41238 | Flnc | Q14315 | Icam1 | P05362 |
| Ifitm3 | Q01628 | Pla2g4a | P47712 | Prss23 | O95084 |
| Ptgr1 | Q14914 | Rab32 | P57729, Q13637 | Slc38a5 | Q8WUX1 |
| Tubb6 | Q9BUF5 | Vim | P08670 | | |

| Input | Ensembl Id | Input | Ensembl Id | Input | Ensembl Id |
|---|---|---|---|---|---|
| Icam1 | ENSG00000090339 | Ifitm3 | ENSG00000142089 | Vim | ENSG00000026025 |

# 7. Identifiers not found

These 6 identifiers were not found neither mapped to any entity in Reactome.

| | | | | | |
|---|---|---|---|---|---|
| 5830408C22Rik | Dclk1 | Lsp1 | Myo1g | Rfx3 | Slfn5 |
