# Supplementary material for: Sex- and region-specific cortical and hippocampal whole genome transcriptome profiles from control and APP/PS1 Alzheimer’s disease mice
Source: PLoS One. 2024 Feb 7;19(2):e0296959. doi: 10.1371/journal.pone.0296959 (PMC10849391; doi:10.1371/journal.pone.0296959)
Supplement: S1 File — S1 Fig: Genotyping of APP/PS1 AD mice and WT control animals. S2 Fig: 3D image of the murine brain including the RS cortex and hippocampus (BROIs) used for transcriptome analysis in our study. S3 Fig: PCA of transcriptomes from the RS cortex and hippocampus of WT controls and APP/PS1 AD mice of both sexes. S4 Fig: Hierarchical clustering of transcriptome data from the RS cortex and hippocampus of WT control and APP/PS1 AD mice of both sexes. S5 Fig: Bar diagrams of the top 30 candidates of DEGs with highest significant FCs (FC > 1.5 and FC < -1.5, p < 0.05). S6 Fig: Pathway analysis of intersectional and signature gene sets in APP/PS1 subgroups. S7 Fig: Comparative qPCR analysis of selected gene transcript levels from the hippocampus of female and male APP/PS1 AD with 5XFAD mice. S1 Table: PCR reaction set-up using PCR Mastermix and genomic DNA. S2 Table: Materials used for one-color microarray-based gene expression data collection. S3 Table: Software used for one-color microarray-based gene expression data collection. S4 Table: Details on genes, forward and reverse primer sequences and annealing temperatures relevant for qPCR experimentation. S5 Table: Characteristics of DEGs in the RS cortex of female APP/PS1 AD mice. S6 Table: Characteristics of DEGs in the hippocampus of female APP/PS1 AD mice. S7 Table: Characteristics of DEGs in the RS cortex of male APP/PS1 AD mice. S8 Table: Characteristics of DEGs in the hippocampus of male APP/PS1 AD mice. S9 Table: Venn analysis of DEGs in the RS cortex and hippocampus of female APP/PS1 AD mice. S10 Table: Venn analysis of DEGs genes in the RS cortex and hippocampus of male APP/PS1 AD mice. S11 Table: Venn analysis of DEGs in the RS cortex of male and female APP/PS1 AD mice. S12 Table: Venn analysis of DEGs in the hippocampus of male and female APP/PS1 AD mice. S13 Table: Differentially regulated l(i)ncRNAs in APP/PS1 AD vs. WT mice. S14 Table: qPCR-based FC analysis of selected genes in the hippocampus of APP/PS1 AD vs. [file pone.0296959.s001.zip › Supplementary Files_R1/Supplementary Figure 6_Pathways_upreg genes/Signature genes up_DEGs_female_Rs Cx_APPPS1/Pathway analysis report.pdf]

reactome

# Pathway Analysis Report

This report contains the pathway analysis results for the submitted sample ''. Analysis was performed against Reactome version 85 on 11/08/2023. The web link to these results is:

https://reactome.org/PathwayBrowser/#/ANALYSIS=MjAyMzA4MTExNDQ2MjdfNzYwMQ%3D%3D

Please keep in mind that analysis results are temporarily stored on our server. The storage period depends on usage of the service but is at least 7 days. As a result, please note that this URL is only valid for a limited time period and it might have expired.

# Table of Contents

1. Introduction
2. Properties
3. Genome-wide overview
4. Most significant pathways
5. Pathways details
6. Identifiers found
7. Identifiers not found

# 1. Introduction

Reactome is a curated database of pathways and reactions in human biology. Reactions can be considered as pathway 'steps'. Reactome defines a 'reaction' as any event in biology that changes the state of a biological molecule. Binding, activation, translocation, degradation and classical biochemical events involving a catalyst are all reactions. Information in the database is authored by expert biologists, entered and maintained by Reactome's team of curators and editorial staff. Reactome content frequently cross-references other resources e.g. NCBI, Ensembl, UniProt, KEGG (Gene and Compound), ChEBI, PubMed and GO. Orthologous reactions inferred from annotation for Homo sapiens are available for 14 non-human species including mouse, rat, chicken, puffer fish, worm, fly and yeast. Pathways are represented by simple diagrams following an SBGN-like format.

Reactome's annotated data describe reactions possible if all annotated proteins and small molecules were present and active simultaneously in a cell. By overlaying an experimental dataset on these annotations, a user can perform a pathway over-representation analysis. By overlaying quantitative expression data or time series, a user can visualize the extent of change in affected pathways and its progression. A binomial test is used to calculate the probability shown for each result, and the p-values are corrected for the multiple testing (Benjamini–Hochberg procedure) that arises from evaluating the submitted list of identifiers against every pathway.

To learn more about our Pathway Analysis, please have a look at our relevant publications:


Fabregat A, Sidiropoulos K, Garapati P, Gillespie M, Hausmann K, Haw R, … D'Eustachio P (2016). The reactome pathway knowledgebase. Nucleic Acids Research, 44(D1), D481–D487. https://doi.org/10.1093/nar/gkv1351.

Fabregat A, Sidiropoulos K, Viteri G, Forner O, Marin-Garcia P, Arnau V, … Hermjakob H (2017). Reactome pathway analysis: a high-performance in-memory approach. BMC Bioinformatics, 18.

## 2. Properties

- This is an **overrepresentation** analysis: A statistical (hypergeometric distribution) test that determines whether certain Reactome pathways are over-represented (enriched) in the submitted data. It answers the question 'Does my list contain more proteins for pathway X than would be expected by chance?' This test produces a probability score, which is corrected for false discovery rate using the Benjamani-Hochberg method.
- 17 out of 25 identifiers in the sample were found in Reactome, where 283 pathways were hit by at least one of them.
- All non-human identifiers have been converted to their human equivalent.
- This report is filtered to show only results for species 'Homo sapiens' and resource 'all resources'.
- The unique ID for this analysis (token) is MjAyMzA4MTExNDQ2MjdfNzYwMQ%3D%3D. This ID is valid for at least 7 days in Reactome's server. Use it to access Reactome services with your data.

# 3. Genome-wide overview

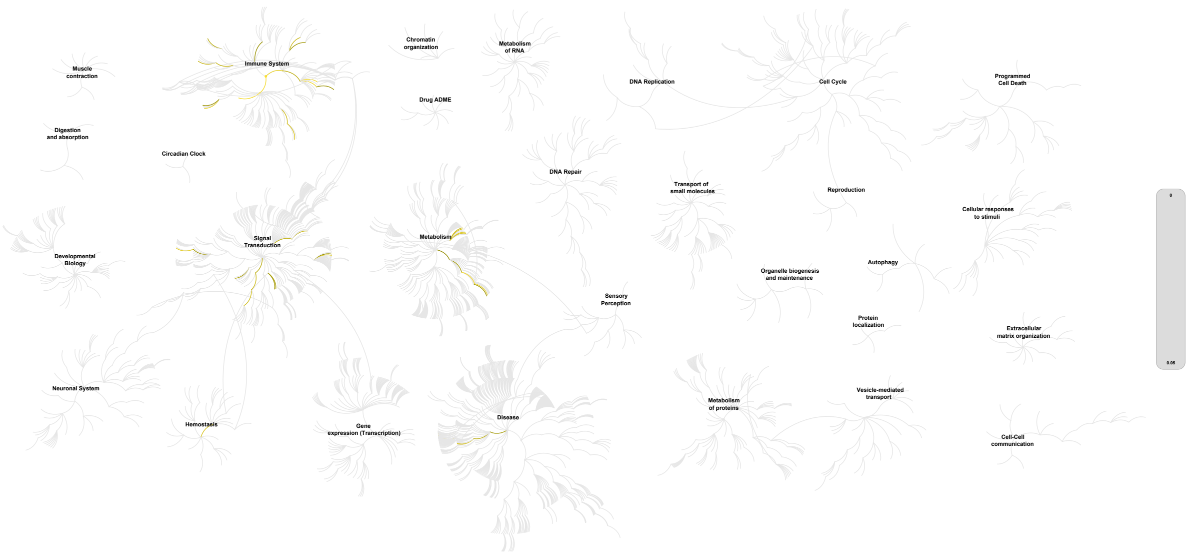


This figure shows a genome-wide overview of the results of your pathway analysis. Reactome pathways are arranged in a hierarchy. The center of each of the circular "bursts" is the root of one top-level pathway, for example "DNA Repair". Each step away from the center represents the next level lower in the pathway hierarchy. The color code denotes over-representation of that pathway in your input dataset. Light grey signifies pathways which are not significantly over-represented.

# 4. Most significant pathways

The following table shows the 25 most relevant pathways sorted by p-value.

| Pathway name | Entities | | | | Reactions | |
|---|---|---|---|---|---|---|
| | found | ratio | p-value | FDR* | found | ratio |
| Hyaluronan uptake and degradation | 2 / 18 | 0.001 | 4.81e-04 | 0.089 | 4 / 10 | 6.99e-04 |
| Hyaluronan metabolism | 2 / 23 | 0.002 | 7.80e-04 | 0.089 | 4 / 13 | 9.09e-04 |
| Immune System | 12 / 2,627 | 0.172 | 9.16e-04 | 0.089 | 80 / 1,664 | 0.116 |
| Neutrophil degranulation | 5 / 478 | 0.031 | 0.001 | 0.1 | 5 / 10 | 6.99e-04 |
| Innate Immune System | 8 / 1,341 | 0.088 | 0.002 | 0.1 | 28 / 725 | 0.051 |
| Interleukin-2 family signaling | 2 / 47 | 0.003 | 0.003 | 0.131 | 7 / 59 | 0.004 |
| Interleukin-3, Interleukin-5 and GM-CSF signaling | 2 / 50 | 0.003 | 0.004 | 0.131 | 25 / 38 | 0.003 |
| Defective HEXB causes GM2G2 | 1 / 4 | 2.63e-04 | 0.007 | 0.131 | 3 / 3 | 2.10e-04 |
| MPS VII - Sly syndrome | 1 / 4 | 2.63e-04 | 0.007 | 0.131 | 3 / 3 | 2.10e-04 |
| The AIM2 inflammasome | 1 / 4 | 2.63e-04 | 0.007 | 0.131 | 2 / 4 | 2.80e-04 |
| Cytokine Signaling in Immune system | 6 / 1,039 | 0.068 | 0.009 | 0.131 | 34 / 745 | 0.052 |
| Adenosine P1 receptors | 1 / 5 | 3.28e-04 | 0.009 | 0.131 | 1 / 2 | 1.40e-04 |
| RAC2 GTPase cycle | 2 / 92 | 0.006 | 0.012 | 0.131 | 2 / 10 | 6.99e-04 |
| PKA-mediated phosphorylation of key metabolic factors | 1 / 7 | 4.60e-04 | 0.012 | 0.131 | 4 / 5 | 3.49e-04 |
| Defective CSF2RB causes SMDP5 | 1 / 8 | 5.25e-04 | 0.014 | 0.131 | 1 / 1 | 6.99e-05 |
| Defective CSF2RA causes SMDP4 | 1 / 8 | 5.25e-04 | 0.014 | 0.131 | 1 / 1 | 6.99e-05 |
| CLEC7A/inflammasome pathway | 1 / 8 | 5.25e-04 | 0.014 | 0.131 | 2 / 4 | 2.80e-04 |
| Platelet activation, signaling and aggregation | 3 / 293 | 0.019 | 0.015 | 0.131 | 28 / 117 | 0.008 |
| ChREBP activates metabolic gene expression | 1 / 9 | 5.91e-04 | 0.016 | 0.131 | 6 / 6 | 4.19e-04 |
| PP2A-mediated dephosphorylation of key metabolic factors | 1 / 9 | 5.91e-04 | 0.016 | 0.131 | 3 / 4 | 2.80e-04 |
| RAF/MAP kinase cascade | 3 / 322 | 0.021 | 0.019 | 0.131 | 10 / 75 | 0.005 |
| CLEC7A (Dectin-1) signaling | 2 / 120 | 0.008 | 0.019 | 0.131 | 4 / 45 | 0.003 |
| AMPK inhibits chREBP transcriptional activation activity | 1 / 11 | 7.22e-04 | 0.019 | 0.131 | 1 / 4 | 2.80e-04 |
| MAPK1/MAPK3 signaling | 3 / 329 | 0.022 | 0.02 | 0.131 | 10 / 82 | 0.006 |
| Antigen processing-Cross presentation | 2 / 128 | 0.008 | 0.022 | 0.131 | 3 / 23 | 0.002 |

* False Discovery Rate

# 5. Pathways details

For every pathway of the most significant pathways, we present its diagram, as well as a short summary, its bibliography and the list of inputs found in it.

## 1. Hyaluronan uptake and degradation (R-HSA-2160916)

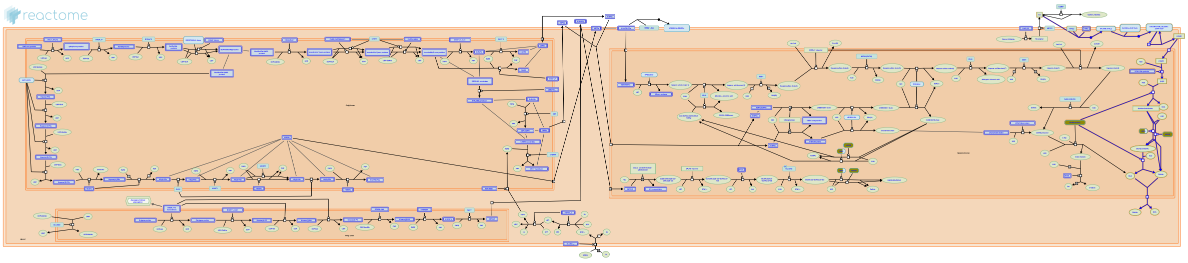

Hyaluronan (HA) turnover can occur locally at the tissue of origin, where it is taken up by cells to be degraded, or released into the lymphatic and vascular systems, where it can be eliminated by the liver and kidneys. Uptake of HA into cells for degradation involves receptor-mediated processes. Once HA enters lysosomes, the acidic conditions favour hyaluronidases to cleave it into small oligosaccharides, the most common size being a tetrasaccharide. Beta-glucuronidases participate in degrading the small oligosaccharides in the lysosome. Ultimately, HA is degraded into its constituent sugars (glucuronic acid and N-acetylglucosamine) which can be used to reform many glycosaminoglycans (GAGs) when released from the lysosome.

A third of the total HA content in humans is turned over daily and it has a short half life of minutes in circulation up to days in many tissues. The reasons why the body eliminates HA so rapidly are unknown but one possible explanation could be HA's role as a reactive oxygen species (ROS) scavenger. Removing these toxic compounds could explain the rapid elimination of HA (Lepperdinger et al. 2004, Menzel & Farr 1998, Erickson & Stern 2012, Stern 2003).

### References


Stern R & Erickson M (2012). Chain gangs: new aspects of hyaluronan metabolism. Biochem Res Int , 2012, 893947.

Menzel EJ & Farr C (1998). Hyaluronidase and its substrate hyaluronan: biochemistry, biological activities and therapeutic uses. Cancer Lett, 131, 3-11.

Stern R (2003). Devising a pathway for hyaluronan catabolism: are we there yet?. Glycobiology, 13, 105R-115R.

Hales CA & Garg HG (2004). *Chemistry and Biology of Hyaluronan* , 71-81.


### Edit history

| Date | Action | Author |
|---|---|---|
| 2012-03-05 | Edited | Jassal B |
| 2012-03-05 | Authored | Jassal B |
| 2012-03-05 | Created | Jassal B |
| 2012-03-28 | Reviewed | D'Eustachio P |

| Date | Action | Author |
|---|---|---|
| 2023-05-21 | Modified | Wright A |

## 2 submitted entities found in this pathway, mapping to 2 Reactome entities

| Input | UniProt Id | Input | UniProt Id |
|---|---|---|---|
| Gusb | P08236 | Hexb | P07686 |

## 2. Hyaluronan metabolism (R-HSA-2142845)

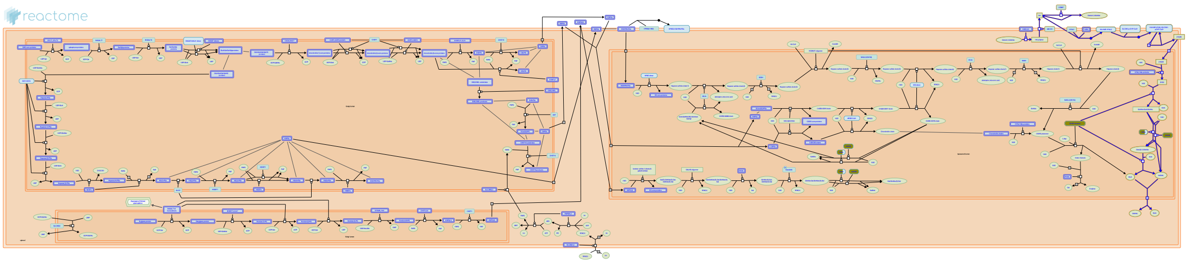

Hyaluronan (hyaluronic acid, hyaluronate or HA) is an anionic glycosaminoglycan (GAG) distributed widely throughout connective, epithelial, and neural tissues and most abundant in the extracellular matrix and skin. HA is unique among the GAGs in that it is not sulfated and is not found covalently attached to proteins as a proteoglycan. HA polymers are very large (they can reach molecular weights of 10 million Da) and can displace a large volume of water making them excellent lubricators and shock absorbers. Another unique feature of HA is that it is synthesized at the plasma membrane unlike other GAGs which are formed in the Golgi. HA is a polymer of the disaccharide unit D-glucuronic acid and D-N-acetylglucosamine, linked via alternating beta-1,4 and beta-1,3 glycosidic bonds (Toole 2000, 2004, Volpi et al. 2009).

### References


Toole BP (2000). Hyaluronan is not just a goo!. J Clin Invest, 106, 335-6.

Schiller J, Volpi N, Stern R & Soltés L (2009). Role, metabolism, chemical modifications and applications of hyaluronan. Curr Med Chem, 16, 1718-45.

Toole BP (2004). Hyaluronan: from extracellular glue to pericellular cue. Nat Rev Cancer, 4, 528-39.


### Edit history

| Date | Action | Author |
|---|---|---|
| 2012-02-24 | Edited | Jassal B |
| 2012-02-24 | Authored | Jassal B |
| 2012-02-24 | Created | Jassal B |
| 2012-03-28 | Reviewed | D'Eustachio P |
| 2023-05-21 | Modified | Wright A |

### 2 submitted entities found in this pathway, mapping to 2 Reactome entities

| Input | UniProt Id | Input | UniProt Id |
|---|---|---|---|
| Gusb | P08236 | Hexb | P07686 |

## 3. Immune System (R-HSA-168256)

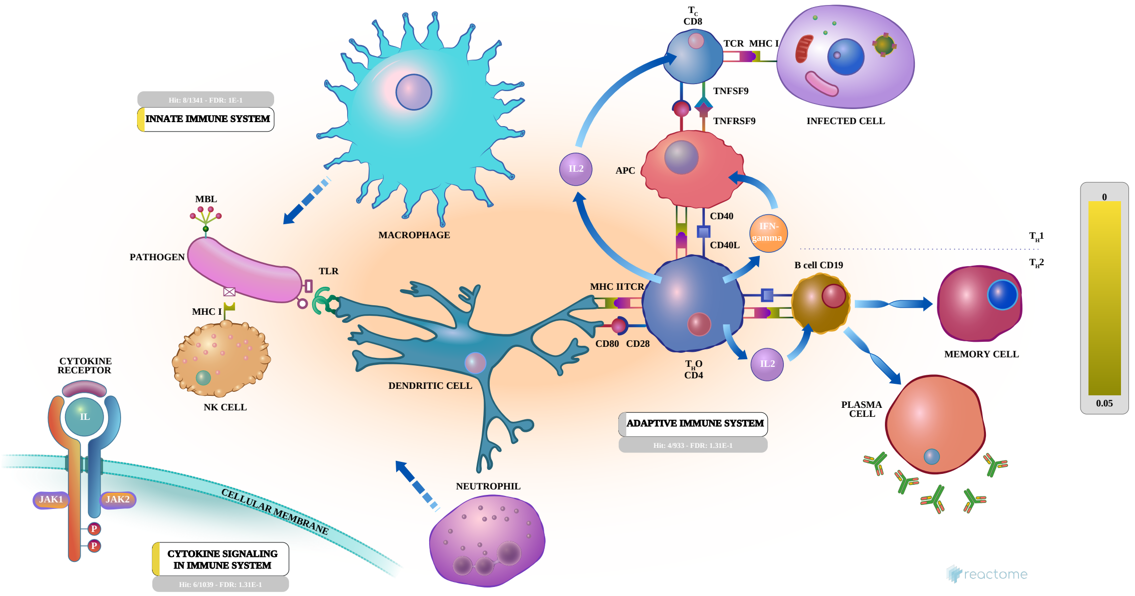


Humans are exposed to millions of potential pathogens daily, through contact, ingestion, and inhalation. Our ability to avoid infection depends on the adaptive immune system and during the first critical hours and days of exposure to a new pathogen, our innate immune system.

### References

### Edit history

| Date | Action | Author |
|---|---|---|
| 2005-11-12 | Created | Gillespie ME |
| 2006-03-30 | Authored | Luo F, Ouwehand WH, Gillespie ME, de Bono B |
| 2006-04-19 | Reviewed | Zwaginga JJ, D'Eustachio P, Gay NJ, Gale M Jr |
| 2023-05-21 | Modified | Wright A |

### 11 submitted entities found in this pathway, mapping to 12 Reactome entities

| Input | UniProt Id | Input | UniProt Id | Input | UniProt Id |
|---|---|---|---|---|---|
| Csf2rb | P32927 | Gusb | P08236 | Havcr2 | Q8TDQ0 |
| Hexb | P07686 | Hk3 | P52790 | Irf5 | Q13568 |
| Lair1 | Q6GTX8 | Ncf4 | Q15080 | Psmb9 | P28065 |
| Pycard | Q9ULZ3 | Vav1 | P15498 | | |

| Input | Ensembl Id |
|---|---|
| Irf5 | ENSG00000128604 |

## 4. Neutrophil degranulation (R-HSA-6798695)

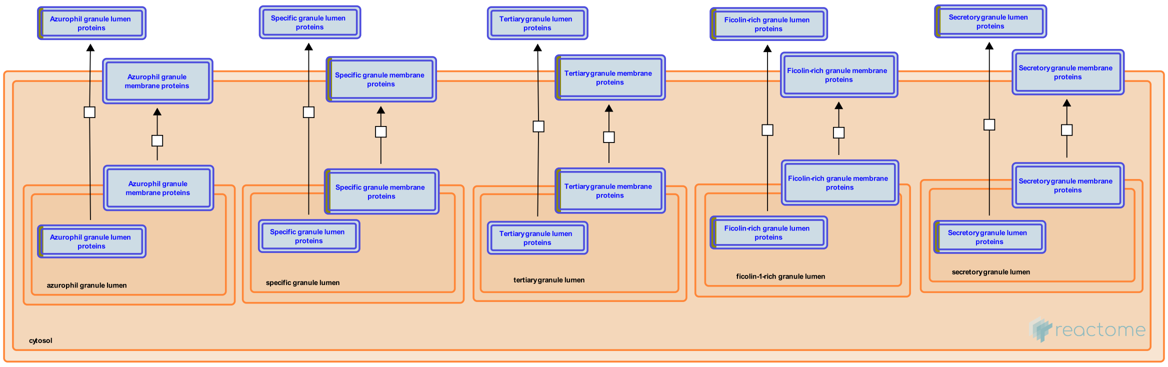


Neutrophils are the most abundant leukocytes (white blood cells), indispensable in defending the body against invading microorganisms. In response to infection, neutrophils leave the circulation and migrate towards the inflammatory focus. They contain several subsets of granules that are mobilized to fuse with the cell membrane or phagosomal membrane, resulting in the exocytosis or exposure of membrane proteins. Traditionally, neutrophil granule constituents are described as antimicrobial or proteolytic, but granules also introduce membrane proteins to the cell surface, changing how the neutrophil responds to its environment (Borregaard et al. 2007). Primed neutrophils actively secrete cytokines and other inflammatory mediators and can present antigens via MHC II, stimulating T-cells (Wright et al. 2010).

Granules form during neutrophil differentiation. Granule subtypes can be distinguished by their content but overlap in structure and composition. The differences are believed to be a consequence of changing protein expression and differential timing of granule formation during the terminal processes of neutrophil differentiation, rather than sorting (Le Cabec et al. 1996).

The classical granule subsets are Azurophil or primary granules (AG), secondary granules (SG) and gelatinase granules (GG). Neutrophils also contain exocytosable storage cell organelles, storage vesicles (SV), formed by endocytosis they contain many cell-surface markers and extracellular, plasma proteins (Borregaard et al. 1992). Ficolin-1-rich granules (FG) are like GGs highly exocytosable but gelatinase-poor (Rorvig et al. 2009).

### References

Heegaard NH, Rørvig S, Borregaard N & Østergaard O (2013). Proteome profiling of human neutrophil granule subsets, secretory vesicles, and cell membrane: correlation with transcriptome profiling of neutrophil precursors. J. Leukoc. Biol., 94, 711-21.

Sørensen OE, Borregaard N & Theilgaard-Mönch K (2007). Neutrophil granules: a library of innate immunity proteins. Trends Immunol., 28, 340-5.

Nielsen MH, Johnsen AH, Bjerrum OW, Borregaard N, Kjeldsen L, Rygaard K, ... Bastholm L (1992). Stimulus-dependent secretion of plasma proteins from human neutrophils. J. Clin. Invest., 90, 86-96.

Bucknall RC, Wright HL, Edwards SW & Moots RJ (2010). Neutrophil function in inflammation and inflammatory diseases. Rheumatology (Oxford), 49, 1618-31.

Le Cabec V, Borregaard N, Calafat J & Cowland JB (1996). Targeting of proteins to granule subsets is determined by timing and not by sorting: The specific granule protein NGAL is localized to azurophil granules when expressed in HL-60 cells. Proc. Natl. Acad. Sci. U.S.A., 93, 6454-7.

## Edit history

| Date | Action | Author |
|---|---|---|
| 2015-09-21 | Authored | Jupe S |
| 2015-09-21 | Created | Jupe S |
| 2016-06-13 | Edited | Jupe S |
| 2016-06-13 | Reviewed | Heegaard N |
| 2023-05-21 | Modified | Wright A |

## 5 submitted entities found in this pathway, mapping to 5 Reactome entities

| Input | UniProt Id | Input | UniProt Id | Input | UniProt Id |
|---|---|---|---|---|---|
| Gusb | P08236 | Hexb | P07686 | Hk3 | P52790 |
| Lair1 | Q6GTX8 | Pycard | Q9ULZ3 | | |

## 5. Innate Immune System (R-HSA-168249)

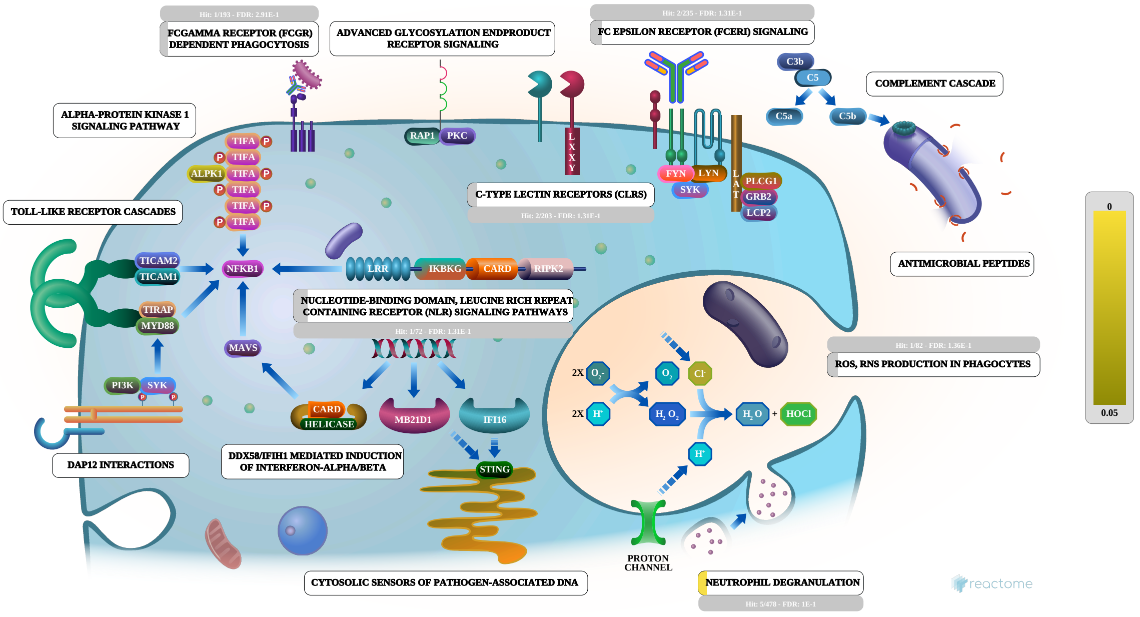


Innate immunity encompases the nonspecific part of immunity tha are part of an individual's natural biologic makeup

## References

## Edit history

| Date | Action | Author |
|---|---|---|
| 2005-11-12 | Created | Gillespie ME |
| 2023-05-21 | Modified | Wright A |

## 8 submitted entities found in this pathway, mapping to 8 Reactome entities

| Input | UniProt Id | Input | UniProt Id | Input | UniProt Id |
|---|---|---|---|---|---|
| Gusb | P08236 | Hexb | P07686 | Hk3 | P52790 |
| Lair1 | Q6GTX8 | Ncf4 | Q15080 | Psmb9 | P28065 |
| Pycard | Q9ULZ3 | Vav1 | P15498 | | |

## 6. Interleukin-2 family signaling (R-HSA-451927)

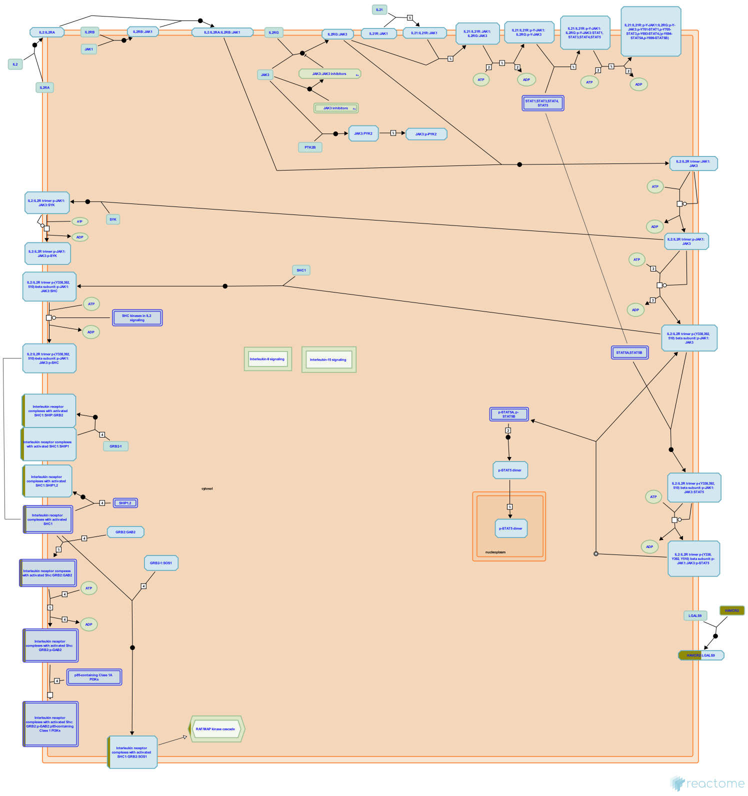

**Cellular compartments:** plasma membrane.

The interleukin-2 family (also called the common gamma chain cytokine family) consists of interleukin (IL)2, IL9, IL15 and IL21. Although sometimes considered to be within this family, the IL4 and IL7 receptors can form complexes with other receptor chains and are represented separately in Reactome. Receptors of this family associate with JAK1 and JAK3, primarily activating STAT5, although certain family members can also activate STAT1, STAT3 or STAT6.

### References


Sim GC & Radvanyi L (2014). The IL-2 cytokine family in cancer immunotherapy. Cytokine Growth Factor Rev., 25, 377-90.

Wang X, Laporte SL, Lupardus P & Garcia KC (2009). Structural biology of shared cytokine receptors. Annu Rev Immunol, 27, 29-60.

Spolski R, Leonard WJ & Rochman Y (2009). New insights into the regulation of T cells by gamma(c) family cytokines. Nat Rev Immunol, 9, 480-90.


### Edit history

| Date | Action | Author |
|---|---|---|
| 2010-01-14 | Created | Jupe S |
| 2010-05-17 | Authored | Ray KP |
| 2010-08-06 | Edited | Jupe S |
| 2011-02-11 | Reviewed | Villarino A |
| 2011-03-17 | Reviewed | Dooms H |

| Date | Action | Author |
| --- | --- | --- |
| 2023-05-30 | Modified | Wright A |

## 2 submitted entities found in this pathway, mapping to 2 Reactome entities

| Input | UniProt Id | Input | UniProt Id |
| --- | --- | --- | --- |
| Csf2rb | P32927 | Havcr2 | Q8TDQ0 |

## 7. Interleukin-3, Interleukin-5 and GM-CSF signaling (R-HSA-512988)

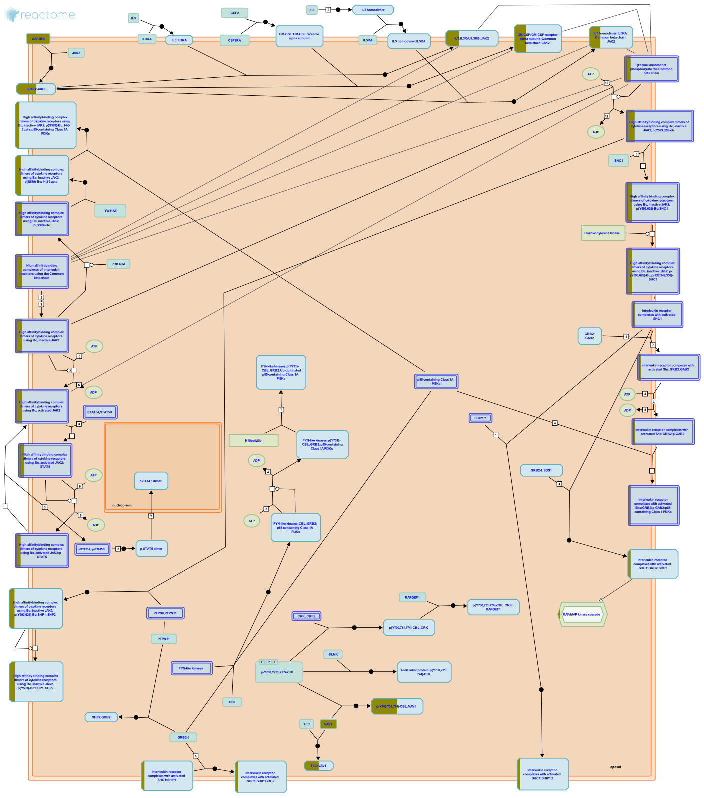

**Cellular compartments:** plasma membrane.

The Interleukin-3 (IL-3), IL-5 and Granulocyte-macrophage colony stimulating factor (GM-CSF) receptors form a family of heterodimeric receptors that have specific alpha chains but share a common beta subunit, often referred to as the common beta (Bc). Both subunits contain extracellular conserved motifs typical of the cytokine receptor superfamily. The cytoplasmic domains have limited similarity with other cytokine receptors and lack detectable catalytic domains such as tyrosine kinase domains.

IL-3 is a 20-26 kDa product of CD4+ T cells that acts on the most immature marrow progenitors. IL-3 is capable of inducing the growth and differentiation of multi-potential hematopoietic stem cells, neutrophils, eosinophils, megakaryocytes, macrophages, lymphoid and erythroid cells. IL-3 has been used to support the proliferation of murine cell lines with properties of multi-potential progenitors, immature myeloid as well as T and pre-B lymphoid cells (Miyajima et al. 1992). IL-5 is a hematopoietic growth factor responsible for the maturation and differentiation of eosinophils. It was originally defined as a T-cell-derived cytokine that triggers activated B cells for terminal differentiation into antibody-secreting plasma cells. It also promotes the generation of cytotoxic T-cells from thymocytes. IL-5 induces the expression of IL-2 receptors (Kouro & Takatsu 2009). GM-CSF is produced by cells (T-lymphocytes, tissue macrophages, endothelial cells, mast cells) found at sites of inflammatory responses. It stimulates the growth and development of progenitors of granulocytes and macrophages, and the production and maturation of dendritic cells. It stimulates myeloblast and monoblast differentiation, synergises with Epo in the proliferation of erythroid and megakaryocytic progenitor cells, acts as an autocrine mediator of growth for some types of acute myeloid leukemia, is a strong chemoattractant for neutrophils and eosinophils. It enhances the activity of neutrophils and macrophages. Under steady-state conditions GM-CSF is not essential for the production of myeloid cells, but it is required for the proper development of alveolar macrophages, otherwise, pulmonary alvelolar proteinosis (PAP) develops. A growing body of evidence suggests that GM-CSF plays a key role in emergency hematopoiesis (predominantly myelopoiesis) in response to infection, including the production of granulocytes and macrophages in the bone marrow and their maintenance, survival, and functional activation at sites of injury or insult (Hercus et al. 2009).

All three receptors have alpha chains that bind their specific ligands with low affinity (de Groot et al. 1998). Bc then associates with the alpha chain forming a high affinity receptor (Geijsen et al. 2001), though the in vivo receptor is likely be a higher order multimer as recently demonstrated for the GM-CSF receptor (Hansen et al. 2008).

The receptor chains lack intrinsic kinase activity, instead they interact with and activate signaling kinases, notably Janus Kinase 2 (JAK2). These phosphorylate the common beta subunit, allowing recruitment of signaling molecules such as Shc, the phosphatidylinositol 3-kinases (PI3Ks), and the Signal Transducers and Activators of Transcription (STATs). The cytoplasmic domain of Bc has two distinct functional domains: the membrane proximal region mediates the induction of proliferation-associated genes such as c-myc, pim-1 and oncostatin M. This region binds multiple signal-transducing proteins including JAK2 (Quelle et al. 1994), STATs, c-Src and PI3 kinase (Rao and Mufson, 1995). The membrane distal domain is required for cytokine-induced growth inhibition and is necessary for the viability of hematopoietic cells (Inhorn et al. 1995). This region interacts with signal-transducing proteins such as Shc (Inhorn et al. 1995) and SHP and mediates the transcriptional activation of c-fos, c-jun, c-Raf and p70S6K (Reddy et al. 2000).

Figure reproduced by permission from Macmillan Publishers Ltd: Leukemia, WL Blalock et al. 13:1109-1166, copyright 1999. Note that residue numbering in this diagram refers to the mature Common beta chain with signal peptide removed.

## References

Bagley CJ, Berndt MC, Stomski FC, Lopez AF, Woodcock JM, Thomas D & Guthridge MA (1998). Mechanism of activation of the GM-CSF, IL-3, and IL-5 family of receptors. Stem Cells, 16, 301-13.

McCubrey JA, Hoyle PE, Steelman LS, Franklin RA, Weinstein-Oppenheimer C, Wang XY, ... Oberhaus SM (1999). Signal transduction, cell cycle regulatory, and anti-apoptotic pathways regulated by IL-3 in hematopoietic cells: possible sites for intervention with anti-neoplastic drugs. Leukemia, 13, 1109-66.

## Edit history

| Date | Action | Author |
|---|---|---|
| 2010-02-16 | Created | Jupe S |
| 2010-05-17 | Authored | Ray KP |
| 2010-08-06 | Edited | Jupe S |
| 2010-09-06 | Reviewed | Hercus TR, Lopez AF |
| 2023-05-30 | Modified | Wright A |

## 2 submitted entities found in this pathway, mapping to 2 Reactome entities

| Input | UniProt Id | Input | UniProt Id |
|---|---|---|---|
| Csf2rb | P32927 | Vav1 | P15498 |

## 8. Defective HEXB causes GM2G2 (R-HSA-3656248)

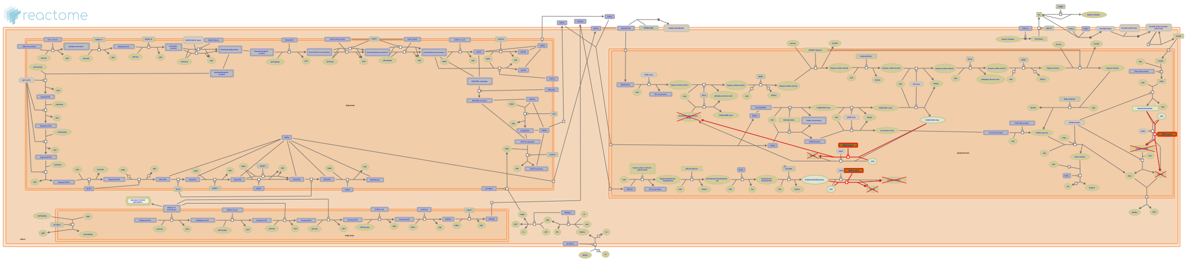

**Diseases:** gangliosidosis GM2.

Beta-hexosaminidase (HEX) cleaves the terminal N-acetyl galactosamine (GalNAc) from glycosaminoglycans (GAGs) and any other molecules containing a terminal GalNAc. There are two forms of HEX; HEXA and B. The A form is a trimer of the subunits alpha, beta A and beta B. The B form is a tetramer of 2 beta A and 2 beta B subunits (O'Dowd et al. 1988). Defects in the two subunits cause lysosomal storage diseases marked by the accumulation of GM2 gangliosides in neuronal cells.

Defects in the beta subunits are the cause of GM2-gangliosidosis type 2 (GM2G2; MIM:268800), also known as Sandhoff disease (Sandhoff et al. 1968, Banerjee et al. 1991). Sandhoff disease is an autosomal recessive lysosomal storage disease clinically indistinguishable from GM2-gangliosidosis type 1, presenting early blindness with cherry-red spots on the macula, progressive motor and mental deterioration and macrocephaly. Death usually occurs by the age of 3 years.

### References


Boyers MJ, Siciliano L, Banerjee P, Oliveri D, Horwitz AL, Li SC, ... Dawson G (1991). Molecular basis of an adult form of beta-hexosaminidase B deficiency with motor neuron disease. Biochem Biophys Res Commun, 181, 108-15.

Jatzkewitz H, Andreae U & Sandhoff K (1968). Deficient hexozaminidase activity in an exceptional case of Tay-Sachs disease with additional storage of kidney globoside in visceral organs. Life Sci. , 7, 283-8.


### Edit history

| Date | Action | Author |
|---|---|---|
| 2013-05-31 | Edited | Jassal B |
| 2013-05-31 | Authored | Jassal B |
| 2013-05-31 | Created | Jassal B |
| 2014-07-09 | Reviewed | Spillmann D |
| 2023-03-08 | Modified | Matthews L |

### 1 submitted entities found in this pathway, mapping to 1 Reactome entities

| Input | UniProt Id |
|---|---|
| Hexb | P07686 |

## 9. MPS VII - Sly syndrome (R-HSA-2206292)

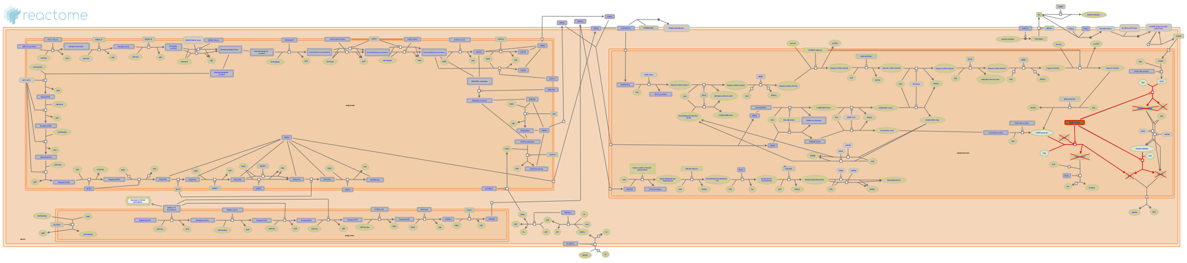

**Diseases:** mucopolysaccharidosis VII.

Mucopolysaccharidosis type VII (MPS VII, Sly syndrome, beta-glucuronidase deficiency; MIM:253220) is an autosomal recessive lysosomal storage disease characterized by a deficiency of the enzyme beta-glucuronidase (GUSB; MIM:611499) which would normally cleave glucuronide residues from dematan sulphate, keratan sulphate and chondroitin sulphate, resulting in build up of these GAGs in cells and tissues (Sly et al. 1973). The gene encoding GUSB is 21 kb long, contains 12 exons and gives rise to two different types of cDNAs, through an alternate splicing mechanism (Miller et al. 1990). It maps to 7q11.21-q11.22 (Speleman et al. 1996). The phenotype is highly variable, ranging from severe causing death, non-immune hydrops fetalis (Vervoort et al. 1996) to mild forms with survival into adulthood (Storch et al. 2003). Most patients with the intermediate phenotype show hepatomegaly, skeletal anomalies, coarse facies, and variable degrees of mental impairment (Shipley et al. 1993, Tomatsu et al. 2009).

### References


Bachinsky DR, Grubb JH, Sly WS, Klinkenberg M, Wu BM & Shipley JM (1993). Mutational analysis of a patient with mucopolysaccharidosis type VII, and identification of pseudogenes. Am. J. Hum. Genet., 52, 517-26.

Dung VC, Sly WS, Montaño AM, Tomatsu S & Grubb JH (2009). Mutations and polymorphisms in GUSB gene in mucopolysaccharidosis VII (Sly Syndrome). Hum Mutat, 30, 511-9.

Storch S, Wittenstein B, Braulke T, Sly WS, Ullrich K & Islam R (2003). Mutational analysis in longest known survivor of mucopolysaccharidosis type VII. Hum. Genet., 112, 190-4.

Zabot MT, Young EP, Liebaers I, Lissens W, Chabas A, Islam MR, ... Fensom A (1996). Molecular analysis of patients with beta-glucuronidase deficiency presenting as hydrops fetalis or as early mucopolysaccharidosis VII. Am. J. Hum. Genet., 58, 457-71.

Bachinsky DR, Miller RD, Hoffmann JW, Powell PP, Kyle JW, Sly WS & Shipley JM (1990). Cloning and characterization of the human beta-glucuronidase gene. Genomics, 7, 280-3.


### Edit history

| Date | Action | Author |
|---|---|---|
| 2012-04-26 | Edited | Jassal B |
| 2012-04-26 | Authored | Jassal B |
| 2012-04-26 | Created | Jassal B |
| 2012-08-27 | Reviewed | Alves S, Coutinho MF |
| 2012-08-28 | Reviewed | Ashworth J |
| 2023-03-08 | Modified | Matthews L |

## 1 submitted entities found in this pathway, mapping to 1 Reactome entities

| Input | UniProt Id |
| --- | --- |
| Gusb | P08236 |

## 10. The AIM2 inflammasome (R-HSA-844615)

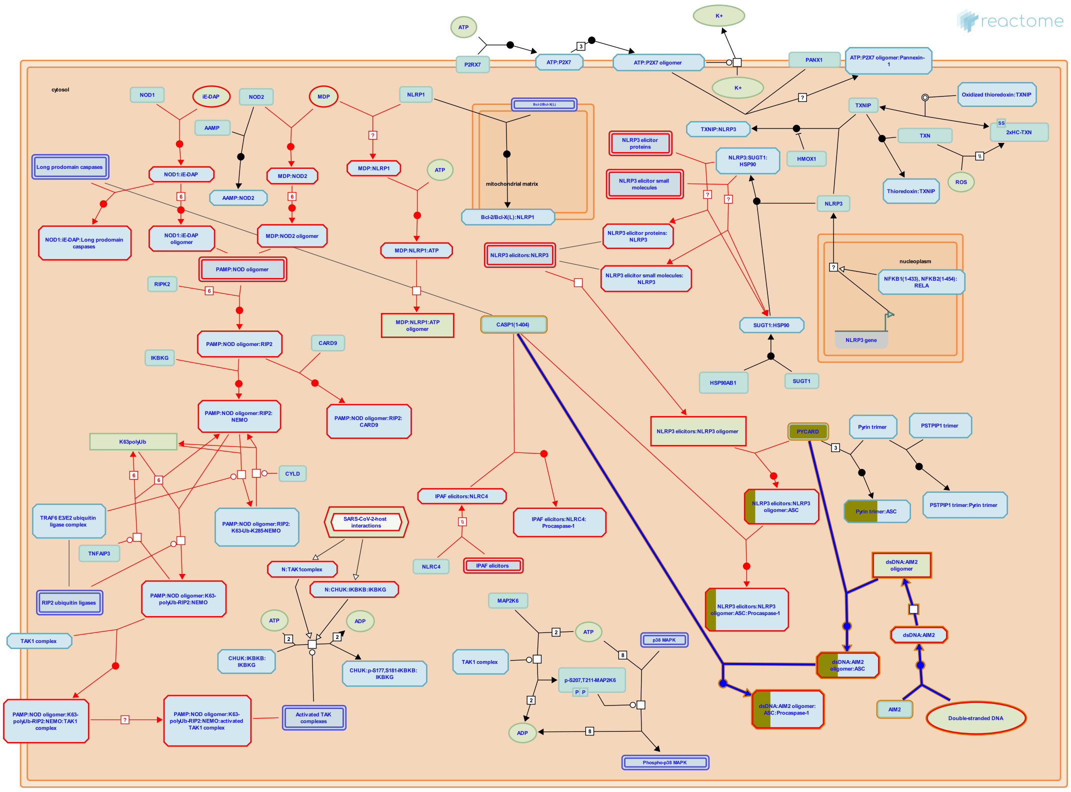


**Cellular compartments:** cytosol.

AIM2 is a member of the PYHIN or HIN200 family. It has a C-terminal HIN domain which can bind double-stranded DNA (dsDNA) and a PYD domain that can bind ASC via a PYD-PYD interaction. In cells expressing procaspase-1, The interaction of AIM2 with ASC leads to recruitment of procaspase-1  forming the ASC pyroptosome which induces pyroptotic cell death by generating active caspase-1. Data from AIM2 deficient mice indicates that the AIM2 inflammasome is a nonredundant sensor for dsDNA that regulates the caspase-1-dependent maturation of IL-1beta and IL-18 (Rathinam et al. 2010, Hornung & Latz, 2009).

### References


Schroder K & Tschopp J (2010). The inflammasomes. Cell, 140, 821-32.


### Edit history

| Date | Action | Author |
|---|---|---|
| 2010-04-22 | Authored | Jupe S |
| 2010-05-28 | Created | Jupe S |
| 2011-04-28 | Edited | Jupe S |
| 2011-04-28 | Reviewed | Kufer TA |
| 2011-06-06 | Reviewed | Rittinger K, Wong E |
| 2023-05-21 | Modified | Wright A |

### 1 submitted entities found in this pathway, mapping to 1 Reactome entities

| Input | UniProt Id |
| --- | --- |
| Pycard | Q9ULZ3 |

## 11. Cytokine Signaling in Immune system (R-HSA-1280215)

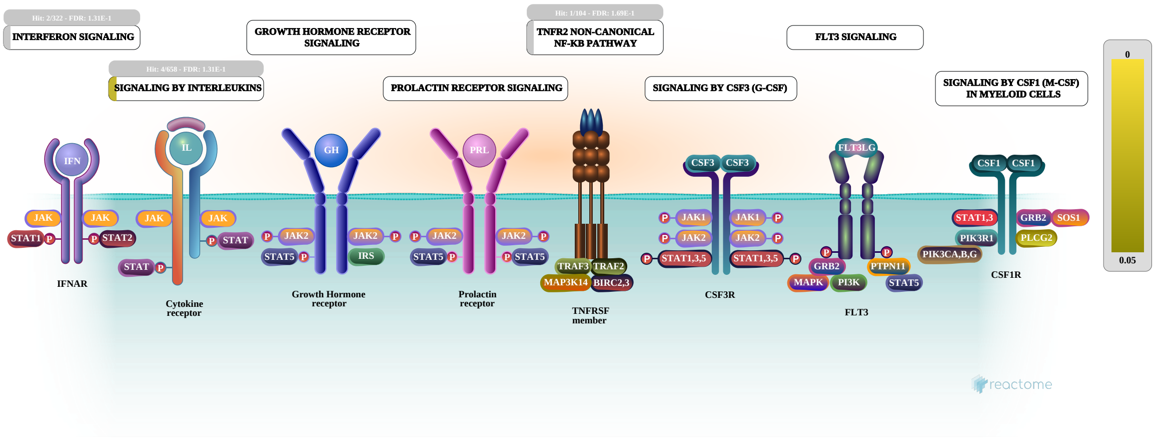


Cytokines are small proteins that regulate and mediate immunity, inflammation, and hematopoiesis. They are secreted in response to immune stimuli, and usually act briefly, locally, at very low concentrations. Cytokines bind to specific membrane receptors, which then signal the cell via second messengers, to regulate cellular activity.

### References


Feldmann M & Oppenheim J (2002). *Cytokines and the immune system, Cytokine Reference* .

IMMPORT:Bioinformatics for the future of immunology. Retrieved from https://www.immport.org/immportWeb/queryref/geneListSummary.do

Santamaria P (2003). Cytokines and chemokines in autoimmune disease: an overview. Adv Exp Med Biol, 520, 1-7.

COPE. Retrieved from http://www.copewithcytokines.org/cope.cgi


### Edit history

| Date | Action | Author |
|---|---|---|
| 2011-05-12 | Created | Garapati P V |
| 2011-05-22 | Edited | Ray KP, Jupe S, Garapati P V |
| 2011-05-22 | Authored | Ray KP, Jupe S, Garapati P V |
| 2011-05-29 | Reviewed | Abdul-Sater AA, Schindler C, Pinteaux E |
| 2023-05-21 | Modified | Wright A |

### 5 submitted entities found in this pathway, mapping to 6 Reactome entities

| Input | UniProt Id | Input | UniProt Id | Input | UniProt Id |
|---|---|---|---|---|---|
| Csf2rb | P32927 | Havcr2 | Q8TDQ0 | Irf5 | Q13568 |
| Psmb9 | P28065 | Vav1 | P15498 | | |

| Input | Ensembl Id |
| --- | --- |
| Irf5 | ENSG00000128604 |

## 12. Adenosine P1 receptors (R-HSA-417973)

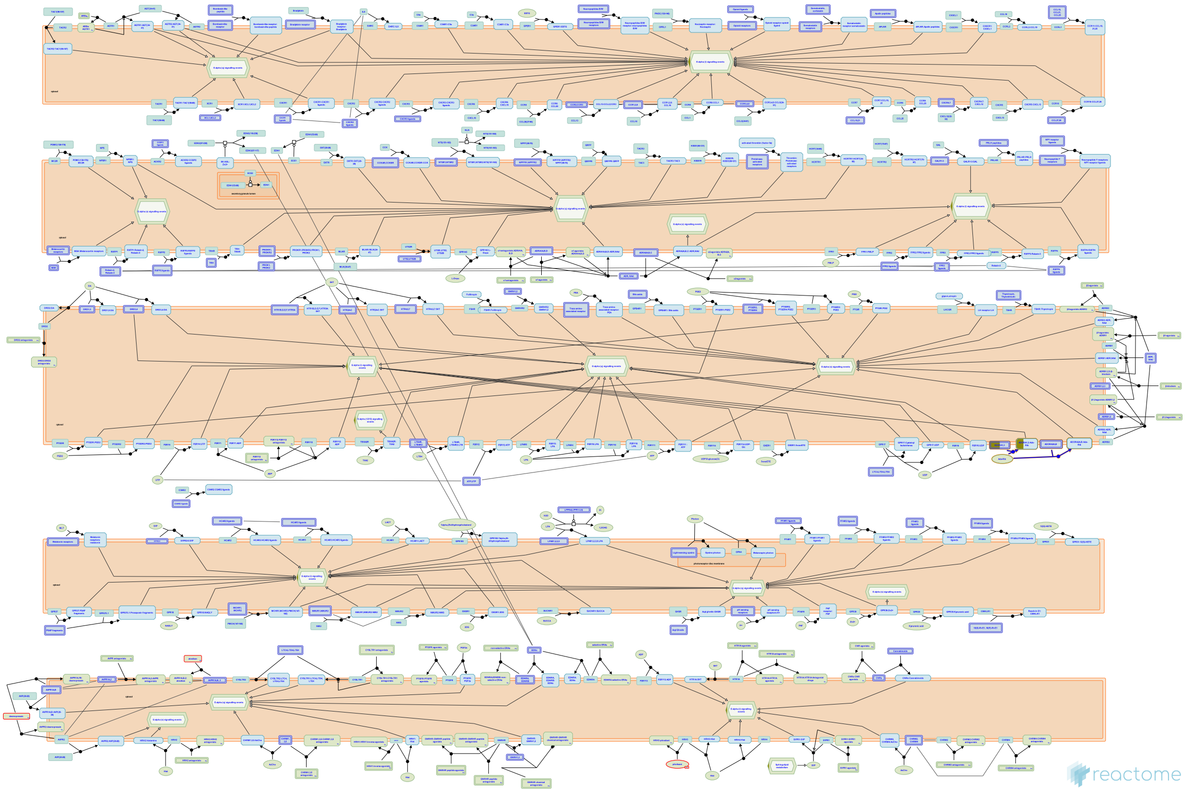

The adenosine receptors (P1 receptors) are a class of purinergic receptors, G-protein coupled receptors with adenosine as their endogenous ligand.In humans, there are four adenosine receptors. Each is encoded by a separate gene and the four receptors have distinct, though overlapping, functions. For instance, both A1 and A2A receptors play roles in the heart, regulating myocardial oxygen consumption and coronary blood flow. They also have important roles in the brain, regulating the release of other neurotransmitters such as dopamine and glutamate. The A2B and A3 receptors are located peripherally and are involved in processes such as inflammation and immune responses. Fredholm BB et al, 2001).

### References


Linden J, Fredholm BB, Jacobson KA, Klotz KN & IJzerman AP (2001). International Union of Pharmacology. XXV. Nomenclature and classification of adenosine receptors. Pharmacol Rev, 53, 527-52.


### Edit history

| Date | Action | Author |
|---|---|---|
| 2009-04-14 | Edited | Jassal B |
| 2009-04-14 | Authored | Jassal B |
| 2009-04-14 | Created | Jassal B |
| 2009-05-29 | Reviewed | D'Eustachio P |
| 2023-05-21 | Modified | Wright A |

### 1 submitted entities found in this pathway, mapping to 1 Reactome entities

| Input | UniProt Id |
| --- | --- |
| Adora3 | P0DMS8 |

## 13. RAC2 GTPase cycle (R-HSA-9013404)

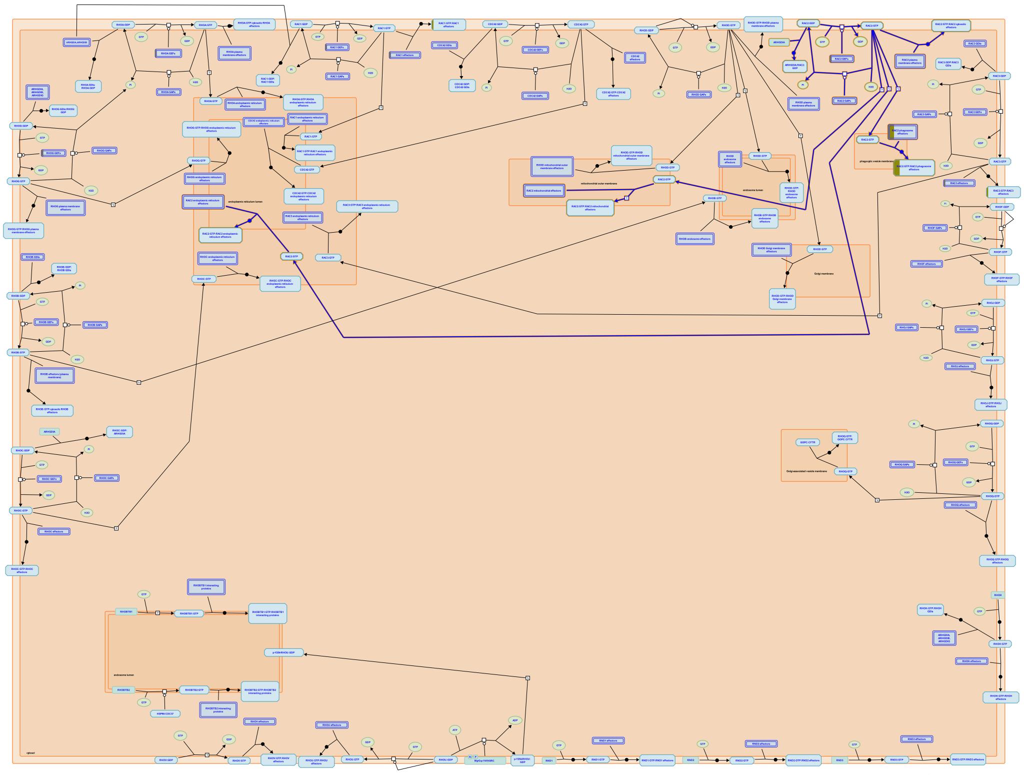

This pathway catalogues RAC2 guanine nucleotide exchange factors (GEFs), GTPase activator proteins (GAPs), GDP dissociation inhibitors (GDIs) and RAC2 effectors. RAC2 is exclusively expressed in hematopoietic cells (Troeger and Williams 2013). RAC2 is a component of the phagocytic oxidase complex in neutrophils (Troeger and Williams 2013). RAC2 is required for adhesion and mobilization of hematopoietic stem cells and progenitor cells (Troeger and Williams 2013). RAC2 is also needed for adhesion, migration and degranulation of mast cells (Troeger and Williams 2013). Mutations in RAC2 have been found in a small number of patients with primary immunodeficiencies (Gu and Williams 2002; Troeger and Williams 2013; Lougaris et al. 2020).

### References


Troeger A & Williams DA (2013). Hematopoietic-specific Rho GTPases Rac2 and RhoH and human blood disorders. Exp. Cell Res., 319, 2375-83.

Benvenuto A, Baronio M, Plebani A, Lougaris V & Gazzurelli L (2020). RAC2 and primary human immune deficiencies. J. Leukoc. Biol..

Gu Y & Williams DA (2002). RAC2 GTPase deficiency and myeloid cell dysfunction in human and mouse. J. Pediatr. Hematol. Oncol., 24, 791-4.


### Edit history

| Date | Action | Author |
|---|---|---|
| 2017-07-25 | Created | Orlic-Milacic M |
| 2020-07-14 | Authored | Rothfels K, Orlic-Milacic M |
| 2021-02-05 | Reviewed | Fort P |
| 2021-02-25 | Edited | Orlic-Milacic M |

| Date | Action | Author |
|---|---|---|
| 2023-05-21 | Modified | Wright A |

## 2 submitted entities found in this pathway, mapping to 2 Reactome entities

| Input | UniProt Id | Input | UniProt Id |
|---|---|---|---|
| Ncf4 | Q15080 | Vav1 | P15498 |

## 14. PKA-mediated phosphorylation of key metabolic factors (R-HSA-163358)

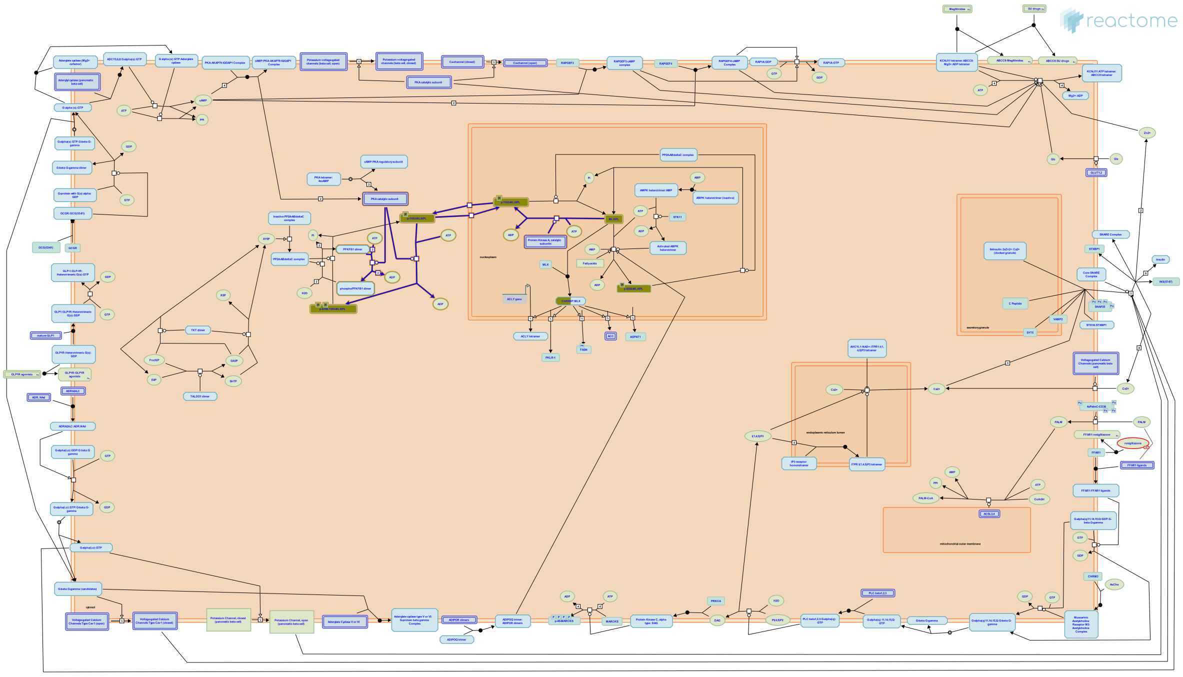

**Cellular compartments:** nucleoplasm, cytosol.

Upon dissociation of protein kinase A (PKA) tetramers in the presence of cAMP, the released PKA catalytic monomers phosphorylate specific serine and threonine residues of several metabolic enzymes. These target enzymes include glycogen phosphorylase kinase, glycogen synthase and PF2K-Pase. PKA also phosphorylates ChREBP (Carbohydrate Response Element Binding Protein), preventing its movement into the nucleus and thus its function as a positive transcription factor for genes involved in glycolytic and lipogenic reactions.

### References


Veech RL (2003). A humble hexose monophosphate pathway metabolite regulates short- and long-term control of lipogenesis. Proc Natl Acad Sci U S A, 100, 5578-80.


### Edit history

| Date | Action | Author |
|---|---|---|
| 2005-04-28 | Created | Gopinathrao G |
| 2005-05-13 | Authored | Gopinathrao G |
| 2023-05-21 | Modified | Wright A |

### 1 submitted entities found in this pathway, mapping to 1 Reactome entities

| Input | UniProt Id |
|---|---|
| Mlxipl | Q9NP71 |

## 15. Defective CSF2RB causes SMDP5 (R-HSA-5688849)

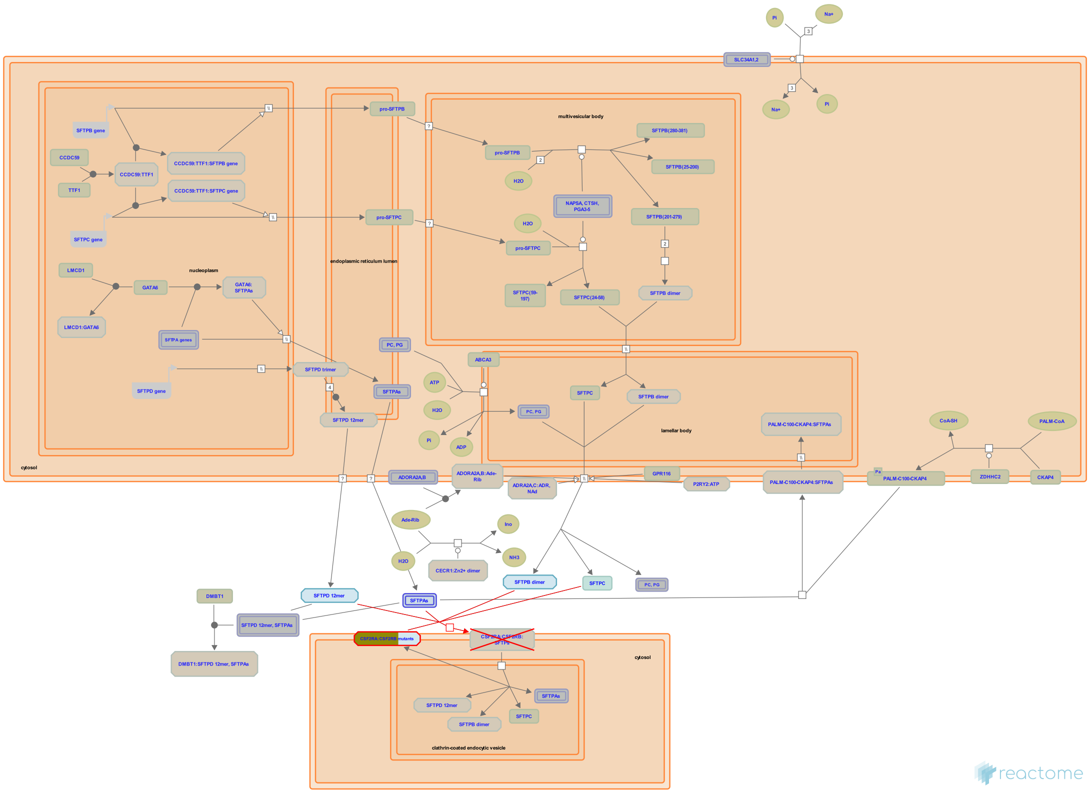

**Diseases:** pulmonary alveolar proteinosis.

Surfactant catabolism by alveolar macrophages plays a small but critical part in surfactant recycling and metabolism. Upon ligand binding, granulocyte-macrophage colony-stimulating factor receptor (GM-CSFR), a heterodimer of alpha (CSF2RA) and beta (CSF2RB) subunits, initiates a signalling process that not only induces proliferation, differentiation and functional activation of hematopoietic cells but can also determine surfactant uptake into alveolar macrophages and its degradation via clathrin-coated vesicles. Defects in human CSF2RB can cause pulmonary surfactant metabolism dysfunction 5 (SMDP5; MIM:614370, aka pulmonary alveolar proteinosis 5, PAP5), a rare lung disorder due to impaired surfactant homeostasis characterised by alveoli filling with floccular material causing respiratory failure (Greenhill & Kotton 2009, Whitsett et al. 2015).

### References


Whitsett JA, Weaver TE & Wert SE (2015). Diseases of pulmonary surfactant homeostasis. Annu Rev Pathol, 10, 371-93.

Kotton DN & Greenhill SR (2009). Pulmonary alveolar proteinosis: a bench-to-bedside story of granulocyte-macrophage colony-stimulating factor dysfunction. Chest, 136, 571-7.


### Edit history

| Date | Action | Author |
|---|---|---|
| 2015-04-20 | Edited | Jassal B |
| 2015-04-20 | Authored | Jassal B |
| 2015-04-20 | Created | Jassal B |
| 2015-08-17 | Reviewed | D'Eustachio P |

| Date | Action | Author |
| --- | --- | --- |
| 2023-03-08 | Modified | Matthews L |

## 1 submitted entities found in this pathway, mapping to 1 Reactome entities

| Input | UniProt Id |
| --- | --- |
| Csf2rb | P32927 |

## 16. Defective CSF2RA causes SMDP4 (R-HSA-5688890)

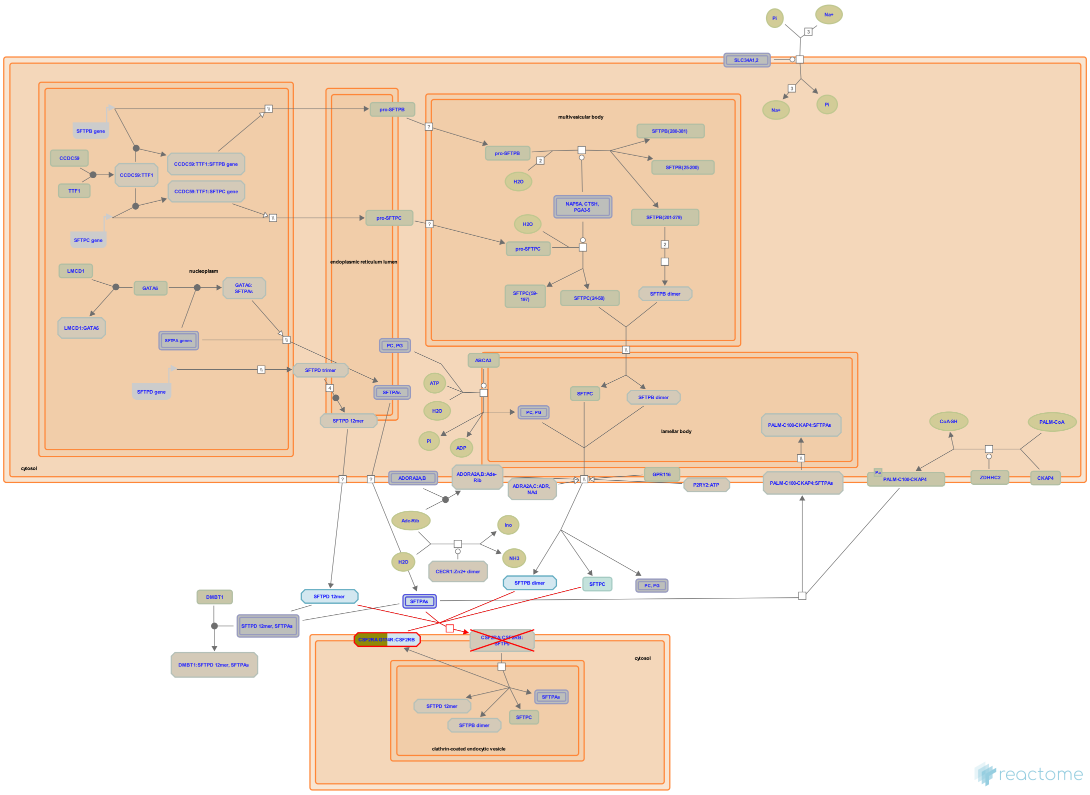

**Diseases:** newborn respiratory distress syndrome, pulmonary alveolar proteinosis.

Surfactant catabolism by alveolar macrophages plays a small but critical part in surfactant recycling and metabolism. Upon ligand binding, granulocyte-macrophage colony-stimulating factor receptor (GM-CSFR), a heterodimer of alpha (CSF2RA) and beta (CSF2RB) subunits, initiates a signalling process that not only induces proliferation, differentiation and functional activation of hematopoietic cells but can also determine surfactant uptake into alveolar macrophages and its degradation via clathrin-coated vesicles. Defects in human CSF2RA can cause pulmonary surfactant metabolism dysfunction 4 (SMDP4; MIM:300770, aka congenital pulmonary alveolar proteinosis, (PAP)), a rare lung disorder due to impaired surfactant homeostasis characterised by alveoli filling with floccular material. Cellular responses to the misfolded pro-SFTPC products include ER stress, the activation of reactive oxygen species and autophagy. Excessive lipoprotein accumulation in the alveoli results in a form of respiratory distress syndrome in premature infants (RDS; MIM:267450) (Whitsett et al. 2015).

### References


Whitsett JA, Weaver TE & Wert SE (2015). Diseases of pulmonary surfactant homeostasis. Annu Rev Pathol, 10, 371-93.


### Edit history

| Date | Action | Author |
| --- | --- | --- |
| 2015-04-20 | Edited | Jassal B |
| 2015-04-20 | Authored | Jassal B |
| 2015-04-20 | Created | Jassal B |

| Date | Action | Author |
|---|---|---|
| 2015-08-17 | Reviewed | D'Eustachio P |
| 2023-03-08 | Modified | Matthews L |

## 1 submitted entities found in this pathway, mapping to 1 Reactome entities

| Input | UniProt Id |
|---|---|
| Csf2rb | P32927 |

## 17. CLEC7A/inflammasome pathway (R-HSA-5660668)

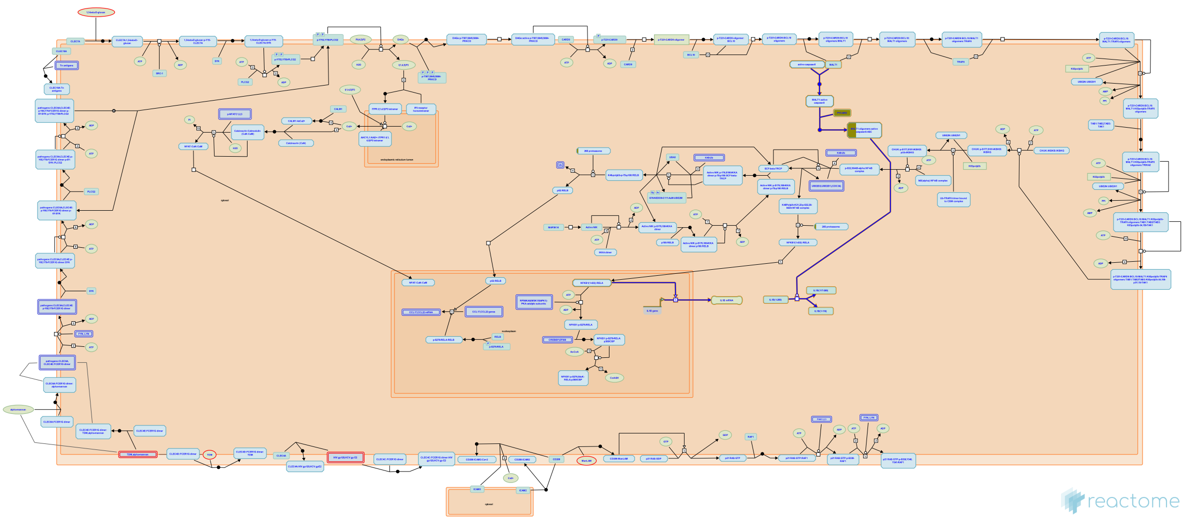

**Cellular compartments:** plasma membrane, cytosol.

Antifungal immunity through the induction of T-helper 17 cells (TH17) responses requires the production of mature, active interleukin-1beta (IL1B). CLEC7A (dectin-1) through the SYK route induces activation of NF-kB and transcription of the gene encoding pro-IL1B via the CARD9-BCL10-MALT1 complex as well as the formation and activation of a MALT1-caspase-8-ASC complex that mediated the processing of pro-IL1B. The inactive precursor pro-IL1B has to be processed into mature bioactive form of IL1B and is usually mediated by inflammatory cysteine protease caspase-1. Gringhuis et al. showed that CLEC7A mediated processing of IL1B occurs through two distinct mechanisms: CLEC7A triggering induced a primary noncanonical caspase-8 inflammasome for pro-IL1B processing that was independent of caspase-1 activity, whereas some fungi triggered a second additional mechanism that required activation of the NLRP3/caspase 1 inflammasome. Unlike the canonical caspase-1 inflammasome, CLEC7A mediated noncanonical caspase-8-dependent inflammasome is independent of pathogen internalization. CLEC7A/inflammasome pathway enables the host immune system to mount a protective TH17 response against fungi and bacterial infection (Gringhuis et al. 2012, Cheng et al. 2011).

### References


Cavalieri D, Stoffels M, Netea MG, Preechasuth K, van de Veerdonk FL, Kanneganti TD, ... Lenardon M (2011). The dectin-1/inflammasome pathway is responsible for the induction of protective T-helper 17 responses that discriminate between yeasts and hyphae of Candida albicans. J. Leukoc. Biol., 90, 357-66.

Geijtenbeek TB, Gringhuis SI, Boekhout T, Theelen B, Wevers BA, van der Vlist M & Kaptein TM (2012). Dectin-1 is an extracellular pathogen sensor for the induction and processing of IL-1? via a noncanonical caspase-8 inflammasome. Nat. Immunol., 13, 246-54.


### Edit history

| Date | Action | Author |
|---|---|---|
| 2014-09-02 | Reviewed | Geijtenbeek TB |
| 2015-01-05 | Edited | Garapati P V |
| 2015-01-05 | Authored | Garapati P V |

| Date | Action | Author |
|---|---|---|
| 2015-01-05 | Created | Garapati P V |
| 2023-05-30 | Modified | Wright A |

## 1 submitted entities found in this pathway, mapping to 1 Reactome entities

| Input | UniProt Id |
|---|---|
| Pycard | Q9ULZ3 |

## 18. Platelet activation, signaling and aggregation (R-HSA-76002)

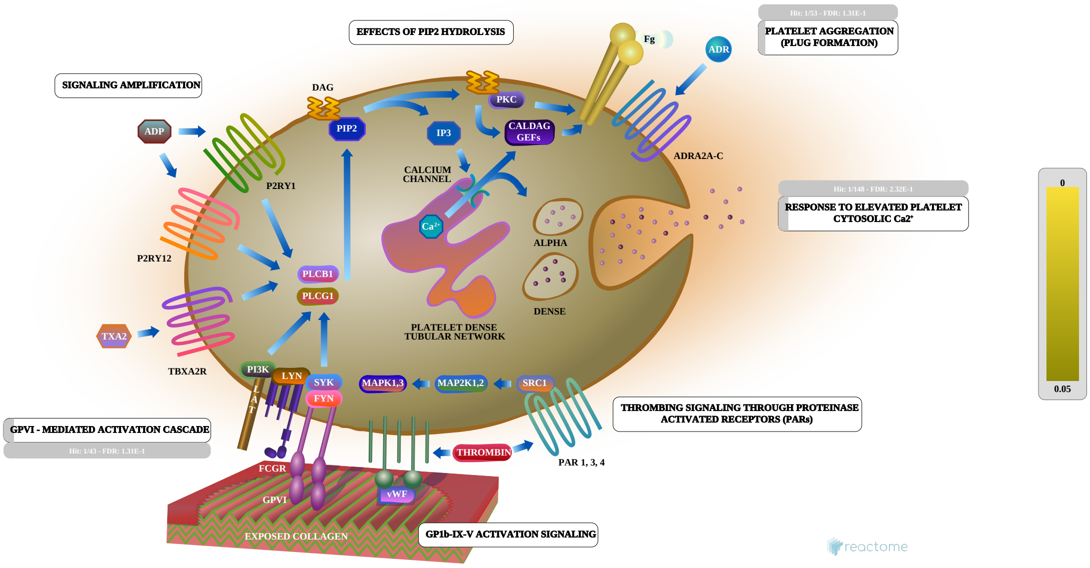

Platelet activation begins with the initial binding of adhesive ligands and of the excitatory platelet agonists (released or generated at the sites of vascular trauma) to cognate receptors on the platelet membrane (Ruggeri 2002). Intracellular signaling reactions then enhance the adhesive and procoagulant properties of tethered platelets or of platelets circulating in the proximity. Once platelets have adhered they degranulate, releasing stored secondary agents such as ADP, ATP, and synthesize thromboxane A2. These amplify the response, activating and recruiting further platelets to the area and promoting platelet aggregation. These amplify the response, activating and recruiting further platelets to the area and promoting platelet aggregation. Adenosine nucleotides signal through P2 purinergic receptors on the platelet membrane. ADP activates P2Y1 and P2Y12, which signal via both the alpha and gamma:beta components of the heterotrimeric G-protein (Hirsch et al. 2001, 2006),
while ATP activates the ionotropic P2X1 receptor (Kunapuli et al. 2003). Activation of these receptors initiates a complex signaling cascade that ultimately results in platelet activation, aggregation and thrombus formation (Kahner et al. 2006).

Integrin AlphaIIbBeta3 is the most abundant platelet receptor, with 40 000 to 80 000 copies per resting platelet, acting as a major receptor for fibrinogen and other adhesive molecules (Wagner et al. 1996). Activation of AlphaIIbBeta3 enhances adhesion and leads to platelet-platelet interactions, and thus aggregation (Philips et al. 1991). GP VI is the most potent collagen receptor initiating signal generation, an ability derived from its interaction with the FcRI gamma chain. This results in the phosphorylation of the gamma-chain by non-receptor tyrosine kinases of the Src family (1). The phosphotyrosine motif is recognized by the SH2 domains of Syk, a tyrosine kinase. This association activates the Syk enzyme, leading to activation (by tyrosine phosphorylation) of PLC gamma2 (2). Thrombin is an important platelet agonist generated on the membrane of stimulated platelets. Thrombin acts via cell surface Protease Activated Receptors (PARs). PARs are G-protein coupled receptors activated by a proteolytic cleavage in an extracellular loop (Vu, 1991) (3). Activated PARs signal via G alpha q (4) and via the beta:gamma component of the G-protein (5). Both stimulate PLC giving rise to PIP2 hydrolysis and consequent activation of PI3K (6). PLCgamma2 activation also gives rise to IP3 (7) which stimulates the IP3 receptor (8) leading to increased intracellular calcium. Platelet activation further results in the scramblase-mediated transport of negatively-charged phospholipids to the platelet surface. These phospholipids provide a catalytic surface (with the charge provided by phosphatidylserine and phosphatidylethanolamine) for the tenase complex (formed by the activated forms of the blood coagulation factors factor VIII and factor I).

## References


Kahner BN, Prasad GL, Kunapuli SP, Shankar H & Murugappan S (2006). Nucleotide receptor signaling in platelets. J Thromb Haemost, 4, 2317-26.

Wymann M, Laffargue M, Tropel P, Hirsch E, Bosco O, Altruda F, ... Calvez R (2001). Resistance to thromboembolism in PI3Kgamma-deficient mice. FASEB J, 15, 2019-21.

Hirsch E, Costa C, Rommel C, Montrucchio G, Barberis L & Lembo G (2006). Signaling through PI3Kgamma: a common platform for leukocyte, platelet and cardiovascular stress sensing. Thromb Haemost, 95, 29-35.

Dorsam RT, Kunapuli SP, Quinton TM & Kim S (2003). Platelet purinergic receptors. Curr Opin Pharmacol, 3, 175-80.

Wheaton VI, Vu TK, Coughlin SR & Hung DT (1991). Molecular cloning of a functional thrombin receptor reveals a novel proteolytic mechanism of receptor activation. Cell, 64, 1057-68.

## Edit history

| Date | Action | Author |
|---|---|---|
| 2004-08-13 | Authored | de Bono B |
| 2004-09-25 | Created | Farndale R, Pace NP, de Bono B |
| 2010-06-07 | Revised | Jupe S |
| 2010-06-07 | Reviewed | Kunapuli SP |
| 2023-05-21 | Modified | Wright A |

## 3 submitted entities found in this pathway, mapping to 3 Reactome entities

| Input | UniProt Id | Input | UniProt Id | Input | UniProt Id |
|---|---|---|---|---|---|
| Apbb1ip | Q7Z5R6 | Plek | P08567 | Vav1 | P15498 |

## 19. ChREBP activates metabolic gene expression (R-HSA-163765)

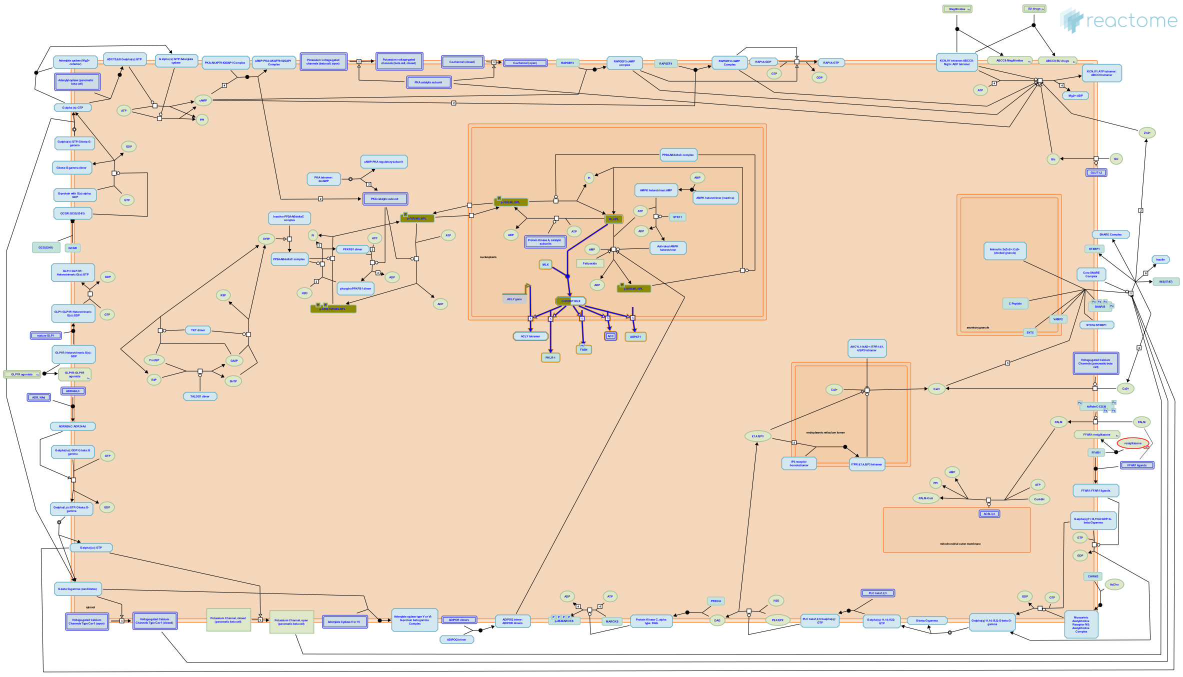

**Cellular compartments:** endoplasmic reticulum membrane, nucleoplasm, cytosol.

ChREBP (Carbohydrate Response Element Binding Protein) is a large multidomain protein containing a nuclear localization signal near its amino terminus, polyproline domains, a basic helix-loop-helix-leucine zipper domain, and a leucine-zipper-like domain (Uyeda et al., 2002). Its dephosphorylation in response to molecular signals associated with the well-fed state allows it to enter the nucleus, interact with MLX protein, and bind to ChRE DNA sequence motifs near Acetyl-CoA carboxylase, Fatty acid synthase, and Pyruvate kinase (L isoform) genes (Ishi et al.2004). This sequence of events is outlined schematically in the picture below (adapted from Kawaguchi et al. (2001) - copyright (2001) National Academy of Sciences, U.S.A.).

### References


Uyeda K, Horton JD, Iizuka K, Liang G & Bruick RK (2004). Deficiency of carbohydrate response element-binding protein (ChREBP) reduces lipogenesis as well as glycolysis. Proc Natl Acad Sci U S A, 101, 7281-6.

Uyeda K, Kabashima T, Kawaguchi T & Takenoshita M (2001). Glucose and cAMP regulate the L-type pyruvate kinase gene by phosphorylation/dephosphorylation of the carbohydrate response element binding protein. Proc Natl Acad Sci U S A, 98, 13710-5.

Ma L, Tsatsos NG & Towle HC (2005). Direct role of ChREBP.Mlx in regulating hepatic glucose-responsive genes. J Biol Chem, 280, 12019-27.


### Edit history

| Date | Action | Author |
|---|---|---|
| 2005-05-06 | Created | Gopinathrao G |
| 2005-05-13 | Authored | Gopinathrao G |
| 2023-05-21 | Modified | Wright A |

## 1 submitted entities found in this pathway, mapping to 1 Reactome entities

| Input | UniProt Id |
| --- | --- |
| Mlxipl | Q9NP71 |

## 20. PP2A-mediated dephosphorylation of key metabolic factors (R-HSA-163767)

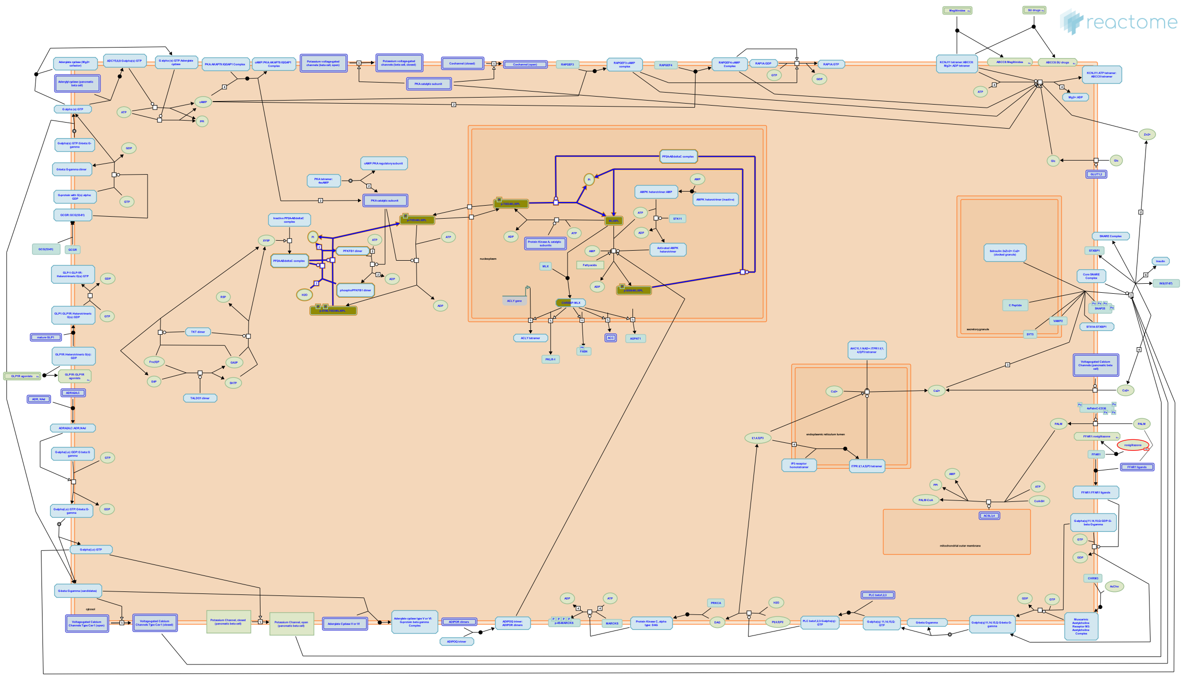

**Cellular compartments:** nucleoplasm, cytosol.

A member of the PP2A family of phosphatases dephosphorylates both cytosolic and nuclear forms of ChREBP (Carbohydrate Response Elemant Binding Protein). In the nucleus, dephosphorylated ChREBP complexes with MLX protein and binds to ChRE sequence elements in chromosomal DNA, activating transcription of genes involved in glycolysis and lipogenesis. The phosphatase is activated by Xylulose-5-phosphate, an intermediate of the pentose phosphate pathway (Kabashima et al. 2003). The rat enzyme has been purified to homogeneity and shown by partial amino acid sequence analysis to differ from previously described PP2A phosphatases (Nishimura and Uyeda 1995) - the human enzyme has not been characterized.

### References


Veech RL (2003). A humble hexose monophosphate pathway metabolite regulates short- and long-term control of lipogenesis. Proc Natl Acad Sci U S A, 100, 5578-80.


### Edit history

| Date | Action | Author |
|---|---|---|
| 2005-05-06 | Created | Gopinathrao G |
| 2005-05-13 | Authored | Gopinathrao G |
| 2023-03-08 | Modified | Matthews L |

### 1 submitted entities found in this pathway, mapping to 1 Reactome entities

| Input | UniProt Id |
|---|---|
| Mlxipl | Q9NP71 |

## 21. RAF/MAP kinase cascade (R-HSA-5673001)

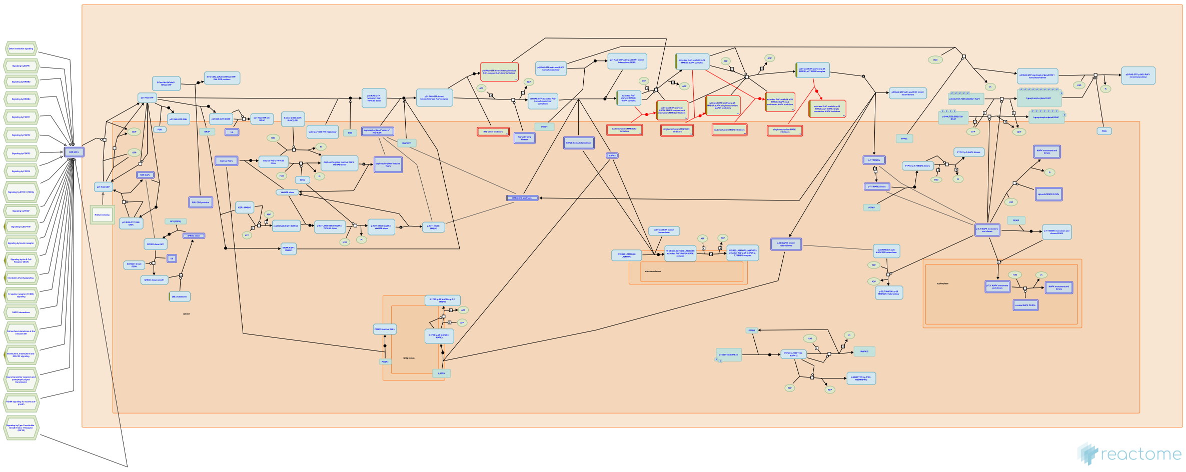

The RAS-RAF-MEK-ERK pathway regulates processes such as proliferation, differentiation, survival, senescence and cell motility in response to growth factors, hormones and cytokines, among others. Binding of these stimuli to receptors in the plasma membrane promotes the GEF-mediated activation of RAS at the plasma membrane and initiates the three-tiered kinase cascade of the conventional MAPK cascades. GTP-bound RAS recruits RAF (the MAPK kinase kinase), and promotes its dimerization and activation (reviewed in Cseh et al, 2014; Roskoski, 2010; McKay and Morrison, 2007; Wellbrock et al, 2004). Activated RAF phosphorylates the MAPK kinase proteins MEK1 and MEK2 (also known as MAP2K1 and MAP2K2), which in turn phophorylate the proline-directed kinases ERK1 and 2 (also known as MAPK3 and MAPK1) (reviewed in Roskoski, 2012a, b; Kryiakis and Avruch, 2012). Activated ERK proteins may undergo dimerization and have identified targets in both the nucleus and the cytosol; consistent with this, a proportion of activated ERK protein relocalizes to the nucleus in response to stimuli (reviewed in Roskoski 2012b; Turjanski et al, 2007; Plotnikov et al, 2010; Cargnello et al, 2011). Although initially seen as a linear cascade originating at the plasma membrane and culminating in the nucleus, the RAS/RAF MAPK cascade is now also known to be activated from various intracellular location. Temporal and spatial specificity of the cascade is achieved in part through the interaction of pathway components with numerous scaffolding proteins (reviewed in McKay and Morrison, 2007; Brown and Sacks, 2009).

The importance of the RAS/RAF MAPK cascade is highlighted by the fact that components of this pathway are mutated with high frequency in a large number of human cancers. Activating mutations in RAS are found in approximately one third of human cancers, while ~8% of tumors express an activated form of BRAF (Roberts and Der, 2007; Davies et al, 2002; Cantwell-Dorris et al, 2011).

### References


Roskoski R Jr (2012). MEK1/2 dual-specificity protein kinases: structure and regulation. Biochem. Biophys. Res. Commun., 417, 5-10.

Brown MD & Sacks DB (2009). Protein scaffolds in MAP kinase signalling. Cell. Signal., 21, 462-9.

Cantwell-Dorris ER, Sheils OM & O'Leary JJ (2011). BRAFV600E: implications for carcinogenesis and molecular therapy. Mol. Cancer Ther., 10, 385-94.

Maitland N, Jayatilake H, Futreal PA, Yuen ST, Marshall CJ, Menzies A, ... Pritchard-Jones K (2002). Mutations of the BRAF gene in human cancer. Nature, 417, 949-54.

Gutkind JS, Turjanski AG & Vaqué JP (2007). MAP kinases and the control of nuclear events. Oncogene, 26, 3240-53.

## Edit history

| Date | Action | Author |
|---|---|---|
| 2015-02-06 | Created | Rothfels K |
| 2015-02-12 | Edited | Rothfels K |
| 2015-02-12 | Authored | Rothfels K |
| 2015-04-29 | Reviewed | Roskoski R Jr |
| 2023-05-30 | Modified | Wright A |

## 3 submitted entities found in this pathway, mapping to 3 Reactome entities

| Input | UniProt Id | Input | UniProt Id | Input | UniProt Id |
|---|---|---|---|---|---|
| Apbb1ip | Q7Z5R6 | Csf2rb | P32927 | Psmb9 | P28065 |

## 22. CLEC7A (Dectin-1) signaling (R-HSA-5607764)

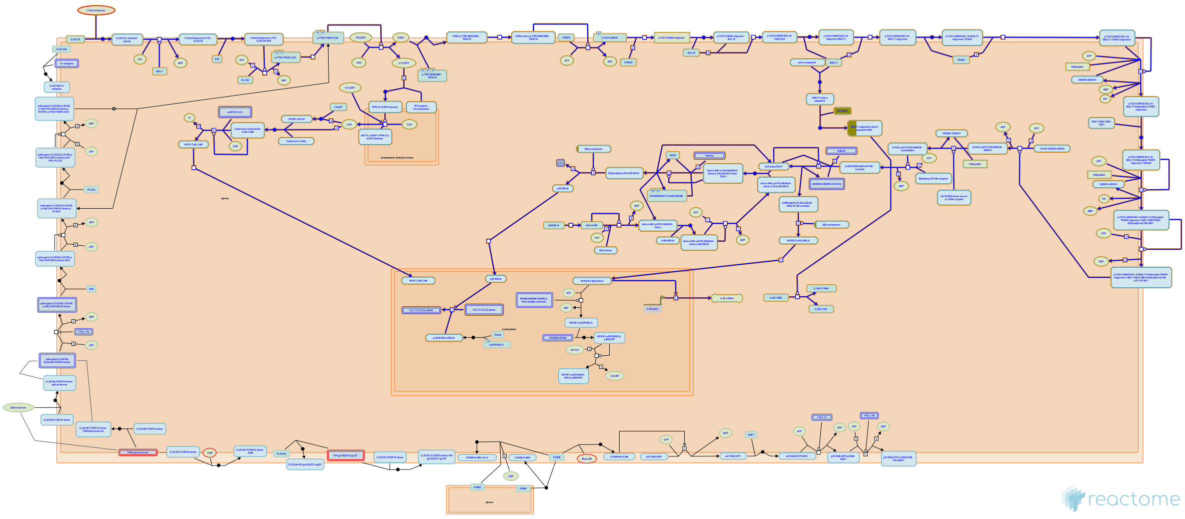

**Cellular compartments:** plasma membrane.

CLEC7A (also known as Dectin-1) is a pattern-recognition receptor (PRR) expressed by myeloid cells (macrophages, dendritic cells and neutrophils) that detects pathogens by binding to beta-1,3-glucans in fungal cell walls and triggers direct innate immune responses to fungal and bacterial infections. CLEC7A belongs to thetype-II C-type lectin receptor (CLR) family that can mediate its own intracellular signaling. Upon binding particulate beta-1,3-glucans, CLEC7A mediates intracellular signalling through its cytoplasmic immunoreceptor tyrosine-based activation motif (ITAM)-like motif (Brown 2006). CLEC7A signaling can induce the production of various cytokines and chemokines, including tumour-necrosis factor (TNF), CXC-chemokine ligand 2 (CXCL2, also known as MIP2), interleukin-1beta (IL-1b), IL-2, IL-10 and IL-12 (Brown et al. 2003), it also triggers phagocytosis and stimulates the production of reactive oxygen species (ROS), thus contributing to microbial killing (Gantner et al. 2003, Herre et al. 2004, Underhill et al. 2005, Goodridge at al. 2011, Reid et al. 2009). These cellular responses mediated by CLEC7A rely on both Syk-dependent and Syk-independent signaling cascades. The pathways leading to the Syk-dependent activation of NF-kB can be categorised into both canonical and non-canonical routes (Gringhuis et al. 2009). Activation of the canonical NF-kB pathway is essential for innate immunity, whereas activation of the non-canonical pathway is involved in lymphoid organ development and adaptive immunity (Plato et al. 2013).

### References


Gordon S, Williams DL, Willment JA, Marshall AS, Brown GD & Herre J (2003). Dectin-1 mediates the biological effects of beta-glucans. J. Exp. Med., 197, 1119-24.

Brown GD, Gow NA & Reid DM (2009). Pattern recognition: recent insights from Dectin-1. Curr. Opin. Immunol., 21, 30-7.


### Edit history

| Date | Action | Author |
|---|---|---|
| 2014-07-14 | Edited | Garapati P V |
| 2014-07-14 | Authored | Garapati P V |
| 2014-07-14 | Created | Garapati P V |
| 2014-09-02 | Reviewed | Geijtenbeek TB |

| Date | Action | Author |
| --- | --- | --- |
| 2023-05-21 | Modified | Wright A |

## 2 submitted entities found in this pathway, mapping to 2 Reactome entities

| Input | UniProt Id | Input | UniProt Id |
| --- | --- | --- | --- |
| Psmb9 | P28065 | Pycard | Q9ULZ3 |

## 23. AMPK inhibits chREBP transcriptional activation activity (R-HSA-163680)

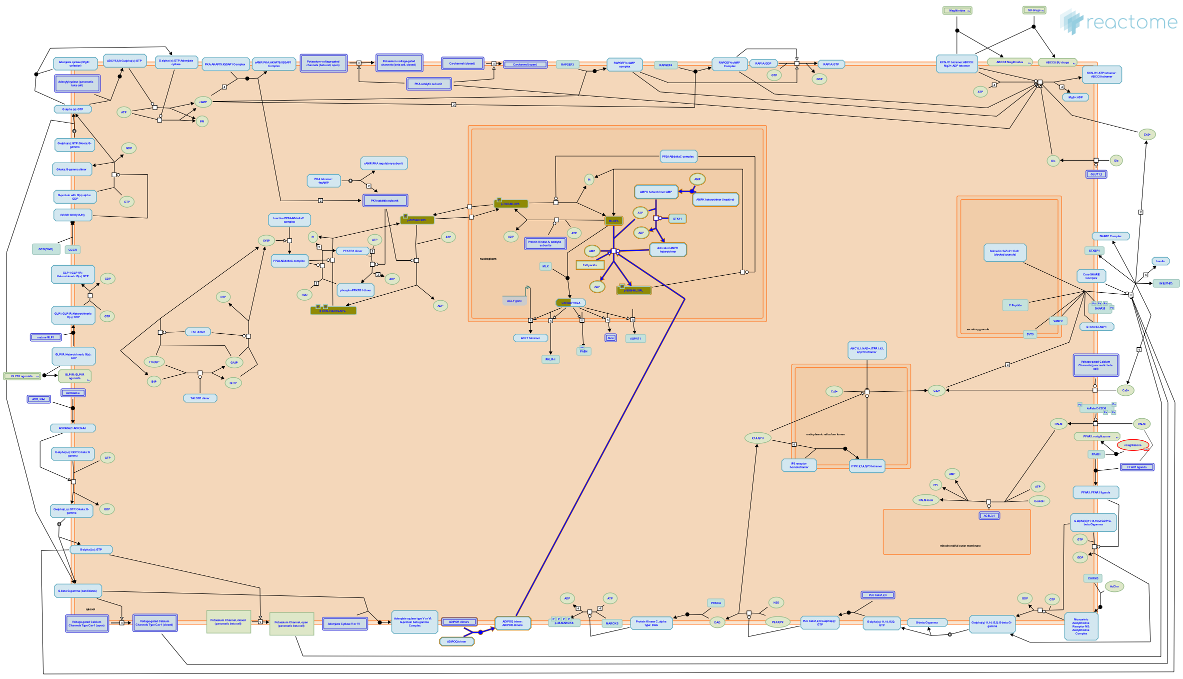

**Cellular compartments:** nucleoplasm.

AMP-activated protein kinase (AMPK) is a sensor of cellular energy levels. A high cellular ratio of AMP:ATP triggers the phosphorylation and activation of AMPK. Activated AMPK in turn phosphorylates a wide array of target proteins, as shown in the figure below (reproduced from (Hardie et al. 2003), with the permission of D.G. Hardie). These targets include ChREBP (Carbohydrate Response Element Binding Protein), whose inactivation by phosphorylation reduces transcription of key enzymes of the glycolytic and lipogenic pathways.

### References


Hudson ER, Scott JW, Pan DA & Hardie DG (2003). Management of cellular energy by the AMP-activated protein kinase system. FEBS Lett, 546, 113-20.

Uyeda K, Osatomi K, Yamashita H, Kabashima T & Kawaguchi T (2002). Mechanism for fatty acid sparing effect on glucose-induced transcription: regulation of carbohydrate-responsive element-binding protein by AMP-activated protein kinase. J Biol Chem, 277, 3829-35.

Hardie DG (2004). The AMP-activated protein kinase pathway--new players upstream and downstream. J Cell Sci, 117, 5479-87.

Carling D, Davies SP, Salt IP, Cheung PC & Hardie DG (2000). Characterization of AMP-activated protein kinase gamma-subunit isoforms and their role in AMP binding. Biochem J, 346, 659-69.


### Edit history

| Date | Action | Author |
|---|---|---|
| 2005-05-05 | Created | Gopinathrao G |
| 2005-05-13 | Authored | Gopinathrao G |
| 2023-05-21 | Modified | Wright A |

## 1 submitted entities found in this pathway, mapping to 1 Reactome entities

| Input | UniProt Id |
|---|---|
| Mlxipl | Q9NP71 |

## 24. MAPK1/MAPK3 signaling (R-HSA-5684996)

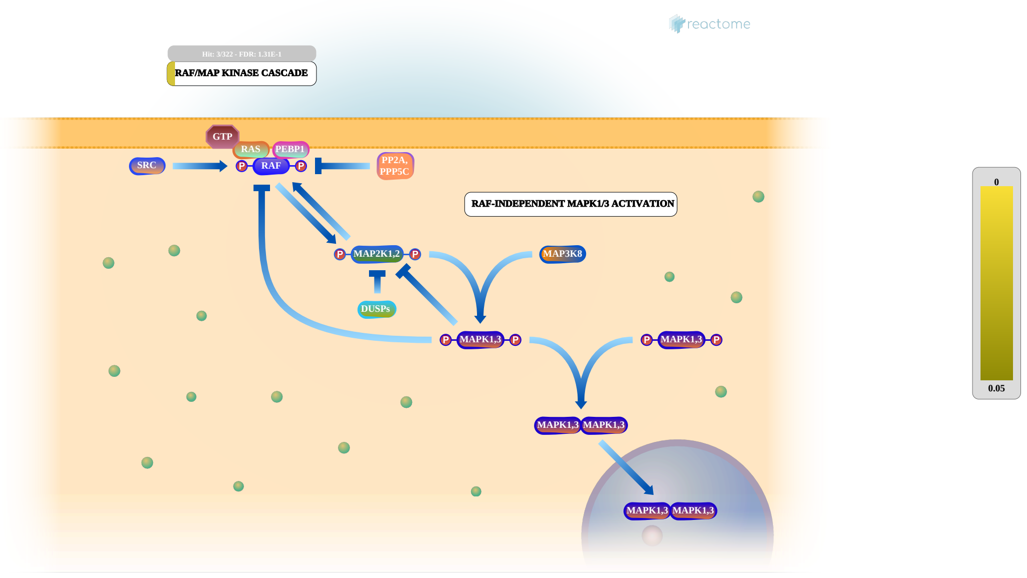


The extracellular signal regulated kinases (ERKs) 1 and 2, also known as MAPK3 and MAPK1, are phosphorylated by the MAP2Ks 1 and 2 in response to a wide range of extracellular stimuli to promote differentiation, proliferation, cell motility, cell survivial, metabolism and transcription, among others (reviewed in Roskoski, 2012b; McKay and Morrison, 2007; Raman et al, 2007). In the classical pathway, MAPK1/3 activation is triggered by the GEF-mediated activation of RAS at the plasma membrane, leading to the activation of the RAF MAP3Ks (reviewed in McKay and Morrison, 2007; Matallanas et al, 2011; Wellbrock et al, 2004). However, many physiological and pathological stimuli have been found to activate MAPK1/3 independently of RAF and RAS, acting instead through MAP3Ks such as MOS, TPL2 and AMPK (Dawson et al, 2008; Wang et al, 2009; Kuriakose et al, 2014; Awane et al, 1999). Activated MAPK1/3 phosphorylate numerous targets in both the nucleus and cytoplasm (reviewed in Yoon and Seger, 2006; Roskoski 2012b).

### References


Wellbrock C, Karasarides M & Marais R (2004). The RAF proteins take centre stage. Nat Rev Mol Cell Biol, 5, 875-85.

Whiteman MW, Lian H, Denmark T, Huang D, Wang J, Singh A & Wang G (2009). A non-canonical MEK/ERK signaling pathway regulates autophagy via regulating Beclin 1. J. Biol. Chem., 284, 21412-24.

Morris MA, Tramoutanis G, Laverick L, Dawson CW & Young LS (2008). Epstein-Barr virus-encoded LMP1 regulates epithelial cell motility and invasion via the ERK-MAPK pathway. J. Virol., 82, 3654-64.

Kuriakose T, Rada B & Watford WT (2014). Tumor progression locus 2-dependent oxidative burst drives phosphorylation of extracellular signal-regulated kinase during TLR3 and 9 signaling. J. Biol. Chem., 289, 36089-100.

Chen W, Raman M & Cobb MH (2007). Differential regulation and properties of MAPKs. Oncogene, 26, 3100-12.


### Edit history

| Date | Action | Author |
|---|---|---|
| 2015-03-11 | Authored | Rothfels K |
| 2015-03-24 | Created | Rothfels K |
| 2015-04-29 | Reviewed | Roskoski R Jr |
| 2023-05-21 | Modified | Wright A |

## 3 submitted entities found in this pathway, mapping to 3 Reactome entities

| Input | UniProt Id | Input | UniProt Id | Input | UniProt Id |
|---|---|---|---|---|---|
| Apbb1ip | Q7Z5R6 | Csf2rb | P32927 | Psmb9 | P28065 |

## 25. Antigen processing-Cross presentation (R-HSA-1236975)

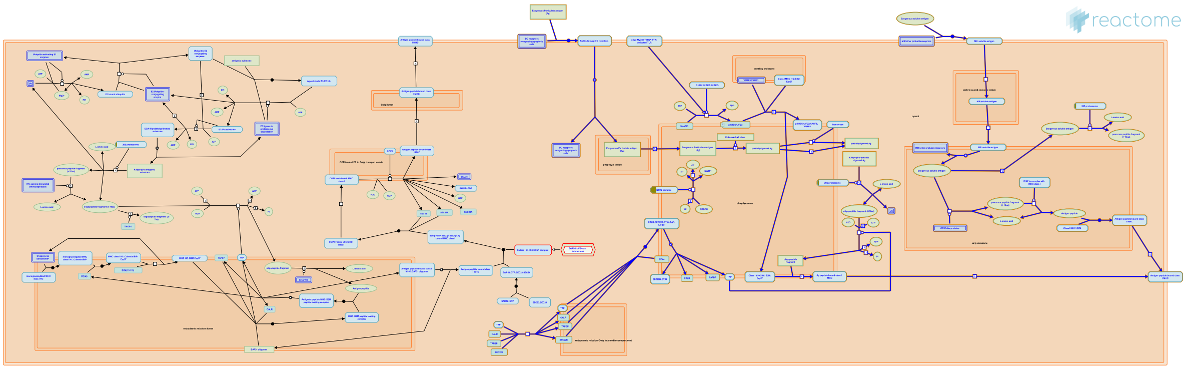

MHC class I molecules generally present peptide antigens derived from proteins synthesized by the cell itself to CD8+ T cells. However, in some circumstances, antigens from extracellular environment can be presented on MHC class I to stimulate CD8+ T cell immunity, a process termed cross-presentation (Rock & Shen. 2005). Cross-presentation/cross-priming is the ability of antigen presenting cells (APCs) to present exogenous antigens on MHC class I molecules to CD8+ T lymphocytes. Among all the APCs, Dendritic cells (DC) are the dominant antigen cross presenting cell types in vivo, although macrophages and B cells appear to cross present model antigens in vitro with a low degree of efficiency (Amigorena & Savina. 2010, Ackermann & Peter Cresswell. 2004). Compared to macrophages, DCs have low levels of lysosomal proteases and exhibit limited lysosomal degradation (Delamarre et al. 2005). This limited proteolysis of internalized antigens by DCs might contribute to their high efficiency for cross presentation (Monua & Trombetta. 2007). APCs acquire the exogenous antigens through endocytic mechanisms, especially phagosomes for particulate/cell-associated antigens and endosomes for soluble protein antigens. There does not seem to be a unique pathway for cross-presentation but rather different potential mechanisms of cross-presentation have been proposed. These proposed pathways can be classified according to the location where two key events occur: 1) processing of the antigenic protein and 2) loading of the processed peptide on to MHC I molecule (Blanchard & Shastri. 2010). Based on the requirement for TAP and cytosolic proteases two mechanisms have been described, a cytosolic pathway (TAP-dependent and proteasome-dependent) or a vacuolar pathway (TAP- and proteasome-independent) (Blanchard & Shastri. 2010, Amigorena & Savina. 2010). Regarding peptide-loading, MHC I could be loaded in the ER or in the phagosome and recycled to cell surface (Blanchard & Shastri. 2010). Exogenous soluble antigens are cross-presented by dendritic cells, albeit with lower efficiency than for particulate substrates. Soluble antigens destined for cross-presentation are taken up by distinct endocytosis mechanisms which route them into stable early endosomes and then to the cytoplasm for proteasomal degradation and peptide loading. The outcome of the cross presentation can be either tolerance or immunity (Rock & Shen. 2005).

### References


Rock KL & Shen L (2005). Cross-presentation: underlying mechanisms and role in immune surveillance. Immunol Rev, 207, 166-83.

Savina A & Amigorena S (2010). Intracellular mechanisms of antigen cross presentation in dendritic cells. Curr Opin Immunol, 22, 109-17.

Trombetta ES & Monu N (2007). Cross-talk between the endocytic pathway and the endoplasmic reticulum in cross-presentation by MHC class I molecules. Curr Opin Immunol, 19, 66-72.

Rahner C, Giodini A & Cresswell P (2009). Receptor-mediated phagocytosis elicits cross-presentation in nonprofessional antigen-presenting cells. Proc Natl Acad Sci U S A, 106, 3324-9.

Trombetta ES, Pack M, Mellman I, Chang H & Delamarre L (2005). Differential lysosomal proteolysis in antigen-presenting cells determines antigen fate. Science, 307, 1630-4.

## Edit history

| Date | Action | Author |
|---|---|---|
| 2011-03-28 | Edited | Garapati P V |
| 2011-03-28 | Authored | Garapati P V |
| 2011-03-28 | Created | Garapati P V |
| 2011-05-13 | Reviewed | Desjardins M, English L |
| 2023-05-21 | Modified | Wright A |

## 2 submitted entities found in this pathway, mapping to 2 Reactome entities

| Input | UniProt Id | Input | UniProt Id |
|---|---|---|---|
| Ncf4 | Q15080 | Psmb9 | P28065 |

# 6. Identifiers found

Below is a list of the input identifiers that have been found or mapped to an equivalent element in Reactome, classified by resource.

### 17 of the submitted entities were found, mapping to 19 Reactome entities

| Input | UniProt Id | Input | UniProt Id | Input | UniProt Id |
|---|---|---|---|---|---|
| Adora3 | P0DMS8 | Apbb1ip | Q7Z5R6 | Csf2rb | P32927 |
| Cyth4 | Q9UIA0 | Gusb | P08236 | Havcr2 | Q8TDQ0 |
| Hexb | P07686 | Hk3 | P52790 | Irf5 | Q13568 |
| Lair1 | Q6GTX8 | Mlxipl | Q9NP71 | Ncf4 | Q15080 |
| Plek | P08567 | Psmb9 | P28065 | Pycard | Q9ULZ3 |
| Slc14a1 | Q13336 | Vav1 | P15498 | | |

| Input | Ensembl Id |
|---|---|
| Irf5 | ENSG00000128604 |

# 7. Identifiers not found

These 8 identifiers were not found neither mapped to any entity in Reactome.

| | | | | | | | |
|---|---|---|---|---|---|---|---|
| Aif1 | Ccl9 | Fcrls | Gpr34 | Ifi27l2a | Mir142hg | Oas1a | Tlr12 |
