# Supplementary material for: Sex- and region-specific cortical and hippocampal whole genome transcriptome profiles from control and APP/PS1 Alzheimer’s disease mice
Source: PLoS One. 2024 Feb 7;19(2):e0296959. doi: 10.1371/journal.pone.0296959 (PMC10849391; doi:10.1371/journal.pone.0296959)
Supplement: S1 File — S1 Fig: Genotyping of APP/PS1 AD mice and WT control animals. S2 Fig: 3D image of the murine brain including the RS cortex and hippocampus (BROIs) used for transcriptome analysis in our study. S3 Fig: PCA of transcriptomes from the RS cortex and hippocampus of WT controls and APP/PS1 AD mice of both sexes. S4 Fig: Hierarchical clustering of transcriptome data from the RS cortex and hippocampus of WT control and APP/PS1 AD mice of both sexes. S5 Fig: Bar diagrams of the top 30 candidates of DEGs with highest significant FCs (FC > 1.5 and FC < -1.5, p < 0.05). S6 Fig: Pathway analysis of intersectional and signature gene sets in APP/PS1 subgroups. S7 Fig: Comparative qPCR analysis of selected gene transcript levels from the hippocampus of female and male APP/PS1 AD with 5XFAD mice. S1 Table: PCR reaction set-up using PCR Mastermix and genomic DNA. S2 Table: Materials used for one-color microarray-based gene expression data collection. S3 Table: Software used for one-color microarray-based gene expression data collection. S4 Table: Details on genes, forward and reverse primer sequences and annealing temperatures relevant for qPCR experimentation. S5 Table: Characteristics of DEGs in the RS cortex of female APP/PS1 AD mice. S6 Table: Characteristics of DEGs in the hippocampus of female APP/PS1 AD mice. S7 Table: Characteristics of DEGs in the RS cortex of male APP/PS1 AD mice. S8 Table: Characteristics of DEGs in the hippocampus of male APP/PS1 AD mice. S9 Table: Venn analysis of DEGs in the RS cortex and hippocampus of female APP/PS1 AD mice. S10 Table: Venn analysis of DEGs genes in the RS cortex and hippocampus of male APP/PS1 AD mice. S11 Table: Venn analysis of DEGs in the RS cortex of male and female APP/PS1 AD mice. S12 Table: Venn analysis of DEGs in the hippocampus of male and female APP/PS1 AD mice. S13 Table: Differentially regulated l(i)ncRNAs in APP/PS1 AD vs. WT mice. S14 Table: qPCR-based FC analysis of selected genes in the hippocampus of APP/PS1 AD vs. [file pone.0296959.s001.zip › Supplementary Files_R1/Supplementary Figure 6_Pathways_upreg genes/Signature genes up_DEGs_male_Hip_APPPS1/Pathway analysis report.pdf]

reactome

Pathway Analysis Report

This report contains the pathway analysis results for the submitted sample ''. Analysis was performed against Reactome version 85 on 14/08/2023. The web link to these results is:

https://reactome.org/PathwayBrowser/#/ANALYSIS=MjAyMzA4MTQwOTU1MjFfODA5NQ%3D%3D

Please keep in mind that analysis results are temporarily stored on our server. The storage period depends on usage of the service but is at least 7 days. As a result, please note that this URL is only valid for a limited time period and it might have expired.

Table of Contents

1. Introduction
2. Properties
3. Genome-wide overview
4. Most significant pathways
5. Pathways details
6. Identifiers found
7. Identifiers not found

1. Introduction

Reactome is a curated database of pathways and reactions in human biology. Reactions can be considered as pathway 'steps'. Reactome defines a 'reaction' as any event in biology that changes the state of a biological molecule. Binding, activation, translocation, degradation and classical biochemical events involving a catalyst are all reactions. Information in the database is authored by expert biologists, entered and maintained by Reactome's team of curators and editorial staff. Reactome content frequently cross-references other resources e.g. NCBI, Ensembl, UniProt, KEGG (Gene and Compound), ChEBI, PubMed and GO. Orthologous reactions inferred from annotation for Homo sapiens are available for 14 non-human species including mouse, rat, chicken, puffer fish, worm, fly and yeast. Pathways are represented by simple diagrams following an SBGN-like format.

Reactome's annotated data describe reactions possible if all annotated proteins and small molecules were present and active simultaneously in a cell. By overlaying an experimental dataset on these annotations, a user can perform a pathway over-representation analysis. By overlaying quantitative expression data or time series, a user can visualize the extent of change in affected pathways and its progression. A binomial test is used to calculate the probability shown for each result, and the p-values are corrected for the multiple testing (Benjamini–Hochberg procedure) that arises from evaluating the submitted list of identifiers against every pathway.

To learn more about our Pathway Analysis, please have a look at our relevant publications:

Fabregat A, Sidiropoulos K, Garapati P, Gillespie M, Hausmann K, Haw R, … D'Eustachio P (2016). The reactome pathway knowledgebase. Nucleic Acids Research, 44(D1), D481–D487. https://doi.org/10.1093/nar/gkv1351.

Fabregat A, Sidiropoulos K, Viteri G, Forner O, Marin-Garcia P, Arnau V, … Hermjakob H (2017). Reactome pathway analysis: a high-performance in-memory approach. BMC Bioinformatics, 18.

2. Properties

- This is an **overrepresentation** analysis: A statistical (hypergeometric distribution) test that determines whether certain Reactome pathways are over-represented (enriched) in the submitted data. It answers the question 'Does my list contain more proteins for pathway X than would be expected by chance?' This test produces a probability score, which is corrected for false discovery rate using the Benjamani-Hochberg method.
- 14 out of 18 identifiers in the sample were found in Reactome, where 212 pathways were hit by at least one of them.
- All non-human identifiers have been converted to their human equivalent.
- This report is filtered to show only results for species 'Homo sapiens' and resource 'all resources'.
- The unique ID for this analysis (token) is MjAyMzA4MTQwOTU1MjFfODA5NQ%3D%3D. This ID is valid for at least 7 days in Reactome's server. Use it to access Reactome services with your data.

3. Genome-wide overview

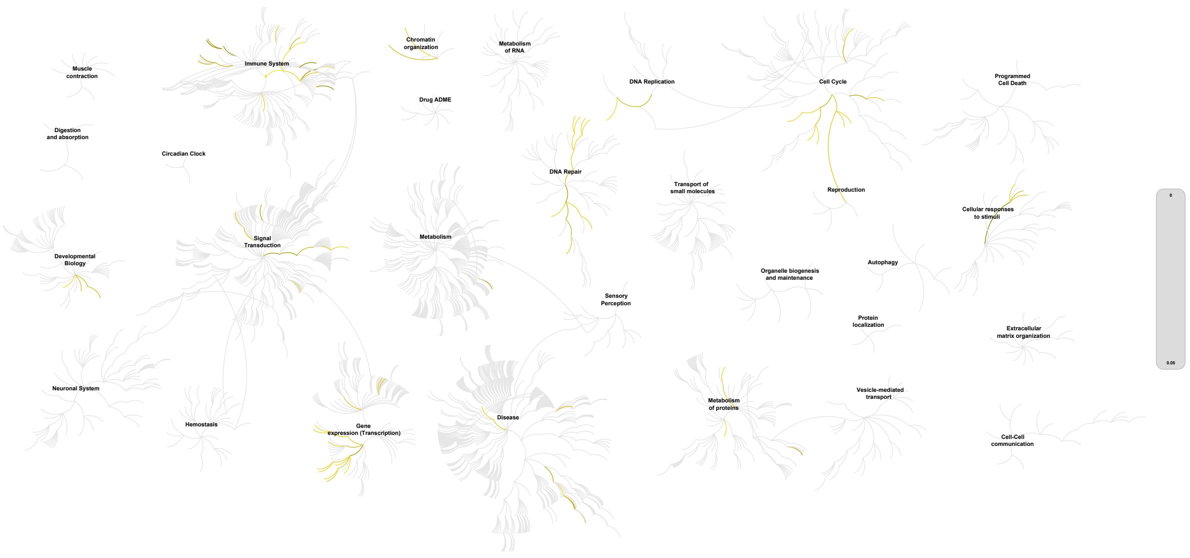

This figure shows a genome-wide overview of the results of your pathway analysis. Reactome pathways are arranged in a hierarchy. The center of each of the circular "bursts" is the root of one top-level pathway, for example "DNA Repair". Each step away from the center represents the next level lower in the pathway hierarchy. The color code denotes over-representation of that pathway in your input dataset. Light grey signifies pathways which are not significantly over-represented.

4. Most significant pathways

The following table shows the 25 most relevant pathways sorted by p-value.

Pathway name	Entities				Reactions	
	found	ratio	p-value	FDR*	found	ratio
Transcriptional regulation of granulopoiesis	5 / 70	0.005	1.52e-07	3.27e-05	19 / 27	0.002
Cytokine Signaling in Immune system	9 / 1,039	0.068	4.82e-05	0.005	36 / 745	0.052
Interferon gamma signaling	4 / 177	0.012	2.58e-04	0.018	2 / 23	0.002
Amyloid fiber formation	3 / 89	0.006	5.25e-04	0.026	3 / 33	0.002
Immune System	12 / 2,627	0.172	9.16e-04	0.026	103 / 1,664	0.116
The role of Nef in HIV-1 replication and disease pathogenesis	2 / 29	0.002	0.001	0.026	5 / 20	0.001
Packaging Of Telomere Ends	2 / 33	0.002	0.002	0.026	2 / 2	1.40e-04
RNA Polymerase I Promoter Opening	2 / 33	0.002	0.002	0.026	1 / 2	1.40e-04
DNA methylation	2 / 35	0.002	0.002	0.026	7 / 7	4.89e-04
Interleukin-37 signaling	2 / 36	0.002	0.002	0.026	1 / 14	9.79e-04
Recognition and association of DNA glycosylase with site containing an affected purine	2 / 38	0.002	0.002	0.026	2 / 10	6.99e-04
Assembly of the ORC complex at the origin of replication	2 / 39	0.003	0.002	0.026	2 / 11	7.69e-04
Interferon Signaling	4 / 322	0.021	0.002	0.026	8 / 79	0.006
Recognition and association of DNA glycosylase with site containing an affected pyrimidine	2 / 41	0.003	0.002	0.026	1 / 21	0.001
PRC2 methylates histones and DNA	2 / 43	0.003	0.003	0.026	4 / 4	2.80e-04
SIRT1 negatively regulates rRNA expression	2 / 44	0.003	0.003	0.026	3 / 5	3.49e-04
Cleavage of the damaged purine	2 / 44	0.003	0.003	0.026	2 / 9	6.29e-04
Depurination	2 / 45	0.003	0.003	0.026	4 / 19	0.001
Inhibition of DNA recombination at telomere	2 / 47	0.003	0.003	0.026	3 / 4	2.80e-04
ERCC6 (CSB) and EHMT2 (G9a) positively regulate rRNA expression	2 / 47	0.003	0.003	0.026	2 / 4	2.80e-04
Activated PKN1 stimulates transcription of AR (androgen receptor) regulated genes KLK2 and KLK3	2 / 48	0.003	0.003	0.026	8 / 11	7.69e-04
Cleavage of the damaged pyrimidine	2 / 50	0.003	0.004	0.026	1 / 20	0.001
Depyrimidination	2 / 50	0.003	0.004	0.026	2 / 41	0.003

Pathway name	Entities				Reactions	
	found	ratio	p-value	FDR*	found	ratio
Defective pyroptosis	2 / 51	0.003	0.004	0.026	1 / 3	2.10e-04
DAP12 interactions	2 / 52	0.003	0.004	0.026	15 / 33	0.002

* False Discovery Rate

5. Pathways details

For every pathway of the most significant pathways, we present its diagram, as well as a short summary, its bibliography and the list of inputs found in it.

1. Transcriptional regulation of granulopoiesis (R-HSA-9616222)

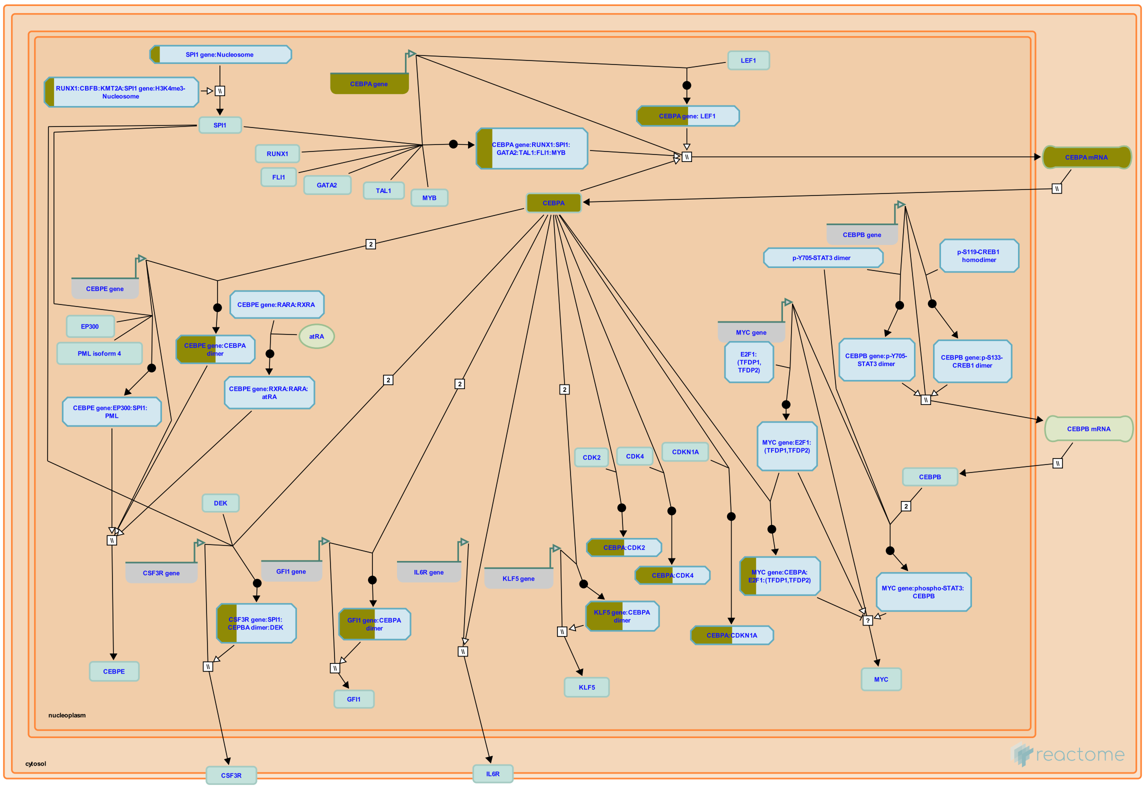

Neutrophilic granulocytes (hereafter called granulocytes) are distinguished by multilobulated nuclei and presence of cytoplasmic granules containing antipathogenic proteins (reviewed in Cowland and Borregaard 2016, Yin and Heit 2018). Granulocytes comprise eosinophils, basophils, mast cells, and neutrophils, all of which are ultimately derived from hemopoietic stem cells (HSCs), a self-renewing population of stem cells located in the bone marrow. A portion of HSCs exit self-renewing proliferation and differentiate to form multipotent progenitors (MPPs). MPPs then differentiate to form common myeloid progenitors (CMPs) as well as the erythrocyte lineage. CMPs further differentiate into granulocyte-monocyte progenitors (GMPs) which can then differentiate into monocytes or any of the types of granulocytes (reviewed in Fiedler and Brunner 2012). granulocytes are the most abundant leukocytes in peripheral blood.

For early granulopoiesis the CEBPA, SPI1 (PU.1), RAR, CBF, and MYB transcription factors are essential. CEBPE, SPI1, SP1, CDP, and HOXA10 transcription factors initiate terminal neutrophil differentiation.

Initially, RUNX1 activates SPI1 (PU.1), which is believed to be the key transcription factor driving the formation of MPPs and CMPs (reviewed in Friedman 2007, Fiedler and Brunner 2012). SPI1, in turn, activates expression of CEBPA, an indispensable transcription factor for granulopoiesis especially important in the transition from CMP to GMP (inferred from mouse homologs in Wilson et al. 2010, Guo et al. 2012, Guo et al. 2014, Cooper et al. 2015). CEBPA, in turn, activates the expression of several transcription factors and receptors characteristic of granulocytes, including CEBPA (autoregulation), CEBPE (Loke et al. 2018, and inferred from mouse homologs in Wang and Friedman 2002, Friedman et al. 2003), GFI1 (inferred from mouse homologs in Lidonnici et al. 2010), KLF5 (Federzoni et al. 2014), IL6R (inferred from mouse homologs in Zhang et al. 1998), and CSF3R (Smith et al. 1996). Importantly, CEBPA dimers repress transcription of MYC (c-Myc) (Johansen et al. 2001, and inferred from mouse homologs in Slomiany et al. 2000, Porse et al. 2001). CEBPA binds CDK2 and CDK4 (Wang et al. 2001) which inhibits their kinase activity by disrupting their association with cyclins thereby limiting proliferation and favoring differentiation of granulocyte progenitors during regular ("steady-state") granulopoiesis (reviewed in Friedman 2015). The transcription factor GFI1 regulates G-CSF signaling and neutrophil development through the Ras activator RasGRP1 (de la Luz Sierra et al. 2010).

Inhibitors of DNA binding (ID) proteins ID1 and ID2 regulate granulopoiesis and eosinophil production such that ID1 induces neutrophil development and inhibits eosinophil differentiation, whereas ID2 induces both eosinophil and neutrophil development (Buitenhuis et al. 2005, Skokowa et al. 2009).

Major infection activates emergency granulopoiesis (reviewed in Manz and Boettcher 2014, Hirai et al. 2015), the production of large numbers of granulocytes in a relatively short period of time. Emergency granulopoiesis is activated by cytokines, CSF2 (GM-CSF) and especially CSF3 (G-CSF, reviewed in Panopoulos and Watowich 2008, Liongue et al. 2009) which bind receptors, CSF2R and CSF3R, respectively, resulting in expression of CEBPB, which interferes with repression of MYC by CEBPA (inferred from mouse homologs in Zhang et al. 2010) and represses MYC less than CEBPA does (Hirai et al. 2006), leading to proliferation of granulocyte progenitors prior to final differentiation.Both, emergency and steady-state granulopoiesis are regulated by direct interaction of CEBPA (steady-state) or CEBPB (emergency) proteins with NAD+-dependent protein deacetylases, SIRT1 and SIRT2 (Skokowa et al. 2009). G-CSF induces the NAD+-generating enzyme, Nicotinamide phosphoribosyltransferase (NAMPT, or PBEF), that in turn activates sirtuins (Skokowa et al. 2009).

GADD45A and GADD45B proteins are essential for stress-induced granulopoiesis and granulocyte chemotaxis by activation of p38 kinase (Gupta et al. 2006, Salerno et al. 2012). SHP2 is required for induction of CEBPA expression and granulopoiesis in response to CSF3 (G-CSF) or other cytokines independent of SHP2-mediated ERK activation (Zhang et al. 2011).

Transcription of neutrophil granule proteins (e.g. ELANE, MPO, AZU1, DEFA4), that play an essential role in bacterial killing are regulated by CEBPE and SPI1 (PU.1) transcription factors (Gombart et al. 2003, Nakajima et al. 2006). RUNX1 and LEF1 also regulate ELANE (ELA2) mRNA expression by binding to its promoter (Li et al. 2003).

References

Slomiany BA, D'Arigo KL, Kurtz DT & Kelly MM (2000). C/EBPalpha inhibits cell growth via direct repression of E2F-DP-mediated transcription. Mol. Cell. Biol., 20, 5986-97.

Datta MW, Zhang P, Tenen DG, Darlington GJ, Iwama A & Link DC (1998). Upregulation of interleukin 6 and granulocyte colony-stimulating factor receptors by transcription factor CCAAT enhancer binding protein alpha (C/EBP alpha) is critical for granulopoiesis. J. Exp. Med., 188, 1173-84.

Boettcher S & Manz MG (2014). Emergency granulopoiesis. Nat. Rev. Immunol., 14, 302-14.

Watanabe N, Shibata F, Nakajima H, Ikeda Y, Handa M & Kitamura T (2006). N-terminal region of CCAAT/enhancer-binding protein epsilon is critical for cell cycle arrest, apoptosis, and functional maturation during myeloid differentiation. J. Biol. Chem., 281, 14494-502.

Yin C & Heit B (2018). Armed for destruction: formation, function and trafficking of neutrophil granules. Cell Tissue Res., 371, 455-471.

Edit history

Date	Action	Author
2018-08-10	Edited	May B
2018-08-10	Authored	May B
2018-08-10	Created	May B
2019-03-10	Reviewed	Skokowa J
2023-05-21	Modified	Wright A

2 submitted entities found in this pathway, mapping to 5 Reactome entities

Input	UniProt Id	Input	UniProt Id
Cebpa	P49715	Hist1h2be	P62807, Q93079

Input	Ensembl Id
Cebpa	ENSG00000245848, ENST00000498907

2. Cytokine Signaling in Immune system (R-HSA-1280215)

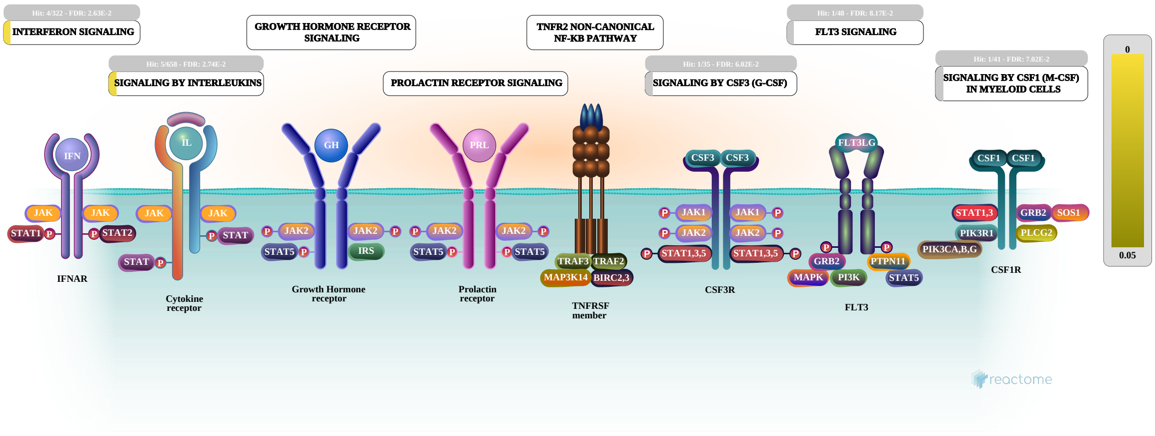

Cytokines are small proteins that regulate and mediate immunity, inflammation, and hematopoiesis. They are secreted in response to immune stimuli, and usually act briefly, locally, at very low concentrations. Cytokines bind to specific membrane receptors, which then signal the cell via second messengers, to regulate cellular activity.

References

Feldmann M & Oppenheim J (2002). *Cytokines and the immune system, Cytokine Reference* .

IMMPORT:Bioinformatics for the future of immunology. Retrieved from https://www.immport.org/immportWeb/queryref/geneListSummary.do

Santamaria P (2003). Cytokines and chemokines in autoimmune disease: an overview. Adv Exp Med Biol, 520, 1-7.

COPE. Retrieved from http://www.copewithcytokines.org/cope.cgi

Edit history

Date	Action	Author
2011-05-12	Created	Garapati P V
2011-05-22	Edited	Ray KP, Jupe S, Garapati P V
2011-05-22	Authored	Ray KP, Jupe S, Garapati P V
2011-05-29	Reviewed	Abdul-Sater AA, Schindler C, Pinteaux E
2023-05-21	Modified	Wright A

5 submitted entities found in this pathway, mapping to 9 Reactome entities

Input	UniProt Id	Input	UniProt Id	Input	UniProt Id
B2m	P61769	Cd86	P42081	Hck	P08631
Irf9	Q00978	Ptpn18	Q99952		

Input	Ensembl Id
B2m	ENSG00000166710
Irf9	ENSG00000213928

Input	Ensembl Id
Cd86	ENSG00000114013
Ptpn18	ENSG00000072135

3. Interferon gamma signaling (R-HSA-877300)

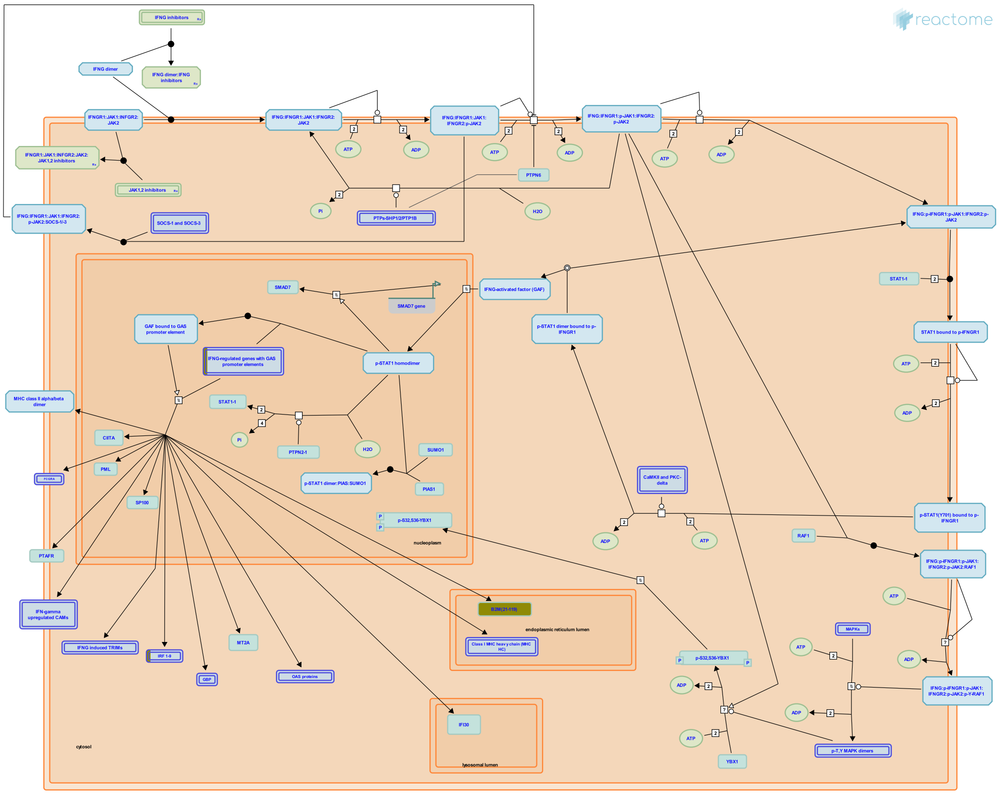

Interferon-gamma (IFN-gamma) belongs to the type II interferon family and is secreted by activated immune cells-primarily T and NK cells, but also B-cells and APC. INFG exerts its effect on cells by interacting with the specific IFN-gamma receptor (IFNGR). IFNGR consists of two chains, namely IFNGR1 (also known as the IFNGR alpha chain) and IFNGR2 (also known as the IFNGR beta chain). IFNGR1 is the ligand binding receptor and is required but not sufficient for signal transduction, whereas IFNGR2 do not bind IFNG independently but mainly plays a role in IFNG signaling and is generally the limiting factor in IFNG responsiveness. Both IFNGR chains lack intrinsic kinase/phosphatase activity and thus rely on other signaling proteins like Janus-activated kinase 1 (JAK1), JAK2 and Signal transducer and activator of transcription 1 (STAT-1) for signal transduction. IFNGR complex in its resting state is a preformed tetramer and upon IFNG association undergoes a conformational change. This conformational change induces the phosphorylation and activation of JAK1, JAK2, and STAT1 which in turn induces genes containing the gamma-interferon activation sequence (GAS) in the promoter.

References

Schroder K, Ravasi T, Hume DA & Hertzog PJ (2004). Interferon-gamma: an overview of signals, mechanisms and functions. J Leukoc Biol, 75, 163-89.

Aguet M, Bach EA & Schreiber RD (1997). The IFN gamma receptor: a paradigm for cytokine receptor signaling. Annu Rev Immunol, 15, 563-91.

Gough DJ, Levy DE, Clarke CJ & Johnstone RW (2008). IFNgamma signaling-does it mean JAK-STAT?. Cytokine Growth Factor Rev, 19, 383-94.

Izotova LS, Garotta G, Muthukumaran G, Kotenko SV, Cook JR & Pestka S (1997). The interferon gamma (IFN-gamma) receptor: a paradigm for the multichain cytokine receptor. Cytokine Growth Factor Rev, 8, 189-206.

Edit history

Date	Action	Author
2010-06-08	Edited	Garapati P V
2010-06-08	Authored	Garapati P V
2010-06-11	Created	Garapati P V
2010-08-17	Reviewed	Abdul-Sater AA, Schindler C
2023-05-30	Modified	Wright A

2 submitted entities found in this pathway, mapping to 4 Reactome entities

Input	UniProt Id
B2m	P61769

Input	UniProt Id
Irf9	Q00978

Input	Ensembl Id
B2m	ENSG00000166710

Input	Ensembl Id
Irf9	ENSG00000213928

4. Amyloid fiber formation (R-HSA-977225)

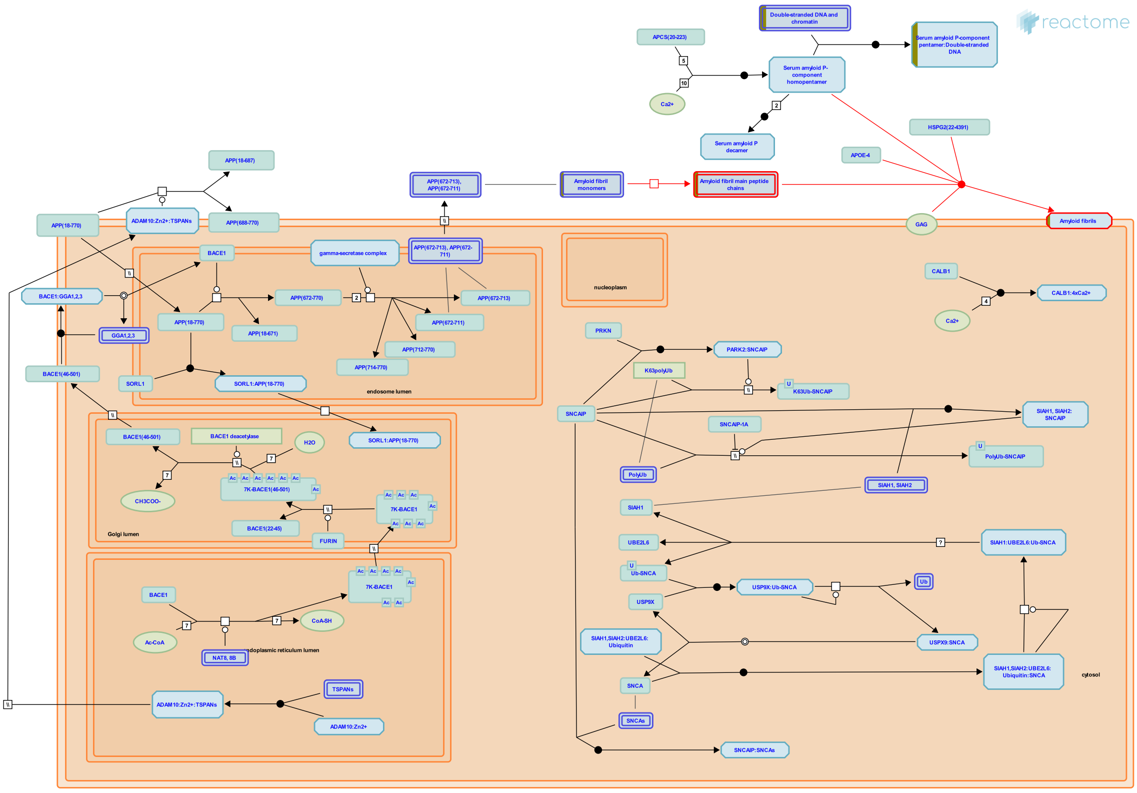

Amyloid is a term used to describe deposits of fibrillar proteins, typically extracellular. The abnormal accumulation of amyloid, amyloidosis, is a term associated with tissue damage caused by amyloid deposition, seen in numerous diseases including neurodegenerative diseases such as Alzheimer's, Parkinson's and Huntington's. Amyloid deposits consist predominantly of amyloid fibrils, rigid, non-branching structures that form ordered assemblies, characteristically with a cross beta-sheet structure where the sheets run parallel to the direction of the fibril (Sawaya et al. 2007). Often the fibril has a left-handed twist (Nelson & Eisenberg 2006). At least 27 human proteins form amyloid fibrils (Sipe et al. 2010). Many of these proteins have non-pathological functions; the trigger that leads to abnormal aggregations differs between proteins and is not well understood but in many cases the peptides are abnormal fragments or mutant forms arising from polymorphisms, suggesting that the initial event may be aggregation of misfolded or unfolded peptides. Early studies of Amyloid-beta assembly led to a widely accepted model that assembly was a nucleation-dependent polymerization reaction (Teplow 1998) but it is now understood to be more complex, with multiple 'off-pathway' events leading to a variety of oligomeric structures in addition to fibrils (Roychaudhuri et al. 2008), though it is unclear whether these intermediate steps are required in vivo. An increasing body of evidence suggests that these oligomeric forms are primarily responsible for the neurotoxic effects of Amyloid-beta (Roychaudhuri et al. 2008), alpha-synuclein (Winner et al. 2011) and tau (Dance & Strobel 2009, Meraz-Rios et al. 2010). Amyloid oligomers are believed to have a common structural motif that is independent of the protein involved and not present in fibrils (Kayed et al. 2003). Conformation dependent, aggregation specific antibodies suggest that there are 3 general classes of amyloid oligomer structures (Glabe 2009) including annular structures which may be responsible for the widely reported membrane permeabilization effect of amyloid oligomers. Toxicity of amyloid oligomers preceeds the appearance of plaques in mouse models (Ferretti et al. 2011).

Fibrils are often associated with other molecules, notably heparan sulfate proteoglycans and Serum Amyloid P-component, which are universally associated and seem to stabilize fibrils, possibly by protecting them from degradation.

References

Westermark P (2005). Aspects on human amyloid forms and their fibril polypeptides. FEBS J, 272, 5942-9.

Edit history

Date	Action	Author
2010-10-15	Authored	Jupe S
2010-10-15	Created	Jupe S
2011-04-08	Edited	Jupe S
2011-04-08	Reviewed	Perry G
2015-11-09	Reviewed	Perry G
2023-03-08	Modified	Matthews L

2 submitted entities found in this pathway, mapping to 3 Reactome entities

Input	UniProt Id	Input	UniProt Id
B2m	P61769	Hist1h2be	P62807, Q93079

5. Immune System (R-HSA-168256)

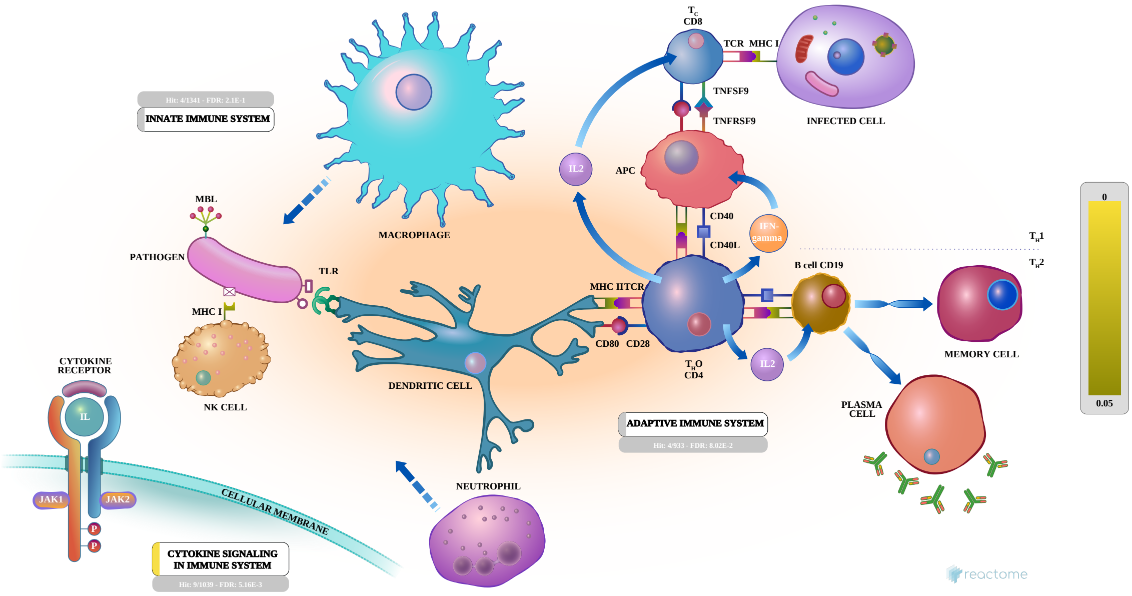

Humans are exposed to millions of potential pathogens daily, through contact, ingestion, and inhalation. Our ability to avoid infection depends on the adaptive immune system and during the first critical hours and days of exposure to a new pathogen, our innate immune system.

References

Edit history

Date	Action	Author
2005-11-12	Created	Gillespie ME
2006-03-30	Authored	Luo F, Ouwehand WH, Gillespie ME, de Bono B
2006-04-19	Reviewed	Zwaginga JJ, D'Eustachio P, Gay NJ, Gale M Jr
2023-05-21	Modified	Wright A

7 submitted entities found in this pathway, mapping to 12 Reactome entities

Input	UniProt Id	Input	UniProt Id	Input	UniProt Id
B2m	P28067, P61769	Cd86	P42081	Clec5a	Q9NY25
Hck	P08631	Irf9	Q00978	Ncf1	P14598
Ptpn18	Q99952				

Input	Ensembl Id	Input	Ensembl Id
B2m	ENSG00000166710	Cd86	ENSG00000114013
Irf9	ENSG00000213928	Ptpn18	ENSG00000072135

6. The role of Nef in HIV-1 replication and disease pathogenesis (R-HSA-164952)

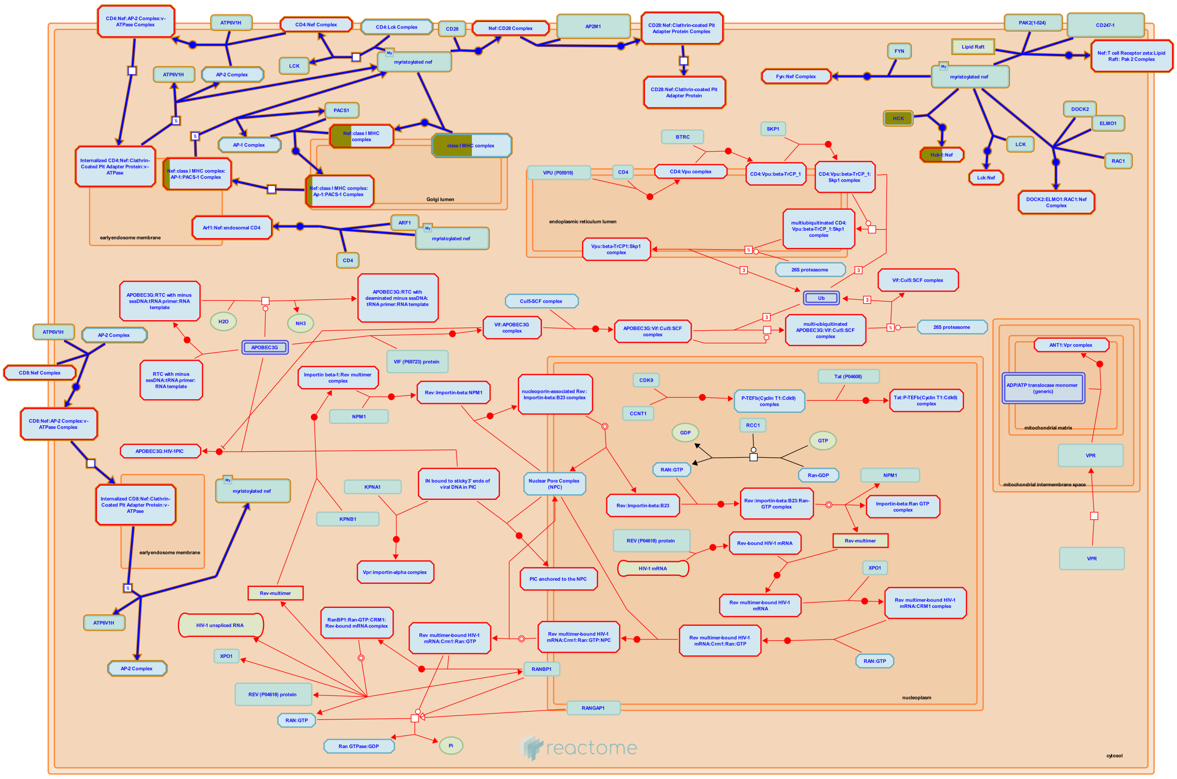

The HIV-1 Nef protein is a 27-kDa myristoylated protein that is abundantly produced during the early phase of viral replication cycle. It is highly conserved in all primate lentiviruses, suggesting that its function is essential for survival of these pathogens. The protein name "Nef" was derived from early reports of its negative effect on viral replication, thus 'negative factor' or Nef. Subsequently it has been demonstrated that Nef plays an important role in several steps of HIV replication. In addition, it appears to be a critical pathogenic factor, as Nef-deficient SIV and HIV are significantly less pathogenic than the wild-type viruses, whereas Nef-transgenic mice show many features characteristic to HIV disease.

The role of Nef in HIV-1 replication and disease pathogenesis is determined by at least four independent activities of this protein. Nef affects the cell surface expression of several cellular proteins, interferes with cellular signal transduction pathways, enhances virion infectivity and viral replication, and regulates cholesterol trafficking in HIV-infected cells.

References

Zhao RY, Bukrinsky M, Pauza CD, Li HS & Li L (2005). Roles of HIV-1 auxiliary proteins in viral pathogenesis and host-pathogen interactions. Cell Res, 15, 923-34.

Edit history

Date	Action	Author
2005-07-23	Created	Gillespie ME
2007-07-25	Authored	Gillespie ME
2007-08-07	Reviewed	Skowronski J
2023-03-08	Modified	Matthews L

2 submitted entities found in this pathway, mapping to 2 Reactome entities

Input	UniProt Id
B2m	P61769

Input	UniProt Id
Hck	P08631

7. Packaging Of Telomere Ends (R-HSA-171306)

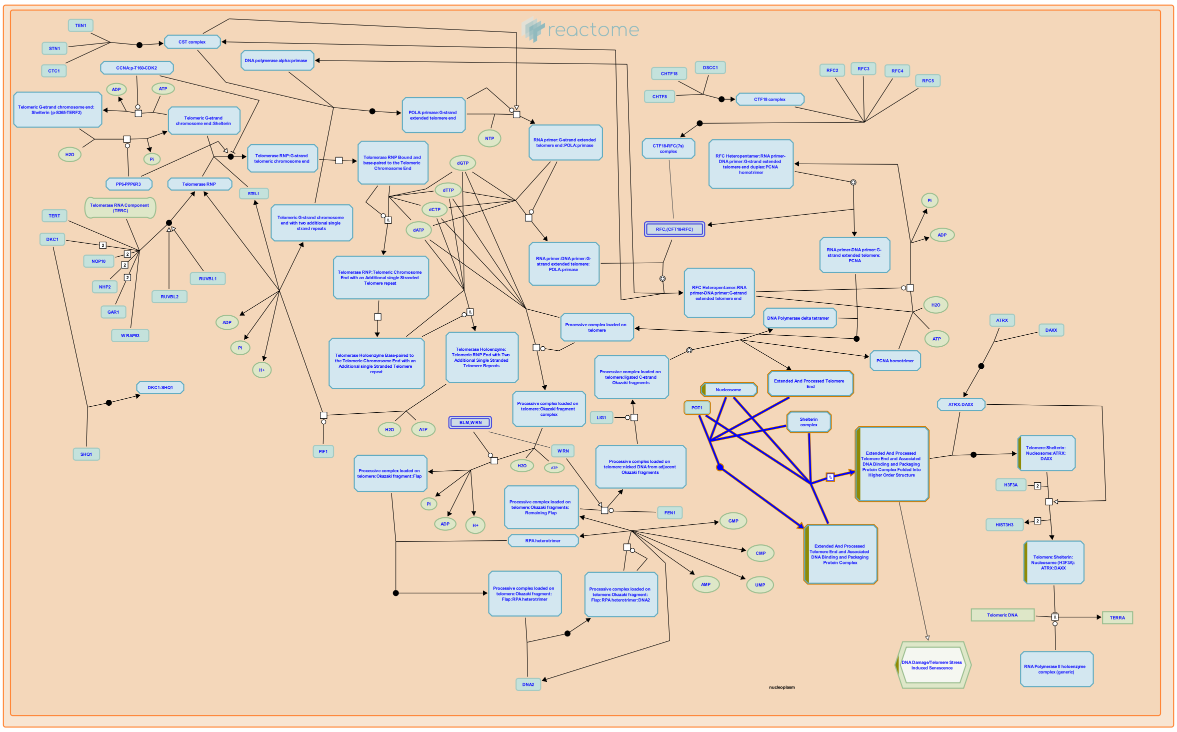

Multiple steps, including C-strand resection, telomerase-mediated elongation, and C-strand synthesis are involved in processing and maintaining the telomere. Though this module posits a linear transit for the steps, in humans it is not well understood how these steps are coordinated and what other events may be involved.

Telomeric DNA can form higher order structures. Electron microscopy of telomeric DNA isolated from human cells provided evidence for lariat-type structures termed telomeric loops, or t-loops (Griffith et al., 1999). t-loops are proposed to result from the invasion of the 3' G-rich single strand overhang into the double stranded telomeric TTAGGG repeat tract. The function of the t-loop is presumed to be the masking of the 3' telomeric overhang. Multiple protein factors can bind telomeric DNA and likely contribute to dynamic, higher order structures.

References

Moss H, Bianchi A, de Lange T, Stansel RM, Rosenfield S, Comeau L & Griffith JD (1999). Mammalian telomeres end in a large duplex loop. Cell, 97, 503-14.

Edit history

Date	Action	Author
2006-02-04	Created	Gillespie ME
2006-03-10	Authored	Seidel J, Blackburn EH
2006-07-13	Reviewed	Price C
2009-06-03	Revised	D'Eustachio P
2019-12-04	Revised	Orlic-Milacic M
2020-04-29	Reviewed	Hayashi MT
2020-05-04	Edited	Orlic-Milacic M

Date	Action	Author
2023-05-21	Modified	Wright A

1 submitted entities found in this pathway, mapping to 2 Reactome entities

Input	UniProt Id
Hist1h2be	P62807, Q93079

8. RNA Polymerase I Promoter Opening (R-HSA-73728)

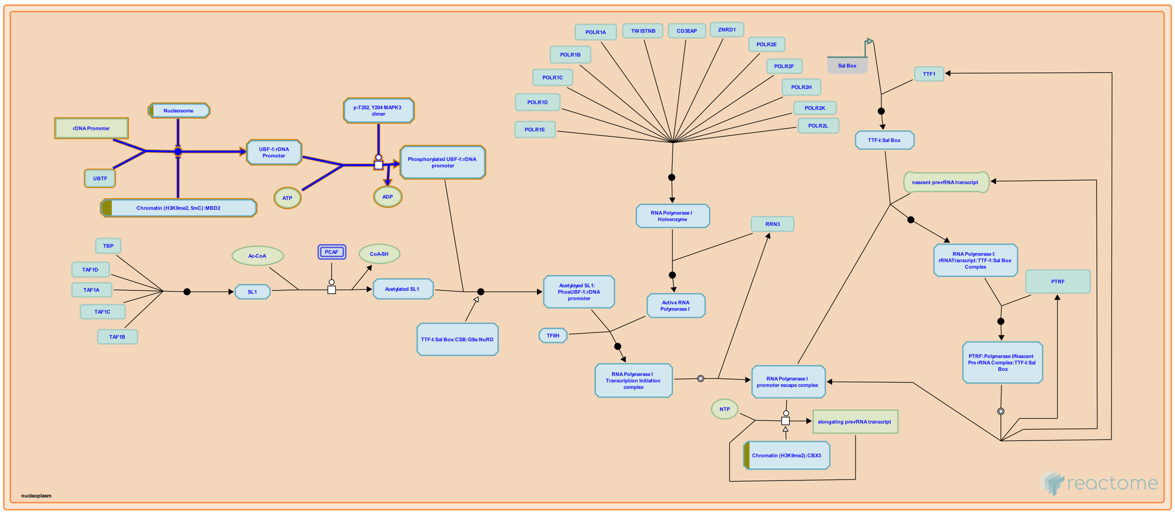

Cellular compartments: nucleolus.

The activity of the upstream binding factor (UBF-1) plays an important role in the regulation of rRNA synthesis. Studies reveal that phosphorylation of UBF-1 is required for its interaction with the RNA polymerase I complex, suggesting that phosphorylation of UBF-1 bound to the rDNA promoter during promoter opening modulates the assembly of the transcription initiation complex.

References

Edit history

Date	Action	Author
2003-07-03	Authored	Comai L
2003-07-03	Created	Comai L
2023-05-19	Edited	Gillespie ME
2023-05-21	Modified	Wright A

1 submitted entities found in this pathway, mapping to 2 Reactome entities

Input	UniProt Id
Hist1h2be	P62807, Q93079

9. DNA methylation (R-HSA-5334118)

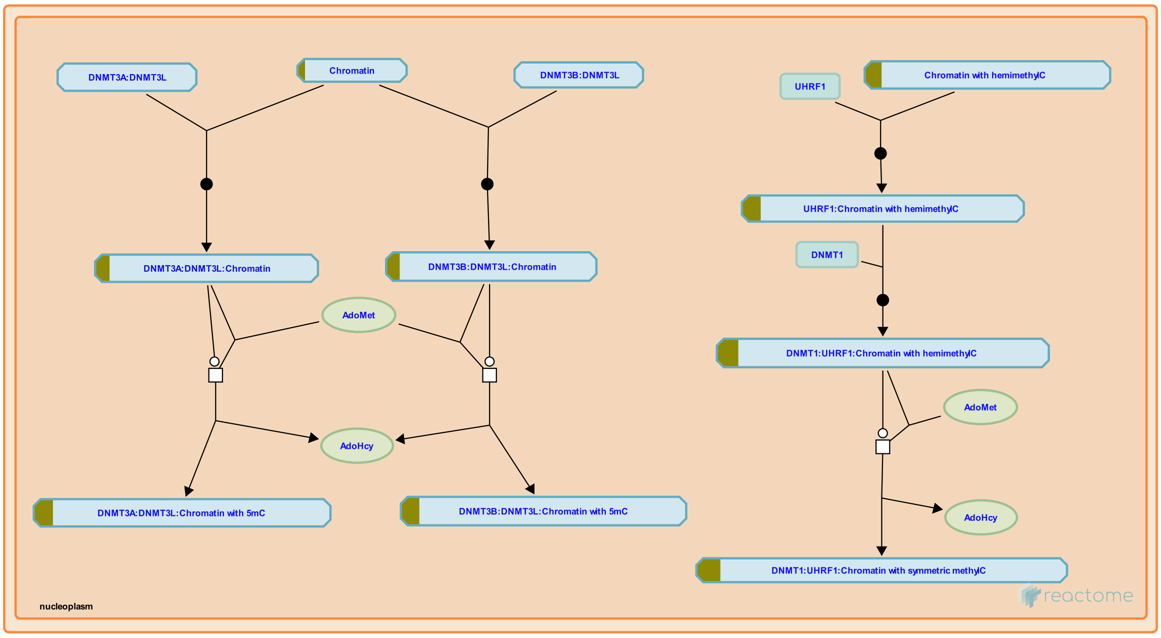

Cellular compartments: nucleoplasm.

Methylation of cytosine is catalyzed by a family of DNA methyltransferases (DNMTs): DNMT1, DNMT3A, and DNMT3B transfer methyl groups from S-adenosylmethionine to cytosine, producing 5-methylcytosine and homocysteine (reviewed in Klose and Bird 2006, Ooi et al. 2009, Jurkowska et al. 2011, Moore et al. 2013). (DNMT2 appears to methylate RNA rather than DNA.) DNMT1, the first enzyme discovered, preferentially methylates hemimethylated CG motifs that are produced by replication (template strand methylated, synthesized strand unmethylated). Thus it maintains existing methylation through cell division. DNMT3A and DNMT3B catalyze de novo methylation at unmethylated sites that include both CG dinucleotides and non-CG motifs.

DNA from adult humans contains about 0.76 to 1.00 mole percent 5-methylcytosine (Ehrlich et al. 1982, reviewed in Klose and Bird 2006, Ooi et al. 2009, Moore et al. 2013). Methylation of DNA occurs at cytosines that are mainly located in CG dinucleotides. CG dinucleotides are unevenly distributed in the genome. Promoter regions tend to have a high CG-content, forming so-called CG-islands (CGIs), while the CG-content in the remaining part of the genome is much lower. CGIs tend to be unmethylated, while the majority of CGs outside CGIs are methylated. Methylation in promoters and first exons tends to repress transcription while methylation in gene bodies (regions of genes downstream of the promoter and first exon) correlates with transcription (reviewed in Ehrlich and Lacey 2013, Kulis et al. 2013). Proteins such as MeCP2 and MBDs specifically bind 5-methylcytosine and may recruit other factors.

Mammalian development has two major episodes of genome-wide demethylation and remethylation (reviewed in Zhou 2012, Guibert and Weber 2013, Hackett and Surani 2013, Dean 2014). In mice about 1 day after fertilization the paternal genome is actively demethylated by TET proteins together with thymine DNA glycosylase and the maternal genome is demethylated by passive dilution during replication, however methylation at imprinted sites is maintained. The genome has its lowest methylation level about 3.5 days post-fertilization. Remethylation occurs by 6.5 days post-fertilization. The second demethylation-remethylation event occurs in primordial germ cells of the developing embryo about 12.5 days post-fertilization. DNMT3A and DNMT3B, together with the non-catalytic DNMT3L, play major roles in the remethylation events (reviewed in Chen and Chan 2014). How the methyltransferases are directed to particular regions of the genome remains an area of active research. The mechanisms at each locus may differ in detail but a connection between histone modifications and DNA methylation has been observed (reviewed in Rose and Klose 2014).

References

Le T, Moore LD & Fan G (2013). DNA methylation and its basic function. Neuropsychopharmacology, 38, 23-38.

McCune RA, Gehrke C, Midgett RM, Kuo KC, Gama-Sosa MA, Huang LH & Ehrlich M (1982). Amount and distribution of 5-methylcytosine in human DNA from different types of tissues of cells. Nucleic Acids Res., 10, 2709-21.

Dean W (2014). DNA methylation and demethylation: A pathway to gametogenesis and development. Mol. Reprod. Dev., 81, 113-25.

Zhou FC (2012). DNA methylation program during development. Front Biol (Beijing), 7, 485-494.

Chen BF & Chan WY (2014). The de novo DNA methyltransferase DNMT3A in development and cancer. Epigenetics, 9.

Edit history

Date	Action	Author
2014-02-21	Edited	May B
2014-02-21	Authored	May B
2014-02-22	Created	May B
2014-07-24	Reviewed	Martín-Subero JI, Beekman R
2023-03-08	Modified	Matthews L

1 submitted entities found in this pathway, mapping to 2 Reactome entities

Input	UniProt Id
Hist1h2be	P62807, Q93079

10. Interleukin-37 signaling (R-HSA-9008059)

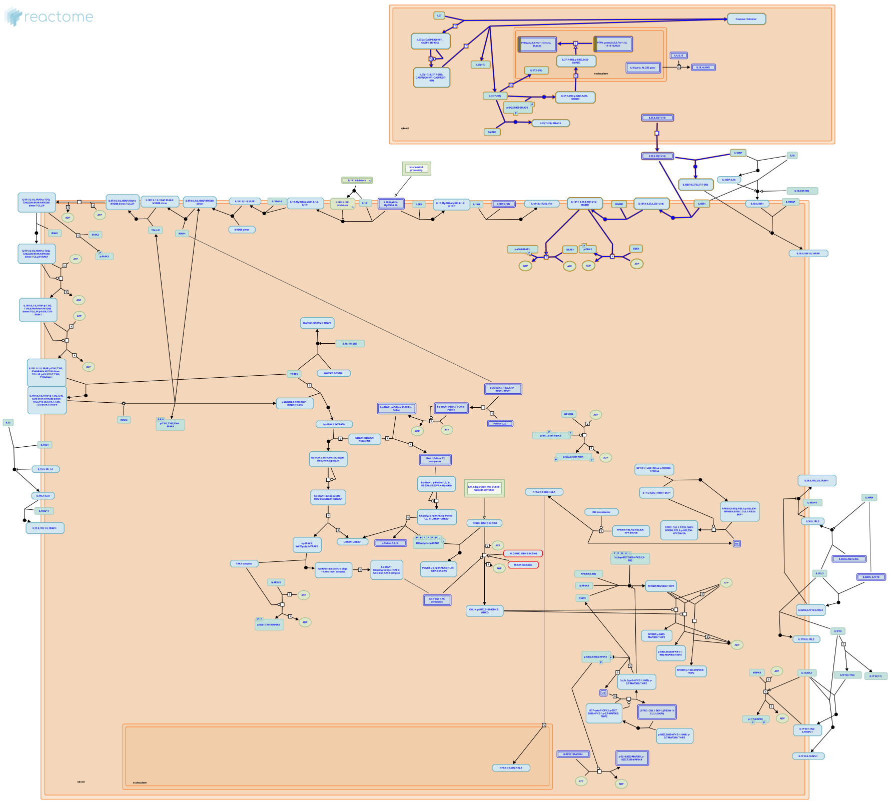

Cellular compartments: plasma membrane, extracellular region, cytosol.

Interleukins (IL) are immunomodulatory proteins that elicit a wide array of responses in cells and tissues. Interleukin 37 (IL37), also known as IL 1F7, is a member of the IL 1 family (Sharma et al. 2008). Isoform b of IL37 (referred just as IL37) is synthesized as a precursor that requires processing (primarily by caspase 1) to attain full receptor agonist or antagonist function (Kumar et al. 2002). Both full length and processed IL37 can bind to the IL 18 binding protein (IL 18BP) and the Interleukin 18 receptor 1 (IL 18R1) (Shi et al. 2003). Upon binding to the IL18R1, IL37 recruits Single Ig IL 1 related receptor (SIGIRR) (Nold-Petry et al. 2015). The IL37:IL18R1 complex can activate phosphorylation of Signal transducer and activator of transcription 3 (STAT3), Tyrosine protein kinase Mer and Phosphatidylinositol 3,4,5 trisphosphate 3 phosphatase and dual specificity protein phosphatase PTEN and can also inhibit Nuclear factor NF kappa B p105 subunit (NFKB) (Nold-Petry et al. 2015). Processed IL37 can be secreted from the cytosol to the extracellular space or translocated into the nucleus (Bulau et al. 2014). Full length IL37 can also be secreted from the cytosol to the extracellular space (Bulau et al. 2014). Processed IL37 can bind with Mothers against decapentaplegic homolog 3 (SMAD3) in the cytosol and then translocate to the nucleus, where it facilitates transcription of Tyrosine protein phosphatase non receptors (PTPNs) (Nold et al. 2010, Luo et al. 2017). These events ultimately lead to suppression of cytokine production in several types of immune cells resulting in reduced inflammation.

References

Dinarello CA, Italiani P, Pfaller T, Pixner C, Nold MF, Lucchesi D, ... Boraschi D (2011). IL-37: a new anti-inflammatory cytokine of the IL-1 family. Eur. Cytokine Netw., 22, 127-47.

Shi H, Wu B, Luo X, Li J, Zhuang X & Jin B (2017). The emerging role of interleukin-37 in cardiovascular diseases. Immun Inflamm Dis.

Edit history

Date	Action	Author
2017-06-07	Created	Varusai TM
2017-08-08	Edited	Varusai TM
2017-08-08	Authored	Varusai TM
2017-11-02	Reviewed	Carriero R, Garlanda C, Mantovani A
2023-05-21	Modified	Wright A

1 submitted entities found in this pathway, mapping to 2 Reactome entities

Input	UniProt Id
Ptpn18	Q99952

Input	Ensembl Id
Ptpn18	ENSG00000072135

11. Recognition and association of DNA glycosylase with site containing an affected purine (R-HSA-110330)

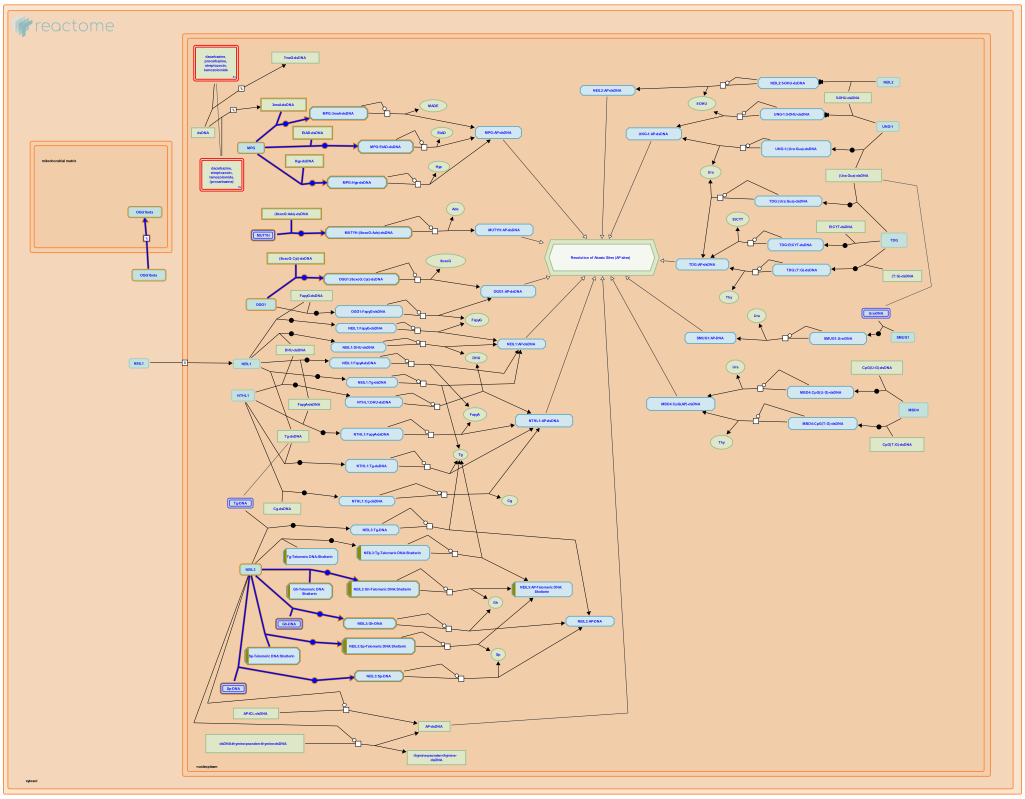

Cellular compartments: nucleoplasm.

The recognition and removal of an altered base by a DNA glycosylase is thought to involve the diffusion of the enzyme along the minor grove of the DNA molecule. The enzyme presumably compresses the backbone of the affected DNA strand at the site of damage. This compression is thought to result in an outward rotation of the damaged residue into a "pocket" of the enzyme that recognizes and cleaves the altered base from the backbone (Slupphaug et al. 1996, Parikh et al. 1998).

References

Kavli B, Tainer JA, Mol CD, Krokan HE, Arvai AS & Slupphaug G (1996). A nucleotide-flipping mechanism from the structure of human uracil-DNA glycosylase bound to DNA. Nature, 384, 87-92.

Tainer JA, Bharati S, Krokan HE, Mol CD, Slupphaug G & Parikh SS (1998). Base excision repair initiation revealed by crystal structures and binding kinetics of human uracil-DNA glycosylase with DNA. EMBO J, 17, 5214-26.

Edit history

Date	Action	Author
2004-01-29	Created	Matthews L
2004-02-03	Edited	Matthews L
2004-02-09	Authored	Matthews L
2014-12-04	Revised	Orlic-Milacic M
2014-12-04	Edited	Orlic-Milacic M

Date	Action	Author
2014-12-22	Reviewed	Borowiec JA
2023-05-21	Modified	Wright A

1 submitted entities found in this pathway, mapping to 2 Reactome entities

Input	UniProt Id
Hist1h2be	P62807, Q93079

12. Assembly of the ORC complex at the origin of replication (R-HSA-68616)

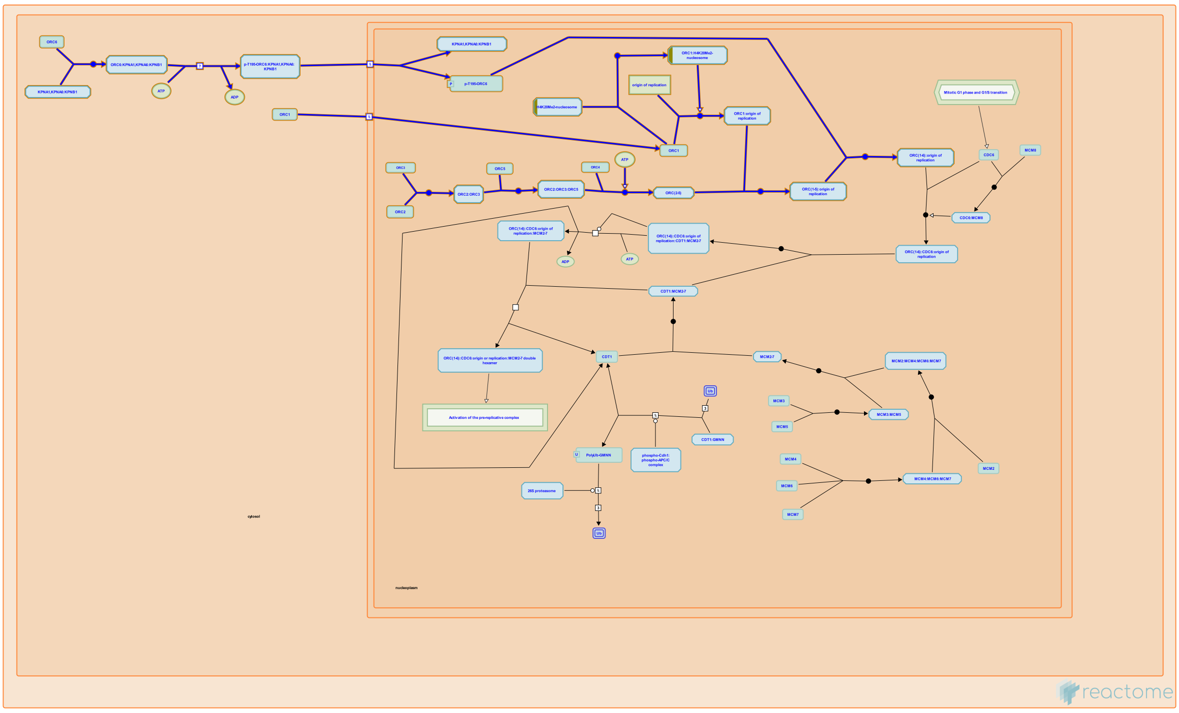

Cellular compartments: nucleoplasm.

Human ORC1 can associate with DNA origin of replication sites independently of other origin of replication complex (ORC) subunits (Hoshina et al. 2013; Eladl et al. 2021). ORC1 localizes to condensed chromosomes during early mitosis (M phase) and serves as a nucleating center for the assembly of the ORC and, subsequently, the pre-replication complex. ORC1 remains associated with late replication origins throughout late G1. Upon S phase entry, ORC1 undergoes ubiquitin-mediated degradation, leading to dissociation of the ORC from chromatin (Kara et al. 2015).

Most human replication origins contain guanine (G)-rich sequences which may form G-quadruplex (G4) structures (Besnard et al. 2012) and these G4 structures may mediate the recognition of replication origins by ORC1 (Hoshina et al. 2013; Eladl et al. 2021). Besides binding to nucleosome-free replication origin DNA, ORC1 interacts with neighboring nucleosomes (Hizume et al. 2013), in particular with nucleosomes containing histone H4 dimethylated at lysine 21 (H4K20me2 mark), which is enriched at replication origins. Binding of ORC1 to H4K20me2 facilitates ORC1 binding to replication origins and ORC chromatin loading (Kuo et al. 2012, Zhang et al. 2015).

ORC1 binding sites are universally associated with transcription start sites (TSSs) of coding and non-coding RNAs. Replication origins associated with moderate to high transcription level TSSs (belonging to coding RNAs) fire in early S phase, while those associated with low transcription level TSSs (belonging to non-coding RNAs) fire throughout the S phase (Dellino et al. 2013).

ORC2 forms a heterodimer with ORC3, which is a prerequisite for the association of ORC5 and, subsequently, ORC4 (Ranjan and Gossen 2006; Siddiqui and Stillman 2007). ORC1 binds to the ORC(2-5) complex in the nucleus to form a stable ORC(1-5) complex (Radichev et al. 2006; Ghosh et al. 2011). ORC1 is necessary for the association of the ORC(2-5) complex to chromatin (Radichev et al. 2006). The ORC(2-5) complex exhibits a tightly autoinhibited conformation, with the winged-helix domain (WHD) of ORC2 completely blocking the central DNA-binding channel. Binding of ORC1 remodels the WHD of ORC2, moving it away from the central channel and partially relieving the autoinhibition (Cheng et al. 2020, Jaremko et al. 2020). ORC6 associates with the ORC(1-5) complex to form the ORC(1-6) complex (Ghosh et al. 2011). The association of ORC6 with the ORC(1-5) complex is weak and it frequently does not co-immunoprecipitate with the other ORC(1-5) subunits. ORC4 is the only ORC(1-5) subunit that was shown to directly bind to ORC6 (Radichev et al. 2006). Some ORC6 mutations reported in Meier-Gorlin syndrome were shown to interfere with ORC6 incorporation into the ORC (Balasov et al. 2015).

References

Yura K, Tominaga A, Kadoma H, Kiyasu N, Kunichika T, Obuse C, ... Teranishi H (2013). Human origin recognition complex binds preferentially to G-quadruplex-preferable RNA and single-stranded DNA. J Biol Chem, 288, 30161-30171.

Araki H, Yagura M & Hizume K (2013). Concerted interaction between origin recognition complex (ORC), nucleosomes and replication origin DNA ensures stable ORC-origin binding. Genes Cells, 18, 764-79.

Balasov M, Akhmetova K & Chesnokov I (2015). Drosophila model of Meier-Gorlin syndrome based on the mutation in a conserved C-Terminal domain of Orc6. Am J Med Genet A, 167, 2533-40.

Siddiqui K & Stillman B (2007). ATP-dependent assembly of the human origin recognition complex. J Biol Chem, 282, 32370-83.

Song J, Gozani O, Zhang W & Sankaran S (2015). A Meier-Gorlin syndrome mutation impairs the ORC1-nucleosome association. ACS Chem Biol, 10, 1176-80.

Edit history

Date	Action	Author
2003-06-05	Authored	Davey MJ, O'Donnell M
2003-06-05	Created	Davey MJ, O'Donnell M
2021-07-30	Revised	Kusic-Tisma J
2021-07-30	Authored	Kusic-Tisma J
2021-08-16	Edited	Orlic-Milacic M
2023-05-21	Modified	Wright A

1 submitted entities found in this pathway, mapping to 2 Reactome entities

Input	UniProt Id
Hist1h2be	P62807, Q93079

13. Interferon Signaling (R-HSA-913531)

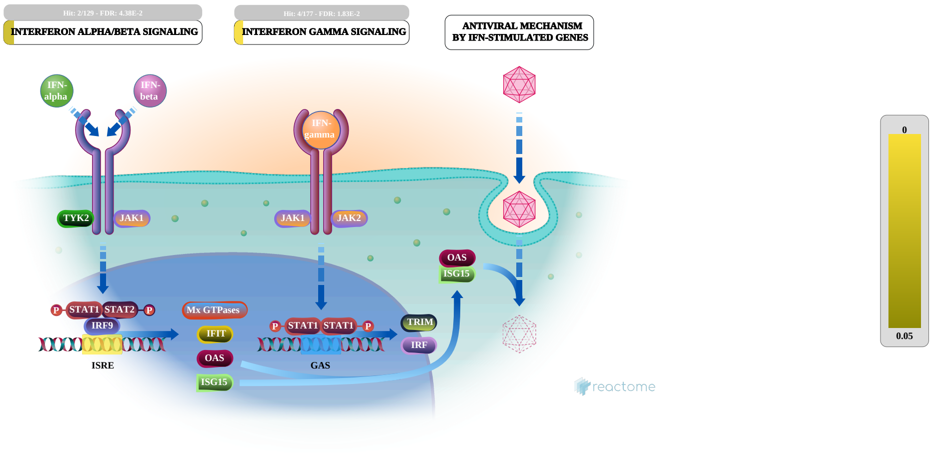

Interferons (IFNs) are cytokines that play a central role in initiating immune responses, especially antiviral and antitumor effects. There are three types of IFNs:Type I (IFN-alpha, -beta and others, such as omega, epsilon, and kappa), Type II (IFN-gamma) and Type III (IFN-lamda). In this module we are mainly focusing on type I IFNs alpha and beta and type II IFN-gamma. Both type I and type II IFNs exert their actions through cognate receptor complexes, IFNAR and IFNGR respectively, present on cell surface membranes. Type I IFNs are broadly expressed heterodimeric receptors composed of the IFNAR1 and IFNAR2 subunits, while the type II IFN receptor consists of IFNGR1 and IFNGR2. Type III interferon lambda has three members: lamda1 (IL-29), lambda2 (IL-28A), and lambda3 (IL-28B) respectively. IFN-lambda signaling is initiated through unique heterodimeric receptor composed of IFN-LR1/IF-28Ralpha and IL10R2 chains.

Type I IFNs typically recruit JAK1 and TYK2 proteins to transduce their signals to STAT1 and 2; in combination with IRF9 (IFN-regulatory factor 9), these proteins form the heterotrimeric complex ISGF3. In nucleus ISGF3 binds to IFN-stimulated response elements (ISRE) to promote gene induction.

Type II IFNs in turn rely upon the activation of JAKs 1 and 2 and STAT1. Once activated, STAT1 dimerizes to form the transcriptional regulator GAF (IFNG activated factor) and this binds to the IFNG activated sequence (GAS) elements and initiate the transcription of IFNG-responsive genes.

Like type I IFNs, IFN-lambda recruits TYK2 and JAK1 kinases and then promote the phosphorylation of STAT1/2, and induce the ISRE3 complex formation.

References

Schroder K, Ravasi T, Hume DA & Hertzog PJ (2004). Interferon-gamma: an overview of signals, mechanisms and functions. J Leukoc Biol, 75, 163-89.

Platanias LC (2005). Mechanisms of type-I- and type-II-interferon-mediated signalling. Nat Rev Immunol, 5, 375-86.

Gough DJ, Levy DE, Clarke CJ & Johnstone RW (2008). IFNgamma signaling-does it mean JAK-STAT?. Cytokine Growth Factor Rev, 19, 383-94.

Ferreira PC, Bonjardim CA & Kroon EG (2009). Interferons: signaling, antiviral and viral evasion. Immunol Lett, 122, 1-11.

Platanias LC & Uddin S (2004). Mechanisms of type-I interferon signal transduction. J Biochem Mol Biol, 37, 635-41.

Edit history

Date	Action	Author
2010-07-07	Edited	Garapati P V
2010-07-07	Authored	Garapati P V
2010-07-16	Created	Garapati P V
2010-08-17	Reviewed	Abdul-Sater AA, Schindler C
2023-05-21	Modified	Wright A

2 submitted entities found in this pathway, mapping to 4 Reactome entities

Input	UniProt Id
B2m	P61769

Input	UniProt Id
Irf9	Q00978

Input	Ensembl Id
B2m	ENSG00000166710

Input	Ensembl Id
Irf9	ENSG00000213928

14. Recognition and association of DNA glycosylase with site containing an affected pyrimidine (R-HSA-110328)

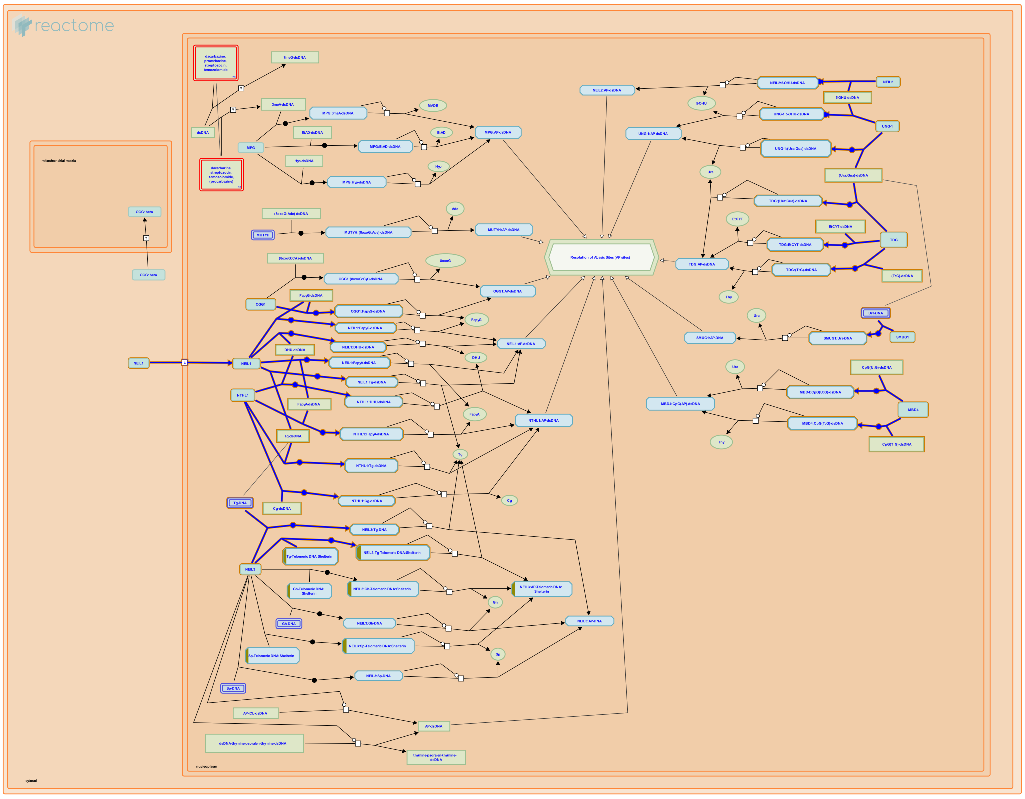

Cellular compartments: nucleoplasm.

Base excision repair is initiated by a DNA glycosylase which first recognizes and removes a damaged or incorrect (e.g. mismatched) base (Sokhansanj et al. 2002).

References

Fitch JP, Sokhansanj BA, Rodrigue GR & Wilson DM 3rd (2002). A quantitative model of human DNA base excision repair. I. Mechanistic insights. Nucleic Acids Res, 30, 1817-25.

Edit history

Date	Action	Author
2004-01-29	Created	Matthews L
2004-02-03	Edited	Matthews L
2004-02-03	Authored	Matthews L
2014-12-04	Revised	Orlic-Milacic M
2014-12-04	Edited	Orlic-Milacic M
2014-12-22	Reviewed	Borowiec JA
2023-05-21	Modified	Wright A

1 submitted entities found in this pathway, mapping to 2 Reactome entities

Input	UniProt Id
Hist1h2be	P62807, Q93079

Input	UniProt Id

15. PRC2 methylates histones and DNA (R-HSA-212300)

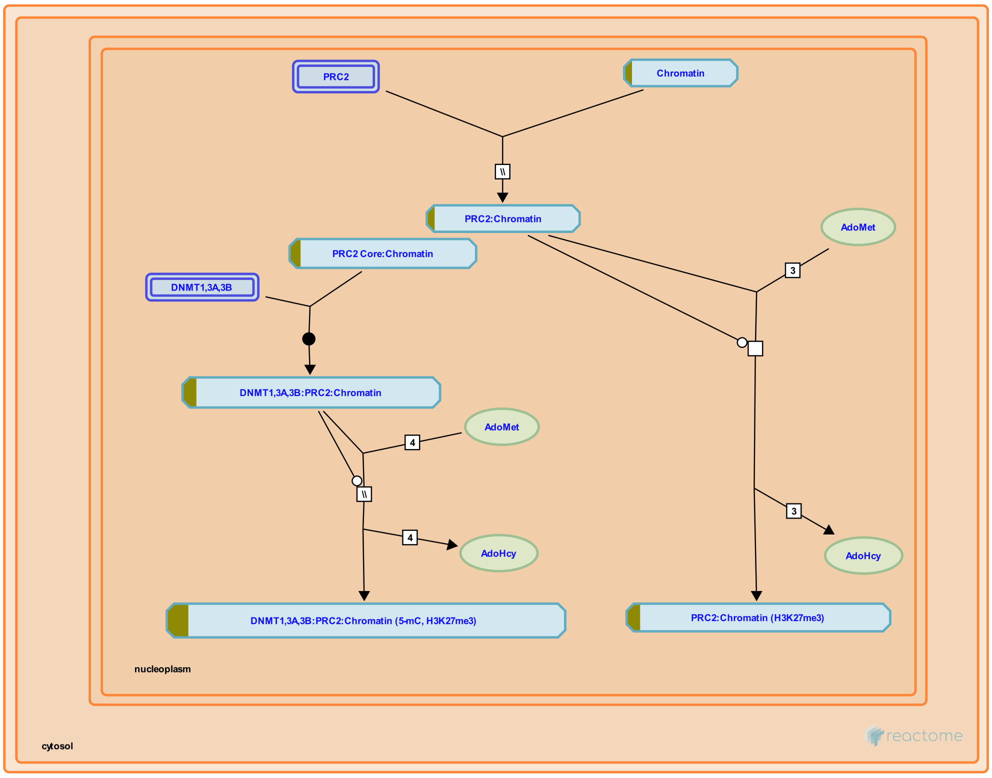

Cellular compartments: nucleoplasm.

Polycomb group proteins are responsible for the heritable repression of genes during development (Lee et al. 2006, Ku et al. 2008, reviewed in Simon and Kingston 2009, Margueron and Reinberg 2011, Di Croce and Helin 2013). Two major families of Polycomb complexes exist: Polycomb Repressive Complex 1 (PRC1) and Polycomb Repressive Complex 2 (PRC2). PRC1 and PRC2 each appear to comprise sets of distinct complexes that contain common core subunits and distinct accessory subunits (reviewed in Nayak et al. 2011). PRC2, through its component EZH2 or, in some complexes, EZH1 produces the initial molecular mark of repression, the trimethylation of lysine-27 of histone H3 (H3K27me3). How PRC2 is initially recruited to a locus remains unknown, however cytosine-guanine (CpG) motifs and transcripts have been suggested. Different mechanisms may be used at different loci. The trimethylated H3K27 produced by PRC2 is bound by the Polycomb subunit of PRC1. PRC1 ubiquitinates histone H2A and maintains repression.

References

Helin K & Di Croce L (2013). Transcriptional regulation by Polycomb group proteins. Nat. Struct. Mol. Biol., 20, 1147-55.

Simon JA & Kingston RE (2013). Occupying chromatin: Polycomb mechanisms for getting to genomic targets, stopping transcriptional traffic, and staying put. Mol. Cell, 49, 808-24.

Min J, Nayak V & Xu C (2011). Composition, recruitment and regulation of the PRC2 complex. Nucleus, 2, 277-82.

Simon JA & Kingston RE (2009). Mechanisms of polycomb gene silencing: knowns and unknowns. Nat Rev Mol Cell Biol, 10, 697-708.

Reinberg D & Margueron R (2011). The Polycomb complex PRC2 and its mark in life. Nature, 469, 343-9.

Edit history

Date	Action	Author
2008-02-09	Authored	May B, Gopinathrao G
2008-02-09	Created	May B, Gopinathrao G
2010-04-06	Edited	May B
2014-02-12	Reviewed	Di Croce L
2023-05-21	Modified	Wright A

1 submitted entities found in this pathway, mapping to 2 Reactome entities

Input	UniProt Id
Hist1h2be	P62807, Q93079

16. SIRT1 negatively regulates rRNA expression (R-HSA-427359)

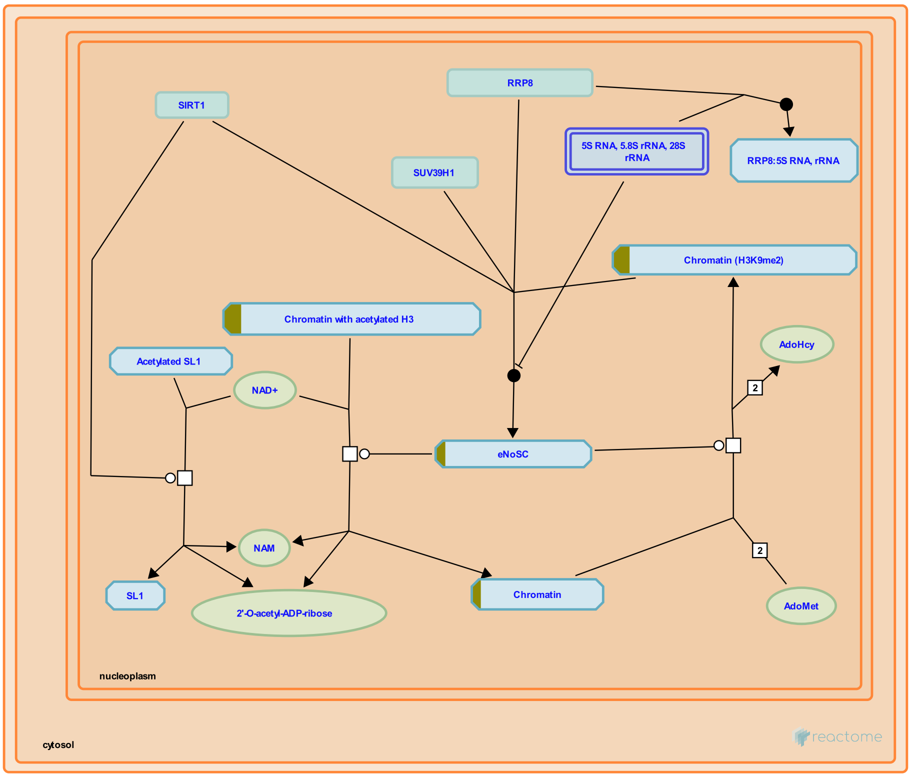

Cellular compartments: nucleoplasm.

Expression of rRNA genes is coupled to the overall metabolism of the cell by the NAD-dependent histone deacetylase SIRT1, a component of the Energy-dependent Nucleolar Silencing Complex (eNoSC) (Murayama et al. 2008, reviewed in Salminen and Kaarniranta 2009, Grummt and Voit 2010). eNoSC comprises Nucleomethylin (NML), SIRT1, and the histone methylase SUV39H1 (Murayama et al. 2008). Deacetylation and methylation of histone H3 in the chromatin of a rRNA gene by eNoSC causes reduced expression of the gene. When glucose is low, NAD is high (NADH is low), activity of SIRT1 is high, and activity of rRNA genes is reduced. It is hypothesized that eNoSC forms on a nucleosome containing dimethylated lysine-9 on histone H3 (H3K9me2) and then eNoSC deacetylates and dimethylates the adjacent nucleosome, thus catalyzing spreading of H3K9me2 throughout the gene.

References

Fujimura A, Murayama A, Minami H, Nagata K, Kuroda T, Kimura K, ... Shimizu T (2008). Epigenetic control of rDNA loci in response to intracellular energy status. Cell, 133, 627-39.

Kaarniranta K & Salminen A (2009). SIRT1 regulates the ribosomal DNA locus: epigenetic candles twinkle longevity in the Christmas tree. Biochem. Biophys. Res. Commun., 378, 6-9.

Voit R & Grummt I (2010). Linking rDNA transcription to the cellular energy supply. Cell Cycle, 9, 225-6.

Edit history

Date	Action	Author
2009-06-20	Created	May B

Date	Action	Author
2009-06-22	Edited	May B
2009-06-22	Authored	May B
2014-01-21	Reviewed	Voit R, Grummt I
2023-05-21	Modified	Wright A

1 submitted entities found in this pathway, mapping to 2 Reactome entities

Input	UniProt Id
Hist1h2be	P62807, Q93079

17. Cleavage of the damaged purine (R-HSA-110331)

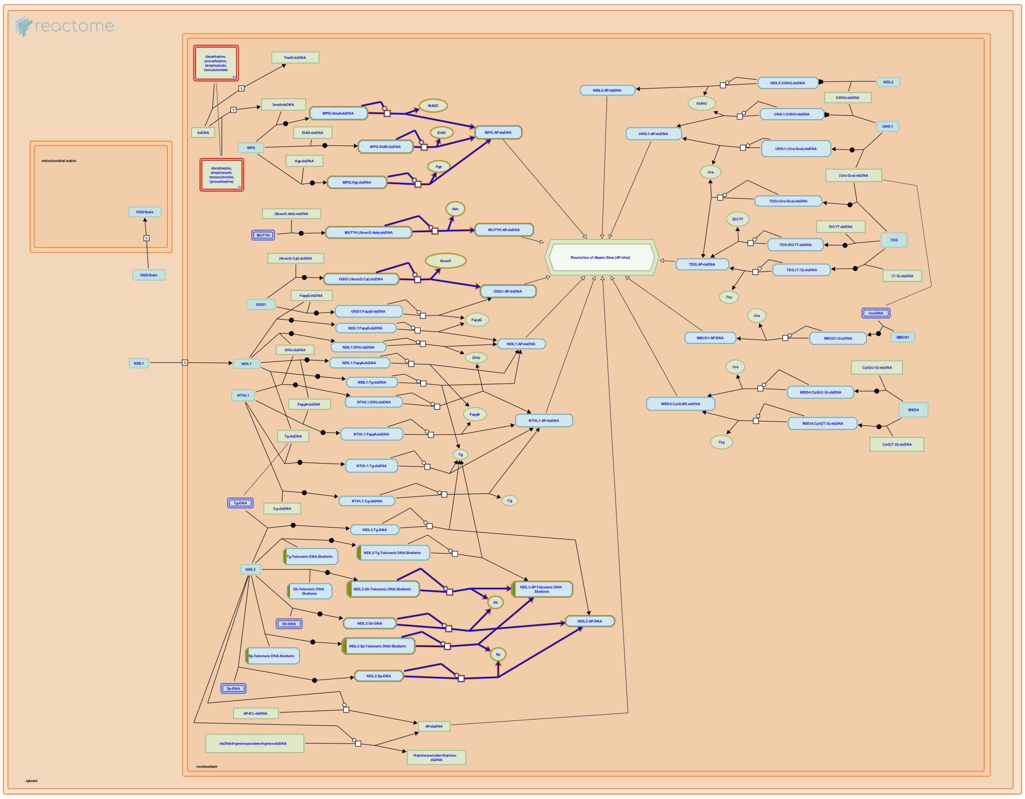

Cellular compartments: nucleoplasm.

Damaged purines are cleaved from the sugar-phosphate backbone by purine-specific glycosylases (Saparbaev and Laval 1994, Lindahl and Wood 1999).

References

Lindahl T & Wood RD (1999). Quality control by DNA repair. Science, 286, 1897-905.

Laval J & Saparbaev M (1994). Excision of hypoxanthine from DNA containing dIMP residues by the Escherichia coli, yeast, rat, and human alkylpurine DNA glycosylases. Proc. Natl. Acad. Sci. U.S.A., 91, 5873-7.

Edit history

Date	Action	Author
2004-01-29	Created	Matthews L
2004-02-03	Edited	Matthews L
2004-02-03	Authored	Matthews L
2014-12-04	Revised	Orlic-Milacic M
2014-12-04	Edited	Orlic-Milacic M
2014-12-22	Reviewed	Borowiec JA
2023-05-21	Modified	Wright A

1 submitted entities found in this pathway, mapping to 2 Reactome entities

Input	UniProt Id
Hist1h2be	P62807, Q93079

18. Depurination (R-HSA-73927)

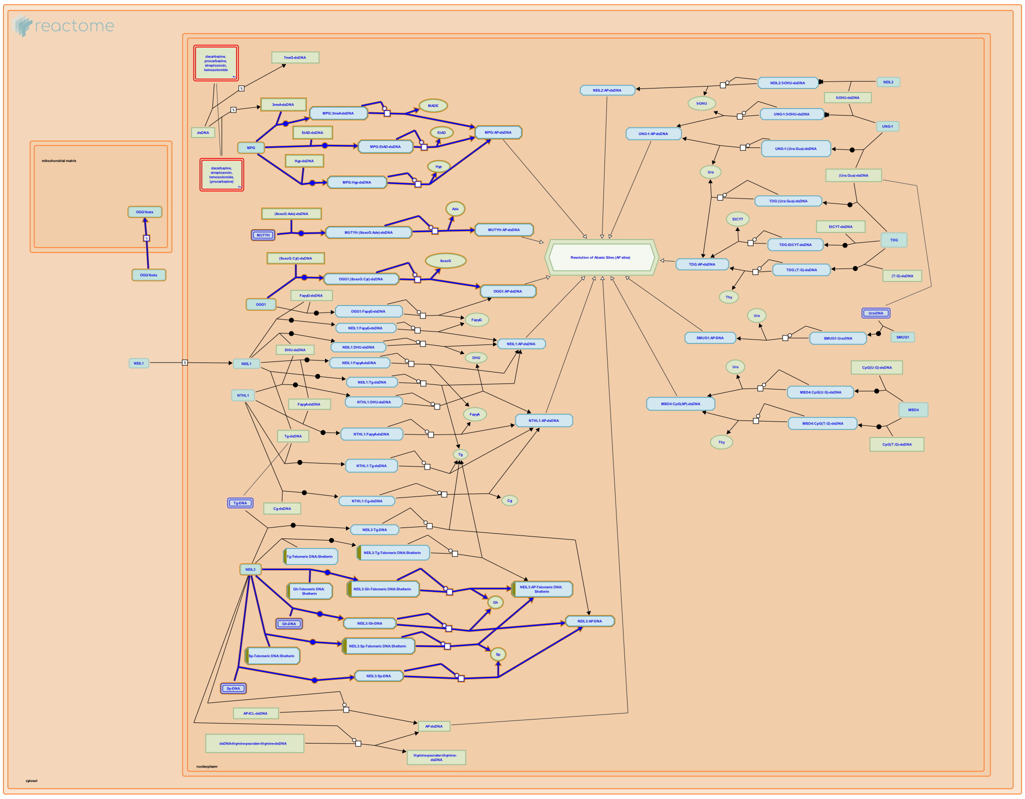

Cellular compartments: nucleoplasm.

Depurination of a damaged nucleotide is mediated by a purine-specific DNA glycosylase. The glycosylase cleaves the N-C1' glycosidic bond between the damaged DNA base and the deoxyribose sugar, generating a free base and an abasic i.e. apurinic/apyrimidinic (AP) site (Slupphaug et al. 1996, Parikh et al. 1998).

References

Kavli B, Tainer JA, Mol CD, Krokan HE, Arvai AS & Slupphaug G (1996). A nucleotide-flipping mechanism from the structure of human uracil-DNA glycosylase bound to DNA. Nature, 384, 87-92.

Tainer JA, Bharati S, Krokan HE, Mol CD, Slupphaug G & Parikh SS (1998). Base excision repair initiation revealed by crystal structures and binding kinetics of human uracil-DNA glycosylase with DNA. EMBO J, 17, 5214-26.

Edit history

Date	Action	Author
2004-02-03	Edited	Matthews L
2004-02-03	Created	Matthews L
2004-02-09	Authored	Matthews L
2014-12-04	Revised	Orlic-Milacic M
2014-12-04	Edited	Orlic-Milacic M
2014-12-22	Reviewed	Borowiec JA
2023-05-21	Modified	Wright A

1 submitted entities found in this pathway, mapping to 2 Reactome entities

Input	UniProt Id
Hist1h2be	P62807, Q93079

19. Inhibition of DNA recombination at telomere (R-HSA-9670095)

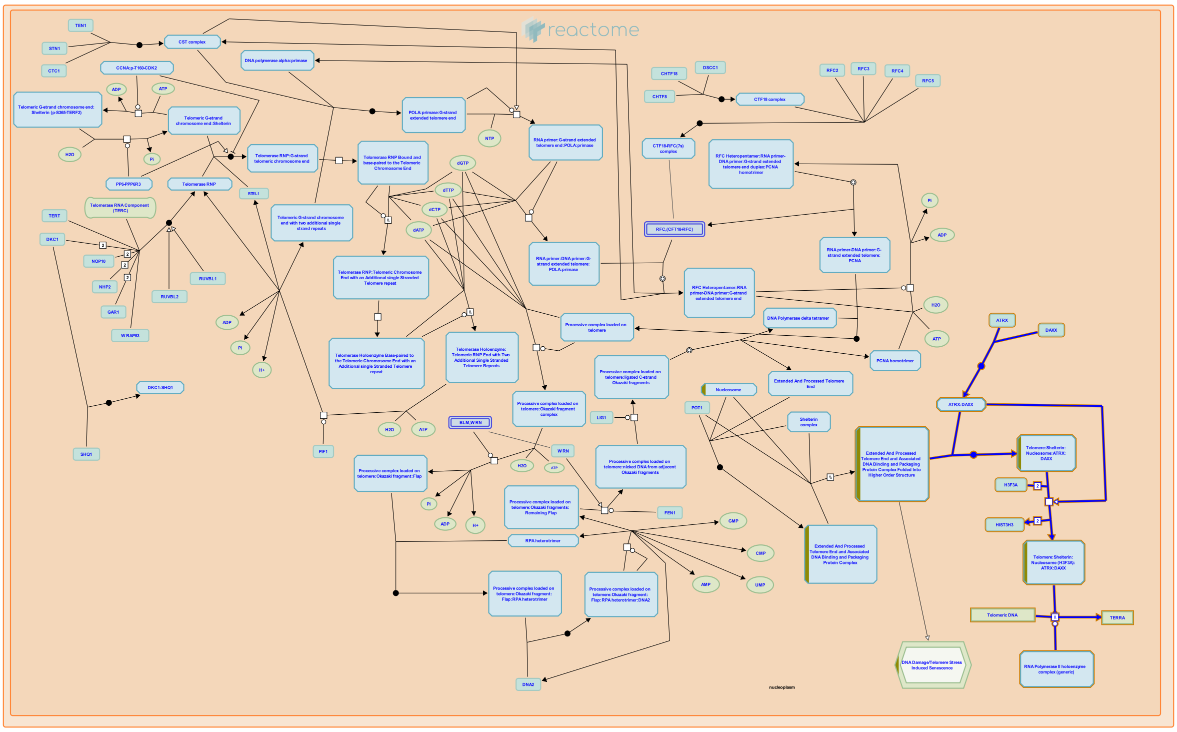

Cellular compartments: nucleoplasm.

Telomeres resemble double strand DNA breaks (DSBs) and, if not properly packaged and protected, are recognized by the DNA double strand break repair (DSBR) machinery. Initiation of DSB signaling at telomeres due to replicative shortening of telomeres is one of the triggers of cellular senescence, which can also be triggered by other cellular stressors, such as oxidative stress, and oncogenic signaling-induced mitotic arrest. The loss of telomere protection can result in telomere fusions via non-homologous end joining (NHEJ) of microhomology-mediated end joining (MMEJ). Loss of telomere protection accompanied by changes in the organization of telomeric chromatin (O'Sullivan et al. 2014) can trigger extension of telomeres via homologous recombination repair-mediated alternative lengthening of telomeres (ALT). ALT occurs in about 5-15% of cancers and is a telomerase-independent mechanism of replicative immortality. For review, please refer to Arnoult and Karlseder 2015 and Pickett and Reddel 2015.

References

Karlseder J, Schreiber SL, O'Sullivan RJ & Kubicek S (2010). Reduced histone biosynthesis and chromatin changes arising from a damage signal at telomeres. Nat. Struct. Mol. Biol., 17, 1218-25.

Karlseder J & Arnoult N (2015). Complex interactions between the DNA-damage response and mammalian telomeres. Nat. Struct. Mol. Biol., 22, 859-66.

Reddel RR & Pickett HA (2015). Molecular mechanisms of activity and derepression of alternative lengthening of telomeres. Nat. Struct. Mol. Biol., 22, 875-80.

Edit history

Date	Action	Author
2019-12-04	Created	Orlic-Milacic M
2019-12-19	Authored	Orlic-Milacic M

Date	Action	Author
2020-04-29	Reviewed	Hayashi MT
2020-05-04	Edited	Orlic-Milacic M
2023-05-21	Modified	Wright A

1 submitted entities found in this pathway, mapping to 2 Reactome entities

Input	UniProt Id
Hist1h2be	P62807, Q93079

20. ERCC6 (CSB) and EHMT2 (G9a) positively regulate rRNA expression (R-HSA-427389)

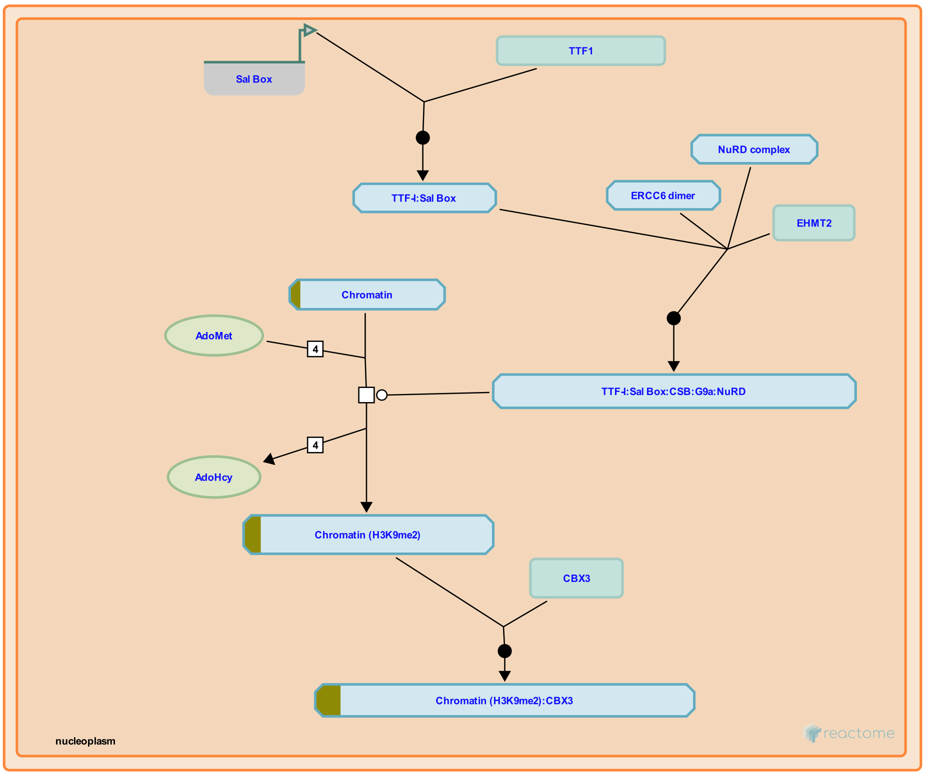

Cellular compartments: nucleoplasm.

About half of the rRNA genes in the genome are actively expressed, being transcribed by RNA polymerase I (reviewed in Nemeth and Langst 2008, Bartova et al. 2010, Goodfellow and Zomerdijk 2012, Grummt and Langst 2013). As inferred from mouse, those genes that are expressed are activated by ERCC6 (also known as Cockayne Syndrome protein, CSB) which interacts with TTF-I bound to the T0 terminator region (also know as the Sal Box) of rRNA genes (Yuan et al. 2007, reviewed in Birch and Zomerdijk 2008, Grummt and Langst 2013). ERCC6 recruits the histone methyltransferase EHMT2 (also known as G9a) which dimethylates histone H3 at lysine-9 in the coding region of rRNA genes. The dimethylated lysine is bound by CBX3 (also known as Heterochromatic Protein-1gamma, HP1gamma) and increases expression of the rRNA gene. Continuing dimethylation depends on continuing transcription. Mutations in CSB result in dysregulation of RNA polymerase I transcription, which plays a role in the symptoms of Cockayne Syndrome (reviewed in Hannan et al. 2013).

References

Uhlírová R, Raska I, Kozubek S, Galiová G, Orlova D, Bártová E & Horáková AH (2010). Structure and epigenetics of nucleoli in comparison with non-nucleolar compartments. J. Histochem. Cytochem., 58, 391-403.

Zhou Y, Grummt I, Feng W, Yuan X & Imhof A (2007). Activation of RNA polymerase I transcription by cockayne syndrome group B protein and histone methyltransferase G9a. Mol Cell, 27, 585-95.

Zomerdijk JC & Goodfellow SJ (2012). Basic mechanisms in RNA polymerase I transcription of the ribosomal RNA genes. Subcell. Biochem., 61, 211-36.

Rothblum LI, Pearson RB, Sanij E, Hannan RD & Hannan KM (2013). Dysregulation of RNA polymerase I transcription during disease. Biochim. Biophys. Acta, 1829, 342-60.

Längst G & Grummt I (2013). Epigenetic control of RNA polymerase I transcription in mammalian cells. Biochim. Biophys. Acta, 1829, 393-404.

Edit history

Date	Action	Author
2009-06-20	Created	May B
2009-06-21	Edited	May B
2009-06-21	Authored	May B
2016-02-12	Reviewed	Iben S
2023-03-08	Modified	Matthews L

1 submitted entities found in this pathway, mapping to 2 Reactome entities

Input	UniProt Id
Hist1h2be	P62807, Q93079

21. Activated PKN1 stimulates transcription of AR (androgen receptor) regulated genes KLK2 and KLK3 (R-HSA-5625886)

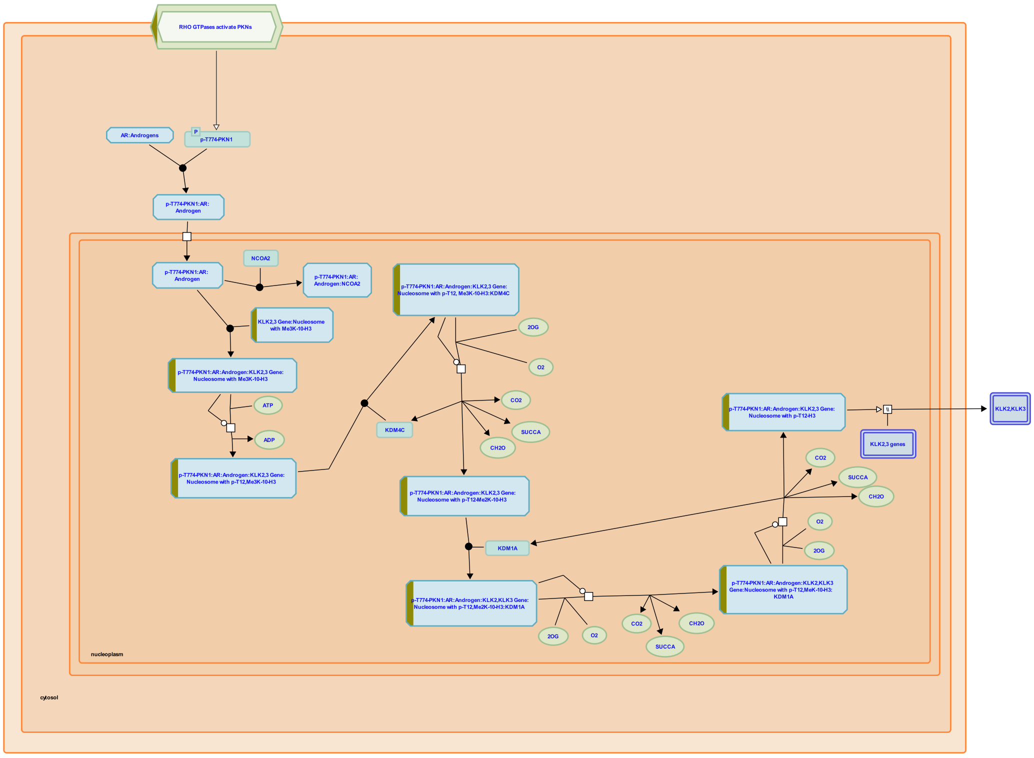

Cellular compartments: nucleoplasm, cytosol.

PKN1, activated by phosphorylation at threonine T774, binds activated AR (androgen receptor) and promotes transcription from AR-regulated promoters. On one hand, phosphorylated PKN1 promotes the formation of a functional complex of AR with the transcriptional coactivator NCOA2 (TIF2) (Metzger et al. 2003). On the other hand, binding of phosphorylated PKN1, in complex with the activated AR, to androgen-reponsive promoters of KLK2 and KLK3 (PSA) genes, leads to PKN1-mediated histone phosphorylation. PKN1-phosphorylated histones recruit histone demethylases KDM4C (JMJD2C) and KDM1A (LSD1), and the ensuing demethylation of histones associated with the promoter regions of KLK2 and KLK3 genes increases their transcription (Metzger et al. 2005, Metzger et al. 2008).

References

Kunowska N, Patnaik D, Schüle R, Metzger E, Wissmann M, Potier N, ... Yin N (2008). Phosphorylation of histone H3 at threonine 11 establishes a novel chromatin mark for transcriptional regulation. Nat. Cell Biol., 10, 53-60.

Peters AH, Schüle R, Metzger E, Müller JM, Wissmann M, Schneider R, ... Yin N (2005). LSD1 demethylates repressive histone marks to promote androgen-receptor-dependent transcription. Nature, 437, 436-9.

Schüle R, Metzger E, Müller JM, Ferrari S & Buettner R (2003). A novel inducible transactivation domain in the androgen receptor: implications for PRK in prostate cancer. EMBO J., 22, 270-80.

Edit history

Date	Action	Author
2014-10-08	Created	Orlic-Milacic M
2014-10-24	Authored	Orlic-Milacic M
2014-12-26	Authored	Rivero Crespo F
2015-02-02	Edited	Orlic-Milacic M
2023-05-21	Modified	Wright A

1 submitted entities found in this pathway, mapping to 2 Reactome entities

Input	UniProt Id
Hist1h2be	P62807, Q93079

22. Cleavage of the damaged pyrimidine (R-HSA-110329)

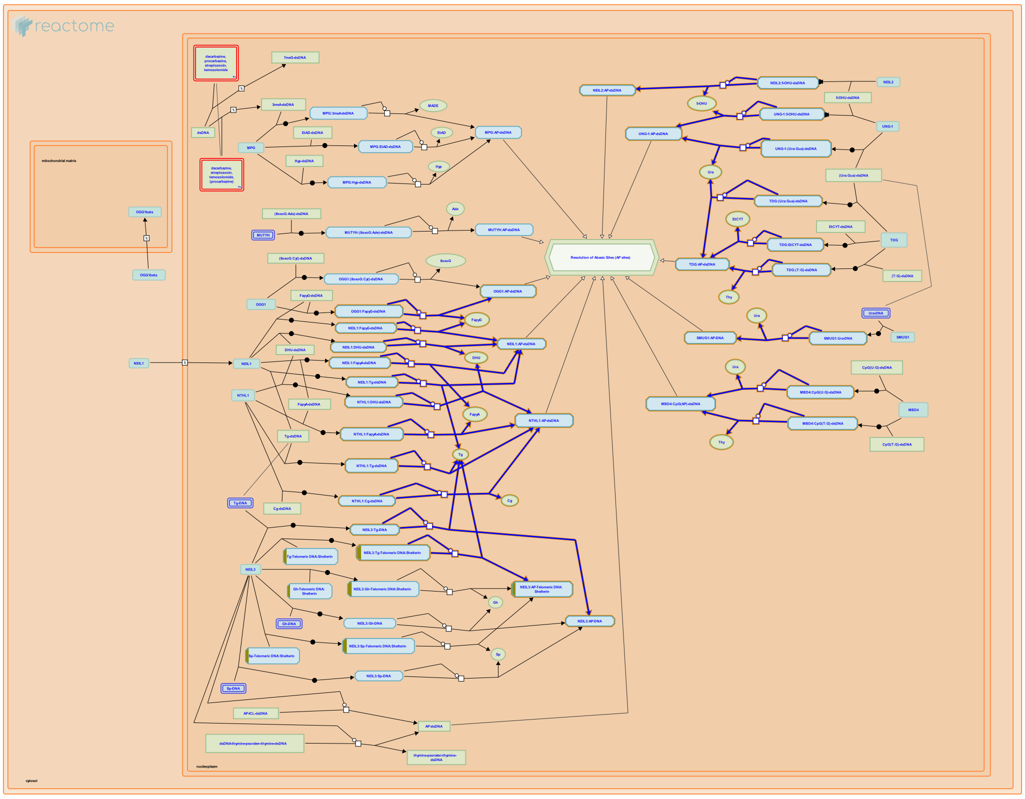

Cellular compartments: nucleoplasm.

Damaged pyrimidines are cleaved by pyrimide-specific glycosylases (Lindahl and Wood 1999).

References

Lindahl T & Wood RD (1999). Quality control by DNA repair. Science, 286, 1897-905.

Edit history

Date	Action	Author
2004-01-29	Created	Matthews L
2004-02-03	Edited	Matthews L
2004-02-03	Authored	Matthews L
2014-12-04	Revised	Orlic-Milacic M
2014-12-04	Edited	Orlic-Milacic M
2014-12-22	Reviewed	Borowiec JA
2023-05-21	Modified	Wright A

1 submitted entities found in this pathway, mapping to 2 Reactome entities

Input	UniProt Id
Hist1h2be	P62807, Q93079

23. Depyrimidination (R-HSA-73928)

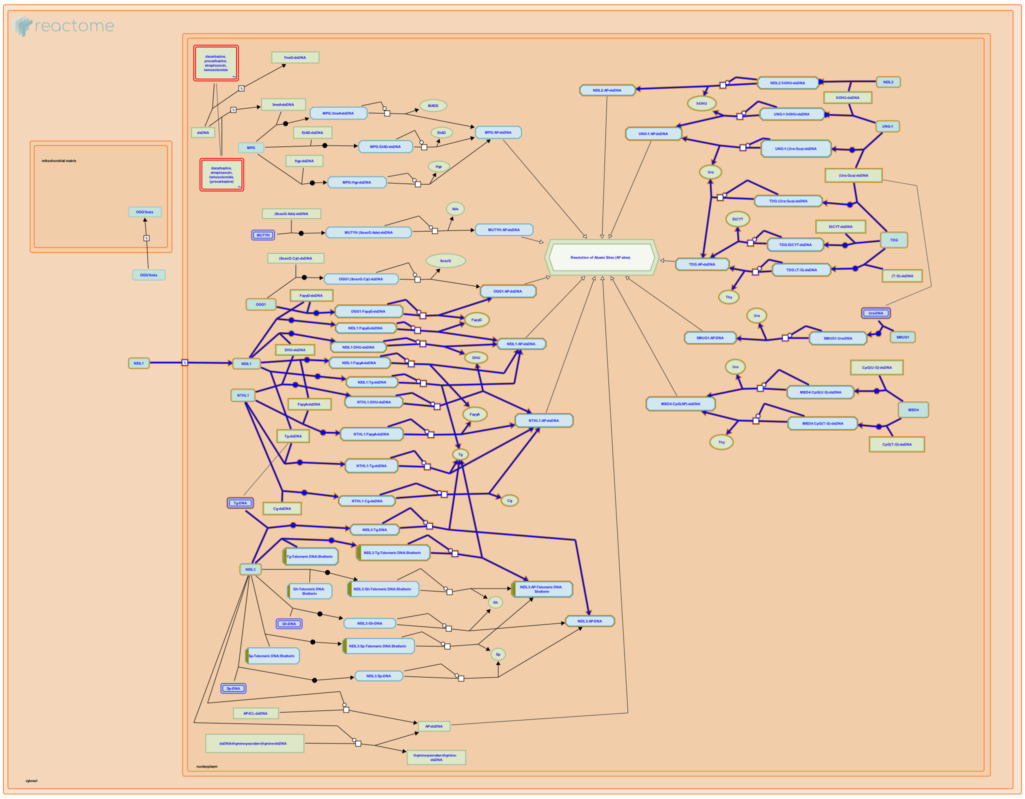

Cellular compartments: nucleoplasm.

Depyrimidination of a damaged nucleotide in DNA is mediated by a pyrimidine-specific DNA glycosylase. The glycosylase cleaves the N-C1' glycosidic bond between the damaged DNA base and the deoxyribose sugar generating a free base and an abasic i.e. apurinic/apyrimidinic (AP) site (Lindahl and Wood 1999).

References

Lindahl T & Wood RD (1999). Quality control by DNA repair. Science, 286, 1897-905.

Edit history

Date	Action	Author
2004-02-03	Edited	Matthews L
2004-02-03	Created	Matthews L
2004-02-09	Authored	Matthews L
2014-12-04	Revised	Orlic-Milacic M
2014-12-04	Edited	Orlic-Milacic M
2014-12-22	Reviewed	Borowiec JA
2023-05-21	Modified	Wright A

1 submitted entities found in this pathway, mapping to 2 Reactome entities

Input	UniProt Id
Hist1h2be	P62807, Q93079

Input	UniProt Id

24. Defective pyroptosis (R-HSA-9710421)

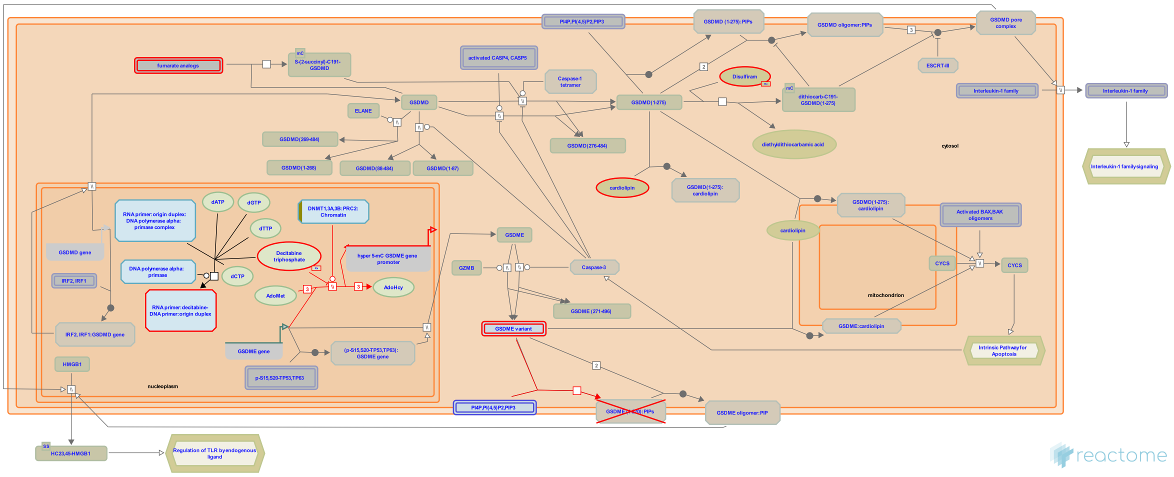

Diseases: gastric adenocarcinoma, breast carcinoma, lung adenocarcinoma, colon adenocarcinoma, melanoma, cancer, head and neck squamous cell carcinoma.

Pyroptosis is a form of lytic inflammatory programmed cell death that is mediated by the pore-forming gasdermins (GSDMs) (Shi J et al. 2017) to stimulate immune responses through the release of pro-inflammatory interleukin (IL)-1[illegible], IL-18 (mainly in GSDMD-mediated pyroptosis) as well as danger signals such as adenosine triphosphate (ATP) or high mobility group protein B1 (HMGB1) (reviewed in Shi J et al. 2017; Man SM et al. 2017; Tang D et al. 2019; Lieberman J et al. 2019). Pyroptosis protects the host from microbial infection but can also lead to pathological inflammation if overactivated or dysregulated (reviewed in Orning P et al. 2019; Tang L et al. 2020). During infections, the excessive production of cytokines can lead to a cytokine storm, which is associated with acute respiratory distress syndrome (ARDS) and systemic inflammatory response syndrome (SIRS) (reviewed in Tisoncik JR et al. 2012; Karki R et al. 2020; Ragab D et al. 2020). Pyroptosis has a close but complicated relationship to tumorigenesis, affected by tissue type and genetic background. Pyroptosis can trigger potent antitumor immune responses or serve as an effector mechanism in antitumor immunity (Wang Q et al. 2020; Zhou Z et al. 2020; Zhang Z et al. 2020), while in other cases, as a type of proinflammatory death, pyroptosis can contribute to the formation of a microenvironment suitable for tumor cell growth (reviewed in Xia X et al. 2019; Jiang M et al. 2020; Zhang Z et al. 2021).

This Reactome module describes the defective GSDME function caused by cancer-related GSDME mutations (Zhang Z et al. 2020). It also shows epigenetic inactivation of GSDME due to hypermethylation of the GSDME promoter region (Akino K et al. 2007; Kim MS et al. 2008a,b; Croes L et al. 2017, 2018; Ibrahim J et al. 2019). Aberrant promoter methylation is considered to be a hallmark of cancer (Ehrlich M et al. 2002; Dong Y et al. 2014; Lam K et al. 2016; Croes L et al. 2018). Treatment with the DNA methyltransferase inhibitor decitabine (5-aza-2'-deoxycytidine or DAC) may elevate GSDME expression in certain cancer cells (Akino K et al. 2007; Fujikane T et al. 2009; Wang Y et al. 2017).

References

Mok TMY, Sengupta S, Li S, Meza-Sosa KF, Junqueira C, Zhang Y, ... Xia S (2020). Gasdermin E suppresses tumour growth by activating anti-tumour immunity. Nature, 579, 415-420.

Suls A, Croes L, Van Camp G, Fransen E, Vanden Berghe W, Beyens M, ... Op de Beeck K (2019). Methylation analysis of Gasdermin E shows great promise as a biomarker for colorectal cancer. Cancer Med, 8, 2133-2145.

Zheng G, Tang L, Burgering BM & Lu C (2020). Emerging insights on the role of gasdermins in infection and inflammatory diseases. Clin Transl Immunology, 9, e1186.

Edit history

Date	Action	Author
2020-11-09	Authored	Shamovsky V
2020-12-30	Created	Shamovsky V
2021-02-17	Edited	Shorser S
2021-02-17	Reviewed	Kanneganti TD, D'Eustachio P, Zhang Z
2021-04-22	Reviewed	Shao F
2023-03-08	Modified	Matthews L

1 submitted entities found in this pathway, mapping to 2 Reactome entities

Input	UniProt Id
Hist1h2be	P62807, Q93079

25. DAP12 interactions (R-HSA-2172127)

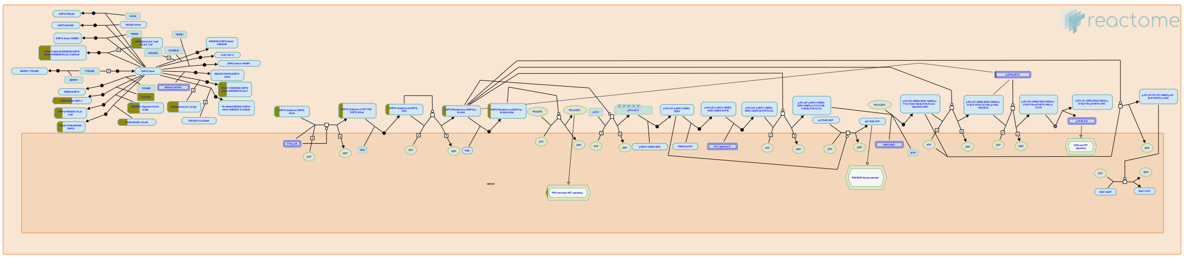

DNAX activation protein of 12kDa (DAP12) is an immunoreceptor tyrosine-based activation motif (ITAM)-bearing adapter molecule that transduces activating signals in natural killer (NK) and myeloid cells. It mediates signalling for multiple cell-surface receptors expressed by these cells, associating with receptor chains through complementary charged transmembrane amino acids that form a salt-bridge in the context of the hydrophobic lipid bilayer (Lanier et al. 1998). DAP12 homodimers associate with a variety of receptors expressed by macrophages, monocytes and myeloid cells including TREM2, Siglec H and SIRP-beta, as well as activating KIR, LY49 and the NKG2C proteins expressed by NK cells. DAP12 is expressed at the cell surface, with most of the protein lying on the cytoplasmic side of the membrane (Turnbull & Colonna 2007, Tessarz & Cerwenka 2008).

References

Lanier LL, Phillips JH, Corliss BC, Leong C & Wu J (1998). Immunoreceptor DAP12 bearing a tyrosine-based activation motif is involved in activating NK cells. Nature, 391, 703-7.

Vivier E & Tomasello E (2005). KARAP/DAP12/TYROBP: three names and a multiplicity of biological functions. Eur J Immunol, 35, 1670-7.

Colonna M & Turnbull IR (2007). Activating and inhibitory functions of DAP12. Nat. Rev. Immunol., 7, 155-61.

Lanier LL (2009). DAP10- and DAP12-associated receptors in innate immunity. Immunol. Rev., 227, 150-60.

Tessarz AS & Cerwenka A (2008). The TREM-1/DAP12 pathway. Immunol Lett, 116, 111-6.

Edit history

Date	Action	Author
2012-03-28	Created	Garapati P V
2012-05-25	Edited	Garapati P V
2012-05-25	Authored	Garapati P V
2012-08-09	Reviewed	Lanier LL
2023-05-21	Modified	Wright A

2 submitted entities found in this pathway, mapping to 2 Reactome entities

Input	UniProt Id	Input	UniProt Id
B2m	P61769	Clec5a	Q9NY25

6. Identifiers found

Below is a list of the input identifiers that have been found or mapped to an equivalent element in Reactome, classified by resource.

14 of the submitted entities were found, mapping to 23 Reactome entities

Input	UniProt Id	Input	UniProt Id	Input	UniProt Id
Adamtsl4	Q6UY14	B2m	P61769	Cd86	P42081
Cebpa	P49715	Clec5a	Q9NY25	Hck	P08631
Hist1h2be	P62807, Q93079	Irf9	Q00978	Ncf1	P14598
Pfkfb3	Q16875	Ptpn18	Q99952	Rhoh	Q15669
Sgk1	O00141	St8sia6	P61647		

Input	Ensembl Id	Input	Ensembl Id	Input	Ensembl Id
B2m	ENSG00000166710	Cd86	ENSG00000114013	Cebpa	ENSG00000245848, ENST00000498907
Irf9	ENSG00000213928	Ptpn18	ENSG00000072135	Sgk1	ENSG00000118515

7. Identifiers not found

These 4 identifiers were not found neither mapped to any entity in Reactome.

Arrdc2	Cep126	Gm20743	Synpo2
