# Supplementary material for: Sex- and region-specific cortical and hippocampal whole genome transcriptome profiles from control and APP/PS1 Alzheimer’s disease mice
Source: PLoS One. 2024 Feb 7;19(2):e0296959. doi: 10.1371/journal.pone.0296959 (PMC10849391; doi:10.1371/journal.pone.0296959)
Supplement: S1 File — S1 Fig: Genotyping of APP/PS1 AD mice and WT control animals. S2 Fig: 3D image of the murine brain including the RS cortex and hippocampus (BROIs) used for transcriptome analysis in our study. S3 Fig: PCA of transcriptomes from the RS cortex and hippocampus of WT controls and APP/PS1 AD mice of both sexes. S4 Fig: Hierarchical clustering of transcriptome data from the RS cortex and hippocampus of WT control and APP/PS1 AD mice of both sexes. S5 Fig: Bar diagrams of the top 30 candidates of DEGs with highest significant FCs (FC > 1.5 and FC < -1.5, p < 0.05). S6 Fig: Pathway analysis of intersectional and signature gene sets in APP/PS1 subgroups. S7 Fig: Comparative qPCR analysis of selected gene transcript levels from the hippocampus of female and male APP/PS1 AD with 5XFAD mice. S1 Table: PCR reaction set-up using PCR Mastermix and genomic DNA. S2 Table: Materials used for one-color microarray-based gene expression data collection. S3 Table: Software used for one-color microarray-based gene expression data collection. S4 Table: Details on genes, forward and reverse primer sequences and annealing temperatures relevant for qPCR experimentation. S5 Table: Characteristics of DEGs in the RS cortex of female APP/PS1 AD mice. S6 Table: Characteristics of DEGs in the hippocampus of female APP/PS1 AD mice. S7 Table: Characteristics of DEGs in the RS cortex of male APP/PS1 AD mice. S8 Table: Characteristics of DEGs in the hippocampus of male APP/PS1 AD mice. S9 Table: Venn analysis of DEGs in the RS cortex and hippocampus of female APP/PS1 AD mice. S10 Table: Venn analysis of DEGs genes in the RS cortex and hippocampus of male APP/PS1 AD mice. S11 Table: Venn analysis of DEGs in the RS cortex of male and female APP/PS1 AD mice. S12 Table: Venn analysis of DEGs in the hippocampus of male and female APP/PS1 AD mice. S13 Table: Differentially regulated l(i)ncRNAs in APP/PS1 AD vs. WT mice. S14 Table: qPCR-based FC analysis of selected genes in the hippocampus of APP/PS1 AD vs. [file pone.0296959.s001.zip › Supplementary Files_R1/Supplementary Figure 6_Pathways_upreg genes/Signature genes up_DEGs_male_Rs Cx_APPPS1/Pathway analysis report.pdf]

reactome

Pathway Analysis Report

This report contains the pathway analysis results for the submitted sample ''. Analysis was performed against Reactome version 85 on 14/08/2023. The web link to these results is:

https://reactome.org/PathwayBrowser/#/ANALYSIS=MjAyMzA4MTQxMDA1MzhfODEwMQ%3D%3D

Please keep in mind that analysis results are temporarily stored on our server. The storage period depends on usage of the service but is at least 7 days. As a result, please note that this URL is only valid for a limited time period and it might have expired.

Table of Contents

1. Introduction
2. Properties
3. Genome-wide overview
4. Most significant pathways
5. Pathways details
6. Identifiers found
7. Identifiers not found

1. Introduction

Reactome is a curated database of pathways and reactions in human biology. Reactions can be considered as pathway 'steps'. Reactome defines a 'reaction' as any event in biology that changes the state of a biological molecule. Binding, activation, translocation, degradation and classical biochemical events involving a catalyst are all reactions. Information in the database is authored by expert biologists, entered and maintained by Reactome’s team of curators and editorial staff. Reactome content frequently cross-references other resources e.g. NCBI, Ensembl, UniProt, KEGG (Gene and Compound), ChEBI, PubMed and GO. Orthologous reactions inferred from annotation for Homo sapiens are available for 14 non-human species including mouse, rat, chicken, puffer fish, worm, fly and yeast. Pathways are represented by simple diagrams following an SBGN-like format.

Reactome's annotated data describe reactions possible if all annotated proteins and small molecules were present and active simultaneously in a cell. By overlaying an experimental dataset on these annotations, a user can perform a pathway over-representation analysis. By overlaying quantitative expression data or time series, a user can visualize the extent of change in affected pathways and its progression. A binomial test is used to calculate the probability shown for each result, and the p-values are corrected for the multiple testing (Benjamini–Hochberg procedure) that arises from evaluating the submitted list of identifiers against every pathway.

To learn more about our Pathway Analysis, please have a look at our relevant publications:

Fabregat A, Sidiropoulos K, Garapati P, Gillespie M, Hausmann K, Haw R, … D’Eustachio P (2016). The reactome pathway knowledgebase. Nucleic Acids Research, 44(D1), D481–D487. https://doi.org/10.1093/nar/gkv1351.

Fabregat A, Sidiropoulos K, Viteri G, Forner O, Marin-Garcia P, Arnau V, … Hermjakob H (2017). Reactome pathway analysis: a high-performance in-memory approach. BMC Bioinformatics, 18.

2. Properties

- This is an **overrepresentation** analysis: A statistical (hypergeometric distribution) test that determines whether certain Reactome pathways are over-represented (enriched) in the submitted data. It answers the question 'Does my list contain more proteins for pathway X than would be expected by chance?' This test produces a probability score, which is corrected for false discovery rate using the Benjamani-Hochberg method.
- 1 out of 2 identifiers in the sample were found in Reactome, where 6 pathways were hit by at least one of them.
- All non-human identifiers have been converted to their human equivalent.
- This report is filtered to show only results for species 'Homo sapiens' and resource 'UniProt'.
- The unique ID for this analysis (token) is MjAyMzA4MTQxMDA1MzhfODEwMQ%3D%3D. This ID is valid for at least 7 days in Reactome's server. Use it to access Reactome services with your data.

3. Genome-wide overview

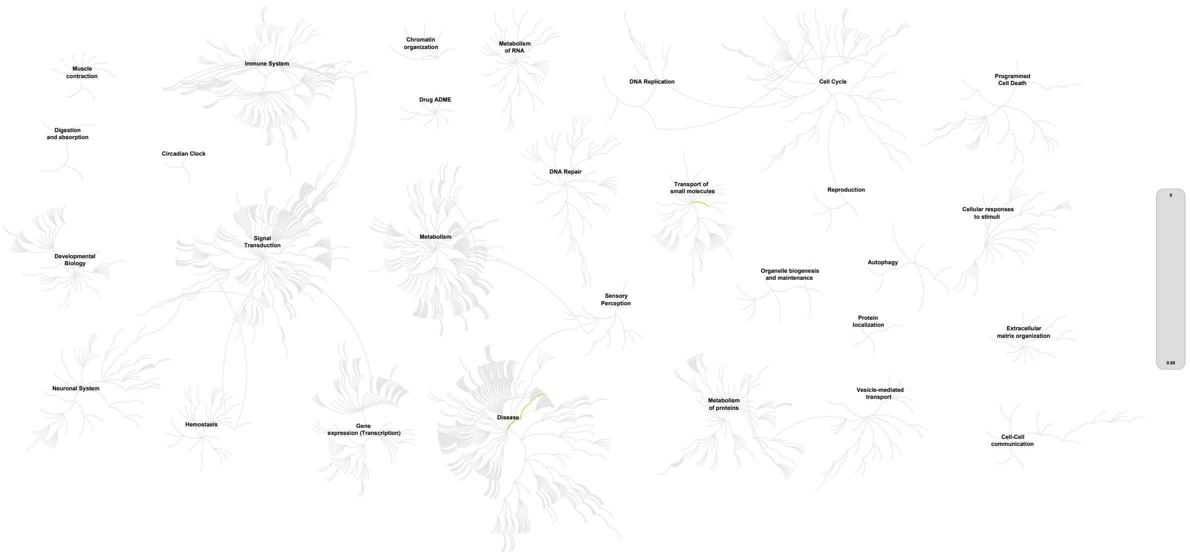

This figure shows a genome-wide overview of the results of your pathway analysis. Reactome pathways are arranged in a hierarchy. The center of each of the circular "bursts" is the root of one top-level pathway, for example "DNA Repair". Each step away from the center represents the next level lower in the pathway hierarchy. The color code denotes over-representation of that pathway in your input dataset. Light grey signifies pathways which are not significantly over-represented.

4. Most significant pathways

The following table shows the 6 most relevant pathways sorted by p-value.

Pathway name	Entities				Reactions	
	found	ratio	p-value	FDR*	found	ratio
Defective CFTR causes cystic fibrosis	1 / 61	0.005	0.01	0.031	3 / 8	5.59e-04
ABC transporter disorders	1 / 78	0.007	0.013	0.031	3 / 23	0.002
ABC-family proteins mediated transport	1 / 103	0.009	0.018	0.031	3 / 27	0.002
Disorders of transmembrane transporters	1 / 181	0.016	0.031	0.031	3 / 88	0.006
Transport of small molecules	1 / 729	0.063	0.122	0.122	3 / 454	0.032
Disease	1 / 2,101	0.181	0.329	0.329	3 / 1,787	0.125

* False Discovery Rate

5. Pathways details

For every pathway of the most significant pathways, we present its diagram, as well as a short summary, its bibliography and the list of inputs found in it.

1. Defective CFTR causes cystic fibrosis (R-HSA-5678895)

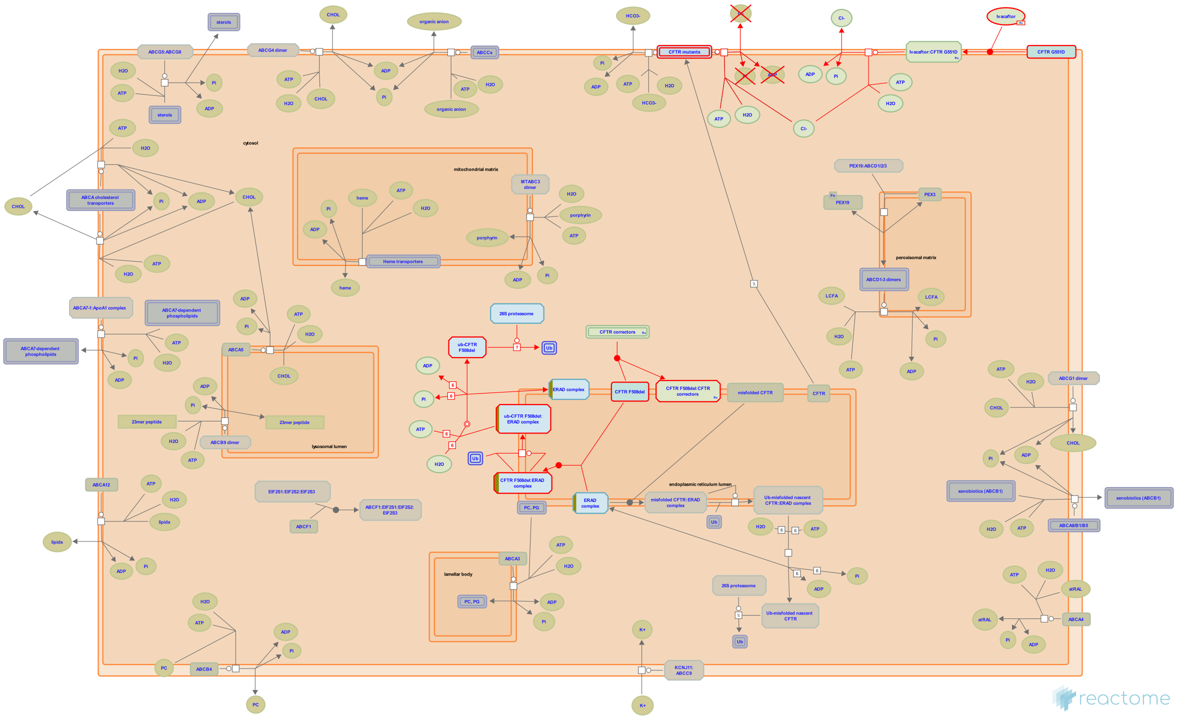

Diseases: cystic fibrosis.

Cystic fibrosis transmembrane conductance regulator (CFTR) is a low conductance chloride-selective channel that mediates the transport of chloride ions in human airway epithelial cells. Chloride ions plays a key role in maintaining homoeostasis of epithelial secretions in the lungs. Defects in CFTR can cause cystic fibrosis (CF; MIM:602421), a common generalised disorder in Caucasians affecting the exocrine glands. CF results in an ionic imbalance that impairs clearance of secretions, not only in the lung, but also in the pancreas, gastrointestinal tract and liver. Wide-ranging manifestations of the disease include chronic lung disease, exocrine pancreatic insufficiency, blockage of the terminal ileum, male infertility and salty sweat. The median survival of CF patients in North America and Western Europe is around 40 years (Davis 2006, Radlovic 2012).

References

Rogan MP, Stoltz DA & Hornick DB (2011). Cystic fibrosis transmembrane conductance regulator intracellular processing, trafficking, and opportunities for mutation-specific treatment. Chest, 139, 1480-90.

Markiewicz D, Buchanan JA, Buchwald M, Kerem B, Cox TK, Tsui LC, ... Rommens JM (1989). Identification of the cystic fibrosis gene: genetic analysis. Science, 245, 1073-80.

Davis PB (2006). Cystic fibrosis since 1938. Am. J. Respir. Crit. Care Med., 173, 475-82.

Radlovi? N (2012). Cystic fibrosis. Srp Arh Celok Lek, 140, 244-9.

Edit history

Date	Action	Author
2015-02-26	Edited	Jassal B
2015-02-26	Authored	Jassal B
2015-02-26	Created	Jassal B
2015-04-28	Reviewed	Moitra K
2023-03-08	Modified	Matthews L

1 submitted entities found in this pathway, mapping to 1 Reactome entities

Input	UniProt Id
Derl3	Q96Q80

2. ABC transporter disorders (R-HSA-5619084)

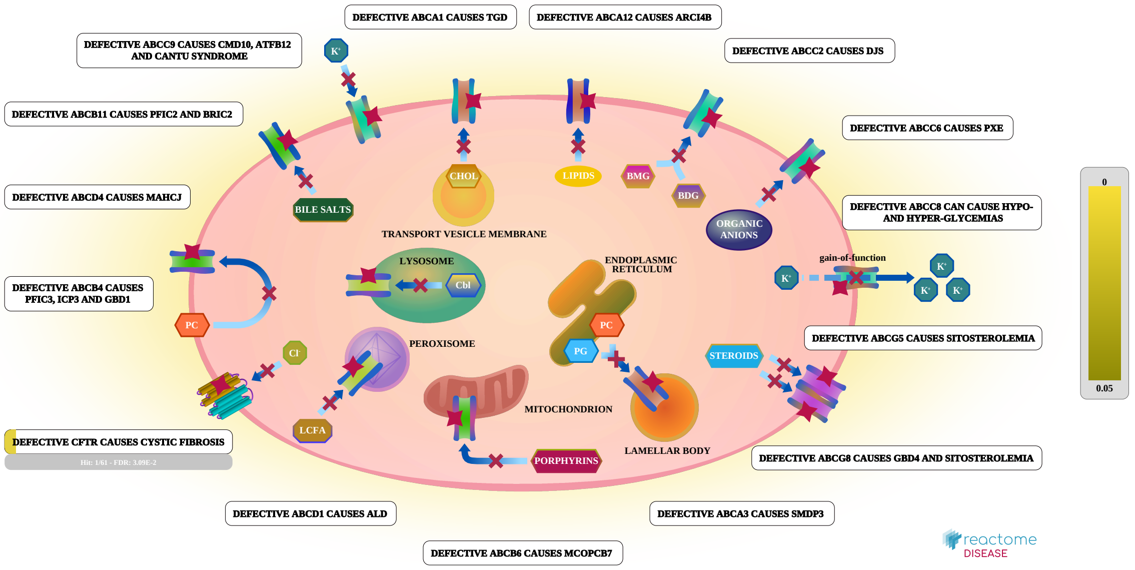

The ATP-binding cassette (ABC) transporters form a large family of transmembrane proteins that utilise the energy from the hydrolysis of ATP to facilitate the movement of a wide variety of substrates against a concentration gradient across membrane bilayers. Substrates include amino acids, lipids, inorganic ions, peptides, saccharides, peptides for antigen presentation, metals, drugs, and proteins. Of the 48 known ABC transporters in humans, 15 are associated with a defined human disease (Tarling et al. 2013, Woodward et al. 2011, Dean 2005, Kemp et al. 2011, Ueda 2011, Chen & Tiwari 2011).

References

Chen ZS & Tiwari AK (2011). Multidrug resistance proteins (MRPs/ABCCs) in cancer chemotherapy and genetic diseases. FEBS J., 278, 3226-45.

Edwards PA, de Aguiar Vallim TQ & Tarling EJ (2013). Role of ABC transporters in lipid transport and human disease. Trends Endocrinol. Metab., 24, 342-50.

Köttgen M, Köttgen A & Woodward OM (2011). ABCG transporters and disease. FEBS J., 278, 3215-25 .

Dean M (2005). The genetics of ATP-binding cassette transporters. Meth. Enzymol., 400, 409-29.

Kemp S, Wanders RJA & Theodoulou FL (2011). Mammalian peroxisomal ABC transporters: from endogenous substrates to pathology and clinical significance. Br. J. Pharmacol., 164, 1753-66.

Edit history

Date	Action	Author
2014-08-22	Edited	Jassal B
2014-08-22	Authored	Jassal B
2014-08-22	Created	Jassal B
2015-04-28	Reviewed	Moitra K
2015-09-15	Reviewed	Shukla S

Date	Action	Author
2023-03-08	Modified	Matthews L

1 submitted entities found in this pathway, mapping to 1 Reactome entities

Input	UniProt Id
Derl3	Q96Q80

3. ABC-family proteins mediated transport (R-HSA-382556)

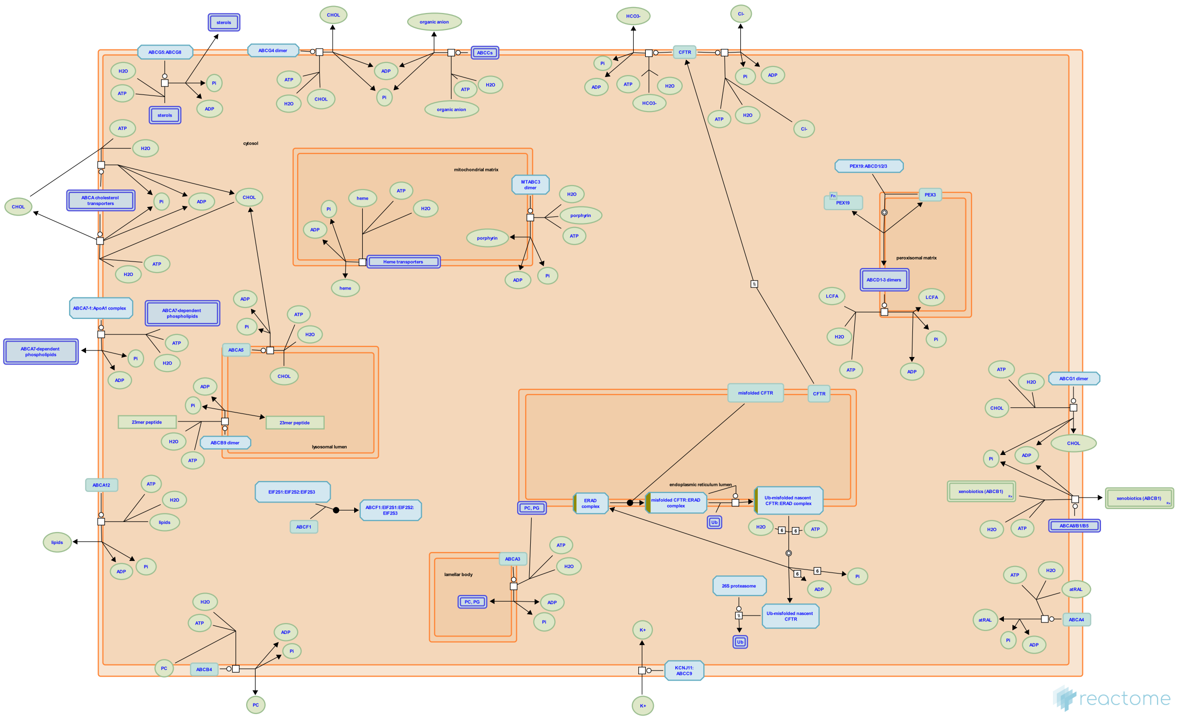

The ATP-binding cassette (ABC) superfamily of active transporters involves a large number of functionally diverse transmembrane proteins. They transport a variety of compounds through membranes against steep concentration gradients at the cost of ATP hydrolysis. These substrates include amino acids, lipids, inorganic ions, peptides, saccharides, peptides for antigen presentation, metals, drugs, and proteins. The ABC transporters not only move a variety of substrates into and out of the cell, but are also involved in intracellular compartmental transport. Energy derived from the hydrolysis of ATP is used to transport the substrate across the membrane against a concentration gradient. Human genome contains 48 ABC genes; 16 of these have a known function and 14 are associated with a defined human disease (Dean et al. 2001, Borst and Elferink 2002, Rees et al. 2009).

References

Lewinson O, Johnson E & Rees DC (2009). ABC transporters: the power to change. Nat Rev Mol Cell Biol, 10, 218-27.

Allikmets R, Rzhetsky A & Dean M (2001). The human ATP-binding cassette (ABC) transporter superfamily. Genome Res, 11, 1156-66.

Elferink RO & Borst P (2002). Mammalian ABC transporters in health and disease. Annu Rev Biochem, 71, 537-92.

Edit history

Date	Action	Author
2008-11-23	Authored	Gopinathrao G
2008-11-23	Created	Gopinathrao G
2008-11-29	Edited	Gopinathrao G
2008-12-02	Reviewed	Matthews L

Date	Action	Author
2011-08-23	Reviewed	D'Eustachio P
2023-05-21	Modified	Wright A

1 submitted entities found in this pathway, mapping to 1 Reactome entities

Input	UniProt Id
Derl3	Q96Q80

4. Disorders of transmembrane transporters (R-HSA-5619115)

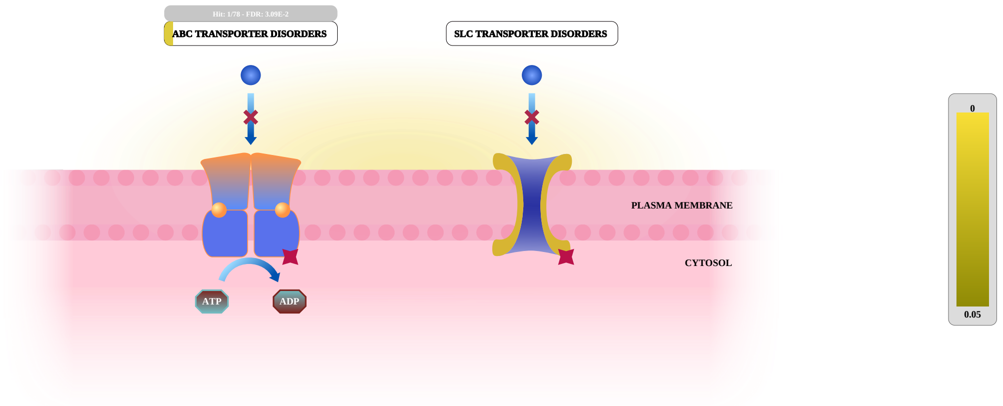

Proteins with transporting functions can be roughly classified into 3 categories: ATP hydrolysis-coupled pumps, ion channels, and transporters. Pumps utilize the energy released by ATP hydrolysis to power the movement of substrates across the membrane against their electrochemical gradient. Channels in their open state can transfer substrates (ions or water) down their electrochemical gradient at an extremely high efficiency (up to 108 s-1). Transporters facilitate the movement of a specific substrate either against or with their concentration gradient at a lower speed (about 102 -104 s-1); as generally believed, conformational change of the transporter protein is involved in the transfer process. Diseases caused by defects in these transporter proteins are detailed in this section. Disorders associated with ABC transporters and SLC transporters are annotated here (Dean 2005).

References

Dean M (2005). The genetics of ATP-binding cassette transporters. Meth. Enzymol., 400, 409-29.

Edit history

Date	Action	Author
2014-08-22	Edited	Jassal B
2014-08-22	Authored	Jassal B
2014-08-22	Created	Jassal B
2015-04-28	Reviewed	Moitra K
2023-03-08	Modified	Matthews L

1 submitted entities found in this pathway, mapping to 1 Reactome entities

Input	UniProt Id
Derl3	Q96Q80

5. Transport of small molecules (R-HSA-382551)

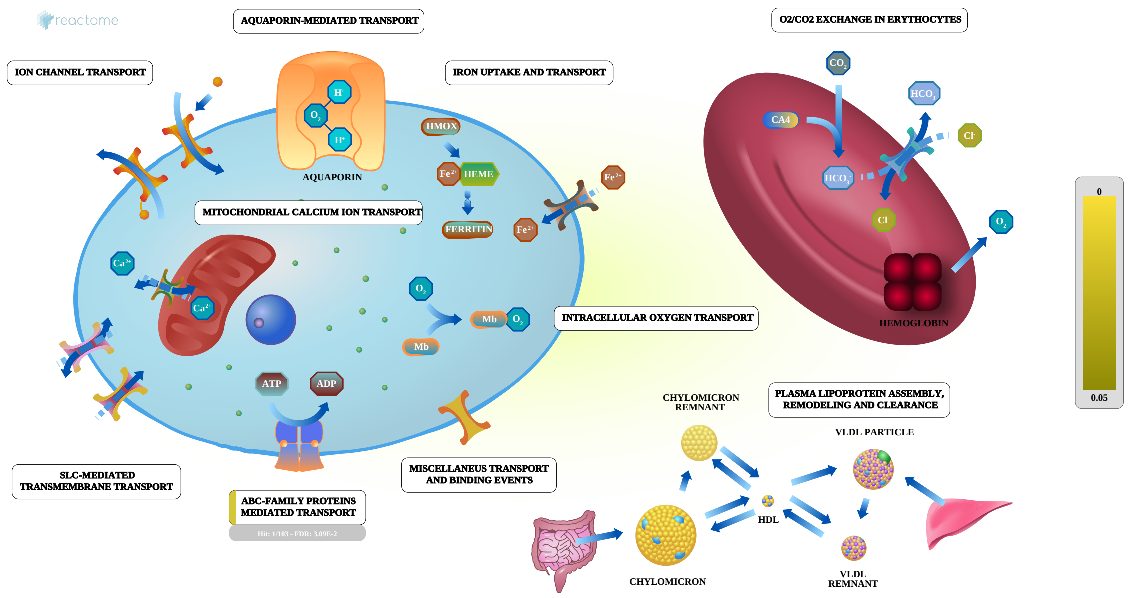

By definition cells have a critical separation between inner (cytoplasmic) and outer (extracellular) compartments. This separation provides for protection, gradient assembly, and environmental control but at the same time isolates the interior compartments of the cell from energy resources, oxygen, and raw materials. Cells have evolved a myriad of mechanisms to regulate, and enable transportation of small molecules ascross plasma membranes and between cellular organelle compartments within cells.

References

Edit history

Date	Action	Author
2008-11-23	Created	Gopinathrao G
2008-12-02	Reviewed	Jassal B, Wright EM, Matthews L
2014-06-09	Revised	Jassal B
2023-05-21	Modified	Wright A

1 submitted entities found in this pathway, mapping to 1 Reactome entities

Input	UniProt Id
Derl3	Q96Q80

6. Disease (R-HSA-1643685)

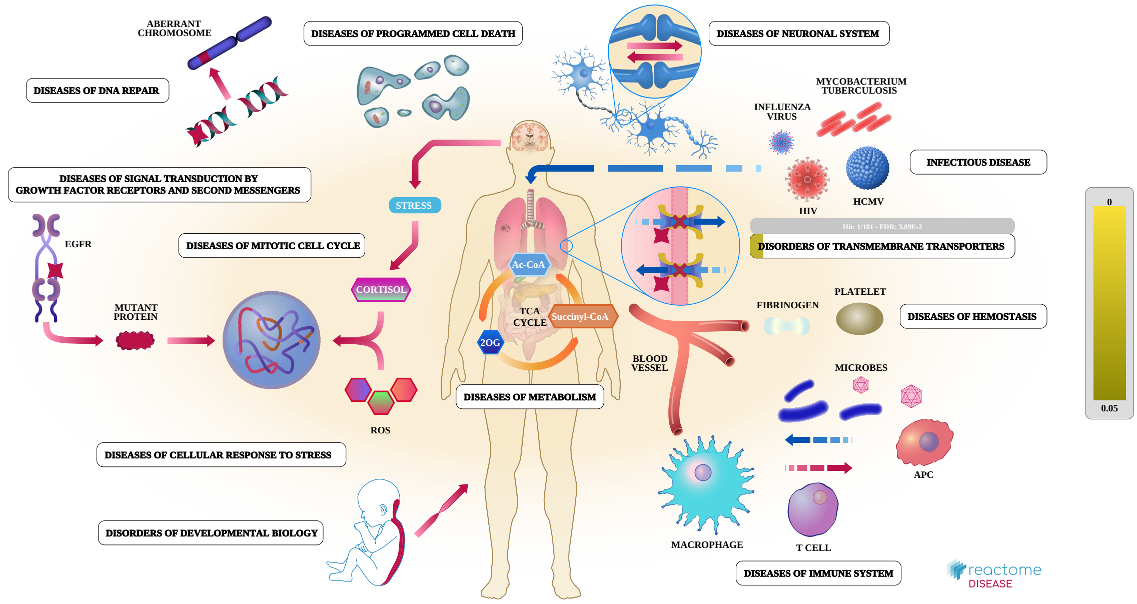

Biological processes are captured in Reactome by identifying the molecules (DNA, RNA, protein, small molecules) involved in them and describing the details of their interactions. From this molecular viewpoint, human disease pathways have three mechanistic causes: the inclusion of microbially-expressed proteins, altered functions of human proteins, or changed expression levels of otherwise functionally normal human proteins.

The first group encompasses the infectious diseases such as influenza, tuberculosis and HIV infection. The second group involves human proteins modified either by a mutation or by an abnormal post-translational event that produces an aberrant protein with a novel function. Examples include somatic mutations of EGFR and FGFR (epidermal and fibroblast growth factor receptor) genes, which encode constitutively active receptors that signal even in the absence of their ligands, or the somatic mutation of IDH1 (isocitrate dehydrogenase 1) that leads to an enzyme active on 2-oxoglutarate rather than isocitrate, or the abnormal protein aggregations of amyloidosis which lead to diseases such as Alzheimer's.

Infectious diseases are represented in Reactome as microbial-human protein interactions and the consequent events. The existence of variant proteins and their association with disease-specific biological processes is represented by inclusion of the modified protein in a new or variant reaction, an extension to the 'normal' pathway. Diseases which result from proteins performing their normal functions but at abnormal rates can also be captured, though less directly. Many mutant alleles encode proteins that retain their normal functions but have abnormal stabilities or catalytic efficiencies, leading to normal reactions that proceed to abnormal extents. The phenotypes of such diseases can be revealed when pathway annotations are combined with expression or rate data from other sources.

Depending on the biological pathway/process immediately affected by disease-causing gene variants, non-infectious diseases in Reactome are organized into diseases of signal transduction by growth factore receptors and second messengers, diseases of mitotic cell cycle, diseases of cellular response to stress, diseases of programmed cell death, diseases of DNA repair, disorders of transmembrane transporters, diseases of metabolism, diseases of immune system, diseases of neuronal system, disorders of developmental biology, disorders of extracellular matrix organization, and diseases of hemostatis.

References

Edit history

Date	Action	Author
2011-10-11	Created	Matthews L
2020-08-24	Edited	Orlic-Milacic M
2023-03-08	Modified	Matthews L

1 submitted entities found in this pathway, mapping to 1 Reactome entities

Input	UniProt Id
Derl3	Q96Q80

6. Identifiers found

Below is a list of the input identifiers that have been found or mapped to an equivalent element in Reactome, classified by resource.

1 of the submitted entities were found, mapping to 1 Reactome entities

Input	UniProt Id
Derl3	Q96Q80

7. Identifiers not found

These 1 identifiers were not found neither mapped to any entity in Reactome.

Mki67
