# Supplementary material for: Sex- and region-specific cortical and hippocampal whole genome transcriptome profiles from control and APP/PS1 Alzheimer’s disease mice
Source: PLoS One. 2024 Feb 7;19(2):e0296959. doi: 10.1371/journal.pone.0296959 (PMC10849391; doi:10.1371/journal.pone.0296959)
Supplement: S1 File — S1 Fig: Genotyping of APP/PS1 AD mice and WT control animals. S2 Fig: 3D image of the murine brain including the RS cortex and hippocampus (BROIs) used for transcriptome analysis in our study. S3 Fig: PCA of transcriptomes from the RS cortex and hippocampus of WT controls and APP/PS1 AD mice of both sexes. S4 Fig: Hierarchical clustering of transcriptome data from the RS cortex and hippocampus of WT control and APP/PS1 AD mice of both sexes. S5 Fig: Bar diagrams of the top 30 candidates of DEGs with highest significant FCs (FC > 1.5 and FC < -1.5, p < 0.05). S6 Fig: Pathway analysis of intersectional and signature gene sets in APP/PS1 subgroups. S7 Fig: Comparative qPCR analysis of selected gene transcript levels from the hippocampus of female and male APP/PS1 AD with 5XFAD mice. S1 Table: PCR reaction set-up using PCR Mastermix and genomic DNA. S2 Table: Materials used for one-color microarray-based gene expression data collection. S3 Table: Software used for one-color microarray-based gene expression data collection. S4 Table: Details on genes, forward and reverse primer sequences and annealing temperatures relevant for qPCR experimentation. S5 Table: Characteristics of DEGs in the RS cortex of female APP/PS1 AD mice. S6 Table: Characteristics of DEGs in the hippocampus of female APP/PS1 AD mice. S7 Table: Characteristics of DEGs in the RS cortex of male APP/PS1 AD mice. S8 Table: Characteristics of DEGs in the hippocampus of male APP/PS1 AD mice. S9 Table: Venn analysis of DEGs in the RS cortex and hippocampus of female APP/PS1 AD mice. S10 Table: Venn analysis of DEGs genes in the RS cortex and hippocampus of male APP/PS1 AD mice. S11 Table: Venn analysis of DEGs in the RS cortex of male and female APP/PS1 AD mice. S12 Table: Venn analysis of DEGs in the hippocampus of male and female APP/PS1 AD mice. S13 Table: Differentially regulated l(i)ncRNAs in APP/PS1 AD vs. WT mice. S14 Table: qPCR-based FC analysis of selected genes in the hippocampus of APP/PS1 AD vs. [file pone.0296959.s001.zip › Supplementary Files_R1/Supplementary Table 1_PCR_Genotyping.pdf]

Supplementary Table 1: Composition of PCR reaction mix used for genotyping.

Reagent	*Volume per reaction (µl)*
ddH_2O	8.7
Forward Primer APP (25 µM)	0.3
Reverse Primer APP (25 µM)	0.3
Forward Primer PS1 (25 µM)	0.3
Reverse Primer PS1 (25 µM)	0.3
Forward Primer WT control (25 µM)	0.3
Reverse Primer WT control (25 µM)	0.3
Red Taq Ready Master Mix	12.5
genomic DNA (~ 20 ng/ml)	2
Total volume	*25*
