# Supplementary material for: Sex- and region-specific cortical and hippocampal whole genome transcriptome profiles from control and APP/PS1 Alzheimer’s disease mice
Source: PLoS One. 2024 Feb 7;19(2):e0296959. doi: 10.1371/journal.pone.0296959 (PMC10849391; doi:10.1371/journal.pone.0296959)
Supplement: S1 File — S1 Fig: Genotyping of APP/PS1 AD mice and WT control animals. S2 Fig: 3D image of the murine brain including the RS cortex and hippocampus (BROIs) used for transcriptome analysis in our study. S3 Fig: PCA of transcriptomes from the RS cortex and hippocampus of WT controls and APP/PS1 AD mice of both sexes. S4 Fig: Hierarchical clustering of transcriptome data from the RS cortex and hippocampus of WT control and APP/PS1 AD mice of both sexes. S5 Fig: Bar diagrams of the top 30 candidates of DEGs with highest significant FCs (FC > 1.5 and FC < -1.5, p < 0.05). S6 Fig: Pathway analysis of intersectional and signature gene sets in APP/PS1 subgroups. S7 Fig: Comparative qPCR analysis of selected gene transcript levels from the hippocampus of female and male APP/PS1 AD with 5XFAD mice. S1 Table: PCR reaction set-up using PCR Mastermix and genomic DNA. S2 Table: Materials used for one-color microarray-based gene expression data collection. S3 Table: Software used for one-color microarray-based gene expression data collection. S4 Table: Details on genes, forward and reverse primer sequences and annealing temperatures relevant for qPCR experimentation. S5 Table: Characteristics of DEGs in the RS cortex of female APP/PS1 AD mice. S6 Table: Characteristics of DEGs in the hippocampus of female APP/PS1 AD mice. S7 Table: Characteristics of DEGs in the RS cortex of male APP/PS1 AD mice. S8 Table: Characteristics of DEGs in the hippocampus of male APP/PS1 AD mice. S9 Table: Venn analysis of DEGs in the RS cortex and hippocampus of female APP/PS1 AD mice. S10 Table: Venn analysis of DEGs genes in the RS cortex and hippocampus of male APP/PS1 AD mice. S11 Table: Venn analysis of DEGs in the RS cortex of male and female APP/PS1 AD mice. S12 Table: Venn analysis of DEGs in the hippocampus of male and female APP/PS1 AD mice. S13 Table: Differentially regulated l(i)ncRNAs in APP/PS1 AD vs. WT mice. S14 Table: qPCR-based FC analysis of selected genes in the hippocampus of APP/PS1 AD vs. [file pone.0296959.s001.zip › Supplementary Files_R1/Supplementary Table 14_R1_qPCR_FC.pdf]

Supplementary Table 14A: qPCR-based fold change analysis of selected genes in the hippocampus of APP/PS1 mice that were previously identified in the 5XFAD AD model (Siwek et al, 2015).

(i) FCs and related p-values in female APP/PS1 mice versus controls.

**Gene**	**Fold-change**	**p-value**
Cacna1c	1.1047	1.000
Cacna1d	1.1681	0.114
Plcd4	-1.0829	0.485
**Casp8**	**1.3674**	**0.028**
Chrm1	1.1682	0.8857
Chrm3	1.1711	0.3428
Chrm5	-1.0344	0.485

(ii) FCs and related p-values in male APP/PS1 mice versus controls.

**Gene**	**Fold-change**	**p-value**
Cacna1c	-2.2217	0.114
**Cacna1d**	**-1.2122**	**0.0285**
Plcd4	1.0201	0.885
Casp8	1.0892	0.685
Chrm1	-1.0418	0.685
Chrm3	-1.1810	0.485
Chrm5	-1.1284	0.885

Supplementary Table 14B: qPCR-based fold change analysis of selected DEGs (from our transcriptome study) in the RS cortex of APP/PS1 mice vs. WT control animals. Fold changes from qPCR studies are listed first, those from microarrays are given in brackets.

(i) FCs and related p-values in female APP/PS1 mice versus controls.

**Gene**	**Fold-change (Microarrays)**	**p-value**
**Siglech**	**1.39 (+2.046)**	**0.032**
**Ptpn6**	**1.744 (+1.853)**	**0.032**
**Laptm5**	**2.376 (+1.646)**	**0.016**
**Plek**	**1.609 (+1.651)**	**0.016**
**Arpp21**	**-1.591 (- 1.464)**	**0.016**
**Shisa9**	**-1.427 (-1.048)**	**0.016**

(ii) FCs and related p-values in male APP/PS1 mice versus controls.

**Gene**	**Fold-change (Microarrays)**	**p-value**
**Siglech**	**1.523 (+1.720)**	**0.051**
**Ptpn6**	**1.877 (+1.538)**	**0.023**
**Laptm5**	**2.391 (+1.583)**	**0.009**
**Plek**	**1.738 (+1.309)**	**0.039**
**Arpp21**	**-1.395 (-1.413)**	**0.039**
Shisa9	-1.194 (-1.107)	0.437
