# Supplementary material for: Sex- and region-specific cortical and hippocampal whole genome transcriptome profiles from control and APP/PS1 Alzheimer’s disease mice
Source: PLoS One. 2024 Feb 7;19(2):e0296959. doi: 10.1371/journal.pone.0296959 (PMC10849391; doi:10.1371/journal.pone.0296959)
Supplement: S1 File — S1 Fig: Genotyping of APP/PS1 AD mice and WT control animals. S2 Fig: 3D image of the murine brain including the RS cortex and hippocampus (BROIs) used for transcriptome analysis in our study. S3 Fig: PCA of transcriptomes from the RS cortex and hippocampus of WT controls and APP/PS1 AD mice of both sexes. S4 Fig: Hierarchical clustering of transcriptome data from the RS cortex and hippocampus of WT control and APP/PS1 AD mice of both sexes. S5 Fig: Bar diagrams of the top 30 candidates of DEGs with highest significant FCs (FC > 1.5 and FC < -1.5, p < 0.05). S6 Fig: Pathway analysis of intersectional and signature gene sets in APP/PS1 subgroups. S7 Fig: Comparative qPCR analysis of selected gene transcript levels from the hippocampus of female and male APP/PS1 AD with 5XFAD mice. S1 Table: PCR reaction set-up using PCR Mastermix and genomic DNA. S2 Table: Materials used for one-color microarray-based gene expression data collection. S3 Table: Software used for one-color microarray-based gene expression data collection. S4 Table: Details on genes, forward and reverse primer sequences and annealing temperatures relevant for qPCR experimentation. S5 Table: Characteristics of DEGs in the RS cortex of female APP/PS1 AD mice. S6 Table: Characteristics of DEGs in the hippocampus of female APP/PS1 AD mice. S7 Table: Characteristics of DEGs in the RS cortex of male APP/PS1 AD mice. S8 Table: Characteristics of DEGs in the hippocampus of male APP/PS1 AD mice. S9 Table: Venn analysis of DEGs in the RS cortex and hippocampus of female APP/PS1 AD mice. S10 Table: Venn analysis of DEGs genes in the RS cortex and hippocampus of male APP/PS1 AD mice. S11 Table: Venn analysis of DEGs in the RS cortex of male and female APP/PS1 AD mice. S12 Table: Venn analysis of DEGs in the hippocampus of male and female APP/PS1 AD mice. S13 Table: Differentially regulated l(i)ncRNAs in APP/PS1 AD vs. WT mice. S14 Table: qPCR-based FC analysis of selected genes in the hippocampus of APP/PS1 AD vs. [file pone.0296959.s001.zip › Supplementary Files_R1/Supplementary Table 15_reference list.pdf]

Reference list

1. Dickey CA, Loring JF, Montgomery J, Gordon MN, Eastman PS, Morgan D. Selectively reduced expression of synaptic plasticity-related genes in amyloid precursor protein + presenilin-1 transgenic mice. J Neurosci. 2003;23(12):5219-26. doi: 10.1523/JNEUROSCI.23-12-05219.2003. PubMed PMID: 12832546; PubMed Central PMCID: PMCPMC6741153.
2. Wirz KT, Bossers K, Stargardt A, Kamphuis W, Swaab DF, Hol EM, Verhaagen J. Cortical beta amyloid protein triggers an immune response, but no synaptic changes in the APPswe/PS1dE9 Alzheimer's disease mouse model. Neurobiol Aging. 2013;34(5):1328-42. Epub 20121212. doi: 10.1016/j.neurobiolaging.2012.11.008. PubMed PMID: 23245294.
3. Lopez-Gonzalez I, Schluter A, Aso E, Garcia-Esparcia P, Ansoleaga B, F LL, et al. Neuroinflammatory signals in Alzheimer disease and APP/PS1 transgenic mice: correlations with plaques, tangles, and oligomeric species. J Neuropathol Exp Neurol. 2015;74(4):319-44. doi: 10.1097/NEN.0000000000000176. PubMed PMID: 25756590.
4. Fang M, Zhang P, Zhao Y, Liu X. Bioinformatics and co-expression network analysis of differentially expressed lncRNAs and mRNAs in hippocampus of APP/PS1 transgenic mice with Alzheimer disease. Am J Transl Res. 2017;9(3):1381-91. Epub 20170315. PubMed PMID: 28386363; PubMed Central PMCID: PMCPMC5376028.
5. Abid NB, Naseer MI, Kim MO. Comparative Gene-Expression Analysis of Alzheimer's Disease Progression with Aging in Transgenic Mouse Model. Int J Mol Sci. 2019;20(5). Epub 20190311. doi: 10.3390/ijms20051219. PubMed PMID: 30862043; PubMed Central PMCID: PMCPMC6429175.
6. Ma N, Pan J, Ye X, Yu B, Zhang W, Wan J. Whole-Transcriptome Analysis of APP/PS1 Mouse Brain and Identification of circRNA-miRNA-mRNA Networks to Investigate AD Pathogenesis. Mol Ther Nucleic Acids. 2019;18:1049-62. Epub 20191106. doi: 10.1016/j.omtn.2019.10.030. PubMed PMID: 31786335; PubMed Central PMCID: PMCPMC6906698.
7. Wang L, Zeng L, Jiang H, Li Z, Liu R. Microarray Profile of Long Noncoding RNA and Messenger RNA Expression in a Model of Alzheimer's Disease. Life (Basel). 2020;10(5). Epub 20200514. doi: 10.3390/life10050064. PubMed PMID: 32423012; PubMed Central PMCID: PMCPMC7281340.
8. Yan H, Yan Y, Gao Y, Zhang N, Kumar G, Fang Q, et al. Transcriptome analysis of fasudil treatment in the APPswe/PSEN1dE9 transgenic (APP/PS1) mice model of Alzheimer's disease. Sci Rep. 2022;12(1):6625. Epub 20220422. doi: 10.1038/s41598-022-10554-9. PubMed PMID: 35459923; PubMed Central PMCID: PMCPMC9033779.
9. Kim TK, Lee S, Im HI. Quantitative Sequencing Analysis of the Striatal Transcriptome in a Mouse Model of Alzheimer Disease. Int Neurourol J. 2022;26(Suppl 2):S117-25. Epub 20221130. doi: 10.5213/inj.2244256.128. PubMed PMID: 36503214; PubMed Central PMCID: PMCPMC9767686.
10. Jiwaji Z, Tiwari SS, Aviles-Reyes RX, Hooley M, Hampton D, Torvell M, et al. Reactive astrocytes acquire neuroprotective as well as deleterious signatures in response to Tau and Ass pathology. Nat Commun. 2022;13(1):135. Epub 20220110. doi: 10.1038/s41467-021-27702-w. PubMed PMID: 35013236; PubMed Central PMCID: PMCPMC8748982.
