# Supplementary material for: Sex- and region-specific cortical and hippocampal whole genome transcriptome profiles from control and APP/PS1 Alzheimer’s disease mice
Source: PLoS One. 2024 Feb 7;19(2):e0296959. doi: 10.1371/journal.pone.0296959 (PMC10849391; doi:10.1371/journal.pone.0296959)
Supplement: S1 File — S1 Fig: Genotyping of APP/PS1 AD mice and WT control animals. S2 Fig: 3D image of the murine brain including the RS cortex and hippocampus (BROIs) used for transcriptome analysis in our study. S3 Fig: PCA of transcriptomes from the RS cortex and hippocampus of WT controls and APP/PS1 AD mice of both sexes. S4 Fig: Hierarchical clustering of transcriptome data from the RS cortex and hippocampus of WT control and APP/PS1 AD mice of both sexes. S5 Fig: Bar diagrams of the top 30 candidates of DEGs with highest significant FCs (FC > 1.5 and FC < -1.5, p < 0.05). S6 Fig: Pathway analysis of intersectional and signature gene sets in APP/PS1 subgroups. S7 Fig: Comparative qPCR analysis of selected gene transcript levels from the hippocampus of female and male APP/PS1 AD with 5XFAD mice. S1 Table: PCR reaction set-up using PCR Mastermix and genomic DNA. S2 Table: Materials used for one-color microarray-based gene expression data collection. S3 Table: Software used for one-color microarray-based gene expression data collection. S4 Table: Details on genes, forward and reverse primer sequences and annealing temperatures relevant for qPCR experimentation. S5 Table: Characteristics of DEGs in the RS cortex of female APP/PS1 AD mice. S6 Table: Characteristics of DEGs in the hippocampus of female APP/PS1 AD mice. S7 Table: Characteristics of DEGs in the RS cortex of male APP/PS1 AD mice. S8 Table: Characteristics of DEGs in the hippocampus of male APP/PS1 AD mice. S9 Table: Venn analysis of DEGs in the RS cortex and hippocampus of female APP/PS1 AD mice. S10 Table: Venn analysis of DEGs genes in the RS cortex and hippocampus of male APP/PS1 AD mice. S11 Table: Venn analysis of DEGs in the RS cortex of male and female APP/PS1 AD mice. S12 Table: Venn analysis of DEGs in the hippocampus of male and female APP/PS1 AD mice. S13 Table: Differentially regulated l(i)ncRNAs in APP/PS1 AD vs. WT mice. S14 Table: qPCR-based FC analysis of selected genes in the hippocampus of APP/PS1 AD vs. [file pone.0296959.s001.zip › Supplementary Files_R1/Supplementary Table 16_DEGs list_functions.pdf]

Supplementary Table 16: Functional implications of DEGs in AD. The list presents gene symbols (top 30, FC > 1.5, in alphabetical order), gene descriptions, functional characteristics and potential roles in AD etiopathogenesis. Note that the information for each individual gene represents a brief summary derived from *Gene* (https://www.ncbi.nlm.nih.gov/gene/) and is not exhaustive in nature. Instead, it is supposed to provide a short and fast orientation for the reader.

Upregulated DEGs (top 30 from the individual subgroups, FC > 1.5):

1. ***Adora3* (adenosine receptor A3):**

   Adora3 encodes a protein of the adenosine receptors (AR) family. The latter are G-protein-coupled receptors (GPCRs) involved in a variety of intracellular signaling pathways and physiological processes. The latter include, i.a., inhibition of neutrophil degranulation in neutrophil-mediated tissue injury, implication in both neuroprotective and neurodegenerative effects, and mediation of both cell proliferation and cell death.

2. ***Aif1* (allograft inflammatory factor 1):**

   Aif encodes a protein that enables actin filament and Ca^{2+} binding activity. It is involved in several processes, including Rac protein signal transduction, actin filament organization and assembly, and ruffle assembly. Allograft inflammatory factor 1 is located in several cellular components, including the actin filament apparatus, phagocytic cups, and ruffle membranes.

3. ***Apbb1ip* (amyloid beta precursor protein binding family B member 1 interacting protein):**

   Apbb1ip encodes a protein that acts upstream of or within T cell activation via T cell receptor contact with antigen bound to MHC molecules on antigen

presenting cells. Furthermore, its involved in the regulation of cell adhesion. It's located in the cytosol and plasma membrane and part of the T cell receptor complex.

4. ***Arhgap9* (Rho GTPase activating protein 9):**
   Arhgap9 encodes a protein that enables GTPase activator activity and phosphatidylinositol-3,4,5-trisphosphate binding activity. It is involved in regulation of GTPase activity and active in the cytoplasm.

5. ***Aspg* (asparaginase):**
   Aspg encodes a protein that is involved in asparagine and phospholipid metabolic process. It's also involved in further lipid catabolic processes.

6. ***B2m* (beta-2 microglobulin):**
   B2m encodes a protein that enables MHC class II protein complex binding activity and protein homodimerization activity. It is involved in learning or memory, the modulation of age-related behavioral decline, and negative regulation of cell differentiation. It acts upstream of or within several processes, including antigen processing and presentation of exogenous protein antigen via MHC class Ib, TAP-dependent; cellular response to iron (III) ions; and protein refolding. Beta-2 microglobulin is located in the external side of the plasma membrane.

7. ***Bcl2a1d* (B cell leukemia/lymphoma 2 related protein A1d):**
   Bcl2a1d encodes a protein that exhibits cysteine-type endopeptidase inhibitor activity involved in apoptotic process. It acts upstream of or within T cell differentiation, T cell receptor signaling pathway, and is engaged in apoptotic processes.

8. *Bcl3* **(B cell leukemia/lymphoma 3):**

   Bcl3 encodes a protein that mediates transcription coactivator activity. It acts upstream of or within several processes, i.a., the defense response to other organism, hematopoietic or lymphoid organ development, and the regulation of gene expression. B cell leukemia/lymphoma 3 is located in several cellular components, including the cytosol, midbody, and nucleoplasm. Structurally, it constitutes part of the Bcl3-Bcl10 and the Bcl3/NF-kappaB2 complex.

9. ***Capg*** **(capping actin protein, gelsolin like):**

   Capg encodes a protein that enables actin filament binding activity, phosphatidylinositol-4,5-bisphosphate binding activity and protein domain specific binding activity. It acts upstream of or within cell projection assembly and cellular responses to interferon-γ. The gelsolin like capping actin protein is located in phagocytic vesicles.

10. ***C1qa*** **(complement C1q A chain):**

    C1qa encodes a protein that is involved in several processes, including complement-mediated synapse pruning, microglial cell activation, and nervous system development. Complement C1q A chain acts upstream of or within complement activation and is located in the postsynapse.

11. ***C1qb*** **(complement C1q B chain):**

    C1qb encodes a complement factor that is engaged in synapse pruning and microglial activation. It is located in the postsynapse.

12. ***C1qc*** **(complement C1q C chain):**

    C1qc encodes a complement factor that is involved in synapse pruning and microglial activation. The complement C1q C chain is located in the postsynapse.

13. ***C4b* (component 4B, Chido blood group):**

C4b encodes a protein that enables carbohydrate and complement component *C1q* complex binding activity. It acts upstream of or within complement activation and immunoglobulin mediated immune response. It is located in the extracellular space.

14. ***Ccl6* (chemokine (C-C motif) ligand 6):**

Ccl6 encodes a protein that mediates CCR1 chemokine receptor binding activity, chemoattractant activity, and chemokine activity. It is involved in several processes, including cellular response to cytokine stimuli, leukocyte chemotaxis and positive regulation of the ERK1 and the ERK2 cascade. Chemokine (C-C motif) ligand 6 is located and active in the extracellular space.

15. ***Cd14* (CD14 antigen):**

Cd14 encodes a protein that plays an important role in the innate immune response and is expressed in monocyte/macrophage cells. It acts as a co-receptor that binds several microbial and fungal molecules, including lipopolysaccharides (LPS). It's LPS-binding activity is enhanced by the LPS binding protein (LBP) to allow binding to the TLR4-MD-2 coreceptor complex. The CD14 antigen is present in two forms, either as a soluble protein or attached to the cell surface by a glycosylphosphatidylinositol anchor.

16. ***Cd300c2* (CD300C molecule 2):**

Cd300c2 encodes a protein that mediates transmembrane signaling receptor activity. It acts upstream of or within positive regulation of cytokine production. The CD300C molecule 2 is located in the plasma membrane.

17. ***Cd48* (CD48 antigen):**

Cd48 encodes a protein that enables antigen binding activity and signaling receptor activity. It acts upstream of or within T cell activation and signal transduction. The CD48 antigen is located in the external side of the plasma membrane.

18. ***Cd52* (CD52 antigen):**

The gene product of *Cd52* acts upstream of or within response to bacteria.

19. ***Cd68* (CD68 antigen):**

The gene product of *Cd68* is involved in several processes, including cellular response to lipopolysaccharides, cellular response to oxidized low-density lipoprotein (LDL) particle stimulus, and negative regulation of dendritic cell antigen processing and presentation. The CD68 antigen acts upstream of or within aging processes and cellular responses to organic substances. The CD68 antigen is located in lysosomes and the plasma membrane and strongly expressed in the CNS.

20. ***Cep126* (centrosomal protein 126):**

Cep126 encodes a protein that is involved in cilium assembly, cytoplasmic microtubule organization, and mitotic spindle organization. The centrosomal protein 126 is located in cell projections, the cytoplasm, and cytoskeleton. It's active in the centrosome, the ciliary base, and the midbody.

21. ***Csf2rb* (colony stimulating factor 2 receptor subunit beta):**

Csf2rb encodes a protein that enables cytokine receptor activity. It acts upstream of or within the cytokine-mediated signaling pathway and the regulation of cell growth. The colony stimulating factor 2 receptor subunit beta is an integral component of the membrane.

22. ***Ctsd*** **(cathepsin D):**

The gene product of *Ctsd*, cathepsin D, mediates aspartic-type endopeptidase activity. It acts upstream of or within the autophagosome assembly. Cathepsin D is located in the extracellular space and lysosomes.

23. ***Ctsh*** **(cathepsin H):**

This gene encodes, cathepsin H, a member of the peptidase C1 (papain) family of cysteine proteases. Alternative splicing results in multiple transcript variants, at least one of which encodes a preproprotein that is proteolytically processed to generate multiple protein products. These products include the cathepsin H mini, heavy, and light chains. In rat and human, these three chains can associate to form the mature enzyme, which has both aminopeptidase and endopeptidase activities.

24. ***Ctss*** **(cathepsin S):**

This gene encodes cathepsin S, a member of the peptidase C1 (papain) family of cysteine proteases. Alternative splicing results in multiple transcript variants, which encode preproproteins that are proteolytically processed to generate mature protein products. This enzyme is secreted by antigen-presenting cells during inflammation and may induce pain and itch via activation of G-protein coupled receptors (GPCRs).

25. ***Cyba*** **(cytochrome B-245 alpha chain):**

The gene product of *Cyba* exhibits SH3 domain binding activity, electron transfer and protein heterodimerization activity. It also contributes to the superoxide-generating NAD(P)H oxidase action. The cytochrome B-245 alpha chain is involved in innate immune response. It acts upstream of or within positive regulation of mucus secretion, reactive oxygen species metabolic

process, and regulation of release of sequestered Ca^{2+} ions into the cytosol. It is located in endosomes and part of the NADPH oxidase complex.

26. ***Fam46c* (family with sequence similarity 46, member C):**

The function of this gene product is largely unknown. It may be involved in type 1 interferon response.

27. ***Fcer1g* (Fc epsilon receptor Ig):**

This gene encodes for a protein with IgE receptor activity and IgG binding activity and is part of Fc-epsilon receptor I complex. It is involved in several processes, including cell surface receptor signaling pathway, positive regulation of interleukin-4 production, and serotonin secretion via platelets. It further acts upstream of or within several processes, including leukocyte differentiation, positive regulation of cytokine production, and positive regulation of immune response. The Fc epsilon receptor Ig is located in the external side of the plasma membrane.

28. ***Fcgr1* (Fc receptor, IgG, high affinity I):**

The gene product of *Fcgr1* enables IgG binding and receptor activity. It acts upstream of or within several processes, including antibody-dependent cellular cytotoxicity, endocytosis, and positive regulation of hypersensitivity. The high affinity IgG Fc receptor is located in the external side of the plasma membrane.

29. ***Fcgr2b* (Fc receptor, IgG, low affinity IIb):**

This gene encodes a protein that enables IgG binding activity. It is involved in several processes, including cellular response to Aβ, nervous system development, and regulation of cellular responses to stress. It acts upstream of or within several processes, including antigen processing and presentation of exogenous peptide antigen via MHC class II, negative regulation of immune

effector processes, and regulation of phagocytosis. The low affinity IIb IgG Fc receptor is located in the cell body, dendritic spines, and the external side of the plasma membrane with an integral component.

30. ***Fcgr3*** **(Fc receptor, IgG, low affinity III):**

This gene encodes a protein that enables IgG binding and IgG receptor activity. It acts upstream of or within several processes, including antibody-dependent cellular cytotoxicity, phagocytosis, and positive regulation of hypersensitivity. It is located in the external side of the plasma membrane.

31. ***Flnc*** **(filamin C, gamma):**

The gene product of *Flnc*, filamin Cg, enables ankyrin binding activity. It is involved in muscle cell development. Filamin Cg is located in the cytosol, the sarcolemma and sarcoplasm.

32. ***Fyb*** **(FYN binding protein):**

The protein encoded by this gene, FYN binding protein, is an adapter molecule that affects T cell receptor signaling and contains multiple PPI domains. It is thought to couple T cell receptor stimulation with activation of integrin function. Alternative splicing results in multiple transcript variants encoding different isoforms.

33. ***Gfap*** **(glial fibrillary acidic protein):**

This gene encodes for the glial fibrillary acidic protein that is a structural constituent of the cytoskeleton and involved in the regulation of chaperone-mediated autophagy. It exerts positive effects on D-aspartate import across the plasma membrane, gene expression, and intracellular protein transport. The glial fibrillary acidic protein acts upstream of or within several processes, including intermediate filament organization, long-term synaptic potentiation,

and neurogenesis. It is located in several cellular components, including astrocyte end-feet, cell bodies, and intermediate filaments.

34. ***Gpr84*** **(G protein-coupled receptor 84):**

Gpr84 encodes a protein that enables urotensin II receptor activity. It is involved in neuropeptide signaling pathways. The G protein-coupled receptor 84 is an integral component of the plasma membrane.

35. ***Havcr2*** **(hepatitis A virus cellular receptor 2):**

The gene product of *Havcr2* mediates metal ion binding activity. It is further involved in the regulation of cytokine production, the regulation of leukocyte activation, and the toll-like receptor signaling pathway. It is located on the cell surface, early endosomes and immunological synapses.

36. ***HcK*** **(HCK proto-oncogene, Src family tyrosine kinase):**

The protein encoded by this gene is a member of the Src family of tyrosine kinases. It is primarily hemopoietic, particularly in cells of the myeloid and B-lymphoid lineages. It may play a role in the innate immune response and the STAT5 signaling pathway. Alternative translation initiation site usage, including a non-AUG (CUG) codon, results in the production of two different isoforms, that have different subcellular localizations.

37. ***H2bc6*** **(H2B clustered histone 6):**

This gene encodes a replication-dependent histone that is a member of the histone H2B family and generates multiple transcripts through alternative splicing, the use of the conserved stem-loop termination motif, and the polyA addition motif.

38. ***Hvcn1* (hydrogen voltage-gated channel 1):**

Hvcn1 encodes a protein that has voltage-gated proton channel activity. It is involved in proton transmembrane transport and responds to pH and zinc ions. The H^+ voltage-gated channel 1 is located in the apical plasma membrane and is an integral component of the plasma membrane.

39. ***Icam1* (intercellular adhesion molecule 1):**

This gene encodes for the intercellular adhesion molecule 1, an integral membrane protein that binds leukocyte adhesion protein LFA-1. It participates in the innate immune response.

40. ***Ifi27l2a* (interferon, alpha-inducible protein 27 like 2A):**

The gene product of *Ifi27l2a* is engaged in aging processes and responses to virus. It is located in the mitochondrial membrane.

41. ***Ifitm3* (interferon induced transmembrane protein 3):**

Ifitm3 encodes a protein that is involved in the negative regulation of viral entry into host cell and responses to viruses. It plays an important role in the defense response to other organism, the negative regulation of cell population proliferation, and receptor-mediated endocytosis. The interferon induced transmembrane protein 3 is located in several cellular components, including apical parts of cells, the cell surface and the endoplasmic reticulum.

42. ***Igf1* (insulin-like growth factor 1):**

This gene encodes a member of the insulin-like growth factor (IGF) family of proteins that promotes growth and development during fetal and postnatal life. Transgenic disruption of this gene in mice results in reduced postnatal survival and severe growth retardation. Mice lacking the encoded protein exhibit generalized organ hypoplasia including underdevelopment of the central

nervous system and developmental defects in bone, muscle and reproductive systems. Alternative splicing results in multiple transcript variants encoding different isoforms that may undergo similar processing to generate mature protein.

43. ***Il4i1*** **(interleukin 4 induced 1):**

The gene product of *Il4i1* mediates L-amino-acid oxidase activity. It is involved in the negative regulation of T cell mediated immune response to tumor cells, the positive regulation of regulatory T cell differentiation, and the regulation of B cell differentiation. It acts upstream of or within aromatic amino acid family metabolic processes and is located in lysosomes.

44. ***Irf8*** **(interferon regulatory factor 8):**

The protein encoded by this gene is a transcription factor that belongs to the interferon regulatory factor family. Proteins belonging to this family have a DNA binding domain at the amino terminus that contains five well-conserved tryptophan-rich repeats. This domain recognizes DNA sequences similar to the interferon-stimulated response element. The interferon regulatory factor 8 promotes or suppresses lineage-specific genes to regulate the differentiation of lymphoid and myeloid lineage cells. Alternative splicing results in multiple transcript variants.

45. ***Irf9*** **(interferon regulatory factor 9):**

Irf9 encodes a protein that enables DNA-binding transcription factor activity, RNA polymerase II-specific and RNA polymerase II cis-regulatory region sequence-specific DNA binding activity. It is involved in immune system processes and the regulation of transcription by RNA polymerase II. The interferon regulatory factor 9 is located in the cytosol and active in the nucleus.

46. ***Itgb2*** **(integrin beta 2):**

Integrin beta2 encoded by *Itgb2* enables Aβ and complement component C3b binding activity. It contributes to cargo receptor activity and is involved in several processes, including cellular response to low-density lipoprotein particle stimulus, endocytosis, and the positive regulation of leukocyte adhesion to vascular endothelial cell. It further acts upstream of or within several processes, including activated T cell proliferation, integrin-mediated signaling pathway, and leukocyte migration. Integrin beta 2 is located in the external side of plasma membrane and membrane rafts and is part of the integrin alphaM-beta2 complex.

47. ***Laptm5*** **(lysosomal-associated transmembrane protein 5):**

The gene product of *Laptm5* has enzyme binding and protein sequestering activity. It is involved in several processes, including lysosomal transport, positive regulation of macromolecule metabolic process, and regulation of signal transduction. The lysosomal-associated transmembrane protein 5 is engaged in cellular response to leukemia inhibitory factor and is located in lysosomes.

48. ***Lag3*** **(lymphocyte-activation gene 3):**

Lag3 encodes a protein that enables MHC class II protein binding activity and transmembrane signaling receptor activity. It's involved in the negative regulation of regulatory T cell differentiation, plasmacytoid dendritic cell activation, and regulation of immune response. It's of central importance for cell surface receptor signaling pathways, the negative regulation of interleukin-2 production, and the positive regulation of natural killer cell mediated cytotoxicity. The lymphocyte-activation gene 3 gene product is located in the external side of the plasma membrane.

49. *Lgals3bp* **(galactoside-binding, soluble, 3 binding protein):**

The gene product of *Lgals3bp* enables scavenger receptor activity. It acts upstream of or within cell adhesion and is located in the extracellular space.

50. *Lcp1* **(lymphocyte cytosolic protein 1):**

Lcp1 encodes a protein that has actin filament binding activity. It is involved in actin filament bundle assembly and is located in several cellular components, phagocytic cups and ruffles.

51. *Ly86* **(lymphocyte antigen 86):**

The gene product of *Ly86* mediates positive regulation of lipopolysaccharide-mediated signaling pathways. The lymphocyte antigen 86 is located in the extracellular region.

52. *Lyz1* **(lysozyme 1):**

The gene product of *Lyz1* has lysozyme activity. It is involved in defense response to Gram-negative and Gram-positive bacteria. Lysozyme 1 is located in several cellular components, including Golgi cisterna, cytoplasmic vesicles, and the lumen of rough endoplasmic reticulum.

53. *Lyz2* **(lysozyme 2):**

Lyz2 encodes a protein that has lysozyme activity. It is involved in defense response to Gram-negative and Gram-positive bacteria. Lysozyme 2 is located in several cellular components, including Golgi cisterna, cytoplasmic vesicles, and the lumen of the rough endoplasmic reticulum.

54. *Mpeg1* **(macrophage expressed gene 1):**

The gene product encodes a protein that is involved in defense response to Gram-negative and Gram-positive bacteria. The macrophage expressed gene 1

can form a pore in the membrane of other organisms and is located in phagocytic vesicles.

55. ***Myo1f*** **(myosin IF):**

Myo1f codes for a protein with actin filament binding activity and microfilament motor activity. It acts upstream of or within several processes, including defense response to Gram-positive bacteria, neutrophil degranulation, and positive regulation of cell migration. Myosin IF is located in cortical actin cytoskeleton and part of filamentous actin.

56. ***Naip2*** **(NLR family, apoptosis inhibitory protein 2):**

Naip2 encodes a protein with ATP binding activity and cysteine-type endopeptidase inhibitor activity involved in apoptotic process. It is further engaged in inflammatory response, pyroptosis, and response to bacteria. The apoptosis inhibitory protein 2 (NLR family) is also related to cellular responses to estrogen stimuli and is part of the IPAF inflammasome complex.

57. ***Ncf2*** **(neutrophil cytosolic factor 2):**

The gene product of *Ncf2* has small GTPase binding activity. It contributes to superoxide-generating NAD(P)H oxidase activity and acts upstream of or within superoxide anion generation.

58. ***Oas1a*** **(2'-5' oligoadenylate synthetase 1A):**

The gene product of *Oas1a* has 2'-5'-oligoadenylate synthetase and double-stranded RNA binding activity. It is involved in purine nucleotide biosynthetic process and contributes to the negative regulation of viral process. It is located in mitochondria, the nucleus and ribosomes.

59. ***Phf11d*** **(PHD finger protein 11D):**

Phf11d encodes a protein with metal ion binding activity. It is located in nuclear membranes and the nucleoplasm.

60. ***Plek*** **(pleckstrin):**

The gene product of *Plek* exhibits phosphatidylinositol-3,4-bisphosphate and protein kinase C binding activity, and protein homodimerization capacity. It is further involved in exocytosis, platelet aggregation, and positive regulation of cellular component organization. Pleckstrin is located in the cytoplasm and ruffle membranes.

61. ***Prnp*** **(prion protein):**

The prion protein encoded by *Prnp* serves several functions, including Aβ and cupric ion binding activity. Furthermore, the prion protein serves as an aspartic-type endopeptidase inhibitor. It negatively regulates macromolecule metabolic processes, regulates protein localization to membranes and protein phosphorylation. In addition, prion protein exerts negative regulation of apoptotic processes, regulation of K^+ ion transmembrane transport, and response to oxidative stress. It is located in several cellular components, including Golgi apparatus, membrane rafts, and terminal boutons.

62. ***Psmb9*** **(proteasome 20S subunit beta 9):**

The gene product of *Psmb9* encodes a protein with proteasome binding activity. It acts upstream of or within antigen processing and presentation and is involved in responses to bacteria. It is part of a proteasome core complex.

63. *Psmb8* (proteasome 20S subunit beta 8):

The gene product of *Psmb8* exhibits endopeptidase activity. It is involved in fat cell differentiation and acts upstream of or within antigen processing and presentation. It's part of a proteasome core complex.

64. *Ptpn6* (protein tyrosine phosphatase non-receptor type 6):

The gene product of *Ptpn6* serves several functions, including phosphotyrosine residue and protein domain specific binding activity, and protein tyrosine phosphatase activity. It is involved in epididymis development, negative regulation of interleukin-6 production, and negative regulation of tumor necrosis factor production. It's further engaged in several processes, including hemopoiesis, negative regulation of protein phosphorylation, and regulation of lymphocyte activation. The protein tyrosine phosphatase non-receptor type 6 is located in cell-cell junctions and part of the alpha-beta T cell receptor complex.

65. *Ptprc* (protein tyrosine phosphatase, receptor type, C):

Ptprc encodes for a protein that can bind heparan sulfate proteoglycans and heparin and exerts protein tyrosine phosphatase activity. It is involved in the regulation of protein phosphorylation, lymphocyte differentiation and activation, positive regulation of macromolecule metabolic processes and regulation of signal transduction. It is located in the external side of the plasma membrane.

66. *Pycard* (PYD and CARD domain containing):

The gene product of *Pycard* is involved in several processes, including activation of cysteine-type endopeptidase activity, positive regulation of cytokine production, and regulation of defense response. It's further involved in defense response to Gram-positive bacteria, the positive regulation of macromolecule

metabolic processes, and the regulation of autophagy. It's located in the cytosol and nucleus and part of inflammasome complexes.

67. *Rac2* (Rac family small GTPase 2):

The gene product of *Rac2* encodes a protein with GTPase activity and protein kinase regulator activity. It's involved in the regulation of leukocyte activation and leukocyte chemotaxis. It is further engaged in G protein-coupled receptor signaling pathways, actin cytoskeleton organization, and cell projection assembly. The Rac family small GTPase 2 is located in the cytoplasm, membrane, and nuclear envelope.

68. *Rab7b* (RAB7B, member RAS oncogene family):

Rab7b encodes a protein with GTP binding activity and GTPase activity. It is involved in the negative regulation of the toll-like receptor 4 and the toll-like receptor 9 signaling pathways. It plays a further role in cellular responses to interferon-gamma and is located in late endosomes, lysosomes, and phagocytic vesicles.

69. *Rhoh* (Ras homolog family member H):

The gene product of *Rhoh* has GTP binding activity and further serves as a GTPase and kinase inhibitor. It's involved in T cell differentiation and mast cell activation. The Ras homolog family member H is located in the cytoplasm and synapses.

70. *Samsn1* (SAM domain, SH3 domain and nuclear localization signals):

The gene product of *Samsn1* has phosphotyrosine residue binding activity. It is involved in the negative regulation of B cell activation, negative regulation of adaptive immune response and negative regulation of peptidyl-tyrosine phosphorylation. It is located in both the cytosol and nucleus.

71. *Sgk1* **(serum/glucocorticoid regulated kinase 1):**

This gene encodes a serine/threonine protein kinase that plays an important role in cellular stress response. It activates certain K^+, Na^+, and Cl^- channels, suggesting an involvement in the regulation of processes such as cell survival, neuronal excitability, and renal Na+ excretion. The serum/glucocorticoid regulated kinase 1 is activated by protein phosphorylation and degraded via the ubiquitination and proteasome pathway. Multiple transcript variants encoding different isoforms have been described for this gene.

72. *Siglech* **(sialic acid binding Ig-like lectin H):**

The gene product of *Siglech* has cargo receptor activity and acts upstream of or within receptor-mediated endocytosis. It is located in the cell surface.

73. *Slamf9* **(SLAM family member 9):**

This gene product of *Slamf9*, SLAM family member 9, acts upstream of or within defense response to bacteria, plasmacytoid dendritic cell chemotaxis, and plasmacytoid dendritic cell differentiation. It is located on the cell surface.

74. *Slc11a1* **(solute carrier family 11, proton-coupled divalent metal ion transporters):**

This gene product has manganese ion transmembrane transporter activity. It is involved in metal ion export. It’s further involved in cation homeostasis, defense response to other organisms, and positive regulation of macromolecule metabolic processes. It is located in cytoplasmic vesicles and lysosomes.

75. *Slc38a5* **(solute carrier family 38, member 5):**

The encoded protein has amino acid transmembrane transporter activity and is located in the plasma membrane.

76. ***St8sia6*** **(ST8 alpha-N-acetyl-neuraminide alpha-2,8-sialyltransferase 6):**

The gene product has sialyltransferase activity and is involved in ganglioside biosynthetic processes, glycoprotein and oligosaccharide metabolic processes. It's engaged in blastocyst hatching, glycolipid biosynthetic processes, and protein O-linked glycosylation and is an integral component of membrane.

77. ***Sp110*** **(SP110 nuclear body protein):**

The gene product of *Sp110* has DNA-binding transcription factor activity. It's involved in the positive regulation of apoptotic process and response to bacteria. It's located in the nucleoplasm.

78. ***Syngr1*** **(synaptogyrin 1):**

The gene product of *Syngr1* is involved in the regulation of long-term neuronal synaptic plasticity and the regulation of short-term neuronal synaptic plasticity. Synaptogyrin 1 acts upstream of or within cellular responses to leukemia inhibitory factor. It's located in synaptic vesicles.

79. ***Synpo2*** **(synaptopodin 2):**

The gene product of *Synpo2* has 14-3-3 protein and muscle alpha-actinin binding activity. It plays a role in chaperone-mediated autophagy, positive regulation of actin filament bundle assembly, and the regulation of Rho-dependent protein serine/threonine kinase activity. It's located in the nucleus.

80. ***Tbxas1*** **(thromboxane A synthase 1):**

The gene encodes a protein with thromboxane-A synthase activity. It's involved in cellular Cl^- ion homeostasis, positive regulation of vasoconstriction, and prostaglandin biosynthetic processes. Furthermore, thromboxane A synthase 1

plays an important role in fatty acid biosynthetic processes and is located in the endoplasmic reticulum membrane.

81. ***Tlr7* (toll-like receptor 7):**

The gene product exerts siRNA and single-stranded RNA binding activity. It is involved the positive regulation of macromolecule metabolic processes, response to viruses, and toll-like receptor 7 signaling pathways. The toll-like receptor 7 is further engaged in the positive regulation of interleukin-6 production and the regulation of protein phosphorylation. It's located in cytoplasmic vesicles, the endoplasmic reticulum, and lysosomes.

82. ***Trem2* (triggering receptor expressed on myeloid cells 2):**

The protein encoded by this gene is part of the immunoglobulin and lectin-like superfamily. It's associated with the adaptor protein Dap-12 and recruits several factors, such as kinases and PLCg, to form a receptor signaling complex that activates myeloid cells, including dendritic cells and microglia. In humans, mutations in this gene may be risk factors to the development of AD.

83. ***Tubb6* (tubulin beta 6 class V):**

The protein encoded by this gene has GTP binding activity. Structurally, it's a constituent of the cytoskeleton. It is involved in microtubule cytoskeleton organization and mitotic cell cycle. The tubulin beta 6 class V is active in the cytoplasm and microtubules.

84. ***Tyrobp* (TYRO protein tyrosine kinase binding protein):**

This gene product has signaling receptor binding activity and is involved in e.g., myeloid cell activation, regulation of cytokine production, and the regulation of lymphocyte activation. It's located in the cell surface.

85. ***Uba7*** **(ubiquitin like modifier activating enzyme 7):**

The gene product of *Uba7* has ISG15 activating enzyme activity. It's involved in Interferon-stimulated gene 15 (ISG15)-protein conjugation and modification-dependent protein catabolic processes. It's active in the cytoplasm and nucleus.

86. ***Vav1*** **(Vav 1 guanine nucleotide exchange factor):**

Vav1 encodes a protein with guanyl-nucleotide exchange factor (GEF) activity and phosphorylation-dependent protein binding activity. It's involved in integrin-mediated signaling pathways, neutrophil chemotaxis, and the positive regulation of natural killer cell mediated cytotoxicity. It's located in cell-cell junctions.

87. ***Vim*** **(vimentin):**

This gene encodes vimentin, a protein with RNA binding activity. It's involved in intermediate filament organization and the positive regulation of gene expression. It's further engaged in SMAD protein signal transduction, astrocyte differentiation, and cellular responses to interferon-gamma. Vimentin is located in several cellular components, including cell leading edge, intermediate filaments, and phagocytic vesicles.

Downregulated DEGs (top 30 from the individual subgroups, FC < 1.5)

1. ***Arpp21* (cyclic AMP-regulated phosphoprotein 21):**
   The gene product of *Arpp21* enables calmodulin and nucleic acid binding activity. It's involved in cellular response to heat and located in the cytoplasm.

2. ***Asic4* (acid-sensing, proton-gated ion channel family member 4):**
   This gene enables ligand-gated Na^+ channel activity. It plays a central role in behavioral fear response and is predicted to be located in the plasma membrane.

3. ***Ccdc184* (coiled-coil domain containing 184):**
   The gene product is predicted to be active in the cytoplasm.

4. ***Ctr9* (CTR9 homolog, Paf1/RNA polymerase II complex component):**
   This gene encodes a protein with SH2 domain binding activity. It is involved in cell surface receptor signaling pathways, histone H3-K4 trimethylation, and the positive regulation of histone H3-K4 methylation. It plays further roles in blastocyst development and the regulation of genetic imprinting. It's located in the nucleus and transcriptionally active chromatin and forms part of the Cdc73/Paf1 complex.

5. ***Etnppl* (ethanolamine phosphate phospholyase):**
   The gene encodes ethanolamine-phosphate phospholyase. It's located in mitochondria.

6. ***Gpr1* (G protein-coupled receptor 1):**
   The gene product of *Gpr1* exhibits G protein-coupled receptor and glucose binding activity. G protein-coupled receptor 1 is involved in the detection of

carbohydrate stimuli, the invasive growth in response to glucose limitation, and sugar mediated signaling pathways. It's located in the plasma membrane.

7. ***Hdac9* (histone deacetylase 9):**

   Hdac9 encodes for histone deacetylase 9 that has DNA-binding transcription factor binding activity and transcription corepressor activity. It is involved in the determination of adult lifespan, the negative regulation of transcription by RNA polymerase II, and the regulation of skeletal muscle fiber development. Furthermore, it forms part of the histone methyltransferase complex.

8. ***Hlcs* (holocarboxylase synthetase (biotin- [propriony-Coenzyme A-carboxylase (ATP-hydrolysing)] ligase):**

   The gene product is predicted to enable biotin binding activity, biotin-protein ligase activity, and enzyme binding activity. It's further involved in histone biotinylation and located in mitochondria.

9. ***Myh7b* (myosin, heavy chain 7B, cardiac muscle, beta):**

   This gene encodes a myosin heavy chain. The encoded protein forms a hexamer comprised of two heavy chains, two alkali light chains, and two regulatory light chain components.

10. ***Myh8* (myosin, heavy polypeptide 8, skeletal muscle, perinatal):**

    This gene encodes a myosin heavy chain. The encoded protein forms a hexamer with two heavy chains, two alkali light chains, and two regulatory light chain components. This complex functions in muscle contraction.

11. ***Pcsk1* (proprotein convertase subtilisin/kexin type 1):**

The gene product exerts serine-type endopeptidase activity. It is involved in peptide biosynthetic processes and peptide hormone processing. It's located in the extracellular space.

12. ***Pdzph1* (PDZ and pleckstrin homology domains 1):**

The gene product is orthologous to the human PDZPH1P (PDZ and pleckstrin homology domains 1, pseudogene).

13. ***Pisd-ps3* (phosphatidylserine decarboxylase, pseudogene 3):**

The phosphatidylserine decarboxylase is expressed in the nervous system and olfactory epithelium.

14. ***Pla2g4e* (phospholipase A2, group IVE):**

The phospholipase A2 encoded by *Pla2g4e* has phosphatidylinositol phosphate, phosphatidylinositol-3,4-bisphosphate and phospholipase A2 activity. It's involved in N-acylphosphatidylethanolamine metabolic processes and the positive regulation of endocytic recycling. Phospholipase A2 is located in early endosome membrane, lysosomal membranes and the plasma membrane.

15. ***Prr16* (BY588929 RIKEN full-length enriched, adult inner ear mus musculus cDNA clone F930004G14 3'):**

The gene product is involved in positive regulation of cell size.

16. ***Ptpn6* (protein tyrosine phosphatase, non-receptor type 6):**

The protein tyrosine phosphatase (non-receptor type 6) is involved in the negative regulation of interleukin-6 production and the negative regulation of tumor necrosis factor production. It is engaged in hemopoiesis, negative regulation of protein phosphorylation, and the regulation of lymphocyte

activation. It's located in cell-cell junction and part of the alpha-beta T cell receptor complex.

17. *Rims3* (regulating synaptic membrane exocytosis 3):

The gene product of *Rims3* enables transmembrane transporter binding activity. It is involved in the regulation of the membrane potential and located in membranes and synapses (cytoskeleton of presynaptic active zone and presynaptic membranes).

18. *Scyl2* (SCY1-like 2):

The SCY1-like 2 protein is involved in pyramidal neuron development and located in the Golgi apparatus, endosomes and membranes.

19. *Shisa9* (shisa family member 9):

The shisa family member 9 is involved in the regulation of AMPA receptor activity, the regulation of postsynaptic neurotransmitter receptor activity, and regulation of short-term neuronal synaptic plasticity. It's located in glutamatergic synapses, postsynaptic densities, and synaptic membranes.

20. *Spag5* (sperm associated antigen 5):

The gene *Spag5* encodes sperm associated antigen 5 with microtubule binding activity. It's involved in the establishment of spindle orientation and located in ciliary basal bodies, ciliary rootlets, and mitotic spindles.

21. *Trim66* (tripartite motif-containing 66):

The gene product of *Trim66* enables chromatin and identical protein binding activity. It is engaged in the negative regulation of transcription (DNA-templated) and located in the chromocenter and nucleus.

22. ***Wnt9a*** **(wingless-type MMTV integration site family, member 9A):**

The gene product enables cytokine activity and frizzled binding activity. It plays a role in embryonic morphogenesis, the negative regulation of chondrocyte differentiation and the positive regulation of smoothened signaling pathway. It is located in extracellular region.
