# Supplementary material for: Sex- and region-specific cortical and hippocampal whole genome transcriptome profiles from control and APP/PS1 Alzheimer’s disease mice
Source: PLoS One. 2024 Feb 7;19(2):e0296959. doi: 10.1371/journal.pone.0296959 (PMC10849391; doi:10.1371/journal.pone.0296959)
Supplement: S1 File — S1 Fig: Genotyping of APP/PS1 AD mice and WT control animals. S2 Fig: 3D image of the murine brain including the RS cortex and hippocampus (BROIs) used for transcriptome analysis in our study. S3 Fig: PCA of transcriptomes from the RS cortex and hippocampus of WT controls and APP/PS1 AD mice of both sexes. S4 Fig: Hierarchical clustering of transcriptome data from the RS cortex and hippocampus of WT control and APP/PS1 AD mice of both sexes. S5 Fig: Bar diagrams of the top 30 candidates of DEGs with highest significant FCs (FC > 1.5 and FC < -1.5, p < 0.05). S6 Fig: Pathway analysis of intersectional and signature gene sets in APP/PS1 subgroups. S7 Fig: Comparative qPCR analysis of selected gene transcript levels from the hippocampus of female and male APP/PS1 AD with 5XFAD mice. S1 Table: PCR reaction set-up using PCR Mastermix and genomic DNA. S2 Table: Materials used for one-color microarray-based gene expression data collection. S3 Table: Software used for one-color microarray-based gene expression data collection. S4 Table: Details on genes, forward and reverse primer sequences and annealing temperatures relevant for qPCR experimentation. S5 Table: Characteristics of DEGs in the RS cortex of female APP/PS1 AD mice. S6 Table: Characteristics of DEGs in the hippocampus of female APP/PS1 AD mice. S7 Table: Characteristics of DEGs in the RS cortex of male APP/PS1 AD mice. S8 Table: Characteristics of DEGs in the hippocampus of male APP/PS1 AD mice. S9 Table: Venn analysis of DEGs in the RS cortex and hippocampus of female APP/PS1 AD mice. S10 Table: Venn analysis of DEGs genes in the RS cortex and hippocampus of male APP/PS1 AD mice. S11 Table: Venn analysis of DEGs in the RS cortex of male and female APP/PS1 AD mice. S12 Table: Venn analysis of DEGs in the hippocampus of male and female APP/PS1 AD mice. S13 Table: Differentially regulated l(i)ncRNAs in APP/PS1 AD vs. WT mice. S14 Table: qPCR-based FC analysis of selected genes in the hippocampus of APP/PS1 AD vs. [file pone.0296959.s001.zip › Supplementary Files_R1/Supplementary Table 2_Materials_Microarrays.pdf]

**Supplementary Table 2: Material (kits) used for One-Color Microarray-Based Gene Expression data collection**

| Kit | Catalog number | Company |
| --- | --- | --- |
| Agilent RNA 6000 Nano Kit | 5067-1511 | Agilent Technologies Germany GmbH & Co. KG, Waldbronn, Germany |
| Gene Expression Hybridization Kit | 5188-5242 | Agilent Technologies Germany GmbH & Co. KG, Waldbronn, Germany |
| Gene Expression Wash Buffer Kit | 5188-5327 | Agilent Technologies Germany GmbH & Co. KG, Waldbronn, Germany |
| Hybridization Gasket Slide Kit (20) | G2534-60015 | Agilent Technologies Germany GmbH & Co. KG, Waldbronn, Germany |
| Low Input Quick Amp Labeling Kit, One-Color | 5190-2305 | Agilent Technologies Germany GmbH & Co. KG, Waldbronn, Germany |
| One-Color RNA Spike-In Kit | 5188-5282 | Agilent Technologies Germany GmbH & Co. KG, Waldbronn, Germany |
| RNase-free DNase Set (50) | 79254 | Qiagen GmbH, Hilden, Germany |
| RNeasy Lipid Tissue Mini Kit (50) | 74804 | Qiagen GmbH, Hilden, Germany |
| RNeasy Mini Kit | 74104 | Qiagen GmbH, Hilden, Germany |
| SurePrint G3 Mouse Gene Expression v2 8x60K Microarray Kit | G4852B | Agilent Technologies Germany GmbH & Co. KG, Waldbronn, Germany |
