# Supplementary material for: Sex- and region-specific cortical and hippocampal whole genome transcriptome profiles from control and APP/PS1 Alzheimer’s disease mice
Source: PLoS One. 2024 Feb 7;19(2):e0296959. doi: 10.1371/journal.pone.0296959 (PMC10849391; doi:10.1371/journal.pone.0296959)
Supplement: S1 File — S1 Fig: Genotyping of APP/PS1 AD mice and WT control animals. S2 Fig: 3D image of the murine brain including the RS cortex and hippocampus (BROIs) used for transcriptome analysis in our study. S3 Fig: PCA of transcriptomes from the RS cortex and hippocampus of WT controls and APP/PS1 AD mice of both sexes. S4 Fig: Hierarchical clustering of transcriptome data from the RS cortex and hippocampus of WT control and APP/PS1 AD mice of both sexes. S5 Fig: Bar diagrams of the top 30 candidates of DEGs with highest significant FCs (FC > 1.5 and FC < -1.5, p < 0.05). S6 Fig: Pathway analysis of intersectional and signature gene sets in APP/PS1 subgroups. S7 Fig: Comparative qPCR analysis of selected gene transcript levels from the hippocampus of female and male APP/PS1 AD with 5XFAD mice. S1 Table: PCR reaction set-up using PCR Mastermix and genomic DNA. S2 Table: Materials used for one-color microarray-based gene expression data collection. S3 Table: Software used for one-color microarray-based gene expression data collection. S4 Table: Details on genes, forward and reverse primer sequences and annealing temperatures relevant for qPCR experimentation. S5 Table: Characteristics of DEGs in the RS cortex of female APP/PS1 AD mice. S6 Table: Characteristics of DEGs in the hippocampus of female APP/PS1 AD mice. S7 Table: Characteristics of DEGs in the RS cortex of male APP/PS1 AD mice. S8 Table: Characteristics of DEGs in the hippocampus of male APP/PS1 AD mice. S9 Table: Venn analysis of DEGs in the RS cortex and hippocampus of female APP/PS1 AD mice. S10 Table: Venn analysis of DEGs genes in the RS cortex and hippocampus of male APP/PS1 AD mice. S11 Table: Venn analysis of DEGs in the RS cortex of male and female APP/PS1 AD mice. S12 Table: Venn analysis of DEGs in the hippocampus of male and female APP/PS1 AD mice. S13 Table: Differentially regulated l(i)ncRNAs in APP/PS1 AD vs. WT mice. S14 Table: qPCR-based FC analysis of selected genes in the hippocampus of APP/PS1 AD vs. [file pone.0296959.s001.zip › Supplementary Files_R1/Supplementary Table 4_Primers_qPCR.pdf]

Supplementary Table 4: Details on genes, forward and reverse primer sequences and primer concentrations relevant for qPCR experimentation.

Gene	Forward primer sequence (5'-3')	Reverse primer sequence (5'-3')	Primer concentration (μM)	Primer source
Cacna1d	CTACCGTTGCACAGATGAAGCC	TCACGGACCACAGGACTGTCAA	0.125	OriGene (https://www.origene.com/)
Cacna1c	CGTTCTCATCCTGCTCAACACC	GAGCTTCAGGATCATCTCCACTG	0.125	OriGene (https://www.origene.com/)
Plcd4	TCTCGCGCAATATGCCTTCCAG	ATCTCGGTCAGATGGTGTGCCA	0.25	OriGene (https://www.origene.com/)
Casp8	ATGGCTACGGTGAAGAACTGCG	TAGTTCACGCCAGTCAGGATGC	0.25	OriGene (https://www.origene.com/)
Chrm1	GCACAGGCACCCACCAAGCAG	AGAGCAGCAGCAGGCGGAACG	0.25	Wei J et al., 1994
Chrm3	GTCTGGCTTGGGTCATCTCCT	GCTGCTGCTGTGGTCTTGGTC	0.25	Wei J et al., 1994
Chrm5	TGGTCATCCTCCCGTAGAAGCA	GCTACAGTTGGTAACCTGCTCAG	0.25	OriGene (https://www.origene.com/)
Siglech	TGGAACCAACCTCACCTGTCAG	CCAACTCTTCCAGAGACATGGG	0.25	OriGene (https://www.origene.com/)
Ptpn6	TTGGCAGGAGAACACTCGTGTC	TGCTCCCTACTGTTGGTCACAG	0.5	OriGene (https://www.origene.com/)
Laptm5	GCGGTAAAGTGTCCTGTAGGTTC	TCTTGACCACGCCGAACAGCAG	0.5	OriGene (https://www.origene.com/)
Plek	GGAGCAGTTCACTTGAGAGGCT	TGGAAGTGGCTGCCTGCAAGTA	0.25	OriGene (https://www.origene.com/)

Arpp21	GGAGTCAGCAAATACCACAGACC	CTCCTTGCTGACTGCTCATCAC	0.25	OriGene (https://www.origene.com/)
Shisa9	CTGCAACACTGACCACATGGAG	ATCTGCTGGAGCAGAGGAGAAG	0.5	OriGene (https://www.origene.com/)
Hprt	GCTGGTGAAAAGGACCTCT	CACAGGACTAGAACACCTGC	0.25	Weiergräber M et al., 2005

References

J Wei, E A Walton, A Milici, J J Buccafusco. m1-m5 muscarinic receptor distribution in rat CNS by RT-PCR and HPLC. J Neurochem, 1994 Sep;63(3):815-21. doi: 10.1046/j.1471-4159.1994.63030815.x.

Marco Weiergräber, Margit Henry, Michael Südkamp, Ernst-Rainer de Vivie, Jürgen Hescheler, Toni Schneider. Ablation of $Ca_v2.3$ / E-type voltage-gated calcium channel results in cardiac arrhythmia and altered autonomic control within the murine cardiovascular system. Basic Res Cardiol, 2005 Jan;100(1):1-13. doi: 10.1007/s00395-004-0488-1. Epub 2004 Oct 20.
