# Supplementary material for: Sex- and region-specific cortical and hippocampal whole genome transcriptome profiles from control and APP/PS1 Alzheimer’s disease mice
Source: PLoS One. 2024 Feb 7;19(2):e0296959. doi: 10.1371/journal.pone.0296959 (PMC10849391; doi:10.1371/journal.pone.0296959)
Supplement: S1 File — S1 Fig: Genotyping of APP/PS1 AD mice and WT control animals. S2 Fig: 3D image of the murine brain including the RS cortex and hippocampus (BROIs) used for transcriptome analysis in our study. S3 Fig: PCA of transcriptomes from the RS cortex and hippocampus of WT controls and APP/PS1 AD mice of both sexes. S4 Fig: Hierarchical clustering of transcriptome data from the RS cortex and hippocampus of WT control and APP/PS1 AD mice of both sexes. S5 Fig: Bar diagrams of the top 30 candidates of DEGs with highest significant FCs (FC > 1.5 and FC < -1.5, p < 0.05). S6 Fig: Pathway analysis of intersectional and signature gene sets in APP/PS1 subgroups. S7 Fig: Comparative qPCR analysis of selected gene transcript levels from the hippocampus of female and male APP/PS1 AD with 5XFAD mice. S1 Table: PCR reaction set-up using PCR Mastermix and genomic DNA. S2 Table: Materials used for one-color microarray-based gene expression data collection. S3 Table: Software used for one-color microarray-based gene expression data collection. S4 Table: Details on genes, forward and reverse primer sequences and annealing temperatures relevant for qPCR experimentation. S5 Table: Characteristics of DEGs in the RS cortex of female APP/PS1 AD mice. S6 Table: Characteristics of DEGs in the hippocampus of female APP/PS1 AD mice. S7 Table: Characteristics of DEGs in the RS cortex of male APP/PS1 AD mice. S8 Table: Characteristics of DEGs in the hippocampus of male APP/PS1 AD mice. S9 Table: Venn analysis of DEGs in the RS cortex and hippocampus of female APP/PS1 AD mice. S10 Table: Venn analysis of DEGs genes in the RS cortex and hippocampus of male APP/PS1 AD mice. S11 Table: Venn analysis of DEGs in the RS cortex of male and female APP/PS1 AD mice. S12 Table: Venn analysis of DEGs in the hippocampus of male and female APP/PS1 AD mice. S13 Table: Differentially regulated l(i)ncRNAs in APP/PS1 AD vs. WT mice. S14 Table: qPCR-based FC analysis of selected genes in the hippocampus of APP/PS1 AD vs. [file pone.0296959.s001.zip › Supplementary Files_R1/Supplementary Table_13_lincRNA_APP_details.pdf]

Supplementary Table 13: A) Differentially regulated l(i)ncRNAs in the RS cortex of female APP/PS1 mice. This table characterizes the individual, differentially regulated l(i)ncRNAs, their fold changes (FC) and related p-values and the related frequencies. Downregulated l(i)ncRNAs are depicted in light green, upregulated l(i)ncRNAs are highlighted in light yellow. Analysis of l(i)ncRNAs for all subgroups was performed using RNAcentral („https://rnacentral.org/“), Rfam (Rfam.org), Coding-Potential Assessment Tool (CPAT) and NONCODE (“http://www.noncode.org/”).

**Description**	**FC**	**P-value**	**Sequence**
l(i)ncRNA:chr14:73729977-73751877 reverse strand	-1.21	0.012	GGGCTCCTCTGTTCCACCCAGGGTGTGGAAGTGCCTTCATCCATTCTTGTATTACTATTT (Rfam database neg, no GO annotations, annotated by 1 database (NONCODE), expression in the hippocampus reported.)
l(i)ncRNA:chr6:116123643-116124550 forward strand	1.126	0.041	TTCCCTGTGGGCCGTATTCATCGACACCTGAAATCTAGGACAACCAGCCACGGACGTGTG (Rfam database neg, no GO annotations, annotated by 1 database (NONCODE), expression in the hippocampus reported.)

Supplementary Table 13: B) Differentially regulated l(i)ncRNAs in the hippocampus of female APP/PS1 mice.

Description	FC	P-value	Sequence
l(i)ncRNA:chr10:120336296-120354471 reverse strand	-1.46	0.006	TTACATTCATTGTCTTGAGAGAGGGTCATTTGGATCTGATTAATTGTGGAGTTTAGTGCC (Rfam database neg, no GO annotations, annotated by 1 database (NONCODE), strong expression in hippocampus reported).
l(i)ncRNA:chr4:129376440-129391690 reverse strand	-1.33	2.66E-04	TTTTGGTAAGTTGACGTTTTCTGGGCCTTGGTTTTAACATCTCTTGGTGTTGGAGGAATC (Rfam database neg, no GO annotations, annotated by 1 database (NONCODE), expression in hippocampus reported.)
l(i)ncRNA:chr16:4525147-4542122 forward strand	-1.33	0.014	AGAATATATTGCAAACTTATTCCCATCTTTCGCTCTGCCTGCATTAGCAGCGCGTGGCTT (No entry in RNAcentral for mus musculus)
l(i)ncRNA:chr13:66381200-66410200 forward strand	-1.30	0.002	TTAGTCCCCCACTCTTCTGTCCAGGTGCTAACAGCCCATTATCTCAAGAGCTGTTTTCTA (Rfam database neg, no GO annotations, annotated by 1 database (NONCODE), expression in hippocampus reported.)
l(i)ncRNA:chr9:20784567-20799942 reverse strand	-1,23	0,039	TAAGGACCTGTGTGAGGACTCCTCTCCTGTCCCTGGCAACACTGGGGAGGGACCCTAGCT (Rfam database neg, no GO annotations, annotated by 1 database (NONCODE), no expression in hippocampus reported).
l(i)ncRNA:chr1:134867906-134885481 forward strand	-1.22	0.006	TCAAGTGTCACAACACAATGCTAATCTTCAGTAAAGCGTTTGCCAAGCTCTAAAAACCAC (Rfam database neg, no GO annotations, annotated by 1 database (NONCODE), no expression in hippocampus reported).
l(i)ncRNA:chr9:30805020-30810645 reverse strand	-1.22	2.59E-04	TGGTTTTGGTAACTGTTAAGACCGCTCCCTGCTCAGTTAACCTGTACTCTGTATACGAAG (Rfam database neg, no GO annotations, annotated by 3 databases (Ensembl/GENCODE, Ensembl, MGI), expression in nervous system reported).
l(i)ncRNA:chr17:8025086-8026227 reverse strand	-1.21	0.012	TAGAAATACCTGAAGGAGAAATGAAAGAGTCCTTGCCTTGATGCCTCCCAGACAACACTG (Rfam database neg, no GO annotations, annotated by 1 database (NONCODE), no expression in the hippocampus reported).
l(i)ncRNA:chr6:11994002-11997859 forward strand	-1.21	0.029	ACAGGAAGATCAATCATTTTCTCTTCTTCTCTAGGGTGCTGTACTTAATCATAACGCTAA (Rfam database neg, no GO annotations, annotated by 1 database (NONCODE), expression in the hippocampus reported).
l(i)ncRNA:chr9:30529709-30589042 reverse strand	-1.19	0.028	AATGGCTCATGCCAGCCCTGCCTCTGTCCACACATACCGTATCTTAGCTCAATGTATTCT (Rfam database neg, no GO annotations, annotated by 1 database (NONCODE): expression in the hippocampus reported).

l(i)ncRNA:chr2:173115795-173116608 reverse strand	-1.18	0.024	CCAGCATGCCTTATGCCACAAAGGGAGATCTGTCCGTCTGTCCCTGCAATGCTCGGAAAA (Rfam database neg, no GO annotations, annotated by 1 database (NONCODE), no information about CNS expression available).
l(i)ncRNA:chr17:36131460-36136694 forward strand	-1.18	0.0200	CCTCCTGCTGTACCTTAACTGCTTTGTTTGCTTAAATAAGAATTAGGGAAACAGATTATT (Rfam database neg, no GO annotations, annotated by 1 database (NONCODE), expression in the hippocampus reported).
l(i)ncRNA:chr5:22928221-22939127 reverse strand	1.25	0.038	TGACCCTTAGAGTTTTAGATGTGCGGTTCCCTTCATGTATACTTTACAGAAAACCAGCTA (Rfam database neg, no GO annotations, annotated by 1 database (NONCODE), no expression data in CNS reported).
l(i)ncRNA:chr8:122920901-123008463 forward strand	1.74	0.020	CATTTGGTTCCCATTGCAAAGAAGAGAAGACAGAGGCTAGGTGGGAACTCATAGCAAGTT (Rfam database neg, no GO annotations, annotated by 5 databases (Ensembl/GENCODE, Ensembl, RefSeq, NONCODE, MGI), expression in hippocampus reported, involvement in in epigenetic effects (Huo Y et al., 2020).

Supplementary Table 13: C) Differentially regulated l(i)ncRNAs in the RS cortex of male APP/PS1 mice.

Description	FC	P-value	Sequence
l(i)ncRNA:chr12:85252300-85253597 reverse strand	-1.47	0.021	GAGACCCTTCCACACCTATATACAAAAATTACCTCAACTCAGGTCATAAATGGTAAAAAA (Rfam database neg, no GO annotations, annotated by 1 database (NONCODE), no information about CNS expression available).
l(i)ncRNA:chr14:62294849-62301165 reverse strand	-1.38	0.026	AAACCAATAACATGAAAGGAAACCGGAAGTGGGACCATCTAGTTCTTGATGACTCAAAGT (Rfam database neg, no GO annotations, annotated by 1 database (NONCODE), no information about CNS expression available).
l(i)ncRNA:chr13:107822042-107823753 forward strand	-1.32	0.020	GGACACCAATAAAAAGTCAAAGGACCAAAATGGATGCATTGAGTATCTTAAAACTTGATC (Rfam database neg, no GO annotations, annotated by 1 database (NONCODE), no information about CNS expression available).
l(i)ncRNA:chr13:107812547-107823601 forward strand	-1.30	0.027	CCAGCATGACAGAAATGTCAGGAGATGTCTGGAATTAAGAGAATTACAAAGAGAAGGGGG (Rfam database neg, no GO annotations, annotated by 1 database (NONCODE), no information about CNS expression available).
l(i)ncRNA:chrX:102568332-102587093 reverse strand	-1.29	0.039	CAAAGTCAGCTCATAGTGGAGAAATACAACAAGCACAAGACTAAGGTTAACATCATTGGG (Rfam database neg, no GO annotations, annotated by 1 database (NONCODE), no information about CNS expression available).
l(i)ncRNA:chrX:102567595-102589870 reverse strand	-1.29	0.047	TTGGAGTAACAATGGCGGGTCAGTGTTGTTGCCTATCATATTTCAAAAGATTTATGAACT (Rfam database neg, no GO annotations, annotated by 1 database (NONCODE), no information about CNS expression available).
l(i)ncRNA:chr18:84760623-84761084 forward strand	-1.28	0.020	ACACAAGGAGGCCATTTCCTGGTGAGAAACTTCAATGTCAACAAAAAGGAATACAGGGAT (Rfam database neg, no GO annotations, annotated by 2 databases (Ensembl/GENCODE, Ensembl), no information about CNS expression available).
l(i)ncRNA:chr2:67411703-67412293 forward strand	-1.24	0.032	TAGGTCTTTTCCGGGTCCTGTCACCAATGCTAGGAATCAGCATCAATTTCTCTCAGTCTT (Rfam database neg, no GO annotations, annotated by 1 database (NONCODE), expressed in the hippocampus).
l(i)ncRNA:chr2:48818825-48822612 forward strand	-1.20	0.040	TAGACCATTGCCCAGAATGACTGAGACCCTTGGTTTAATCTACAAAAGTACAAGAAAAAA (Rfam database neg, no GO annotations, annotated by 1 database (NONCODE), expressed in the hippocampus).
l(i)ncRNA:chr4:135138014-135174889 forward strand	1.20	0.037	TCACCACGTTACACAAGGCTCCTTATCTCTGGATTCCCTGTGCCCTACTCCTGGAGCTGA (Rfam database neg, no GO annotations, annotated by 1 database (NONCODE)).
L(i)ncRNA:chr16:11008534-11013759 forward strand	1.21	0.033	TCTTGGCCCTCCACACCCACGCTTTAGGGAAGATCAGATATTTCTTCTTGTACTAACTGT

			(Rfam database neg, no GO annotations, annotated by 1 database (NONCODE), expressed in the hippocampus).
l(i)ncRNA:chrX:50087925-50101700 forward strand	1.22	0.040	AATTAGGAAATGGAACCCCCACACCGCCCAGGCCAAAGCCCACGGTGCCCCAGTGACACA (Rfam database neg, no GO annotations, annotated by 1 database (NONCODE)).
l(i)ncRNA:chr2:152172745-152184745 reverse strand	1.43	0.044	ATCACGGCGGTGCGTCAGGGATTGCCACGCGGGGTTTAAAGACGATGTCACTCCAACGAG (Rfam database neg, no GO annotations, annotated by 1 database (NONCODE), expressed in the hippocampus).

Supplementary Table 13: D) Differentially regulated l(i)ncRNAs in the hippocampus of male APP/PS1 mice.

Description	FC	P value	Sequence
l(i)ncRNA:chr7:86662963-86663649 forward strand	-1.39	0.038	GAATTTAGAAGGCAAATTAAATGCAGGCACCCCTTTATTGAATAGGTAGTGTCCATTCAC (Rfam database neg, no GO annotations, annotated by 1 database (NONCODE)).
L(i)ncRNA:chr9:113233366-113261487 reverse strand	-1.35	0.021	AATAACTTTAATGTCCACTGGAATGGCTCTGTGGAAGCTGCCCTGCCAAAGAAAACAGGT (Rfam database neg, no GO annotations, annotated by 1 database (NONCODE)).
L(i)ncRNA:chr9:31873801-31874407 forward strand	-1.30	0.008	TCTTATTAATGTCACAGGAATCCATTGATGGTTTGACCTATTACTAGAGTCTTTAGGATC (Rfam database neg, no GO annotations, annotated by 1 database (NONCODE), expressed in the hippocampus).
l(i)ncRNA:chr2:67411703-67412293 forward strand	-1.28	0.005	TAGGTCTTTTCCGGGTCCTGTCACCAATGCTAGGAATCAGCATCAATTTCTCTCAGTCTT (Rfam database neg, no GO annotations, annotated by 1 database (NONCODE), expressed in the hippocampus).
l(i)ncRNA:chr18:38776580-38841080 reverse strand	-1.23	0.032	GTCTCTTCCTGAACCTGTTCTGTATGCTGTCCCAGAGGCAGGCGTCTCCCTTTCCCTGAA (Rfam database neg, no GO annotations, annotated by 1 database (NONCODE), expressed in the hippocampus).
l(i)ncRNA:chr4:40840725-40860875 forward strand	-1.22	0.011	CTCCTACCTGAAGGACAGTTGGCAATCTAAATAATAAAAACACTGGTATGCATTCAAATC (Rfam database neg, no GO annotations, annotated by 1 database (NONCODE), expressed in the hippocampus).
l(i)ncRNA:chr4:123361002-123364781 reverse strand	-1.20	0.040	AAGAATGATCCTCAGCGTTTGCTAGCTGGCTGCTGCAGGGTTGAGGGAGTCTGGGTCAGT (Rfam database neg, no GO annotations, annotated by 1 database (NONCODE), expressed in the hippocampus).
l(i)ncRNA:chr17:22076711-22099431 reverse strand	-1.18	0.009	TGATTGTCTTTCCTTACCTGGCATTCAATCTGGCACTCTAAGAAAAGGTCTTTGGTGCTC (Rfam database neg, no GO annotations, annotated by 1 database (NONCODE)).
l(i)ncRNA:chr16:46384974-46391331 reverse strand	1.22	0.026	GGCGGGTTTTAGACAGTCCTTAAGTTTCCAGAAATTGTCTTTTATTTTTGCATTTTGAAC (Rfam database neg, no GO annotations, annotated by 1 database (NONCODE), expressed in the hippocampus).
l(i)ncRNA:chr9:14460976-14488051 forward strand	1.25	0.032	CACAGTAAAGAGCTCAGTCAAATGCAACTGCAAGTAGGTTGTTTTAAGTTGTTCAAGATA (Rfam database neg, no GO annotations, annotated by 1 database (NONCODE), expressed in the hippocampus).
l(i)ncRNA:chr10:6497224-6497863 reverse strand	1.36	0.039	TGACTCAGAATAGTCCTTAAGGCCACAGAAAACTAAACAAGGTTAAACGTGACAAGCCTG (Rfam database neg, no GO annotations, annotated by 1 database (NONCODE)).

l(i)ncRNA:chr8:26343277-26352902 reverse strand	1.38	0.044	AATTAAGAAGGACATGAGTCAGTGGTAGAGGCTTGCCTTAGCTCTGGGTTTCATACTCAG (Rfam database neg, no GO annotations, annotated by 1 database (NONCODE), expressed in the hippocampus).
l(i)ncRNA:chr6:4841700-4847400 reverse strand	1.44	0.014	GGACTCCCTGGTGGTGTGTGAAGTGGACCCGGAGCTAAAGGAAACATTGAGGAAATTCCG (Rfam database neg, no GO annotations, annotated by 1 database (NONCODE), expressed in the hippocampus).
