# Supplementary material for: Sex- and region-specific cortical and hippocampal whole genome transcriptome profiles from control and APP/PS1 Alzheimer’s disease mice
Source: PLoS One. 2024 Feb 7;19(2):e0296959. doi: 10.1371/journal.pone.0296959 (PMC10849391; doi:10.1371/journal.pone.0296959)
Supplement: S1 File — S1 Fig: Genotyping of APP/PS1 AD mice and WT control animals. S2 Fig: 3D image of the murine brain including the RS cortex and hippocampus (BROIs) used for transcriptome analysis in our study. S3 Fig: PCA of transcriptomes from the RS cortex and hippocampus of WT controls and APP/PS1 AD mice of both sexes. S4 Fig: Hierarchical clustering of transcriptome data from the RS cortex and hippocampus of WT control and APP/PS1 AD mice of both sexes. S5 Fig: Bar diagrams of the top 30 candidates of DEGs with highest significant FCs (FC > 1.5 and FC < -1.5, p < 0.05). S6 Fig: Pathway analysis of intersectional and signature gene sets in APP/PS1 subgroups. S7 Fig: Comparative qPCR analysis of selected gene transcript levels from the hippocampus of female and male APP/PS1 AD with 5XFAD mice. S1 Table: PCR reaction set-up using PCR Mastermix and genomic DNA. S2 Table: Materials used for one-color microarray-based gene expression data collection. S3 Table: Software used for one-color microarray-based gene expression data collection. S4 Table: Details on genes, forward and reverse primer sequences and annealing temperatures relevant for qPCR experimentation. S5 Table: Characteristics of DEGs in the RS cortex of female APP/PS1 AD mice. S6 Table: Characteristics of DEGs in the hippocampus of female APP/PS1 AD mice. S7 Table: Characteristics of DEGs in the RS cortex of male APP/PS1 AD mice. S8 Table: Characteristics of DEGs in the hippocampus of male APP/PS1 AD mice. S9 Table: Venn analysis of DEGs in the RS cortex and hippocampus of female APP/PS1 AD mice. S10 Table: Venn analysis of DEGs genes in the RS cortex and hippocampus of male APP/PS1 AD mice. S11 Table: Venn analysis of DEGs in the RS cortex of male and female APP/PS1 AD mice. S12 Table: Venn analysis of DEGs in the hippocampus of male and female APP/PS1 AD mice. S13 Table: Differentially regulated l(i)ncRNAs in APP/PS1 AD vs. WT mice. S14 Table: qPCR-based FC analysis of selected genes in the hippocampus of APP/PS1 AD vs. [file pone.0296959.s001.zip › Supplementary Files_R1/Supplementary text file.pdf]

Supporting information

Sex- and region-specific cortical and hippocampal whole genome transcriptome profiles from control and APP/PS1 Alzheimer's disease mice

Anna Papazoglou [1], Christina Henseler [1], Sandra Weickhardt [2], Jenni Teipelke [1], Panagiota Papazoglou [1], Johanna Daubner [1], Teresa Schiffer [1], Damian Krings [1], Karl Broich [2], Jürgen Hescheler [3,4], Agapios Sachinidis [3,4], Dan Ehninger [5,6], Catharina Scholl [2], Britta Haenisch [2,6,7] and Marco Weiergräber [1,2,3,4,*]

[1] Experimental Neuropsychopharmacology, Federal Institute for Drugs and Medical Devices (Bundesinstitut für Arzneimittel und Medizinprodukte, BfArM), Bonn, Germany.

[2] Federal Institute for Drugs and Medical Devices (Bundesinstitut für Arzneimittel und Medizinprodukte, BfArM), Bonn, Germany.

[3] Faculty of Medicine, Institute of Neurophysiology, University of Cologne, Cologne, Germany.

[4] Center of Physiology and Pathophysiology, Faculty of Medicine, University of Cologne, Cologne, Germany.

[5] Translational Biogerontology, German Center for Neurodegenerative Diseases (Deutsches Zentrum für Neurodegenerative Erkrankungen, DZNE), Venusberg-Campus 1/99, Bonn, Germany.

[6] German Center for Neurodegenerative Diseases (Deutsches Zentrum für Neurodegenerative Erkrankungen, DZNE), Venusberg-Campus 1/99, Bonn, Germany.

[7] Center for Translational Medicine, Medical Faculty, University of Bonn, Bonn, Germany.

* Corresponding author

Email: marco.weiergraeber@bfarm.de.

Supplementary Figures

Suppl Fig 1: Genotyping of APP/PS1 AD mice and WT control animals. Genotyping of APPswePS1dE9 (lane 3) and control (WT) mice (lane 2). The individual genotypes are characterized by specific DNA fragments. The 377 bp fragment is indicative of the mutant APP variant, whereas the 608 bp fragment characterizes the mutant PS1 variant. The muscarinic receptor 5 (*Chrm5*) was used as a positive control (see 257 bp fragment). For the negative control (no genomic DNA) see lane 4.

Suppl Fig 2: 3D image of the murine brain including the RS cortex and hippocampus (BROIs) used for transcriptome analysis in our study. The RS cortex and hippocampus (ammon's horn) were extirpated for subsequent RNA isolation and further processing for microarray-based transcriptome analysis. Both BROIs were chosen due to their early functional involvement in AD pathogenesis in both mice and humans. The 3D image was created using the Allen Brain Explorer® beta version (https://connectivity.brain-map.org/static/brainexplorer).

Suppl Fig 3: PCA of transcriptomes from the RS cortex and hippocampus of WT controls and APP/PS1 AD mice of both sexes. (A) The PCA image depicted here represents the PCA scores in a 3D scatter plot. The replicates in the eight individual subgroups (WT ♀ Cx, WT ♀ Hip, WT ♂ Cx, WT ♂ Hip, APP/PS1 ♀ Cx, APP/PS1 ♀ Hip, APP/PS1 ♂ Cx, APP/PS1 ♂ Hip) cluster together and separate from arrays in other subgroups. The individual subgroups are color-coded as indicated. Note that the related transcriptomes are derived from individual mice in a social, i.e., hierarchical dominance-subordination, network context that can potentially affect the transcriptome profile. (B)

PCA image (as in A) with color-coding denoted to both genotypes. (C) PCA plot (as in A) with color-coding denoted to sex. (D) PCA image (as in A) with color coding denoted to the BROI. There is a general cluster tendency of samples related to genotype, sex and BROI.

Suppl Fig 4: Hierarchical clustering of transcriptome data from the RS cortex and hippocampus of WT control and APP/PS1 AD mice of both sexes. Results are provided for the individual subgroups, i.e., WT ♀ Cx, WT ♀ Hip, WT ♂ Cx, WT ♂ Hip, APP/PS1 ♀ Cx, APP/PS1 ♀ Hip, APP/PS1 ♂ Cx, APP/PS1 ♂ Hip. The related FC are color-coded and scaled as indicated. Notably, sex and BROI seem to be the most important clustering factors.

Suppl Fig 5: Bar diagrams of the top 30 candidates of DEGs with highest significant FCs (FC > 1.5 and FC < -1.5, p < 0.05). (A) the RS cortex in female APP/PS1 AD vs. WT mice, (B) the hippocampus of female APP/PS1 AD vs. WT mice, (C) the RS cortex of male APP/PS1 AD vs. WT mice and (D) the hippocampus of male APP/PS1 AD vs. WT mice. Fold changes of downregulated genes are high-lighted in blue, FCs of upregulated genes are highlighted in orange. Note that the number of upregulated genes substantially exceeds the number of downregulated genes in the individual four subgroups.

Suppl Fig 6: Pathway analysis of intersectional and signature gene sets in APP/PS1 subgroups. The co-upregulated and co-downregulated gene sets determined in the Venn studies were analyzed using *Reactome* (reactome.org). The same approach was carried out for the fingerprint genes/signature genes which are indicative of the individual APP/PS1 subgroups. For each gene set, a pathway analysis report from *Reactome* is provided. The latter includes a hierarchical list of the top pathways involved/affected and a detailed biochemical/physiological description/scheme of the individual pathways. Note that pathway analysis of intersectional, downregulated genes was not applicable, due to the lack of gene candidates.

Suppl Fig 7: Comparative qPCR analysis of selected gene transcript levels from the hippocampus of female and male APP/PS1 AD with 5XFAD mice. Transcript levels (CNRQ) of eight APP/PS1 AD mice (four ♂, four ♀, blue) and eight WT control animals (four ♂, four ♀, black) are depicted using scatter plots including mean ± SEM. Results

are illustrated for *Casp8* (A, B), *Plcd4* (C, D), *Cacna1d* (E, F), *Cacna1c* (G, H) and *Chrm1, Chrm3, Chrm5* (I - N). These gene candidates were selected based on previous studies in 5XFAD mice. Note that not all DEGs validated in 5XFAD mice were altered in APP/PS1. The latter finding stresses the functional etiopathological differences between individual AD mouse models.

Supplementary Tables

Suppl Tab 1: PCR reaction set-up using PCR Mastermix and genomic DNA. All components necessary for genotyping are listed including forward and reverse primer sequence information for characterization of APP and PSEN1 of APP/PS1 AD mutant mice and muscarinic receptor 5 (*Chrm5*) for WT control animals/positive control.

Suppl Tab 2: Materials used for one-color microarray-based gene expression data collection. This table contains a list of all experimental kits used for isolation and purification of RNA, 1st and 2nd strand cDNA synthesis, amplification and labelling, hybridization and washing steps. All procedures applied in our study are in accordance with the manufacturer's instructions.

Suppl Tab 3: Software used for one-color microarray-based gene expression data collection. This table illustrates software used for RNA quantification, microarray scanning, feature extraction, and transcriptome analysis. All procedures applied in our study are in accordance with the manufacturer's instructions.

Suppl Tab 4: Details on genes, forward and reverse primer sequences and annealing temperatures relevant for qPCR experimentation. The following genes, i.e., *Cacna1d, Cacna1c, Plcd4, Casp8, Chrm1, Chrm3, Chrm5, Siglech, Ptpn6, Laptm5, Plek, Arpp21, Shisa9*, were analyzed for quantitative transcriptional alterations using qPCR. Both forward and reverse primer sequences, annealing temperatures and information about the primer sources are provided. *Hprt* was used as an internal reference gene (positive control).

Suppl Tab 5: Characteristics of DEGs in the RS cortex of female APP/PS1 AD vs. WT mice. Following the microarray scan via Agilent SureScan Microarray Scanner, feature extraction was carried out using Feature Extraction Software (both from Agilent Technologies Germany GmbH & Co. KG, Germany). This table contains relevant information of DEGs in the RS cortex of female APP/PS1 AD mice vs. WT controls, i.e., gene symbol, gene name, gene description and sequence, FCs, and GO categorization. Note that p-values are based on one-way ANOVA, Tukey's *post-hoc* test and Benjamini-Hochberg correction. All genes with $p < 0.05$ are listed and ranked based on their FC values. For orientation, gene candidates with a FC > 1.5 and a FC < -1.5 are highlighted in light blue.

Suppl Tab 6: Characteristics of DEGs in the hippocampus of female APP/PS1 AD vs. WT mice. Following the microarray scan via Agilent SureScan Microarray Scanner, feature extraction was carried out using Feature Extraction Software (both from Agilent Technologies Germany GmbH & Co. KG, Germany). This table contains relevant information of DEGs in the hippocampus of female APP/PS1 AD mice vs. WT controls, i.e., gene symbol, gene name, gene description and sequence, FCs, and GO categorization. Note that p-values are based on one-way ANOVA, Tukey's *post-hoc* test and Benjamini-Hochberg correction. All genes with $p < 0.05$ are listed and ranked based on their FC values. Candidates with a FC > 1.5 and a FC < -1.5 are highlighted in light blue.

Suppl Tab 7: Characteristics of DEGs in the RS cortex of male APP/PS1 AD vs. WT mice. Following the microarray scan via Agilent SureScan Microarray Scanner, feature extraction was carried out using Feature Extraction Software (both from Agilent Technologies Germany GmbH & Co. KG, Germany). This table contains relevant information of DEGs in the RS cortex of male APP/PS1 AD mice vs. WT controls, i.e., gene symbol, gene name, gene description and sequence, FCs, and GO categorization. Note that p-values are based on one-way ANOVA, Tukey's *post-hoc* test and Benjamini-Hochberg correction. All genes with $p < 0.05$ are listed and ranked based on their FC values. Candidates with a FC > 1.5 and a FC < -1.5 are highlighted in light blue.

Suppl Tab 8: Characteristics of DEGs in the hippocampus of male APP/PS1 AD vs. WT mice. Following the microarray scan via Agilent SureScan Microarray Scanner, feature extraction was carried out using Feature

Extraction Software (both from Agilent Technologies Germany GmbH & Co. KG, Germany). This table contains relevant information of DEGs in the hippocampus of male APP/PS1 AD mice vs. WT controls, i.e., gene symbol, gene name, gene description and sequence, FC, and GO categorization. Note that p-values are based on one-way ANOVA, Tukey's *post-hoc* test and Benjamini-Hochberg correction. All genes with $p < 0.05$ are listed and ranked based on their FC values. Candidates with a FC > 1.5 and a FC < -1.5 are highlighted in light blue.

Suppl Tab 9: Venn analysis of DEGs in the RS cortex and hippocampus of female APP/PS1 AD vs. WT mice. Detailed information about DEGs is provided for both up (FC > 1.5) (**Suppl Tab 9A**) and downregulated (FC < -1.5) (**Suppl Tab 9B**) gene candidates. Each file contains a Venn diagram, a separate list of all upregulated/downregulated genes in the RS cortex of female APP/PS1 AD mice and the hippocampus of female APP/PS1 AD mice, a list of co-upregulated/co-downregulated genes in both subgroups and gene candidate lists that are exclusively upregulated/downregulated in each individual subgroup.

Suppl Tab 10: Venn analysis of DEGs genes in the RS cortex and hippocampus of male APP/PS1 AD vs. WT mice. Detailed information about DEGs is provided for both up (FC > 1.5) (**Suppl Tab 10A**) and downregulated (FC < -1.5) (**Suppl Tab 10B**) gene candidates. Each file contains a Venn diagram, a separate list of all upregulated/downregulated genes in the RS cortex of male APP/PS1 AD mice and the hippocampus of male APP/PS1 AD mice, a list of co-upregulated/co-downregulated genes in both subgroups and candidate lists that are exclusively upregulated/downregulated in each individual subgroup.

Suppl tab 11: Venn analysis of DEGs in the RS cortex of male and female APP/PS1 AD vs. WT mice. Detailed information about DEGs is provided for both up (FC > 1.5) (**Suppl Tab 11A**) and downregulated (FC < -1.5) (**Suppl Tab 11B**) candidates. Each file contains a Venn diagram, a separate list of all upregulated/downregulated genes in the RS cortex of male APP/PS1 AD mice and the RS cortex of female APP/PS1 AD mice, a list of co-upregulated/co-downregulated genes in both subgroups and candidate lists that are exclusively upregulated/downregulated in each individual subgroup.

Suppl Tab 12: Venn analysis of DEGs in the hippocampus of male and female APP/PS1 AD vs. WT mice. Detailed information about DEGs is provided for both up (FC > 1.5) (**Suppl Tab 12A**) and downregulated (FC < -1.5) (**Suppl Tab 12B**) candidates. Each file contains a Venn diagram, a separate list of all upregulated/downregulated genes in the hippocampus of male APP/PS1 AD mice and the hippocampus of female APP/PS1 AD mice, a list of co-upregulated/co-downregulated genes in both subgroups and candidate lists that are exclusively upregulated/downregulated in each individual subgroup.

Suppl Tab 13: Differentially regulated l(i)ncRNAs in APP/PS1 AD vs. WT mice. This table characterizes the individual, differentially regulated l(i)ncRNAs, their FCs, p-values and the related sequences. Downregulated l(i)ncRNAs are depicted in light green, upregulated l(i)ncRNAs are highlighted in light yellow for (A) the RS cortex of female APP/PS1 AD mice, for (B) the hippocampus of female APP/PS1 AD mice, for (C) the RS cortex of male APP/PS1 AD mice and (D) the hippocampus of male APP/PS1 AD mice. In addition, information about potential roles of individual l(i)ncRNAs in AD are depicted below the individual l(i)ncRNA sequences (based on RNAcentral („https://rnacentral.org/"), Rfam (Rfam.org), the Coding-Potential Assessment Tool (CPAT), and various databases, e.g., NONCODE ("http://www.noncode.org/")).

Suppl Tab 14: qPCR-based fold change analysis of selected genes in the cortex and hippocampus of APP/PS1 AD vs. WT control mice. (A) qPCR-based fold change analysis of selected genes in the hippocampus of APP/PS1 mice that were previously identified in the 5XFAD AD model (33,34). (B) qPCR-based FC analysis of selected DEGs (from our transcriptome study) in the RS cortex of APP/PS1 mice vs. WT control animals. FCs from qPCR studies are listed first, those from microarrays are given in brackets. Note that negative FCs indicate downregulation, positive FCs indicate upregulation of related gene candidates. Significant values ($p < 0.05$) and statistical trends ($0.05 < p < 0.1$) are highlighted in bold.

Suppl Tab 15: Design of transcriptome studies carried out in APP/PS1 AD mice. All studies listed here utilized the same APP/PS1 AD model. However, the ages of the experimental animals and the target tissue/BROI severely differ between the individual studies. Importantly, there is no sex-specific analysis so far, as previous studies have either used male mice only or pooled both sexes (in a fixed ratio or without making further comments on the sex

distribution pattern). Note that pharmacological transcriptome studies in APP/PS1 AD mice are not included. The same holds true for transcriptome studies in other AD mouse models.

Suppl Tab 16: Functional implications of DEGs in AD. The list presents gene symbols (top 30, FC > 1.5, in alphabetical order), gene descriptions, functional characteristics and potential roles in AD etiopathogenesis. Note that the information for each individual gene is not exhaustive in nature. Instead, it is supposed to provide a brief and fast orientation for the reader.
