# Supplementary figures and images for: Sex- and region-specific cortical and hippocampal whole genome transcriptome profiles from control and APP/PS1 Alzheimer’s disease mice
Source: PLoS One. 2024 Feb 7;19(2):e0296959. doi: 10.1371/journal.pone.0296959 (PMC10849391; doi:10.1371/journal.pone.0296959)

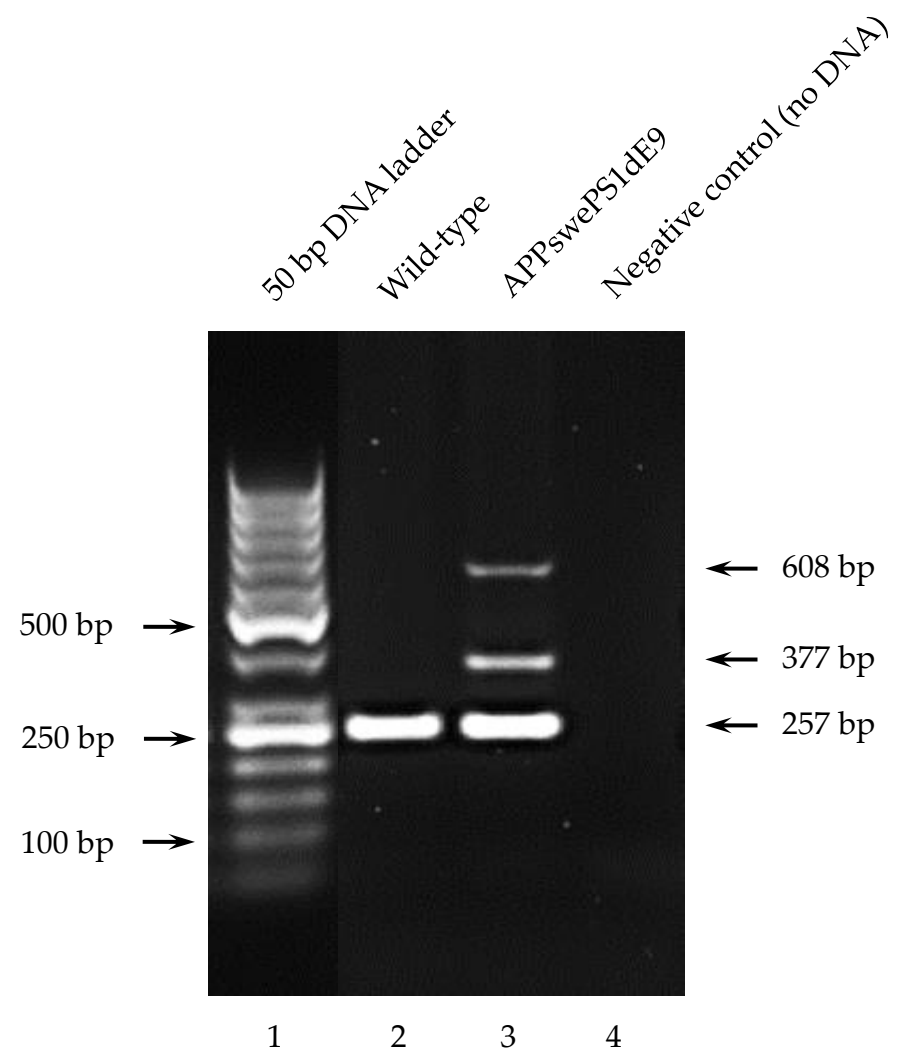

Supplement: S1 File — S1 Fig: Genotyping of APP/PS1 AD mice and WT control animals. S2 Fig: 3D image of the murine brain including the RS cortex and hippocampus (BROIs) used for transcriptome analysis in our study. S3 Fig: PCA of transcriptomes from the RS cortex and hippocampus of WT controls and APP/PS1 AD mice of both sexes. S4 Fig: Hierarchical clustering of transcriptome data from the RS cortex and hippocampus of WT control and APP/PS1 AD mice of both sexes. S5 Fig: Bar diagrams of the top 30 candidates of DEGs with highest significant FCs (FC > 1.5 and FC < -1.5, p < 0.05). S6 Fig: Pathway analysis of intersectional and signature gene sets in APP/PS1 subgroups. S7 Fig: Comparative qPCR analysis of selected gene transcript levels from the hippocampus of female and male APP/PS1 AD with 5XFAD mice. S1 Table: PCR reaction set-up using PCR Mastermix and genomic DNA. S2 Table: Materials used for one-color microarray-based gene expression data collection. S3 Table: Software used for one-color microarray-based gene expression data collection. S4 Table: Details on genes, forward and reverse primer sequences and annealing temperatures relevant for qPCR experimentation. S5 Table: Characteristics of DEGs in the RS cortex of female APP/PS1 AD mice. S6 Table: Characteristics of DEGs in the hippocampus of female APP/PS1 AD mice. S7 Table: Characteristics of DEGs in the RS cortex of male APP/PS1 AD mice. S8 Table: Characteristics of DEGs in the hippocampus of male APP/PS1 AD mice. S9 Table: Venn analysis of DEGs in the RS cortex and hippocampus of female APP/PS1 AD mice. S10 Table: Venn analysis of DEGs genes in the RS cortex and hippocampus of male APP/PS1 AD mice. S11 Table: Venn analysis of DEGs in the RS cortex of male and female APP/PS1 AD mice. S12 Table: Venn analysis of DEGs in the hippocampus of male and female APP/PS1 AD mice. S13 Table: Differentially regulated l(i)ncRNAs in APP/PS1 AD vs. WT mice. S14 Table: qPCR-based FC analysis of selected genes in the hippocampus of APP/PS1 AD vs. [file pone.0296959.s001.zip › Supplementary Files_R1/Supplementary Figure 1_Genotyping.pdf]

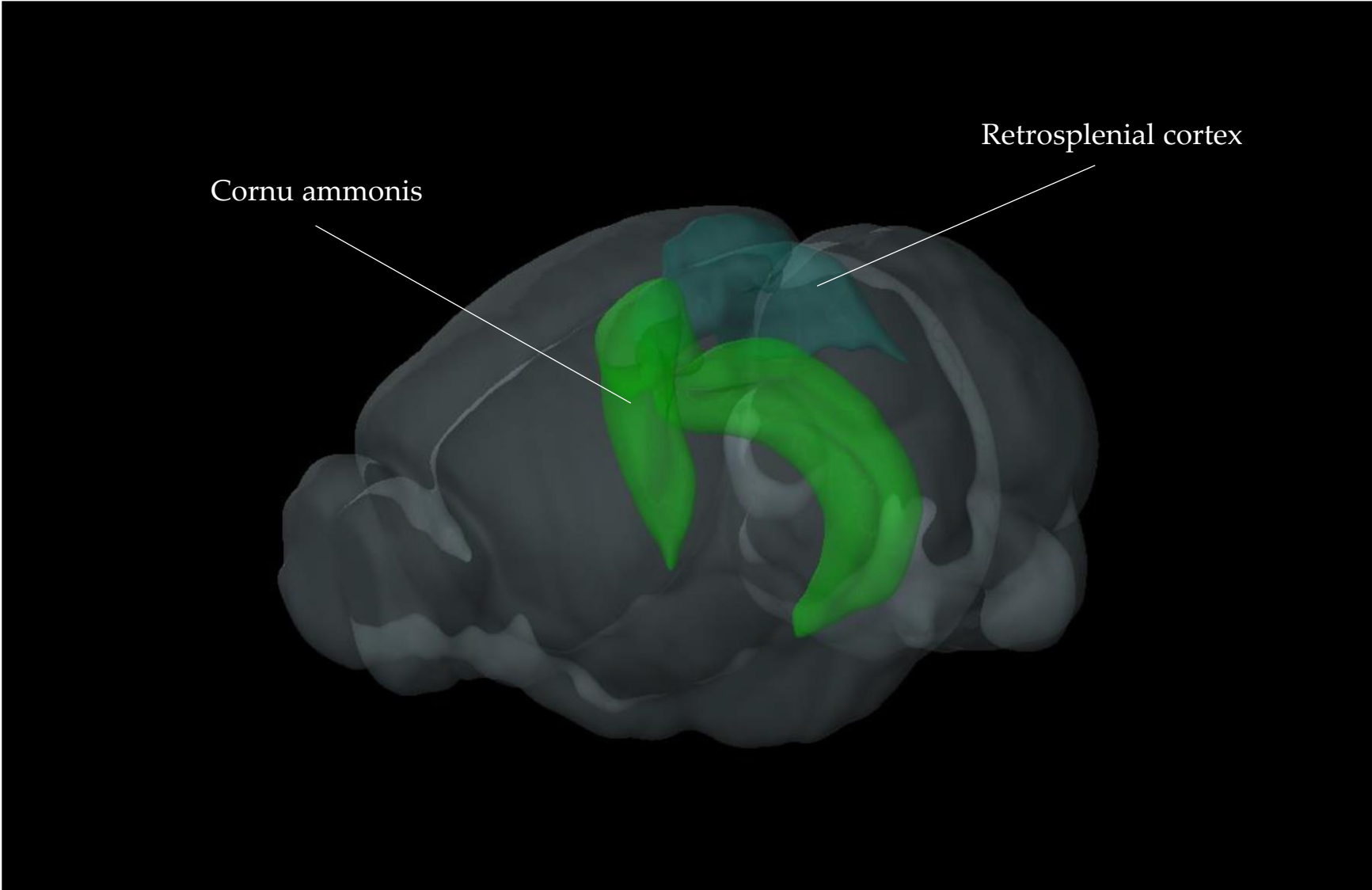

Supplement: S1 File — S1 Fig: Genotyping of APP/PS1 AD mice and WT control animals. S2 Fig: 3D image of the murine brain including the RS cortex and hippocampus (BROIs) used for transcriptome analysis in our study. S3 Fig: PCA of transcriptomes from the RS cortex and hippocampus of WT controls and APP/PS1 AD mice of both sexes. S4 Fig: Hierarchical clustering of transcriptome data from the RS cortex and hippocampus of WT control and APP/PS1 AD mice of both sexes. S5 Fig: Bar diagrams of the top 30 candidates of DEGs with highest significant FCs (FC > 1.5 and FC < -1.5, p < 0.05). S6 Fig: Pathway analysis of intersectional and signature gene sets in APP/PS1 subgroups. S7 Fig: Comparative qPCR analysis of selected gene transcript levels from the hippocampus of female and male APP/PS1 AD with 5XFAD mice. S1 Table: PCR reaction set-up using PCR Mastermix and genomic DNA. S2 Table: Materials used for one-color microarray-based gene expression data collection. S3 Table: Software used for one-color microarray-based gene expression data collection. S4 Table: Details on genes, forward and reverse primer sequences and annealing temperatures relevant for qPCR experimentation. S5 Table: Characteristics of DEGs in the RS cortex of female APP/PS1 AD mice. S6 Table: Characteristics of DEGs in the hippocampus of female APP/PS1 AD mice. S7 Table: Characteristics of DEGs in the RS cortex of male APP/PS1 AD mice. S8 Table: Characteristics of DEGs in the hippocampus of male APP/PS1 AD mice. S9 Table: Venn analysis of DEGs in the RS cortex and hippocampus of female APP/PS1 AD mice. S10 Table: Venn analysis of DEGs genes in the RS cortex and hippocampus of male APP/PS1 AD mice. S11 Table: Venn analysis of DEGs in the RS cortex of male and female APP/PS1 AD mice. S12 Table: Venn analysis of DEGs in the hippocampus of male and female APP/PS1 AD mice. S13 Table: Differentially regulated l(i)ncRNAs in APP/PS1 AD vs. WT mice. S14 Table: qPCR-based FC analysis of selected genes in the hippocampus of APP/PS1 AD vs. [file pone.0296959.s001.zip › Supplementary Files_R1/Supplementary Figure 2_BROIs.pdf]

Supplementary Figure 4 (Papazoglou et al., 2023)

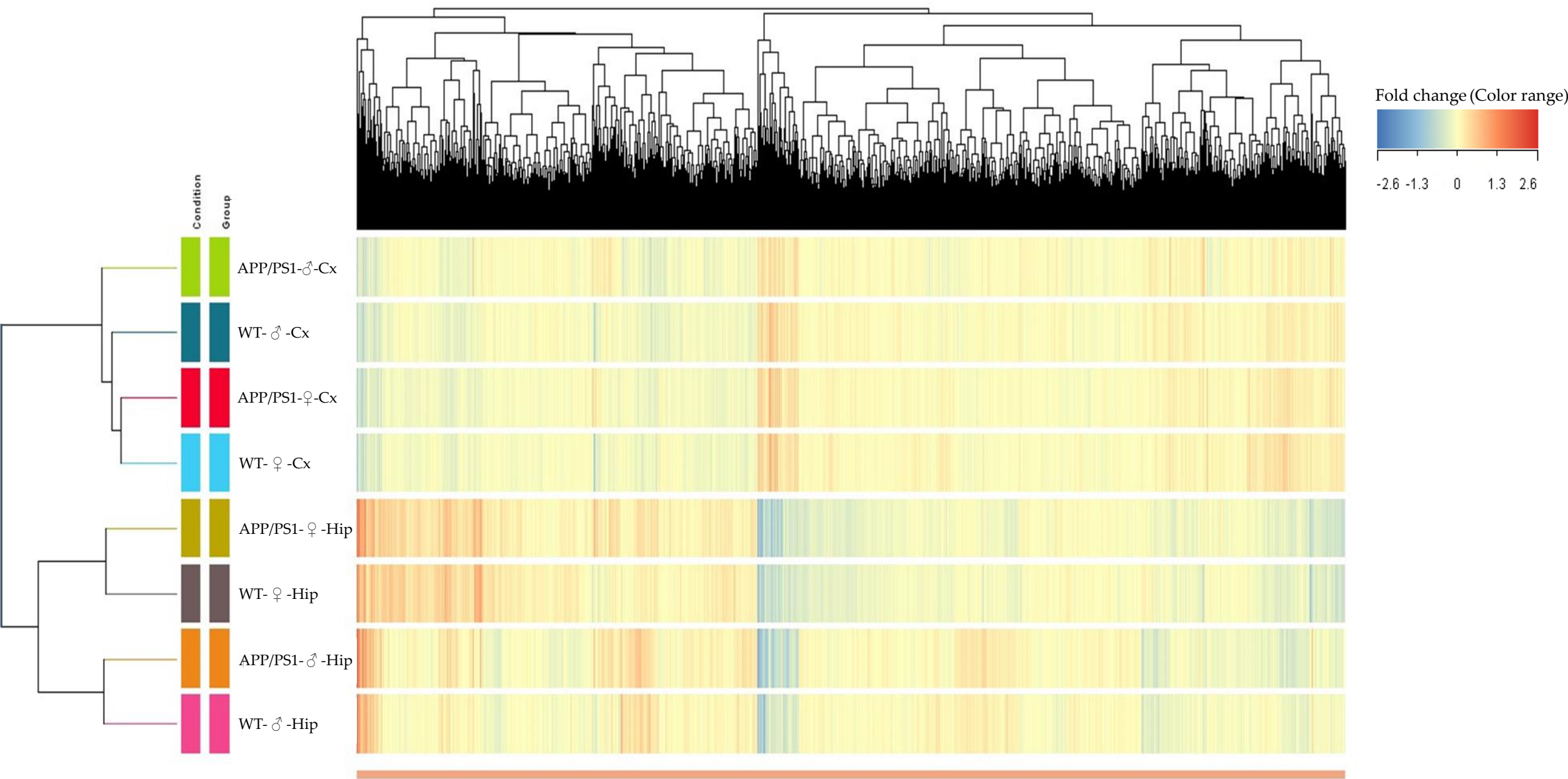

Supplement: S1 File — S1 Fig: Genotyping of APP/PS1 AD mice and WT control animals. S2 Fig: 3D image of the murine brain including the RS cortex and hippocampus (BROIs) used for transcriptome analysis in our study. S3 Fig: PCA of transcriptomes from the RS cortex and hippocampus of WT controls and APP/PS1 AD mice of both sexes. S4 Fig: Hierarchical clustering of transcriptome data from the RS cortex and hippocampus of WT control and APP/PS1 AD mice of both sexes. S5 Fig: Bar diagrams of the top 30 candidates of DEGs with highest significant FCs (FC > 1.5 and FC < -1.5, p < 0.05). S6 Fig: Pathway analysis of intersectional and signature gene sets in APP/PS1 subgroups. S7 Fig: Comparative qPCR analysis of selected gene transcript levels from the hippocampus of female and male APP/PS1 AD with 5XFAD mice. S1 Table: PCR reaction set-up using PCR Mastermix and genomic DNA. S2 Table: Materials used for one-color microarray-based gene expression data collection. S3 Table: Software used for one-color microarray-based gene expression data collection. S4 Table: Details on genes, forward and reverse primer sequences and annealing temperatures relevant for qPCR experimentation. S5 Table: Characteristics of DEGs in the RS cortex of female APP/PS1 AD mice. S6 Table: Characteristics of DEGs in the hippocampus of female APP/PS1 AD mice. S7 Table: Characteristics of DEGs in the RS cortex of male APP/PS1 AD mice. S8 Table: Characteristics of DEGs in the hippocampus of male APP/PS1 AD mice. S9 Table: Venn analysis of DEGs in the RS cortex and hippocampus of female APP/PS1 AD mice. S10 Table: Venn analysis of DEGs genes in the RS cortex and hippocampus of male APP/PS1 AD mice. S11 Table: Venn analysis of DEGs in the RS cortex of male and female APP/PS1 AD mice. S12 Table: Venn analysis of DEGs in the hippocampus of male and female APP/PS1 AD mice. S13 Table: Differentially regulated l(i)ncRNAs in APP/PS1 AD vs. WT mice. S14 Table: qPCR-based FC analysis of selected genes in the hippocampus of APP/PS1 AD vs. [file pone.0296959.s001.zip › Supplementary Files_R1/Supplementary Figure 4_Heatmaps.pdf]

- Wild-type (n = 4)
- APP/PS1 (n = 4)

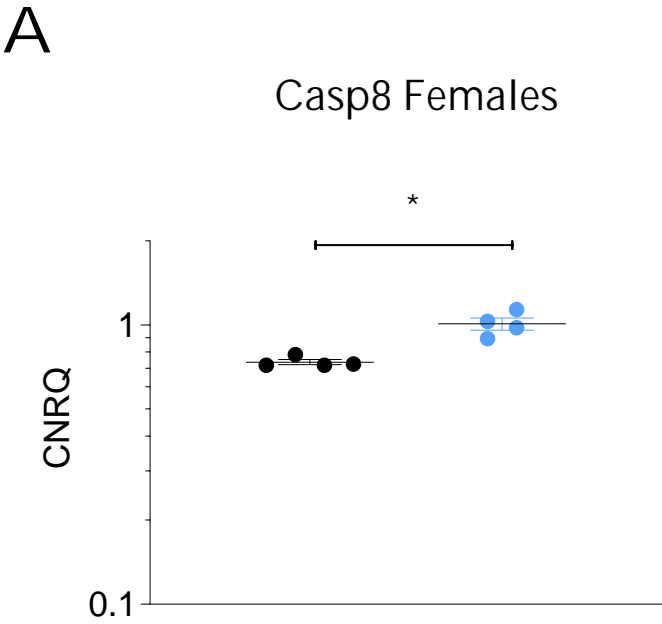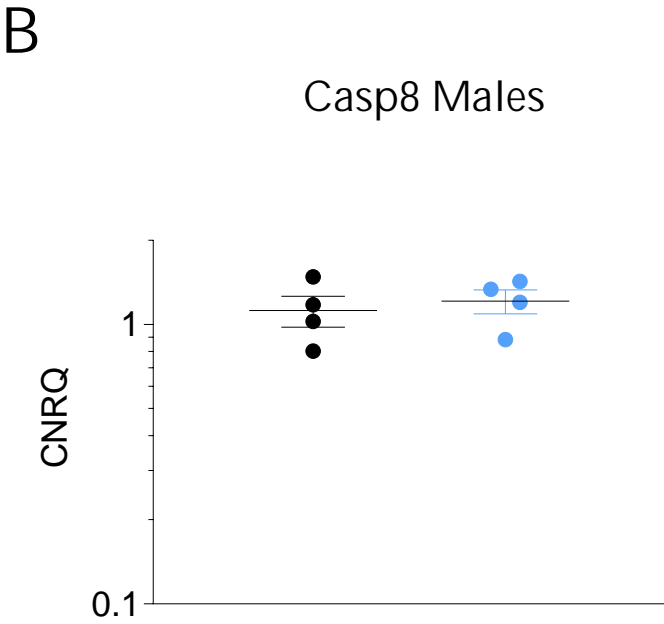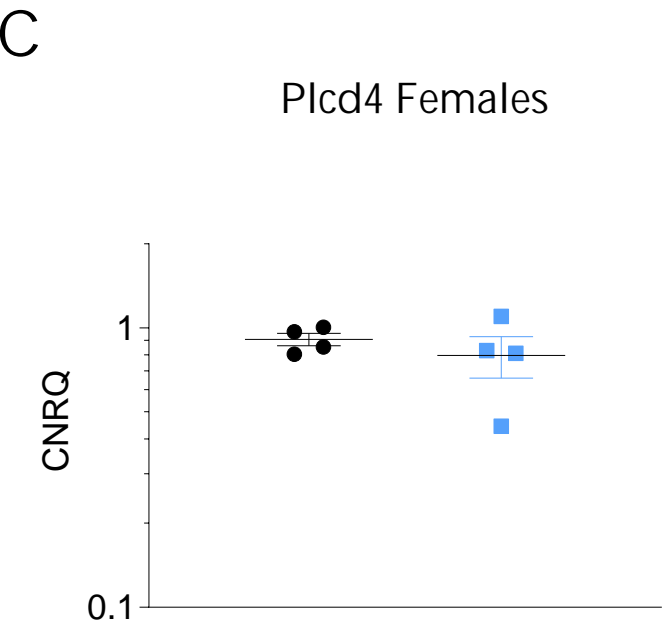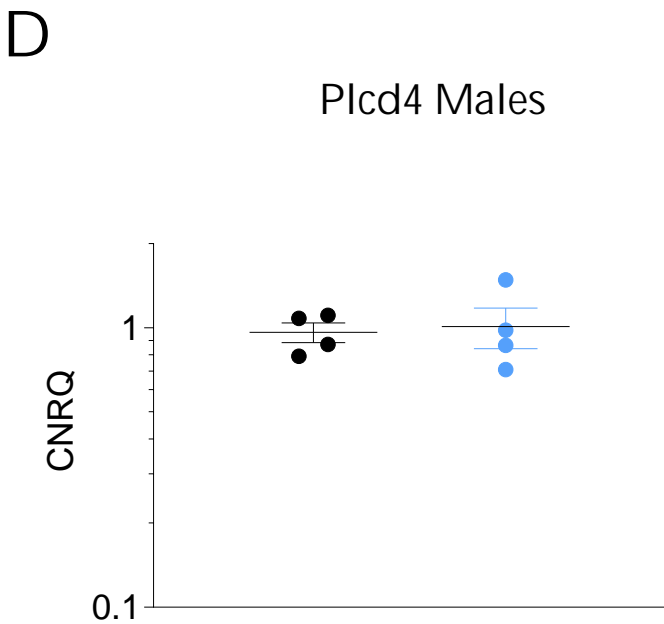

Supplement: S1 File — S1 Fig: Genotyping of APP/PS1 AD mice and WT control animals. S2 Fig: 3D image of the murine brain including the RS cortex and hippocampus (BROIs) used for transcriptome analysis in our study. S3 Fig: PCA of transcriptomes from the RS cortex and hippocampus of WT controls and APP/PS1 AD mice of both sexes. S4 Fig: Hierarchical clustering of transcriptome data from the RS cortex and hippocampus of WT control and APP/PS1 AD mice of both sexes. S5 Fig: Bar diagrams of the top 30 candidates of DEGs with highest significant FCs (FC > 1.5 and FC < -1.5, p < 0.05). S6 Fig: Pathway analysis of intersectional and signature gene sets in APP/PS1 subgroups. S7 Fig: Comparative qPCR analysis of selected gene transcript levels from the hippocampus of female and male APP/PS1 AD with 5XFAD mice. S1 Table: PCR reaction set-up using PCR Mastermix and genomic DNA. S2 Table: Materials used for one-color microarray-based gene expression data collection. S3 Table: Software used for one-color microarray-based gene expression data collection. S4 Table: Details on genes, forward and reverse primer sequences and annealing temperatures relevant for qPCR experimentation. S5 Table: Characteristics of DEGs in the RS cortex of female APP/PS1 AD mice. S6 Table: Characteristics of DEGs in the hippocampus of female APP/PS1 AD mice. S7 Table: Characteristics of DEGs in the RS cortex of male APP/PS1 AD mice. S8 Table: Characteristics of DEGs in the hippocampus of male APP/PS1 AD mice. S9 Table: Venn analysis of DEGs in the RS cortex and hippocampus of female APP/PS1 AD mice. S10 Table: Venn analysis of DEGs genes in the RS cortex and hippocampus of male APP/PS1 AD mice. S11 Table: Venn analysis of DEGs in the RS cortex of male and female APP/PS1 AD mice. S12 Table: Venn analysis of DEGs in the hippocampus of male and female APP/PS1 AD mice. S13 Table: Differentially regulated l(i)ncRNAs in APP/PS1 AD vs. WT mice. S14 Table: qPCR-based FC analysis of selected genes in the hippocampus of APP/PS1 AD vs. [file pone.0296959.s001.zip › Supplementary Files_R1/Supplementary Figure 7_A_D_qPCR.pdf]

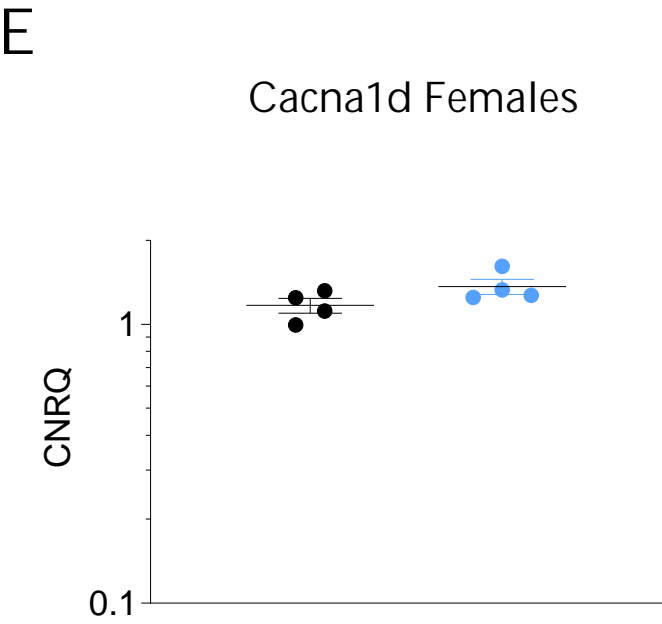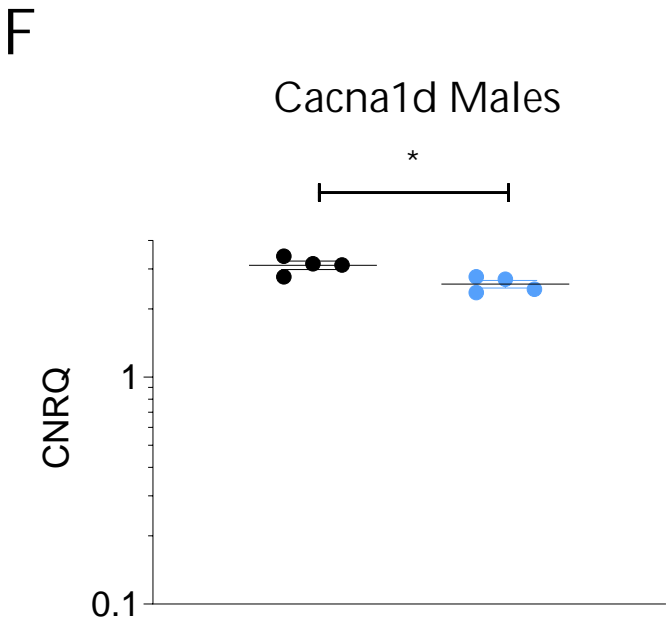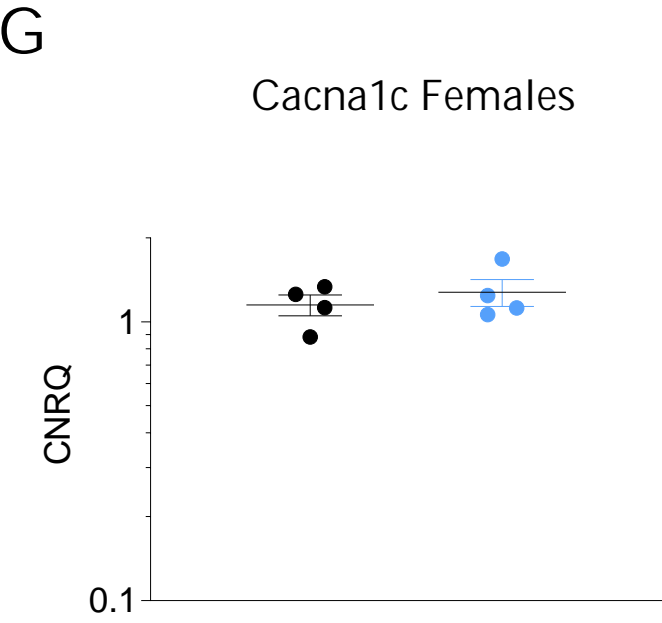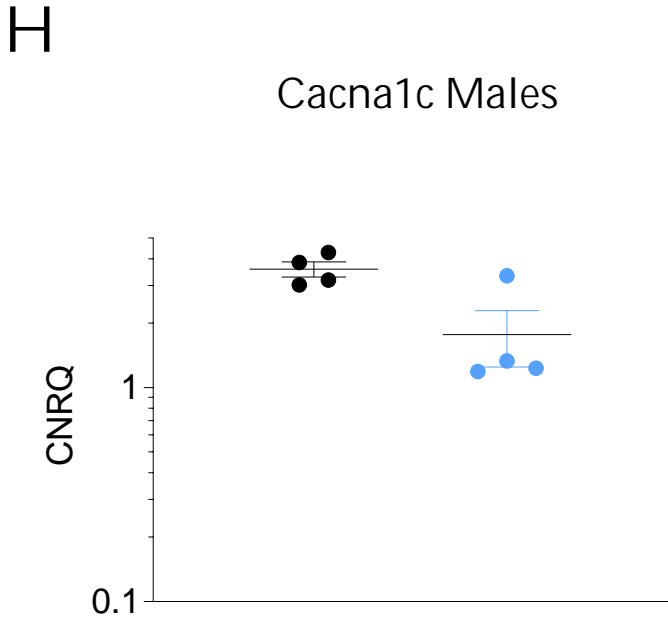

Supplement: S1 File — S1 Fig: Genotyping of APP/PS1 AD mice and WT control animals. S2 Fig: 3D image of the murine brain including the RS cortex and hippocampus (BROIs) used for transcriptome analysis in our study. S3 Fig: PCA of transcriptomes from the RS cortex and hippocampus of WT controls and APP/PS1 AD mice of both sexes. S4 Fig: Hierarchical clustering of transcriptome data from the RS cortex and hippocampus of WT control and APP/PS1 AD mice of both sexes. S5 Fig: Bar diagrams of the top 30 candidates of DEGs with highest significant FCs (FC > 1.5 and FC < -1.5, p < 0.05). S6 Fig: Pathway analysis of intersectional and signature gene sets in APP/PS1 subgroups. S7 Fig: Comparative qPCR analysis of selected gene transcript levels from the hippocampus of female and male APP/PS1 AD with 5XFAD mice. S1 Table: PCR reaction set-up using PCR Mastermix and genomic DNA. S2 Table: Materials used for one-color microarray-based gene expression data collection. S3 Table: Software used for one-color microarray-based gene expression data collection. S4 Table: Details on genes, forward and reverse primer sequences and annealing temperatures relevant for qPCR experimentation. S5 Table: Characteristics of DEGs in the RS cortex of female APP/PS1 AD mice. S6 Table: Characteristics of DEGs in the hippocampus of female APP/PS1 AD mice. S7 Table: Characteristics of DEGs in the RS cortex of male APP/PS1 AD mice. S8 Table: Characteristics of DEGs in the hippocampus of male APP/PS1 AD mice. S9 Table: Venn analysis of DEGs in the RS cortex and hippocampus of female APP/PS1 AD mice. S10 Table: Venn analysis of DEGs genes in the RS cortex and hippocampus of male APP/PS1 AD mice. S11 Table: Venn analysis of DEGs in the RS cortex of male and female APP/PS1 AD mice. S12 Table: Venn analysis of DEGs in the hippocampus of male and female APP/PS1 AD mice. S13 Table: Differentially regulated l(i)ncRNAs in APP/PS1 AD vs. WT mice. S14 Table: qPCR-based FC analysis of selected genes in the hippocampus of APP/PS1 AD vs. [file pone.0296959.s001.zip › Supplementary Files_R1/Supplementary Figure 7_E_H_qPCR.pdf]

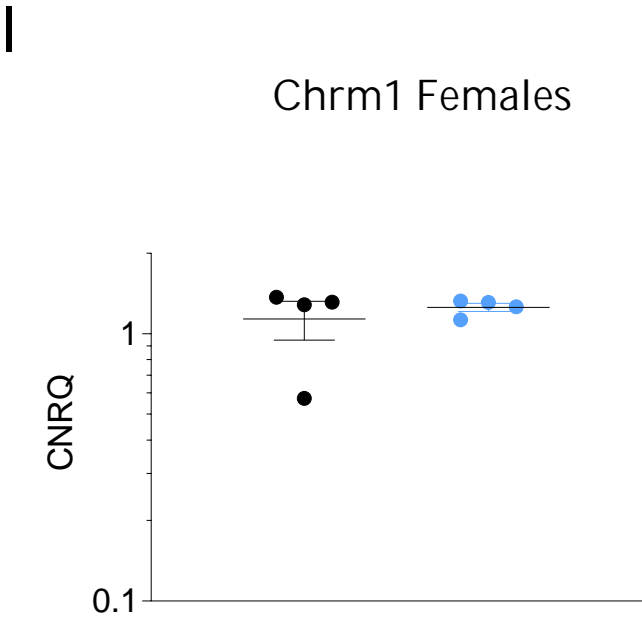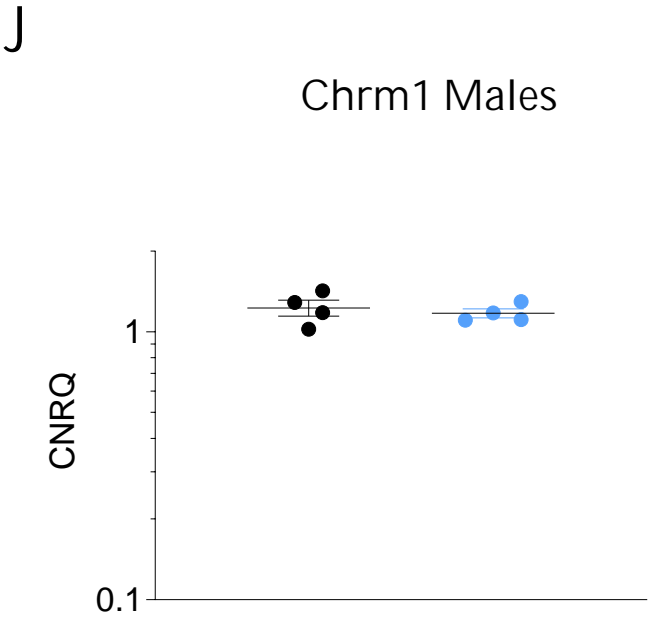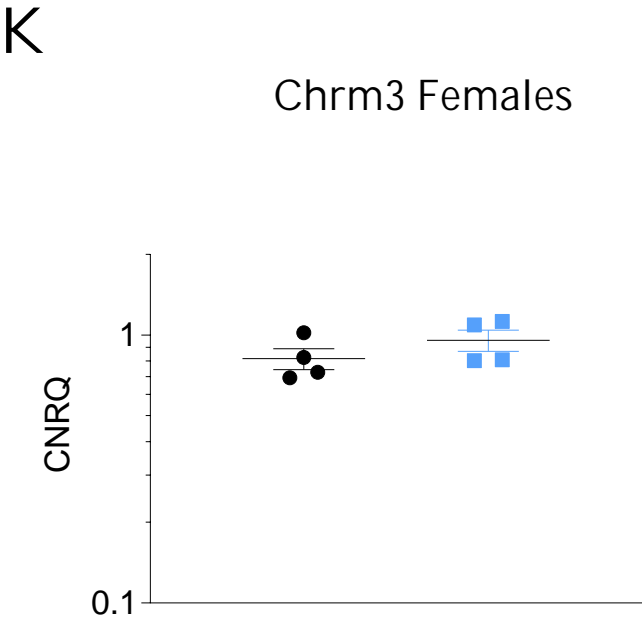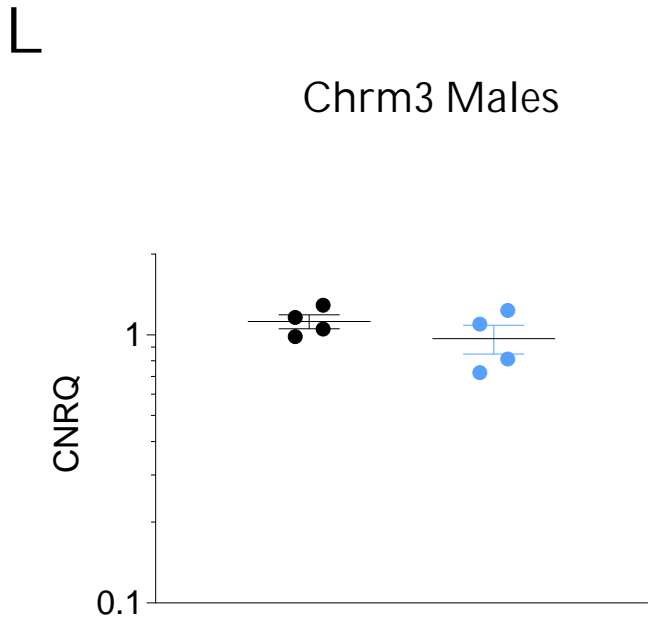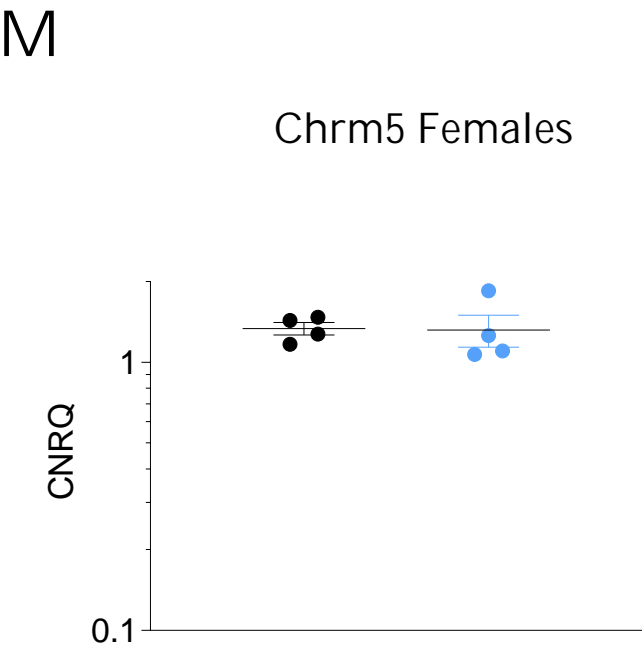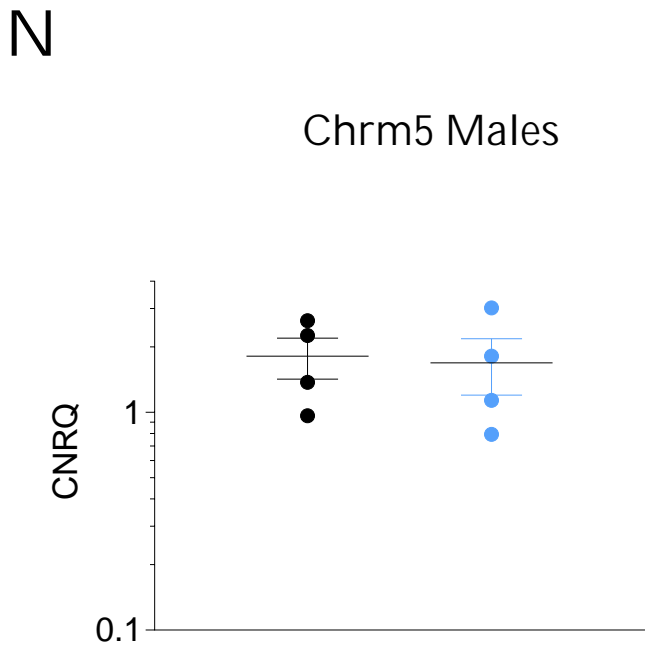

Supplement: S1 File — S1 Fig: Genotyping of APP/PS1 AD mice and WT control animals. S2 Fig: 3D image of the murine brain including the RS cortex and hippocampus (BROIs) used for transcriptome analysis in our study. S3 Fig: PCA of transcriptomes from the RS cortex and hippocampus of WT controls and APP/PS1 AD mice of both sexes. S4 Fig: Hierarchical clustering of transcriptome data from the RS cortex and hippocampus of WT control and APP/PS1 AD mice of both sexes. S5 Fig: Bar diagrams of the top 30 candidates of DEGs with highest significant FCs (FC > 1.5 and FC < -1.5, p < 0.05). S6 Fig: Pathway analysis of intersectional and signature gene sets in APP/PS1 subgroups. S7 Fig: Comparative qPCR analysis of selected gene transcript levels from the hippocampus of female and male APP/PS1 AD with 5XFAD mice. S1 Table: PCR reaction set-up using PCR Mastermix and genomic DNA. S2 Table: Materials used for one-color microarray-based gene expression data collection. S3 Table: Software used for one-color microarray-based gene expression data collection. S4 Table: Details on genes, forward and reverse primer sequences and annealing temperatures relevant for qPCR experimentation. S5 Table: Characteristics of DEGs in the RS cortex of female APP/PS1 AD mice. S6 Table: Characteristics of DEGs in the hippocampus of female APP/PS1 AD mice. S7 Table: Characteristics of DEGs in the RS cortex of male APP/PS1 AD mice. S8 Table: Characteristics of DEGs in the hippocampus of male APP/PS1 AD mice. S9 Table: Venn analysis of DEGs in the RS cortex and hippocampus of female APP/PS1 AD mice. S10 Table: Venn analysis of DEGs genes in the RS cortex and hippocampus of male APP/PS1 AD mice. S11 Table: Venn analysis of DEGs in the RS cortex of male and female APP/PS1 AD mice. S12 Table: Venn analysis of DEGs in the hippocampus of male and female APP/PS1 AD mice. S13 Table: Differentially regulated l(i)ncRNAs in APP/PS1 AD vs. WT mice. S14 Table: qPCR-based FC analysis of selected genes in the hippocampus of APP/PS1 AD vs. [file pone.0296959.s001.zip › Supplementary Files_R1/Supplementary Figure 7_I_N_qPCR.pdf]
