# Supplementary material for: Sex- and region-specific cortical and hippocampal whole genome transcriptome profiles from control and APP/PS1 Alzheimer’s disease mice
Source: PLoS One. 2024 Feb 7;19(2):e0296959. doi: 10.1371/journal.pone.0296959 (PMC10849391; doi:10.1371/journal.pone.0296959)
Supplement: Attachment — Submitted filename: peer review.docx [file pone.0296959.s002.docx]

Summary

The authors of this manuscript has performed bulk microarray studies in two specific regions of the brain in WT and APP/PS1 mouse model of Alzheimer’s Disease. Overall, DEGs and GO analysis indicate upregulation of different functional responses that largely belong to the inflammatory pathways. Nevertheless, the authors highlight several unique genes and gene pathways that may be attributed to region and sex-specific differences. Some of the findings are unique and interesting and should further be highlighted in the results and discussion.

Please note that I am unable to open the supplementary files as they are corrupted.

Figure 1A mislabeled axes typo

Figure 2 volcano plots axes should not be compressed at the top with individual DEGs labeled in the graph. At least the top targets. Otherwise the graphs are not informative. Some of the labels are truncated and not assembled properly.

Pg. 14 text and also throughout the entire results section. The target genes do not have to be completely listed in the text. It makes it incredibly laborious to read. Also these targets are then shown in the table once again. Both sources of information appear redundant. Much better if added as supplementary tables and the results sections is written in a more concise manner to highlight major differences that are of interest instead of just regurgitating all the outputs from the figures which the readers can already see for themselves. In addition, figures looks disorganized and cut and pasted directly from screenshots or generated by other software. They are pixelated and of different sizes. The entire organization of the paper makes it challenging to read.

Pg. 29. Line 865: There is no Figure 4E

Fig. 5E: Any reason why DEGs are entities?

Pg. 37. Line1014: Citation of online resources should be edited. Notations does not look accurate.

Section 3.8. It is not clear to me at all what the results are. It is merely stating what are on supplementary information without elaborating at all, the significance of the findings. What is the major conclusion for lincRNA?

Section 3.9 The rationale for choosing these gene targets for qPCR is not well explained. Why are these target genes chosen? Is this based on previous results. You mentioned in the text “similar phenotypic features”. What are these features that have been shown that is relevant for your target genes? Why not look at more interesting sex-specific differences in gene expression?

Table 3: Consider condensing the table. It does not need to take up 3 pages. It is also hard to compare the data when it is scattered across multiple pages. Consider reordering the gene list by degree of overlap across genotypes. Alphabetical ordering contributes nothing to hierarchy.

Consider reorganizing the discussion section. At the moment, the discussion reads like a literature review of the target genes. There are interesting discoveries your studies have revealed, for example, specific sex-related genes that appear to be region specific, like ICAM. Those should be highlighted and stressed. Perhaps even performed qPCR for verification. In any case, these interesting findings are embedded in a wall of text. That microglial/phagocytotic/neutrophil pathways are shared and activated is not surprising and should not be reiterated.
