# Supplementary material for: Sex- and region-specific cortical and hippocampal whole genome transcriptome profiles from control and APP/PS1 Alzheimer’s disease mice
Source: PLoS One. 2024 Feb 7;19(2):e0296959. doi: 10.1371/journal.pone.0296959 (PMC10849391; doi:10.1371/journal.pone.0296959)
Supplement: Attachment — Submitted filename: PLOS One.docx [file pone.0296959.s003.docx]

In this article entitled “Sex- and region-specific cortical and hippocampal whole genome transcriptome profiles from control and APP/PS1 Alzheimer’s disease mice”, Papazoglou et al have showed differentially expressed gene signatures from retrosplenial cortex and hippocampus of age-matched male and female APP/PS1 AD mice and control animals. They found genes for activation of microglial, astrocytic and neutrophilic cells, innate immune response/immune effector response and neuroinflammation, phagosome and proteasome activation, and synaptic transmission were upregulated, while the genes for synaptic vesicle docking/fusion machinery, synaptic transmission, rRNA processing, ubiquitination and proteasome degradation, histone modification and cellular senescence were downregulated.

Although sex dependent transcriptome profiling is not very novel in hippocampus, and there are a few studies which have done transcriptome profiling in a sex dependent manner. Although the study is not completely novel it is interesting in terms of the systematic profiling of both regions cortex and hippocampus in a sex dependent fashion.

Please address these concerns

1) Line 111 suggest Sudden death in APP/PS1 mice peaks around 3–4 months of age, This is not commonly seen at 3-4 months there are many studies that reported is the time when the synaptic deficits starts to appear.
2) Please discuss the impact of the study based on an region and sex specific changes in transcriptome on the pathogenesis and prevention.
3) The role of immune mechanisms in the etiology of AD is well studied, so discuss in terms of comparisons of region and sex.
4) Are these upregulated genes pathogenic or is there any neuroprotective geneS?
5) In synaptic plasticity upregulation of certain genes can lead to increase in synaptic transmission, while upregulation of some genes can lead to downregulation of synaptic plasticity. Please discuss.
6) APP/PS1 is a model where pathology also involves APP over production, so how would you negate its effects?
7) As some DEGs are exclusively upregulated or downregulated in males or females, this could be a target for gender specific medicine.Please discuss.
